# The Transcriptional Profile of Mesenchymal Stem Cell Populations in Primary Osteoporosis Is Distinct and Shows Overexpression of Osteogenic Inhibitors

**DOI:** 10.1371/journal.pone.0045142

**Published:** 2012-09-24

**Authors:** Peggy Benisch, Tatjana Schilling, Ludger Klein-Hitpass, Sönke P. Frey, Lothar Seefried, Nadja Raaijmakers, Melanie Krug, Martina Regensburger, Sabine Zeck, Thorsten Schinke, Michael Amling, Regina Ebert, Franz Jakob

**Affiliations:** 1 Orthopedic Center for Musculoskeletal Research, University of Wuerzburg, Wuerzburg, Germany; 2 Institute of Cell Biology (Tumor Research), University Hospital Essen, Essen, Germany; 3 Department of Trauma, Hand-, Plastic- and Reconstructive Surgery, University Hospital of Wuerzburg, Wuerzburg, Germany; 4 Department of Osteology and Biomechanics, University Medical Center Hamburg Eppendorf, Hamburg, Germany; Georgia Health Sciences University, United States of America

## Abstract

Primary osteoporosis is an age-related disease characterized by an imbalance in bone homeostasis. While the resorptive aspect of the disease has been studied intensely, less is known about the anabolic part of the syndrome or presumptive deficiencies in bone regeneration. Multipotent mesenchymal stem cells (MSC) are the primary source of osteogenic regeneration. In the present study we aimed to unravel whether MSC biology is directly involved in the pathophysiology of the disease and therefore performed microarray analyses of hMSC of elderly patients (79–94 years old) suffering from osteoporosis (hMSC-OP). In comparison to age-matched controls we detected profound changes in the transcriptome in hMSC-OP, e.g. enhanced mRNA expression of known osteoporosis-associated genes (*LRP5*, *RUNX2*, *COL1A1*) and of genes involved in osteoclastogenesis (*CSF1*, *PTH1R*), but most notably of genes coding for inhibitors of WNT and BMP signaling, such as Sclerostin and MAB21L2. These candidate genes indicate intrinsic deficiencies in self-renewal and differentiation potential in osteoporotic stem cells. We also compared both hMSC-OP and non-osteoporotic hMSC-old of elderly donors to hMSC of ∼30 years younger donors and found that the transcriptional changes acquired between the sixth and the ninth decade of life differed widely between osteoporotic and non-osteoporotic stem cells. In addition, we compared the osteoporotic transcriptome to long term-cultivated, senescent hMSC and detected some signs for pre-senescence in hMSC-OP.

Our results suggest that in primary osteoporosis the transcriptomes of hMSC populations show distinct signatures and little overlap with non-osteoporotic aging, although we detected some hints for senescence-associated changes. While there are remarkable inter-individual variations as expected for polygenetic diseases, we could identify many susceptibility genes for osteoporosis known from genetic studies. We also found new candidates, e.g. *MAB21L2*, a novel repressor of BMP-induced transcription. Such transcriptional changes may reflect epigenetic changes, which are part of a specific osteoporosis-associated aging process.

## Introduction

Primary osteoporosis is a polygenetic disease characterized by low bone mineral density and microarchitectural deteriorations, leading to an increased risk of fragility fractures of vertebrae, femoral neck and other typical localizations of lower incidence [1]. Advanced age, gender and immobilization are major risk factors for developing osteoporosis besides a series of other contributors, e.g. diminished sex steroid production in elderly individuals and after menopause [2], [3]. During the first decades of osteoporosis research the main focus has been the imbalance of bone resorption over bone formation as a consequence of pathologically enhanced osteoclast development and function [4]. Hence, antiresorptive treatment, targeting mature osteoclasts and the osteoclastogenesis promoting RANK (Receptor Activator of NF-κB)/RANKL (RANK ligand) pathway has evolved as a standard therapy over the last decades [1], [5]. In contrast, research on presumptive deficiencies in bone anabolism has been relatively neglected. Little is known about the impact of bone forming osteoblasts on the pathophysiology of osteoporosis in humans, although evidence was found for reduced activity [6] and enhanced apoptosis [7], [8]. Osteoblasts derive from mesenchymal stem cells (MSC), which can also give rise to other mesodermal cell types, such as adipocytes, chondrocytes and fibroblasts [9]. Despite of MSC as the source for bone regeneration it is currently unknown if intrinsic deficiencies in these cells contribute to osteoporotic bone loss.

Three major signaling pathways have been identified to govern bone regeneration with an intense intracellular crosstalk: a) Bone morphogenetic protein (BMP) signaling, b) WNT signaling and c) signaling through parathyroid hormone receptor (PTH1R) activation. Recent research has highlighted the relevance of inhibitors of the respective pathways for the regulation of bone mass and thereby suggested new targets for the treatment of bone loss [1].

BMP proteins belong to the TGFβ superfamily and activation of BMP receptors leads to induction of transcription through either MAP kinase signaling or phosphorylation of SMAD1/5/8 proteins [10], [11]. Signaling through BMP proteins is regulated by either extracellular antagonists such as Noggin and Gremlin [12], [13] or by intracellular inhibitors, e.g. inhibitory SMAD proteins [14] or nuclear MAB21L2 (Mab-21-like 2), a recently discovered BMP4 inhibitor [15].

Depending on coreceptors WNT signaling can be divided into canonical and non-canonical pathways. Canonical signaling is induced by binding of WNT ligands to the receptors of the Frizzled (FZD) family and LRP5/6 coreceptors, which results in activation of WNT-specific gene transcription by stabilization and nuclear translocation of β-Catenin. Non-canonical WNT signaling is transduced through FZD and ROR2/RYK coreceptors, which leads to the activation of G-protein or Ca^2+^-dependent cascades [16]. In MSC canonical signaling through WNT2, WNT3 or WNT3a induces proliferation and keeps the cells in an undifferentiated state, whereas non-canonical signaling, e.g. by WNT5a, WNT5b or WNT11, supports osteogenesis [17], [18], [19].

The osteocyte-specific factor Sclerostin (*SOST*) was described as an inhibitor of canonical WNT signaling, whereas there is ongoing discussion about its putative inhibitory effect on BMP signaling [20], [21]. Sclerostin leads to reduced bone formation [22] and loss of function mutations are responsible for the high bone mass syndromes Van Buchem disease and sclerosteosis [23]. A neutralizing antibody against Sclerostin is a new, upcoming therapeutic treatment for osteoporosis [1], [24].

Intermittent treatment with parathyroid hormone (PTH) is another therapeutical approach for osteoporosis and activates the third major signaling pathway in bone regeneration. However, continuous activation of PTH receptor has negative effects on bone homeostasis because subsequently enhanced RANKL expression on maturing osteoblasts stimulates osteoclast formation and bone resorption [25], [26].

Interestingly, the genetic loci of proteins involved in the signaling pathways mentioned above, e.g. LRP5, LRP4, Sclerostin, PTH, BMPs or BMP receptor BMPR1B, have already been linked to the polygenetic nature of primary osteoporosis by whole-genome association studies and meta-analyses [27], [28], [29], [30].

Besides genetic predisposition, advanced age is another strong risk factor for developing osteoporosis with adult stem cells being the restrictive parameter for unlimited tissue regeneration. *In vitro*, cells exhibit limited dividing capacity and enter replicative senescence, a state of irreversible G1 phase arrest, after about 50 population doublings [31], [32]. It is caused by multiple factors like telomere shortening, oxidative stress, deficiencies in DNA repair and epigenetic changes. Currently it is still controversial, whether clock-driven, organismic aging is caused by the loss of self-regeneration due to replicative senescence of stem cells or by extrinsic environmental factors [33].

The impact of presumptive deficiencies of hMSC in elderly, osteoporotic patients has not been studied intensely yet and to our knowledge changes at the gene expression level have not been examined before. Therefore, we performed microarray analyses of hMSC of elderly donors with and without osteoporosis to detect disease-associated changes in gene expression. With osteoporosis being an age-related disease, we also investigated the impact of aging on hMSC in general by analyzing the transcriptome of *in vivo*-aged and *in vitro*-aged, senescent cells. We discovered that hMSC of patients suffering from severe osteoporosis display a disease-specific gene expression pattern that is distinct from the effects of organismic aging *per se*. Besides the induced expression of inhibitors of bone formation we detected promising new candidate genes for osteoporosis and even found evidence for reduced stem cell function.

## Results

### Osteoporosis-induced changes in gene expression

**Figure 1:**
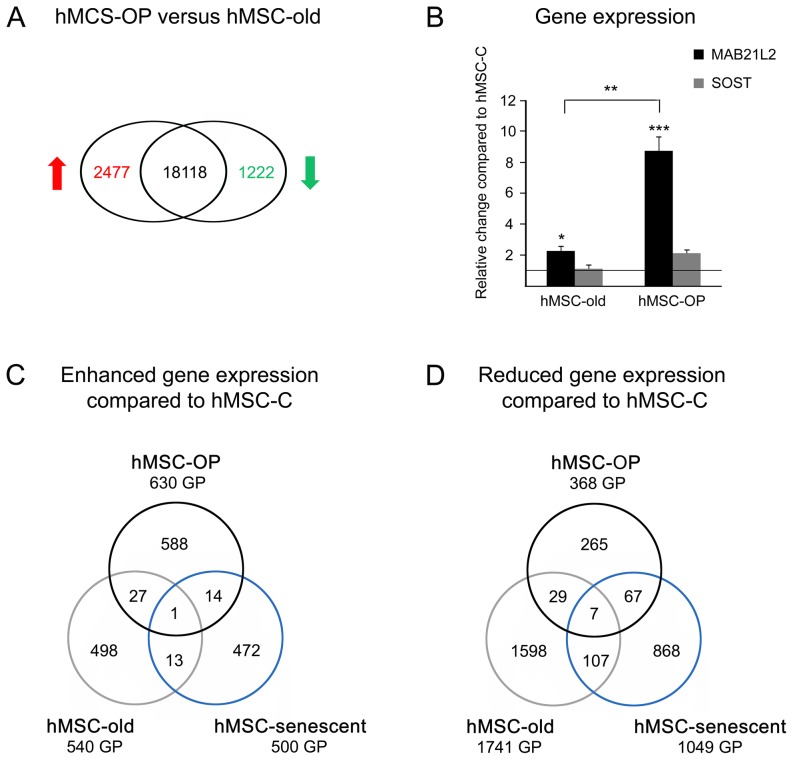
Differential gene expression of osteoporotic and aged hMSC. (A) Microarray comparison of hMSC-OP of elderly patients suffering from primary osteoporosis to age-matched control group hMSC-old. The numbers indicate the number of gene products with enhanced expression (red) and reduced expression (green) in hMSC-OP (for gene names see Table S1). Black numbers mark expressed gene products without significant change in expression. (B) Quantitative PCR of relative change in gene expression of *SOST* (Sclerostin) and *MAB21L2* (Mab-21-like 2) in hMSC-old and osteoporotic hMSC-OP in comparison to hMSC-C. Complementary DNA of hMSC-OP of patients suffering from primary osteoporosis (n = 12, including 4 samples also used for microarray hybridization; age 84.2±6.3), hMSC-old from non-osteoporotic donors of advanced age (n = 13, including 4 samples also used for microarray hybridization; age 82.3±3.6) and hMSC-C of middle-aged, healthy donors (n = 11, including one sample also used for microarray hybridization; age 41.6±2.6) was used. Asterisks indicate significant differences as analyzed by Mann-Whitney U test (*p<0.05, **p<0.01, ***p<0.001). (C–D) Comparison of differential gene expression patterns of hMSC-OP, hMSC-old and hMSC-senescent when compared to hMSC-C of middle-aged, healthy donors by microarray analyses. The numbers indicate the number of gene products (GP) with significantly enhanced (C) or reduced (D) expression, respectively (for gene names see Table S2).

In this study, we compared the transcriptome of hMSC from 5 patients (79–94 years old) suffering from primary osteoporosis (hMSC-OP) with hMSC of the age-matched control group (hMSC-old; donor age 79–89 years) (Table 1). Genome-wide gene expression patterns were examined by employing microarray hybridizations; the obtained data was compared by SAM method (GEO accession number GSE35958). Fold changes (FC) in gene expression were regarded as significant at a threshold of at least 2fold and a false discovery rate (FDR) of less than 10%. We detected 2477 gene products with higher and 1222 gene products with reduced expression in osteoporotic hMSC-OP in comparison to non-osteoporotic hMSC-old (Figure 1A, Table S1).

**Table 1:** Human MSC populations used for microarray hybridization.

hMSC group	hMSC-C	hMSC-OP	hMSC-old	hMSC-senescent
Donors (n)	5	5	4	5
Average donor age (years)	57.6±9.56	86.2±5.89	81.75±4.86	56.4±8.96
Donors showed signs of osteoporosis	no	yes	no	no
Gender	4x f, 1x m	5x f	3x f, 1x m	3x f, 2x m
RNA of hMSC used in passage	4x P1, 1x P2	4x P1, 1x P2	P1	Px

hMSC-C = control hMSC; hMSC-OP = osteoporotic hMSC; hMSC-old = hMSC of non-osteoporotic, elderly donors; hMSC-senescent = long term-cultivated hMSC in the state of replicative senescence; standard deviations are indicated by ±; n = number; f = female; m = male; P = passage; Px = senescent passage.

Osteoporosis as a polygenetic disease has been studied intensively on gene level, resulting in the detection of gene loci and polymorphisms associated with low bone mineral density (BMD), osteoporosis and fracture risk. In contrast to these approaches, our data represents the effects of both genetic and epigenetic changes in hMSC during the development of osteoporosis.

To see if our results coincide at least partly with the genes associated to BMD by specific single nucleotide polymorphisms (SNP) and copy number variations, we searched the NCBI data base for genome-wide association studies, meta-analyses and candidate gene association studies. The genes listed in these studies were compared to all gene products differentially expressed in the approach hMSC-OP versus hMSC-old.

We identified enhanced expression of 39 genes in hMSC-OP and reduced expression of 16 genes that are already described as reliable or promising candidates for osteoporosis, including susceptibility genes like *LRP5*, *SPP1* (Osteopontin), *COL1A1* and *SOST* (Table 2).

**Table 2:** Differentially expressed genes in hMSC-OP in comparison to hMSC-old with known association to BMD or fracture risk.

Symbol	Gene name	Probeset ID	FC	FDR (%)	Reference
**Enhanced expression in hMSC-OP**					
AAS	achalasia, adrenocortical insufficiency, alacrimia	218075_at	3.57	0.08	[58]
ANKH	ankylosis, progressive homolog	223094_s_at	3.60	0.26	[27]
		1560369_at	3.40	0.35	
ARHGAP1	Rho GTPase activating protein 1	216689_x_at	7.06	0.00	[58]
ASPH	aspartate beta-hydroxylase	205808_at	7.80	0.00	[28]
ASXL2	additional sex combs like 2	1555266_a_at	9.07	0.00	[59]
		218659_at	2.39	0.33	[59]
CAMK1G	calcium/calmodulin-dependent protein kinase IG	217128_s_at	2.45	0.44	[60]
CKAP5	cytoskeleton associated protein 5	1555278_a_at	2.78	0.26	[58]
COL1A1	collagen, type I, alpha 1	217430_x_at	22.19	0.00	[27]
CRTAP	cartilage associated protein	1554464_a_at	2.44	0.86	[61]
CUL7	cullin 7	203558_at	4.09	0.00	[28]
		241747_s_at	3.41	0.35	
		36084_at	3.07	0.08	
DBP	D site of albumin promoter (albumin D-box) binding protein	209782_s_at	2.87	2.45	[27]
DIO2	deiodinase, iodothyronine, type II	231240_at	4.48	0.00	[62]
DMWD	dystrophia myotonica, WD repeat containing	213231_at	4.11	0.26	[59]
		33768_at	3.11	0.23	
		1554429_a_at	2.71	0.19	
E2F7	E2F transcription factor 7	241725_at	2.21	0.86	[63]
ERCC2	excision repair cross-complementing rodent repair deficiency, complementation group 2	213468_at	2.97	0.08	[59]
ERLIN1	ER lipid raft associated 1	202444_s_at	4.33	0.08	[58]
FOXC2	forkhead box C2 (MFH-1, mesenchyme forkhead 1)	214520_at	6.24	0.00	[27]
FZD1	frizzled homolog 1	204452_s_at	2.85	0.35	[27]
GSR	glutathione reductase	205770_at	2.10	2.06	[64]
GSTM1	glutathione S-transferase mu 1	204550_x_at	3.57	0.19	[64]
		215333_x_at	3.19	0.35	
HMGA2	high mobility group AT-hook 2	1558682_at	3.33	0.63	[27]
HSD11B1	hydroxysteroid (11-beta) dehydrogenase 1	205404_at	2.15	9.00	[65]
IBSP	integrin-binding sialoprotein	207370_at	9.41	0.00	[29], [66]
		236028_at	4.88	0.26	
KPNA4	karyopherin alpha 4 (importin alpha 3)	209653_at	4.04	0.19	[58]
LRP5	low density lipoprotein receptor-related protein 5	209468_at	3.54	0.33	[27]
MRPL2	mitochondrial ribosomal protein L2	218887_at	2.07	0.73	[28]
ND2	mitochondrially encoded NADH dehydrogenase 2 (MTND2)	1553551_s_at	2.66	0.14	[67]
PDE7B	phosphodiesterase 7B	220343_at	3.39	0.55	[28]
PRR16	proline rich 16	1554867_a_at	2.12	2.72	[68]
PTPRD	protein tyrosine phosphatase, receptor type, D	213362_at	3.07	3.21	[69]
		205712_at	2.91	1.67	
RARG	retinoic acid receptor, gamma	204189_at	3.85	0.26	[58]
RERE	arginine-glutamic acid dipeptide (RE) repeats	221643_s_at	5.26	0.00	[27]
RUNX2	runt-related transcription factor 2	216994_s_at	11.86	0.00	[27]
SIX5	SIX homeobox 5	229009_at	2.69	0.26	[59]
SOST	sclerostin	223869_at	4.60	1.00	[27]
SOX4	SRY (sex determining region Y)-box 4	201418_s_at	2.23	2.72	[29]
SP1	Sp1 transcription factor	1553685_s_at	4.19	0.08	[58]
		214732_at	3.49	0.35	
SPP1	secreted phosphoprotein 1	209875_s_at	2.53	4.15	[70]
TBC1D1	TBC1 (tre-2/USP6, BUB2, cdc16) domain family, member 1	1568713_a_at	4.11	0.35	[58]
**Reduced expression in hMSC-OP**					
CTNNB1	catenin (cadherin-associated protein), beta 1, 88 kdf	201533_at	0.44	1.67	[66]
		1554411_at	0.23	0.23	
FAM3C	family with sequence similarity 3, member C	236316_at	0.44	9.00	[27]
FBXL17	F-box and leucine-rich repeat protein 17	227203_at	0.32	0.55	[68]
FGF14	fibroblast growth factor 14	230231_at	0.50	5.82	[68]
FGFR2	fibroblast growth factor receptor 2	208229_at	0.20	0.73	[70]
IFNAR2	interferon (alpha, beta and omega) receptor 2	204786_s_at	0.43	2.72	[71]
ITIH5	inter-alpha (globulin) inhibitor H5	1553243_at	0.30	2.72	[69]
JAG1	Jagged 1 (Alagille syndrome)	231183_s_at	0.33	2.06	[72]
NHS	Nance-Horan syndrome	242800_at	0.38	2.72	[28]
PLCL1	phospholipase C-like 1	205934_at	0.25	5.82	[27]
PTN	pleiotrophin	211737_x_at	0.49	6.81	[70]
		209465_x_at	0.46	5.82	
PTPRM	protein tyrosine phosphatase, receptor type, M	1555578_at	0.47	6.81	[28]
RAPGEF4	Rap guanine nucleotide exchange factor (GEF) 4	205651_x_at	0.46	3.67	[69]
SERPINE2	Serpin peptidase inhibitor, clade E, member 2	227487_s_at	0.44	4.93	[28]
SFRP4	secreted frizzled-related protein 4	204052_s_at	0.26	3.67	[73]
SMAD1	SMAD family member 1	227798_at	0.47	3.21	[71]

FC = fold change; FDR = false discovery rate.

### Effects of osteoporosis are independent of clock-driven aging

One of the main risk factors for developing primary osteoporosis is advanced age. Therefore, in the next step, we focused on gene expression patterns that were identical in hMSC-OP of elderly patients suffering from osteoporosis and hMSC-old of non-osteoporotic, elderly donors. As a new control group for microarray comparisons, we used hMSC of middle-aged donors (hMSC-C; donor age 42–67 years).

In the comparison of hMSC-OP versus hMSC-C (GEO accession number GSE35956) we detected 630 gene products with higher and 368 gene products with reduced expression due to osteoporosis and advanced donor age. By comparing hMSC-old with hMSC-C (GEO accession number GSE35955) we obtained gene expression changes due to advanced age *per se* and found enhanced expression of 540 gene products and decreased expression of 1741 gene products in hMSC-old.

Due to the fact that we used hMSC-C as a control in both SAM approaches we could compare the differentially gene expression patterns of hMSC-OP and hMSC-old (Figure 1C and D). Surprisingly we detected a minority of 28 gene products with enhanced and 36 gene products with reduced expression in both approaches (for gene names see Table S2).

One of the genes that was enhanced expressed due to osteoporosis but also due to advanced age was *MAB21L2* with FC[hMSC-old versus hMSC-C] = 2.7 and FC[hMSC-OP versus hMSC-C] = 14.4. By performing qPCR analysis with up to 13 samples per hMSC group we confirmed that the expression of *MAB21L2* is significantly higher in osteoporotic hMSC-OP than in hMSC-old when compared to hMSC-C of the middle-aged control group (Figure 1B).

In contrast, *SOST*, the gene coding for Sclerostin, is not associated with advanced age (no significant FC), but both microarray analysis (FC[hMSC-OP versus hMSC-C] = 7.3) and qPCR revealed the enhanced expression of the gene in osteoporotic hMSC-OP (Figure 1B).

### Osteoporotic stem cells show few signs of replicative senescence

Because donors of osteoporotic cells were of highly advanced age (79+ years old) and due to the hypothesis that aging is caused by stem cells losing their self-renewal capacity due to replication limits, we investigated whether hMSC-OP showed any signs of replicative senescence. Therefore, we performed long-term cultivation of hMSC from healthy donors of medium age (42–64 years old) until they entered senescence (hMSC-senescent), proved by proliferation stop and positive senescence-associated β-galactosidase staining (data not shown).

Microarray analyses of hMSC in senescent passage Px revealed 500 gene products with enhanced and 1049 gene products with reduced expression when compared to the previously used control group hMSC-C of early passages (GEO accession number GSE35957).

By using hMSC-C as control cells for all three SAM datasets we could compare the differential gene expression pattern of hMSC-OP, hMSC-old and hMSC-senescent to find parallels in gene expression. We detected small overlap for gene products with enhanced expression when comparing hMSC-OP with hMSC-senescent (15) and hMSC-old with hMSC-senescent (14) (Figure 1C). More senescence-associated gene products were found reduced expressed in hMSC-old (114) and hMSC-OP (74) (Figure 1 D, Table S2). Few genes were differentially expressed in all three datasets: *TMEFF1* showed induced expression, whereas *MED13L*, *ANLN*, *ZWILCH*, *CMPK2*, *DDX17*, *MCM2* and *MCM8* showed diminished expression in hMSC-OP, hMSC-old and hMSC-senescent when compared to hMSC-C.

**Figure 2:**
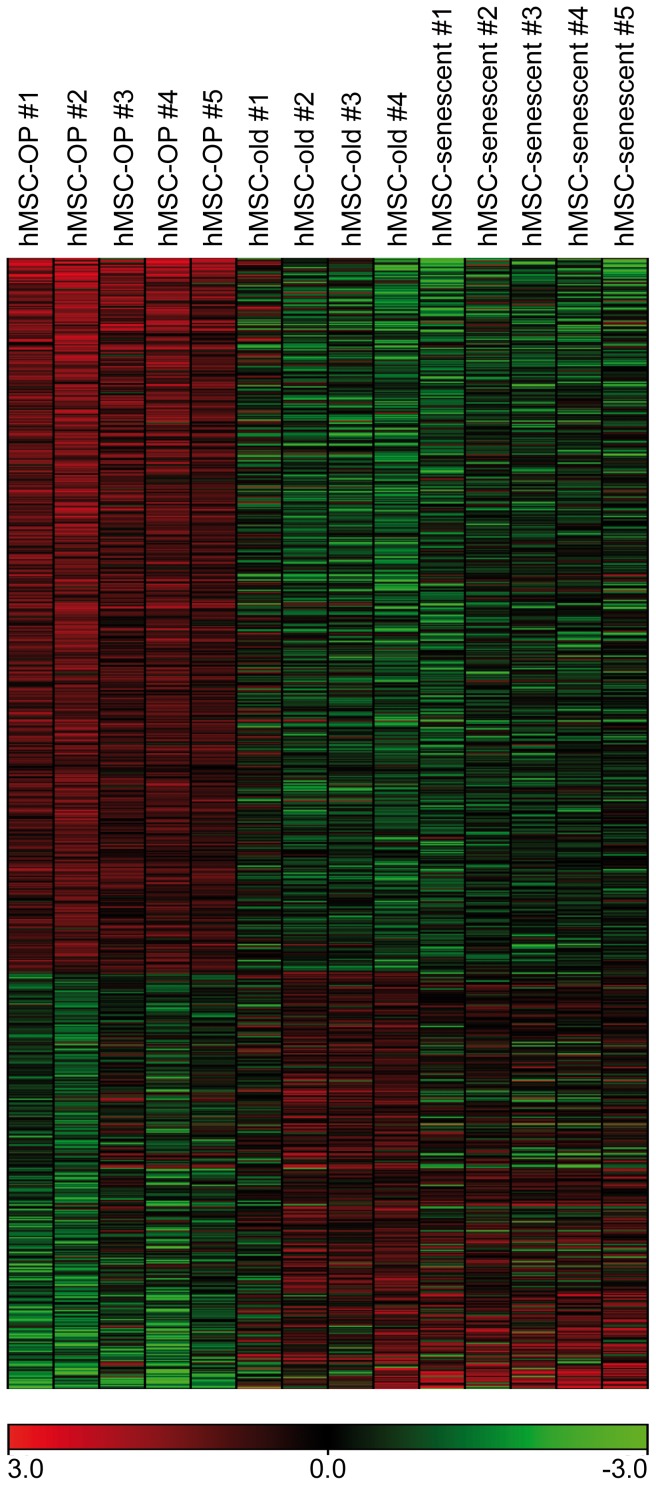
Heat map of microarray results of osteoporotic and aged hMSC. Color-coded microarray hybridization signals (green to red = low to high signals) of hMSC-OP, hMSC-old and hMSC-senescent. The 998 gene products depicted showed at least 2fold differential gene expression (630 enhanced, 368 reduced; FDR<10%) in SAM comparison of hMSC-OP versus hMSC-C (for gene names see Table S2).

By generating a heat map for gene products at least 2fold differentially expressed in hMSC-OP compared to hMSC-C we could highlight the difference between hMSC-OP, hMSC-old and hMSC-senescence (Figure 2). Osteoporotic cells exhibit a distinct gene expression profile independent of both clock-driven aging and cellular aging.

### Relevance of transcriptional changes for stem cell function

To unravel if changes in gene expression profile could cause deficiencies in cellular processes we carried out gene function and pathway identifications by Gene Ontology classification and by searching within the NCBI database for literature. By comparing functions of genes differentially expressed in hMSC-OP, hMSC-old and hMSC-senescent when compared to hMSC-C we detected differences in the effect of osteoporosis, age and senescence on stem cell characteristics. Hereby we focused on genes with known relevance in the following 4 processes: (1) osteoblastogenesis, (2) osteoclastogenesis, (3) proliferation and (4) DNA repair (Table 3). These categories play important roles in sustaining bone homeostasis by influencing bone formation, bone resorption and self-renewal of stem cells.

**Table 3:** Functional clustering of differentially expressed genes of hMSC-OP, hMSC-old and hMSC-senescent when compared to hMSC-C.

Symbol	Gene name		hMSC-OP	hMSC-old		hMSC-senescent			Reference		
**(1) Osteoblastogenesis**											
**positive**											
PTH1R	parathyroid hormone 1 receptor		**↑**						[74]		
IBSP	integrin-binding sialoprotein		**↑**						[75]		
INHA	inhibin, alpha		**↑**						[76]		
IGFBP2	insulin-like growth factor binding protein 2		**↑**						[77]		
IGF2	insulin-like growth factor 2		**↑**			**↓**			[77]		
VEGFB	vascular endothelial growth factor B		**↑**	**↓**					[78]		
VEGFA	vascular endothelial growth factor A		**↑**	**↓**		**↓**			[78]		
FOXC2	forkhead box C2 (MFH-1, mesenchyme forkhead 1)		**↑**	**↓**					[79]		
COL1A1	collagen, type I, alpha 1			**↓**					[75]		
RUNX2	runt-related transcription factor 2			**↓**					[75]		
ANKH*	ankylosis, progressive homolog		**↓**	**↓**					[80]		
SMAD3^B^	SMAD family member 3			**↓**					[81]		
SPP1	secreted phosphoprotein 1					**↓**			[75]		
EFNB2	ephrin-B2					**↓**			[82]		
ALPL	alkaline phosphatase, liver/bone/kidney					**↓**			[80]		
CYP2R1	cytochrome P450, family 2, subfamily R, polypeptide 1					**↓**			[80], [83]		
FOXC1	forkhead box C1			**↑**		**↓**			[84]		
IL6ST	interleukin 6 signal transducer (Oncostatin M receptor)			**↓**		**↑**			[85]		
PDGFA	platelet-derived growth factor alpha polypeptide			**↑**					[11]		
VDR	vitamin D receptor			**↑**					[80]		
FGFR2*	fibroblast growth factor receptor 2			**↑**		**↓**			[86]		
BMP6^B^	bone morphogenetic protein 6					**↑**			[11]		
ROR1^W^	receptor tyrosine kinase-like orphan receptor 1		**↓**			**↑**			[87]		
ANKRD6^W^	ankyrin repeat domain 6					**↑**			[16], [18]		
**negative**											
TGFB1	transforming growth factor, beta 1		**↑**	**↓**					[88]		
MAB21L2^B^	mab-21-like 2		**↑**	**↑**					[15]		
FST	follistatin		**↑**	**↑**					[89], [90]		
FSTL3	follistatin-like 3		**↑**						[91]		
KREMEN1^W^	kringle containing transmembrane protein 1		**↑**						[38]		
SOST^W^ ^B^	sclerostin		**↑**						[23]		
FGFR1	fibroblast growth factor receptor 1		**↑**						[92]		
IGFBP5	insulin-like growth factor binding protein 5		**↑**			**↑**			[11]		
IGFBP4	insulin-like growth factor binding protein 4			**↓**					[11]		
EGFR	epidermal growth factor receptor			**↓**					[93]		
GREM2^B^	gremlin 2, cysteine knot superfamily, homolog		**↓**	**↓**					[94]		
NOG^B^	noggin			**↑**					[11]		
CTNNB1^W^	catenin, beta 1			**↑**					[17], [18]		
SFRP4^W^	secreted frizzled-related protein 4					**↑**			[95]		
WNT2^W^	wingless-type MMTV integration site family, member 2					**↑**			[17], [18]		
WNT3^W^	wingless-type MMTV integration site family, member 3					**↑**			[17], [18]		
**(2) Osteoclastogenesis**											
**positive**											
PTH1R	parathyroid hormone 1 receptor		**↑**						[25]		
CSF1	colony stimulating factor 1		**↑**						[4]		
PTGS2	prostaglandin-endoperoxide synthase 2		**↑**			**↓**			[96]		
IGF2	insulin-like growth factor 2		**↑**			**↓**			[97]		
TNFSF11	tumor necrosis factor superfamily, member 11					**↓**			[98]		
SPP1	secreted phosphoprotein 1					**↓**			[99]		
IL7	interleukin 7					**↓**			[100]		
THBS1	thrombospondin 1					**↑**			[101]		
IL1A*	interleukin 1, alpha			**↓**		**↑**			[67]		
TNFSF10	tumor necrosis factor superfamily, member 10			**↓**		**↓**			[102]		
TGFB2	transforming growth factor, beta 2			**↓**		**↓**			[103]		
VEGFA	vascular endothelial growth factor A		**↑**	**↓**		**↓**			[46]		
VEGFB	vascular endothelial growth factor B		**↑**	**↓**					[46]		
TGFB1	transforming growth factor, beta 1		**↑**	**↓**					[47]		
RUNX2	runt-related transcription factor 2			**↓**					[104]		
**negative**											
TNFRSF11B	tumor necrosis factor receptor superfamily, member 11b						**↑**		[98]		
FSTL3	follistatin-like 3		**↑**						[105]		
**(3) Proliferation**											
**positive**											
HMMR		hyaluronan-mediated motility receptor	**↓**					**↓**		[49]	
HELLS		helicase, lymphoid-specific						**↓**		[106]	
PTN		pleiotrophin						**↓**		[107]	
SOD2		superoxide dismutase 2, mitochondrial			**↓**			**↑**		[108]	
CCNB2		cyclin B2						**↓**		[109]	
CDC2		cell division cycle 2, G1 to S and G2 to M	**↓**					**↓**		[109]	
CCNA2		cyclin A2	**↓**		**↓**			**↓**		[109]	
CCNE2		cyclin E2	**↓**		**↓**			**↓**		[109]	
CCNF		cyclin F						**↓**		[110]	
CCND1		cyclin D1						**↑**		[109]	
CCND2		cyclin D2						**↑**		[109]	
CDC25A		cell division cycle 25 homolog A						**↓**		[111]	
CDC25B		cell division cycle 25 homolog B						**↓**		[112]	
CDC25C		cell division cycle 25 homolog C						**↓**		[113]	
CDK2		cyclin-dependent kinase 2			**↓**					[109]	
**negative**											
PSG1		pregnancy specific beta-1-glycoprotein 1						**↑**		[114]	
PSG2		pregnancy specific beta-1-glycoprotein 2						**↑**		[114]	
PSG3		pregnancy specific beta-1-glycoprotein 3			**↓**			**↑**		[114]	
PSG4		pregnancy specific beta-1-glycoprotein 4						**↑**		[114]	
PSG6		pregnancy specific beta-1-glycoprotein 6						**↑**		[114]	
PSG7		pregnancy specific beta-1-glycoprotein 7						**↑**		[114]	
ARHGAP29		Rho GTPase activating protein 29	**↓**					**↑**		[107]	
CDKN2A		cyclin-dependent kinase inhibitor 2A						**↑**		[107]	
CDKN1A		cyclin-dependent kinase inhibitor 1A	**↑**							[50]	
**(4) DNA-repair**											
**positive**											
POLD1		polymerase (DNA directed), delta 1, catalytic subunit 125kDa						**↓**		[115]	
POLE2		polymerase (DNA directed), epsilon 2 (p59 subunit)	**↓**					**↓**		[115]	
POLQ		polymerase (DNA directed), theta						**↓**		[115]	
POLH		polymerase (DNA directed), eta			**↓**					[115]	
POLK		polymerase (DNA directed) kappa			**↓**					[115]	
MRE11A		MRE11 meiotic recombination 11 homolog A			**↓**					[116]	
PARP3		poly (ADP-ribose) polymerase family, member 3			**↓**					[117]	
RAD50		RAD50 homolog	**↓**							[116]	
RAD51		RAD51 homolog						**↓**		[116]	
RAD51AP1		RAD51 associated protein 1	**↓**					**↓**		[118]	
TOP2A		topoisomerase (DNA) II alpha 170 kDa	**↓**					**↓**		[119]	
EXO1		exonuclease 1						**↓**		[116]	
CHEK1		CHK1 checkpoint homolog						**↓**		[120]	
HMGB2		high-mobility group box 2						**↓**		[121]	

arrows pointing downward = at least 2fold reduced expression in comparison to hMSC-C; arrows pointing upward = at least 2fold enhanced expression in comparison to hMSC-C;

W = gene associated with WNT signaling;

B = gene associated with BMP signaling;

* = probesets that refer to the gene are not identical in the indicated comparisons.

In hMSC-OP we found enhanced expression of gene products with relevance in osteoblastogenesis by autocrine and paracrine stimulation, respectively (*PTH1R*, *IBSP*, *IGF2*, *VEGFA* and *VEGFB*). In senescent hMSC and hMSC-old we detected reduced expression of genes coding for enhancers of osteoblast differentiation and matrix mineralization (*SPP1*, *ALPL*, *EFNB2*, *COL1A1*, *RUNX2* and *ANKH*).

Genes coding for inhibitors of WNT signaling (*SOST*, *KREMEN1*) showed enhanced expression in hMSC-OP in comparison to hMSC-C, whereas activators of canonical WNT signaling that indirectly inhibit osteogenic differentiation by augmenting proliferation, were more highly expressed in *in vitro*- aged and senescent hMSC (*WNT2*, *WNT3*, *CTNNB1*). Next to *MAB21L2*, which codes for a repressor of BMP-induced transcription, another negative regulator of osteoblastogenesis was enhanced expressed in hMSC-OP and hMSC-old: Follistatin (*FST*), which is associated with inhibition of Activin.

Genes linked to bone resorption were differentially expressed in all three hMSC groups with senescent cells exhibiting strongly diminished potential for inducing osteoclastogenesis by decreased expression of secreted ligands (*TGFB*, *VEGF*, *IL7*, *IL1A*) and other stimulators like *TNFSF11* (RANKL). The gene coding for the osteoclast inhibitor Osteoprotegerin (*TNFRSF11B*) was expressed to a higher extent in hMSC-senescent. *In vivo*-aged hMSC-old showed a similar gene expression pattern whereas osteoporotic hMSC-OP revealed enhanced expression of genes indirectly (*PTH1R*, *PTGS2* and *IGF2*) as well as directly (*CSF1*, *VEGFA* and *VEGFB*) involved in promoting osteoclast formation.

By examining the expression of genes related to proliferation we found a substantial number of repressed genes that code for proteins important for cell division, like several Cyclins, CDC2 and CDC25 proteins in hMSC-senescent. Markers for cellular senescence and genes described as mediators of cell cycle were also differentially expressed in these cells, e.g. *CDKN2A* (P16), several *PSG*, *PTN*, *ARHGAP29* (*PARG1*), *HMMR* and *HELLS*. Clock-driven aging and osteoporosis showed less negative effects on proliferative capacity of stem cells, but in hMSC-OP the expression of a second well known marker of replicative senescence – besides P16 – was increased: *CDKN1A*, which codes for P21.

DNA repair is one of the reasons for cell cycle arrest at the G1, S or G2 checkpoints of mitosis to prevent the accumulation of DNA damage or mutations that could result in tumor development. Again hMSC-senescent exhibited the most severe deficiencies with a diminished expression of genes involved in DNA repair like *TOP2A*, *EXO1* and several DNA polymerases. Osteoporotic and aged hMSC showed minor changes.

## Discussion

During aging, a continuous decrease in bone mass and bone density occurs and peaks in the development of primary osteoporosis in one of three women and one of eight men over the age of 50 [2], [27]. Induced by a variety of risk factors like advanced age, loss of sex steroid production and unhealthy life style [2], [3], [34], recent research has largely unraveled the polygenetic nature and the multifaceted pathophysiology of this syndrome [27], [29], [35]. Hitherto, approaches for studying the disease mostly consisted of whole genome association studies of BMD-associated gene loci as well as of manipulating expression of candidate genes in animal models or cells *in vitro*, followed by characterization of phenotypes [36], [37], [38]. However, bone loss associated with increasing age is a continuous process not only caused by gene polymorphisms but very likely also by epigenetic modulations of gene expression changes that accompany aging [39]. So far, analyses of these changes in primary cells of osteoporotic patients or in whole bone samples have been almost neglected.

We analyzed the effect of primary osteoporosis on the source of bone regeneration and performed microarray hybridizations of hMSC of elderly patients suffering from severe osteoporosis (hMSC-OP) and of donors of advanced age without any indication for the syndrome (hMSC-old). We detected several genes connected to BMD with either reduced (16) or increased (39) expression in hMSC-OP including well-investigated susceptibility genes like *LRP5*, *SPP1* (Osteopontin), *COL1A1* and *SOST* (Table 2) [27]. The latter codes for the osteocyte-specific protein Sclerostin, which acts as a WNT antagonist and is also controversially discussed as a BMP inhibitor [20], [22], [23]. Upon release, the protein inhibits proliferation of MSC and osteoblasts, blocks osteogenic differentiation and even induces apoptosis in osteoblasts [23], [40]. Direct connections between the protein and osteoporosis have already been described: serum levels of Sclerostin were found enhanced in postmenopausal women [41] and one of the upcoming treatments for osteoporosis is the application of anti-Sclerostin-antibodies [24], [42]. It is conceivable that the premature expression of *SOST* in osteoporotic stem cells auto-inhibits proliferation and self-renewal of hMSC-OP and thereby leads to the reduced ratios of formation to resorption observed in primary osteoporosis [43].

Furthermore we also found higher expression of *MAB21L2* (Mab-21-like 2) in hMSC-OP in comparison to hMSC-old. QPCR revealed that, even though the expression was induced by advanced donor age itself, the transcription of *MAB21L2* was even more triggered in osteoporotic stem cells (Figure 1B). In *Xenopus laevis* gastrulae it was shown that MAB21L2 antagonizes the effects of BMP4 by repressing the BMP-induced gene expression. The nuclear protein binds SMAD1, the transducer of BMP2/4/7 signaling, but so far it is still unknown if MAB21L2 exerts its effects in a DNA-binding or a non-binding fashion [15]. Our data of age- and osteoporosis-induced expression of *MAB21L2* in hMSC made us hypothesize that BMP-signaling in stem cells is less effective in advanced age and even less so in primary osteoporosis due to transcriptional repression of BMP-target genes.

Despite high inter-individual variability in the gene expression level, as demonstrated in our heat map (Figure 2), we could validate the microarray results for both *SOST* and *MAB21L2* in qPCR analysis with up to 13 different hMSC-OP and hMSC-old populations. We hereby demonstrate the reliability of our microarray approach, which was performed with a comparably low number of samples. Being inhibitors of WNT and BMP signaling, our two leading candidates are major hubs in blocking differentiation programs right at the beginning. Hereby, our data support the results of Rodriguez *et al.* and Dalle Carbonare *et al*., who demonstrated *in vitro* that osteoporotic hMSC exhibit diminished osteogenic differentiation potential [44], [45]. Future research will have to unravel how many of the genes differentially expressed in osteoporotic hMSC-OP (Table S1) are downstream *SOST* or *MAB21L2* over-expression.

Furthermore, we detected indications for osteoporotic stem cells actively enhancing osteoclastogenesis and therefore bone resorption. Besides the enhanced expression of genes coding for osteoclast stimulating ligands, e.g. VEGF, TGFB and CSF1 [4], [46], [47], we also detected the osteoporosis-induced expression of Parathyroid hormone receptor *PTH1R*. Activation of PTH1R triggers osteoblast maturation and induces RANKL expression which leads to osteoclast precursor differentiation and activation [26]. The enhanced expression of osteoclastogenesis promoting factors has already been described in fragility fractured bone [48] and is in general consistent with the enhanced bone resorption described for osteoporosis [2].

Because high age is one of the main risk factors for developing osteoporosis, we tried to dissect effects of aging from effects of primary osteoporosis by using hMSC from middle-aged donors as control cells (hMSC-C) for comparisons with hMSC-OP and hMSC-old, respectively, of elderly individuals (Figure 1C and 1D, Table S2). Surprisingly, the patterns of the differential gene expression in aged and osteoporotic hMSC differed widely. Only a few gene products with identical expression profiles in hMSC-old and hMSC-OP were observed and we therefore conclude that osteoporosis-associated changes are very distinct and independent of effects of clock-driven aging. We hypothesize that donors of advanced age who suffered from osteoarthritis but not from osteoporosis, aged in a healthier way than osteoporotic patients, or *vice versa* that osteoporosis is a distinct syndrome of premature aging.

One hypothetical reason for aging is the loss of tissue regeneration due to replicative senescence of stem cells, which accumulates over time and ends in organ failure and death of the organism [33]. Due to the fact that donors of hMSC-OP were of advanced age we analyzed whether these cells exhibited signs of replicative senescence by comparing them to the gene expression pattern of long term-cultivated, senescent hMSC. Thereby we detected a small overlap of genes differentially expressed in hMSC-OP and hMSC-senescent when compared to the identical control group hMSC-C (Figure 1C and D). Despite the distinct gene expression pattern, we found some markers for replicative senescence in osteoporotic hMSC-OP, like the reduced expression of Hyaluronan receptor *HMMR*, which was described as inversely regulated to tumor suppressor P53 [49], and the induction of *CDKN1A*, which codes for P21, another inhibitor of cyclin-dependent kinases (Table 3) [50]. In contrast, analyses of non-osteoporotic hMSC-old of the age-matched donor group revealed no expression of markers for senescence and highlighted even more the differences between aging with and without primary osteoporosis. Our findings suggest that osteoporotic stem cells exhibit deficiencies in proliferation and might already be prone to a pre-senescent state. So far, reduction in proliferative activity in osteoporotic cells has only been described for osteoblasts [51], [52]. For confirmation, more detailed investigations of hMSC-OP on protein level and by proliferation or senescence studies are needed.

In summary, this study indicates that intrinsic alterations in stem cell biology are involved in the pathophysiology of osteoporosis. By microarray analyses, we detected significant differences between hMSC of elderly donors with and without osteoporosis, suggesting that primary osteoporosis causes distinct transcriptional changes, which differ from age-related changes in non-osteoporotic donors. Next to indications for a pre-senescent state we detected enhanced transcription of inhibitors of WNT and BMP signaling in osteoporotic hMSC-OP, which can lead to functional deficiencies, such as autoinhibition of osteogenic differentiation and loss of self-renewal. Our data facilitate the importance of well-known susceptibility genes of osteoporosis such as *SOST*, *COL1A1* and *LRP5*, and additionally, we detected new candidate genes for further investigations, e.g. *MAB21L2*. Our study confirms that disturbed bone homeostasis by inhibition of osteogenic regeneration is at least an equally important feature of primary osteoporosis besides enhanced bone resorption. Therefore, “inhibition of inhibitors” of bone regeneration by using, e.g. SOST antibodies, is a mechanistically plausible treatment of the syndrome and will get even more attention in the future.

## Materials and Methods

### Ethics Statement

Bone material was used under agreement of the local Ethics Committee of the Medical Faculty of the University of Wuerzburg with written informed consent of each patient.

### Cell culture

Human MSC of non-osteoporotic donors were obtained from bone marrow of femoral heads according to the described protocol [53] after total hip arthroplasty due to osteoarthritis and/or hip dysplasia. MSC of patients suffering from osteoporosis were isolated from femoral heads after low-energy fracture of the femoral neck. Additional criteria for confirming primary osteoporosis in these donors were vertebrae fractures and advanced age.

Cell culture medium, fetal calf serum (FCS), trypsin-EDTA and antibiotics were obtained from PAA Laboratories GmbH, Linz, Austria. Human MSC were selected by surface adherence and expanded in DMEM/Ham's F-12 (1∶1) medium supplemented with 10% heat-inactivated FCS, 1 U/ml penicillin, 100 µg/ml streptomycin and 50 µg/ml L-ascorbic acid 2-phosphate (Sigma Aldrich GmbH, Schnelldorf, Germany).

For long term cultivation, cells were expanded at 70–90% confluence by trypsinization with 1× trypsin-EDTA and reseeding in a ratio of 1∶3. This procedure was repeated for up to x passages when the hMSC did not become confluent within 3 weeks due to replicative senescence.

### RNA isolation

At 80–90% confluence human MSC monolayers were lysed directly in the cell culture flask in passage (P) 1 or 2 and the last, senescent passage Px, respectively. Total RNA was isolated using the NucleoSpin RNA II Purification Kit (Macherey-Nagel, Düren, Germany) according to the manufacturer's instructions including DNase digestion.

### Microarray analysis

For microarray analyses total RNA of hMSC-C, hMSC-senescent and hMSC-OP (Table 1) was amplified and labeled according to the GeneChip One-Cycle cDNA Synthesis Kit (Affymetrix, High Wycombe, United Kingdom). Total RNA of hMSC-old was amplified and labeled according to the Affymetrix GeneChip 3′IVT Express Kit. Following fragmentation, 10 µg of cRNA were hybridized for 16 hr at 45°C on Affymetrix GeneChips Human Genome U133_Plus_2.0. GeneChips were washed and stained in the Affymetrix Fluidics Station 450 using the Affymetrix Hybridization, Wash and Stain Kit. Hybridization signals were detected with Affymetrix Gene Chip Scanner 3000 and global scaling was performed by Affymetrix GeneChipOperatingSoftware 1.4 using the MAS5 algorithm. Microarray data of all 4 hMSC groups have been published in Gene Expression Omnibus (GEO, http://www.ncbi.nlm.nih.gov/geo/) and are accessible through GEO superSeries accession number GSE35959. Gene expression patterns of two groups of hMSC populations were compared with the significance analysis of microarrays (SAM) approach by using the SAM software of Stanford University, Palo Alto, USA (http://www-stat.stanford.edu/~tibs/SAM/) [54]. For data interpretation we only took those gene products into account that provided present hybridization signals in at least 3 of x hMSC populations in at least one of the two groups compared. Furthermore, only gene products (probesets) with fold changes (FC) ≤0.5 or ≥2.0, and a false discovery rate (FDR) <10% were considered as significantly, differentially expressed.

Heat maps were generated by CARMAweb using globally normalized data [55].

Differentially expressed gene products were assigned to protein function by Gene Ontology classification (http://www.geneontology.org/) and NCBI PubMed literature search (http://www.ncbi.nlm.nih.gov/sites/entrez). Genes with at least one differentially expressed probeset were taken into account.

Additionally, SAM data was compared to publically available data from genome-wide association studies, meta-analyses or candidate gene association studies obtained by a NCBI PubMed search for reviews and original publications from 2010 and later with the following search terms: genome-wide association/polymorphism/meta-analysis+osteoporosis or+bone mineral density.

### Quantitative PCR analysis

One microgram of total RNA was reverse-transcribed with Oligo(dT)15 primers (peqlab Biotechnologie GmbH, Erlangen, Germany) and MMLV reverse transcriptase (Promega GmbH, Mannheim, Germany) according to the manufacturer's instructions. Quantitative real-time PCR (qPCR) was performed in triplets in 20 µl with 16 ng cDNA, 5 µl KAPA SYBR FAST Universal 2× qPCR Master Mix (peqlab Biotechnologie GmbH) and 0.25 pmol of sequence specific primers obtained from biomers.net GmbH, Ulm, Germany. The following primer sequences were used (5′-3′ forward and reverse, respectively): RPLP0 (ribosomal protein, large, P0) as housekeeping gene (NM_001002.3) [56], TGCATCAGTACCCCATTCTATCAT and AGGCAGATGGATCAGCCAAGA; SOST (NM_025237.2), CAGGCGTTCAAGAATGATGC and TACTCGGACACGTCTTTGGTC; and MAB21L2 (NM_006439.4), TGGGTGCTACAGTTCG and CAGGCAGGAGATGAGC. QPCR was performed with Opticon DNA Engine (MJ Research, Waltham, USA) and the following conditions: 95°C for 3 min; 40 cycles: 95°C for 15 s; 60°C for 15 s; 72°C for 10 s; followed by melting curve analysis. Results were calculated with the ΔΔC_T_ method.

### Senescence-associated β-galactosidase staining

To confirm replicative senescence in the last, non-confluent passage of hMSC after long time cultivation, senescence-associated β-galactosidase staining was performed as described [57]. After each passage 2×10^5^ cells were seeded on coverslips in 9.6 cm^2^ petri dishes and cultured to 70–90% confluence. After fixation in 2% formaldehyde/0.2% glutaraldehyde for 5 min the coverslips were stored at 4°C. Staining was performed for hMSC in P1and Px simultaneously by incubating the cells for 16 h at 37°C (normal air CO_2_) with 1 ml staining solution (1 mg/ml 5-bromo-4-chloro-3-indolyl β-D-galactosidase (Sigma Aldrich GmbH), 40 mM citric acid/sodium phosphate (pH 6.0), 5 mM potassium ferrocyanide, 5 mM potassium ferricyanide, 150 mM NaCl and 2 mM MgCl_2_). Counterstaining with nuclear fast red was performed after washing twice with ddH_2_O.

## Supporting Information

Table S1 **Gene products with significant expression changes in hMSC-OP compared to hMSC-old.** FC = fold change (at least 2fold); FDR = false discovery rate (<10%).
(DOC)

Table S2 **Gene products differentially expressed in hMSC-OP, hMSC-old and hMSC-senescent when compared to hMSC-C.** arrows pointing downward = significant, reduced expression in comparison to hMSC-C; arrows pointing upward = significant, enhanced expression in comparison to hMSC-C; FC = fold change (at least 2fold); FDR = false discovery rate (<10%); — = no expression in both hMSC groups compared.
(DOC)
