# Supplementary material for: The Transcriptional Profile of Mesenchymal Stem Cell Populations in Primary Osteoporosis Is Distinct and Shows Overexpression of Osteogenic Inhibitors
Source: PLoS One. 2012 Sep 24;7(9):e45142. doi: 10.1371/journal.pone.0045142 (PMC3454401; doi:10.1371/journal.pone.0045142)
Supplement: Table S1 — Gene products with significant expression changes in hMSC-OP compared to hMSC-old. FC = fold change (at least 2fold); FDR = false discovery rate (<10%). (DOC) [file pone.0045142.s001.doc]

**Table S1.** Gene products with significant expression changes in hMSC-OP compared to hMSC-old.

|  | | | **hMSC-OP versus hMSC-old** | |
| --- | --- | --- | --- | --- |
| **Symbol** | **Gene Name** | **Probeset ID** | **FC** | **FDR (%)** |
| REPIN1 | replication initiator 1 | 222501_s_at | 78.47 | 0.00 |
| CYP1B1 | cytochrome P450, family 1, subfamily B, polypeptide 1 | 202434_s_at | 67.17 | 0.00 |
| ZFP36L2 | zinc finger protein 36, C3H type-like 2 | 201367_s_at | 48.34 | 0.00 |
| SMAD3 | SMAD family member 3 | 205397_x_at | 43.27 | 0.00 |
| PCDHGA10 /// PCDHGA11 /// PCDHGA12 /// PCDHGA3 /// PCDHGA5 /// PCDHGA6 | protocadherin gamma subfamily A, 10 /// protocadherin gamma subfamily A, 11 /// protocadherin gamma subfamily A, 12 /// protocadherin gamma subfamily A, 3 /// protocadherin gamma subfamily A, 5 /// protocadherin gamma subfamily A, 6 | 211876_x_at | 33.22 | 0.00 |
| GAS1 | growth arrest-specific 1 | 204456_s_at | 29.04 | 0.00 |
| PLXNA3 | plexin A3 | 1553139_s_at | 26.60 | 0.00 |
| HIPK1 | homeodomain interacting protein kinase 1 | 1552516_a_at | 22.97 | 0.00 |
| AP2A1 | adaptor-related protein complex 2, alpha 1 subunit | 234068_s_at | 22.72 | 0.00 |
| COL1A1 | collagen, type I, alpha 1 | 217430_x_at | 22.19 | 0.00 |
| FKBP8 | FK506 binding protein 8, 38kDa | 40850_at | 20.37 | 0.00 |
| COL6A1 | collagen, type VI, alpha 1 | 212937_s_at | 19.59 | 0.00 |
| IGF2 /// INS-IGF2 | insulin-like growth factor 2 (somatomedin A) /// INS-IGF2 readthrough transcript | 210881_s_at | 18.99 | 0.00 |
| BCL3 | B-cell CLL/lymphoma 3 | 204907_s_at | 18.18 | 0.00 |
| PRR14 | proline rich 14 | 1559397_s_at | 18.16 | 0.00 |
| LOC652346 /// PML | probable transcription factor PML-like /// promyelocytic leukemia | 211588_s_at | 17.95 | 0.00 |
| FGFR1 | fibroblast growth factor receptor 1 | 210973_s_at | 16.59 | 0.00 |
| RARA | retinoic acid receptor, alpha | 203750_s_at | 16.33 | 0.00 |
| ADAMTSL4 | ADAMTS-like 4 | 226071_at | 16.22 | 0.00 |
| FBXO38 | F-box protein 38 | 219608_s_at | 16.20 | 0.00 |
| CDK18 | cyclin-dependent kinase 18 | 214797_s_at | 16.06 | 0.00 |
| PKD1 | polycystic kidney disease 1 (autosomal dominant) | 216949_s_at | 16.04 | 0.00 |
| YWHAE | tyrosine 3-monooxygenase/tryptophan 5-monooxygenase activation protein, epsilon polypeptide | 210317_s_at | 15.94 | 0.00 |
| PLEC | plectin | 216971_s_at | 15.55 | 0.00 |
| ARID1A | AT rich interactive domain 1A (SWI-like) | 210649_s_at | 15.46 | 0.00 |
| LOXL2 | lysyl oxidase-like 2 | 202997_s_at | 15.20 | 0.00 |
| TGFB2 | transforming growth factor, beta 2 | 209908_s_at | 15.16 | 0.00 |
| SETD5 | SET domain containing 5 | 1569106_s_at | 14.79 | 0.00 |
| B4GALT6 | UDP-Gal:betaGlcNAc beta 1,4- galactosyltransferase, polypeptide 6 | 206232_s_at | 14.69 | 0.00 |
| BCL2L1 | BCL2-like 1 | 206665_s_at | 14.20 | 0.00 |
| KDM1B | lysine (K)-specific demethylase 1B | 1553150_at | 14.01 | 0.00 |
| PRKCSH | protein kinase C substrate 80K-H | 200707_at | 13.96 | 0.00 |
| LMAN1 | lectin, mannose-binding, 1 | 203294_s_at | 13.93 | 0.00 |
| MAZ | MYC-associated zinc finger protein (purine-binding transcription factor) | 207824_s_at | 13.50 | 0.00 |
| DHX9 | DEAH (Asp-Glu-Ala-His) box polypeptide 9 | 212105_s_at | 13.49 | 0.00 |
| IGF2 /// INS-IGF2 | insulin-like growth factor 2 (somatomedin A) /// INS-IGF2 readthrough transcript | 202410_x_at | 13.07 | 0.19 |
| AP2A1 | adaptor-related protein complex 2, alpha 1 subunit | 223237_x_at | 12.73 | 0.00 |
| NUCB1 | nucleobindin 1 | 200646_s_at | 12.73 | 0.00 |
| NAV3 | neuron navigator 3 | 1562234_a_at | 12.69 | 0.00 |
| C1QTNF1 | C1q and tumor necrosis factor related protein 1 | 224197_s_at | 12.66 | 0.00 |
| DTX3 | deltex homolog 3 (Drosophila) | 49049_at | 12.46 | 0.00 |
| USP9X | ubiquitin specific peptidase 9, X-linked | 230543_at | 12.26 | 0.00 |
| WNT5B | wingless-type MMTV integration site family, member 5B | 223537_s_at | 12.24 | 0.00 |
| BGN | biglycan | 201262_s_at | 12.20 | 0.00 |
| JUND | jun D proto-oncogene | 203751_x_at | 12.16 | 0.00 |
| CREB3L1 | cAMP responsive element binding protein 3-like 1 | 213498_at | 12.10 | 0.00 |
| RUNX2 | runt-related transcription factor 2 | 216994_s_at | 11.86 | 0.00 |
| GTPBP2 | GTP binding protein 2 | 223789_s_at | 11.86 | 0.08 |
| PCDHGA3 | protocadherin gamma subfamily A, 3 | 216352_x_at | 11.60 | 0.00 |
| SBNO2 | strawberry notch homolog 2 (Drosophila) | 215760_s_at | 11.59 | 0.00 |
| CSGALNACT2 | chondroitin sulfate N-acetylgalactosaminyltransferase 2 | 239077_at | 11.48 | 0.00 |
| COL6A1 | collagen, type VI, alpha 1 | 212091_s_at | 11.44 | 0.00 |
| MTA1 | metastasis associated 1 | 202247_s_at | 11.28 | 0.00 |
| ITGA11 | integrin, alpha 11 | 1554819_a_at | 11.24 | 0.00 |
| CSF1 | colony stimulating factor 1 (macrophage) | 211839_s_at | 11.20 | 0.00 |
| SORBS3 | sorbin and SH3 domain containing 3 | 207788_s_at | 11.16 | 0.00 |
| FSCN1 | fascin homolog 1, actin-bundling protein (Strongylocentrotus purpuratus) | 210933_s_at | 11.15 | 0.00 |
| VAMP2 | vesicle-associated membrane protein 2 (synaptobrevin 2) | 201556_s_at | 11.13 | 0.00 |
| COL6A1 | collagen, type VI, alpha 1 | 212938_at | 11.02 | 0.00 |
| RHOB | ras homolog gene family, member B | 1553962_s_at | 10.99 | 0.00 |
| NUMA1 | nuclear mitotic apparatus protein 1 | 214251_s_at | 10.91 | 0.00 |
| SCIN | scinderin | 1552367_a_at | 10.84 | 0.00 |
| HSF1 | heat shock transcription factor 1 | 213756_s_at | 10.77 | 0.00 |
| RIN3 | Ras and Rab interactor 3 | 219456_s_at | 10.72 | 0.00 |
| ZFP36L1 | zinc finger protein 36, C3H type-like 1 | 211965_at | 10.65 | 0.00 |
| BTBD2 | BTB (POZ) domain containing 2 | 207722_s_at | 10.59 | 0.00 |
| PHLDA1 | pleckstrin homology-like domain, family A, member 1 | 218000_s_at | 10.58 | 0.00 |
| ZYX | zyxin | 215706_x_at | 10.44 | 0.00 |
| SLC4A4 | solute carrier family 4, sodium bicarbonate cotransporter, member 4 | 210738_s_at | 10.40 | 0.00 |
| SMAD3 | SMAD family member 3 | 205396_at | 10.34 | 0.00 |
| TRAPPC1 | trafficking protein particle complex 1 | 225294_s_at | 10.30 | 0.00 |
| ATN1 | atrophin 1 | 208871_at | 10.25 | 0.00 |
| CD74 | CD74 molecule, major histocompatibility complex, class II invariant chain | 209619_at | 10.00 | 0.00 |
| HSPB6 | heat shock protein, alpha-crystallin-related, B6 | 214767_s_at | 9.94 | 0.00 |
| PKM2 | pyruvate kinase, muscle | 201251_at | 9.92 | 0.00 |
| EPN1 | epsin 1 | 221141_x_at | 9.69 | 0.00 |
| ATN1 | atrophin 1 | 40489_at | 9.69 | 0.00 |
| SPRED2 | sprouty-related, EVH1 domain containing 2 | 212466_at | 9.49 | 0.00 |
| TRPV2 | transient receptor potential cation channel, subfamily V, member 2 | 222855_s_at | 9.45 | 0.00 |
| IBSP | integrin-binding sialoprotein | 207370_at | 9.41 | 0.00 |
| PARVB | parvin, beta | 216253_s_at | 9.40 | 0.00 |
| BHLHE40 | basic helix-loop-helix family, member e40 | 201169_s_at | 9.39 | 0.00 |
| MAST2 | microtubule associated serine/threonine kinase 2 | 215660_s_at | 9.33 | 0.00 |
| DYNC2H1 | dynein, cytoplasmic 2, heavy chain 1 | 1561939_at | 9.28 | 0.08 |
| PDLIM7 | PDZ and LIM domain 7 (enigma) | 203370_s_at | 9.20 | 0.00 |
| PHF12 | PHD finger protein 12 | 234939_s_at | 9.17 | 0.00 |
| RXRB | retinoid X receptor, beta | 215099_s_at | 9.08 | 0.00 |
| ASXL2 | additional sex combs like 2 (Drosophila) | 1555266_a_at | 9.07 | 0.00 |
| CYB5R3 | cytochrome b5 reductase 3 | 1554574_a_at | 9.07 | 0.00 |
| ELN | elastin | 212670_at | 9.06 | 0.00 |
| TRIB1 | tribbles homolog 1 (Drosophila) | 239818_x_at | 9.04 | 0.00 |
| TEAD2 | TEA domain family member 2 | 238323_at | 9.03 | 0.00 |
| SHC1 | SHC (Src homology 2 domain containing) transforming protein 1 | 201469_s_at | 9.00 | 0.00 |
| HSPB6 | heat shock protein, alpha-crystallin-related, B6 | 226304_at | 8.96 | 0.00 |
| PVRL2 | poliovirus receptor-related 2 (herpesvirus entry mediator B) | 232078_at | 8.95 | 0.00 |
| TFE3 | transcription factor binding to IGHM enhancer 3 | 1565347_s_at | 8.94 | 0.00 |
| MAPKAPK2 | mitogen-activated protein kinase-activated protein kinase 2 | 201461_s_at | 8.91 | 0.00 |
| NEK9 | NIMA (never in mitosis gene a)- related kinase 9 | 214738_s_at | 8.90 | 0.00 |
| RAC2 | ras-related C3 botulinum toxin substrate 2 (rho family, small GTP binding protein Rac2) | 207419_s_at | 8.89 | 0.00 |
| KIAA1826 | KIAA1826 | 223799_at | 8.83 | 0.00 |
| ELN | elastin | 216269_s_at | 8.81 | 0.00 |
| STX16 | syntaxin 16 | 221638_s_at | 8.78 | 0.00 |
| DCAF15 | DDB1 and CUL4 associated factor 15 | 221851_at | 8.73 | 0.00 |
| LRSAM1 | leucine rich repeat and sterile alpha motif containing 1 | 235449_at | 8.69 | 0.00 |
| SORT1 | sortilin 1 | 212797_at | 8.68 | 0.00 |
| TGFB1 | transforming growth factor, beta 1 | 203085_s_at | 8.64 | 0.00 |
| SYDE1 | synapse defective 1, Rho GTPase, homolog 1 (C. elegans) | 212962_at | 8.62 | 0.00 |
| PML | promyelocytic leukemia | 210362_x_at | 8.60 | 0.00 |
| EPN1 | epsin 1 | 226667_x_at | 8.60 | 0.00 |
| ZER1 | zer-1 homolog (C. elegans) | 202448_s_at | 8.55 | 0.00 |
| CANT1 | calcium activated nucleotidase 1 | 1554327_a_at | 8.50 | 0.00 |
| TRAM2 | translocation associated membrane protein 2 | 1554383_a_at | 8.49 | 0.08 |
| SPTAN1 | spectrin, alpha, non-erythrocytic 1 (alpha-fodrin) | 208611_s_at | 8.44 | 0.00 |
| EPOR | erythropoietin receptor | 209962_at | 8.41 | 0.00 |
| AKIRIN2 | akirin 2 | 223143_s_at | 8.40 | 0.00 |
| TPP1 | tripeptidyl peptidase I | 214196_s_at | 8.40 | 0.00 |
| THBS1 | thrombospondin 1 | 201107_s_at | 8.27 | 0.00 |
| MAPRE3 | microtubule-associated protein, RP/EB family, member 3 | 203841_x_at | 8.27 | 0.00 |
| ZYX | zyxin | 200808_s_at | 8.23 | 0.00 |
| VAMP2 | vesicle-associated membrane protein 2 (synaptobrevin 2) | 201557_at | 8.23 | 0.00 |
| PTPN12 | protein tyrosine phosphatase, non-receptor type 12 | 216915_s_at | 8.21 | 0.00 |
| SAMD4B | Sterile alpha motif domain containing 4B | 227511_at | 8.20 | 0.00 |
| PREX1 | phosphatidylinositol-3,4,5-trisphosphate-dependent Rac exchange factor 1 | 224909_s_at | 8.18 | 0.00 |
| EPHX1 | epoxide hydrolase 1, microsomal (xenobiotic) | 202017_at | 8.15 | 0.00 |
| SLC6A6 | solute carrier family 6 (neurotransmitter transporter, taurine), member 6 | 205920_at | 8.14 | 0.00 |
| SSFA2 | sperm specific antigen 2 | 236207_at | 8.14 | 0.00 |
| MBNL2 | muscleblind-like 2 (Drosophila) | 205018_s_at | 8.13 | 0.08 |
| LMAN1 | lectin, mannose-binding, 1 | 203293_s_at | 8.00 | 0.00 |
| CD74 | CD74 molecule, major histocompatibility complex, class II invariant chain | 1567628_at | 8.00 | 0.00 |
| APBB1IP | amyloid beta (A4) precursor protein-binding, family B, member 1 interacting protein | 219994_at | 7.95 | 0.00 |
| RCAN1 | regulator of calcineurin 1 | 215253_s_at | 7.93 | 0.00 |
| TRIM8 | tripartite motif-containing 8 | 223131_s_at | 7.92 | 0.00 |
| WIZ | widely interspaced zinc finger motifs | 221785_at | 7.89 | 0.00 |
| HIPK1 | homeodomain interacting protein kinase 1 | 212291_at | 7.83 | 0.00 |
| ASPH | aspartate beta-hydroxylase | 205808_at | 7.80 | 0.00 |
| PDK4 | pyruvate dehydrogenase kinase, isozyme 4 | 205960_at | 7.79 | 0.00 |
| RAB6A | RAB6A, member RAS oncogene family | 201048_x_at | 7.76 | 0.00 |
| LOC100288985 | Hypothetical protein LOC100288985 | 230746_s_at | 7.74 | 0.08 |
| EPAS1 | endothelial PAS domain protein 1 | 200879_s_at | 7.71 | 0.00 |
| NAPA | N-ethylmaleimide-sensitive factor attachment protein, alpha | 206491_s_at | 7.70 | 0.00 |
| PLD3 | phospholipase D family, member 3 | 201050_at | 7.69 | 0.00 |
| MBNL1 | muscleblind-like (Drosophila) | 1555594_a_at | 7.67 | 0.14 |
| CTNND1 | catenin (cadherin-associated protein), delta 1 | 1557944_s_at | 7.66 | 0.00 |
| CDC42EP1 | CDC42 effector protein (Rho GTPase binding) 1 | 204693_at | 7.63 | 0.00 |
| VEGFA | vascular endothelial growth factor A | 211527_x_at | 7.63 | 0.00 |
| HSPG2 | heparan sulfate proteoglycan 2 | 201654_s_at | 7.62 | 0.00 |
| HS3ST3B1 | Heparan sulfate (glucosamine) 3-O-sulfotransferase 3B1 | 1561908_a_at | 7.58 | 0.00 |
| PCGF1 | polycomb group ring finger 1 | 210023_s_at | 7.56 | 0.00 |
| BUB1 | budding uninhibited by benzimidazoles 1 homolog (yeast) | 215509_s_at | 7.56 | 0.33 |
| LY6E | lymphocyte antigen 6 complex, locus E | 202145_at | 7.52 | 0.00 |
| NUCB1 | nucleobindin 1 | 200649_at | 7.48 | 0.00 |
| CLPTM1 | cleft lip and palate associated transmembrane protein 1 | 211136_s_at | 7.45 | 0.00 |
| GTF2I | general transcription factor IIi | 210892_s_at | 7.40 | 0.00 |
| PPP1R9B | protein phosphatase 1, regulatory (inhibitor) subunit 9B | 225124_at | 7.38 | 0.00 |
| MAB21L2 | mab-21-like 2 (C. elegans) | 210303_at | 7.38 | 0.00 |
| LOX | lysyl oxidase | 213640_s_at | 7.36 | 0.00 |
| PLTP | phospholipid transfer protein | 202075_s_at | 7.35 | 0.00 |
| HNRNPUL1 | heterogeneous nuclear ribonucleoprotein U-like 1 | 209675_s_at | 7.31 | 0.00 |
| NAPA | N-ethylmaleimide-sensitive factor attachment protein, alpha | 208751_at | 7.31 | 0.00 |
| CLINT1 | clathrin interactor 1 | 230609_at | 7.31 | 0.00 |
| WDR6 | WD repeat domain 6 | 233573_s_at | 7.25 | 0.00 |
| DIRAS1 | DIRAS family, GTP-binding RAS-like 1 | 226573_at | 7.23 | 0.00 |
| SBNO2 | strawberry notch homolog 2 (Drosophila) | 204166_at | 7.22 | 0.00 |
| ZNF358 | zinc finger protein 358 | 226260_x_at | 7.22 | 0.00 |
| CALR | calreticulin | 200935_at | 7.21 | 0.00 |
| PPDPF | pancreatic progenitor cell differentiation and proliferation factor homolog (zebrafish) | 218010_x_at | 7.15 | 0.00 |
| PTPN11 | protein tyrosine phosphatase, non-receptor type 11 | 205867_at | 7.10 | 0.00 |
| RNF208 | ring finger protein 208 | 221273_s_at | 7.09 | 0.00 |
| MET | met proto-oncogene (hepatocyte growth factor receptor) | 213807_x_at | 7.07 | 0.00 |
| ARHGAP1 | Rho GTPase activating protein 1 | 216689_x_at | 7.06 | 0.00 |
| SPAG9 | sperm associated antigen 9 | 206748_s_at | 7.06 | 0.00 |
| ITGB2 | integrin, beta 2 (complement component 3 receptor 3 and 4 subunit) | 1555349_a_at | 7.05 | 0.00 |
| LRRC15 | leucine rich repeat containing 15 | 1552960_at | 7.01 | 0.08 |
| DCAF15 | DDB1 and CUL4 associated factor 15 | 91952_at | 7.00 | 0.00 |
| ISYNA1 | inositol-3-phosphate synthase 1 | 222240_s_at | 6.99 | 0.00 |
| C16orf58 | chromosome 16 open reading frame 58 | 217891_at | 6.99 | 0.00 |
| EGFR | epidermal growth factor receptor | 211607_x_at | 6.96 | 0.08 |
| CBX4 | chromobox homolog 4 | 206724_at | 6.94 | 0.00 |
| SCYL2 | SCY1-like 2 (S. cerevisiae) | 221220_s_at | 6.93 | 1.00 |
| VEGFB | vascular endothelial growth factor B | 203683_s_at | 6.92 | 0.00 |
| CDV3 | CDV3 homolog (mouse) | 213548_s_at | 6.89 | 0.00 |
| SBF2 | SET binding factor 2 | 242935_at | 6.85 | 0.00 |
| RBBP9 | retinoblastoma binding protein 9 | 232751_at | 6.85 | 0.00 |
| B4GALT1 | UDP-Gal:betaGlcNAc beta 1,4- galactosyltransferase, polypeptide 1 | 228498_at | 6.81 | 0.00 |
| BCL2L13 | BCL2-like 13 (apoptosis facilitator) | 224035_s_at | 6.80 | 0.00 |
| PNPLA2 | patatin-like phospholipase domain containing 2 | 212705_x_at | 6.73 | 0.00 |
| TMEM206 | transmembrane protein 206 | 218814_s_at | 6.72 | 0.23 |
| SPTBN1 | spectrin, beta, non-erythrocytic 1 | 200671_s_at | 6.72 | 0.00 |
| OGDH | oxoglutarate (alpha-ketoglutarate) dehydrogenase (lipoamide) | 1554152_a_at | 6.70 | 0.08 |
| LARP1 | La ribonucleoprotein domain family, member 1 | 212193_s_at | 6.68 | 0.00 |
| TLN1 | talin 1 | 203254_s_at | 6.68 | 0.00 |
| KIAA1199 | KIAA1199 | 1554685_a_at | 6.65 | 0.19 |
| RAB11B | RAB11B, member RAS oncogene family | 34478_at | 6.65 | 0.00 |
| SFRS11 | Splicing factor, arginine/serine-rich 11 | 237746_at | 6.64 | 0.00 |
| DCAF15 | DDB1 and CUL4 associated factor 15 | 221849_s_at | 6.63 | 0.00 |
| ERAP2 | endoplasmic reticulum aminopeptidase 2 | 1554273_a_at | 6.63 | 0.08 |
| HMGA1 | high mobility group AT-hook 1 | 210457_x_at | 6.61 | 0.00 |
| DCHS1 | dachsous 1 (Drosophila) | 218892_at | 6.61 | 0.00 |
| TP53 | tumor protein p53 | 211300_s_at | 6.61 | 0.00 |
| MAPRE3 | microtubule-associated protein, RP/EB family, member 3 | 214270_s_at | 6.60 | 0.00 |
| RNF6 | ring finger protein (C3H2C3 type) 6 | 210932_s_at | 6.60 | 0.00 |
| SLC1A1 | solute carrier family 1 (neuronal/epithelial high affinity glutamate transporter, system Xag), member 1 | 206396_at | 6.59 | 0.00 |
| CYGB | cytoglobin | 1553572_a_at | 6.57 | 0.00 |
| SEC61A1 | Sec61 alpha 1 subunit (S. cerevisiae) | 222385_x_at | 6.56 | 0.00 |
| CIZ1 | CDKN1A interacting zinc finger protein 1 | 213977_s_at | 6.56 | 0.00 |
| FBXO28 | F-box protein 28 | 1555972_s_at | 6.56 | 0.33 |
| CDCP1 | CUB domain containing protein 1 | 234932_s_at | 6.55 | 0.00 |
| VAMP2 | vesicle-associated membrane protein 2 (synaptobrevin 2) | 214792_x_at | 6.53 | 0.00 |
| PARVB | parvin, beta | 37965_at | 6.53 | 0.00 |
| PVR | poliovirus receptor | 214444_s_at | 6.52 | 0.08 |
| SLC12A4 | solute carrier family 12 (potassium/chloride transporters), member 4 | 209401_s_at | 6.50 | 0.00 |
| GM2A | GM2 ganglioside activator | 215891_s_at | 6.50 | 0.00 |
| JOSD2 | Josephin domain containing 2 | 227096_at | 6.50 | 0.00 |
| MBD1 | methyl-CpG binding domain protein 1 | 1555611_s_at | 6.49 | 0.00 |
| SEC16B | SEC16 homolog B (S. cerevisiae) | 1564423_a_at | 6.47 | 0.23 |
| DDX42 | DEAD (Asp-Glu-Ala-Asp) box polypeptide 42 | 1559954_s_at | 6.45 | 0.00 |
| PEA15 | phosphoprotein enriched in astrocytes 15 | 200787_s_at | 6.45 | 0.00 |
| GLYR1 | glyoxylate reductase 1 homolog (Arabidopsis) | 221628_s_at | 6.45 | 0.08 |
| JAM3 | junctional adhesion molecule 3 | 231721_at | 6.44 | 0.00 |
| LARP4B | La ribonucleoprotein domain family, member 4B | 214216_s_at | 6.44 | 0.00 |
| UBE3B | ubiquitin protein ligase E3B | 212404_s_at | 6.44 | 0.00 |
| MAZ | MYC-associated zinc finger protein (purine-binding transcription factor) | 212064_x_at | 6.44 | 0.00 |
| EDIL3 | EGF-like repeats and discoidin I-like domains 3 | 207379_at | 6.44 | 0.00 |
| GSN | gelsolin | 214040_s_at | 6.40 | 0.00 |
| CNOT3 | CCR4-NOT transcription complex, subunit 3 | 203239_s_at | 6.39 | 0.00 |
| ITGA7 | integrin, alpha 7 | 209663_s_at | 6.36 | 0.00 |
| SH3GLB2 | SH3-domain GRB2-like endophilin B2 | 218813_s_at | 6.36 | 0.00 |
| C19orf6 | chromosome 19 open reading frame 6 | 212574_x_at | 6.34 | 0.00 |
| FLOT2 | flotillin 2 | 211299_s_at | 6.34 | 0.00 |
| TPM4 | tropomyosin 4 | 209344_at | 6.31 | 0.00 |
| C19orf6 | chromosome 19 open reading frame 6 | 213986_s_at | 6.31 | 0.00 |
| TNFRSF10B | tumor necrosis factor receptor superfamily, member 10b | 209294_x_at | 6.30 | 0.00 |
| FOXC2 | forkhead box C2 (MFH-1, mesenchyme forkhead 1) | 214520_at | 6.24 | 0.00 |
| ANGPTL2 | angiopoietin-like 2 | 219514_at | 6.18 | 0.00 |
| USP25 | ubiquitin specific peptidase 25 | 1555559_s_at | 6.16 | 0.14 |
| GNA13 | guanine nucleotide binding protein (G protein), alpha 13 | 206917_at | 6.15 | 0.00 |
| SCARF2 | scavenger receptor class F, member 2 | 239454_at | 6.15 | 0.00 |
| SLC35A2 | solute carrier family 35 (UDP-galactose transporter), member A2 | 207439_s_at | 6.15 | 0.00 |
| TNFRSF19 | tumor necrosis factor receptor superfamily, member 19 | 223827_at | 6.12 | 0.00 |
| GRINA | glutamate receptor, ionotropic, N-methyl D-aspartate-associated protein 1 (glutamate binding) | 212090_at | 6.11 | 0.00 |
| SNTB1 | syntrophin, beta 1 (dystrophin-associated protein A1, 59kDa, basic component 1) | 208608_s_at | 6.09 | 0.08 |
| TGFBR2 | transforming growth factor, beta receptor II (70/80kDa) | 207334_s_at | 6.07 | 0.00 |
| LOC100506266 | hypothetical LOC100506266 | 243641_at | 6.06 | 0.08 |
| TP53I11 | tumor protein p53 inducible protein 11 | 214667_s_at | 6.01 | 0.00 |
| TWF1 | twinfilin, actin-binding protein, homolog 1 (Drosophila) | 214007_s_at | 6.01 | 0.00 |
| TGM2 | transglutaminase 2 (C polypeptide, protein-glutamine-gamma-glutamyltransferase) | 211003_x_at | 6.01 | 0.14 |
| KHSRP | KH-type splicing regulatory protein | 212303_x_at | 5.97 | 0.00 |
| PCDHGA1 /// PCDHGA10 /// PCDHGA11 /// PCDHGA12 /// PCDHGA2 /// PCDHGA3 /// PCDHGA4 /// PCDHGA5 /// PCDHGA6 /// PCDHGA7 /// PCDHGA8 /// PCDHGA9 /// PCDHGB1 /// PCDHGB2 /// PCDHGB3 /// PCDHGB4 /// PCDHGB5 /// PCDHGB6 /// PCDHGB7 /// PCDHGC3 /// PCDHGC4 /// PCDHGC5 | protocadherin gamma subfamily A, 1 /// protocadherin gamma subfamily A, 10 /// protocadherin gamma subfamily A, 11 /// protocadherin gamma subfamily A, 12 /// protocadherin gamma subfamily A, 2 /// protocadherin gamma subfamily A, 3 /// protocadherin gamma subfamily A, 4 /// protocadherin gamma subfamily A, 5 /// protocadherin gamma subfamily A, 6 /// protocadherin gamma subfamily A, 7 /// protocadherin gamma subfamily A, 8 /// protocadherin gamma subfamily A, 9 /// protocadherin gamma subfamily B, 1 /// protocadherin gamma subfamily B, 2 /// protocadherin gamma subfamily B, 3 /// protocadherin gamma subfamily B, 4 /// protocadherin gamma subfamily B, 5 /// protocadherin gamma subfamily B, 6 /// protocadherin gamma subfamily B, 7 /// protocadherin gamma subfamily C, 3 /// protocadherin gamma subfamily C, 4 /// protocadherin gamma subfamily C, 5 | 205717_x_at | 5.97 | 0.00 |
| VASH1 | Vasohibin 1 | 1556423_at | 5.96 | 0.08 |
| PPP1R15A | protein phosphatase 1, regulatory (inhibitor) subunit 15A | 202014_at | 5.96 | 0.00 |
| DTX3 | deltex homolog 3 (Drosophila) | 49051_g_at | 5.95 | 0.00 |
| FBXW11 | F-box and WD repeat domain containing 11 | 209456_s_at | 5.95 | 0.00 |
| ATXN2L | ataxin 2-like | 201806_s_at | 5.92 | 0.00 |
| SCIN | scinderin | 1552365_at | 5.91 | 1.25 |
| PEG10 | paternally expressed 10 | 212092_at | 5.90 | 0.00 |
| SLC39A14 | solute carrier family 39 (zinc transporter), member 14 | 1555434_a_at | 5.90 | 0.00 |
| XYLT2 | Xylosyltransferase II | 231550_at | 5.89 | 0.00 |
| DSEL | dermatan sulfate epimerase-like | 244852_at | 5.88 | 0.33 |
| C9orf95 | chromosome 9 open reading frame 95 | 1562761_at | 5.88 | 0.08 |
| SMTN | smoothelin | 209427_at | 5.87 | 0.00 |
| ARHGDIA | Rho GDP dissociation inhibitor (GDI) alpha | 213606_s_at | 5.85 | 0.00 |
| INPP5A | inositol polyphosphate-5-phosphatase, 40kDa | 1554757_a_at | 5.84 | 0.00 |
| KDM6B | lysine (K)-specific demethylase 6B | 213146_at | 5.84 | 0.00 |
| TRAF7 | TNF receptor-associated factor 7 | 223029_s_at | 5.81 | 0.00 |
| NPRL3 | nitrogen permease regulator-like 3 (S. cerevisiae) | 203289_s_at | 5.80 | 0.00 |
| PCDHGA1 /// PCDHGA10 /// PCDHGA11 /// PCDHGA12 /// PCDHGA2 /// PCDHGA3 /// PCDHGA4 /// PCDHGA5 /// PCDHGA6 /// PCDHGA7 /// PCDHGA8 /// PCDHGA9 /// PCDHGB1 /// PCDHGB2 /// PCDHGB3 /// PCDHGB4 /// PCDHGB5 /// PCDHGB6 /// PCDHGB7 /// PCDHGC3 /// PCDHGC4 /// PCDHGC5 | protocadherin gamma subfamily A, 1 /// protocadherin gamma subfamily A, 10 /// protocadherin gamma subfamily A, 11 /// protocadherin gamma subfamily A, 12 /// protocadherin gamma subfamily A, 2 /// protocadherin gamma subfamily A, 3 /// protocadherin gamma subfamily A, 4 /// protocadherin gamma subfamily A, 5 /// protocadherin gamma subfamily A, 6 /// protocadherin gamma subfamily A, 7 /// protocadherin gamma subfamily A, 8 /// protocadherin gamma subfamily A, 9 /// protocadherin gamma subfamily B, 1 /// protocadherin gamma subfamily B, 2 /// protocadherin gamma subfamily B, 3 /// protocadherin gamma subfamily B, 4 /// protocadherin gamma subfamily B, 5 /// protocadherin gamma subfamily B, 6 /// protocadherin gamma subfamily B, 7 /// protocadherin gamma subfamily C, 3 /// protocadherin gamma subfamily C, 4 /// protocadherin gamma subfamily C, 5 | 215836_s_at | 5.80 | 0.00 |
| EWSR1 /// FLI1 | Ewing sarcoma breakpoint region 1 /// Friend leukemia virus integration 1 | 211825_s_at | 5.80 | 0.00 |
| H2AFX | H2A histone family, member X | 212525_s_at | 5.79 | 0.00 |
| ARID1A | AT rich interactive domain 1A (SWI-like) | 212152_x_at | 5.77 | 0.00 |
| GNS | glucosamine (N-acetyl)-6-sulfatase | 203676_at | 5.77 | 0.00 |
| SLC29A1 | solute carrier family 29 (nucleoside transporters), member 1 | 201801_s_at | 5.77 | 0.00 |
| KRTAP8-1 | keratin associated protein 8-1 | 1564974_at | 5.76 | 0.08 |
| TNFRSF10B | tumor necrosis factor receptor superfamily, member 10b | 210405_x_at | 5.76 | 0.00 |
| PHTF2 | putative homeodomain transcription factor 2 | 217097_s_at | 5.76 | 0.00 |
| PVRL2 | poliovirus receptor-related 2 (herpesvirus entry mediator B) | 232079_s_at | 5.71 | 0.00 |
| DGKA | diacylglycerol kinase, alpha 80kDa | 211272_s_at | 5.71 | 0.00 |
| PLAU | plasminogen activator, urokinase | 211668_s_at | 5.70 | 0.08 |
| BCL2L1 | BCL2-like 1 | 215037_s_at | 5.67 | 0.00 |
| CTXN1 | cortexin 1 | 228126_x_at | 5.65 | 0.08 |
| ATP2B4 | ATPase, Ca++ transporting, plasma membrane 4 | 205410_s_at | 5.65 | 0.08 |
| PIP5K1A | phosphatidylinositol-4-phosphate 5-kinase, type I, alpha | 211205_x_at | 5.64 | 0.08 |
| DCTN1 | dynactin 1 | 201082_s_at | 5.63 | 0.00 |
| FZR1 | fizzy/cell division cycle 20 related 1 (Drosophila) | 209416_s_at | 5.60 | 0.00 |
| CDK16 | cyclin-dependent kinase 16 | 207239_s_at | 5.59 | 0.00 |
| GRPEL2 | GrpE-like 2, mitochondrial (E. coli) | 238427_at | 5.58 | 0.00 |
| WTAP | Wilms tumor 1 associated protein | 227621_at | 5.58 | 0.00 |
| EHD2 | EH-domain containing 2 | 205341_at | 5.57 | 0.00 |
| KHSRP | KH-type splicing regulatory protein | 204371_s_at | 5.57 | 0.00 |
| RC3H2 | ring finger and CCCH-type domains 2 | 230134_s_at | 5.55 | 0.00 |
| SLC39A6 | solute carrier family 39 (zinc transporter), member 6 | 1556551_s_at | 5.53 | 0.00 |
| SRPR | signal recognition particle receptor (docking protein) | 200917_s_at | 5.53 | 0.00 |
| SIRPA | signal-regulatory protein alpha | 202895_s_at | 5.52 | 0.00 |
| MCL1 | myeloid cell leukemia sequence 1 (BCL2-related) | 200796_s_at | 5.52 | 0.63 |
| RAB11B | RAB11B, member RAS oncogene family | 217793_at | 5.51 | 0.08 |
| DHX35 | DEAH (Asp-Glu-Ala-His) box polypeptide 35 | 234728_s_at | 5.50 | 0.00 |
| RASSF8 | Ras association (RalGDS/AF-6) domain family (N-terminal) member 8 | 235996_at | 5.50 | 0.19 |
| PTPN14 | protein tyrosine phosphatase, non-receptor type 14 | 205503_at | 5.49 | 0.00 |
| SOLH | small optic lobes homolog (Drosophila) | 204275_at | 5.49 | 0.00 |
| FAM198B | family with sequence similarity 198, member B | 219872_at | 5.48 | 1.00 |
| PTBP1 | polypyrimidine tract binding protein 1 | 212016_s_at | 5.48 | 0.00 |
| RNF26 | ring finger protein 26 | 224338_s_at | 5.46 | 0.00 |
| SHB | Src homology 2 domain containing adaptor protein B | 204657_s_at | 5.45 | 0.00 |
| TRIM28 | tripartite motif-containing 28 | 200990_at | 5.44 | 0.00 |
| ARHGDIA | Rho GDP dissociation inhibitor (GDI) alpha | 201167_x_at | 5.44 | 0.00 |
| MICAL2 | microtubule associated monoxygenase, calponin and LIM domain containing 2 | 206275_s_at | 5.44 | 0.08 |
| VEGFA | vascular endothelial growth factor A | 210513_s_at | 5.43 | 0.00 |
| RBM6 | RNA binding motif protein 6 | 228030_at | 5.43 | 0.00 |
| NFIB | Nuclear factor I/B | 230291_s_at | 5.41 | 0.23 |
| CLDN18 | claudin 18 | 214135_at | 5.41 | 0.00 |
| FAM129B | family with sequence similarity 129, member B | 233974_s_at | 5.41 | 0.00 |
| ADAT3 | adenosine deaminase, tRNA-specific 3, TAD3 homolog (S. cerevisiae) | 1553967_at | 5.40 | 1.25 |
| MDC1 | mediator of DNA-damage checkpoint 1 | 203061_s_at | 5.40 | 0.08 |
| EPHB3 | EPH receptor B3 | 1438_at | 5.40 | 0.00 |
| MARCKS | myristoylated alanine-rich protein kinase C substrate | 201668_x_at | 5.39 | 0.00 |
| MYO9B | myosin IXB | 208452_x_at | 5.39 | 0.00 |
| ZNF672 | zinc finger protein 672 | 222509_s_at | 5.39 | 0.33 |
| SSBP3 | single stranded DNA binding protein 3 | 223635_s_at | 5.37 | 0.00 |
| FOSL2 | FOS-like antigen 2 | 205409_at | 5.36 | 0.00 |
| TGFA | transforming growth factor, alpha | 205016_at | 5.36 | 0.26 |
| RBM14 | RNA binding motif protein 14 | 1555639_a_at | 5.36 | 0.00 |
| EXOC4 | exocyst complex component 4 | 240528_s_at | 5.35 | 0.14 |
| GM2A | GM2 ganglioside activator | 209727_at | 5.33 | 0.26 |
| SUPT16H | suppressor of Ty 16 homolog (S. cerevisiae) | 233827_s_at | 5.32 | 0.55 |
| HPCAL1 | hippocalcin-like 1 | 205462_s_at | 5.32 | 0.00 |
| CALCOCO1 | calcium binding and coiled-coil domain 1 | 209002_s_at | 5.32 | 0.00 |
| CFL1 | cofilin 1 (non-muscle) | 1555730_a_at | 5.31 | 0.00 |
| FOXK2 | forkhead box K2 | 242938_s_at | 5.31 | 0.00 |
| COL6A2 | collagen, type VI, alpha 2 | 209156_s_at | 5.31 | 0.00 |
| DYRK1B | dual-specificity tyrosine-(Y)-phosphorylation regulated kinase 1B | 217270_s_at | 5.30 | 0.08 |
| ARHGEF2 | Rho/Rac guanine nucleotide exchange factor (GEF) 2 | 1554783_s_at | 5.30 | 0.19 |
| UBA1 | ubiquitin-like modifier activating enzyme 1 | 200964_at | 5.30 | 0.00 |
| MBNL2 | muscleblind-like 2 (Drosophila) | 205017_s_at | 5.30 | 0.63 |
| ZC3H12A | zinc finger CCCH-type containing 12A | 218810_at | 5.29 | 0.08 |
| RALGDS | ral guanine nucleotide dissociation stimulator | 209051_s_at | 5.28 | 0.00 |
| RERE | arginine-glutamic acid dipeptide (RE) repeats | 221643_s_at | 5.26 | 0.00 |
| TCF3 | transcription factor 3 (E2A immunoglobulin enhancer binding factors E12/E47) | 215260_s_at | 5.23 | 0.00 |
| PCDH7 | protocadherin 7 | 210273_at | 5.23 | 0.19 |
| MINK1 | misshapen-like kinase 1 | 215909_x_at | 5.23 | 0.00 |
| PDLIM7 | PDZ and LIM domain 7 (enigma) | 214266_s_at | 5.21 | 0.08 |
| GPR176 | G protein-coupled receptor 176 | 206673_at | 5.21 | 0.00 |
| TAB3 | TGF-beta activated kinase 1/MAP3K7 binding protein 3 | 1552928_s_at | 5.20 | 0.44 |
| GNAQ | guanine nucleotide binding protein (G protein), q polypeptide | 211426_x_at | 5.18 | 0.19 |
| ARHGDIA | Rho GDP dissociation inhibitor (GDI) alpha | 201168_x_at | 5.16 | 0.00 |
| MAP3K2 | mitogen-activated protein kinase kinase kinase 2 | 221695_s_at | 5.16 | 0.00 |
| RANGAP1 | Ran GTPase activating protein 1 | 212125_at | 5.15 | 0.00 |
| ESYT2 | extended synaptotagmin-like protein 2 | 1555830_s_at | 5.14 | 0.00 |
| MLF2 | myeloid leukemia factor 2 | 200948_at | 5.12 | 0.00 |
| KRT33A | keratin 33A | 208483_x_at | 5.12 | 1.00 |
| COG3 | component of oligomeric golgi complex 3 | 1554339_a_at | 5.11 | 0.08 |
| NRP2 | neuropilin 2 | 210841_s_at | 5.11 | 0.00 |
| GSTM4 | glutathione S-transferase mu 4 | 204149_s_at | 5.10 | 0.00 |
| ROCK2 | Rho-associated, coiled-coil containing protein kinase 2 | 211504_x_at | 5.09 | 0.00 |
| ABHD2 | abhydrolase domain containing 2 | 87100_at | 5.08 | 1.25 |
| SCAMP4 | secretory carrier membrane protein 4 | 235073_at | 5.08 | 0.08 |
| MIAT | myocardial infarction associated transcript (non-protein coding) | 237322_at | 5.07 | 0.35 |
| PCGF2 | polycomb group ring finger 2 | 203792_x_at | 5.06 | 0.00 |
| PPP1R3B | protein phosphatase 1, regulatory (inhibitor) subunit 3B | 1552670_a_at | 5.06 | 0.00 |
| GHDC | GH3 domain containing | 227159_at | 5.06 | 0.00 |
| CECR2 | cat eye syndrome chromosome region, candidate 2 | 223729_at | 5.06 | 0.26 |
| SMARCD1 | SWI/SNF related, matrix associated, actin dependent regulator of chromatin, subfamily d, member 1 | 203183_s_at | 5.06 | 0.00 |
| AKT2 | v-akt murine thymoma viral oncogene homolog 2 | 203809_s_at | 5.05 | 0.33 |
| VAC14 | Vac14 homolog (S. cerevisiae) | 218169_at | 5.04 | 0.00 |
| YIF1B | Yip1 interacting factor homolog B (S. cerevisiae) | 1554553_s_at | 5.03 | 0.00 |
| UBE4B | ubiquitination factor E4B (UFD2 homolog, yeast) | 202316_x_at | 5.03 | 0.14 |
| GART | phosphoribosylglycinamide formyltransferase, phosphoribosylglycinamide synthetase, phosphoribosylaminoimidazole synthetase | 217445_s_at | 5.03 | 0.00 |
| CDK16 | cyclin-dependent kinase 16 | 208824_x_at | 5.02 | 0.00 |
| SSH1 | slingshot homolog 1 (Drosophila) | 1555624_a_at | 5.02 | 0.08 |
| GNB4 | guanine nucleotide binding protein (G protein), beta polypeptide 4 | 223488_s_at | 5.01 | 0.00 |
| NME7 | non-metastatic cells 7, protein expressed in (nucleoside-diphosphate kinase) | 227556_at | 5.01 | 0.00 |
| BANF1 | barrier to autointegration factor 1 | 210125_s_at | 5.01 | 0.00 |
| SMTN | smoothelin | 207390_s_at | 5.00 | 0.00 |
| HIP1 | huntingtin interacting protein 1 | 205426_s_at | 5.00 | 0.33 |
| PARVB | parvin, beta | 204629_at | 5.00 | 0.00 |
| SLC6A8 | solute carrier family 6 (neurotransmitter transporter, creatine), member 8 | 210854_x_at | 5.00 | 0.00 |
| LSS | lanosterol synthase (2,3-oxidosqualene-lanosterol cyclase) | 211019_s_at | 4.99 | 0.00 |
| ATP6V1A | ATPase, H+ transporting, lysosomal 70kDa, V1 subunit A | 201971_s_at | 4.99 | 0.14 |
| ADAMTS2 | ADAM metallopeptidase with thrombospondin type 1 motif, 2 | 214535_s_at | 4.96 | 0.00 |
| ST8SIA1 | ST8 alpha-N-acetyl-neuraminide alpha-2,8-sialyltransferase 1 | 210073_at | 4.96 | 0.23 |
| LSP1 | lymphocyte-specific protein 1 | 203523_at | 4.95 | 0.08 |
| KDELR1 | KDEL (Lys-Asp-Glu-Leu) endoplasmic reticulum protein retention receptor 1 | 200922_at | 4.94 | 0.00 |
| C1S | complement component 1, s subcomponent | 1555229_a_at | 4.94 | 0.00 |
| BMP8B | bone morphogenetic protein 8b | 235275_at | 4.94 | 2.06 |
| TAPBP | TAP binding protein (tapasin) | 1555565_s_at | 4.93 | 0.00 |
| ZNF107 | Zinc finger protein 107 | 243312_at | 4.92 | 0.63 |
| NBL1 | neuroblastoma, suppression of tumorigenicity 1 | 201621_at | 4.92 | 0.00 |
| MAF | v-maf musculoaponeurotic fibrosarcoma oncogene homolog (avian) | 209347_s_at | 4.92 | 0.00 |
| PPDPF | pancreatic progenitor cell differentiation and proliferation factor homolog (zebrafish) | 227994_x_at | 4.92 | 0.00 |
| CLPTM1 | cleft lip and palate associated transmembrane protein 1 | 201640_x_at | 4.91 | 0.00 |
| FAM126A | family with sequence similarity 126, member A | 244115_at | 4.91 | 0.08 |
| C10orf18 | chromosome 10 open reading frame 18 | 238795_at | 4.90 | 0.00 |
| DDR2 | Discoidin domain receptor tyrosine kinase 2 | 235631_at | 4.90 | 0.26 |
| GLCCI1 | glucocorticoid induced transcript 1 | 1560316_s_at | 4.90 | 0.00 |
| FYN | FYN oncogene related to SRC, FGR, YES | 216033_s_at | 4.90 | 0.00 |
| SLC6A8 | solute carrier family 6 (neurotransmitter transporter, creatine), member 8 | 213843_x_at | 4.88 | 0.08 |
| MCM3AP | minichromosome maintenance complex component 3 associated protein | 215581_s_at | 4.88 | 0.14 |
| NSMAF | neutral sphingomyelinase (N-SMase) activation associated factor | 1558775_s_at | 4.88 | 0.19 |
| AGRN | agrin | 217419_x_at | 4.88 | 0.00 |
| IBSP | integrin-binding sialoprotein | 236028_at | 4.88 | 0.26 |
| ETNK1 | Ethanolamine kinase 1 | 231576_at | 4.87 | 0.19 |
| CEP170 /// CEP170P1 | centrosomal protein 170kDa /// centrosomal protein 170kDa pseudogene 1 | 1552717_s_at | 4.87 | 0.00 |
| AKAP17A | A kinase (PRKA) anchor protein 17A | 228447_at | 4.86 | 0.23 |
| ERI3 | ERI1 exoribonuclease family member 3 | 208973_at | 4.85 | 0.00 |
| PVR | poliovirus receptor | 32699_s_at | 4.85 | 0.00 |
| CTTN | Cortactin | 227473_at | 4.85 | 0.08 |
| C1QTNF1 | C1q and tumor necrosis factor related protein 1 | 220975_s_at | 4.83 | 0.00 |
| LARP4 | La ribonucleoprotein domain family, member 4 | 238959_at | 4.83 | 0.19 |
| RAP1GDS1 | RAP1, GTP-GDP dissociation stimulator 1 | 229905_at | 4.81 | 0.00 |
| PTPRO | protein tyrosine phosphatase, receptor type, O | 211600_at | 4.81 | 0.00 |
| NCRNA00213 | non-protein coding RNA 213 | 1564069_at | 4.80 | 0.44 |
| ZNF275 | zinc finger protein 275 | 225382_at | 4.80 | 0.00 |
| LOC284998 | hypothetical LOC284998 | 239879_at | 4.80 | 0.86 |
| IL6ST | interleukin 6 signal transducer (gp130, oncostatin M receptor) | 204864_s_at | 4.79 | 0.55 |
| FOSL2 | FOS-like antigen 2 | 218881_s_at | 4.78 | 0.00 |
| LOC441167 | HCG1820801 | 1559620_at | 4.78 | 0.86 |
| ABR | active BCR-related gene | 214671_s_at | 4.77 | 0.00 |
| OAF | OAF homolog (Drosophila) | 225510_at | 4.76 | 0.14 |
| RNF126 | ring finger protein 126 | 205748_s_at | 4.76 | 0.00 |
| LRRC41 | leucine rich repeat containing 41 | 1555831_s_at | 4.76 | 0.00 |
| PLUNC | palate, lung and nasal epithelium associated | 220542_s_at | 4.76 | 0.23 |
| SOD2 | superoxide dismutase 2, mitochondrial | 221477_s_at | 4.75 | 0.00 |
| SPRED1 | sprouty-related, EVH1 domain containing 1 | 244439_at | 4.75 | 0.14 |
| ATP2A2 | ATPase, Ca++ transporting, cardiac muscle, slow twitch 2 | 212362_at | 4.73 | 0.00 |
| EGFR | epidermal growth factor receptor | 210984_x_at | 4.73 | 0.86 |
| SLC2A4RG | SLC2A4 regulator | 222650_s_at | 4.73 | 0.00 |
| CLIP3 | CAP-GLY domain containing linker protein 3 | 235243_at | 4.72 | 0.35 |
| MST4 | serine/threonine protein kinase MST4 | 224407_s_at | 4.71 | 0.63 |
| ENG | endoglin | 201808_s_at | 4.71 | 1.25 |
| C9orf16 | chromosome 9 open reading frame 16 | 204480_s_at | 4.70 | 0.00 |
| CAPN5 | calpain 5 | 205166_at | 4.70 | 0.00 |
| ELK3 | ELK3, ETS-domain protein (SRF accessory protein 2) | 206127_at | 4.69 | 0.00 |
| CENPB | centromere protein B, 80kDa | 212437_at | 4.69 | 0.00 |
| TGFBR1 | transforming growth factor, beta receptor 1 | 206943_at | 4.65 | 0.33 |
| JAM3 | junctional adhesion molecule 3 | 231720_s_at | 4.65 | 0.26 |
| PRAF2 | PRA1 domain family, member 2 | 203456_at | 4.65 | 0.00 |
| WBP2 | WW domain binding protein 2 | 209117_at | 4.64 | 0.00 |
| NCRNA00092 | non-protein coding RNA 92 | 1562733_at | 4.64 | 0.19 |
| DMPK | dystrophia myotonica-protein kinase | 37996_s_at | 4.63 | 0.00 |
| RRBP1 | ribosome binding protein 1 homolog 180kDa (dog) | 201203_s_at | 4.63 | 0.00 |
| SH3D19 | SH3 domain containing 19 | 1558647_at | 4.62 | 0.26 |
| KREMEN1 | kringle containing transmembrane protein 1 | 224534_at | 4.61 | 0.08 |
| HSPB7 | heat shock 27kDa protein family, member 7 (cardiovascular) | 218934_s_at | 4.60 | 0.35 |
| GJD3 | gap junction protein, delta 3, 31.9kDa | 1553511_at | 4.60 | 0.55 |
| SOST | sclerostin | 223869_at | 4.60 | 1.00 |
| C1orf61 | chromosome 1 open reading frame 61 | 205103_at | 4.60 | 0.44 |
| HSPA4 | heat shock 70kDa protein 4 | 211016_x_at | 4.59 | 0.00 |
| TRIM3 | tripartite motif-containing 3 | 204911_s_at | 4.58 | 0.08 |
| FKBP1A | FK506 binding protein 1A, 12kDa | 210186_s_at | 4.58 | 0.08 |
| C18orf25 | chromosome 18 open reading frame 25 | 217539_at | 4.58 | 0.14 |
| MYO18A /// TIAF1 | myosin XVIIIA /// TGFB1-induced anti-apoptotic factor 1 | 202039_at | 4.57 | 0.00 |
| EXOC5 | exocyst complex component 5 | 218748_s_at | 4.57 | 0.08 |
| MARCH6 | membrane-associated ring finger (C3HC4) 6 | 215512_at | 4.56 | 0.14 |
| ATP13A3 | ATPase type 13A3 | 219558_at | 4.55 | 0.26 |
| EHMT2 | euchromatic histone-lysine N-methyltransferase 2 | 202326_at | 4.54 | 0.00 |
| MTMR1 | myotubularin related protein 1 | 214975_s_at | 4.53 | 0.08 |
| DLGAP4 | discs, large (Drosophila) homolog-associated protein 4 | 202572_s_at | 4.53 | 0.00 |
| PPP1R15A | protein phosphatase 1, regulatory (inhibitor) subunit 15A | 37028_at | 4.53 | 0.00 |
| PADI2 | peptidyl arginine deiminase, type II | 209791_at | 4.52 | 1.67 |
| NR1D1 /// THRA | nuclear receptor subfamily 1, group D, member 1 /// thyroid hormone receptor, alpha (erythroblastic leukemia viral (v-erb-a) oncogene homolog, avian) | 204760_s_at | 4.52 | 0.08 |
| G6PC3 | glucose 6 phosphatase, catalytic, 3 | 221759_at | 4.52 | 0.26 |
| IGF2BP2 | insulin-like growth factor 2 mRNA binding protein 2 | 223963_s_at | 4.51 | 0.00 |
| ATG13 | ATG13 autophagy related 13 homolog (S. cerevisiae) | 203363_s_at | 4.51 | 0.00 |
| FLYWCH1 | FLYWCH-type zinc finger 1 | 234106_s_at | 4.51 | 0.00 |
| FKBP1A | FK506 binding protein 1A, 12kDa | 200709_at | 4.50 | 0.00 |
| DHX9 | DEAH (Asp-Glu-Ala-His) box polypeptide 9 | 212107_s_at | 4.48 | 3.21 |
| PDE4B | phosphodiesterase 4B, cAMP-specific | 211302_s_at | 4.48 | 0.08 |
| DIO2 | deiodinase, iodothyronine, type II | 231240_at | 4.48 | 0.00 |
| PRRX1 | paired related homeobox 1 | 205991_s_at | 4.48 | 0.08 |
| MBTPS2 | membrane-bound transcription factor peptidase, site 2 | 206473_at | 4.48 | 0.86 |
| TMEM214 | transmembrane protein 214 | 217899_at | 4.47 | 0.00 |
| TRUB1 | TruB pseudouridine (psi) synthase homolog 1 (E. coli) | 235447_at | 4.47 | 1.44 |
| PIP4K2A | Phosphatidylinositol-5-phosphate 4-kinase, type II, alpha | 229713_at | 4.47 | 0.86 |
| ZBTB7A | zinc finger and BTB domain containing 7A | 213299_at | 4.46 | 0.00 |
| PTPN23 | protein tyrosine phosphatase, non-receptor type 23 | 223149_s_at | 4.45 | 0.00 |
| PAK2 | p21 protein (Cdc42/Rac)-activated kinase 2 | 1559052_s_at | 4.45 | 0.00 |
| MINK1 | misshapen-like kinase 1 | 209241_x_at | 4.44 | 0.00 |
| GM2A | GM2 ganglioside activator | 33646_g_at | 4.44 | 0.08 |
| GOLGA6L4 /// PML | golgin A6 family-like 4 /// promyelocytic leukemia | 211014_s_at | 4.44 | 0.08 |
| USP34 | ubiquitin specific peptidase 34 | 242647_at | 4.43 | 0.14 |
| BSG | basigin (Ok blood group) | 208677_s_at | 4.43 | 0.00 |
| HP1BP3 | heterochromatin protein 1, binding protein 3 | 220633_s_at | 4.43 | 0.44 |
| ZDHHC8 | zinc finger, DHHC-type containing 8 | 225744_at | 4.42 | 0.00 |
| ARHGEF1 | Rho guanine nucleotide exchange factor (GEF) 1 | 203055_s_at | 4.42 | 0.00 |
| MUSK | muscle, skeletal, receptor tyrosine kinase | 207633_s_at | 4.42 | 1.44 |
| AKT1S1 | AKT1 substrate 1 (proline-rich) | 224982_at | 4.42 | 0.00 |
| PPP2R1A | protein phosphatase 2, regulatory subunit A, alpha | 200695_at | 4.42 | 0.00 |
| ELMO2 | engulfment and cell motility 2 | 220363_s_at | 4.41 | 0.14 |
| SLC37A4 | solute carrier family 37 (glucose-6-phosphate transporter), member 4 | 217289_s_at | 4.41 | 0.08 |
| RAD23A | RAD23 homolog A (S. cerevisiae) | 201039_s_at | 4.41 | 0.00 |
| P4HB | prolyl 4-hydroxylase, beta polypeptide | 1564494_s_at | 4.40 | 0.00 |
| LIMK2 | LIM domain kinase 2 | 217475_s_at | 4.40 | 0.08 |
| BMP1 | bone morphogenetic protein 1 | 207595_s_at | 4.39 | 0.00 |
| FNDC3A | fibronectin type III domain containing 3A | 215910_s_at | 4.39 | 0.23 |
| ANAPC5 | anaphase promoting complex subunit 5 | 208721_s_at | 4.38 | 0.00 |
| DOCK6 | dedicator of cytokinesis 6 | 222003_s_at | 4.38 | 0.00 |
| ATP6V1C1 | ATPase, H+ transporting, lysosomal 42kDa, V1 subunit C1 | 202873_at | 4.38 | 0.08 |
| ADAM15 | ADAM metallopeptidase domain 15 | 217007_s_at | 4.38 | 0.00 |
| ADD1 | adducin 1 (alpha) | 214726_x_at | 4.36 | 0.00 |
| SCARF2 | scavenger receptor class F, member 2 | 227557_at | 4.35 | 0.23 |
| IQSEC2 | IQ motif and Sec7 domain 2 | 229840_at | 4.35 | 0.14 |
| BCL9L | B-cell CLL/lymphoma 9-like | 227616_at | 4.35 | 0.00 |
| C2orf49 | chromosome 2 open reading frame 49 | 243093_at | 4.34 | 0.14 |
| GSK3B | glycogen synthase kinase 3 beta | 242336_at | 4.34 | 0.00 |
| AP1M1 | adaptor-related protein complex 1, mu 1 subunit | 223025_s_at | 4.34 | 0.00 |
| TTTY5 | testis-specific transcript, Y-linked 5 (non-protein coding) | 224040_at | 4.33 | 0.14 |
| KLHDC3 | kelch domain containing 3 | 208784_s_at | 4.33 | 0.00 |
| TRIP10 | thyroid hormone receptor interactor 10 | 202734_at | 4.33 | 0.00 |
| POM121L9P | POM121 membrane glycoprotein-like 9, pseudogene | 206819_at | 4.33 | 0.44 |
| ERLIN1 | ER lipid raft associated 1 | 202444_s_at | 4.33 | 0.08 |
| LAPTM5 | lysosomal protein transmembrane 5 | 201720_s_at | 4.32 | 0.23 |
| APPL2 | adaptor protein, phosphotyrosine interaction, PH domain and leucine zipper containing 2 | 218218_at | 4.32 | 0.14 |
| RELA | v-rel reticuloendotheliosis viral oncogene homolog A (avian) | 209878_s_at | 4.32 | 0.08 |
| CA12 | carbonic anhydrase XII | 210735_s_at | 4.29 | 0.00 |
| KIAA1632 | KIAA1632 | 232031_s_at | 4.29 | 0.14 |
| CD99L2 | CD99 molecule-like 2 | 1554758_a_at | 4.29 | 0.08 |
| PDLIM5 | PDZ and LIM domain 5 | 211681_s_at | 4.29 | 0.08 |
| SMPD1 | sphingomyelin phosphodiesterase 1, acid lysosomal | 216230_x_at | 4.28 | 0.00 |
| MRC2 | mannose receptor, C type 2 | 209280_at | 4.28 | 0.00 |
| C9orf16 | chromosome 9 open reading frame 16 | 222165_x_at | 4.28 | 0.00 |
| PAK2 | p21 protein (Cdc42/Rac)-activated kinase 2 | 208876_s_at | 4.28 | 0.00 |
| GLS | glutaminase | 223079_s_at | 4.28 | 0.00 |
| IGFBP4 | insulin-like growth factor binding protein 4 | 201508_at | 4.28 | 0.08 |
| NCOA2 | nuclear receptor coactivator 2 | 205732_s_at | 4.27 | 0.19 |
| XIST | X (inactive)-specific transcript (non-protein coding) | 235446_at | 4.26 | 0.44 |
| FOSL1 | FOS-like antigen 1 | 204420_at | 4.26 | 0.00 |
| AQP1 | aquaporin 1 (Colton blood group) | 209047_at | 4.26 | 1.00 |
| TNFRSF10D | tumor necrosis factor receptor superfamily, member 10d, decoy with truncated death domain | 210654_at | 4.25 | 0.26 |
| CERCAM | cerebral endothelial cell adhesion molecule | 224794_s_at | 4.25 | 0.00 |
| SNX19 | sorting nexin 19 | 1554986_a_at | 4.24 | 0.23 |
| PNPLA6 | patatin-like phospholipase domain containing 6 | 203718_at | 4.24 | 0.00 |
| HSP90AB1 | heat shock protein 90kDa alpha (cytosolic), class B member 1 | 1557910_at | 4.23 | 0.14 |
| SMARCB1 | SWI/SNF related, matrix associated, actin dependent regulator of chromatin, subfamily b, member 1 | 212167_s_at | 4.22 | 0.00 |
| COPA | coatomer protein complex, subunit alpha | 214336_s_at | 4.22 | 0.73 |
| SP1 | Sp1 transcription factor | 1553685_s_at | 4.19 | 0.08 |
| TMTC1 | transmembrane and tetratricopeptide repeat containing 1 | 244506_at | 4.19 | 0.55 |
| DRAP1 | DR1-associated protein 1 (negative cofactor 2 alpha) | 203258_at | 4.19 | 0.00 |
| CUX1 | cut-like homeobox 1 | 227069_at | 4.18 | 0.08 |
| TFRC | transferrin receptor (p90, CD71) | 237215_s_at | 4.18 | 1.25 |
| ANKHD1 /// ANKHD1-EIF4EBP3 | ankyrin repeat and KH domain containing 1 /// ANKHD1-EIF4EBP3 readthrough | 233292_s_at | 4.17 | 0.00 |
| PEG10 | paternally expressed 10 | 212094_at | 4.17 | 0.73 |
| COL8A1 | collagen, type VIII, alpha 1 | 221152_at | 4.17 | 0.33 |
| BAT2L1 | HLA-B associated transcript 2-like 1 | 212069_s_at | 4.17 | 0.08 |
| IGSF8 | immunoglobulin superfamily, member 8 | 225025_at | 4.17 | 0.00 |
| SLC17A7 | solute carrier family 17 (sodium-dependent inorganic phosphate cotransporter), member 7 | 204230_s_at | 4.16 | 0.08 |
| PCDHGA1 /// PCDHGA10 /// PCDHGA11 /// PCDHGA12 /// PCDHGA2 /// PCDHGA3 /// PCDHGA4 /// PCDHGA5 /// PCDHGA6 /// PCDHGA7 /// PCDHGA8 /// PCDHGA9 /// PCDHGB1 /// PCDHGB2 /// PCDHGB3 /// PCDHGB4 /// PCDHGB5 /// PCDHGB6 /// PCDHGB7 /// PCDHGC3 /// PCDHGC4 /// PCDHGC5 | protocadherin gamma subfamily A, 1 /// protocadherin gamma subfamily A, 10 /// protocadherin gamma subfamily A, 11 /// protocadherin gamma subfamily A, 12 /// protocadherin gamma subfamily A, 2 /// protocadherin gamma subfamily A, 3 /// protocadherin gamma subfamily A, 4 /// protocadherin gamma subfamily A, 5 /// protocadherin gamma subfamily A, 6 /// protocadherin gamma subfamily A, 7 /// protocadherin gamma subfamily A, 8 /// protocadherin gamma subfamily A, 9 /// protocadherin gamma subfamily B, 1 /// protocadherin gamma subfamily B, 2 /// protocadherin gamma subfamily B, 3 /// protocadherin gamma subfamily B, 4 /// protocadherin gamma subfamily B, 5 /// protocadherin gamma subfamily B, 6 /// protocadherin gamma subfamily B, 7 /// protocadherin gamma subfamily C, 3 /// protocadherin gamma subfamily C, 4 /// protocadherin gamma subfamily C, 5 | 209079_x_at | 4.16 | 0.00 |
| PRKAB1 | protein kinase, AMP-activated, beta 1 non-catalytic subunit | 201835_s_at | 4.16 | 0.00 |
| TSC2 | tuberous sclerosis 2 | 215735_s_at | 4.16 | 0.00 |
| FN1 | fibronectin 1 | 214701_s_at | 4.16 | 0.33 |
| AGPAT3 | 1-acylglycerol-3-phosphate O-acyltransferase 3 | 219723_x_at | 4.14 | 0.23 |
| GIMAP8 | GTPase, IMAP family member 8 | 235306_at | 4.14 | 0.55 |
| ELK1 | ELK1, member of ETS oncogene family | 210376_x_at | 4.14 | 0.00 |
| PIK3C2A | phosphoinositide-3-kinase, class 2, alpha polypeptide | 1553694_a_at | 4.14 | 0.08 |
| TTC37 | tetratricopeptide repeat domain 37 | 1554029_a_at | 4.14 | 0.44 |
| SLC17A9 | solute carrier family 17, member 9 | 232922_s_at | 4.14 | 0.00 |
| DDA1 | DET1 and DDB1 associated 1 | 218260_at | 4.14 | 0.23 |
| UBAP2L | ubiquitin associated protein 2-like | 201378_s_at | 4.14 | 0.00 |
| WDR1 | WD repeat domain 1 | 210935_s_at | 4.13 | 0.08 |
| ACO2 | aconitase 2, mitochondrial | 200793_s_at | 4.12 | 0.00 |
| SRGAP1 | SLIT-ROBO Rho GTPase activating protein 1 | 1555875_at | 4.12 | 0.00 |
| ABCC1 | ATP-binding cassette, sub-family C (CFTR/MRP), member 1 | 202805_s_at | 4.12 | 0.00 |
| B4GALT1 | UDP-Gal:betaGlcNAc beta 1,4- galactosyltransferase, polypeptide 1 | 216627_s_at | 4.12 | 0.63 |
| FOXO3 /// FOXO3B | forkhead box O3 /// forkhead box O3B pseudogene | 210655_s_at | 4.12 | 0.08 |
| DMWD | dystrophia myotonica, WD repeat containing | 213231_at | 4.11 | 0.26 |
| TBC1D1 | TBC1 (tre-2/USP6, BUB2, cdc16) domain family, member 1 | 1568713_a_at | 4.11 | 0.35 |
| NOTCH3 | notch 3 | 203238_s_at | 4.11 | 0.08 |
| NFKB2 | nuclear factor of kappa light polypeptide gene enhancer in B-cells 2 (p49/p100) | 207535_s_at | 4.11 | 0.00 |
| COL4A2 | collagen, type IV, alpha 2 | 211966_at | 4.11 | 0.08 |
| LOC389906 | hypothetical LOC389906 | 1569629_x_at | 4.11 | 0.19 |
| ADCY9 | adenylate cyclase 9 | 204498_s_at | 4.10 | 0.00 |
| SLC1A5 | solute carrier family 1 (neutral amino acid transporter), member 5 | 208916_at | 4.10 | 0.00 |
| MOV10 | Mov10, Moloney leukemia virus 10, homolog (mouse) | 233917_s_at | 4.10 | 0.14 |
| MAF1 | MAF1 homolog (S. cerevisiae) | 222998_at | 4.09 | 0.00 |
| CUL7 | cullin 7 | 203558_at | 4.09 | 0.00 |
| LIX1L | Lix1 homolog (mouse)-like | 235036_at | 4.09 | 0.00 |
| ATP6V1G2 /// BAT1 | ATPase, H+ transporting, lysosomal 13kDa, V1 subunit G2 /// HLA-B associated transcript 1 | 200041_s_at | 4.09 | 0.00 |
| PIK3R2 | phosphoinositide-3-kinase, regulatory subunit 2 (beta) | 207105_s_at | 4.08 | 0.08 |
| CHPF | chondroitin polymerizing factor | 202175_at | 4.08 | 0.00 |
| SLC16A1 | solute carrier family 16, member 1 (monocarboxylic acid transporter 1) | 1557918_s_at | 4.08 | 0.26 |
| HHLA3 | HERV-H LTR-associating 3 | 234665_x_at | 4.08 | 0.00 |
| MTSS1L | metastasis suppressor 1-like | 1555894_s_at | 4.07 | 0.08 |
| ARFGAP1 | ADP-ribosylation factor GTPase activating protein 1 | 234001_s_at | 4.07 | 0.00 |
| LOC100130522 | hypothetical LOC100130522 | 1557590_at | 4.06 | 0.73 |
| EHD4 | EH-domain containing 4 | 233660_at | 4.06 | 0.00 |
| NFKB2 | nuclear factor of kappa light polypeptide gene enhancer in B-cells 2 (p49/p100) | 209636_at | 4.06 | 0.00 |
| CTDSPL2 | CTD (carboxy-terminal domain, RNA polymerase II, polypeptide A) small phosphatase like 2 | 1555106_a_at | 4.05 | 0.14 |
| FBXO44 | F-box protein 44 | 223517_at | 4.05 | 0.00 |
| PDLIM7 | PDZ and LIM domain 7 (enigma) | 214121_x_at | 4.05 | 0.00 |
| FASN | fatty acid synthase | 212218_s_at | 4.04 | 0.00 |
| YTHDF3 | YTH domain family, member 3 | 1564053_a_at | 4.04 | 0.44 |
| KPNA4 | karyopherin alpha 4 (importin alpha 3) | 209653_at | 4.04 | 0.19 |
| ANKFY1 | ankyrin repeat and FYVE domain containing 1 | 219868_s_at | 4.03 | 0.00 |
| TMOD3 | tropomodulin 3 (ubiquitous) | 220800_s_at | 4.03 | 0.14 |
| RAB27B | RAB27B, member RAS oncogene family | 207018_s_at | 4.02 | 0.14 |
| RAB3B | RAB3B, member RAS oncogene family | 242629_at | 4.01 | 0.08 |
| GDNF | glial cell derived neurotrophic factor | 230090_at | 4.00 | 0.19 |
| KDM6B | lysine (K)-specific demethylase 6B | 41386_i_at | 4.00 | 0.08 |
| ABHD14B | abhydrolase domain containing 14B | 224821_at | 3.99 | 0.00 |
| MET | met proto-oncogene (hepatocyte growth factor receptor) | 211599_x_at | 3.99 | 0.55 |
| MRPL38 | mitochondrial ribosomal protein L38 | 225103_at | 3.97 | 0.00 |
| ACTN4 | actinin, alpha 4 | 200601_at | 3.97 | 0.19 |
| KLHL22 | kelch-like 22 (Drosophila) | 221837_at | 3.96 | 0.08 |
| PVR | poliovirus receptor | 216283_s_at | 3.96 | 0.00 |
| ATF7 | activating transcription factor 7 | 244587_at | 3.96 | 0.00 |
| REEP3 | receptor accessory protein 3 | 235016_at | 3.96 | 0.35 |
| DAPK3 | death-associated protein kinase 3 | 203890_s_at | 3.96 | 0.00 |
| ANGPTL4 | angiopoietin-like 4 | 223333_s_at | 3.95 | 0.26 |
| TEAD2 | TEA domain family member 2 | 238322_s_at | 3.95 | 0.00 |
| STX16 | syntaxin 16 | 1558249_s_at | 3.95 | 0.08 |
| CDT1 | chromatin licensing and DNA replication factor 1 | 209832_s_at | 3.94 | 0.86 |
| PKNOX1 | PBX/knotted 1 homeobox 1 | 216004_s_at | 3.94 | 1.25 |
| GPR65 | G protein-coupled receptor 65 | 214467_at | 3.94 | 6.81 |
| STAT6 | signal transducer and activator of transcription 6, interleukin-4 induced | 201332_s_at | 3.94 | 0.00 |
| HDLBP | high density lipoprotein binding protein | 222916_s_at | 3.93 | 0.00 |
| ARNT | aryl hydrocarbon receptor nuclear translocator | 210828_s_at | 3.92 | 0.19 |
| SGSM2 | small G protein signaling modulator 2 | 212319_at | 3.92 | 0.00 |
| PKN1 | protein kinase N1 | 202161_at | 3.92 | 0.00 |
| MTMR3 | myotubularin related protein 3 | 202198_s_at | 3.92 | 0.19 |
| ADRBK1 | adrenergic, beta, receptor kinase 1 | 201401_s_at | 3.92 | 0.00 |
| TMEM121 | transmembrane protein 121 | 222914_s_at | 3.91 | 0.55 |
| GLT25D1 | glycosyltransferase 25 domain containing 1 | 222644_s_at | 3.91 | 0.08 |
| LARP1 | La ribonucleoprotein domain family, member 1 | 210966_x_at | 3.91 | 0.00 |
| IL17RC | interleukin 17 receptor C | 64440_at | 3.90 | 0.00 |
| CA9 | carbonic anhydrase IX | 205199_at | 3.90 | 0.86 |
| PSEN1 | presenilin 1 | 238816_at | 3.90 | 0.00 |
| INO80B | INO80 complex subunit B | 65133_i_at | 3.90 | 0.00 |
| ZMAT3 | zinc finger, matrin-type 3 | 1555609_a_at | 3.89 | 0.08 |
| PITPNC1 | phosphatidylinositol transfer protein, cytoplasmic 1 | 1568949_at | 3.88 | 0.14 |
| CTF1 | cardiotrophin 1 | 206813_at | 3.88 | 0.00 |
| HTRA4 | HtrA serine peptidase 4 | 1553706_at | 3.87 | 0.44 |
| ZFHX3 | zinc finger homeobox 3 | 208033_s_at | 3.87 | 0.08 |
| GALNT4 /// POC1B | UDP-N-acetyl-alpha-D-galactosamine:polypeptide N-acetylgalactosaminyltransferase 4 (GalNAc-T4) /// POC1 centriolar protein homolog B (Chlamydomonas) | 220442_at | 3.87 | 0.26 |
| CHST1 | carbohydrate (keratan sulfate Gal-6) sulfotransferase 1 | 205567_at | 3.87 | 1.67 |
| PATL1 | protein associated with topoisomerase II homolog 1 (yeast) | 235235_s_at | 3.87 | 0.35 |
| RASA3 | RAS p21 protein activator 3 | 206220_s_at | 3.87 | 0.00 |
| AGRN | agrin | 212285_s_at | 3.87 | 0.14 |
| TYRO3 | TYRO3 protein tyrosine kinase | 211431_s_at | 3.87 | 0.26 |
| EPB41L1 | erythrocyte membrane protein band 4.1-like 1 | 212336_at | 3.86 | 0.08 |
| CITED2 | Cbp/p300-interacting transactivator, with Glu/Asp-rich carboxy-terminal domain, 2 | 207980_s_at | 3.85 | 0.08 |
| C19orf29 | chromosome 19 open reading frame 29 | 215954_s_at | 3.85 | 0.00 |
| RARG | retinoic acid receptor, gamma | 204189_at | 3.85 | 0.26 |
| PRUNE | prune homolog (Drosophila) | 209599_s_at | 3.84 | 0.86 |
| TMPO | thymopoietin | 209754_s_at | 3.83 | 0.35 |
| PLEKHA2 | pleckstrin homology domain containing, family A (phosphoinositide binding specific) member 2 | 238013_at | 3.83 | 0.08 |
| MATR3 | matrin 3 | 1558093_s_at | 3.82 | 0.14 |
| CIZ1 | CDKN1A interacting zinc finger protein 1 | 211358_s_at | 3.82 | 0.00 |
| SUPT6H | suppressor of Ty 6 homolog (S. cerevisiae) | 1554311_a_at | 3.82 | 0.14 |
| NF2 | neurofibromin 2 (merlin) | 238618_at | 3.82 | 1.00 |
| APCDD1 | adenomatosis polyposis coli down-regulated 1 | 225016_at | 3.82 | 1.44 |
| C1QTNF6 | C1q and tumor necrosis factor related protein 6 | 242444_at | 3.82 | 0.00 |
| LRRC58 | leucine rich repeat containing 58 | 238506_at | 3.81 | 0.08 |
| PTPN11 | protein tyrosine phosphatase, non-receptor type 11 | 205868_s_at | 3.81 | 0.35 |
| ZER1 | zer-1 homolog (C. elegans) | 202456_s_at | 3.80 | 0.19 |
| CPNE3 | copine III | 202118_s_at | 3.80 | 0.00 |
| C10orf10 | chromosome 10 open reading frame 10 | 209182_s_at | 3.80 | 0.35 |
| STIP1 | stress-induced-phosphoprotein 1 | 212009_s_at | 3.79 | 0.26 |
| SLC2A5 | solute carrier family 2 (facilitated glucose/fructose transporter), member 5 | 204429_s_at | 3.79 | 0.19 |
| MFN2 | mitofusin 2 | 216205_s_at | 3.79 | 0.08 |
| TMEM49 | transmembrane protein 49 | 1569003_at | 3.79 | 0.00 |
| SNX27 | sorting nexin family member 27 | 221006_s_at | 3.78 | 0.00 |
| C5orf22 | chromosome 5 open reading frame 22 | 1552660_a_at | 3.78 | 0.19 |
| CDK13 | cyclin-dependent kinase 13 | 207319_s_at | 3.78 | 0.14 |
| GRLF1 | glucocorticoid receptor DNA binding factor 1 | 202046_s_at | 3.78 | 0.00 |
| CAB39 | calcium binding protein 39 | 224311_s_at | 3.78 | 0.00 |
| MAP2K2 | mitogen-activated protein kinase kinase 2 | 213490_s_at | 3.77 | 0.08 |
| SYPL2 | synaptophysin-like 2 | 230611_at | 3.77 | 0.55 |
| SERINC2 | serine incorporator 2 | 224762_at | 3.77 | 1.67 |
| CXCL5 | chemokine (C-X-C motif) ligand 5 | 215101_s_at | 3.76 | 0.55 |
| TRIB2 | tribbles homolog 2 (Drosophila) | 202479_s_at | 3.76 | 0.00 |
| ILK | integrin-linked kinase | 201234_at | 3.75 | 0.00 |
| PDPN | podoplanin | 226658_at | 3.75 | 0.26 |
| TRIB3 | tribbles homolog 3 (Drosophila) | 1555788_a_at | 3.75 | 1.67 |
| SLMAP | sarcolemma associated protein | 224149_x_at | 3.75 | 0.19 |
| DAZAP2 | DAZ associated protein 2 | 212595_s_at | 3.75 | 0.00 |
| FLNA | filamin A, alpha | 213746_s_at | 3.75 | 0.00 |
| KLF11 | Kruppel-like factor 11 | 1553137_s_at | 3.75 | 0.33 |
| ASMTL | acetylserotonin O-methyltransferase-like | 36554_at | 3.75 | 0.00 |
| ZNF580 | zinc finger protein 580 | 220748_s_at | 3.74 | 0.08 |
| MBTPS1 | membrane-bound transcription factor peptidase, site 1 | 217543_s_at | 3.74 | 0.00 |
| PFKL | phosphofructokinase, liver | 201102_s_at | 3.74 | 0.08 |
| MOSPD3 | motile sperm domain containing 3 | 219070_s_at | 3.73 | 0.00 |
| CNOT1 | CCR4-NOT transcription complex, subunit 1 | 200861_at | 3.73 | 0.00 |
| EIF4A2 | eukaryotic translation initiation factor 4A2 | 1555996_s_at | 3.72 | 0.55 |
| IKBKB | inhibitor of kappa light polypeptide gene enhancer in B-cells, kinase beta | 211027_s_at | 3.71 | 1.25 |
| MLL2 | myeloid/lymphoid or mixed-lineage leukemia 2 | 231974_at | 3.71 | 0.00 |
| HHLA3 | HERV-H LTR-associating 3 | 220387_s_at | 3.71 | 0.08 |
| LAPTM4B | lysosomal protein transmembrane 4 beta | 1554679_a_at | 3.71 | 0.19 |
| MAP2K2 | mitogen-activated protein kinase kinase 2 | 202424_at | 3.71 | 0.00 |
| TAB3 | TGF-beta activated kinase 1/MAP3K7 binding protein 3 | 1552927_at | 3.71 | 0.26 |
| HIPK3 | homeodomain interacting protein kinase 3 | 207764_s_at | 3.71 | 1.25 |
| SIN3B | SIN3 homolog B, transcription regulator (yeast) | 209352_s_at | 3.71 | 0.08 |
| BIN1 | bridging integrator 1 | 214439_x_at | 3.69 | 0.00 |
| SEPN1 | selenoprotein N, 1 | 224659_at | 3.69 | 0.00 |
| FGFRL1 | fibroblast growth factor receptor-like 1 | 223321_s_at | 3.69 | 0.00 |
| RBM33 | RNA binding motif protein 33 | 238801_at | 3.68 | 0.33 |
| SAR1B | SAR1 homolog B (S. cerevisiae) | 230397_at | 3.68 | 0.08 |
| SMCR8 | Smith-Magenis syndrome chromosome region, candidate 8 | 1557986_s_at | 3.67 | 0.23 |
| FGD6 | FYVE, RhoGEF and PH domain containing 6 | 1555137_a_at | 3.67 | 1.00 |
| CRTC1 | CREB regulated transcription coactivator 1 | 207159_x_at | 3.67 | 0.19 |
| RC3H2 | ring finger and CCCH-type domains 2 | 238421_at | 3.66 | 0.44 |
| LOC100130776 | hypothetical LOC100130776 | 1555907_at | 3.66 | 0.19 |
| RTKN | rhotekin | 225150_s_at | 3.65 | 0.26 |
| TGM2 | transglutaminase 2 (C polypeptide, protein-glutamine-gamma-glutamyltransferase) | 211573_x_at | 3.65 | 0.55 |
| MMP3 | matrix metallopeptidase 3 (stromelysin 1, progelatinase) | 205828_at | 3.65 | 0.44 |
| GGA2 | golgi-associated, gamma adaptin ear containing, ARF binding protein 2 | 214190_x_at | 3.64 | 0.26 |
| TK2 | Thymidine kinase 2, mitochondrial | 230313_at | 3.64 | 0.33 |
| CBFB | core-binding factor, beta subunit | 206788_s_at | 3.63 | 0.14 |
| RBAK | RB-associated KRAB zinc finger | 1553122_s_at | 3.63 | 0.08 |
| ZFR | zinc finger RNA binding protein | 213286_at | 3.63 | 0.14 |
| DUSP1 | dual specificity phosphatase 1 | 201044_x_at | 3.63 | 0.73 |
| TTC7A | tetratricopeptide repeat domain 7A | 224924_at | 3.63 | 0.14 |
| SMG7 | Smg-7 homolog, nonsense mediated mRNA decay factor (C. elegans) | 217189_s_at | 3.63 | 0.08 |
| RRAD | Ras-related associated with diabetes | 204802_at | 3.62 | 1.67 |
| C20orf117 | chromosome 20 open reading frame 117 | 225473_at | 3.62 | 0.00 |
| FLJ30430 | hypothetical protein FLJ30430 | 1552942_at | 3.62 | 1.67 |
| OTUB1 | OTU domain, ubiquitin aldehyde binding 1 | 201246_s_at | 3.62 | 0.33 |
| HLA-DRA | major histocompatibility complex, class II, DR alpha | 208894_at | 3.62 | 1.44 |
| MYL9 | myosin, light chain 9, regulatory | 201058_s_at | 3.62 | 0.00 |
| PRKCSH | protein kinase C substrate 80K-H | 214080_x_at | 3.61 | 0.00 |
| SPTBN1 | spectrin, beta, non-erythrocytic 1 | 215918_s_at | 3.61 | 0.33 |
| TMTC1 | transmembrane and tetratricopeptide repeat containing 1 | 226931_at | 3.61 | 0.23 |
| STC1 | stanniocalcin 1 | 204596_s_at | 3.61 | 0.55 |
| TCF7 | transcription factor 7 (T-cell specific, HMG-box) | 205254_x_at | 3.61 | 0.08 |
| MED15 | mediator complex subunit 15 | 222175_s_at | 3.60 | 0.00 |
| RALGPS2 | Ral GEF with PH domain and SH3 binding motif 2 | 242458_at | 3.60 | 0.33 |
| ANKH | ankylosis, progressive homolog (mouse) | 223094_s_at | 3.60 | 0.26 |
| SYNJ1 | synaptojanin 1 | 207594_s_at | 3.60 | 0.19 |
| KRTAP7-1 | keratin associated protein 7-1 (gene/pseudogene) | 1564960_at | 3.59 | 2.72 |
| TUBGCP2 | tubulin, gamma complex associated protein 2 | 202476_s_at | 3.58 | 0.19 |
| PSMF1 | proteasome (prosome, macropain) inhibitor subunit 1 (PI31) | 201052_s_at | 3.58 | 0.00 |
| PEX19 | peroxisomal biogenesis factor 19 | 201706_s_at | 3.58 | 0.00 |
| CDH10 | cadherin 10, type 2 (T2-cadherin) | 220115_s_at | 3.58 | 0.73 |
| CTNNA1 | catenin (cadherin-associated protein), alpha 1, 102kDa | 1558214_s_at | 3.58 | 0.26 |
| TMEM33 | transmembrane protein 33 | 238831_at | 3.58 | 0.14 |
| MAP1S | microtubule-associated protein 1S | 218522_s_at | 3.57 | 0.00 |
| GPX3 | glutathione peroxidase 3 (plasma) | 214091_s_at | 3.57 | 0.14 |
| CAMK2G | calcium/calmodulin-dependent protein kinase II gamma | 212669_at | 3.57 | 0.00 |
| AAAS | achalasia, adrenocortical insufficiency, alacrimia | 218075_at | 3.57 | 0.08 |
| CD46 | CD46 molecule, complement regulatory protein | 207549_x_at | 3.57 | 0.00 |
| CALB2 | calbindin 2 | 205428_s_at | 3.57 | 0.55 |
| GSTM1 | glutathione S-transferase mu 1 | 204550_x_at | 3.57 | 0.19 |
| SLC5A3 | solute carrier family 5 (sodium/myo-inositol cotransporter), member 3 | 213167_s_at | 3.57 | 0.35 |
| LOC100510314 /// LOC100510451 /// RRP7A /// RRP7B | ribosomal RNA-processing protein 7 homolog A-like /// ribosomal RNA-processing protein 7 homolog A-like /// ribosomal RNA processing 7 homolog A (S. cerevisiae) /// ribosomal RNA processing 7 homolog B (S. cerevisiae) | 202938_x_at | 3.56 | 0.35 |
| ELL2 | elongation factor, RNA polymerase II, 2 | 214446_at | 3.56 | 0.00 |
| UQCC | ubiquinol-cytochrome c reductase complex chaperone | 222470_s_at | 3.56 | 0.26 |
| FOXP1 | forkhead box P1 | 223936_s_at | 3.56 | 0.33 |
| SH3GLB2 | SH3-domain GRB2-like endophilin B2 | 224907_s_at | 3.56 | 0.00 |
| NPIPL2 /// NPIPL3 /// PDXDC2P | nuclear pore complex interacting protein-like 2 /// nuclear pore complex interacting protein-like 3 /// pyridoxal-dependent decarboxylase domain containing 2, pseudogene | 215920_s_at | 3.55 | 0.08 |
| KYNU | kynureninase (L-kynurenine hydrolase) | 204385_at | 3.55 | 4.15 |
| SEL1L3 | sel-1 suppressor of lin-12-like 3 (C. elegans) | 212311_at | 3.54 | 0.19 |
| SLC35A2 | solute carrier family 35 (UDP-galactose transporter), member A2 | 207440_at | 3.54 | 0.08 |
| LRP5 | low density lipoprotein receptor-related protein 5 | 209468_at | 3.54 | 0.33 |
| RTTN | rotatin | 1557388_at | 3.54 | 1.00 |
| SPG7 | spastic paraplegia 7 (pure and complicated autosomal recessive) | 230884_s_at | 3.54 | 0.26 |
| FLVCR2 | feline leukemia virus subgroup C cellular receptor family, member 2 | 222866_s_at | 3.53 | 0.63 |
| LIG3 | ligase III, DNA, ATP-dependent | 207348_s_at | 3.53 | 0.23 |
| ZBTB10 | Zinc finger and BTB domain containing 10 | 235491_at | 3.53 | 0.73 |
| BCOR | BCL6 corepressor | 223916_s_at | 3.52 | 0.63 |
| PRELP | proline/arginine-rich end leucine-rich repeat protein | 204223_at | 3.52 | 0.35 |
| DBN1 | drebrin 1 | 217025_s_at | 3.52 | 0.00 |
| ALDH3A2 | aldehyde dehydrogenase 3 family, member A2 | 210544_s_at | 3.52 | 0.26 |
| TMEM148 | transmembrane protein 148 | 1552856_at | 3.52 | 0.26 |
| CSRNP2 | cysteine-serine-rich nuclear protein 2 | 225042_s_at | 3.51 | 0.19 |
| CMTM4 | CKLF-like MARVEL transmembrane domain containing 4 | 225009_at | 3.51 | 0.00 |
| GPR137 | G protein-coupled receptor 137 | 43934_at | 3.51 | 0.00 |
| HTATSF1 | HIV-1 Tat specific factor 1 | 202601_s_at | 3.50 | 0.08 |
| CALU | calumenin | 200756_x_at | 3.50 | 0.14 |
| ECM1 | extracellular matrix protein 1 | 209365_s_at | 3.50 | 0.14 |
| FAM86C | family with sequence similarity 86, member C | 220353_at | 3.50 | 0.35 |
| ACRV1 | acrosomal vesicle protein 1 | 207969_x_at | 3.50 | 1.67 |
| HGS | hepatocyte growth factor-regulated tyrosine kinase substrate | 210428_s_at | 3.50 | 0.00 |
| SLC7A1 | solute carrier family 7 (cationic amino acid transporter, y+ system), member 1 | 206566_at | 3.50 | 0.23 |
| ERF | Ets2 repressor factor | 203643_at | 3.50 | 0.08 |
| TPCN1 | two pore segment channel 1 | 217914_at | 3.50 | 0.26 |
| OTUD5 | OTU domain containing 5 | 1555426_a_at | 3.50 | 0.00 |
| HPS3 | Hermansky-Pudlak syndrome 3 | 238539_at | 3.49 | 0.23 |
| RANGAP1 | Ran GTPase activating protein 1 | 1553535_a_at | 3.49 | 0.33 |
| UBTF | upstream binding transcription factor, RNA polymerase I | 1558215_s_at | 3.49 | 0.23 |
| SP1 | Sp1 transcription factor | 214732_at | 3.49 | 0.35 |
| BIN1 | bridging integrator 1 | 210202_s_at | 3.49 | 0.14 |
| ZNF385A | zinc finger protein 385A | 226111_s_at | 3.49 | 0.73 |
| KCTD5 | potassium channel tetramerisation domain containing 5 | 222645_s_at | 3.48 | 0.00 |
| PDE7A | phosphodiesterase 7A | 224046_s_at | 3.48 | 0.14 |
| B4GALT1 | UDP-Gal:betaGlcNAc beta 1,4- galactosyltransferase, polypeptide 1 | 211631_x_at | 3.48 | 0.44 |
| PPDPF | pancreatic progenitor cell differentiation and proliferation factor homolog (zebrafish) | 233571_x_at | 3.47 | 0.08 |
| RARS2 | arginyl-tRNA synthetase 2, mitochondrial | 1561048_at | 3.47 | 0.23 |
| OGDH | oxoglutarate (alpha-ketoglutarate) dehydrogenase (lipoamide) | 201282_at | 3.47 | 0.23 |
| P2RY4 | pyrimidinergic receptor P2Y, G-protein coupled, 4 | 221466_at | 3.46 | 2.06 |
| OSMR | oncostatin M receptor | 205729_at | 3.46 | 0.23 |
| ZNF192 | zinc finger protein 192 | 206579_at | 3.46 | 2.06 |
| CLIP2 | CAP-GLY domain containing linker protein 2 | 211031_s_at | 3.46 | 0.08 |
| SPSB1 | splA/ryanodine receptor domain and SOCS box containing 1 | 226075_at | 3.46 | 0.08 |
| HFE | hemochromatosis | 211863_x_at | 3.46 | 0.19 |
| XIST | X (inactive)-specific transcript (non-protein coding) | 224589_at | 3.46 | 0.73 |
| C17orf63 | chromosome 17 open reading frame 63 | 222641_s_at | 3.46 | 0.08 |
| DDX11 /// DDX12 /// LOC642846 | DEAD/H (Asp-Glu-Ala-Asp/His) box polypeptide 11 /// DEAD/H (Asp-Glu-Ala-Asp/His) box polypeptide 12 /// DEAD/H (Asp-Glu-Ala-Asp/His) box polypeptide 11-like | 213378_s_at | 3.45 | 0.14 |
| GSTM2 | glutathione S-transferase mu 2 (muscle) | 204418_x_at | 3.45 | 0.08 |
| KIF1C | kinesin family member 1C | 209244_s_at | 3.45 | 0.63 |
| SEZ6L2 | seizure related 6 homolog (mouse)-like 2 | 233337_s_at | 3.45 | 0.73 |
| RALGPS2 | Ral GEF with PH domain and SH3 binding motif 2 | 232112_at | 3.45 | 0.44 |
| KBTBD4 | kelch repeat and BTB (POZ) domain containing 4 | 223765_s_at | 3.45 | 2.45 |
| VDAC1 | voltage-dependent anion channel 1 | 217139_at | 3.45 | 0.86 |
| ARSD | arylsulfatase D | 206831_s_at | 3.44 | 0.33 |
| PDE4DIP | phosphodiesterase 4D interacting protein | 212392_s_at | 3.44 | 0.26 |
| PATZ1 | POZ (BTB) and AT hook containing zinc finger 1 | 211391_s_at | 3.44 | 0.14 |
| PICALM | phosphatidylinositol binding clathrin assembly protein | 215236_s_at | 3.44 | 0.35 |
| PHF20 | PHD finger protein 20 | 209423_s_at | 3.43 | 0.08 |
| ERC1 | ELKS/RAB6-interacting/CAST family member 1 | 1552663_a_at | 3.43 | 0.19 |
| ARID2 | AT rich interactive domain 2 (ARID, RFX-like) | 231090_s_at | 3.43 | 0.14 |
| ABCD3 | ATP-binding cassette, sub-family D (ALD), member 3 | 1554878_a_at | 3.42 | 1.25 |
| TSKU | tsukushi small leucine rich proteoglycan homolog (Xenopus laevis) | 218245_at | 3.42 | 0.14 |
| RRAD | Ras-related associated with diabetes | 204803_s_at | 3.42 | 1.00 |
| BTN3A1 | butyrophilin, subfamily 3, member A1 | 207485_x_at | 3.41 | 0.23 |
| ULBP2 | UL16 binding protein 2 | 221291_at | 3.41 | 0.19 |
| CUL7 | cullin 7 | 241747_s_at | 3.41 | 0.35 |
| NLGN2 | neuroligin 2 | 226288_s_at | 3.41 | 0.08 |
| CHCHD10 | coiled-coil-helix-coiled-coil-helix domain containing 10 | 224932_at | 3.41 | 0.14 |
| DNAJC13 | DnaJ (Hsp40) homolog, subfamily C, member 13 | 1560020_at | 3.41 | 1.00 |
| BMP1 | bone morphogenetic protein 1 | 205574_x_at | 3.40 | 0.00 |
| HTR2A | 5-hydroxytryptamine (serotonin) receptor 2A | 211616_s_at | 3.40 | 0.86 |
| FZR1 | fizzy/cell division cycle 20 related 1 (Drosophila) | 211865_s_at | 3.40 | 0.14 |
| RCE1 | RCE1 homolog, prenyl protein peptidase (S. cerevisiae) | 205333_s_at | 3.40 | 0.08 |
| HLA-DRA | major histocompatibility complex, class II, DR alpha | 210982_s_at | 3.40 | 1.44 |
| AES | amino-terminal enhancer of split | 217729_s_at | 3.40 | 0.23 |
| TNKS1BP1 | tankyrase 1 binding protein 1, 182kDa | 224792_at | 3.40 | 0.08 |
| ANAPC2 | anaphase promoting complex subunit 2 | 218555_at | 3.40 | 0.08 |
| ANKH | Ankylosis, progressive homolog (mouse) | 1560369_at | 3.40 | 0.35 |
| MAPK1 | mitogen-activated protein kinase 1 | 208351_s_at | 3.39 | 0.33 |
| MPHOSPH9 | M-phase phosphoprotein 9 | 237158_s_at | 3.39 | 0.33 |
| PDE7B | phosphodiesterase 7B | 220343_at | 3.39 | 0.55 |
| PIP5K1C | phosphatidylinositol-4-phosphate 5-kinase, type I, gamma | 212518_at | 3.39 | 0.19 |
| ICAM1 | intercellular adhesion molecule 1 | 202637_s_at | 3.38 | 0.73 |
| KYNU | kynureninase (L-kynurenine hydrolase) | 210663_s_at | 3.38 | 2.45 |
| KDM5C | lysine (K)-specific demethylase 5C | 202383_at | 3.38 | 0.08 |
| ABCA2 | ATP-binding cassette, sub-family A (ABC1), member 2 | 212772_s_at | 3.38 | 0.08 |
| MAOA | monoamine oxidase A | 204388_s_at | 3.37 | 0.86 |
| FBLIM1 | filamin binding LIM protein 1 | 1555480_a_at | 3.37 | 0.23 |
| TLE4 | transducin-like enhancer of split 4 (E(sp1) homolog, Drosophila) | 216997_x_at | 3.37 | 0.33 |
| C14orf34 | chromosome 14 open reading frame 34 | 1555786_s_at | 3.37 | 1.00 |
| XAB2 | XPA binding protein 2 | 218110_at | 3.37 | 0.08 |
| AQP1 | aquaporin 1 (Colton blood group) | 207542_s_at | 3.36 | 0.44 |
| C11orf72 | chromosome 11 open reading frame 72 | 1553438_at | 3.36 | 1.00 |
| LOC100510546 /// STX5 | syntaxin-5-like /// syntaxin 5 | 203330_s_at | 3.36 | 0.00 |
| PLXNB3 | plexin B3 | 205957_at | 3.36 | 0.44 |
| KCNE4 | potassium voltage-gated channel, Isk-related family, member 4 | 1552507_at | 3.36 | 0.23 |
| SLC4A2 | solute carrier family 4, anion exchanger, member 2 (erythrocyte membrane protein band 3-like 1) | 202111_at | 3.36 | 0.33 |
| WWOX | WW domain containing oxidoreductase | 223868_s_at | 3.35 | 2.72 |
| C5orf53 | chromosome 5 open reading frame 53 | 241874_at | 3.35 | 0.14 |
| TBL1X | transducin (beta)-like 1X-linked | 201868_s_at | 3.35 | 0.08 |
| ITCH | Itchy E3 ubiquitin protein ligase homolog (mouse) | 236235_at | 3.35 | 1.25 |
| INO80E | INO80 complex subunit E | 227286_at | 3.35 | 0.00 |
| NFS1 | NFS1 nitrogen fixation 1 homolog (S. cerevisiae) | 1554321_a_at | 3.35 | 0.33 |
| GRK6 | G protein-coupled receptor kinase 6 | 211543_s_at | 3.34 | 2.45 |
| RAB3D | RAB3D, member RAS oncogene family | 225001_at | 3.34 | 0.63 |
| MARCH8 | membrane-associated ring finger (C3HC4) 8 | 231933_at | 3.34 | 0.73 |
| C6orf136 | chromosome 6 open reading frame 136 | 227455_at | 3.34 | 0.26 |
| WISP2 | WNT1 inducible signaling pathway protein 2 | 205792_at | 3.33 | 0.86 |
| PCIF1 | PDX1 C-terminal inhibiting factor 1 | 221762_s_at | 3.33 | 0.14 |
| HMGA2 | high mobility group AT-hook 2 | 1558682_at | 3.33 | 0.63 |
| RPP25 | ribonuclease P/MRP 25kDa subunit | 219143_s_at | 3.33 | 0.33 |
| MRPL4 | mitochondrial ribosomal protein L4 | 223743_s_at | 3.33 | 0.08 |
| KDM6A | lysine (K)-specific demethylase 6A | 203990_s_at | 3.33 | 0.14 |
| PRKAR1A | protein kinase, cAMP-dependent, regulatory, type I, alpha (tissue specific extinguisher 1) | 200604_s_at | 3.33 | 0.63 |
| IL1R1 | interleukin 1 receptor, type I | 215561_s_at | 3.32 | 0.23 |
| BAZ2A | bromodomain adjacent to zinc finger domain, 2A | 201353_s_at | 3.32 | 0.08 |
| GTF3C2 | general transcription factor IIIC, polypeptide 2, beta 110kDa | 210620_s_at | 3.32 | 2.72 |
| MMP14 | matrix metallopeptidase 14 (membrane-inserted) | 217279_x_at | 3.31 | 0.14 |
| C9orf16 | chromosome 9 open reading frame 16 | 41047_at | 3.31 | 0.00 |
| MAN1A1 | mannosidase, alpha, class 1A, member 1 | 208116_s_at | 3.31 | 1.25 |
| OR7E104P | olfactory receptor, family 7, subfamily E, member 104 pseudogene | 1566956_at | 3.31 | 0.35 |
| BAP1 | BRCA1 associated protein-1 (ubiquitin carboxy-terminal hydrolase) | 1555735_a_at | 3.30 | 0.08 |
| FKBP1A | FK506 binding protein 1A, 12kDa | 214119_s_at | 3.30 | 0.35 |
| LOC100132288 /// MAFIP | hypothetical protein LOC100132288 /// MAFF interacting protein | 227715_at | 3.30 | 0.73 |
| WAC | WW domain containing adaptor with coiled-coil | 219679_s_at | 3.30 | 0.23 |
| CADM1 | cell adhesion molecule 1 | 209030_s_at | 3.30 | 0.23 |
| CARS | cysteinyl-tRNA synthetase | 240983_s_at | 3.30 | 0.08 |
| BHLHE41 | basic helix-loop-helix family, member e41 | 231243_s_at | 3.29 | 1.00 |
| AK5 | adenylate kinase 5 | 222862_s_at | 3.29 | 0.26 |
| TMEM54 | transmembrane protein 54 | 225536_at | 3.29 | 0.08 |
| NAMPT | nicotinamide phosphoribosyltransferase | 1555167_s_at | 3.29 | 0.26 |
| VIPAR | VPS33B interacting protein, apical-basolateral polarity regulator | 233140_s_at | 3.29 | 0.23 |
| LZTS2 | leucine zipper, putative tumor suppressor 2 | 232129_s_at | 3.29 | 0.00 |
| CPD | carboxypeptidase D | 201942_s_at | 3.29 | 0.35 |
| PTPN11 | protein tyrosine phosphatase, non-receptor type 11 | 209895_at | 3.29 | 0.19 |
| CDC42 | cell division cycle 42 (GTP binding protein, 25kDa) | 208727_s_at | 3.28 | 1.67 |
| RGNEF | 190 kDa guanine nucleotide exchange factor | 1560348_at | 3.28 | 0.19 |
| PTGFR | prostaglandin F receptor (FP) | 1555097_a_at | 3.28 | 1.67 |
| DDX3X | DEAD (Asp-Glu-Ala-Asp) box polypeptide 3, X-linked | 212514_x_at | 3.28 | 0.26 |
| GOLGA6L4 /// PML | golgin A6 family-like 4 /// promyelocytic leukemia | 211012_s_at | 3.28 | 0.19 |
| NFX1 | nuclear transcription factor, X-box binding 1 | 1553348_a_at | 3.27 | 0.19 |
| TXNIP | thioredoxin interacting protein | 201008_s_at | 3.27 | 0.26 |
| CD44 | CD44 molecule (Indian blood group) | 210916_s_at | 3.27 | 0.08 |
| UFM1 | ubiquitin-fold modifier 1 | 222502_s_at | 3.27 | 0.33 |
| TOM1 | target of myb1 (chicken) | 202807_s_at | 3.27 | 0.08 |
| DDR2 | discoidin domain receptor tyrosine kinase 2 | 205168_at | 3.26 | 0.00 |
| SCAMP4 | secretory carrier membrane protein 4 | 213244_at | 3.26 | 0.08 |
| DOK1 | docking protein 1, 62kDa (downstream of tyrosine kinase 1) | 211121_s_at | 3.26 | 0.14 |
| CACNA1D | calcium channel, voltage-dependent, L type, alpha 1D subunit | 1555993_at | 3.26 | 1.67 |
| TBC1D5 | TBC1 domain family, member 5 | 201815_s_at | 3.25 | 0.23 |
| MBD1 | methyl-CpG binding domain protein 1 | 208595_s_at | 3.25 | 0.08 |
| ZNF229 | zinc finger protein 229 | 1562789_at | 3.25 | 0.73 |
| MDK | midkine (neurite growth-promoting factor 2) | 209035_at | 3.25 | 0.55 |
| SSC5D | scavenger receptor cysteine-rich glycoprotein | 230228_at | 3.25 | 0.73 |
| CLEC11A | C-type lectin domain family 11, member A | 210783_x_at | 3.24 | 0.08 |
| NRP2 | neuropilin 2 | 223510_at | 3.24 | 0.44 |
| RHBDF2 | rhomboid 5 homolog 2 (Drosophila) | 219202_at | 3.24 | 0.35 |
| NT5DC2 | 5'-nucleotidase domain containing 2 | 218051_s_at | 3.24 | 0.23 |
| PRUNE | prune homolog (Drosophila) | 210988_s_at | 3.24 | 0.19 |
| APOBEC3C | apolipoprotein B mRNA editing enzyme, catalytic polypeptide-like 3C | 209584_x_at | 3.24 | 0.00 |
| TOX4 | TOX high mobility group box family member 4 | 217448_s_at | 3.24 | 0.08 |
| ITGB2 | integrin, beta 2 (complement component 3 receptor 3 and 4 subunit) | 202803_s_at | 3.24 | 0.14 |
| CD46 | CD46 molecule, complement regulatory protein | 211574_s_at | 3.24 | 0.14 |
| PAK2 | p21 protein (Cdc42/Rac)-activated kinase 2 | 208875_s_at | 3.23 | 0.08 |
| PPARD | peroxisome proliferator-activated receptor delta | 208044_s_at | 3.23 | 0.08 |
| SMARCA2 | SWI/SNF related, matrix associated, actin dependent regulator of chromatin, subfamily a, member 2 | 212257_s_at | 3.23 | 0.14 |
| HECTD3 | HECT domain containing 3 | 218632_at | 3.23 | 0.00 |
| C9orf41 | chromosome 9 open reading frame 41 | 241781_at | 3.23 | 0.23 |
| PPARA | peroxisome proliferator-activated receptor alpha | 1558631_at | 3.23 | 0.14 |
| PHF6 | PHD finger protein 6 | 224442_at | 3.23 | 1.67 |
| STARD10 | StAR-related lipid transfer (START) domain containing 10 | 223103_at | 3.22 | 0.23 |
| SLC35F5 | solute carrier family 35, member F5 | 240335_at | 3.22 | 0.73 |
| PAPPA | pregnancy-associated plasma protein A, pappalysin 1 | 201982_s_at | 3.22 | 0.44 |
| UNC45A | unc-45 homolog A (C. elegans) | 207499_x_at | 3.21 | 0.19 |
| SCARB1 | scavenger receptor class B, member 1 | 1552256_a_at | 3.21 | 0.23 |
| PSEN1 | presenilin 1 | 207782_s_at | 3.21 | 0.08 |
| OBSL1 | obscurin-like 1 | 227573_s_at | 3.21 | 0.63 |
| PPFIBP1 | PTPRF interacting protein, binding protein 1 (liprin beta 1) | 203736_s_at | 3.21 | 0.19 |
| TAOK2 | TAO kinase 2 | 204986_s_at | 3.21 | 0.23 |
| PHLDB3 | pleckstrin homology-like domain, family B, member 3 | 1557948_at | 3.21 | 0.35 |
| BARX1 | BARX homeobox 1 | 219845_at | 3.20 | 1.67 |
| NONO | non-POU domain containing, octamer-binding | 208698_s_at | 3.19 | 0.19 |
| GSTM1 | glutathione S-transferase mu 1 | 215333_x_at | 3.19 | 0.35 |
| GFM2 | G elongation factor, mitochondrial 2 | 231918_s_at | 3.19 | 0.44 |
| FAM129B | family with sequence similarity 129, member B | 223019_at | 3.19 | 0.19 |
| RHBDD2 | rhomboid domain containing 2 | 232053_x_at | 3.19 | 0.19 |
| CLIC4 | chloride intracellular channel 4 | 201559_s_at | 3.19 | 0.73 |
| CALU | calumenin | 214845_s_at | 3.19 | 0.14 |
| NDUFA10 | NADH dehydrogenase (ubiquinone) 1 alpha subcomplex, 10, 42kDa | 227206_at | 3.19 | 0.44 |
| SPEN | spen homolog, transcriptional regulator (Drosophila) | 1556058_s_at | 3.18 | 0.55 |
| TBX2 | T-box 2 | 205993_s_at | 3.18 | 0.23 |
| ACAP3 | ArfGAP with coiled-coil, ankyrin repeat and PH domains 3 | 225529_at | 3.18 | 0.23 |
| NUP98 | nucleoporin 98kDa | 203194_s_at | 3.18 | 0.14 |
| UEVLD | UEV and lactate/malate dehyrogenase domains | 1554397_s_at | 3.18 | 0.73 |
| LOC100506866 | hypothetical LOC100506866 | 242679_at | 3.18 | 1.44 |
| TTYH3 | tweety homolog 3 (Drosophila) | 224674_at | 3.18 | 0.14 |
| TFDP1 | transcription factor Dp-1 | 204147_s_at | 3.17 | 0.14 |
| ATL2 | atlastin GTPase 2 | 237968_at | 3.17 | 0.33 |
| PDE5A | phosphodiesterase 5A, cGMP-specific | 1562228_s_at | 3.17 | 0.73 |
| KIAA0467 | KIAA0467 | 203900_at | 3.17 | 0.00 |
| ABCC4 | ATP-binding cassette, sub-family C (CFTR/MRP), member 4 | 1555039_a_at | 3.17 | 0.73 |
| ITGA5 | integrin, alpha 5 (fibronectin receptor, alpha polypeptide) | 201389_at | 3.17 | 0.19 |
| CIZ1 | CDKN1A interacting zinc finger protein 1 | 205516_x_at | 3.16 | 0.63 |
| ASAM | adipocyte-specific adhesion molecule | 228082_at | 3.16 | 0.00 |
| IRF3 | interferon regulatory factor 3 | 202621_at | 3.16 | 0.00 |
| PLSCR3 | phospholipid scramblase 3 | 218828_at | 3.16 | 0.23 |
| POM121 | POM121 membrane glycoprotein | 205096_at | 3.16 | 0.35 |
| FAHD2A /// LOC285014 | fumarylacetoacetate hydrolase domain containing 2A /// hypothetical protein LOC285014 | 235621_at | 3.16 | 0.35 |
| PI4KB | phosphatidylinositol 4-kinase, catalytic, beta | 210417_s_at | 3.16 | 0.00 |
| G6PD | glucose-6-phosphate dehydrogenase | 202275_at | 3.16 | 0.35 |
| SF3A1 | splicing factor 3a, subunit 1, 120kDa | 201357_s_at | 3.16 | 0.08 |
| RASA2 | RAS p21 protein activator 2 | 206636_at | 3.16 | 0.19 |
| TMEM132A | transmembrane protein 132A | 222758_s_at | 3.15 | 1.00 |
| AGFG1 | ArfGAP with FG repeats 1 | 213926_s_at | 3.15 | 0.55 |
| GATAD2B | GATA zinc finger domain containing 2B | 225393_at | 3.15 | 0.14 |
| PPP2R4 | protein phosphatase 2A activator, regulatory subunit 4 | 208874_x_at | 3.15 | 0.00 |
| MDM4 | Mdm4 p53 binding protein homolog (mouse) | 235589_s_at | 3.15 | 0.14 |
| AP2M1 | adaptor-related protein complex 2, mu 1 subunit | 200613_at | 3.15 | 0.00 |
| AKT3 | v-akt murine thymoma viral oncogene homolog 3 (protein kinase B, gamma) | 222880_at | 3.15 | 0.23 |
| TTC3 | tetratricopeptide repeat domain 3 | 208664_s_at | 3.15 | 0.26 |
| CDK17 | cyclin-dependent kinase 17 | 206474_at | 3.15 | 0.86 |
| COL8A1 | collagen, type VIII, alpha 1 | 214587_at | 3.14 | 0.73 |
| TBC1D17 | TBC1 domain family, member 17 | 218466_at | 3.14 | 0.00 |
| FOXP4 | forkhead box P4 | 227120_at | 3.14 | 0.23 |
| GBA2 | glucosidase, beta (bile acid) 2 | 223921_s_at | 3.14 | 0.08 |
| CCDC124 | coiled-coil domain containing 124 | 225454_at | 3.13 | 0.08 |
| MIB2 | mindbomb homolog 2 (Drosophila) | 228261_at | 3.13 | 0.35 |
| VEGFA | vascular endothelial growth factor A | 212171_x_at | 3.13 | 0.55 |
| IGF2 /// INS-IGF2 | insulin-like growth factor 2 (somatomedin A) /// INS-IGF2 readthrough transcript | 202409_at | 3.13 | 1.00 |
| CNST | consortin, connexin sorting protein | 1554660_a_at | 3.13 | 0.63 |
| FURIN | furin (paired basic amino acid cleaving enzyme) | 201945_at | 3.13 | 0.73 |
| ASMTL | acetylserotonin O-methyltransferase-like | 209394_at | 3.12 | 0.08 |
| ALG3 | asparagine-linked glycosylation 3, alpha-1,3- mannosyltransferase homolog (S. cerevisiae) | 207396_s_at | 3.12 | 0.08 |
| MALAT1 | metastasis associated lung adenocarcinoma transcript 1 (non-protein coding) | 227510_x_at | 3.12 | 1.25 |
| CLEC11A | C-type lectin domain family 11, member A | 205131_x_at | 3.12 | 0.19 |
| TOB1 | transducer of ERBB2, 1 | 228834_at | 3.12 | 0.19 |
| DMWD | dystrophia myotonica, WD repeat containing | 33768_at | 3.11 | 0.23 |
| LGALS3BP | lectin, galactoside-binding, soluble, 3 binding protein | 200923_at | 3.11 | 0.14 |
| PEX5 | peroxisomal biogenesis factor 5 | 215481_s_at | 3.11 | 0.19 |
| EPB41 | erythrocyte membrane protein band 4.1 (elliptocytosis 1, RH-linked) | 207793_s_at | 3.11 | 0.73 |
| OTUD5 | OTU domain containing 5 | 233933_s_at | 3.10 | 0.00 |
| PTMS | parathymosin | 218045_x_at | 3.10 | 0.14 |
| PPARA | peroxisome proliferator-activated receptor alpha | 206870_at | 3.10 | 0.86 |
| MSC | musculin | 209928_s_at | 3.10 | 0.23 |
| KLF12 | Kruppel-like factor 12 | 206966_s_at | 3.10 | 0.08 |
| MFGE8 | milk fat globule-EGF factor 8 protein | 210605_s_at | 3.09 | 0.73 |
| PRPS2 | Phosphoribosyl pyrophosphate synthetase 2 | 230352_at | 3.09 | 0.33 |
| PCDHGA1 /// PCDHGA10 /// PCDHGA11 /// PCDHGA12 /// PCDHGA2 /// PCDHGA3 /// PCDHGA4 /// PCDHGA5 /// PCDHGA6 /// PCDHGA7 /// PCDHGA8 /// PCDHGA9 /// PCDHGB1 /// PCDHGB2 /// PCDHGB3 /// PCDHGB4 /// PCDHGB5 /// PCDHGB6 /// PCDHGB7 /// PCDHGC3 /// PCDHGC4 /// PCDHGC5 | protocadherin gamma subfamily A, 1 /// protocadherin gamma subfamily A, 10 /// protocadherin gamma subfamily A, 11 /// protocadherin gamma subfamily A, 12 /// protocadherin gamma subfamily A, 2 /// protocadherin gamma subfamily A, 3 /// protocadherin gamma subfamily A, 4 /// protocadherin gamma subfamily A, 5 /// protocadherin gamma subfamily A, 6 /// protocadherin gamma subfamily A, 7 /// protocadherin gamma subfamily A, 8 /// protocadherin gamma subfamily A, 9 /// protocadherin gamma subfamily B, 1 /// protocadherin gamma subfamily B, 2 /// protocadherin gamma subfamily B, 3 /// protocadherin gamma subfamily B, 4 /// protocadherin gamma subfamily B, 5 /// protocadherin gamma subfamily B, 6 /// protocadherin gamma subfamily B, 7 /// protocadherin gamma subfamily C, 3 /// protocadherin gamma subfamily C, 4 /// protocadherin gamma subfamily C, 5 | 211066_x_at | 3.09 | 0.00 |
| UBXN6 | UBX domain protein 6 | 220757_s_at | 3.09 | 0.00 |
| PTH1R | parathyroid hormone 1 receptor | 205911_at | 3.09 | 1.25 |
| KRTAP1-3 | keratin associated protein 1-3 | 234880_x_at | 3.08 | 1.25 |
| PSD4 | pleckstrin and Sec7 domain containing 4 | 203317_at | 3.08 | 0.33 |
| WWP2 | WW domain containing E3 ubiquitin protein ligase 2 | 210200_at | 3.08 | 0.26 |
| MASP2 | mannan-binding lectin serine peptidase 2 | 207041_at | 3.08 | 1.67 |
| TMEM115 | transmembrane protein 115 | 216267_s_at | 3.08 | 0.14 |
| HLA-DRB1 /// HLA-DRB3 /// HLA-DRB4 | major histocompatibility complex, class II, DR beta 1 /// major histocompatibility complex, class II, DR beta 3 /// major histocompatibility complex, class II, DR beta 4 | 215193_x_at | 3.08 | 3.67 |
| MEF2D | myocyte enhancer factor 2D | 203003_at | 3.08 | 0.23 |
| RALGPS2 | Ral GEF with PH domain and SH3 binding motif 2 | 220338_at | 3.08 | 1.25 |
| RAB5A | RAB5A, member RAS oncogene family | 206113_s_at | 3.08 | 0.63 |
| SPEM1 | spermatid maturation 1 | 240878_at | 3.08 | 7.84 |
| GALT | galactose-1-phosphate uridylyltransferase | 232708_at | 3.07 | 0.73 |
| GPR172A | G protein-coupled receptor 172A | 222155_s_at | 3.07 | 0.35 |
| SYNRG | synergin, gamma | 1553165_at | 3.07 | 1.25 |
| ARHGDIA | Rho GDP dissociation inhibitor (GDI) alpha | 211716_x_at | 3.07 | 0.00 |
| PTPRD | protein tyrosine phosphatase, receptor type, D | 213362_at | 3.07 | 3.21 |
| C6orf1 | chromosome 6 open reading frame 1 | 226306_at | 3.07 | 0.23 |
| TADA3 | transcriptional adaptor 3 | 215273_s_at | 3.07 | 0.08 |
| CUL7 | cullin 7 | 36084_at | 3.07 | 0.08 |
| CSNK2A1 | casein kinase 2, alpha 1 polypeptide | 212075_s_at | 3.07 | 0.23 |
| CALM3 | calmodulin 3 (phosphorylase kinase, delta) | 200623_s_at | 3.06 | 0.19 |
| NDE1 | nudE nuclear distribution gene E homolog 1 (A. nidulans) | 222625_s_at | 3.06 | 0.19 |
| FGF5 | fibroblast growth factor 5 | 210311_at | 3.06 | 0.44 |
| CST3 | cystatin C | 201360_at | 3.06 | 0.08 |
| WWP2 | WW domain containing E3 ubiquitin protein ligase 2 | 1552737_s_at | 3.06 | 2.06 |
| CDK14 | Cyclin-dependent kinase 14 | 243459_x_at | 3.06 | 0.26 |
| NID1 | nidogen 1 | 202008_s_at | 3.05 | 0.44 |
| SUMO3 | SMT3 suppressor of mif two 3 homolog 3 (S. cerevisiae) | 200739_s_at | 3.05 | 0.14 |
| EML3 | echinoderm microtubule associated protein like 3 | 212969_x_at | 3.05 | 0.23 |
| PI4K2A | phosphatidylinositol 4-kinase type 2 alpha | 209346_s_at | 3.05 | 0.55 |
| GMDS | GDP-mannose 4,6-dehydratase | 204875_s_at | 3.05 | 0.35 |
| LAMP1 | lysosomal-associated membrane protein 1 | 201551_s_at | 3.05 | 0.08 |
| SCARB2 | scavenger receptor class B, member 2 | 201646_at | 3.04 | 0.44 |
| ITGA3 | integrin, alpha 3 (antigen CD49C, alpha 3 subunit of VLA-3 receptor) | 201474_s_at | 3.04 | 1.00 |
| LMLN | leishmanolysin-like (metallopeptidase M8 family) | 1553284_s_at | 3.04 | 0.44 |
| FOSL2 | FOS-like antigen 2 | 218880_at | 3.04 | 0.33 |
| MYO1D | myosin ID | 212338_at | 3.04 | 0.14 |
| ENTPD4 | ectonucleoside triphosphate diphosphohydrolase 4 | 1555358_a_at | 3.03 | 0.73 |
| EXD2 | exonuclease 3'-5' domain containing 2 | 1555808_a_at | 3.03 | 1.25 |
| HIST3H2A | histone cluster 3, H2a | 221582_at | 3.03 | 0.73 |
| LMOD1 | leiomodin 1 (smooth muscle) | 211562_s_at | 3.03 | 0.33 |
| NOTCH2 | notch 2 | 210756_s_at | 3.03 | 0.26 |
| FAM86B1 | family with sequence similarity 86, member B1 | 65585_at | 3.03 | 0.19 |
| DSTYK | dual serine/threonine and tyrosine protein kinase | 211515_s_at | 3.03 | 0.44 |
| SLC39A9 | solute carrier family 39 (zinc transporter), member 9 | 217859_s_at | 3.02 | 1.25 |
| MIA3 | melanoma inhibitory activity family, member 3 | 1569057_s_at | 3.02 | 0.14 |
| LMF1 | lipase maturation factor 1 | 219135_s_at | 3.02 | 0.44 |
| SAMD1 | sterile alpha motif domain containing 1 | 225650_at | 3.02 | 0.23 |
| PEX14 | peroxisomal biogenesis factor 14 | 203503_s_at | 3.02 | 0.26 |
| DDX3X | DEAD (Asp-Glu-Ala-Asp) box polypeptide 3, X-linked | 201211_s_at | 3.02 | 0.26 |
| UTRN | utrophin | 213023_at | 3.02 | 0.14 |
| TAF9B | TAF9B RNA polymerase II, TATA box binding protein (TBP)-associated factor, 31kDa | 221618_s_at | 3.02 | 4.15 |
| OS9 | osteosarcoma amplified 9, endoplasmic reticulum lectin | 215399_s_at | 3.01 | 0.08 |
| EMP3 | epithelial membrane protein 3 | 203729_at | 3.01 | 0.73 |
| RRM1 | ribonucleotide reductase M1 | 201476_s_at | 3.01 | 0.23 |
| BCL6 | B-cell CLL/lymphoma 6 | 215990_s_at | 3.01 | 0.26 |
| ZDHHC5 | zinc finger, DHHC-type containing 5 | 224868_at | 3.01 | 0.33 |
| SLC2A4RG | SLC2A4 regulator | 218494_s_at | 3.01 | 0.14 |
| AKT1 | v-akt murine thymoma viral oncogene homolog 1 | 207163_s_at | 3.00 | 0.08 |
| FKBP7 | FK506 binding protein 7 | 223667_at | 3.00 | 0.55 |
| TPM4 | tropomyosin 4 | 212481_s_at | 3.00 | 0.23 |
| STRN4 | striatin, calmodulin binding protein 4 | 217903_at | 3.00 | 0.14 |
| PATL1 | protein associated with topoisomerase II homolog 1 (yeast) | 235234_at | 2.99 | 0.14 |
| ITGB1 | integrin, beta 1 (fibronectin receptor, beta polypeptide, antigen CD29 includes MDF2, MSK12) | 216178_x_at | 2.99 | 0.86 |
| STAT2 | signal transducer and activator of transcription 2, 113kDa | 205170_at | 2.99 | 0.63 |
| DCTN1 | dynactin 1 | 211780_x_at | 2.99 | 0.26 |
| MAPK1IP1L | mitogen-activated protein kinase 1 interacting protein 1-like | 212497_at | 2.99 | 0.86 |
| GSPT1 | G1 to S phase transition 1 | 234975_at | 2.99 | 0.26 |
| TTLL3 | tubulin tyrosine ligase-like family, member 3 | 230872_s_at | 2.99 | 0.73 |
| AGTR1 | angiotensin II receptor, type 1 | 208016_s_at | 2.99 | 2.06 |
| TM9SF4 | transmembrane 9 superfamily protein member 4 | 212194_s_at | 2.98 | 0.08 |
| GBF1 | golgi brefeldin A resistant guanine nucleotide exchange factor 1 | 201439_at | 2.98 | 0.00 |
| RPS2 | ribosomal protein S2 | 217466_x_at | 2.98 | 2.45 |
| JAK1 | Janus kinase 1 | 1552610_a_at | 2.98 | 0.33 |
| KLF16 | Kruppel-like factor 16 | 226328_at | 2.98 | 0.33 |
| OBSL1 | obscurin-like 1 | 212776_s_at | 2.98 | 0.23 |
| ASAH2B | N-acylsphingosine amidohydrolase (non-lysosomal ceramidase) 2B | 231791_at | 2.98 | 1.00 |
| SYNPO2 | synaptopodin 2 | 232119_at | 2.97 | 1.00 |
| KIAA1109 | KIAA1109 | 216294_s_at | 2.97 | 0.26 |
| ERCC2 | excision repair cross-complementing rodent repair deficiency, complementation group 2 | 213468_at | 2.97 | 0.08 |
| CTSD | cathepsin D | 200766_at | 2.97 | 0.44 |
| NADK | NAD kinase | 213607_x_at | 2.97 | 0.14 |
| PHTF2 | putative homeodomain transcription factor 2 | 1554780_a_at | 2.97 | 0.23 |
| NADK | NAD kinase | 208917_x_at | 2.97 | 0.55 |
| SLC9A3R1 | solute carrier family 9 (sodium/hydrogen exchanger), member 3 regulator 1 | 201349_at | 2.97 | 0.55 |
| DAB2IP | DAB2 interacting protein | 225020_at | 2.97 | 0.08 |
| GLE1 | GLE1 RNA export mediator homolog (yeast) | 206920_s_at | 2.97 | 0.23 |
| MAPKAP1 | mitogen-activated protein kinase associated protein 1 | 229846_s_at | 2.96 | 0.35 |
| KBTBD2 | kelch repeat and BTB (POZ) domain containing 2 | 223585_x_at | 2.96 | 0.23 |
| LARP4 | La ribonucleoprotein domain family, member 4 | 1555384_a_at | 2.95 | 0.14 |
| RPAP3 | RNA polymerase II associated protein 3 | 1557984_s_at | 2.95 | 0.73 |
| E2F1 | E2F transcription factor 1 | 2028_s_at | 2.95 | 0.26 |
| PHLDB2 | pleckstrin homology-like domain, family B, member 2 | 238419_at | 2.95 | 0.55 |
| HSF2 | heat shock transcription factor 2 | 211220_s_at | 2.95 | 0.86 |
| TRUB1 | TruB pseudouridine (psi) synthase homolog 1 (E. coli) | 241606_s_at | 2.95 | 0.23 |
| C22orf30 | chromosome 22 open reading frame 30 | 216555_at | 2.95 | 0.86 |
| SAR1A | SAR1 homolog A (S. cerevisiae) | 210790_s_at | 2.95 | 1.44 |
| SGCB | sarcoglycan, beta (43kDa dystrophin-associated glycoprotein) | 205121_at | 2.95 | 0.86 |
| EGR1 | Early growth response 1 | 227404_s_at | 2.95 | 1.25 |
| ZXDA /// ZXDB | zinc finger, X-linked, duplicated A /// zinc finger, X-linked, duplicated B | 215263_at | 2.94 | 0.63 |
| ADAM9 | ADAM metallopeptidase domain 9 | 1555326_a_at | 2.94 | 0.86 |
| NCRNA00204B | Non-protein coding RNA 204B | 244164_at | 2.94 | 0.55 |
| POMGNT1 | protein O-linked mannose beta1,2-N-acetylglucosaminyltransferase | 233638_s_at | 2.94 | 0.86 |
| ZNF438 | zinc finger protein 438 | 244752_at | 2.94 | 0.86 |
| NR1D2 | nuclear receptor subfamily 1, group D, member 2 | 209750_at | 2.94 | 0.44 |
| SOCS3 | suppressor of cytokine signaling 3 | 206359_at | 2.93 | 0.73 |
| PTGDS | prostaglandin D2 synthase 21kDa (brain) | 211663_x_at | 2.93 | 1.25 |
| PLAC8L1 | PLAC8-like 1 | 237783_at | 2.93 | 4.15 |
| VAMP3 | vesicle-associated membrane protein 3 (cellubrevin) | 201337_s_at | 2.93 | 0.63 |
| RCN3 | reticulocalbin 3, EF-hand calcium binding domain | 219102_at | 2.93 | 0.19 |
| GAMT | guanidinoacetate N-methyltransferase | 205354_at | 2.93 | 0.14 |
| MYO9B | myosin IXB | 217297_s_at | 2.93 | 0.26 |
| MYO15A | myosin XVA | 220288_at | 2.93 | 1.25 |
| PLCD1 | phospholipase C, delta 1 | 205125_at | 2.93 | 0.55 |
| LOC100128988 | hypothetical LOC100128988 | 232849_at | 2.93 | 2.72 |
| HFE | hemochromatosis | 210864_x_at | 2.93 | 0.86 |
| POU2F2 | POU class 2 homeobox 2 | 228343_at | 2.92 | 2.72 |
| FOXJ3 | forkhead box J3 | 217310_s_at | 2.92 | 0.33 |
| ZBTB22 | zinc finger and BTB domain containing 22 | 213081_at | 2.92 | 0.08 |
| SLC1A4 | solute carrier family 1 (glutamate/neutral amino acid transporter), member 4 | 209611_s_at | 2.92 | 0.35 |
| BCL2L11 | BCL2-like 11 (apoptosis facilitator) | 1558143_a_at | 2.92 | 1.44 |
| FXYD5 | FXYD domain containing ion transport regulator 5 | 218084_x_at | 2.92 | 0.14 |
| HLA-DRB1 /// HLA-DRB4 | major histocompatibility complex, class II, DR beta 1 /// major histocompatibility complex, class II, DR beta 4 | 204670_x_at | 2.91 | 3.67 |
| BAP1 | BRCA1 associated protein-1 (ubiquitin carboxy-terminal hydrolase) | 201419_at | 2.91 | 0.23 |
| TP53 | tumor protein p53 | 201746_at | 2.91 | 0.35 |
| SYDE1 | synapse defective 1, Rho GTPase, homolog 1 (C. elegans) | 216271_x_at | 2.91 | 0.55 |
| RPL37 | ribosomal protein L37 | 224767_at | 2.91 | 1.67 |
| PTPRD | protein tyrosine phosphatase, receptor type, D | 205712_at | 2.91 | 1.67 |
| CPZ /// GPR78 | carboxypeptidase Z /// G protein-coupled receptor 78 | 211062_s_at | 2.91 | 0.86 |
| SLC16A6 | solute carrier family 16, member 6 (monocarboxylic acid transporter 7) | 207038_at | 2.91 | 2.06 |
| INO80B | INO80 complex subunit B | 222093_s_at | 2.91 | 0.26 |
| RPRD2 | regulation of nuclear pre-mRNA domain containing 2 | 212553_at | 2.91 | 0.35 |
| UBE4B | ubiquitination factor E4B (UFD2 homolog, yeast) | 215533_s_at | 2.90 | 0.08 |
| FCHSD1 | FCH and double SH3 domains 1 | 226699_at | 2.90 | 1.25 |
| ANGPT1 | angiopoietin 1 | 205608_s_at | 2.90 | 0.55 |
| KCTD13 | potassium channel tetramerisation domain containing 13 | 45653_at | 2.90 | 0.14 |
| STEAP3 | STEAP family member 3 | 1554830_a_at | 2.90 | 0.44 |
| B3GAT3 | beta-1,3-glucuronyltransferase 3 (glucuronosyltransferase I) | 203452_at | 2.90 | 0.35 |
| PLCE1 | Phospholipase C, epsilon 1 | 1566739_at | 2.89 | 0.55 |
| ARF3 | ADP-ribosylation factor 3 | 211622_s_at | 2.89 | 0.35 |
| TBX18 | T-box 18 | 1559840_s_at | 2.89 | 0.14 |
| PINK1 | PTEN induced putative kinase 1 | 209018_s_at | 2.89 | 0.19 |
| MED20 | mediator complex subunit 20 | 206961_s_at | 2.89 | 0.86 |
| NEGR1 | neuronal growth regulator 1 | 1553194_at | 2.89 | 0.73 |
| HLA-A /// HLA-F /// HLA-J | major histocompatibility complex, class I, A /// major histocompatibility complex, class I, F /// major histocompatibility complex, class I, J (pseudogene) | 217436_x_at | 2.89 | 0.33 |
| SLC43A3 | solute carrier family 43, member 3 | 210692_s_at | 2.89 | 0.19 |
| LHPP | phospholysine phosphohistidine inorganic pyrophosphate phosphatase | 218523_at | 2.89 | 0.14 |
| HDAC10 | histone deacetylase 10 | 226672_s_at | 2.89 | 0.14 |
| CHD7 | chromodomain helicase DNA binding protein 7 | 226123_at | 2.89 | 0.44 |
| USP21 | ubiquitin specific peptidase 21 | 234735_s_at | 2.89 | 0.26 |
| GLUL | glutamate-ammonia ligase | 217202_s_at | 2.88 | 0.19 |
| TAPBP | TAP binding protein (tapasin) | 208829_at | 2.88 | 0.26 |
| COL13A1 | collagen, type XIII, alpha 1 | 211809_x_at | 2.88 | 0.35 |
| ALDH1B1 | aldehyde dehydrogenase 1 family, member B1 | 209645_s_at | 2.88 | 2.45 |
| SDC1 | syndecan 1 | 201287_s_at | 2.88 | 0.73 |
| MGC23270 | hypothetical LOC196872 | 1553780_at | 2.88 | 0.86 |
| STYX | serine/threonine/tyrosine interacting protein | 244030_at | 2.88 | 0.44 |
| MAP3K7 | mitogen-activated protein kinase kinase kinase 7 | 211536_x_at | 2.87 | 0.33 |
| DBP | D site of albumin promoter (albumin D-box) binding protein | 209782_s_at | 2.87 | 2.45 |
| C12orf69 | chromosome 12 open reading frame 69 | 237484_at | 2.87 | 2.45 |
| INF2 | inverted formin, FH2 and WH2 domain containing | 222534_s_at | 2.87 | 0.33 |
| NCRNA00094 | non-protein coding RNA 94 | 213788_s_at | 2.87 | 0.19 |
| ETV5 | ets variant 5 | 216375_s_at | 2.87 | 0.73 |
| H2AFY2 | H2A histone family, member Y2 | 218445_at | 2.87 | 1.67 |
| CHD1L | chromodomain helicase DNA binding protein 1-like | 207645_s_at | 2.87 | 0.63 |
| RUFY3 | RUN and FYVE domain containing 3 | 229334_at | 2.87 | 0.33 |
| CABIN1 | calcineurin binding protein 1 | 202624_s_at | 2.87 | 0.55 |
| SAE1 | SUMO1 activating enzyme subunit 1 | 1555618_s_at | 2.87 | 0.55 |
| IFT140 | intraflagellar transport 140 homolog (Chlamydomonas) | 204792_s_at | 2.87 | 0.19 |
| GART | phosphoribosylglycinamide formyltransferase, phosphoribosylglycinamide synthetase, phosphoribosylaminoimidazole synthetase | 212379_at | 2.86 | 3.67 |
| SEPT1 | septin 1 | 227552_at | 2.86 | 1.00 |
| FAM108B1 | family with sequence similarity 108, member B1 | 220285_at | 2.86 | 1.67 |
| TRANK1 | tetratricopeptide repeat and ankyrin repeat containing 1 | 213261_at | 2.86 | 0.19 |
| FZD1 | frizzled homolog 1 (Drosophila) | 204452_s_at | 2.85 | 0.35 |
| UBE3C | ubiquitin protein ligase E3C | 1554794_a_at | 2.85 | 1.00 |
| PRIC285 | peroxisomal proliferator-activated receptor A interacting complex 285 | 228230_at | 2.85 | 1.25 |
| C16orf13 | chromosome 16 open reading frame 13 | 228114_x_at | 2.85 | 0.63 |
| NXPH3 | neurexophilin 3 | 228210_at | 2.85 | 0.73 |
| CC2D1A | coiled-coil and C2 domain containing 1A | 58994_at | 2.84 | 0.14 |
| WFS1 | Wolfram syndrome 1 (wolframin) | 1555270_a_at | 2.84 | 0.14 |
| HOXC10 | homeobox C10 | 218959_at | 2.84 | 0.44 |
| IDUA | iduronidase, alpha-L- | 205059_s_at | 2.84 | 0.19 |
| MAP1LC3A | microtubule-associated protein 1 light chain 3 alpha | 232011_s_at | 2.84 | 0.23 |
| MGEA5 | Meningioma expressed antigen 5 (hyaluronidase) | 214972_at | 2.84 | 2.06 |
| L3MBTL2 | l(3)mbt-like 2 (Drosophila) | 1555815_a_at | 2.84 | 0.33 |
| PTPN21 | protein tyrosine phosphatase, non-receptor type 21 | 1320_at | 2.84 | 0.35 |
| DDX17 | DEAD (Asp-Glu-Ala-Asp) box polypeptide 17 | 208151_x_at | 2.84 | 0.33 |
| MTSS1 | metastasis suppressor 1 | 210360_s_at | 2.84 | 0.86 |
| NME4 | non-metastatic cells 4, protein expressed in | 212739_s_at | 2.84 | 0.14 |
| DAK | dihydroxyacetone kinase 2 homolog (S. cerevisiae) | 218688_at | 2.83 | 3.21 |
| STARD10 | StAR-related lipid transfer (START) domain containing 10 | 232322_x_at | 2.83 | 0.55 |
| KCTD20 | potassium channel tetramerisation domain containing 20 | 214849_at | 2.83 | 0.86 |
| TLE3 | transducin-like enhancer of split 3 (E(sp1) homolog, Drosophila) | 206472_s_at | 2.83 | 0.55 |
| C13orf31 | chromosome 13 open reading frame 31 | 1553141_at | 2.83 | 2.06 |
| TMEM132D | transmembrane protein 132D | 236377_at | 2.83 | 1.25 |
| GPX3 | glutathione peroxidase 3 (plasma) | 201348_at | 2.83 | 0.63 |
| OTUD5 | OTU domain containing 5 | 224745_x_at | 2.82 | 0.14 |
| FMO2 | flavin containing monooxygenase 2 (non-functional) | 211726_s_at | 2.82 | 0.44 |
| KIAA1462 | KIAA1462 | 231841_s_at | 2.82 | 0.55 |
| CRELD1 | cysteine-rich with EGF-like domains 1 | 203368_at | 2.82 | 1.44 |
| SMURF1 | SMAD specific E3 ubiquitin protein ligase 1 | 215458_s_at | 2.82 | 2.72 |
| POLH | polymerase (DNA directed), eta | 1557701_s_at | 2.82 | 1.00 |
| GPM6B | glycoprotein M6B | 209167_at | 2.82 | 2.45 |
| PLAUR | plasminogen activator, urokinase receptor | 211924_s_at | 2.82 | 0.33 |
| SLC25A39 | solute carrier family 25, member 39 | 223649_s_at | 2.82 | 0.19 |
| GLRB | glycine receptor, beta | 205279_s_at | 2.82 | 0.33 |
| HFE | hemochromatosis | 211866_x_at | 2.82 | 0.55 |
| RABL2A /// RABL2B | RAB, member of RAS oncogene family-like 2A /// RAB, member of RAS oncogene family-like 2B | 220500_s_at | 2.82 | 0.44 |
| SETDB1 | SET domain, bifurcated 1 | 214197_s_at | 2.81 | 1.44 |
| POLR2A | polymerase (RNA) II (DNA directed) polypeptide A, 220kDa | 202725_at | 2.81 | 0.26 |
| ZNF331 | zinc finger protein 331 | 227613_at | 2.81 | 0.19 |
| SLC25A37 | solute carrier family 25, member 37 | 218978_s_at | 2.81 | 0.44 |
| C19orf63 | chromosome 19 open reading frame 63 | 224727_at | 2.81 | 0.23 |
| B3GALNT2 | beta-1,3-N-acetylgalactosaminyltransferase 2 | 1553691_at | 2.81 | 3.67 |
| PDGFC | platelet derived growth factor C | 222719_s_at | 2.81 | 0.23 |
| VPS53 | vacuolar protein sorting 53 homolog (S. cerevisiae) | 1557112_a_at | 2.81 | 1.00 |
| G3BP2 | GTPase activating protein (SH3 domain) binding protein 2 | 206383_s_at | 2.81 | 0.14 |
| PAPD4 | PAP associated domain containing 4 | 238706_at | 2.80 | 0.73 |
| TRPV4 | transient receptor potential cation channel, subfamily V, member 4 | 219516_at | 2.80 | 0.86 |
| DLG1 | discs, large homolog 1 (Drosophila) | 217208_s_at | 2.80 | 0.44 |
| DAB2IP | DAB2 interacting protein | 228942_s_at | 2.80 | 1.00 |
| SRPR | signal recognition particle receptor (docking protein) | 200918_s_at | 2.80 | 0.23 |
| MGC16384 | Hypothetical LOC114130 | 234011_at | 2.80 | 6.81 |
| DDAH1 | Dimethylarginine dimethylaminohydrolase 1 | 229456_s_at | 2.80 | 0.86 |
| ABHD12 | abhydrolase domain containing 12 | 228123_s_at | 2.80 | 0.14 |
| SEPT2 | septin 2 | 1554747_a_at | 2.79 | 0.86 |
| SNAI1 | snail homolog 1 (Drosophila) | 219480_at | 2.79 | 1.00 |
| FLJ33544 | hypothetical LOC728283 | 1564157_at | 2.79 | 1.25 |
| ZCCHC4 | zinc finger, CCHC domain containing 4 | 220473_s_at | 2.79 | 0.14 |
| GEMIN4 | gem (nuclear organelle) associated protein 4 | 217099_s_at | 2.79 | 0.44 |
| BIN1 | bridging integrator 1 | 210201_x_at | 2.79 | 0.26 |
| BCL9L | B-cell CLL/lymphoma 9-like | 228065_at | 2.78 | 0.33 |
| NOV | nephroblastoma overexpressed gene | 204501_at | 2.78 | 1.44 |
| ZNF208 | zinc finger protein 208 | 1568646_x_at | 2.78 | 1.00 |
| BIN1 | bridging integrator 1 | 202931_x_at | 2.78 | 0.14 |
| CKAP5 | cytoskeleton associated protein 5 | 1555278_a_at | 2.78 | 0.26 |
| NCRNA00032 | Non-protein coding RNA 32 | 1559293_x_at | 2.78 | 4.15 |
| TRIM59 | tripartite motif-containing 59 | 235476_at | 2.78 | 0.73 |
| OSBP | oxysterol binding protein | 201799_s_at | 2.78 | 0.44 |
| MAP2K7 | mitogen-activated protein kinase kinase 7 | 209951_s_at | 2.77 | 0.23 |
| ZNF480 | zinc finger protein 480 | 222283_at | 2.77 | 0.86 |
| NAB2 | NGFI-A binding protein 2 (EGR1 binding protein 2) | 212803_at | 2.77 | 0.63 |
| BACH1 | BTB and CNC homology 1, basic leucine zipper transcription factor 1 | 210818_s_at | 2.77 | 1.00 |
| FOLR1 | folate receptor 1 (adult) | 211074_at | 2.77 | 0.26 |
| ADAMTS1 | ADAM metallopeptidase with thrombospondin type 1 motif, 1 | 222486_s_at | 2.77 | 2.06 |
| NRP2 | neuropilin 2 | 211844_s_at | 2.77 | 6.81 |
| SNAP23 | synaptosomal-associated protein, 23kDa | 209131_s_at | 2.77 | 0.44 |
| ADPRH | ADP-ribosylarginine hydrolase | 238505_at | 2.77 | 0.63 |
| CBX5 | chromobox homolog 5 | 234990_at | 2.77 | 2.72 |
| HLA-G | major histocompatibility complex, class I, G | 211528_x_at | 2.77 | 0.55 |
| NBR1 | neighbor of BRCA1 gene 1 | 201383_s_at | 2.77 | 0.33 |
| GNB4 | guanine nucleotide binding protein (G protein), beta polypeptide 4 | 223487_x_at | 2.77 | 1.44 |
| AK2 | adenylate kinase 2 | 212172_at | 2.76 | 2.72 |
| ALDH3B1 | aldehyde dehydrogenase 3 family, member B1 | 205640_at | 2.76 | 0.73 |
| LRRC27 | leucine rich repeat containing 27 | 1558483_at | 2.76 | 0.73 |
| HFE | hemochromatosis | 211327_x_at | 2.76 | 2.45 |
| GPC1 | glypican 1 | 202755_s_at | 2.76 | 0.35 |
| TNRC6B | Trinucleotide repeat containing 6B | 240044_x_at | 2.76 | 4.15 |
| NIPSNAP1 | nipsnap homolog 1 (C. elegans) | 201708_s_at | 2.76 | 0.14 |
| CAMKK2 | calcium/calmodulin-dependent protein kinase kinase 2, beta | 212252_at | 2.76 | 0.26 |
| SENP1 | SUMO1/sentrin specific peptidase 1 | 1552812_a_at | 2.76 | 0.33 |
| HCFC1R1 | host cell factor C1 regulator 1 (XPO1 dependent) | 218537_at | 2.76 | 1.44 |
| ABHD10 | abhydrolase domain containing 10 | 222697_s_at | 2.76 | 0.33 |
| COCH | coagulation factor C homolog, cochlin (Limulus polyphemus) | 1554241_at | 2.76 | 0.55 |
| HMGA1 | high mobility group AT-hook 1 | 206074_s_at | 2.76 | 0.33 |
| F7 | coagulation factor VII (serum prothrombin conversion accelerator) | 207300_s_at | 2.76 | 0.55 |
| MAOA | monoamine oxidase A | 212741_at | 2.76 | 0.86 |
| C3orf23 | chromosome 3 open reading frame 23 | 241666_at | 2.76 | 0.63 |
| IKBKG | inhibitor of kappa light polypeptide gene enhancer in B-cells, kinase gamma | 36004_at | 2.75 | 0.19 |
| ADAMTS5 | ADAM metallopeptidase with thrombospondin type 1 motif, 5 | 1558636_s_at | 2.75 | 1.25 |
| CCBE1 | collagen and calcium binding EGF domains 1 | 243805_at | 2.75 | 0.33 |
| STK11 | serine/threonine kinase 11 | 41657_at | 2.75 | 0.14 |
| ITGA7 | integrin, alpha 7 | 216331_at | 2.75 | 0.55 |
| CNN2 | calponin 2 | 201605_x_at | 2.75 | 0.55 |
| CYGB | cytoglobin | 226632_at | 2.75 | 0.86 |
| ESCO1 | establishment of cohesion 1 homolog 1 (S. cerevisiae) | 235645_at | 2.75 | 0.44 |
| GDI1 | GDP dissociation inhibitor 1 | 201864_at | 2.75 | 0.08 |
| YIF1B | Yip1 interacting factor homolog B (S. cerevisiae) | 226437_at | 2.75 | 0.00 |
| LPPR2 | lipid phosphate phosphatase-related protein type 2 | 64899_at | 2.75 | 0.19 |
| STK10 | serine/threonine kinase 10 | 203047_at | 2.75 | 0.44 |
| DPYSL4 | dihydropyrimidinase-like 4 | 205492_s_at | 2.74 | 2.45 |
| ABCD1 | ATP-binding cassette, sub-family D (ALD), member 1 | 205142_x_at | 2.74 | 0.33 |
| RCN3 | reticulocalbin 3, EF-hand calcium binding domain | 61734_at | 2.74 | 0.14 |
| AGPAT1 | 1-acylglycerol-3-phosphate O-acyltransferase 1 (lysophosphatidic acid acyltransferase, alpha) | 215535_s_at | 2.74 | 0.14 |
| KITLG | KIT ligand | 207029_at | 2.74 | 7.84 |
| PACS1 | phosphofurin acidic cluster sorting protein 1 | 224658_x_at | 2.74 | 0.19 |
| SLC35A4 | solute carrier family 35, member A4 | 224626_at | 2.74 | 0.33 |
| BAI2 | brain-specific angiogenesis inhibitor 2 | 204966_at | 2.74 | 0.26 |
| NRG1 | neuregulin 1 | 208230_s_at | 2.74 | 0.44 |
| SETX | senataxin | 232229_at | 2.74 | 0.63 |
| TPR | translocated promoter region (to activated MET oncogene) | 215220_s_at | 2.74 | 0.86 |
| KYNU | kynureninase (L-kynurenine hydrolase) | 217388_s_at | 2.73 | 4.15 |
| UBTF | upstream binding transcription factor, RNA polymerase I | 214881_s_at | 2.73 | 0.44 |
| PNPLA3 | patatin-like phospholipase domain containing 3 | 220675_s_at | 2.73 | 1.67 |
| LATS1 | LATS, large tumor suppressor, homolog 1 (Drosophila) | 219813_at | 2.73 | 3.21 |
| ARHGAP22 | Rho GTPase activating protein 22 | 206298_at | 2.73 | 0.73 |
| PROM2 | prominin 2 | 239528_at | 2.73 | 1.25 |
| CHKB-CPT1B /// CPT1B | choline kinase-like, carnitine palmitoyltransferase 1B (muscle) transcription unit /// carnitine palmitoyltransferase 1B (muscle) | 210070_s_at | 2.73 | 0.73 |
| LASS6 | LAG1 homolog, ceramide synthase 6 | 242019_at | 2.73 | 1.44 |
| LOC643988 | hypothetical LOC643988 | 227185_at | 2.73 | 0.86 |
| JUNB | jun B proto-oncogene | 201473_at | 2.73 | 0.86 |
| IGFBP5 | insulin-like growth factor binding protein 5 | 203425_s_at | 2.73 | 1.25 |
| SLFN5 | schlafen family member 5 | 1557078_at | 2.73 | 2.06 |
| GPS1 | G protein pathway suppressor 1 | 217782_s_at | 2.73 | 0.19 |
| LAPTM5 | lysosomal protein transmembrane 5 | 201721_s_at | 2.73 | 0.35 |
| FLNA | filamin A, alpha | 200859_x_at | 2.73 | 0.35 |
| ARFIP2 | ADP-ribosylation factor interacting protein 2 | 202109_at | 2.73 | 0.14 |
| PARP6 | poly (ADP-ribose) polymerase family, member 6 | 234710_s_at | 2.72 | 0.23 |
| ZFPL1 | zinc finger protein-like 1 | 209428_s_at | 2.72 | 0.26 |
| BVES | blood vessel epicardial substance | 223853_at | 2.72 | 0.33 |
| GANAB | glucosidase, alpha; neutral AB | 211934_x_at | 2.72 | 0.33 |
| OCRL | oculocerebrorenal syndrome of Lowe | 208316_s_at | 2.72 | 0.19 |
| MYSM1 | Myb-like, SWIRM and MPN domains 1 | 238644_at | 2.72 | 4.15 |
| HNRNPA3 /// HNRNPA3P1 | heterogeneous nuclear ribonucleoprotein A3 /// heterogeneous nuclear ribonucleoprotein A3 pseudogene 1 | 1555653_at | 2.72 | 0.19 |
| KIAA0494 | KIAA0494 | 201775_s_at | 2.72 | 0.33 |
| DMWD | dystrophia myotonica, WD repeat containing | 1554429_a_at | 2.71 | 0.19 |
| LRP1 | low density lipoprotein receptor-related protein 1 | 200784_s_at | 2.71 | 0.35 |
| HEATR2 | HEAT repeat containing 2 | 1554761_a_at | 2.71 | 0.23 |
| NPAS2 | neuronal PAS domain protein 2 | 205459_s_at | 2.71 | 0.44 |
| SQLE | squalene epoxidase | 213562_s_at | 2.71 | 1.00 |
| FGF7 | fibroblast growth factor 7 | 1555103_s_at | 2.71 | 0.33 |
| ZSCAN18 | zinc finger and SCAN domain containing 18 | 217593_at | 2.71 | 0.86 |
| TLE4 | transducin-like enhancer of split 4 (E(sp1) homolog, Drosophila) | 233575_s_at | 2.71 | 2.06 |
| PTCRA | pre T-cell antigen receptor alpha | 211252_x_at | 2.71 | 0.86 |
| CYTH3 | cytohesin 3 | 206523_at | 2.71 | 4.15 |
| MPZL2 | myelin protein zero-like 2 | 230518_at | 2.70 | 1.44 |
| BCAT1 | branched chain amino-acid transaminase 1, cytosolic | 214390_s_at | 2.70 | 0.55 |
| EPHA4 | EPH receptor A4 | 229374_at | 2.70 | 0.55 |
| PPP1CB | Protein phosphatase 1, catalytic subunit, beta isozyme | 228222_at | 2.70 | 0.55 |
| SSX2IP | synovial sarcoma, X breakpoint 2 interacting protein | 203015_s_at | 2.70 | 2.45 |
| DUSP3 | dual specificity phosphatase 3 | 201538_s_at | 2.70 | 0.23 |
| NONO | non-POU domain containing, octamer-binding | 210470_x_at | 2.69 | 0.35 |
| NGF | nerve growth factor (beta polypeptide) | 206814_at | 2.69 | 0.55 |
| SORBS3 | sorbin and SH3 domain containing 3 | 209253_at | 2.69 | 0.26 |
| LMNA | lamin A/C | 212089_at | 2.69 | 0.44 |
| DDAH1 | dimethylarginine dimethylaminohydrolase 1 | 1553565_s_at | 2.69 | 3.67 |
| LENG8 | leukocyte receptor cluster (LRC) member 8 | 224673_at | 2.69 | 0.08 |
| OBFC2B | oligonucleotide/oligosaccharide-binding fold containing 2B | 218903_s_at | 2.69 | 0.23 |
| TNS1 | tensin 1 | 221747_at | 2.69 | 1.25 |
| SNAP25 | Synaptosomal-associated protein, 25kDa | 1556629_a_at | 2.69 | 0.73 |
| PDPN | podoplanin | 204879_at | 2.69 | 2.45 |
| LARP4 | La ribonucleoprotein domain family, member 4 | 238960_s_at | 2.69 | 1.67 |
| MKLN1 | muskelin 1, intracellular mediator containing kelch motifs | 244171_at | 2.69 | 2.06 |
| ELP2 | elongation protein 2 homolog (S. cerevisiae) | 243735_at | 2.69 | 2.45 |
| PLK1 | polo-like kinase 1 | 202240_at | 2.69 | 1.00 |
| KPNA1 | karyopherin alpha 1 (importin alpha 5) | 202058_s_at | 2.69 | 0.86 |
| COPE | coatomer protein complex, subunit epsilon | 201264_at | 2.69 | 0.33 |
| SIX5 | SIX homeobox 5 | 229009_at | 2.69 | 0.26 |
| SCARB1 | scavenger receptor class B, member 1 | 201819_at | 2.68 | 1.44 |
| LAMB2P1 | laminin, beta 2 pseudogene 1 | 230616_at | 2.68 | 3.21 |
| OAS2 | 2'-5'-oligoadenylate synthetase 2, 69/71kDa | 206553_at | 2.68 | 4.15 |
| CNOT6L | CCR4-NOT transcription complex, subunit 6-like | 238438_at | 2.68 | 0.44 |
| ARHGEF11 | Rho guanine nucleotide exchange factor (GEF) 11 | 202914_s_at | 2.68 | 0.73 |
| ATP11B | ATPase, class VI, type 11B | 238811_at | 2.68 | 5.82 |
| BCL2L1 | BCL2-like 1 | 212312_at | 2.68 | 0.55 |
| RNF19B | ring finger protein 19B | 213038_at | 2.68 | 0.44 |
| TRIO | triple functional domain (PTPRF interacting) | 208178_x_at | 2.68 | 0.33 |
| PCBD1 | pterin-4 alpha-carbinolamine dehydratase/dimerization cofactor of hepatocyte nuclear factor 1 alpha | 203557_s_at | 2.68 | 0.26 |
| CAPRIN1 | cell cycle associated protein 1 | 200722_s_at | 2.68 | 0.86 |
| ST3GAL2 | ST3 beta-galactoside alpha-2,3-sialyltransferase 2 | 205346_at | 2.68 | 1.67 |
| PPP1R11 | protein phosphatase 1, regulatory (inhibitor) subunit 11 | 1566303_s_at | 2.67 | 0.33 |
| PLA2G15 | phospholipase A2, group XV | 204458_at | 2.67 | 0.86 |
| C9orf5 | chromosome 9 open reading frame 5 | 223005_s_at | 2.67 | 0.73 |
| SIGMAR1 | sigma non-opioid intracellular receptor 1 | 214484_s_at | 2.67 | 0.63 |
| MAMDC2 | MAM domain containing 2 | 228885_at | 2.67 | 1.67 |
| AP3S2 | adaptor-related protein complex 3, sigma 2 subunit | 213215_at | 2.67 | 0.26 |
| ZNF335 | zinc finger protein 335 | 78330_at | 2.67 | 9.00 |
| WTAP | Wilms tumor 1 associated protein | 1560274_at | 2.67 | 1.44 |
| GFM2 | G elongation factor, mitochondrial 2 | 231917_at | 2.67 | 1.25 |
| SLAIN2 | SLAIN motif family, member 2 | 233230_s_at | 2.67 | 0.44 |
| FYTTD1 | forty-two-three domain containing 1 | 224642_at | 2.67 | 0.44 |
| NLRX1 | NLR family member X1 | 1553695_a_at | 2.66 | 0.26 |
| VDAC1 | voltage-dependent anion channel 1 | 217140_s_at | 2.66 | 0.14 |
| NFYA | nuclear transcription factor Y, alpha | 204109_s_at | 2.66 | 0.14 |
| TIRAP | toll-interleukin 1 receptor (TIR) domain containing adaptor protein | 1554091_a_at | 2.66 | 0.55 |
| NMT1 | N-myristoyltransferase 1 | 201159_s_at | 2.66 | 0.44 |
| ND2 | MTND2 | 1553551_s_at | 2.66 | 0.14 |
| RAP2A /// RAP2B | RAP2A, member of RAS oncogene family /// RAP2B, member of RAS oncogene family | 214487_s_at | 2.66 | 0.55 |
| CPE | carboxypeptidase E | 201116_s_at | 2.66 | 9.00 |
| PRIM2 | primase, DNA, polypeptide 2 (58kDa) | 215708_s_at | 2.66 | 3.21 |
| LMF2 | lipase maturation factor 2 | 31837_at | 2.66 | 0.14 |
| MRPL30 | mitochondrial ribosomal protein L30 | 224173_s_at | 2.66 | 0.33 |
| DEDD | death effector domain containing | 211255_x_at | 2.65 | 0.23 |
| LPP | LIM domain containing preferred translocation partner in lipoma | 235000_at | 2.65 | 0.44 |
| AKT1S1 | AKT1 substrate 1 (proline-rich) | 1555821_a_at | 2.65 | 0.55 |
| AGPAT3 | 1-acylglycerol-3-phosphate O-acyltransferase 3 | 225440_at | 2.65 | 0.86 |
| H1FX | H1 histone family, member X | 204805_s_at | 2.65 | 0.55 |
| SCARB2 | scavenger receptor class B, member 2 | 201647_s_at | 2.65 | 1.25 |
| CDK10 | cyclin-dependent kinase 10 | 210622_x_at | 2.65 | 0.33 |
| WIZ | widely interspaced zinc finger motifs | 221783_at | 2.65 | 0.63 |
| DIP2C | DIP2 disco-interacting protein 2 homolog C (Drosophila) | 1565681_s_at | 2.65 | 0.23 |
| SERPINB2 | serpin peptidase inhibitor, clade B (ovalbumin), member 2 | 204614_at | 2.65 | 3.67 |
| ARL8A | ADP-ribosylation factor-like 8A | 225347_at | 2.65 | 0.26 |
| SFSWAP | splicing factor, suppressor of white-apricot homolog (Drosophila) | 202773_s_at | 2.65 | 0.44 |
| ITFG3 | integrin alpha FG-GAP repeat containing 3 | 224749_at | 2.64 | 0.14 |
| ARHGEF19 | Rho guanine nucleotide exchange factor (GEF) 19 | 226857_at | 2.64 | 1.00 |
| PRKACA | protein kinase, cAMP-dependent, catalytic, alpha | 202801_at | 2.64 | 0.23 |
| HOXA6 | homeobox A6 | 208557_at | 2.64 | 1.67 |
| DNAJB12 | DnaJ (Hsp40) homolog, subfamily B, member 12 | 202865_at | 2.64 | 0.19 |
| SUPT6H | suppressor of Ty 6 homolog (S. cerevisiae) | 208831_x_at | 2.64 | 0.26 |
| NUP188 | nucleoporin 188kDa | 212691_at | 2.64 | 0.33 |
| PLEC | plectin | 201373_at | 2.64 | 0.73 |
| TBC1D10B | TBC1 domain family, member 10B | 220947_s_at | 2.64 | 0.14 |
| TRIO | triple functional domain (PTPRF interacting) | 209011_at | 2.64 | 0.73 |
| EML4 | echinoderm microtubule associated protein like 4 | 223069_s_at | 2.64 | 0.63 |
| KLHDC3 | kelch domain containing 3 | 214383_x_at | 2.64 | 0.26 |
| PVR | poliovirus receptor | 214443_at | 2.63 | 0.73 |
| LAPTM4B | lysosomal protein transmembrane 4 beta | 208767_s_at | 2.63 | 0.44 |
| C2orf81 | chromosome 2 open reading frame 81 | 236070_at | 2.63 | 1.25 |
| SLC39A6 | solute carrier family 39 (zinc transporter), member 6 | 1555460_a_at | 2.63 | 0.63 |
| SLC39A13 | solute carrier family 39 (zinc transporter), member 13 | 1552295_a_at | 2.63 | 0.26 |
| ARF1 | ADP-ribosylation factor 1 | 208750_s_at | 2.63 | 0.35 |
| HHEX | hematopoietically expressed homeobox | 215933_s_at | 2.63 | 0.73 |
| CNOT3 | CCR4-NOT transcription complex, subunit 3 | 211141_s_at | 2.63 | 1.25 |
| IGDCC3 | immunoglobulin superfamily, DCC subclass, member 3 | 232037_at | 2.63 | 0.73 |
| ANTXR2 | anthrax toxin receptor 2 | 1555536_at | 2.63 | 5.82 |
| CAPZB | capping protein (actin filament) muscle Z-line, beta | 201950_x_at | 2.63 | 0.23 |
| USP34 | ubiquitin specific peptidase 34 | 212065_s_at | 2.62 | 0.63 |
| KRBA2 | KRAB-A domain containing 2 | 1558533_at | 2.62 | 4.15 |
| PDE4D | phosphodiesterase 4D, cAMP-specific | 210837_s_at | 2.62 | 0.86 |
| YWHAE | tyrosine 3-monooxygenase/tryptophan 5-monooxygenase activation protein, epsilon polypeptide | 210996_s_at | 2.62 | 0.26 |
| SLFN5 | schlafen family member 5 | 1553055_a_at | 2.62 | 1.44 |
| PDXP /// SH3BP1 | pyridoxal (pyridoxine, vitamin B6) phosphatase /// SH3-domain binding protein 1 | 223290_at | 2.62 | 0.44 |
| C19orf61 | chromosome 19 open reading frame 61 | 221335_x_at | 2.61 | 0.44 |
| HDLBP | high density lipoprotein binding protein | 200643_at | 2.61 | 0.26 |
| MAP4K5 | mitogen-activated protein kinase kinase kinase kinase 5 | 211081_s_at | 2.61 | 1.25 |
| CPNE1 | copine I | 206918_s_at | 2.61 | 0.23 |
| ABHD6 | abhydrolase domain containing 6 | 221679_s_at | 2.61 | 0.26 |
| HMG20B | high-mobility group 20B | 210719_s_at | 2.61 | 0.33 |
| ENO2 | enolase 2 (gamma, neuronal) | 201313_at | 2.61 | 1.67 |
| P4HB | prolyl 4-hydroxylase, beta polypeptide | 200656_s_at | 2.61 | 0.33 |
| DAGLB | diacylglycerol lipase, beta | 225833_at | 2.61 | 1.25 |
| FAM115A | family with sequence similarity 115, member A | 210529_s_at | 2.61 | 0.35 |
| EDIL3 | EGF-like repeats and discoidin I-like domains 3 | 1558643_s_at | 2.61 | 3.67 |
| POM121L2 | POM121 membrane glycoprotein-like 2 | 216582_at | 2.61 | 2.72 |
| SMYD3 | SET and MYND domain containing 3 | 218788_s_at | 2.61 | 0.44 |
| STAT4 | signal transducer and activator of transcription 4 | 206118_at | 2.61 | 0.26 |
| SPIRE1 | spire homolog 1 (Drosophila) | 1559517_a_at | 2.60 | 0.86 |
| SPATA5L1 | spermatogenesis associated 5-like 1 | 215694_at | 2.60 | 1.00 |
| CXXC1 | CXXC finger protein 1 | 218058_at | 2.60 | 0.23 |
| C1orf144 | chromosome 1 open reading frame 144 | 212005_at | 2.60 | 0.86 |
| NRP2 | neuropilin 2 | 214632_at | 2.60 | 0.63 |
| CANX | calnexin | 208853_s_at | 2.60 | 0.73 |
| IGFBP5 | insulin-like growth factor binding protein 5 | 203426_s_at | 2.60 | 1.00 |
| TMCO3 | transmembrane and coiled-coil domains 3 | 220240_s_at | 2.60 | 0.44 |
| TMEM158 | transmembrane protein 158 (gene/pseudogene) | 213338_at | 2.59 | 0.73 |
| WWC1 | WW and C2 domain containing 1 | 213085_s_at | 2.59 | 1.00 |
| PFKL | phosphofructokinase, liver | 211065_x_at | 2.59 | 0.55 |
| KBTBD2 | kelch repeat and BTB (POZ) domain containing 2 | 223584_s_at | 2.59 | 0.44 |
| SLC8A1 | solute carrier family 8 (sodium/calcium exchanger), member 1 | 211805_s_at | 2.59 | 3.21 |
| MSH6 | mutS homolog 6 (E. coli) | 211450_s_at | 2.59 | 0.55 |
| ABI2 | abl-interactor 2 | 211793_s_at | 2.59 | 0.33 |
| TMEM38B | transmembrane protein 38B | 222736_s_at | 2.59 | 3.21 |
| ABCB4 | ATP-binding cassette, sub-family B (MDR/TAP), member 4 | 207819_s_at | 2.59 | 2.06 |
| PRKCD | protein kinase C, delta | 202545_at | 2.59 | 0.55 |
| SLC48A1 | solute carrier family 48 (heme transporter), member 1 | 218417_s_at | 2.59 | 0.26 |
| SLC26A6 | solute carrier family 26, member 6 | 221572_s_at | 2.59 | 0.73 |
| LOC100132815 | hypothetical LOC100132815 | 1560446_at | 2.59 | 4.93 |
| HS1BP3 | HCLS1 binding protein 3 | 219020_at | 2.59 | 0.33 |
| CLIC6 | chloride intracellular channel 6 | 242913_at | 2.58 | 3.21 |
| C10orf58 | chromosome 10 open reading frame 58 | 224435_at | 2.58 | 3.21 |
| GPSM2 | G-protein signaling modulator 2 | 230002_at | 2.58 | 0.86 |
| MBOAT7 | membrane bound O-acyltransferase domain containing 7 | 209179_s_at | 2.58 | 0.26 |
| UAP1L1 | UDP-N-acteylglucosamine pyrophosphorylase 1-like 1 | 214755_at | 2.58 | 0.33 |
| BCL7A | B-cell CLL/lymphoma 7A | 203795_s_at | 2.58 | 1.00 |
| POU4F1 | POU class 4 homeobox 1 | 206940_s_at | 2.58 | 3.21 |
| CDK8 | cyclin-dependent kinase 8 | 1553112_s_at | 2.58 | 0.63 |
| THADA | thyroid adenoma associated | 1554493_s_at | 2.58 | 0.63 |
| HOMER2 | homer homolog 2 (Drosophila) | 217080_s_at | 2.58 | 0.86 |
| ULK2 | unc-51-like kinase 2 (C. elegans) | 1554112_a_at | 2.58 | 0.23 |
| PRNP | prion protein | 215707_s_at | 2.57 | 0.63 |
| ADRBK2 | adrenergic, beta, receptor kinase 2 | 204184_s_at | 2.57 | 2.45 |
| FOXN3 | forkhead box N3 | 241984_at | 2.57 | 0.35 |
| MGAT4B | mannosyl (alpha-1,3-)-glycoprotein beta-1,4-N-acetylglucosaminyltransferase, isozyme B | 220189_s_at | 2.57 | 0.55 |
| ADSSL1 | adenylosuccinate synthase like 1 | 226325_at | 2.57 | 1.25 |
| PTGR1 | prostaglandin reductase 1 | 228824_s_at | 2.57 | 0.35 |
| TUBGCP3 | tubulin, gamma complex associated protein 3 | 215739_s_at | 2.57 | 0.35 |
| SESN2 | sestrin 2 | 223195_s_at | 2.57 | 0.73 |
| AMOTL1 | angiomotin like 1 | 225459_at | 2.57 | 0.63 |
| ALDH6A1 | aldehyde dehydrogenase 6 family, member A1 | 204290_s_at | 2.57 | 0.23 |
| TMEM119 | transmembrane protein 119 | 227300_at | 2.57 | 0.86 |
| TM7SF2 | transmembrane 7 superfamily member 2 | 210130_s_at | 2.57 | 1.00 |
| TNFAIP2 | tumor necrosis factor, alpha-induced protein 2 | 202510_s_at | 2.56 | 0.86 |
| ALKBH5 | alkB, alkylation repair homolog 5 (E. coli) | 228034_x_at | 2.56 | 1.00 |
| FAM47E /// STBD1 | family with sequence similarity 47, member E /// starch binding domain 1 | 203986_at | 2.56 | 1.44 |
| WIPF1 | WAS/WASL interacting protein family, member 1 | 202665_s_at | 2.56 | 0.44 |
| EIF4EBP2 | eukaryotic translation initiation factor 4E binding protein 2 | 208769_at | 2.56 | 0.73 |
| ANKRD13B | ankyrin repeat domain 13B | 227720_at | 2.56 | 0.44 |
| BAT3 | HLA-B associated transcript 3 | 213318_s_at | 2.56 | 0.19 |
| LCAT | lecithin-cholesterol acyltransferase | 204428_s_at | 2.56 | 1.25 |
| KLHL26 | kelch-like 26 (Drosophila) | 219354_at | 2.56 | 0.35 |
| SUPT6H | suppressor of Ty 6 homolog (S. cerevisiae) | 208830_s_at | 2.56 | 0.23 |
| MAB21L2 | mab-21-like 2 (C. elegans) | 210302_s_at | 2.56 | 1.44 |
| SLC11A2 | solute carrier family 11 (proton-coupled divalent metal ion transporters), member 2 | 203125_x_at | 2.56 | 0.44 |
| HOXA11 | homeobox A11 | 208493_at | 2.56 | 1.67 |
| PRELP | proline/arginine-rich end leucine-rich repeat protein | 37022_at | 2.56 | 0.44 |
| ARRDC1 | arrestin domain containing 1 | 226405_s_at | 2.56 | 0.26 |
| ZNF644 | zinc finger protein 644 | 1553725_s_at | 2.55 | 0.73 |
| DLG1 | discs, large homolog 1 (Drosophila) | 202516_s_at | 2.55 | 0.44 |
| TEAD2 | TEA domain family member 2 | 226408_at | 2.55 | 0.44 |
| MAOA | monoamine oxidase A | 204389_at | 2.55 | 1.25 |
| CTNND2 | catenin (cadherin-associated protein), delta 2 (neural plakophilin-related arm-repeat protein) | 209617_s_at | 2.55 | 2.72 |
| FAM65A | family with sequence similarity 65, member A | 218029_at | 2.55 | 0.33 |
| LARS | leucyl-tRNA synthetase | 223888_s_at | 2.55 | 9.00 |
| EGFR | epidermal growth factor receptor | 201983_s_at | 2.55 | 0.35 |
| SEZ6L2 | seizure related 6 homolog (mouse)-like 2 | 218720_x_at | 2.55 | 1.44 |
| CCDC71 | coiled-coil domain containing 71 | 219893_at | 2.55 | 2.06 |
| PTOV1 | prostate tumor overexpressed 1 | 212032_s_at | 2.55 | 0.35 |
| CENPJ | centromere protein J | 220885_s_at | 2.55 | 1.44 |
| RHOBTB3 | Rho-related BTB domain containing 3 | 216048_s_at | 2.55 | 1.00 |
| HCFC1R1 | host cell factor C1 regulator 1 (XPO1 dependent) | 45714_at | 2.55 | 1.67 |
| SYNJ2 | synaptojanin 2 | 1555009_a_at | 2.55 | 0.73 |
| RAD23A | RAD23 homolog A (S. cerevisiae) | 201046_s_at | 2.55 | 0.23 |
| RCC1 | regulator of chromosome condensation 1 | 215747_s_at | 2.55 | 1.00 |
| DDX54 | DEAD (Asp-Glu-Ala-Asp) box polypeptide 54 | 225428_s_at | 2.55 | 0.55 |
| SEZ6L2 | seizure related 6 homolog (mouse)-like 2 | 238406_x_at | 2.54 | 1.25 |
| CRK | v-crk sarcoma virus CT10 oncogene homolog (avian) | 202226_s_at | 2.54 | 0.35 |
| TRIP6 | thyroid hormone receptor interactor 6 | 209129_at | 2.54 | 0.33 |
| SEMA4F | sema domain, immunoglobulin domain (Ig), transmembrane domain (TM) and short cytoplasmic domain, (semaphorin) 4F | 208124_s_at | 2.54 | 0.55 |
| C5orf62 | chromosome 5 open reading frame 62 | 223276_at | 2.54 | 0.26 |
| CREB1 | cAMP responsive element binding protein 1 | 204312_x_at | 2.54 | 0.55 |
| ADC | arginine decarboxylase | 1554393_a_at | 2.54 | 1.00 |
| SBF1 | SET binding factor 1 | 212393_at | 2.54 | 0.23 |
| SLC2A14 /// SLC2A3 | solute carrier family 2 (facilitated glucose transporter), member 14 /// solute carrier family 2 (facilitated glucose transporter), member 3 | 216236_s_at | 2.54 | 0.63 |
| TLR4 | toll-like receptor 4 | 221060_s_at | 2.54 | 1.00 |
| UBXN4 | UBX domain protein 4 | 212008_at | 2.54 | 1.00 |
| SNAP25 | synaptosomal-associated protein, 25kDa | 202508_s_at | 2.53 | 2.06 |
| AVL9 | AVL9 homolog (S. cerevisiase) | 212475_at | 2.53 | 0.35 |
| CPSF3L | cleavage and polyadenylation specific factor 3-like | 233563_s_at | 2.53 | 0.26 |
| TPCN1 | two pore segment channel 1 | 1557186_s_at | 2.53 | 0.33 |
| MAFB | v-maf musculoaponeurotic fibrosarcoma oncogene homolog B (avian) | 222670_s_at | 2.53 | 0.86 |
| MPP2 | membrane protein, palmitoylated 2 (MAGUK p55 subfamily member 2) | 213270_at | 2.53 | 0.86 |
| LOC100128292 | hypothetical LOC100128292 | 1562598_at | 2.53 | 1.00 |
| AMACR | alpha-methylacyl-CoA racemase | 236365_at | 2.53 | 4.15 |
| FAM55C | family with sequence similarity 55, member C | 243011_at | 2.53 | 1.25 |
| CLIC4 | chloride intracellular channel 4 | 221881_s_at | 2.53 | 3.67 |
| SVIL | supervillin | 202566_s_at | 2.53 | 0.63 |
| SSBP3 | single stranded DNA binding protein 3 | 217991_x_at | 2.53 | 0.33 |
| CLOCK | clock homolog (mouse) | 204980_at | 2.53 | 0.33 |
| SPP1 | secreted phosphoprotein 1 | 209875_s_at | 2.53 | 4.15 |
| KLF12 | Kruppel-like factor 12 | 229881_at | 2.53 | 0.44 |
| MAT2A | methionine adenosyltransferase II, alpha | 200769_s_at | 2.52 | 1.00 |
| SLC8A1 | solute carrier family 8 (sodium/calcium exchanger), member 1 | 241752_at | 2.52 | 0.86 |
| VASN | vasorin | 225867_at | 2.52 | 0.73 |
| BCKDK | branched chain ketoacid dehydrogenase kinase | 202030_at | 2.52 | 0.44 |
| TPM4 | tropomyosin 4 | 1567107_s_at | 2.52 | 0.55 |
| ICAM1 | intercellular adhesion molecule 1 | 215485_s_at | 2.52 | 0.73 |
| VAT1 | vesicle amine transport protein 1 homolog (T. californica) | 208626_s_at | 2.52 | 0.26 |
| PVRL2 | poliovirus receptor-related 2 (herpesvirus entry mediator B) | 225418_at | 2.52 | 1.25 |
| HFE | hemochromatosis | 211329_x_at | 2.52 | 0.35 |
| TXNIP | thioredoxin interacting protein | 201009_s_at | 2.52 | 0.63 |
| SLC25A28 | solute carrier family 25, member 28 | 221432_s_at | 2.52 | 0.26 |
| MOBKL2C | MOB1, Mps One Binder kinase activator-like 2C (yeast) | 243052_at | 2.52 | 1.67 |
| BRPF3 | Bromodomain and PHD finger containing, 3 | 1565844_at | 2.52 | 9.00 |
| MAPK3 | mitogen-activated protein kinase 3 | 212046_x_at | 2.52 | 0.26 |
| SLC8A1 | solute carrier family 8 (sodium/calcium exchanger), member 1 | 210804_x_at | 2.52 | 3.21 |
| MIIP | migration and invasion inhibitory protein | 48659_at | 2.52 | 0.73 |
| SERPINF1 | serpin peptidase inhibitor, clade F (alpha-2 antiplasmin, pigment epithelium derived factor), member 1 | 202283_at | 2.52 | 1.25 |
| SDAD1 | SDA1 domain containing 1 | 218607_s_at | 2.52 | 0.63 |
| CDS1 | CDP-diacylglycerol synthase (phosphatidate cytidylyltransferase) 1 | 226185_at | 2.51 | 4.15 |
| PEX10 | peroxisomal biogenesis factor 10 | 206351_s_at | 2.51 | 0.35 |
| MAPK14 | mitogen-activated protein kinase 14 | 210449_x_at | 2.51 | 0.63 |
| NES | nestin | 218678_at | 2.51 | 3.21 |
| EML4 | echinoderm microtubule associated protein like 4 | 220386_s_at | 2.51 | 1.44 |
| EXPH5 | exophilin 5 | 214734_at | 2.51 | 0.44 |
| NBL1 | neuroblastoma, suppression of tumorigenicity 1 | 37005_at | 2.51 | 0.44 |
| XRN2 | 5'-3' exoribonuclease 2 | 233878_s_at | 2.51 | 1.00 |
| CD151 | CD151 molecule (Raph blood group) | 204306_s_at | 2.51 | 0.44 |
| ARSG | arylsulfatase G | 1552632_a_at | 2.51 | 1.00 |
| TRIO | triple functional domain (PTPRF interacting) | 209013_x_at | 2.51 | 0.55 |
| MXRA8 | matrix-remodelling associated 8 | 213422_s_at | 2.51 | 0.63 |
| PAK4 | p21 protein (Cdc42/Rac)-activated kinase 4 | 33814_at | 2.51 | 0.26 |
| PIK3C2A | phosphoinositide-3-kinase, class 2, alpha polypeptide | 1569022_a_at | 2.50 | 1.00 |
| OAS3 | 2'-5'-oligoadenylate synthetase 3, 100kDa | 218400_at | 2.50 | 2.72 |
| PIGQ | phosphatidylinositol glycan anchor biosynthesis, class Q | 204144_s_at | 2.50 | 1.25 |
| ZNF160 | zinc finger protein 160 | 239954_at | 2.50 | 2.72 |
| CMTM4 | CKLF-like MARVEL transmembrane domain containing 4 | 1554677_s_at | 2.50 | 2.45 |
| SRPK2 | SRSF protein kinase 2 | 214931_s_at | 2.50 | 0.44 |
| EIF4B | eukaryotic translation initiation factor 4B | 211937_at | 2.50 | 1.67 |
| MECP2 | methyl CpG binding protein 2 (Rett syndrome) | 202617_s_at | 2.50 | 0.44 |
| WBSCR16 | Williams-Beuren syndrome chromosome region 16 | 1554410_a_at | 2.50 | 1.25 |
| RBM42 | RNA binding motif protein 42 | 205740_s_at | 2.50 | 0.44 |
| OPTC | opticin | 223884_at | 2.50 | 2.06 |
| PDK2 | pyruvate dehydrogenase kinase, isozyme 2 | 202590_s_at | 2.50 | 0.26 |
| GSTCD | glutathione S-transferase, C-terminal domain containing | 1554518_at | 2.50 | 2.06 |
| PEX6 | peroxisomal biogenesis factor 6 | 204545_at | 2.50 | 1.25 |
| COL6A2 | collagen, type VI, alpha 2 | 213290_at | 2.50 | 0.63 |
| SFRS15 | Splicing factor, arginine/serine-rich 15 | 243759_at | 2.50 | 1.00 |
| NR1H2 | nuclear receptor subfamily 1, group H, member 2 | 218215_s_at | 2.50 | 0.44 |
| CRIP2 | cysteine-rich protein 2 | 208978_at | 2.50 | 1.25 |
| HBP1 | HMG-box transcription factor 1 | 207361_at | 2.49 | 1.00 |
| SYNGR2 | synaptogyrin 2 | 201079_at | 2.49 | 1.25 |
| GPR125 | G protein-coupled receptor 125 | 1555122_at | 2.49 | 3.21 |
| SLC16A3 | solute carrier family 16, member 3 (monocarboxylic acid transporter 4) | 202855_s_at | 2.49 | 0.86 |
| CAPNS1 | calpain, small subunit 1 | 200001_at | 2.49 | 0.63 |
| PLCG1 | phospholipase C, gamma 1 | 216551_x_at | 2.49 | 0.63 |
| FBXL6 | F-box and leucine-rich repeat protein 6 | 219189_at | 2.49 | 0.86 |
| SAMD13 | sterile alpha motif domain containing 13 | 229402_at | 2.49 | 1.25 |
| ST3GAL3 | ST3 beta-galactoside alpha-2,3-sialyltransferase 3 | 1555702_a_at | 2.49 | 0.63 |
| MTF1 | metal-regulatory transcription factor 1 | 205322_s_at | 2.49 | 0.86 |
| GPC6 | glypican 6 | 223730_at | 2.49 | 0.86 |
| MKRN2 | makorin ring finger protein 2 | 222510_s_at | 2.49 | 0.73 |
| SBF1 | SET binding factor 1 | 39835_at | 2.49 | 0.33 |
| METRN | meteorin, glial cell differentiation regulator | 219051_x_at | 2.49 | 0.86 |
| PHLDA1 | pleckstrin homology-like domain, family A, member 1 | 217999_s_at | 2.48 | 1.00 |
| RHOG | ras homolog gene family, member G (rho G) | 203175_at | 2.48 | 0.19 |
| PKD1 | polycystic kidney disease 1 (autosomal dominant) | 202328_s_at | 2.48 | 1.67 |
| SUPT6H | suppressor of Ty 6 homolog (S. cerevisiae) | 208420_x_at | 2.48 | 0.26 |
| HMOX2 | heme oxygenase (decycling) 2 | 218121_at | 2.48 | 4.15 |
| ALS2CL | ALS2 C-terminal like | 229887_at | 2.48 | 1.25 |
| LOC100130938 | hypothetical LOC100130938 | 230574_at | 2.48 | 0.86 |
| COL10A1 | collagen, type X, alpha 1 | 205941_s_at | 2.48 | 1.44 |
| EPOR | erythropoietin receptor | 396_f_at | 2.48 | 0.86 |
| KIAA1632 | KIAA1632 | 1563471_at | 2.47 | 1.44 |
| SMO | smoothened homolog (Drosophila) | 218629_at | 2.47 | 0.55 |
| BAT2 | HLA-B associated transcript 2 | 212081_x_at | 2.47 | 0.26 |
| REXO1 | REX1, RNA exonuclease 1 homolog (S. cerevisiae) | 226144_at | 2.47 | 0.73 |
| RIPK1 | receptor (TNFRSF)-interacting serine-threonine kinase 1 | 209941_at | 2.47 | 0.55 |
| PYGO2 | pygopus homolog 2 (Drosophila) | 225370_at | 2.47 | 0.35 |
| PDE5A | phosphodiesterase 5A, cGMP-specific | 1553175_s_at | 2.47 | 1.67 |
| TMED4 | transmembrane emp24 protein transport domain containing 4 | 224676_at | 2.47 | 0.35 |
| DGKZ | diacylglycerol kinase, zeta 104kDa | 207556_s_at | 2.47 | 2.06 |
| LMOD1 | leiomodin 1 (smooth muscle) | 203766_s_at | 2.47 | 2.06 |
| TRIM27 | tripartite motif-containing 27 | 210541_s_at | 2.47 | 0.33 |
| IPO8 | importin 8 | 205701_at | 2.47 | 1.00 |
| TNKS2 | tankyrase, TRF1-interacting ankyrin-related ADP-ribose polymerase 2 | 222563_s_at | 2.47 | 0.26 |
| PNKD | paroxysmal nonkinesigenic dyskinesia | 233177_s_at | 2.47 | 2.45 |
| ALKBH5 | alkB, alkylation repair homolog 5 (E. coli) | 1553101_a_at | 2.47 | 0.44 |
| FAM100B | family with sequence similarity 100, member B | 224785_at | 2.47 | 0.26 |
| MLL3 | myeloid/lymphoid or mixed-lineage leukemia 3 | 1557158_s_at | 2.47 | 1.00 |
| TMED2 | transmembrane emp24 domain trafficking protein 2 | 204426_at | 2.47 | 0.63 |
| UBL7 | ubiquitin-like 7 (bone marrow stromal cell-derived) | 225063_at | 2.46 | 0.33 |
| SLC25A1 | solute carrier family 25 (mitochondrial carrier; citrate transporter), member 1 | 210010_s_at | 2.46 | 0.23 |
| FOXN3 | forkhead box N3 | 205021_s_at | 2.46 | 0.44 |
| TRPM2 | transient receptor potential cation channel, subfamily M, member 2 | 205708_s_at | 2.46 | 3.67 |
| ZIK1 | zinc finger protein interacting with K protein 1 homolog (mouse) | 232774_x_at | 2.46 | 1.44 |
| PIK3CD | phosphoinositide-3-kinase, catalytic, delta polypeptide | 211230_s_at | 2.46 | 2.06 |
| KDELR1 | KDEL (Lys-Asp-Glu-Leu) endoplasmic reticulum protein retention receptor 1 | 1555575_a_at | 2.46 | 0.26 |
| AK2 | adenylate kinase 2 | 205996_s_at | 2.46 | 1.25 |
| PCM1 | pericentriolar material 1 | 209997_x_at | 2.46 | 0.55 |
| CD24 | CD24 molecule | 208650_s_at | 2.46 | 1.67 |
| MACROD1 | MACRO domain containing 1 | 219188_s_at | 2.46 | 0.55 |
| KATNB1 | katanin p80 (WD repeat containing) subunit B 1 | 203163_at | 2.46 | 0.63 |
| DNAJA4 | DnaJ (Hsp40) homolog, subfamily A, member 4 | 1554334_a_at | 2.46 | 1.00 |
| GALK1 | galactokinase 1 | 204374_s_at | 2.45 | 1.67 |
| SLC16A7 | solute carrier family 16, member 7 (monocarboxylic acid transporter 2) | 210807_s_at | 2.45 | 1.44 |
| THAP4 | THAP domain containing 4 | 220417_s_at | 2.45 | 0.33 |
| SMYD5 | SMYD family member 5 | 209516_at | 2.45 | 0.26 |
| ST3GAL3 | ST3 beta-galactoside alpha-2,3-sialyltransferase 3 | 225905_s_at | 2.45 | 0.55 |
| HEYL | hairy/enhancer-of-split related with YRPW motif-like | 226828_s_at | 2.45 | 3.67 |
| GPM6B | glycoprotein M6B | 209168_at | 2.45 | 2.45 |
| PPP2R4 | protein phosphatase 2A activator, regulatory subunit 4 | 216105_x_at | 2.45 | 0.86 |
| BRD4 | bromodomain containing 4 | 226052_at | 2.45 | 0.63 |
| FOXO3 /// FOXO3B | forkhead box O3 /// forkhead box O3B pseudogene | 204132_s_at | 2.45 | 0.55 |
| IGHG1 | immunoglobulin heavy constant gamma 1 (G1m marker) | 217369_at | 2.45 | 0.73 |
| CAMK1G | calcium/calmodulin-dependent protein kinase IG | 217128_s_at | 2.45 | 0.44 |
| BBS5 | Bardet-Biedl syndrome 5 | 230697_at | 2.45 | 0.86 |
| C19orf66 | chromosome 19 open reading frame 66 | 1555491_a_at | 2.45 | 0.73 |
| LPPR2 | lipid phosphate phosphatase-related protein type 2 | 218509_at | 2.44 | 0.26 |
| TRBC1 | T cell receptor beta constant 1 | 213193_x_at | 2.44 | 0.63 |
| PIP4K2B | phosphatidylinositol-5-phosphate 4-kinase, type II, beta | 201081_s_at | 2.44 | 0.44 |
| CCDC88A | coiled-coil domain containing 88A | 221078_s_at | 2.44 | 1.25 |
| ATXN7 | ataxin 7 | 209964_s_at | 2.44 | 0.23 |
| TRAF7 | TNF receptor-associated factor 7 | 223031_s_at | 2.44 | 0.44 |
| NFYA | nuclear transcription factor Y, alpha | 215720_s_at | 2.44 | 1.44 |
| CRTAP | cartilage associated protein | 1554464_a_at | 2.44 | 0.86 |
| GPAA1 | glycosylphosphatidylinositol anchor attachment protein 1 homolog (yeast) | 201618_x_at | 2.44 | 0.33 |
| LOC402778 | CD225 family protein FLJ76511 | 227863_at | 2.44 | 1.44 |
| MKX | mohawk homeobox | 241902_at | 2.44 | 9.00 |
| FXYD3 | FXYD domain containing ion transport regulator 3 | 202488_s_at | 2.44 | 4.15 |
| CAMTA2 | calmodulin binding transcription activator 2 | 212948_at | 2.44 | 0.26 |
| WDR48 | WD repeat domain 48 | 222157_s_at | 2.44 | 0.73 |
| SOS2 | son of sevenless homolog 2 (Drosophila) | 211665_s_at | 2.43 | 0.86 |
| PTRF | polymerase I and transcript release factor | 1557938_s_at | 2.43 | 0.33 |
| RIPK3 | receptor-interacting serine-threonine kinase 3 | 228139_at | 2.43 | 1.00 |
| PLIN2 | perilipin 2 | 209122_at | 2.43 | 1.44 |
| SGSM3 | small G protein signaling modulator 3 | 215519_x_at | 2.43 | 0.55 |
| TANC1 | tetratricopeptide repeat, ankyrin repeat and coiled-coil containing 1 | 234870_at | 2.43 | 2.72 |
| WDR59 | WD repeat domain 59 | 228295_at | 2.43 | 2.06 |
| HLA-C | major histocompatibility complex, class I, C | 211799_x_at | 2.43 | 1.00 |
| EVI5L | ecotropic viral integration site 5-like | 226411_at | 2.43 | 0.35 |
| RAPGEF5 | Rap guanine nucleotide exchange factor (GEF) 5 | 204681_s_at | 2.43 | 1.25 |
| PDE8A | phosphodiesterase 8A | 1552931_a_at | 2.43 | 1.00 |
| PPIF | peptidylprolyl isomerase F | 201490_s_at | 2.43 | 0.86 |
| CHD3 | chromodomain helicase DNA binding protein 3 | 208806_at | 2.42 | 1.25 |
| ULK1 | unc-51-like kinase 1 (C. elegans) | 209333_at | 2.42 | 0.86 |
| MYL4 | myosin, light chain 4, alkali; atrial, embryonic | 210395_x_at | 2.42 | 2.06 |
| FAHD2A /// LOC285014 | fumarylacetoacetate hydrolase domain containing 2A /// hypothetical protein LOC285014 | 235418_at | 2.42 | 7.84 |
| ABCF2 | ATP-binding cassette, sub-family F (GCN20), member 2 | 207623_at | 2.42 | 1.67 |
| ZFR | zinc finger RNA binding protein | 33148_at | 2.42 | 6.81 |
| SVEP1 | sushi, von Willebrand factor type A, EGF and pentraxin domain containing 1 | 219552_at | 2.42 | 2.45 |
| DOCK5 | Dedicator of cytokinesis 5 | 230206_at | 2.42 | 1.25 |
| SCARA3 | scavenger receptor class A, member 3 | 223842_s_at | 2.42 | 2.06 |
| FXYD5 | FXYD domain containing ion transport regulator 5 | 224252_s_at | 2.42 | 0.33 |
| PACSIN2 | protein kinase C and casein kinase substrate in neurons 2 | 1554691_a_at | 2.42 | 0.44 |
| SENP3 | SUMO1/sentrin/SMT3 specific peptidase 3 | 203871_at | 2.42 | 0.35 |
| ARFIP1 | ADP-ribosylation factor interacting protein 1 | 214483_s_at | 2.42 | 1.00 |
| PGK1 | phosphoglycerate kinase 1 | 1558365_at | 2.42 | 3.21 |
| TGOLN2 | trans-golgi network protein 2 | 203834_s_at | 2.41 | 1.25 |
| PPM1A | protein phosphatase, Mg2+/Mn2+ dependent, 1A | 235344_at | 2.41 | 0.44 |
| STK4 | serine/threonine kinase 4 | 1569791_at | 2.41 | 6.81 |
| LOC147004 | hypothetical protein LOC147004 | 1564203_at | 2.41 | 7.84 |
| CSNK1D | casein kinase 1, delta | 207945_s_at | 2.41 | 0.35 |
| FAM3A | family with sequence similarity 3, member A | 209405_s_at | 2.41 | 0.86 |
| DSCR3 | Down syndrome critical region gene 3 | 217309_s_at | 2.41 | 1.44 |
| ATP7A | ATPase, Cu++ transporting, alpha polypeptide | 205197_s_at | 2.41 | 0.86 |
| MAP2K7 | mitogen-activated protein kinase kinase 7 | 226053_at | 2.41 | 0.33 |
| FAM102A | family with sequence similarity 102, member A | 212400_at | 2.41 | 0.86 |
| MUC3A | mucin 3A, cell surface associated | 217117_x_at | 2.41 | 0.63 |
| PDPN | podoplanin | 221898_at | 2.41 | 3.21 |
| FAM126A | family with sequence similarity 126, member A | 231396_s_at | 2.41 | 0.86 |
| LOC100507513 | hypothetical LOC100507513 | 237709_at | 2.41 | 2.72 |
| CMAH | cytidine monophosphate-N-acetylneuraminic acid hydroxylase (CMP-N-acetylneuraminate monooxygenase) pseudogene | 210571_s_at | 2.41 | 9.00 |
| PER1 | period homolog 1 (Drosophila) | 36829_at | 2.40 | 4.15 |
| PLD1 | phospholipase D1, phosphatidylcholine-specific | 215723_s_at | 2.40 | 6.81 |
| ETNK1 | ethanolamine kinase 1 | 224453_s_at | 2.40 | 2.06 |
| TRIM3 | tripartite motif-containing 3 | 213885_at | 2.40 | 1.25 |
| LRRC15 | leucine rich repeat containing 15 | 213909_at | 2.40 | 1.67 |
| KLF3 | Kruppel-like factor 3 (basic) | 225140_at | 2.40 | 1.00 |
| KLHL20 | kelch-like 20 (Drosophila) | 210635_s_at | 2.40 | 0.44 |
| MED12 | mediator complex subunit 12 | 216071_x_at | 2.40 | 0.44 |
| WDFY2 | WD repeat and FYVE domain containing 2 | 1560112_at | 2.40 | 0.86 |
| FLI1 | Friend leukemia virus integration 1 | 210786_s_at | 2.40 | 1.00 |
| WDTC1 | WD and tetratricopeptide repeats 1 | 40829_at | 2.40 | 0.14 |
| KRAS | v-Ki-ras2 Kirsten rat sarcoma viral oncogene homolog | 214352_s_at | 2.40 | 0.35 |
| ZFYVE1 | zinc finger, FYVE domain containing 1 | 223388_s_at | 2.40 | 0.55 |
| TYR | tyrosinase (oculocutaneous albinism IA) | 206630_at | 2.40 | 7.84 |
| OS9 | osteosarcoma amplified 9, endoplasmic reticulum lectin | 200714_x_at | 2.39 | 0.35 |
| FER1L4 | fer-1-like 4 (C. elegans) pseudogene | 222245_s_at | 2.39 | 2.45 |
| SLC6A10P /// SLC6A8 | solute carrier family 6 (neurotransmitter transporter, creatine), member 10 (pseudogene) /// solute carrier family 6 (neurotransmitter transporter, creatine), member 8 | 215812_s_at | 2.39 | 0.86 |
| KIAA2013 | KIAA2013 | 1555933_at | 2.39 | 1.67 |
| KCNK2 | potassium channel, subfamily K, member 2 | 210261_at | 2.39 | 1.67 |
| LMBR1 | limb region 1 homolog (mouse) | 224410_s_at | 2.39 | 2.72 |
| ASXL2 | additional sex combs like 2 (Drosophila) | 218659_at | 2.39 | 0.33 |
| RNF19B | ring finger protein 19B | 36564_at | 2.39 | 0.35 |
| MAPK14 | mitogen-activated protein kinase 14 | 211561_x_at | 2.39 | 0.44 |
| WDTC1 | WD and tetratricopeptide repeats 1 | 216036_x_at | 2.39 | 1.44 |
| DOCK5 | Dedicator of cytokinesis 5 | 230207_s_at | 2.39 | 1.67 |
| HMG20B | high-mobility group 20B | 209113_s_at | 2.39 | 0.55 |
| RBM22 | RNA binding motif protein 22 | 222527_s_at | 2.39 | 2.45 |
| ZNF282 | zinc finger protein 282 | 212892_at | 2.38 | 0.26 |
| RNF123 | ring finger protein 123 | 221063_x_at | 2.38 | 0.63 |
| ALDOC | aldolase C, fructose-bisphosphate | 202022_at | 2.38 | 4.15 |
| FAM126A | family with sequence similarity 126, member A | 223625_at | 2.38 | 1.25 |
| SOCS6 | suppressor of cytokine signaling 6 | 214462_at | 2.38 | 0.86 |
| CDH26 | cadherin 26 | 233391_at | 2.38 | 6.81 |
| LOC100133612 | similar to hCG1815312 | 1569604_at | 2.38 | 1.00 |
| LOC100507620 | hypothetical LOC100507620 | 211973_at | 2.38 | 0.35 |
| C9orf85 | chromosome 9 open reading frame 85 | 238579_at | 2.38 | 3.67 |
| DRG2 | developmentally regulated GTP binding protein 2 | 203267_s_at | 2.37 | 0.55 |
| SLC22A17 | solute carrier family 22, member 17 | 218675_at | 2.37 | 1.00 |
| SNAP25 | synaptosomal-associated protein, 25kDa | 202507_s_at | 2.37 | 0.86 |
| ASMTL-AS | ASMTL antisense RNA (non-protein coding) | 1552873_s_at | 2.37 | 0.63 |
| C1GALT1C1 | C1GALT1-specific chaperone 1 | 238989_at | 2.37 | 1.67 |
| BCAP29 | B-cell receptor-associated protein 29 | 217657_at | 2.37 | 2.72 |
| FSCN1 | fascin homolog 1, actin-bundling protein (Strongylocentrotus purpuratus) | 201564_s_at | 2.37 | 0.26 |
| NF1 | neurofibromin 1 | 212678_at | 2.37 | 0.33 |
| ZFYVE27 | zinc finger, FYVE domain containing 27 | 225218_at | 2.37 | 0.55 |
| SEPT11 | septin 11 | 201308_s_at | 2.37 | 1.25 |
| DGKA | diacylglycerol kinase, alpha 80kDa | 203385_at | 2.37 | 0.63 |
| CDCP1 | CUB domain containing protein 1 | 218451_at | 2.37 | 1.67 |
| ATXN7L1 | ataxin 7-like 1 | 227732_at | 2.37 | 1.25 |
| HDAC6 | histone deacetylase 6 | 206846_s_at | 2.37 | 0.55 |
| DLGAP4 | discs, large (Drosophila) homolog-associated protein 4 | 202571_s_at | 2.37 | 0.63 |
| PPP1R16A | protein phosphatase 1, regulatory (inhibitor) subunit 16A | 225203_at | 2.37 | 2.72 |
| ZNF160 | zinc finger protein 160 | 1567032_s_at | 2.37 | 0.63 |
| TMEM201 | transmembrane protein 201 | 228671_at | 2.37 | 1.00 |
| ARNT | Aryl hydrocarbon receptor nuclear translocator | 231016_s_at | 2.37 | 0.63 |
| FIZ1 | FLT3-interacting zinc finger 1 | 226967_at | 2.37 | 0.73 |
| G3BP2 | GTPase activating protein (SH3 domain) binding protein 2 | 208840_s_at | 2.36 | 1.44 |
| FAM168B | family with sequence similarity 168, member B | 229946_at | 2.36 | 0.73 |
| IGFBP5 | insulin-like growth factor binding protein 5 | 211958_at | 2.36 | 2.06 |
| EPHA4 | EPH receptor A4 | 228948_at | 2.36 | 1.44 |
| SPATA20 | spermatogenesis associated 20 | 218164_at | 2.36 | 0.55 |
| CHD8 | chromodomain helicase DNA binding protein 8 | 212571_at | 2.36 | 0.63 |
| KIAA0430 | KIAA0430 | 1558697_a_at | 2.36 | 9.00 |
| ITGB3 | integrin, beta 3 (platelet glycoprotein IIIa, antigen CD61) | 204627_s_at | 2.36 | 1.25 |
| HSD11B1L | hydroxysteroid (11-beta) dehydrogenase 1-like | 228757_at | 2.36 | 0.86 |
| EXOC7 | exocyst complex component 7 | 212035_s_at | 2.36 | 0.55 |
| SENP7 | SUMO1/sentrin specific peptidase 7 | 220735_s_at | 2.36 | 2.45 |
| KLHL22 | kelch-like 22 (Drosophila) | 221838_at | 2.36 | 0.55 |
| PSAP | prosaposin | 200866_s_at | 2.36 | 0.44 |
| RGMB | RGM domain family, member B | 227340_s_at | 2.36 | 1.25 |
| GPR108 | G protein-coupled receptor 108 | 225058_at | 2.36 | 0.19 |
| IL1A | interleukin 1, alpha | 208200_at | 2.36 | 2.06 |
| SH2D3A | SH2 domain containing 3A | 222169_x_at | 2.36 | 1.44 |
| LYNX1 | Ly6/neurotoxin 1 | 226305_at | 2.36 | 0.73 |
| MXD4 | MAX dimerization protein 4 | 212346_s_at | 2.35 | 3.21 |
| RANBP2 | RAN binding protein 2 | 201711_x_at | 2.35 | 1.25 |
| KALRN | kalirin, RhoGEF kinase | 206078_at | 2.35 | 3.21 |
| XPO7 | exportin 7 | 208459_s_at | 2.35 | 0.86 |
| SLC1A3 | solute carrier family 1 (glial high affinity glutamate transporter), member 3 | 202800_at | 2.35 | 2.06 |
| RASD1 | RAS, dexamethasone-induced 1 | 223467_at | 2.35 | 2.06 |
| C1orf38 | chromosome 1 open reading frame 38 | 210785_s_at | 2.35 | 1.67 |
| CCNE2 | cyclin E2 | 211814_s_at | 2.35 | 9.00 |
| RIT1 | Ras-like without CAAX 1 | 236223_s_at | 2.35 | 0.73 |
| PPP1R14C | protein phosphatase 1, regulatory (inhibitor) subunit 14C | 226907_at | 2.35 | 3.21 |
| SLC38A5 | solute carrier family 38, member 5 | 234973_at | 2.35 | 3.67 |
| PILRB | paired immunoglobin-like type 2 receptor beta | 220954_s_at | 2.35 | 2.72 |
| ELOVL5 | ELOVL family member 5, elongation of long chain fatty acids (FEN1/Elo2, SUR4/Elo3-like, yeast) | 215082_at | 2.35 | 3.21 |
| TMEM127 | transmembrane protein 127 | 222887_s_at | 2.35 | 2.45 |
| VASH1 | vasohibin 1 | 203940_s_at | 2.35 | 0.55 |
| FAM45A /// FAM45B | family with sequence similarity 45, member A /// family with sequence similarity 45, member A pseudogene | 222955_s_at | 2.35 | 0.63 |
| NRP2 | neuropilin 2 | 1555468_at | 2.35 | 0.63 |
| GAMT | guanidinoacetate N-methyltransferase | 1552474_a_at | 2.35 | 0.44 |
| TFPI | tissue factor pathway inhibitor (lipoprotein-associated coagulation inhibitor) | 209676_at | 2.35 | 3.21 |
| ABCF2 | ATP-binding cassette, sub-family F (GCN20), member 2 | 207622_s_at | 2.35 | 0.73 |
| NFIB | nuclear factor I/B | 211467_s_at | 2.34 | 4.15 |
| SS18 | synovial sarcoma translocation, chromosome 18 | 216684_s_at | 2.34 | 0.63 |
| KPNA5 | karyopherin alpha 5 (importin alpha 6) | 206241_at | 2.34 | 2.06 |
| LARP4B | La ribonucleoprotein domain family, member 4B | 228196_s_at | 2.34 | 1.00 |
| STIP1 | stress-induced-phosphoprotein 1 | 213330_s_at | 2.34 | 0.55 |
| GALNT6 | UDP-N-acetyl-alpha-D-galactosamine:polypeptide N-acetylgalactosaminyltransferase 6 (GalNAc-T6) | 219956_at | 2.34 | 3.67 |
| CCDC6 | coiled-coil domain containing 6 | 204716_at | 2.34 | 1.44 |
| MTFP1 | mitochondrial fission process 1 | 223172_s_at | 2.34 | 3.67 |
| EN1 | engrailed homeobox 1 | 220559_at | 2.34 | 1.44 |
| ZNF652 | zinc finger protein 652 | 205594_at | 2.33 | 2.06 |
| LOC440983 | Hypothetical gene supported by BC066916 | 227338_at | 2.33 | 2.45 |
| ALDH3B1 | aldehyde dehydrogenase 3 family, member B1 | 211004_s_at | 2.33 | 0.86 |
| GRLF1 | glucocorticoid receptor DNA binding factor 1 | 202044_at | 2.33 | 1.67 |
| POLR2E | polymerase (RNA) II (DNA directed) polypeptide E, 25kDa | 217854_s_at | 2.33 | 0.86 |
| GLYR1 | glyoxylate reductase 1 homolog (Arabidopsis) | 222115_x_at | 2.33 | 1.00 |
| C20orf200 | chromosome 20 open reading frame 200 | 1553205_at | 2.33 | 3.67 |
| ARID1A | AT rich interactive domain 1A (SWI-like) | 207591_s_at | 2.33 | 1.00 |
| HFE | hemochromatosis | 214647_s_at | 2.33 | 6.81 |
| GALC | galactosylceramidase | 211810_s_at | 2.33 | 0.86 |
| TPM3 | tropomyosin 3 | 224164_at | 2.33 | 4.93 |
| FNIP1 | folliculin interacting protein 1 | 228250_at | 2.33 | 2.06 |
| SHISA5 | shisa homolog 5 (Xenopus laevis) | 222986_s_at | 2.33 | 0.73 |
| UBR4 | ubiquitin protein ligase E3 component n-recognin 4 | 211950_at | 2.33 | 0.73 |
| CLASP1 | Cytoplasmic linker associated protein 1 | 240757_at | 2.33 | 0.86 |
| WSB1 | WD repeat and SOCS box-containing 1 | 201295_s_at | 2.33 | 1.25 |
| PACS1 | phosphofurin acidic cluster sorting protein 1 | 220557_s_at | 2.33 | 1.00 |
| KCTD10 | potassium channel tetramerisation domain containing 10 | 223208_at | 2.33 | 0.35 |
| ACTR3B /// LOC100290215 | ARP3 actin-related protein 3 homolog B (yeast) /// actin-related protein 3B-like | 1555487_a_at | 2.32 | 1.67 |
| WIPI2 | WD repeat domain, phosphoinositide interacting 2 | 1570033_at | 2.32 | 7.84 |
| PELO | pelota homolog (Drosophila) | 1553140_at | 2.32 | 3.21 |
| FSTL3 | follistatin-like 3 (secreted glycoprotein) | 203592_s_at | 2.32 | 3.67 |
| API5 | apoptosis inhibitor 5 | 214959_s_at | 2.32 | 1.67 |
| DDX11 | DEAD/H (Asp-Glu-Ala-Asp/His) box polypeptide 11 | 208149_x_at | 2.32 | 0.86 |
| C14orf101 | chromosome 14 open reading frame 101 | 219757_s_at | 2.32 | 0.35 |
| LOC149086 | ornithine decarboxylase 1 pseudogene | 1566646_at | 2.32 | 7.84 |
| MAX | MYC associated factor X | 208403_x_at | 2.32 | 3.21 |
| ME2 | malic enzyme 2, NAD(+)-dependent, mitochondrial | 210154_at | 2.32 | 0.63 |
| EHD2 | EH-domain containing 2 | 221870_at | 2.32 | 0.86 |
| SKAP2 | Src kinase associated phosphoprotein 2 | 241074_at | 2.32 | 3.67 |
| N4BP2L1 | NEDD4 binding protein 2-like 1 | 214906_x_at | 2.32 | 1.67 |
| C21orf56 | chromosome 21 open reading frame 56 | 223360_at | 2.32 | 0.73 |
| C16orf57 | chromosome 16 open reading frame 57 | 218060_s_at | 2.32 | 1.25 |
| CSNK2A1 | casein kinase 2, alpha 1 polypeptide | 206075_s_at | 2.32 | 1.00 |
| HAPLN3 | hyaluronan and proteoglycan link protein 3 | 227262_at | 2.31 | 1.44 |
| RXRA | retinoid X receptor, alpha | 202426_s_at | 2.31 | 1.00 |
| PTPN23 | protein tyrosine phosphatase, non-receptor type 23 | 223150_s_at | 2.31 | 0.35 |
| C16orf58 | chromosome 16 open reading frame 58 | 222190_s_at | 2.31 | 0.26 |
| GTDC1 | glycosyltransferase-like domain containing 1 | 238585_at | 2.31 | 0.86 |
| PIK3IP1 | phosphoinositide-3-kinase interacting protein 1 | 221757_at | 2.31 | 5.82 |
| DDX17 | DEAD (Asp-Glu-Ala-Asp) box polypeptide 17 | 208719_s_at | 2.31 | 6.81 |
| ATOH8 | Atonal homolog 8 (Drosophila) | 1558705_at | 2.31 | 4.93 |
| CTDP1 | CTD (carboxy-terminal domain, RNA polymerase II, polypeptide A) phosphatase, subunit 1 | 205035_at | 2.31 | 0.55 |
| KIF2C | kinesin family member 2C | 211519_s_at | 2.31 | 1.00 |
| HLA-G | major histocompatibility complex, class I, G | 211530_x_at | 2.31 | 3.21 |
| SOD2 | superoxide dismutase 2, mitochondrial | 215223_s_at | 2.31 | 2.06 |
| LOC100289058 | Hypothetical protein LOC100289058 | 1563077_at | 2.31 | 2.45 |
| CYHR1 | cysteine/histidine-rich 1 | 213681_at | 2.31 | 1.67 |
| NACC1 | nucleus accumbens associated 1, BEN and BTB (POZ) domain containing | 227651_at | 2.31 | 0.35 |
| MYOCD | myocardin | 229339_at | 2.31 | 1.44 |
| POLRMT | polymerase (RNA) mitochondrial (DNA directed) | 203782_s_at | 2.31 | 0.73 |
| TRAK1 | trafficking protein, kinesin binding 1 | 201283_s_at | 2.30 | 2.45 |
| HNRNPM | Heterogeneous nuclear ribonucleoprotein M | 1555843_at | 2.30 | 4.93 |
| TMEM64 | transmembrane protein 64 | 242338_at | 2.30 | 1.25 |
| PAPOLA | poly(A) polymerase alpha | 212720_at | 2.30 | 1.44 |
| SATB2 | SATB homeobox 2 | 215591_at | 2.30 | 2.72 |
| CTSB | cathepsin B | 213274_s_at | 2.30 | 0.86 |
| DHPS | deoxyhypusine synthase | 202802_at | 2.30 | 0.35 |
| RASA2 | RAS p21 protein activator 2 | 230669_at | 2.30 | 0.44 |
| MAPK1 | mitogen-activated protein kinase 1 | 1552264_a_at | 2.30 | 1.00 |
| COL12A1 | collagen, type XII, alpha 1 | 231879_at | 2.30 | 1.25 |
| ZFYVE28 | zinc finger, FYVE domain containing 28 | 232408_at | 2.30 | 2.72 |
| PARP3 | poly (ADP-ribose) polymerase family, member 3 | 209940_at | 2.29 | 0.73 |
| EBF4 | early B-cell factor 4 | 233850_s_at | 2.29 | 3.21 |
| CAPN1 | calpain 1, (mu/I) large subunit | 200752_s_at | 2.29 | 1.67 |
| DNAL1 | dynein, axonemal, light chain 1 | 223959_at | 2.29 | 2.45 |
| ALDH18A1 | aldehyde dehydrogenase 18 family, member A1 | 217791_s_at | 2.29 | 0.44 |
| ISLR | immunoglobulin superfamily containing leucine-rich repeat | 207191_s_at | 2.29 | 2.45 |
| STAT5B | signal transducer and activator of transcription 5B | 205026_at | 2.29 | 0.86 |
| LEMD2 | LEM domain containing 2 | 224980_at | 2.29 | 0.63 |
| SASH1 | SAM and SH3 domain containing 1 | 213236_at | 2.29 | 0.26 |
| FAM89B | family with sequence similarity 89, member B | 212484_at | 2.29 | 0.86 |
| POLR3G | polymerase (RNA) III (DNA directed) polypeptide G (32kD) | 206653_at | 2.29 | 1.25 |
| CMIP | c-Maf-inducing protein | 224991_at | 2.29 | 0.73 |
| RNF126 | ring finger protein 126 | 223332_x_at | 2.29 | 1.00 |
| ZNF526 | zinc finger protein 526 | 228229_at | 2.29 | 0.73 |
| NRIP1 | nuclear receptor interacting protein 1 | 202600_s_at | 2.29 | 0.73 |
| B3GAT3 | beta-1,3-glucuronyltransferase 3 (glucuronosyltransferase I) | 35179_at | 2.29 | 1.00 |
| RPS23 | ribosomal protein S23 | 227722_at | 2.29 | 6.81 |
| GGA3 | golgi-associated, gamma adaptin ear containing, ARF binding protein 3 | 211815_s_at | 2.29 | 0.86 |
| ARSD | arylsulfatase D | 232423_at | 2.29 | 2.72 |
| USP21 | ubiquitin specific peptidase 21 | 218367_x_at | 2.29 | 0.63 |
| SIK3 | SIK family kinase 3 | 204156_at | 2.29 | 1.44 |
| BAT2 | HLA-B associated transcript 2 | 208132_x_at | 2.29 | 0.55 |
| PHLPP1 | PH domain and leucine rich repeat protein phosphatase 1 | 212719_at | 2.29 | 0.63 |
| PRPF31 | PRP31 pre-mRNA processing factor 31 homolog (S. cerevisiae) | 202407_s_at | 2.29 | 0.86 |
| ZNF384 | zinc finger protein 384 | 212369_at | 2.29 | 0.55 |
| SLFN5 | schlafen family member 5 | 243999_at | 2.29 | 1.44 |
| CLINT1 | clathrin interactor 1 | 201768_s_at | 2.29 | 0.44 |
| HLA-E | major histocompatibility complex, class I, E | 200904_at | 2.29 | 1.00 |
| SYNPO2 | synaptopodin 2 | 225894_at | 2.29 | 6.81 |
| NAT14 | N-acetyltransferase 14 (GCN5-related, putative) | 223284_at | 2.29 | 0.86 |
| FAM3A | family with sequence similarity 3, member A | 38043_at | 2.29 | 1.25 |
| POMZP3 /// ZP3 | POM121 and ZP3 fusion /// zona pellucida glycoprotein 3 (sperm receptor) | 204148_s_at | 2.29 | 3.21 |
| NLRP1 | NLR family, pyrin domain containing 1 | 211824_x_at | 2.28 | 1.67 |
| PVT1 | Pvt1 oncogene (non-protein coding) | 1558290_a_at | 2.28 | 2.06 |
| C1orf38 | chromosome 1 open reading frame 38 | 207571_x_at | 2.28 | 1.25 |
| TMEM63A | transmembrane protein 63A | 214833_at | 2.28 | 1.25 |
| MYPOP | Myb-related transcription factor, partner of profilin | 227937_at | 2.28 | 0.86 |
| C10orf103 | chromosome 10 open reading frame 103 | 1557827_at | 2.28 | 6.81 |
| CREB1 | cAMP responsive element binding protein 1 | 214513_s_at | 2.28 | 1.44 |
| PITPNC1 | phosphatidylinositol transfer protein, cytoplasmic 1 | 238649_at | 2.28 | 9.00 |
| PPP2R5B | protein phosphatase 2, regulatory subunit B', beta | 635_s_at | 2.28 | 2.06 |
| LIF | leukemia inhibitory factor (cholinergic differentiation factor) | 205266_at | 2.28 | 4.15 |
| LRRC32 | leucine rich repeat containing 32 | 203835_at | 2.28 | 2.72 |
| SHC4 | SHC (Src homology 2 domain containing) family, member 4 | 230538_at | 2.28 | 5.82 |
| MOGS | mannosyl-oligosaccharide glucosidase | 210627_s_at | 2.27 | 0.73 |
| CCDC91 | Coiled-coil domain containing 91 | 1570571_at | 2.27 | 2.45 |
| PAFAH1B1 | platelet-activating factor acetylhydrolase 1b, regulatory subunit 1 (45kDa) | 211547_s_at | 2.27 | 1.00 |
| LOC100506696 | hypothetical LOC100506696 | 231233_at | 2.27 | 2.45 |
| MED12 | mediator complex subunit 12 | 203506_s_at | 2.27 | 0.86 |
| HSP90AB1 | heat shock protein 90kDa alpha (cytosolic), class B member 1 | 214359_s_at | 2.27 | 0.73 |
| TNRC18 | trinucleotide repeat containing 18 | 229257_at | 2.27 | 2.06 |
| KIAA0467 | KIAA0467 | 215364_s_at | 2.27 | 0.63 |
| GLUL | glutamate-ammonia ligase | 200648_s_at | 2.27 | 3.21 |
| RHBDD2 | rhomboid domain containing 2 | 222995_s_at | 2.27 | 0.63 |
| DCTN4 | dynactin 4 (p62) | 218013_x_at | 2.27 | 1.67 |
| CYFIP2 | cytoplasmic FMR1 interacting protein 2 | 215785_s_at | 2.27 | 4.15 |
| ATL2 | atlastin GTPase 2 | 1553603_s_at | 2.27 | 1.25 |
| GALNT2 | UDP-N-acetyl-alpha-D-galactosamine:polypeptide N-acetylgalactosaminyltransferase 2 (GalNAc-T2) | 217787_s_at | 2.27 | 2.06 |
| MUL1 | mitochondrial E3 ubiquitin protein ligase 1 | 218246_at | 2.27 | 1.44 |
| FBXL18 | F-box and leucine-rich repeat protein 18 | 215068_s_at | 2.26 | 1.25 |
| RIN3 | Ras and Rab interactor 3 | 219457_s_at | 2.26 | 1.44 |
| MAP2K3 | mitogen-activated protein kinase kinase 3 | 207667_s_at | 2.26 | 1.00 |
| NCSTN | nicastrin | 208759_at | 2.26 | 0.73 |
| C20orf27 | chromosome 20 open reading frame 27 | 50314_i_at | 2.26 | 0.86 |
| MGRN1 | mahogunin, ring finger 1 | 212576_at | 2.26 | 0.86 |
| TUBGCP3 | tubulin, gamma complex associated protein 3 | 1554086_at | 2.26 | 2.06 |
| ING5 | inhibitor of growth family, member 5 | 223871_x_at | 2.26 | 2.72 |
| FGFR1 | fibroblast growth factor receptor 1 | 207937_x_at | 2.26 | 1.00 |
| TOB2 | transducer of ERBB2, 2 | 221496_s_at | 2.26 | 1.44 |
| APEX2 | APEX nuclease (apurinic/apyrimidinic endonuclease) 2 | 204408_at | 2.26 | 0.86 |
| ZBTB7A | zinc finger and BTB domain containing 7A | 219186_at | 2.26 | 1.00 |
| ANKRD32 | ankyrin repeat domain 32 | 1558076_at | 2.26 | 2.72 |
| CDKN1A | cyclin-dependent kinase inhibitor 1A (p21, Cip1) | 202284_s_at | 2.26 | 1.00 |
| SH3GL1 | SH3-domain GRB2-like 1 | 201851_at | 2.26 | 0.44 |
| TLE2 | transducin-like enhancer of split 2 (E(sp1) homolog, Drosophila) | 40837_at | 2.26 | 2.06 |
| CORO1B | coronin, actin binding protein, 1B | 221754_s_at | 2.26 | 4.93 |
| PXN | paxillin | 201087_at | 2.26 | 0.35 |
| ATM | ataxia telangiectasia mutated | 208442_s_at | 2.25 | 1.00 |
| C16orf63 | chromosome 16 open reading frame 63 | 225088_at | 2.25 | 3.67 |
| OR2A20P /// OR2A9P | olfactory receptor, family 2, subfamily A, member 20 pseudogene /// olfactory receptor, family 2, subfamily A, member 9 pseudogene | 223971_at | 2.25 | 4.15 |
| KIAA1147 | KIAA1147 | 223161_at | 2.25 | 1.44 |
| AIG1 | androgen-induced 1 | 232810_at | 2.25 | 1.44 |
| TOR3A | torsin family 3, member A | 233851_s_at | 2.25 | 1.00 |
| C7orf58 | chromosome 7 open reading frame 58 | 220032_at | 2.25 | 3.67 |
| LANCL1 | LanC lantibiotic synthetase component C-like 1 (bacterial) | 202019_s_at | 2.25 | 2.72 |
| LOC728743 | similar to GLI-Kruppel family member HKR1 | 225909_at | 2.25 | 1.25 |
| STAT3 | signal transducer and activator of transcription 3 (acute-phase response factor) | 208992_s_at | 2.25 | 1.00 |
| SLC4A7 | solute carrier family 4, sodium bicarbonate cotransporter, member 7 | 207604_s_at | 2.25 | 2.72 |
| NARG2 | NMDA receptor regulated 2 | 235189_at | 2.25 | 7.84 |
| ASAP1 | ArfGAP with SH3 domain, ankyrin repeat and PH domain 1 | 224790_at | 2.25 | 1.00 |
| NDRG3 | NDRG family member 3 | 221082_s_at | 2.25 | 1.00 |
| ZNF79 | zinc finger protein 79 | 216486_x_at | 2.25 | 1.67 |
| DOT1L | DOT1-like, histone H3 methyltransferase (S. cerevisiae) | 226201_at | 2.25 | 4.15 |
| ZBTB7A | zinc finger and BTB domain containing 7A | 213303_x_at | 2.25 | 0.44 |
| GPM6B | glycoprotein M6B | 209170_s_at | 2.25 | 2.72 |
| MLEC | malectin | 200617_at | 2.25 | 0.44 |
| PTCRA | pre T-cell antigen receptor alpha | 215492_x_at | 2.25 | 1.00 |
| LTBP4 | latent transforming growth factor beta binding protein 4 | 210628_x_at | 2.25 | 0.86 |
| OSBPL5 | oxysterol binding protein-like 5 | 233734_s_at | 2.25 | 0.44 |
| RWDD2B | RWD domain containing 2B | 222614_at | 2.25 | 0.86 |
| CFLAR | CASP8 and FADD-like apoptosis regulator | 211862_x_at | 2.25 | 1.25 |
| DCAF8 | DDB1 and CUL4 associated factor 8 | 233637_at | 2.24 | 3.21 |
| CLIP3 | CAP-GLY domain containing linker protein 3 | 212358_at | 2.24 | 0.86 |
| DDX19A | DEAD (Asp-Glu-Ala-As) box polypeptide 19A | 202578_s_at | 2.24 | 1.00 |
| DCAF8 | DDB1 and CUL4 associated factor 8 | 202249_s_at | 2.24 | 1.44 |
| TOX2 | TOX high mobility group box family member 2 | 228737_at | 2.24 | 2.72 |
| USP10 | ubiquitin specific peptidase 10 | 209136_s_at | 2.24 | 1.00 |
| BET1L | blocked early in transport 1 homolog (S. cerevisiae)-like | 220470_at | 2.24 | 0.55 |
| STX16 | syntaxin 16 | 221499_s_at | 2.24 | 0.86 |
| STIM1 | stromal interaction molecule 1 | 202764_at | 2.24 | 1.00 |
| GPR124 | G protein-coupled receptor 124 | 221814_at | 2.24 | 0.63 |
| KCNG1 | potassium voltage-gated channel, subfamily G, member 1 | 214595_at | 2.24 | 3.67 |
| TOLLIP | toll interacting protein | 233881_s_at | 2.24 | 4.93 |
| FAM127B | family with sequence similarity 127, member B | 217948_at | 2.24 | 0.86 |
| SOD3 | superoxide dismutase 3, extracellular | 205236_x_at | 2.24 | 4.93 |
| SLC7A1 | solute carrier family 7 (cationic amino acid transporter, y+ system), member 1 | 212292_at | 2.24 | 0.86 |
| NEDD9 | neural precursor cell expressed, developmentally down-regulated 9 | 202150_s_at | 2.23 | 2.06 |
| CLIP4 | CAP-GLY domain containing linker protein family, member 4 | 219944_at | 2.23 | 0.86 |
| SRGAP1 | SLIT-ROBO Rho GTPase activating protein 1 | 233888_s_at | 2.23 | 0.63 |
| POR | P450 (cytochrome) oxidoreductase | 208928_at | 2.23 | 7.84 |
| POLD2 | polymerase (DNA directed), delta 2, regulatory subunit 50kDa | 201115_at | 2.23 | 0.44 |
| SQSTM1 | sequestosome 1 | 213112_s_at | 2.23 | 7.84 |
| SPRYD3 | SPRY domain containing 3 | 225134_at | 2.23 | 0.55 |
| MAFF | v-maf musculoaponeurotic fibrosarcoma oncogene homolog F (avian) | 205193_at | 2.23 | 1.44 |
| ATP6V0E2 | ATPase, H+ transporting V0 subunit e2 | 213587_s_at | 2.23 | 0.86 |
| PAWR | PRKC, apoptosis, WT1, regulator | 214237_x_at | 2.23 | 2.72 |
| TFE3 | transcription factor binding to IGHM enhancer 3 | 206649_s_at | 2.23 | 0.63 |
| SGMS2 | sphingomyelin synthase 2 | 243141_at | 2.23 | 3.21 |
| GPI | glucose-6-phosphate isomerase | 208308_s_at | 2.23 | 1.44 |
| RAB23 | RAB23, member RAS oncogene family | 220955_x_at | 2.23 | 1.44 |
| SOX4 | SRY (sex determining region Y)-box 4 | 201418_s_at | 2.23 | 2.72 |
| HLA-DRB1 /// HLA-DRB4 /// HLA-DRB5 | major histocompatibility complex, class II, DR beta 1 /// major histocompatibility complex, class II, DR beta 4 /// major histocompatibility complex, class II, DR beta 5 | 209312_x_at | 2.22 | 9.00 |
| LAMA4 | laminin, alpha 4 | 210990_s_at | 2.22 | 2.06 |
| EML3 | echinoderm microtubule associated protein like 3 | 203442_x_at | 2.22 | 0.44 |
| C16orf13 | chromosome 16 open reading frame 13 | 227378_x_at | 2.22 | 1.00 |
| THRA | thyroid hormone receptor, alpha (erythroblastic leukemia viral (v-erb-a) oncogene homolog, avian) | 35846_at | 2.22 | 1.25 |
| RANBP9 | RAN binding protein 9 | 216125_s_at | 2.22 | 1.00 |
| CFLAR | CASP8 and FADD-like apoptosis regulator | 208485_x_at | 2.22 | 1.25 |
| LCN12 | lipocalin 12 | 230717_at | 2.22 | 0.73 |
| CUX1 | cut-like homeobox 1 | 202367_at | 2.22 | 0.73 |
| KCNJ15 | potassium inwardly-rectifying channel, subfamily J, member 15 | 211806_s_at | 2.22 | 2.45 |
| INF2 | inverted formin, FH2 and WH2 domain containing | 218144_s_at | 2.22 | 1.00 |
| SLC27A1 | solute carrier family 27 (fatty acid transporter), member 1 | 226728_at | 2.22 | 2.06 |
| RAB3B | RAB3B, member RAS oncogene family | 205925_s_at | 2.22 | 7.84 |
| NLGN2 | neuroligin 2 | 1554428_s_at | 2.22 | 2.72 |
| ENSA | endosulfine alpha | 221487_s_at | 2.22 | 2.45 |
| PTPRU | protein tyrosine phosphatase, receptor type, U | 211320_s_at | 2.22 | 1.00 |
| SOS2 | son of sevenless homolog 2 (Drosophila) | 217576_x_at | 2.21 | 1.25 |
| MAP3K11 | mitogen-activated protein kinase kinase kinase 11 | 203652_at | 2.21 | 1.67 |
| CDK2 | cyclin-dependent kinase 2 | 211804_s_at | 2.21 | 2.72 |
| CDC42EP5 | CDC42 effector protein (Rho GTPase binding) 5 | 227850_x_at | 2.21 | 1.44 |
| KIF6 | kinesin family member 6 | 1556232_at | 2.21 | 3.21 |
| PRKACB | protein kinase, cAMP-dependent, catalytic, beta | 202742_s_at | 2.21 | 2.45 |
| SNAP23 | synaptosomal-associated protein, 23kDa | 214544_s_at | 2.21 | 1.44 |
| E2F7 | E2F transcription factor 7 | 241725_at | 2.21 | 0.86 |
| RUNX3 | runt-related transcription factor 3 | 204198_s_at | 2.21 | 1.67 |
| AOX1 | aldehyde oxidase 1 | 205082_s_at | 2.21 | 6.81 |
| HERC4 | hect domain and RLD 4 | 208055_s_at | 2.21 | 4.93 |
| NRP2 | neuropilin 2 | 229225_at | 2.21 | 1.25 |
| ATXN2 | ataxin 2 | 202622_s_at | 2.21 | 0.73 |
| FLII | flightless I homolog (Drosophila) | 212025_s_at | 2.21 | 1.44 |
| PFN1 | profilin 1 | 200634_at | 2.21 | 0.55 |
| JMJD6 | jumonji domain containing 6 | 215233_at | 2.21 | 3.67 |
| DDHD1 | DDHD domain containing 1 | 244154_at | 2.21 | 4.15 |
| SORD | sorbitol dehydrogenase | 201562_s_at | 2.21 | 1.00 |
| SNRPA | small nuclear ribonucleoprotein polypeptide A | 201770_at | 2.21 | 0.86 |
| ZNF160 | zinc finger protein 160 | 224014_at | 2.21 | 2.45 |
| C16orf5 | chromosome 16 open reading frame 5 | 223960_s_at | 2.21 | 1.44 |
| ARHGEF2 | Rho/Rac guanine nucleotide exchange factor (GEF) 2 | 207629_s_at | 2.21 | 0.86 |
| SPPL2B | signal peptide peptidase-like 2B | 225248_at | 2.21 | 1.00 |
| LEPR | leptin receptor | 211354_s_at | 2.21 | 7.84 |
| HLA-G | major histocompatibility complex, class I, G | 210514_x_at | 2.21 | 1.44 |
| TRIM23 | tripartite motif-containing 23 | 210995_s_at | 2.21 | 0.86 |
| GFRA1 | GDNF family receptor alpha 1 | 205696_s_at | 2.20 | 2.06 |
| MAML2 | mastermind-like 2 (Drosophila) | 235106_at | 2.20 | 1.44 |
| DNAJC4 | DnaJ (Hsp40) homolog, subfamily C, member 4 | 206782_s_at | 2.20 | 0.86 |
| VPS39 | vacuolar protein sorting 39 homolog (S. cerevisiae) | 212156_at | 2.20 | 3.21 |
| MIA3 | melanoma inhibitory activity family, member 3 | 212305_s_at | 2.20 | 1.00 |
| FAM160A1 | family with sequence similarity 160, member A1 | 242687_at | 2.20 | 9.00 |
| TP63 | tumor protein p63 | 209863_s_at | 2.20 | 3.21 |
| ASNA1 | arsA arsenite transporter, ATP-binding, homolog 1 (bacterial) | 202024_at | 2.20 | 0.86 |
| PGD | phosphogluconate dehydrogenase | 201118_at | 2.20 | 1.25 |
| ZBTB38 | zinc finger and BTB domain containing 38 | 1558733_at | 2.20 | 3.21 |
| CABIN1 | calcineurin binding protein 1 | 37652_at | 2.20 | 1.25 |
| MLL4 | myeloid/lymphoid or mixed-lineage leukemia 4 | 203419_at | 2.20 | 2.45 |
| VAMP3 | vesicle-associated membrane protein 3 (cellubrevin) | 211749_s_at | 2.20 | 1.25 |
| SPRY4 | sprouty homolog 4 (Drosophila) | 221489_s_at | 2.20 | 2.72 |
| NPRL3 | nitrogen permease regulator-like 3 (S. cerevisiae) | 214273_x_at | 2.20 | 2.45 |
| GNB1L | guanine nucleotide binding protein (G protein), beta polypeptide 1-like | 220762_s_at | 2.20 | 1.25 |
| ACSF2 | acyl-CoA synthetase family member 2 | 218844_at | 2.20 | 1.25 |
| SLC35C2 | solute carrier family 35, member C2 | 219447_s_at | 2.20 | 0.63 |
| ICA1L | islet cell autoantigen 1,69kDa-like | 1554205_s_at | 2.19 | 3.67 |
| SH3BGRL3 | SH3 domain binding glutamic acid-rich protein like 3 | 221269_s_at | 2.19 | 1.25 |
| MLL | myeloid/lymphoid or mixed-lineage leukemia (trithorax homolog, Drosophila) | 212080_at | 2.19 | 1.25 |
| PTGIS | prostaglandin I2 (prostacyclin) synthase | 210702_s_at | 2.19 | 9.00 |
| TMTC4 | transmembrane and tetratricopeptide repeat containing 4 | 1554102_a_at | 2.19 | 1.00 |
| EHD2 | EH-domain containing 2 | 45297_at | 2.19 | 1.00 |
| ABI2 | abl-interactor 2 | 207268_x_at | 2.19 | 1.00 |
| CNPY3 | canopy 3 homolog (zebrafish) | 1552977_a_at | 2.19 | 0.63 |
| USP6 | ubiquitin specific peptidase 6 (Tre-2 oncogene) | 1555063_at | 2.19 | 1.67 |
| DVL2 | dishevelled, dsh homolog 2 (Drosophila) | 218759_at | 2.19 | 1.25 |
| PGPEP1 | pyroglutamyl-peptidase I | 223469_at | 2.19 | 0.63 |
| HOXC11 | homeobox C11 | 206745_at | 2.19 | 1.44 |
| USO1 | USO1 vesicle docking protein homolog (yeast) | 201831_s_at | 2.19 | 1.44 |
| UTRN | utrophin | 213022_s_at | 2.19 | 7.84 |
| IL6ST | interleukin 6 signal transducer (gp130, oncostatin M receptor) | 212196_at | 2.19 | 2.72 |
| PRPF4B | PRP4 pre-mRNA processing factor 4 homolog B (yeast) | 211090_s_at | 2.19 | 2.45 |
| ROD1 | ROD1 regulator of differentiation 1 (S. pombe) | 207223_s_at | 2.19 | 0.86 |
| FADS3 | fatty acid desaturase 3 | 216080_s_at | 2.19 | 0.73 |
| ITGB8 | integrin, beta 8 | 211488_s_at | 2.18 | 2.45 |
| DAB2 | disabled homolog 2, mitogen-responsive phosphoprotein (Drosophila) | 201279_s_at | 2.18 | 0.86 |
| BTG1 | B-cell translocation gene 1, anti-proliferative | 200920_s_at | 2.18 | 2.45 |
| LOC646762 | hypothetical LOC646762 | 1560006_a_at | 2.18 | 2.06 |
| MAP4 | microtubule-associated protein 4 | 200836_s_at | 2.18 | 1.00 |
| NCDN | neurochondrin | 209556_at | 2.18 | 0.63 |
| NOD1 | nucleotide-binding oligomerization domain containing 1 | 224190_x_at | 2.18 | 2.06 |
| PLOD3 | procollagen-lysine, 2-oxoglutarate 5-dioxygenase 3 | 202185_at | 2.18 | 0.55 |
| HIPK3 | homeodomain interacting protein kinase 3 | 210148_at | 2.18 | 2.45 |
| SEC16B | SEC16 homolog B (S. cerevisiae) | 1555289_at | 2.18 | 4.15 |
| SETD3 | SET domain containing 3 | 229940_at | 2.18 | 2.45 |
| CD44 | CD44 molecule (Indian blood group) | 1557905_s_at | 2.18 | 1.00 |
| TJP1 | tight junction protein 1 (zona occludens 1) | 214168_s_at | 2.18 | 1.67 |
| MAX | MYC associated factor X | 210734_x_at | 2.18 | 2.45 |
| GATSL3 /// TBC1D10A | GATS protein-like 3 /// TBC1 domain family, member 10A | 233528_s_at | 2.18 | 1.25 |
| LRRC41 | leucine rich repeat containing 41 | 201932_at | 2.17 | 0.63 |
| PEX6 | peroxisomal biogenesis factor 6 | 320_at | 2.17 | 3.67 |
| MARCH9 | membrane-associated ring finger (C3HC4) 9 | 226454_at | 2.17 | 3.67 |
| MEN1 | multiple endocrine neoplasia I | 202645_s_at | 2.17 | 0.63 |
| CYS1 | cystin 1 | 228739_at | 2.17 | 9.00 |
| ACOT8 | acyl-CoA thioesterase 8 | 204212_at | 2.17 | 0.63 |
| C7orf64 | chromosome 7 open reading frame 64 | 232661_s_at | 2.17 | 1.67 |
| DEDD | death effector domain containing | 202480_s_at | 2.17 | 0.63 |
| OASL | 2'-5'-oligoadenylate synthetase-like | 210797_s_at | 2.17 | 4.15 |
| LRSAM1 | leucine rich repeat and sterile alpha motif containing 1 | 227675_at | 2.17 | 0.86 |
| NEURL4 | neuralized homolog 4 (Drosophila) | 226703_at | 2.17 | 1.44 |
| IGHA1 | Immunoglobulin heavy constant alpha 1 | 211693_at | 2.17 | 1.67 |
| SYNPO | synaptopodin | 202796_at | 2.17 | 1.67 |
| GRAMD1A | GRAM domain containing 1A | 224807_at | 2.17 | 1.25 |
| FASTK | Fas-activated serine/threonine kinase | 210975_x_at | 2.17 | 0.55 |
| MFN1 | mitofusin 1 | 211801_x_at | 2.17 | 2.06 |
| PTK2 | PTK2 protein tyrosine kinase 2 | 207821_s_at | 2.17 | 0.73 |
| CADM1 | cell adhesion molecule 1 | 209031_at | 2.17 | 2.72 |
| C17orf80 | chromosome 17 open reading frame 80 | 223352_s_at | 2.17 | 1.25 |
| RIT1 | Ras-like without CAAX 1 | 236224_at | 2.17 | 1.25 |
| NFATC4 | nuclear factor of activated T-cells, cytoplasmic, calcineurin-dependent 4 | 213345_at | 2.16 | 2.45 |
| CCNG2 | cyclin G2 | 211559_s_at | 2.16 | 1.67 |
| EPSTI1 | epithelial stromal interaction 1 (breast) | 227609_at | 2.16 | 3.21 |
| GM2A | GM2 ganglioside activator | 235678_at | 2.16 | 4.15 |
| NIPAL1 | NIPA-like domain containing 1 | 1562792_at | 2.16 | 2.72 |
| PRIM2 | primase, DNA, polypeptide 2 (58kDa) | 1554885_a_at | 2.16 | 4.15 |
| UHMK1 | U2AF homology motif (UHM) kinase 1 | 227740_at | 2.16 | 1.00 |
| TTLL3 | tubulin tyrosine ligase-like family, member 3 | 210129_s_at | 2.16 | 2.06 |
| FARP1 | FERM, RhoGEF (ARHGEF) and pleckstrin domain protein 1 (chondrocyte-derived) | 201911_s_at | 2.16 | 1.00 |
| HFE | hemochromatosis | 206086_x_at | 2.16 | 1.44 |
| FCGRT | Fc fragment of IgG, receptor, transporter, alpha | 218831_s_at | 2.16 | 1.44 |
| PDPR | pyruvate dehydrogenase phosphatase regulatory subunit | 220236_at | 2.16 | 3.21 |
| POMZP3 | POM121 and ZP3 fusion | 210910_s_at | 2.16 | 2.72 |
| LOC100505894 | hypothetical LOC100505894 | 229815_at | 2.16 | 1.67 |
| FLOT1 | flotillin 1 | 210142_x_at | 2.16 | 0.73 |
| C10orf54 | chromosome 10 open reading frame 54 | 225373_at | 2.16 | 2.45 |
| KLF2 | Kruppel-like factor 2 (lung) | 226646_at | 2.16 | 1.67 |
| INO80 | INO80 homolog (S. cerevisiae) | 225357_s_at | 2.16 | 0.63 |
| BYSL | bystin-like | 203612_at | 2.16 | 1.00 |
| ASXL1 | additional sex combs like 1 (Drosophila) | 212238_at | 2.15 | 0.86 |
| IFI44L | interferon-induced protein 44-like | 204439_at | 2.15 | 6.81 |
| USP21 | ubiquitin specific peptidase 21 | 232219_x_at | 2.15 | 0.63 |
| AGPAT1 | 1-acylglycerol-3-phosphate O-acyltransferase 1 (lysophosphatidic acid acyltransferase, alpha) | 32836_at | 2.15 | 0.55 |
| NALCN | sodium leak channel, non-selective | 242880_at | 2.15 | 1.67 |
| DEAF1 | deformed epidermal autoregulatory factor 1 (Drosophila) | 209407_s_at | 2.15 | 0.86 |
| CAT | catalase | 211922_s_at | 2.15 | 2.72 |
| PPP2R4 | protein phosphatase 2A activator, regulatory subunit 4 | 206452_x_at | 2.15 | 1.44 |
| SNCAIP | synuclein, alpha interacting protein | 237833_s_at | 2.15 | 2.45 |
| ST7 | suppression of tumorigenicity 7 | 207524_at | 2.15 | 0.86 |
| ATXN2L | ataxin 2-like | 207798_s_at | 2.15 | 4.93 |
| MGEA5 | meningioma expressed antigen 5 (hyaluronidase) | 200898_s_at | 2.15 | 1.00 |
| LINS | lines homolog (Drosophila) | 231976_at | 2.15 | 1.25 |
| ZXDC | ZXD family zinc finger C | 230106_at | 2.15 | 1.00 |
| NT5E | 5'-nucleotidase, ecto (CD73) | 1553995_a_at | 2.15 | 1.67 |
| MLL2 | myeloid/lymphoid or mixed-lineage leukemia 2 | 227528_s_at | 2.15 | 1.00 |
| IDS | iduronate 2-sulfatase | 202438_x_at | 2.15 | 5.82 |
| ADAT3 | adenosine deaminase, tRNA-specific 3, TAD3 homolog (S. cerevisiae) | 230634_x_at | 2.15 | 5.82 |
| PORCN | porcupine homolog (Drosophila) | 219483_s_at | 2.15 | 3.67 |
| ENGASE | endo-beta-N-acetylglucosaminidase | 65635_at | 2.15 | 2.06 |
| EHBP1L1 | EH domain binding protein 1-like 1 | 91703_at | 2.15 | 1.44 |
| HSD11B1 | hydroxysteroid (11-beta) dehydrogenase 1 | 205404_at | 2.15 | 9.00 |
| CES2 | carboxylesterase 2 | 209668_x_at | 2.15 | 0.73 |
| EIF5A | Eukaryotic translation initiation factor 5A | 213757_at | 2.15 | 3.67 |
| NEURL | neuralized homolog (Drosophila) | 204889_s_at | 2.14 | 4.15 |
| ANGPTL4 | angiopoietin-like 4 | 221009_s_at | 2.14 | 1.67 |
| TFCP2 | transcription factor CP2 | 207627_s_at | 2.14 | 0.86 |
| KIAA1217 | KIAA1217 | 237167_at | 2.14 | 3.67 |
| CDON | Cdon homolog (mouse) | 227526_at | 2.14 | 1.67 |
| GABRE | gamma-aminobutyric acid (GABA) A receptor, epsilon | 204537_s_at | 2.14 | 3.21 |
| C5orf41 | chromosome 5 open reading frame 41 | 1554229_at | 2.14 | 0.86 |
| MED12 | mediator complex subunit 12 | 211342_x_at | 2.14 | 1.00 |
| ANO10 | anoctamin 10 | 218910_at | 2.14 | 0.86 |
| PDGFA | Platelet-derived growth factor alpha polypeptide | 229830_at | 2.14 | 2.06 |
| FKBP4 | FK506 binding protein 4, 59kDa | 200894_s_at | 2.14 | 0.86 |
| API5 | apoptosis inhibitor 5 | 201686_x_at | 2.14 | 1.67 |
| C17orf63 | chromosome 17 open reading frame 63 | 229418_at | 2.14 | 1.67 |
| RGL3 | ral guanine nucleotide dissociation stimulator-like 3 | 228877_at | 2.14 | 5.82 |
| PCIF1 | PDX1 C-terminal inhibiting factor 1 | 222045_s_at | 2.14 | 3.21 |
| ACTR1B | ARP1 actin-related protein 1 homolog B, centractin beta (yeast) | 202135_s_at | 2.14 | 1.00 |
| IRF2BP2 | interferon regulatory factor 2 binding protein 2 | 224572_s_at | 2.14 | 2.45 |
| PCGF3 | polycomb group ring finger 3 | 204564_at | 2.14 | 1.25 |
| UHRF1BP1L | UHRF1 binding protein 1-like | 1554292_a_at | 2.14 | 5.82 |
| RPTOR | regulatory associated protein of MTOR, complex 1 | 225715_at | 2.14 | 1.00 |
| C9orf25 | chromosome 9 open reading frame 25 | 225146_at | 2.14 | 3.67 |
| MOSPD1 | motile sperm domain containing 1 | 1557455_s_at | 2.14 | 1.44 |
| DFFA | DNA fragmentation factor, 45kDa, alpha polypeptide | 203277_at | 2.14 | 1.00 |
| ZDHHC20 | zinc finger, DHHC-type containing 20 | 243786_at | 2.14 | 5.82 |
| PDE1A | phosphodiesterase 1A, calmodulin-dependent | 1558680_s_at | 2.14 | 3.67 |
| GLYR1 | glyoxylate reductase 1 homolog (Arabidopsis) | 222652_s_at | 2.14 | 1.00 |
| LOC729970 | hCG2028352-like | 235362_at | 2.14 | 2.06 |
| LRRC68 | leucine rich repeat containing 68 | 213751_at | 2.14 | 3.21 |
| RCHY1 | ring finger and CHY zinc finger domain containing 1 | 212743_at | 2.13 | 1.44 |
| USP31 | ubiquitin specific peptidase 31 | 1558117_s_at | 2.13 | 3.67 |
| CCDC74A /// CCDC74B | coiled-coil domain containing 74A /// coiled-coil domain containing 74B | 227966_s_at | 2.13 | 1.67 |
| ISM2 | isthmin 2 homolog (zebrafish) | 228212_at | 2.13 | 5.82 |
| ZNF398 | zinc finger protein 398 | 1555015_a_at | 2.13 | 1.25 |
| HHEX | hematopoietically expressed homeobox | 204689_at | 2.13 | 9.00 |
| CDC42EP3 | CDC42 effector protein (Rho GTPase binding) 3 | 209287_s_at | 2.13 | 2.06 |
| TBC1D9 | TBC1 domain family, member 9 (with GRAM domain) | 212960_at | 2.13 | 1.67 |
| SEPT9 | septin 9 | 207425_s_at | 2.13 | 1.67 |
| LOC100510649 | hypothetical LOC100510649 | 227260_at | 2.13 | 3.67 |
| ICAM1 | intercellular adhesion molecule 1 | 202638_s_at | 2.13 | 3.21 |
| EAF2 | ELL associated factor 2 | 219551_at | 2.13 | 2.72 |
| PPHLN1 | periphilin 1 | 234459_at | 2.13 | 5.82 |
| N4BP2L1 | NEDD4 binding protein 2-like 1 | 217197_x_at | 2.13 | 1.25 |
| ZNF581 | zinc finger protein 581 | 223389_s_at | 2.13 | 2.06 |
| ERAL1 | Era G-protein-like 1 (E. coli) | 212087_s_at | 2.13 | 1.44 |
| FOXQ1 | forkhead box Q1 | 227475_at | 2.13 | 2.45 |
| PYCR2 | pyrroline-5-carboxylate reductase family, member 2 | 231715_s_at | 2.13 | 2.45 |
| PTGDS | prostaglandin D2 synthase 21kDa (brain) | 212187_x_at | 2.13 | 4.93 |
| TMEM30A | transmembrane protein 30A | 232591_s_at | 2.13 | 2.45 |
| CNTNAP1 | contactin associated protein 1 | 219400_at | 2.13 | 0.86 |
| SKP2 | S-phase kinase-associated protein 2 (p45) | 210567_s_at | 2.12 | 1.67 |
| FLOT2 | flotillin 2 | 201350_at | 2.12 | 0.63 |
| CRKL | v-crk sarcoma virus CT10 oncogene homolog (avian)-like | 206184_at | 2.12 | 1.44 |
| SH2B2 | SH2B adaptor protein 2 | 205367_at | 2.12 | 3.67 |
| BRPF3 | bromodomain and PHD finger containing, 3 | 1565843_s_at | 2.12 | 4.93 |
| KIAA2013 | KIAA2013 | 224706_at | 2.12 | 0.73 |
| CCBL1 | cysteine conjugate-beta lyase, cytoplasmic | 206037_at | 2.12 | 2.06 |
| IRF2 | interferon regulatory factor 2 | 203275_at | 2.12 | 2.06 |
| CCNL2 | cyclin L2 | 221427_s_at | 2.12 | 1.00 |
| NAALADL1 | N-acetylated alpha-linked acidic dipeptidase-like 1 | 207895_at | 2.12 | 1.25 |
| POLR2E | polymerase (RNA) II (DNA directed) polypeptide E, 25kDa | 213887_s_at | 2.12 | 1.44 |
| CD68 | CD68 molecule | 203507_at | 2.12 | 1.67 |
| EGFR | epidermal growth factor receptor | 211551_at | 2.12 | 3.21 |
| SLC2A13 | solute carrier family 2 (facilitated glucose transporter), member 13 | 1552695_a_at | 2.12 | 2.72 |
| ERP27 | endoplasmic reticulum protein 27 | 227450_at | 2.12 | 5.82 |
| PRR7 | proline rich 7 (synaptic) | 219742_at | 2.12 | 3.21 |
| PRR16 | proline rich 16 | 1554867_a_at | 2.12 | 2.72 |
| RBMX | RNA binding motif protein, X-linked | 1556336_at | 2.12 | 3.67 |
| TBRG4 | transforming growth factor beta regulator 4 | 220789_s_at | 2.12 | 1.44 |
| NAV3 | neuron navigator 3 | 1552658_a_at | 2.12 | 1.67 |
| C1orf25 | chromosome 1 open reading frame 25 | 233750_s_at | 2.12 | 1.00 |
| DAG1 | dystroglycan 1 (dystrophin-associated glycoprotein 1) | 212128_s_at | 2.12 | 2.45 |
| CNIH3 | cornichon homolog 3 (Drosophila) | 214841_at | 2.12 | 2.45 |
| HCN2 | hyperpolarization activated cyclic nucleotide-gated potassium channel 2 | 214893_x_at | 2.11 | 2.45 |
| SLC2A3 | solute carrier family 2 (facilitated glucose transporter), member 3 | 202497_x_at | 2.11 | 2.06 |
| ASAP1 | ArfGAP with SH3 domain, ankyrin repeat and PH domain 1 | 221039_s_at | 2.11 | 1.44 |
| SETD5 | SET domain containing 5 | 221806_s_at | 2.11 | 1.44 |
| CDK11A /// CDK11B | cyclin-dependent kinase 11A /// cyclin-dependent kinase 11B | 207428_x_at | 2.11 | 2.06 |
| ABI2 | abl-interactor 2 | 209856_x_at | 2.11 | 0.86 |
| SH3TC1 | SH3 domain and tetratricopeptide repeats 1 | 219256_s_at | 2.11 | 2.06 |
| LOC100506942 | hypothetical LOC100506942 | 238247_at | 2.11 | 2.06 |
| C6orf62 | Chromosome 6 open reading frame 62 | 213872_at | 2.11 | 3.21 |
| CUL4B | cullin 4B | 210257_x_at | 2.11 | 1.67 |
| MARCH4 | membrane-associated ring finger (C3HC4) 4 | 230112_at | 2.11 | 5.82 |
| RAB8B | RAB8B, member RAS oncogene family | 222846_at | 2.11 | 0.86 |
| NEU1 | sialidase 1 (lysosomal sialidase) | 208926_at | 2.11 | 0.86 |
| SIAH2 | seven in absentia homolog 2 (Drosophila) | 209339_at | 2.11 | 1.44 |
| PAFAH2 | platelet-activating factor acetylhydrolase 2, 40kDa | 205232_s_at | 2.11 | 1.25 |
| ARIH2 | ariadne homolog 2 (Drosophila) | 201228_s_at | 2.11 | 1.00 |
| TUBB | tubulin, beta | 209026_x_at | 2.10 | 1.44 |
| CABP4 | calcium binding protein 4 | 1554202_x_at | 2.10 | 3.67 |
| TIMP2 | TIMP metallopeptidase inhibitor 2 | 203167_at | 2.10 | 0.86 |
| ZNF598 | zinc finger protein 598 | 225104_at | 2.10 | 0.73 |
| FASTK | Fas-activated serine/threonine kinase | 202676_x_at | 2.10 | 0.86 |
| NFRKB | nuclear factor related to kappaB binding protein | 206968_s_at | 2.10 | 0.73 |
| NDRG3 | NDRG family member 3 | 224368_s_at | 2.10 | 2.06 |
| GSR | glutathione reductase | 205770_at | 2.10 | 2.06 |
| SCARA3 | scavenger receptor class A, member 3 | 219416_at | 2.10 | 1.44 |
| IFIT3 | interferon-induced protein with tetratricopeptide repeats 3 | 204747_at | 2.10 | 7.84 |
| PPP1R10 | protein phosphatase 1, regulatory (inhibitor) subunit 10 | 201703_s_at | 2.10 | 0.73 |
| GOSR2 | golgi SNAP receptor complex member 2 | 213144_at | 2.10 | 1.25 |
| SEC22B | SEC22 vesicle trafficking protein homolog B (S. cerevisiae) (gene/pseudogene) | 214257_s_at | 2.10 | 1.44 |
| SLC39A8 | solute carrier family 39 (zinc transporter), member 8 | 219869_s_at | 2.10 | 3.67 |
| ERGIC3 | ERGIC and golgi 3 | 216032_s_at | 2.10 | 1.00 |
| CPSF3L | cleavage and polyadenylation specific factor 3-like | 233625_x_at | 2.10 | 0.35 |
| LEPR | leptin receptor | 211355_x_at | 2.10 | 9.00 |
| TANK | TRAF family member-associated NFKB activator | 210458_s_at | 2.10 | 4.93 |
| SCAMP2 | secretory carrier membrane protein 2 | 218143_s_at | 2.10 | 0.73 |
| EXOSC5 | exosome component 5 | 218481_at | 2.10 | 1.00 |
| PLEKHM2 | pleckstrin homology domain containing, family M (with RUN domain) member 2 | 212146_at | 2.10 | 1.44 |
| COASY | CoA synthase | 201913_s_at | 2.09 | 0.63 |
| MAP3K7 | mitogen-activated protein kinase kinase kinase 7 | 211537_x_at | 2.09 | 1.44 |
| CACYBP | calcyclin binding protein | 201382_at | 2.09 | 3.21 |
| PNPO | pyridoxamine 5'-phosphate oxidase | 222653_at | 2.09 | 2.06 |
| AR | androgen receptor | 211621_at | 2.09 | 3.67 |
| CLK3 | CDC-like kinase 3 | 202140_s_at | 2.09 | 2.45 |
| SEC61A2 | Sec61 alpha 2 subunit (S. cerevisiae) | 228747_at | 2.09 | 2.45 |
| LGALS8 | lectin, galactoside-binding, soluble, 8 | 210732_s_at | 2.09 | 1.00 |
| MAMSTR | MEF2 activating motif and SAP domain containing transcriptional regulator | 1557091_at | 2.09 | 4.15 |
| SPATA24 | spermatogenesis associated 24 | 1558641_at | 2.09 | 1.25 |
| FOXS1 | forkhead box S1 | 229731_at | 2.09 | 2.72 |
| XPNPEP3 | X-prolyl aminopeptidase (aminopeptidase P) 3, putative | 220020_at | 2.09 | 0.86 |
| C6orf106 | chromosome 6 open reading frame 106 | 217924_at | 2.09 | 4.93 |
| RNF123 | ring finger protein 123 | 224186_s_at | 2.09 | 0.73 |
| SORBS2 | sorbin and SH3 domain containing 2 | 204288_s_at | 2.09 | 1.44 |
| PATL1 | protein associated with topoisomerase II homolog 1 (yeast) | 225468_at | 2.09 | 1.67 |
| TAB2 | TGF-beta activated kinase 1/MAP3K7 binding protein 2 | 210284_s_at | 2.09 | 1.44 |
| TERF2 | telomeric repeat binding factor 2 | 203611_at | 2.09 | 1.00 |
| CALHM1 | calcium homeostasis modulator 1 | 1554367_at | 2.09 | 3.21 |
| PTAR1 | protein prenyltransferase alpha subunit repeat containing 1 | 235484_at | 2.09 | 0.86 |
| WASH1 /// WASH2P | WAS protein family homolog 1 /// WAS protein family homolog 2 pseudogene | 225995_x_at | 2.08 | 1.25 |
| PLAUR | plasminogen activator, urokinase receptor | 210845_s_at | 2.08 | 1.44 |
| WNT5B | wingless-type MMTV integration site family, member 5B | 221029_s_at | 2.08 | 1.00 |
| CELSR3 | cadherin, EGF LAG seven-pass G-type receptor 3 (flamingo homolog, Drosophila) | 40020_at | 2.08 | 2.06 |
| IPO13 | importin 13 | 203546_at | 2.08 | 1.44 |
| ZNF579 | zinc finger protein 579 | 229140_at | 2.08 | 2.45 |
| RNF40 | ring finger protein 40 | 206845_s_at | 2.08 | 0.86 |
| TRIM16L | tripartite motif-containing 16-like | 1559681_a_at | 2.08 | 2.72 |
| SPIRE1 | spire homolog 1 (Drosophila) | 1554807_a_at | 2.08 | 2.72 |
| PHTF2 | putative homeodomain transcription factor 2 | 215286_s_at | 2.08 | 2.72 |
| RBCK1 | RanBP-type and C3HC4-type zinc finger containing 1 | 207713_s_at | 2.08 | 1.67 |
| APBA3 | amyloid beta (A4) precursor protein-binding, family A, member 3 | 215148_s_at | 2.08 | 1.25 |
| ASAH1 | N-acylsphingosine amidohydrolase (acid ceramidase) 1 | 1555419_a_at | 2.07 | 1.25 |
| UGT1A1 /// UGT1A10 /// UGT1A4 /// UGT1A6 /// UGT1A8 /// UGT1A9 | UDP glucuronosyltransferase 1 family, polypeptide A1 /// UDP glucuronosyltransferase 1 family, polypeptide A10 /// UDP glucuronosyltransferase 1 family, polypeptide A4 /// UDP glucuronosyltransferase 1 family, polypeptide A6 /// UDP glucuronosyltransferase 1 family, polypeptide A8 /// UDP glucuronosyltransferase 1 family, polypeptide A9 | 204532_x_at | 2.07 | 1.67 |
| CCNT2 | cyclin T2 | 214638_s_at | 2.07 | 2.45 |
| PCDH18 | protocadherin 18 | 225977_at | 2.07 | 1.67 |
| RALB | v-ral simian leukemia viral oncogene homolog B (ras related; GTP binding protein) | 202101_s_at | 2.07 | 1.67 |
| PHC2 | polyhomeotic homolog 2 (Drosophila) | 200919_at | 2.07 | 0.86 |
| MIF4GD | MIF4G domain containing | 231727_s_at | 2.07 | 1.44 |
| ARIH2 | ariadne homolog 2 (Drosophila) | 216008_s_at | 2.07 | 2.72 |
| PPFIA1 | protein tyrosine phosphatase, receptor type, f polypeptide (PTPRF), interacting protein (liprin), alpha 1 | 210235_s_at | 2.07 | 2.45 |
| MLLT6 | myeloid/lymphoid or mixed-lineage leukemia (trithorax homolog, Drosophila); translocated to, 6 | 224784_at | 2.07 | 0.73 |
| DBT | dihydrolipoamide branched chain transacylase E2 | 205369_x_at | 2.07 | 4.93 |
| CBLN3 | cerebellin 3 precursor | 235221_at | 2.07 | 2.06 |
| TRPM4 | transient receptor potential cation channel, subfamily M, member 4 | 219360_s_at | 2.07 | 3.21 |
| CREB3L1 | cAMP responsive element binding protein 3-like 1 | 213059_at | 2.07 | 1.00 |
| MRPL2 | mitochondrial ribosomal protein L2 | 218887_at | 2.07 | 0.73 |
| LRP1 | low density lipoprotein receptor-related protein 1 | 200785_s_at | 2.07 | 1.67 |
| CFLAR | CASP8 and FADD-like apoptosis regulator | 210564_x_at | 2.07 | 2.45 |
| ZNF548 | zinc finger protein 548 | 1553718_at | 2.07 | 3.67 |
| C1orf43 | chromosome 1 open reading frame 43 | 1555226_s_at | 2.07 | 1.25 |
| POLH | polymerase (DNA directed), eta | 222879_s_at | 2.07 | 1.25 |
| SMARCC2 | SWI/SNF related, matrix associated, actin dependent regulator of chromatin, subfamily c, member 2 | 201320_at | 2.06 | 2.72 |
| GALNT5 | UDP-N-acetyl-alpha-D-galactosamine:polypeptide N-acetylgalactosaminyltransferase 5 (GalNAc-T5) | 229555_at | 2.06 | 3.21 |
| SMEK1 | SMEK homolog 1, suppressor of mek1 (Dictyostelium) | 220368_s_at | 2.06 | 1.00 |
| CNOT8 | CCR4-NOT transcription complex, subunit 8 | 202162_s_at | 2.06 | 1.25 |
| NCS1 | neuronal calcium sensor 1 | 222570_at | 2.06 | 1.00 |
| TP53I11 | tumor protein p53 inducible protein 11 | 203421_at | 2.06 | 3.21 |
| SYDE1 | synapse defective 1, Rho GTPase, homolog 1 (C. elegans) | 216272_x_at | 2.06 | 2.72 |
| XPO5 | exportin 5 | 223057_s_at | 2.06 | 2.06 |
| EDC3 | enhancer of mRNA decapping 3 homolog (S. cerevisiae) | 226042_at | 2.06 | 1.44 |
| RAB30 | RAB30, member RAS oncogene family | 227842_at | 2.06 | 2.45 |
| SLC2A5 | solute carrier family 2 (facilitated glucose/fructose transporter), member 5 | 204430_s_at | 2.06 | 3.67 |
| HTRA2 | HtrA serine peptidase 2 | 211152_s_at | 2.06 | 1.25 |
| DGCR8 | DiGeorge syndrome critical region gene 8 | 64474_g_at | 2.06 | 2.45 |
| TTLL5 | tubulin tyrosine ligase-like family, member 5 | 208099_x_at | 2.06 | 3.21 |
| OGFR | opioid growth factor receptor | 211513_s_at | 2.06 | 2.06 |
| EPOR | erythropoietin receptor | 209963_s_at | 2.06 | 2.45 |
| DKK3 | dickkopf homolog 3 (Xenopus laevis) | 202196_s_at | 2.06 | 2.72 |
| ADSL | adenylosuccinate lyase | 210250_x_at | 2.06 | 1.67 |
| SLC4A7 | solute carrier family 4, sodium bicarbonate cotransporter, member 7 | 210286_s_at | 2.06 | 3.21 |
| ARAF | v-raf murine sarcoma 3611 viral oncogene homolog | 201895_at | 2.06 | 2.06 |
| BLOC1S1 | biogenesis of lysosomal organelles complex-1, subunit 1 | 202592_at | 2.06 | 1.25 |
| THTPA | thiamine triphosphatase | 218540_at | 2.06 | 3.67 |
| PCNX | pecanex homolog (Drosophila) | 239100_x_at | 2.06 | 4.15 |
| GTF2A2 | general transcription factor IIA, 2, 12kDa | 243985_at | 2.06 | 2.45 |
| GGT1 | gamma-glutamyltransferase 1 | 207131_x_at | 2.05 | 5.82 |
| ZNF175 | zinc finger protein 175 | 205497_at | 2.05 | 1.44 |
| RIN1 | Ras and Rab interactor 1 | 205211_s_at | 2.05 | 2.45 |
| PHLDA1 | pleckstrin homology-like domain, family A, member 1 | 217998_at | 2.05 | 1.44 |
| PHF23 | PHD finger protein 23 | 1555789_s_at | 2.05 | 1.67 |
| DNM2 | dynamin 2 | 202253_s_at | 2.05 | 1.25 |
| TIMM17B | translocase of inner mitochondrial membrane 17 homolog B (yeast) | 203342_at | 2.05 | 0.86 |
| CTSB | cathepsin B | 227961_at | 2.05 | 1.67 |
| XPNPEP1 | X-prolyl aminopeptidase (aminopeptidase P) 1, soluble | 208453_s_at | 2.05 | 1.25 |
| SMAD3 | SMAD family member 3 | 205398_s_at | 2.05 | 3.21 |
| OGFR | opioid growth factor receptor | 211512_s_at | 2.05 | 2.72 |
| METT5D1 | methyltransferase 5 domain containing 1 | 1562830_at | 2.05 | 1.67 |
| PIK3R1 | phosphoinositide-3-kinase, regulatory subunit 1 (alpha) | 212249_at | 2.05 | 2.45 |
| FXR1 | fragile X mental retardation, autosomal homolog 1 | 201635_s_at | 2.05 | 2.06 |
| ZC3H11A | zinc finger CCCH-type containing 11A | 205787_x_at | 2.05 | 1.44 |
| C8orf60 | chromosome 8 open reading frame 60 | 220712_at | 2.05 | 4.15 |
| THBS1 | thrombospondin 1 | 201108_s_at | 2.05 | 0.63 |
| SOD2 | superoxide dismutase 2, mitochondrial | 216841_s_at | 2.05 | 2.06 |
| BICD1 | bicaudal D homolog 1 (Drosophila) | 204741_at | 2.05 | 1.44 |
| CNPY3 | canopy 3 homolog (zebrafish) | 217931_at | 2.05 | 0.73 |
| XPO5 | exportin 5 | 223055_s_at | 2.05 | 2.72 |
| CD99L2 | CD99 molecule-like 2 | 233825_s_at | 2.04 | 0.86 |
| YIPF3 | Yip1 domain family, member 3 | 216338_s_at | 2.04 | 1.00 |
| ST6GALNAC4 | ST6 (alpha-N-acetyl-neuraminyl-2,3-beta-galactosyl-1,3)-N-acetylgalactosaminide alpha-2,6-sialyltransferase 4 | 220937_s_at | 2.04 | 2.06 |
| PKMYT1 | protein kinase, membrane associated tyrosine/threonine 1 | 204267_x_at | 2.04 | 4.15 |
| ATP6V0A1 | ATPase, H+ transporting, lysosomal V0 subunit a1 | 212383_at | 2.04 | 2.06 |
| ELL | elongation factor RNA polymerase II | 204095_s_at | 2.04 | 1.67 |
| SLC25A36 | Solute carrier family 25, member 36 | 237741_at | 2.04 | 2.45 |
| RNF26 | ring finger protein 26 | 224947_at | 2.04 | 1.00 |
| ENTPD7 | ectonucleoside triphosphate diphosphohydrolase 7 | 220153_at | 2.04 | 1.25 |
| PHLDA3 | pleckstrin homology-like domain, family A, member 3 | 218634_at | 2.04 | 1.44 |
| EPB41L4B | erythrocyte membrane protein band 4.1 like 4B | 223427_s_at | 2.04 | 3.67 |
| FAM174B | family with sequence similarity 174, member B | 51158_at | 2.04 | 7.84 |
| GORASP2 | golgi reassembly stacking protein 2, 55kDa | 208842_s_at | 2.04 | 1.25 |
| MLX | MAX-like protein X | 217909_s_at | 2.04 | 1.67 |
| ZIK1 | zinc finger protein interacting with K protein 1 homolog (mouse) | 235773_at | 2.04 | 1.44 |
| LIN7C | lin-7 homolog C (C. elegans) | 219399_at | 2.04 | 3.21 |
| ANGEL1 | angel homolog 1 (Drosophila) | 213099_at | 2.04 | 1.25 |
| SS18 | synovial sarcoma translocation, chromosome 18 | 209954_x_at | 2.04 | 1.44 |
| TNFAIP3 | tumor necrosis factor, alpha-induced protein 3 | 202643_s_at | 2.04 | 1.67 |
| CASP1 | caspase 1, apoptosis-related cysteine peptidase (interleukin 1, beta, convertase) | 211367_s_at | 2.04 | 3.21 |
| ZMYM3 | zinc finger, MYM-type 3 | 207559_s_at | 2.04 | 1.67 |
| GJC1 | gap junction protein, gamma 1, 45kDa | 208460_at | 2.04 | 2.72 |
| SURF4 | surfeit 4 | 222979_s_at | 2.04 | 1.67 |
| WDR26 | WD repeat domain 26 | 224898_at | 2.03 | 0.86 |
| MTAP | methylthioadenosine phosphorylase | 211364_at | 2.03 | 2.72 |
| ELAVL1 | ELAV (embryonic lethal, abnormal vision, Drosophila)-like 1 (Hu antigen R) | 201727_s_at | 2.03 | 1.00 |
| SPATA6 | spermatogenesis associated 6 | 220299_at | 2.03 | 2.45 |
| CDC5L | CDC5 cell division cycle 5-like (S. pombe) | 209055_s_at | 2.03 | 2.45 |
| MMP14 | matrix metallopeptidase 14 (membrane-inserted) | 202827_s_at | 2.03 | 2.72 |
| ATP6V0D2 | ATPase, H+ transporting, lysosomal 38kDa, V0 subunit d2 | 1553155_x_at | 2.03 | 4.93 |
| HLA-F | major histocompatibility complex, class I, F | 221875_x_at | 2.03 | 1.67 |
| SH2D4A | SH2 domain containing 4A | 219749_at | 2.03 | 1.44 |
| FUZ | fuzzy homolog (Drosophila) | 221187_s_at | 2.03 | 2.45 |
| ASPSCR1 | alveolar soft part sarcoma chromosome region, candidate 1 | 218908_at | 2.03 | 1.44 |
| NOB1 | NIN1/RPN12 binding protein 1 homolog (S. cerevisiae) | 223018_at | 2.03 | 2.45 |
| LONRF3 | LON peptidase N-terminal domain and ring finger 3 | 242931_at | 2.03 | 2.72 |
| TRAM1 | translocation associated membrane protein 1 | 201399_s_at | 2.03 | 1.67 |
| MED16 | mediator complex subunit 16 | 221938_x_at | 2.03 | 2.45 |
| SMA4 | glucuronidase, beta pseudogene | 206565_x_at | 2.03 | 3.67 |
| BCAT1 | branched chain amino-acid transaminase 1, cytosolic | 214452_at | 2.03 | 2.45 |
| DENR | density-regulated protein | 234915_s_at | 2.03 | 2.06 |
| BAT3 | HLA-B associated transcript 3 | 201255_x_at | 2.03 | 0.73 |
| ABCC10 | ATP-binding cassette, sub-family C (CFTR/MRP), member 10 | 215873_x_at | 2.03 | 1.44 |
| TMTC1 | transmembrane and tetratricopeptide repeat containing 1 | 226322_at | 2.03 | 4.93 |
| COL11A2 | collagen, type XI, alpha 2 | 216993_s_at | 2.03 | 3.67 |
| PTPN9 | protein tyrosine phosphatase, non-receptor type 9 | 202958_at | 2.03 | 0.55 |
| TNFSF12-TNFSF13 /// TNFSF13 | TNFSF12-TNFSF13 readthrough /// tumor necrosis factor (ligand) superfamily, member 13 | 209499_x_at | 2.03 | 5.82 |
| EFNB2 | ephrin-B2 | 202669_s_at | 2.03 | 9.00 |
| TECR | trans-2,3-enoyl-CoA reductase | 208336_s_at | 2.03 | 1.00 |
| LPAR1 | lysophosphatidic acid receptor 1 | 204038_s_at | 2.03 | 2.72 |
| DNAJB4 | DnaJ (Hsp40) homolog, subfamily B, member 4 | 203811_s_at | 2.03 | 4.15 |
| GLT25D2 | glycosyltransferase 25 domain containing 2 | 209883_at | 2.03 | 3.67 |
| NAP1L4 | nucleosome assembly protein 1-like 4 | 1560339_s_at | 2.03 | 0.86 |
| H2AFY | H2A histone family, member Y | 214500_at | 2.03 | 1.00 |
| FANCI | Fanconi anemia, complementation group I | 223785_at | 2.02 | 3.67 |
| VPS26B | vacuolar protein sorting 26 homolog B (S. pombe) | 230306_at | 2.02 | 1.44 |
| FAM60A | family with sequence similarity 60, member A | 220147_s_at | 2.02 | 1.67 |
| PSME3 | proteasome (prosome, macropain) activator subunit 3 (PA28 gamma; Ki) | 209852_x_at | 2.02 | 2.72 |
| PPAP2B | phosphatidic acid phosphatase type 2B | 209355_s_at | 2.02 | 6.81 |
| GIT1 | G protein-coupled receptor kinase interacting ArfGAP 1 | 218030_at | 2.02 | 3.21 |
| RPUSD3 | RNA pseudouridylate synthase domain containing 3 | 225743_at | 2.02 | 3.21 |
| KLHL7 | kelch-like 7 (Drosophila) | 220238_s_at | 2.02 | 1.44 |
| ZNF19 | zinc finger protein 19 | 228958_at | 2.02 | 2.45 |
| ZFP36L2 | zinc finger protein 36, C3H type-like 2 | 201369_s_at | 2.02 | 2.06 |
| DAB2 | disabled homolog 2, mitogen-responsive phosphoprotein (Drosophila) | 240873_x_at | 2.02 | 1.67 |
| C7orf27 | chromosome 7 open reading frame 27 | 225437_s_at | 2.02 | 0.86 |
| SCAMP2 | secretory carrier membrane protein 2 | 224921_at | 2.02 | 0.86 |
| C1orf213 | chromosome 1 open reading frame 213 | 229086_at | 2.02 | 2.45 |
| PAM16 | presequence translocase-associated motor 16 homolog (S. cerevisiae) | 218969_at | 2.02 | 1.44 |
| TPRA1 | transmembrane protein, adipocyte asscociated 1 | 218855_at | 2.02 | 1.00 |
| SNX11 | sorting nexin 11 | 53912_at | 2.02 | 1.44 |
| CSRP2 | cysteine and glycine-rich protein 2 | 211126_s_at | 2.02 | 4.93 |
| TMEM194A | transmembrane protein 194A | 212619_at | 2.02 | 1.44 |
| SLC16A6 | solute carrier family 16, member 6 (monocarboxylic acid transporter 7) | 230748_at | 2.02 | 3.21 |
| RPH3AL | rabphilin 3A-like (without C2 domains) | 221614_s_at | 2.02 | 4.15 |
| PREX1 | phosphatidylinositol-3,4,5-trisphosphate-dependent Rac exchange factor 1 | 224925_at | 2.02 | 2.45 |
| PRR12 | proline rich 12 | 226716_at | 2.02 | 1.44 |
| TNPO2 | transportin 2 | 221507_at | 2.01 | 2.06 |
| MLLT10 | myeloid/lymphoid or mixed-lineage leukemia (trithorax homolog, Drosophila); translocated to, 10 | 205408_at | 2.01 | 2.06 |
| MAP1LC3A | microtubule-associated protein 1 light chain 3 alpha | 227219_x_at | 2.01 | 0.73 |
| STX6 | syntaxin 6 | 1552618_at | 2.01 | 1.00 |
| FLOT1 | flotillin 1 | 208749_x_at | 2.01 | 1.44 |
| DNASE2 | deoxyribonuclease II, lysosomal | 214992_s_at | 2.01 | 0.86 |
| C6orf138 | chromosome 6 open reading frame 138 | 234689_at | 2.01 | 3.21 |
| C11orf68 | chromosome 11 open reading frame 68 | 221534_at | 2.01 | 1.00 |
| PSG1 | pregnancy specific beta-1-glycoprotein 1 | 210195_s_at | 2.01 | 6.81 |
| SHROOM2 | shroom family member 2 | 204967_at | 2.01 | 2.45 |
| POGZ | pogo transposable element with ZNF domain | 215281_x_at | 2.01 | 3.67 |
| MYOM3 | myomesin family, member 3 | 238124_at | 2.01 | 1.00 |
| B3GNT2 | UDP-GlcNAc:betaGal beta-1,3-N-acetylglucosaminyltransferase 2 | 219326_s_at | 2.01 | 1.25 |
| KIAA1539 | KIAA1539 | 207765_s_at | 2.01 | 1.25 |
| PDS5B | PDS5, regulator of cohesion maintenance, homolog B (S. cerevisiae) | 229704_at | 2.01 | 6.81 |
| LOC100288778 /// WASH1 /// WASH2P /// WASH3P /// WASH7P | WAS protein family homolog 1 pseudogene /// WAS protein family homolog 1 /// WAS protein family homolog 2 pseudogene /// WAS protein family homolog 3 pseudogene /// WAS protein family homolog 7 pseudogene | 225035_x_at | 2.01 | 2.45 |
| JRK | jerky homolog (mouse) | 216309_x_at | 2.01 | 2.06 |
| WDR8 | WD repeat domain 8 | 236381_s_at | 2.01 | 1.44 |
| WBP11 | WW domain binding protein 11 | 217821_s_at | 2.01 | 1.25 |
| SSH1 | slingshot homolog 1 (Drosophila) | 221753_at | 2.01 | 1.44 |
| CIC | capicua homolog (Drosophila) | 212784_at | 2.01 | 4.93 |
| PSME4 | proteasome (prosome, macropain) activator subunit 4 | 212220_at | 2.01 | 2.72 |
| UTP23 | UTP23, small subunit (SSU) processome component, homolog (yeast) | 238561_s_at | 2.00 | 2.72 |
| MED24 | mediator complex subunit 24 | 213043_s_at | 2.00 | 4.15 |
| SLC17A5 | solute carrier family 17 (anion/sugar transporter), member 5 | 221041_s_at | 2.00 | 1.00 |
| MID1IP1 | MID1 interacting protein 1 (gastrulation specific G12 homolog (zebrafish)) | 218251_at | 2.00 | 1.44 |
| GDPD3 | glycerophosphodiester phosphodiesterase domain containing 3 | 219722_s_at | 2.00 | 3.21 |
| ABHD14A /// ACY1 | abhydrolase domain containing 14A /// aminoacylase 1 | 202740_at | 2.00 | 1.00 |
| BCKDHA | branched chain keto acid dehydrogenase E1, alpha polypeptide | 202331_at | 2.00 | 3.67 |
| CFLAR | CASP8 and FADD-like apoptosis regulator | 211317_s_at | 2.00 | 2.45 |
| MFSD3 | major facilitator superfamily domain containing 3 | 227296_at | 2.00 | 3.21 |
| LOC440104 | 1110012D08Rik pseudogene | 227106_at | 2.00 | 2.45 |
| PQLC2 | PQ loop repeat containing 2 | 220453_at | 2.00 | 3.67 |
| RAD23B | RAD23 homolog B (S. cerevisiae) | 223598_at | 0.50 | 3.21 |
| SLTM | SAFB-like, transcription modulator | 217828_at | 0.50 | 3.67 |
| GRB2 | growth factor receptor-bound protein 2 | 215075_s_at | 0.50 | 2.72 |
| RPL13P5 | ribosomal protein L13 pseudogene 5 | 210822_at | 0.50 | 6.81 |
| LYPLA1 | lysophospholipase I | 212449_s_at | 0.50 | 2.72 |
| QKI | quaking homolog, KH domain RNA binding (mouse) | 212263_at | 0.50 | 2.72 |
| ITFG1 | integrin alpha FG-GAP repeat containing 1 | 227191_at | 0.50 | 3.67 |
| ZBED1 | zinc finger, BED-type containing 1 | 1554821_a_at | 0.50 | 5.82 |
| BAT2L2 | HLA-B associated transcript 2-like 2 | 214055_x_at | 0.50 | 3.21 |
| CDC14B | CDC14 cell division cycle 14 homolog B (S. cerevisiae) | 221555_x_at | 0.50 | 2.72 |
| TTC3 | tetratricopeptide repeat domain 3 | 208663_s_at | 0.50 | 3.21 |
| DCAF13 | DDB1 and CUL4 associated factor 13 | 231784_s_at | 0.50 | 5.82 |
| HTATSF1 | HIV-1 Tat specific factor 1 | 202602_s_at | 0.50 | 4.15 |
| KLF5 | Kruppel-like factor 5 (intestinal) | 209211_at | 0.50 | 4.15 |
| N4BP1 | NEDD4 binding protein 1 | 32069_at | 0.50 | 3.67 |
| ATE1 | arginyltransferase 1 | 225497_at | 0.50 | 3.67 |
| FGFR1OP2 | FGFR1 oncogene partner 2 | 243619_at | 0.50 | 3.67 |
| SPTBN1 | Spectrin, beta, non-erythrocytic 1 | 226765_at | 0.50 | 5.82 |
| ARAF | v-raf murine sarcoma 3611 viral oncogene homolog | 230652_at | 0.50 | 5.82 |
| FGF14 | fibroblast growth factor 14 | 230231_at | 0.50 | 5.82 |
| AKAP7 | A kinase (PRKA) anchor protein 7 | 205771_s_at | 0.50 | 4.93 |
| AGBL3 | ATP/GTP binding protein-like 3 | 232395_x_at | 0.50 | 4.93 |
| TRAPPC10 | trafficking protein particle complex 10 | 209412_at | 0.50 | 3.21 |
| RAPH1 | Ras association (RalGDS/AF-6) and pleckstrin homology domains 1 | 231075_x_at | 0.50 | 4.15 |
| LOC729680 | hypothetical protein LOC729680 | 228977_at | 0.50 | 7.84 |
| ZNF207 | zinc finger protein 207 | 228157_at | 0.50 | 4.15 |
| LOC644538 | hypothetical protein LOC644538 | 227976_at | 0.50 | 4.15 |
| IL6ST | interleukin 6 signal transducer (gp130, oncostatin M receptor) | 204863_s_at | 0.50 | 3.67 |
| ZNF439 | zinc finger protein 439 | 236562_at | 0.50 | 6.81 |
| DDX46 | DEAD (Asp-Glu-Ala-Asp) box polypeptide 46 | 228039_at | 0.50 | 4.93 |
| PGLS | 6-phosphogluconolactonase | 230699_at | 0.50 | 2.72 |
| ARL10 | ADP-ribosylation factor-like 10 | 228843_at | 0.50 | 3.67 |
| ZNF428 | zinc finger protein 428 | 215429_s_at | 0.50 | 3.67 |
| PLEKHA1 | pleckstrin homology domain containing, family A (phosphoinositide binding specific) member 1 | 226247_at | 0.50 | 2.72 |
| SNX21 | sorting nexin family member 21 | 1553960_at | 0.50 | 3.67 |
| GTF2H2B | general transcription factor IIH, polypeptide 2B | 215470_at | 0.50 | 4.93 |
| LOC100130097 | Hypothetical LOC100130097 | 241376_at | 0.50 | 3.67 |
| ZNF283 | zinc finger protein 283 | 243188_at | 0.50 | 4.93 |
| ITPR2 | inositol 1,4,5-triphosphate receptor, type 2 | 202660_at | 0.50 | 4.93 |
| API5 | apoptosis inhibitor 5 | 214960_at | 0.50 | 4.93 |
| ZBTB24 | zinc finger and BTB domain containing 24 | 205340_at | 0.50 | 3.67 |
| TNPO3 | transportin 3 | 212317_at | 0.50 | 2.72 |
| LOC100506395 | hypothetical LOC100506395 | 238341_at | 0.50 | 7.84 |
| RSBN1 | round spermatid basic protein 1 | 222788_s_at | 0.50 | 5.82 |
| PDZD8 | PDZ domain containing 8 | 213549_at | 0.49 | 3.67 |
| FKBP3 | FK506 binding protein 3, 25kDa | 218003_s_at | 0.49 | 4.15 |
| CSPP1 | centrosome and spindle pole associated protein 1 | 227105_at | 0.49 | 3.21 |
| SYNCRIP | synaptotagmin binding, cytoplasmic RNA interacting protein | 1555427_s_at | 0.49 | 4.93 |
| GATAD2B | GATA zinc finger domain containing 2B | 238076_at | 0.49 | 4.15 |
| FLJ10038 | hypothetical protein FLJ10038 | 205510_s_at | 0.49 | 4.15 |
| TRIM59 | tripartite motif-containing 59 | 227801_at | 0.49 | 3.67 |
| YTHDC1 | YTH domain containing 1 | 240459_at | 0.49 | 5.82 |
| FAM66C /// FAM66D | family with sequence similarity 66, member C /// family with sequence similarity 66, member D | 1559952_x_at | 0.49 | 4.93 |
| SLC37A3 | solute carrier family 37 (glycerol-3-phosphate transporter), member 3 | 223304_at | 0.49 | 3.67 |
| CELF1 | CUGBP, Elav-like family member 1 | 221743_at | 0.49 | 1.67 |
| TADA2B | transcriptional adaptor 2B | 236248_x_at | 0.49 | 5.82 |
| PPP1CB | protein phosphatase 1, catalytic subunit, beta isozyme | 201407_s_at | 0.49 | 2.06 |
| SLC26A2 | solute carrier family 26 (sulfate transporter), member 2 | 224959_at | 0.49 | 4.93 |
| KCND3 | potassium voltage-gated channel, Shal-related subfamily, member 3 | 215014_at | 0.49 | 3.67 |
| FAM101B | family with sequence similarity 101, member B | 226876_at | 0.49 | 4.93 |
| SYNPO2 | synaptopodin 2 | 227662_at | 0.49 | 4.93 |
| OSBPL10 | oxysterol binding protein-like 10 | 219073_s_at | 0.49 | 4.15 |
| HSD17B6 | hydroxysteroid (17-beta) dehydrogenase 6 homolog (mouse) | 205700_at | 0.49 | 7.84 |
| VEPH1 | ventricular zone expressed PH domain homolog 1 (zebrafish) | 229759_s_at | 0.49 | 9.00 |
| PLXNA1 | plexin A1 | 221538_s_at | 0.49 | 3.67 |
| ITSN2 | intersectin 2 | 209898_x_at | 0.49 | 2.72 |
| PHKB | phosphorylase kinase, beta | 238601_at | 0.49 | 3.67 |
| KLHDC5 | kelch domain containing 5 | 225732_at | 0.49 | 2.72 |
| ISCA1 | iron-sulfur cluster assembly 1 homolog (S. cerevisiae) | 209274_s_at | 0.49 | 2.45 |
| MTDH | metadherin | 212248_at | 0.49 | 3.21 |
| LSM14B | LSM14B, SCD6 homolog B (S. cerevisiae) | 231200_at | 0.49 | 4.15 |
| FAM115A | family with sequence similarity 115, member A | 212981_s_at | 0.49 | 2.45 |
| DNAJC9 | DnaJ (Hsp40) homolog, subfamily C, member 9 | 213092_x_at | 0.49 | 4.15 |
| CXCR7 | chemokine (C-X-C motif) receptor 7 | 212977_at | 0.49 | 4.93 |
| THEM4 | thioesterase superfamily member 4 | 229253_at | 0.49 | 3.67 |
| HAUS6 | HAUS augmin-like complex, subunit 6 | 222685_at | 0.49 | 5.82 |
| KIAA1841 | KIAA1841 | 243539_at | 0.49 | 4.15 |
| PAG1 | phosphoprotein associated with glycosphingolipid microdomains 1 | 227354_at | 0.49 | 3.21 |
| FAM133B /// LOC728066 | family with sequence similarity 133, member B /// family with sequence similarity 133, member B pseudogene | 226332_at | 0.49 | 2.72 |
| EPT1 | ethanolaminephosphotransferase 1 (CDP-ethanolamine-specific) | 1555274_a_at | 0.49 | 6.81 |
| TRNT1 | tRNA nucleotidyl transferase, CCA-adding, 1 | 222754_at | 0.49 | 5.82 |
| ACER3 | alkaline ceramidase 3 | 222688_at | 0.49 | 3.21 |
| RB1CC1 | RB1-inducible coiled-coil 1 | 202034_x_at | 0.49 | 2.45 |
| GTF2H1 | general transcription factor IIH, polypeptide 1, 62kDa | 202451_at | 0.49 | 2.72 |
| PTN | pleiotrophin | 211737_x_at | 0.49 | 6.81 |
| PCM1 | pericentriolar material 1 | 228905_at | 0.49 | 3.67 |
| GATAD2A | GATA zinc finger domain containing 2A | 218131_s_at | 0.49 | 3.21 |
| MYSM1 | Myb-like, SWIRM and MPN domains 1 | 225760_at | 0.49 | 4.15 |
| SAR1A | SAR1 homolog A (S. cerevisiae) | 201542_at | 0.49 | 2.72 |
| TTC39C | tetratricopeptide repeat domain 39C | 238480_at | 0.49 | 7.84 |
| MTDH | metadherin | 212251_at | 0.49 | 2.06 |
| LEPR | leptin receptor | 209894_at | 0.49 | 4.93 |
| SRSF3 | serine/arginine-rich splicing factor 3 | 208672_s_at | 0.49 | 3.67 |
| LYRM2 | LYR motif containing 2 | 227712_at | 0.49 | 4.93 |
| C11orf82 | chromosome 11 open reading frame 82 | 228281_at | 0.49 | 7.84 |
| ANKRD20A1 /// ANKRD20A2 /// ANKRD20A3 /// ANKRD20A4 /// C21orf81 /// LOC100132733 /// LOC284232 | ankyrin repeat domain 20 family, member A1 /// ankyrin repeat domain 20 family, member A2 /// ankyrin repeat domain 20 family, member A3 /// ankyrin repeat domain 20 family, member A4 /// ankyrin repeat domain 20 family, member A3 pseudogene /// ankyrin repeat domain-containing protein 20A3-like /// ankyrin repeat domain 20 family, member A2 pseudogene | 1569607_s_at | 0.49 | 9.00 |
| ZBTB26 | zinc finger and BTB domain containing 26 | 227162_at | 0.49 | 2.72 |
| RASEF | RAS and EF-hand domain containing | 1553186_x_at | 0.49 | 4.93 |
| C14orf126 | chromosome 14 open reading frame 126 | 227158_at | 0.49 | 3.67 |
| PICALM | phosphatidylinositol binding clathrin assembly protein | 212511_at | 0.49 | 2.45 |
| ACN9 | ACN9 homolog (S. cerevisiae) | 218981_at | 0.49 | 2.72 |
| C2orf3 | chromosome 2 open reading frame 3 | 210175_at | 0.49 | 6.81 |
| GFM1 | G elongation factor, mitochondrial 1 | 225158_at | 0.49 | 9.00 |
| PREPL | prolyl endopeptidase-like | 212215_at | 0.49 | 3.67 |
| FAM76B | family with sequence similarity 76, member B | 226753_at | 0.49 | 3.21 |
| FAM102B | family with sequence similarity 102, member B | 226568_at | 0.49 | 2.72 |
| C17orf63 | chromosome 17 open reading frame 63 | 218464_s_at | 0.49 | 2.72 |
| MED23 | mediator complex subunit 23 | 223946_at | 0.49 | 4.15 |
| SH3BGR | SH3 domain binding glutamic acid-rich protein | 204979_s_at | 0.49 | 6.81 |
| TMEM123 | transmembrane protein 123 | 211967_at | 0.49 | 2.72 |
| NAPEPLD | N-acyl phosphatidylethanolamine phospholipase D | 242635_s_at | 0.49 | 5.82 |
| PPM1B | protein phosphatase, Mg2+/Mn2+ dependent, 1B | 213225_at | 0.49 | 4.15 |
| SP3 | Sp3 transcription factor | 232529_at | 0.49 | 9.00 |
| EWSR1 | Ewing sarcoma breakpoint region 1 | 229966_at | 0.49 | 3.67 |
| FLJ31306 | hypothetical LOC379025 | 225724_at | 0.49 | 3.21 |
| MRVI1 | murine retrovirus integration site 1 homolog | 226047_at | 0.49 | 9.00 |
| MCL1 | Myeloid cell leukemia sequence 1 (BCL2-related) | 214057_at | 0.49 | 4.93 |
| ADAL | adenosine deaminase-like | 238424_at | 0.49 | 3.21 |
| C22orf29 | chromosome 22 open reading frame 29 | 226204_at | 0.49 | 3.67 |
| MATR3 | matrin 3 | 228012_at | 0.49 | 3.21 |
| TCF7L2 | transcription factor 7-like 2 (T-cell specific, HMG-box) | 212761_at | 0.49 | 3.21 |
| ATMIN | ATM interactor | 201855_s_at | 0.49 | 3.67 |
| LOC100128727 | hypothetical LOC100128727 | 240395_at | 0.49 | 4.15 |
| LIN7C | lin-7 homolog C (C. elegans) | 221568_s_at | 0.49 | 2.06 |
| MYO10 | myosin X | 236718_at | 0.49 | 4.15 |
| NFIA | nuclear factor I/A | 224975_at | 0.49 | 7.84 |
| KIDINS220 | kinase D-interacting substrate, 220kDa | 212163_at | 0.49 | 3.21 |
| HEATR1 | HEAT repeat containing 1 | 218595_s_at | 0.49 | 3.21 |
| ZWINT | ZW10 interactor | 204026_s_at | 0.49 | 9.00 |
| MYO1C | myosin IC | 214656_x_at | 0.49 | 3.67 |
| HAUS6 | HAUS augmin-like complex, subunit 6 | 218602_s_at | 0.49 | 6.81 |
| RSF1 | remodeling and spacing factor 1 | 229885_at | 0.49 | 2.72 |
| EBNA1BP2 | EBNA1 binding protein 2 | 201323_at | 0.49 | 3.67 |
| LOC100507486 | hypothetical LOC100507486 | 235304_at | 0.48 | 5.82 |
| ATP11A | ATPase, class VI, type 11A | 230875_s_at | 0.48 | 2.72 |
| NDUFA5 | NADH dehydrogenase (ubiquinone) 1 alpha subcomplex, 5, 13kDa | 201304_at | 0.48 | 2.72 |
| PLXDC2 | plexin domain containing 2 | 227276_at | 0.48 | 3.67 |
| MOBKL1A | MOB1, Mps One Binder kinase activator-like 1A (yeast) | 225997_at | 0.48 | 2.06 |
| TBC1D24 | TBC1 domain family, member 24 | 227908_at | 0.48 | 3.21 |
| CKAP4 | cytoskeleton-associated protein 4 | 200999_s_at | 0.48 | 2.72 |
| STK38 | serine/threonine kinase 38 | 202951_at | 0.48 | 3.21 |
| TMEM66 | transmembrane protein 66 | 200847_s_at | 0.48 | 2.72 |
| ZMYND19 | zinc finger, MYND-type containing 19 | 227477_at | 0.48 | 4.93 |
| ZNF117 | zinc finger protein 117 | 235408_x_at | 0.48 | 5.82 |
| DNAJC10 | DnaJ (Hsp40) homolog, subfamily C, member 10 | 229588_at | 0.48 | 2.72 |
| ERAP1 | endoplasmic reticulum aminopeptidase 1 | 209788_s_at | 0.48 | 4.93 |
| IKBKAP | inhibitor of kappa light polypeptide gene enhancer in B-cells, kinase complex-associated protein | 202490_at | 0.48 | 3.67 |
| GBP1 | guanylate binding protein 1, interferon-inducible, 67kDa | 202270_at | 0.48 | 3.67 |
| ATF1 | activating transcription factor 1 | 1558233_s_at | 0.48 | 2.72 |
| PURA | purine-rich element binding protein A | 204021_s_at | 0.48 | 3.21 |
| CUL4A | cullin 4A | 201424_s_at | 0.48 | 3.67 |
| SLC35F5 | solute carrier family 35, member F5 | 220123_at | 0.48 | 4.93 |
| STRN3 | striatin, calmodulin binding protein 3 | 204496_at | 0.48 | 2.06 |
| GFM1 | G elongation factor, mitochondrial 1 | 225153_at | 0.48 | 4.93 |
| TASP1 | taspase, threonine aspartase, 1 | 219443_at | 0.48 | 3.21 |
| MAGT1 | magnesium transporter 1 | 224899_s_at | 0.48 | 3.21 |
| SAMD9 | sterile alpha motif domain containing 9 | 228531_at | 0.48 | 4.93 |
| C3orf55 | chromosome 3 open reading frame 55 | 238969_at | 0.48 | 5.82 |
| DDX21 | DEAD (Asp-Glu-Ala-Asp) box polypeptide 21 | 208152_s_at | 0.48 | 3.67 |
| C1orf104 | Chromosome 1 open reading frame 104 | 230256_at | 0.48 | 4.15 |
| FBXO3 | F-box protein 3 | 218432_at | 0.48 | 3.21 |
| ZNF596 | zinc finger protein 596 | 232641_at | 0.48 | 4.93 |
| SIKE1 | suppressor of IKBKE 1 | 204665_at | 0.48 | 5.82 |
| NPEPPS | aminopeptidase puromycin sensitive | 201454_s_at | 0.48 | 4.15 |
| KIAA1377 | KIAA1377 | 236325_at | 0.48 | 3.67 |
| IFT74 | intraflagellar transport 74 homolog (Chlamydomonas) | 219174_at | 0.48 | 3.21 |
| PGGT1B | protein geranylgeranyltransferase type I, beta subunit | 206288_at | 0.48 | 5.82 |
| FDX1 | ferredoxin 1 | 203646_at | 0.48 | 2.45 |
| CWC25 | CWC25 spliceosome-associated protein homolog (S. cerevisiae) | 222706_at | 0.48 | 3.67 |
| CAV2 | Caveolin 2 | 213426_s_at | 0.48 | 3.67 |
| MRVI1 | murine retrovirus integration site 1 homolog | 230214_at | 0.48 | 6.81 |
| UACA | uveal autoantigen with coiled-coil domains and ankyrin repeats | 236715_x_at | 0.48 | 4.93 |
| RPS24 | Ribosomal protein S24 | 1555878_at | 0.48 | 2.72 |
| MSRB3 | methionine sulfoxide reductase B3 | 225782_at | 0.48 | 2.72 |
| KIAA2018 | KIAA2018 | 227435_at | 0.48 | 1.67 |
| ARHGEF25 | Rho guanine nucleotide exchange factor (GEF) 25 | 227427_at | 0.48 | 4.15 |
| NME5 | non-metastatic cells 5, protein expressed in (nucleoside-diphosphate kinase) | 206197_at | 0.48 | 4.93 |
| SIKE1 | suppressor of IKBKE 1 | 228026_at | 0.48 | 2.45 |
| C1orf107 | chromosome 1 open reading frame 107 | 214193_s_at | 0.48 | 3.67 |
| MAT2B | methionine adenosyltransferase II, beta | 217993_s_at | 0.48 | 2.45 |
| AGPS | alkylglycerone phosphate synthase | 225114_at | 0.48 | 3.67 |
| CCAR1 | cell division cycle and apoptosis regulator 1 | 224736_at | 0.48 | 2.72 |
| PRKAR2A | protein kinase, cAMP-dependent, regulatory, type II, alpha | 225011_at | 0.48 | 3.67 |
| ZNF214 | zinc finger protein 214 | 220497_at | 0.48 | 4.15 |
| RIN2 | Ras and Rab interactor 2 | 209684_at | 0.48 | 4.15 |
| OBFC2A | oligonucleotide/oligosaccharide-binding fold containing 2A | 233085_s_at | 0.48 | 3.67 |
| ABAT | 4-aminobutyrate aminotransferase | 209459_s_at | 0.48 | 5.82 |
| SLC12A6 | solute carrier family 12 (potassium/chloride transporters), member 6 | 226741_at | 0.48 | 9.00 |
| DHFRL1 | dihydrofolate reductase-like 1 | 235675_at | 0.48 | 4.93 |
| JPH2 | junctophilin 2 | 229578_at | 0.48 | 4.93 |
| BLVRA | biliverdin reductase A | 203773_x_at | 0.48 | 2.45 |
| MRPL4 | mitochondrial ribosomal protein L4 | 223742_at | 0.48 | 4.15 |
| GKAP1 | G kinase anchoring protein 1 | 229312_s_at | 0.48 | 6.81 |
| TMTC3 | transmembrane and tetratricopeptide repeat containing 3 | 226600_at | 0.48 | 3.21 |
| ZNF302 | zinc finger protein 302 | 218490_s_at | 0.48 | 2.06 |
| ATL1 | atlastin GTPase 1 | 223340_at | 0.48 | 4.15 |
| HIPK2 | homeodomain interacting protein kinase 2 | 219028_at | 0.48 | 3.21 |
| LOC642236 | Similar to FRG1 protein (FSHD region gene 1 protein) | 242770_at | 0.48 | 4.93 |
| TRIM2 | tripartite motif-containing 2 | 202342_s_at | 0.48 | 2.72 |
| CKS2 | CDC28 protein kinase regulatory subunit 2 | 204170_s_at | 0.48 | 4.93 |
| FBXO11 | F-box protein 11 | 219208_at | 0.48 | 3.67 |
| ACOX1 | acyl-CoA oxidase 1, palmitoyl | 209600_s_at | 0.48 | 3.21 |
| KIF3A | kinesin family member 3A | 228680_at | 0.48 | 3.21 |
| FAM101B | family with sequence similarity 101, member B | 226905_at | 0.48 | 4.15 |
| BMP2K | BMP2 inducible kinase | 219546_at | 0.48 | 6.81 |
| DARS | aspartyl-tRNA synthetase | 201623_s_at | 0.48 | 3.21 |
| TAS2R14 | taste receptor, type 2, member 14 | 235762_at | 0.48 | 3.67 |
| RBM25 | RNA binding motif protein 25 | 212030_at | 0.48 | 3.21 |
| ST7OT1 | ST7 overlapping transcript 1 (non-protein coding) | 1555912_at | 0.48 | 4.15 |
| MFAP5 | microfibrillar associated protein 5 | 209758_s_at | 0.48 | 5.82 |
| EFHA2 | EF-hand domain family, member A2 | 238458_at | 0.48 | 3.21 |
| LPP | LIM domain containing preferred translocation partner in lipoma | 224811_at | 0.48 | 2.45 |
| FEN1 | flap structure-specific endonuclease 1 | 204767_s_at | 0.48 | 7.84 |
| NAP1L1 | nucleosome assembly protein 1-like 1 | 1556121_at | 0.48 | 2.72 |
| LOC100508426 | hypothetical LOC100508426 | 230126_s_at | 0.48 | 4.15 |
| TMEM30A | transmembrane protein 30A | 222391_at | 0.48 | 2.45 |
| FAM122B | family with sequence similarity 122B | 225361_x_at | 0.48 | 3.21 |
| NAPEPLD | N-acyl phosphatidylethanolamine phospholipase D | 226041_at | 0.48 | 4.15 |
| FBXO3 | F-box protein 3 | 229955_at | 0.48 | 3.21 |
| MALAT1 | metastasis associated lung adenocarcinoma transcript 1 (non-protein coding) | 224558_s_at | 0.48 | 3.67 |
| NIPBL | Nipped-B homolog (Drosophila) | 212483_at | 0.48 | 2.72 |
| TMEM97 | transmembrane protein 97 | 212281_s_at | 0.47 | 4.93 |
| ZNF37BP | zinc finger protein 37B, pseudogene | 215358_x_at | 0.47 | 3.67 |
| DAZAP1 | DAZ associated protein 1 | 229813_x_at | 0.47 | 2.45 |
| CACNA2D1 | calcium channel, voltage-dependent, alpha 2/delta subunit 1 | 227623_at | 0.47 | 6.81 |
| GATAD2A | GATA zinc finger domain containing 2A | 234294_x_at | 0.47 | 2.45 |
| MAP9 | microtubule-associated protein 9 | 228423_at | 0.47 | 2.45 |
| GPCPD1 | glycerophosphocholine phosphodiesterase GDE1 homolog (S. cerevisiae) | 224826_at | 0.47 | 3.67 |
| PIK3R1 | phosphoinositide-3-kinase, regulatory subunit 1 (alpha) | 212239_at | 0.47 | 3.67 |
| SRSF10 | serine/arginine-rich splicing factor 10 | 204299_at | 0.47 | 2.45 |
| C8orf48 | chromosome 8 open reading frame 48 | 236634_at | 0.47 | 3.67 |
| ALDH1A3 | aldehyde dehydrogenase 1 family, member A3 | 203180_at | 0.47 | 4.15 |
| ANK3 | ankyrin 3, node of Ranvier (ankyrin G) | 206385_s_at | 0.47 | 3.67 |
| GPATCH2 | G patch domain containing 2 | 239768_x_at | 0.47 | 6.81 |
| ABCA8 | ATP-binding cassette, sub-family A (ABC1), member 8 | 204719_at | 0.47 | 4.15 |
| GANC | glucosidase, alpha; neutral C | 235714_at | 0.47 | 5.82 |
| CCNYL1 | cyclin Y-like 1 | 227280_s_at | 0.47 | 5.82 |
| KDM6A | lysine (K)-specific demethylase 6A | 203991_s_at | 0.47 | 3.21 |
| PTPRM | protein tyrosine phosphatase, receptor type, M | 1555578_at | 0.47 | 6.81 |
| NSL1 | NSL1, MIND kinetochore complex component, homolog (S. cerevisiae) | 209484_s_at | 0.47 | 2.06 |
| REV3L | REV3-like, catalytic subunit of DNA polymerase zeta (yeast) | 238736_at | 0.47 | 6.81 |
| KIAA0562 | KIAA0562 | 204075_s_at | 0.47 | 2.45 |
| PRSS12 | protease, serine, 12 (neurotrypsin, motopsin) | 213802_at | 0.47 | 9.00 |
| CXorf26 | chromosome X open reading frame 26 | 223294_at | 0.47 | 4.15 |
| SYNC | syncoilin, intermediate filament protein | 221276_s_at | 0.47 | 4.93 |
| MEG3 | maternally expressed 3 (non-protein coding) | 212732_at | 0.47 | 3.21 |
| USP47 | ubiquitin specific peptidase 47 | 221518_s_at | 0.47 | 2.45 |
| PHOSPHO2 | phosphatase, orphan 2 | 230434_at | 0.47 | 6.81 |
| LOC100505895 | hypothetical LOC100505895 | 229480_at | 0.47 | 3.67 |
| MED27 | mediator complex subunit 27 | 221598_s_at | 0.47 | 4.93 |
| CMC1 | COX assembly mitochondrial protein homolog (S. cerevisiae) | 228283_at | 0.47 | 2.72 |
| MALAT1 | metastasis associated lung adenocarcinoma transcript 1 (non-protein coding) | 223578_x_at | 0.47 | 5.82 |
| GPC6 | glypican 6 | 227059_at | 0.47 | 3.21 |
| UBE2D1 | ubiquitin-conjugating enzyme E2D 1 (UBC4/5 homolog, yeast) | 211764_s_at | 0.47 | 2.45 |
| ZCCHC6 | Zinc finger, CCHC domain containing 6 | 236155_at | 0.47 | 2.06 |
| EID1 | EP300 interacting inhibitor of differentiation 1 | 208670_s_at | 0.47 | 3.67 |
| PYROXD1 | pyridine nucleotide-disulphide oxidoreductase domain 1 | 213878_at | 0.47 | 4.15 |
| METAP2 | methionyl aminopeptidase 2 | 227993_at | 0.47 | 3.21 |
| CHM | choroideremia (Rab escort protein 1) | 227871_at | 0.47 | 2.72 |
| C15orf29 | chromosome 15 open reading frame 29 | 222745_s_at | 0.47 | 2.72 |
| PABPN1 | poly(A) binding protein, nuclear 1 | 201545_s_at | 0.47 | 2.06 |
| RRAGB | Ras-related GTP binding B | 205540_s_at | 0.47 | 2.45 |
| SOCS6 | suppressor of cytokine signaling 6 | 206020_at | 0.47 | 5.82 |
| DSEL | dermatan sulfate epimerase-like | 232235_at | 0.47 | 3.67 |
| MGAT2 | mannosyl (alpha-1,6-)-glycoprotein beta-1,2-N-acetylglucosaminyltransferase | 203102_s_at | 0.47 | 3.21 |
| GOPC | golgi-associated PDZ and coiled-coil motif containing | 225022_at | 0.47 | 2.45 |
| BAG5 | BCL2-associated athanogene 5 | 202984_s_at | 0.47 | 2.45 |
| ALKBH8 | alkB, alkylation repair homolog 8 (E. coli) | 235610_at | 0.47 | 6.81 |
| ACVR2A | activin A receptor, type IIA | 228416_at | 0.47 | 9.00 |
| CCPG1 | cell cycle progression 1 | 221156_x_at | 0.47 | 2.72 |
| ODZ2 | odz, odd Oz/ten-m homolog 2 (Drosophila) | 231867_at | 0.47 | 4.15 |
| FAM91A1 | family with sequence similarity 91, member A1 | 226294_x_at | 0.47 | 2.45 |
| KIAA1432 | KIAA1432 | 226222_at | 0.47 | 3.21 |
| MBNL3 | muscleblind-like 3 (Drosophila) | 229498_at | 0.47 | 9.00 |
| GOLIM4 | golgi integral membrane protein 4 | 238002_at | 0.47 | 3.67 |
| BMP2K | BMP2 inducible kinase | 59644_at | 0.47 | 6.81 |
| UBE2J1 | ubiquitin-conjugating enzyme E2, J1 (UBC6 homolog, yeast) | 217824_at | 0.47 | 3.21 |
| ACVR2A | activin A receptor, type IIA | 205327_s_at | 0.47 | 4.15 |
| C2orf84 | chromosome 2 open reading frame 84 | 231386_at | 0.47 | 5.82 |
| RFXAP | regulatory factor X-associated protein | 229431_at | 0.47 | 2.06 |
| RBM33 | RNA binding motif protein 33 | 1554095_at | 0.47 | 6.81 |
| EIF1 | eukaryotic translation initiation factor 1 | 228967_at | 0.47 | 4.93 |
| DHRS3 | dehydrogenase/reductase (SDR family) member 3 | 202481_at | 0.47 | 6.81 |
| PTEN | phosphatase and tensin homolog | 225363_at | 0.47 | 2.06 |
| TK2 | thymidine kinase 2, mitochondrial | 204276_at | 0.47 | 2.72 |
| LOC400657 | hypothetical LOC400657 | 226924_at | 0.47 | 4.15 |
| NCKAP5 | NCK-associated protein 5 | 239650_at | 0.47 | 5.82 |
| KTN1 | kinectin 1 (kinesin receptor) | 214709_s_at | 0.47 | 1.67 |
| DROSHA | drosha, ribonuclease type III | 218269_at | 0.47 | 2.45 |
| SMAD1 | SMAD family member 1 | 227798_at | 0.47 | 3.21 |
| XYLT1 | xylosyltransferase I | 213725_x_at | 0.47 | 3.21 |
| CALD1 | Caldesmon 1 | 235834_at | 0.47 | 3.67 |
| PDE4DIP | phosphodiesterase 4D interacting protein | 205872_x_at | 0.47 | 6.81 |
| SFRS2IP | Splicing factor, arginine/serine-rich 2, interacting protein | 1570507_at | 0.47 | 4.93 |
| PPM1A | protein phosphatase, Mg2+/Mn2+ dependent, 1A | 210407_at | 0.47 | 2.72 |
| SUMO1 | SMT3 suppressor of mif two 3 homolog 1 (S. cerevisiae) | 208762_at | 0.47 | 3.67 |
| ALG10B | asparagine-linked glycosylation 10, alpha-1,2-glucosyltransferase homolog B (yeast) | 228941_at | 0.47 | 2.45 |
| TMEM87B | transmembrane protein 87B | 225411_at | 0.47 | 2.06 |
| SMAD2 | SMAD family member 2 | 203075_at | 0.47 | 3.21 |
| NIPSNAP3A | nipsnap homolog 3A (C. elegans) | 224436_s_at | 0.47 | 4.15 |
| HP1BP3 | heterochromatin protein 1, binding protein 3 | 1554251_at | 0.47 | 2.45 |
| CCPG1 | cell cycle progression 1 | 214152_at | 0.47 | 4.15 |
| GPATCH4 | G patch domain containing 4 | 224632_at | 0.47 | 6.81 |
| TRAM1L1 | translocation associated membrane protein 1-like 1 | 244334_at | 0.47 | 3.67 |
| ZFX | zinc finger protein, X-linked | 214678_x_at | 0.47 | 3.21 |
| NUMB | numb homolog (Drosophila) | 230462_at | 0.46 | 4.93 |
| VHL | von Hippel-Lindau tumor suppressor | 1559227_s_at | 0.46 | 2.72 |
| ARID1B | AT rich interactive domain 1B (SWI1-like) | 225181_at | 0.46 | 1.67 |
| CHUK | conserved helix-loop-helix ubiquitous kinase | 209666_s_at | 0.46 | 4.93 |
| ZNF789 | zinc finger protein 789 | 1569194_at | 0.46 | 3.67 |
| CELF2 | CUGBP, Elav-like family member 2 | 202157_s_at | 0.46 | 5.82 |
| AFG3L2 | AFG3 ATPase family gene 3-like 2 (S. cerevisiae) | 1557820_at | 0.46 | 2.06 |
| TTBK2 | tau tubulin kinase 2 | 213922_at | 0.46 | 4.15 |
| PTN | pleiotrophin | 209465_x_at | 0.46 | 5.82 |
| SLCO3A1 | solute carrier organic anion transporter family, member 3A1 | 219229_at | 0.46 | 3.67 |
| ITGBL1 | Integrin, beta-like 1 (with EGF-like repeat domains) | 1557079_at | 0.46 | 4.15 |
| ATF7 | activating transcription factor 7 | 228829_at | 0.46 | 4.15 |
| LOC375295 | hypothetical LOC375295 | 228564_at | 0.46 | 5.82 |
| TBL1XR1 | transducin (beta)-like 1 X-linked receptor 1 | 235890_at | 0.46 | 3.21 |
| RBBP8 | retinoblastoma binding protein 8 | 203344_s_at | 0.46 | 3.21 |
| SNAPC3 | small nuclear RNA activating complex, polypeptide 3, 50kDa | 204001_at | 0.46 | 3.21 |
| ZNF326 | zinc finger protein 326 | 241720_at | 0.46 | 3.67 |
| APITD1 | apoptosis-inducing, TAF9-like domain 1 | 213454_at | 0.46 | 3.67 |
| PDS5A | PDS5, regulator of cohesion maintenance, homolog A (S. cerevisiae) | 213984_at | 0.46 | 3.21 |
| NUP98 | nucleoporin 98kDa | 210793_s_at | 0.46 | 7.84 |
| UBE2B | ubiquitin-conjugating enzyme E2B (RAD6 homolog) | 202334_s_at | 0.46 | 2.72 |
| TNRC6B | trinucleotide repeat containing 6B | 230779_at | 0.46 | 2.45 |
| PRICKLE1 | prickle homolog 1 (Drosophila) | 230708_at | 0.46 | 4.15 |
| TPP2 | tripeptidyl peptidase II | 203374_s_at | 0.46 | 4.15 |
| RANBP2 /// RGPD1 /// RGPD2 /// RGPD3 /// RGPD4 /// RGPD5 /// RGPD6 /// RGPD8 | RAN binding protein 2 /// RANBP2-like and GRIP domain containing 1 /// RANBP2-like and GRIP domain containing 2 /// RANBP2-like and GRIP domain containing 3 /// RANBP2-like and GRIP domain containing 4 /// RANBP2-like and GRIP domain containing 5 /// RANBP2-like and GRIP domain containing 6 /// RANBP2-like and GRIP domain containing 8 | 242712_x_at | 0.46 | 4.93 |
| C9orf82 | chromosome 9 open reading frame 82 | 231995_at | 0.46 | 3.21 |
| FHL1 | four and a half LIM domains 1 | 201540_at | 0.46 | 3.67 |
| RAPGEF4 | Rap guanine nucleotide exchange factor (GEF) 4 | 205651_x_at | 0.46 | 3.67 |
| FTSJD1 | FtsJ methyltransferase domain containing 1 | 222811_at | 0.46 | 2.06 |
| BRIP1 | BRCA1 interacting protein C-terminal helicase 1 | 235609_at | 0.46 | 4.93 |
| MYPN | myopalladin | 235367_at | 0.46 | 4.93 |
| AGK | acylglycerol kinase | 218568_at | 0.46 | 2.45 |
| C13orf33 | chromosome 13 open reading frame 33 | 227058_at | 0.46 | 4.15 |
| C10orf72 | chromosome 10 open reading frame 72 | 244057_s_at | 0.46 | 3.67 |
| FAHD1 | fumarylacetoacetate hydrolase domain containing 1 | 226767_s_at | 0.46 | 2.06 |
| RPP40 | ribonuclease P/MRP 40kDa subunit | 213427_at | 0.46 | 6.81 |
| C3orf70 | chromosome 3 open reading frame 70 | 242447_at | 0.46 | 4.15 |
| BBS10 | Bardet-Biedl syndrome 10 | 219487_at | 0.46 | 4.93 |
| JUN | jun proto-oncogene | 201464_x_at | 0.46 | 4.15 |
| PLAGL1 | pleiomorphic adenoma gene-like 1 | 207943_x_at | 0.46 | 1.67 |
| HHIP | hedgehog interacting protein | 230135_at | 0.46 | 4.15 |
| PDE4DIP | phosphodiesterase 4D interacting protein | 209700_x_at | 0.46 | 6.81 |
| CEPT1 | choline/ethanolamine phosphotransferase 1 | 219375_at | 0.46 | 2.06 |
| SRGAP2P1 | SLIT-ROBO Rho GTPase activating protein 2 pseudogene 1 | 1568955_at | 0.46 | 3.67 |
| MEGF6 | multiple EGF-like-domains 6 | 226869_at | 0.46 | 4.93 |
| RPUSD3 | RNA pseudouridylate synthase domain containing 3 | 1566603_s_at | 0.46 | 4.15 |
| PABPC1L | poly(A) binding protein, cytoplasmic 1-like | 226670_s_at | 0.46 | 4.93 |
| TRDMT1 | tRNA aspartic acid methyltransferase 1 | 206308_at | 0.46 | 3.67 |
| FUBP3 | far upstream element (FUSE) binding protein 3 | 212824_at | 0.46 | 3.21 |
| NBN | nibrin | 202906_s_at | 0.46 | 3.67 |
| RNF24 | ring finger protein 24 | 204669_s_at | 0.46 | 3.67 |
| NEK1 | NIMA (never in mitosis gene a)-related kinase 1 | 213328_at | 0.46 | 1.44 |
| MGC24103 | hypothetical MGC24103 | 232568_at | 0.46 | 3.67 |
| EXOC5 | exocyst complex component 5 | 228418_at | 0.46 | 2.45 |
| SET | SET nuclear oncogene | 210231_x_at | 0.46 | 1.67 |
| HIGD1A | HIG1 hypoxia inducible domain family, member 1A | 242317_at | 0.46 | 4.93 |
| PSIP1 | PC4 and SFRS1 interacting protein 1 | 210758_at | 0.46 | 2.72 |
| NEXN | nexilin (F actin binding protein) | 226103_at | 0.46 | 3.21 |
| ITGBL1 | Integrin, beta-like 1 (with EGF-like repeat domains) | 231993_at | 0.46 | 4.15 |
| RRP15 | ribosomal RNA processing 15 homolog (S. cerevisiae) | 219037_at | 0.46 | 9.00 |
| FBXO9 | F-box protein 9 | 1566509_s_at | 0.46 | 2.72 |
| DLST | dihydrolipoamide S-succinyltransferase (E2 component of 2-oxo-glutarate complex) | 215210_s_at | 0.46 | 2.45 |
| MPHOSPH8 | M-phase phosphoprotein 8 | 225041_at | 0.46 | 2.06 |
| ATP9B | ATPase, class II, type 9B | 214934_at | 0.46 | 3.21 |
| FNBP4 | Formin binding protein 4 | 242472_x_at | 0.46 | 3.21 |
| SATB1 | SATB homeobox 1 | 241365_at | 0.46 | 7.84 |
| PRKAA1 | protein kinase, AMP-activated, alpha 1 catalytic subunit | 225984_at | 0.46 | 3.67 |
| MYST4 | MYST histone acetyltransferase (monocytic leukemia) 4 | 212462_at | 0.45 | 2.45 |
| GK5 | glycerol kinase 5 (putative) | 238121_at | 0.45 | 2.06 |
| PRDM16 | PR domain containing 16 | 232424_at | 0.45 | 4.15 |
| DSP | desmoplakin | 200606_at | 0.45 | 4.93 |
| KRIT1 | KRIT1, ankyrin repeat containing | 216713_at | 0.45 | 2.72 |
| GALNT5 | UDP-N-acetyl-alpha-D-galactosamine:polypeptide N-acetylgalactosaminyltransferase 5 (GalNAc-T5) | 240390_at | 0.45 | 6.81 |
| SACS | spastic ataxia of Charlevoix-Saguenay (sacsin) | 213262_at | 0.45 | 2.72 |
| CSPP1 | centrosome and spindle pole associated protein 1 | 220072_at | 0.45 | 3.21 |
| ANKDD1A | ankyrin repeat and death domain containing 1A | 229497_at | 0.45 | 4.93 |
| FBXW7 | F-box and WD repeat domain containing 7 | 229419_at | 0.45 | 2.06 |
| KBTBD11 | kelch repeat and BTB (POZ) domain containing 11 | 204301_at | 0.45 | 3.21 |
| USP48 | ubiquitin specific peptidase 48 | 225925_s_at | 0.45 | 2.06 |
| AURKAIP1 | aurora kinase A interacting protein 1 | 225555_x_at | 0.45 | 1.44 |
| MSL2 | male-specific lethal 2 homolog (Drosophila) | 218733_at | 0.45 | 2.45 |
| FRMD8 | FERM domain containing 8 | 227964_at | 0.45 | 4.15 |
| PAPSS2 | 3'-phosphoadenosine 5'-phosphosulfate synthase 2 | 203060_s_at | 0.45 | 1.67 |
| C7orf55 | chromosome 7 open reading frame 55 | 226781_at | 0.45 | 4.93 |
| BCLAF1 | BCL2-associated transcription factor 1 | 239897_at | 0.45 | 2.45 |
| RSF1 | remodeling and spacing factor 1 | 223818_s_at | 0.45 | 3.67 |
| HUS1 | HUS1 checkpoint homolog (S. pombe) | 217618_x_at | 0.45 | 4.93 |
| ENOSF1 | enolase superfamily member 1 | 204142_at | 0.45 | 1.67 |
| ZXDB | zinc finger, X-linked, duplicated B | 228005_at | 0.45 | 3.21 |
| LOC284757 | hypothetical protein LOC284757 | 236846_at | 0.45 | 6.81 |
| NCKAP1 | NCK-associated protein 1 | 207738_s_at | 0.45 | 2.45 |
| TBL1XR1 | transducin (beta)-like 1 X-linked receptor 1 | 222633_at | 0.45 | 2.72 |
| MRPL41 | mitochondrial ribosomal protein L41 | 230034_x_at | 0.45 | 3.21 |
| DPY19L4 | dpy-19-like 4 (C. elegans) | 1556613_s_at | 0.45 | 2.06 |
| IKZF4 | IKAROS family zinc finger 4 (Eos) | 229752_at | 0.45 | 2.72 |
| ADSS | adenylosuccinate synthase | 221761_at | 0.45 | 2.45 |
| NHLRC2 | NHL repeat containing 2 | 219353_at | 0.45 | 2.06 |
| LOC339803 | hypothetical LOC339803 | 227941_at | 0.45 | 3.67 |
| RBM45 | RNA binding motif protein 45 | 228578_at | 0.45 | 4.15 |
| CAP2 | CAP, adenylate cyclase-associated protein, 2 (yeast) | 212554_at | 0.45 | 3.21 |
| LANCL2 | LanC lantibiotic synthetase component C-like 2 (bacterial) | 222561_at | 0.45 | 3.67 |
| DDX31 | DEAD (Asp-Glu-Ala-Asp) box polypeptide 31 | 235436_at | 0.45 | 3.21 |
| NR2F2 | nuclear receptor subfamily 2, group F, member 2 | 209120_at | 0.45 | 3.67 |
| ZNF704 | zinc finger protein 704 | 223366_at | 0.45 | 2.72 |
| KBTBD6 | kelch repeat and BTB (POZ) domain containing 6 | 1553111_a_at | 0.45 | 3.21 |
| IPW | imprinted in Prader-Willi syndrome (non-protein coding) | 221974_at | 0.45 | 1.67 |
| SLC16A9 | solute carrier family 16, member 9 (monocarboxylic acid transporter 9) | 227506_at | 0.45 | 4.15 |
| LOC100130219 | hypothetical LOC100130219 | 236042_at | 0.45 | 4.93 |
| MED13 | Mediator complex subunit 13 | 244611_at | 0.45 | 4.15 |
| TOMM40L | translocase of outer mitochondrial membrane 40 homolog (yeast)-like | 226059_at | 0.45 | 4.93 |
| MALAT1 | metastasis associated lung adenocarcinoma transcript 1 (non-protein coding) | 231735_s_at | 0.45 | 3.67 |
| YTHDC2 | YTH domain containing 2 | 213077_at | 0.45 | 1.67 |
| UBE3B | ubiquitin protein ligase E3B | 213822_s_at | 0.45 | 6.81 |
| SLCO3A1 | solute carrier organic anion transporter family, member 3A1 | 229776_at | 0.45 | 2.06 |
| IK /// TMCO6 | IK cytokine, down-regulator of HLA II /// transmembrane and coiled-coil domains 6 | 213550_s_at | 0.45 | 4.15 |
| BOLA2 /// LOC440354 /// LOC595101 | bolA homolog 2 (E. coli) /// PI-3-kinase-related kinase SMG-1 pseudogene /// PI-3-kinase-related kinase SMG-1 pseudogene | 210396_s_at | 0.45 | 2.72 |
| TRIM33 | tripartite motif-containing 33 | 210266_s_at | 0.45 | 1.44 |
| SFRS18 | splicing factor, arginine/serine-rich 18 | 225507_at | 0.45 | 2.45 |
| RAP2B | RAP2B, member of RAS oncogene family | 213923_at | 0.45 | 2.45 |
| PSMD7 | Proteasome (prosome, macropain) 26S subunit, non-ATPase, 7 | 238738_at | 0.45 | 3.67 |
| LOC100190939 | hypothetical LOC100190939 | 228913_at | 0.45 | 2.45 |
| NAA25 | N(alpha)-acetyltransferase 25, NatB auxiliary subunit | 227245_at | 0.45 | 2.06 |
| AKAP12 | A kinase (PRKA) anchor protein 12 | 227529_s_at | 0.45 | 5.82 |
| TRAF5 | TNF receptor-associated factor 5 | 204352_at | 0.45 | 4.15 |
| C3orf38 | chromosome 3 open reading frame 38 | 229174_at | 0.45 | 1.67 |
| TRIM2 | tripartite motif-containing 2 | 202341_s_at | 0.45 | 3.21 |
| SMARCE1 | SWI/SNF related, matrix associated, actin dependent regulator of chromatin, subfamily e, member 1 | 229511_at | 0.45 | 4.93 |
| METAP2 | methionyl aminopeptidase 2 | 213899_at | 0.45 | 3.21 |
| DLG5 | discs, large homolog 5 (Drosophila) | 201681_s_at | 0.45 | 2.45 |
| ATF6B /// TNXB | activating transcription factor 6 beta /// tenascin XB | 211611_s_at | 0.45 | 2.06 |
| LCORL | ligand dependent nuclear receptor corepressor-like | 235970_at | 0.45 | 3.21 |
| PEX13 | peroxisomal biogenesis factor 13 | 1556009_at | 0.45 | 2.06 |
| RSU1 | Ras suppressor protein 1 | 230490_x_at | 0.45 | 3.67 |
| ABCC10 | ATP-binding cassette, sub-family C (CFTR/MRP), member 10 | 213485_s_at | 0.45 | 3.21 |
| SVIP | small VCP/p97-interacting protein | 230005_at | 0.45 | 4.15 |
| TRA2A | transformer 2 alpha homolog (Drosophila) | 204658_at | 0.45 | 2.45 |
| OIP5 | Opa interacting protein 5 | 213599_at | 0.45 | 6.81 |
| LARP1B | La ribonucleoprotein domain family, member 1B | 242153_at | 0.45 | 3.67 |
| MRPS16 | mitochondrial ribosomal protein S16 | 222499_at | 0.45 | 3.21 |
| ANAPC7 | anaphase promoting complex subunit 7 | 225521_at | 0.45 | 5.82 |
| PROCR | protein C receptor, endothelial | 203650_at | 0.45 | 5.82 |
| LYAR | Ly1 antibody reactive homolog (mouse) | 223413_s_at | 0.45 | 3.67 |
| MOCS2 | molybdenum cofactor synthesis 2 | 236208_at | 0.45 | 3.67 |
| KTN1 | kinectin 1 (kinesin receptor) | 200914_x_at | 0.45 | 1.67 |
| PIGM | phosphatidylinositol glycan anchor biosynthesis, class M | 235168_at | 0.45 | 2.72 |
| RPL27A | Ribosomal protein L27a | 212044_s_at | 0.45 | 2.06 |
| KIAA1731 | KIAA1731 | 229878_at | 0.45 | 2.45 |
| C14orf118 | Chromosome 14 open reading frame 118 | 231970_at | 0.45 | 3.67 |
| FAM122B | family with sequence similarity 122B | 222673_x_at | 0.45 | 3.21 |
| NCRNA00292 | non-protein coding RNA 292 | 235126_at | 0.45 | 4.93 |
| MED17 | mediator complex subunit 17 | 232483_at | 0.45 | 9.00 |
| ZDHHC2 | zinc finger, DHHC-type containing 2 | 244779_at | 0.45 | 5.82 |
| SRD5A1 | steroid-5-alpha-reductase, alpha polypeptide 1 (3-oxo-5 alpha-steroid delta 4-dehydrogenase alpha 1) | 204675_at | 0.45 | 2.72 |
| FAT4 | FAT tumor suppressor homolog 4 (Drosophila) | 219427_at | 0.45 | 2.72 |
| BRWD3 | bromodomain and WD repeat domain containing 3 | 1553252_a_at | 0.44 | 4.15 |
| NIPBL | Nipped-B homolog (Drosophila) | 213918_s_at | 0.44 | 3.67 |
| DDX46 | DEAD (Asp-Glu-Ala-Asp) box polypeptide 46 | 202462_s_at | 0.44 | 3.67 |
| NUCKS1 | nuclear casein kinase and cyclin-dependent kinase substrate 1 | 229353_s_at | 0.44 | 2.45 |
| CTNNB1 | catenin (cadherin-associated protein), beta 1, 88kDa | 201533_at | 0.44 | 1.67 |
| DNAJC21 | DnaJ (Hsp40) homolog, subfamily C, member 21 | 238336_s_at | 0.44 | 3.67 |
| SH3KBP1 | SH3-domain kinase binding protein 1 | 1554168_a_at | 0.44 | 2.72 |
| YTHDC1 | YTH domain containing 1 | 214814_at | 0.44 | 2.45 |
| C15orf5 | chromosome 15 open reading frame 5 | 208109_s_at | 0.44 | 9.00 |
| CDC14B | CDC14 cell division cycle 14 homolog B (S. cerevisiae) | 208022_s_at | 0.44 | 2.45 |
| HOOK3 | hook homolog 3 (Drosophila) | 236192_at | 0.44 | 1.44 |
| CDC42SE1 | CDC42 small effector 1 | 218157_x_at | 0.44 | 1.44 |
| ILKAP | integrin-linked kinase-associated serine/threonine phosphatase | 221548_s_at | 0.44 | 2.72 |
| ATXN1L | ataxin 1-like | 227373_at | 0.44 | 2.06 |
| CDC23 | cell division cycle 23 homolog (S. cerevisiae) | 223651_x_at | 0.44 | 3.67 |
| EIF2C4 | Eukaryotic translation initiation factor 2C, 4 | 227930_at | 0.44 | 2.72 |
| FAM3C | family with sequence similarity 3, member C | 236316_at | 0.44 | 9.00 |
| LOC100509635 | hypothetical LOC100509635 | 226865_at | 0.44 | 3.21 |
| BOLA2 | BolA homolog 2 (E. coli) | 231500_s_at | 0.44 | 4.15 |
| SCD | stearoyl-CoA desaturase (delta-9-desaturase) | 223839_s_at | 0.44 | 9.00 |
| HGF | hepatocyte growth factor (hepapoietin A; scatter factor) | 210755_at | 0.44 | 6.81 |
| DDX27 | DEAD (Asp-Glu-Ala-Asp) box polypeptide 27 | 215693_x_at | 0.44 | 3.67 |
| MIPOL1 | mirror-image polydactyly 1 | 244246_at | 0.44 | 3.67 |
| RAP1A | RAP1A, member of RAS oncogene family | 202362_at | 0.44 | 2.72 |
| DSG2 | desmoglein 2 | 217901_at | 0.44 | 3.67 |
| CAB39L | calcium binding protein 39-like | 225915_at | 0.44 | 3.67 |
| SERPINE2 | Serpin peptidase inhibitor, clade E (nexin, plasminogen activator inhibitor type 1), member 2 | 227487_s_at | 0.44 | 4.93 |
| AKAP12 | A kinase (PRKA) anchor protein 12 | 210517_s_at | 0.44 | 4.93 |
| SRSF10 | serine/arginine-rich splicing factor 10 | 225348_at | 0.44 | 2.72 |
| GCLM | glutamate-cysteine ligase, modifier subunit | 234986_at | 0.44 | 2.45 |
| PEAR1 | platelet endothelial aggregation receptor 1 | 228618_at | 0.44 | 2.06 |
| ZNF521 | zinc finger protein 521 | 226677_at | 0.44 | 5.82 |
| LPHN2 | latrophilin 2 | 206953_s_at | 0.44 | 3.21 |
| FAM36A | family with sequence similarity 36, member A | 224824_at | 0.44 | 4.93 |
| PRKDC | protein kinase, DNA-activated, catalytic polypeptide | 208694_at | 0.44 | 3.67 |
| LOC285359 /// PDCL3 | phosducin-like 3 pseudogene /// phosducin-like 3 | 219043_s_at | 0.44 | 4.93 |
| KLF7 | Kruppel-like factor 7 (ubiquitous) | 204334_at | 0.44 | 3.21 |
| PTGER4 | prostaglandin E receptor 4 (subtype EP4) | 204897_at | 0.44 | 3.21 |
| RPL27A | ribosomal protein L27a | 223707_at | 0.44 | 4.93 |
| NEK4 | NIMA (never in mitosis gene a)-related kinase 4 | 204634_at | 0.44 | 1.67 |
| ZDHHC21 | zinc finger, DHHC-type containing 21 | 229240_at | 0.44 | 2.72 |
| HERC4 | hect domain and RLD 4 | 232026_at | 0.44 | 2.72 |
| RSF1 | remodeling and spacing factor 1 | 222540_s_at | 0.44 | 1.25 |
| LRRC34 | leucine rich repeat containing 34 | 236918_s_at | 0.44 | 3.67 |
| USP34 | ubiquitin specific peptidase 34 | 212980_at | 0.44 | 4.15 |
| NUDT12 | nudix (nucleoside diphosphate linked moiety X)-type motif 12 | 223535_at | 0.44 | 3.21 |
| TJP1 | tight junction protein 1 (zona occludens 1) | 202011_at | 0.44 | 2.45 |
| CBWD1 /// CBWD2 /// CBWD3 /// CBWD6 | COBW domain containing 1 /// COBW domain containing 2 /// COBW domain containing 3 /// COBW domain containing 6 | 229804_x_at | 0.44 | 2.45 |
| C14orf126 | chromosome 14 open reading frame 126 | 1553801_a_at | 0.44 | 4.93 |
| UBE2W | ubiquitin-conjugating enzyme E2W (putative) | 218521_s_at | 0.44 | 1.67 |
| DOCK9 | dedicator of cytokinesis 9 | 212538_at | 0.44 | 2.72 |
| CDK13 | cyclin-dependent kinase 13 | 210965_x_at | 0.44 | 5.82 |
| PCMTD2 | protein-L-isoaspartate (D-aspartate) O-methyltransferase domain containing 2 | 212406_s_at | 0.44 | 1.25 |
| NOX4 | NADPH oxidase 4 | 219773_at | 0.44 | 6.81 |
| WDR1 | WD repeat domain 1 | 240282_at | 0.44 | 5.82 |
| GLUD1 | glutamate dehydrogenase 1 | 200947_s_at | 0.44 | 2.06 |
| NBPF1 | neuroblastoma breakpoint family, member 1 | 236273_at | 0.44 | 4.15 |
| RBMS1 | RNA binding motif, single stranded interacting protein 1 | 215127_s_at | 0.44 | 1.00 |
| FOXO1 | forkhead box O1 | 202723_s_at | 0.44 | 4.15 |
| KIAA1109 | KIAA1109 | 212779_at | 0.43 | 2.45 |
| AHNAK2 | AHNAK nucleoprotein 2 | 212992_at | 0.43 | 5.82 |
| CDKN2B | cyclin-dependent kinase inhibitor 2B (p15, inhibits CDK4) | 236313_at | 0.43 | 9.00 |
| LOC339290 | hypothetical LOC339290 | 228160_at | 0.43 | 5.82 |
| NKAPL | NFKB activating protein-like | 229340_at | 0.43 | 3.21 |
| KBTBD4 /// PTPMT1 | kelch repeat and BTB (POZ) domain containing 4 /// protein tyrosine phosphatase, mitochondrial 1 | 218570_at | 0.43 | 1.44 |
| RIMKLB | ribosomal modification protein rimK-like family member B | 225978_at | 0.43 | 3.21 |
| SERPINB6 | serpin peptidase inhibitor, clade B (ovalbumin), member 6 | 1556950_s_at | 0.43 | 6.81 |
| PYROXD1 | pyridine nucleotide-disulphide oxidoreductase domain 1 | 219802_at | 0.43 | 4.15 |
| CSAD | cysteine sulfinic acid decarboxylase | 221139_s_at | 0.43 | 2.72 |
| TYMP | thymidine phosphorylase | 217497_at | 0.43 | 5.82 |
| MYCBP2 | MYC binding protein 2 | 201960_s_at | 0.43 | 1.44 |
| TACC1 | transforming, acidic coiled-coil containing protein 1 | 1554690_a_at | 0.43 | 2.45 |
| BMI1 | BMI1 polycomb ring finger oncogene | 202265_at | 0.43 | 1.00 |
| MAP9 | microtubule-associated protein 9 | 239415_at | 0.43 | 2.45 |
| IFNAR2 | interferon (alpha, beta and omega) receptor 2 | 204786_s_at | 0.43 | 2.72 |
| PRKAG2 | Protein kinase, AMP-activated, gamma 2 non-catalytic subunit | 215231_at | 0.43 | 3.21 |
| TPBG | trophoblast glycoprotein | 239903_at | 0.43 | 3.21 |
| MUC1 | mucin 1, cell surface associated | 213693_s_at | 0.43 | 4.93 |
| HMGCS1 | 3-hydroxy-3-methylglutaryl-CoA synthase 1 (soluble) | 221750_at | 0.43 | 2.72 |
| SERF1A /// SERF1B | small EDRK-rich factor 1A (telomeric) /// small EDRK-rich factor 1B (centromeric) | 219982_s_at | 0.43 | 3.21 |
| ZDHHC21 | zinc finger, DHHC-type containing 21 | 235068_at | 0.43 | 3.67 |
| VCAN | versican | 204619_s_at | 0.43 | 3.21 |
| HMCN1 | hemicentin 1 | 235944_at | 0.43 | 3.67 |
| TEK | TEK tyrosine kinase, endothelial | 206702_at | 0.43 | 9.00 |
| NCRNA00183 | non-protein coding RNA 183 | 1554447_at | 0.43 | 2.06 |
| C16orf52 | Chromosome 16 open reading frame 52 | 244835_at | 0.43 | 5.82 |
| TSC22D1 | TSC22 domain family, member 1 | 235315_at | 0.43 | 4.15 |
| KIAA0232 | KIAA0232 | 232366_at | 0.43 | 2.45 |
| DPY19L3 | dpy-19-like 3 (C. elegans) | 225633_at | 0.43 | 2.06 |
| HSPA1A | heat shock 70kDa protein 1A | 200799_at | 0.43 | 5.82 |
| KCND3 | potassium voltage-gated channel, Shal-related subfamily, member 3 | 213832_at | 0.43 | 4.15 |
| C17orf91 | chromosome 17 open reading frame 91 | 214696_at | 0.43 | 1.67 |
| C1orf43 | chromosome 1 open reading frame 43 | 1555225_at | 0.43 | 3.67 |
| PGK1 | Phosphoglycerate kinase 1 | 217383_at | 0.43 | 3.21 |
| TSPYL4 | TSPY-like 4 | 212928_at | 0.43 | 1.25 |
| HCG11 | HLA complex group 11 | 1557167_at | 0.43 | 4.15 |
| ZNF506 | zinc finger protein 506 | 238493_at | 0.43 | 1.44 |
| FLJ90757 | hypothetical LOC440465 | 1566558_x_at | 0.43 | 3.21 |
| KCNT2 | potassium channel, subfamily T, member 2 | 244455_at | 0.43 | 9.00 |
| PPIP5K2 | diphosphoinositol pentakisphosphate kinase 2 | 203253_s_at | 0.43 | 2.06 |
| WDR4 | WD repeat domain 4 | 241937_s_at | 0.42 | 6.81 |
| LYRM7 | Lyrm7 homolog (mouse) | 228841_at | 0.42 | 2.06 |
| LOC100132247 /// LOC348162 /// LOC613037 /// LOC728888 /// NPIPL3 | similar to Uncharacterized protein KIAA0220 /// hypothetical protein 348162 /// nuclear pore complex interacting protein pseudogene /// nuclear pore complex-interacting protein-like 3-like /// nuclear pore complex interacting protein-like 3 | 211996_s_at | 0.42 | 3.21 |
| NEDD1 | neural precursor cell expressed, developmentally down-regulated 1 | 1560116_a_at | 0.42 | 1.25 |
| C4orf32 | chromosome 4 open reading frame 32 | 227856_at | 0.42 | 2.45 |
| CHD2 | Chromodomain helicase DNA binding protein 2 | 230156_x_at | 0.42 | 2.06 |
| STAG1 | stromal antigen 1 | 202294_at | 0.42 | 2.72 |
| RP2 | retinitis pigmentosa 2 (X-linked recessive) | 205191_at | 0.42 | 2.45 |
| SET | SET nuclear oncogene | 200630_x_at | 0.42 | 1.25 |
| RSBN1L | round spermatid basic protein 1-like | 232221_x_at | 0.42 | 2.45 |
| MTMR6 | myotubularin related protein 6 | 228789_at | 0.42 | 2.06 |
| HIRA | HIR histone cell cycle regulation defective homolog A (S. cerevisiae) | 217427_s_at | 0.42 | 3.67 |
| CLIC3 | chloride intracellular channel 3 | 219529_at | 0.42 | 7.84 |
| NANOS1 | nanos homolog 1 (Drosophila) | 228523_at | 0.42 | 6.81 |
| HNRNPD | heterogeneous nuclear ribonucleoprotein D (AU-rich element RNA binding protein 1, 37kDa) | 221480_at | 0.42 | 3.67 |
| ACADSB | acyl-CoA dehydrogenase, short/branched chain | 226030_at | 0.42 | 2.45 |
| KLHDC5 | kelch domain containing 5 | 225961_at | 0.42 | 1.67 |
| ACER3 | alkaline ceramidase 3 | 227776_at | 0.42 | 1.67 |
| DMAP1 | DNA methyltransferase 1 associated protein 1 | 224163_s_at | 0.42 | 3.67 |
| BRP44L | brain protein 44-like | 218024_at | 0.42 | 3.67 |
| KCNQ1OT1 | KCNQ1 overlapping transcript 1 (non-protein coding) | 243435_at | 0.42 | 4.93 |
| ZCCHC8 | zinc finger, CCHC domain containing 8 | 218478_s_at | 0.42 | 1.44 |
| LOC100132832 | postmeiotic segregation increased 2-like 5-like | 1557996_at | 0.42 | 3.67 |
| USP4 | ubiquitin specific peptidase 4 (proto-oncogene) | 202682_s_at | 0.42 | 0.86 |
| PHF17 | PHD finger protein 17 | 225820_at | 0.42 | 4.15 |
| VEZT | vezatin, adherens junctions transmembrane protein | 223089_at | 0.42 | 1.25 |
| ENTPD1 | Ectonucleoside triphosphate diphosphohydrolase 1 | 243111_at | 0.42 | 3.21 |
| ZNF643 | zinc finger protein 643 | 207219_at | 0.42 | 4.93 |
| FAM21A /// FAM21B /// FAM21C /// FAM21D | family with sequence similarity 21, member A /// family with sequence similarity 21, member B /// family with sequence similarity 21, member C /// family with sequence similarity 21, member D | 212929_s_at | 0.42 | 2.06 |
| SFPQ | splicing factor proline/glutamine-rich | 201586_s_at | 0.42 | 4.93 |
| HMGCR | 3-hydroxy-3-methylglutaryl-CoA reductase | 202540_s_at | 0.42 | 2.06 |
| HBS1L | HBS1-like (S. cerevisiae) | 209314_s_at | 0.42 | 2.45 |
| KIAA1632 | KIAA1632 | 232030_at | 0.42 | 5.82 |
| KIAA1908 | hypothetical LOC114796 | 1561666_a_at | 0.42 | 3.67 |
| LOC284926 | hypothetical protein LOC284926 | 1556064_at | 0.42 | 3.67 |
| C6orf62 | chromosome 6 open reading frame 62 | 213875_x_at | 0.42 | 1.25 |
| GABPA | GA binding protein transcription factor, alpha subunit 60kDa | 227428_at | 0.42 | 0.73 |
| FANCI | Fanconi anemia, complementation group I | 213007_at | 0.42 | 5.82 |
| SNRPE | small nuclear ribonucleoprotein polypeptide E | 215450_at | 0.42 | 2.06 |
| CRISPLD1 | cysteine-rich secretory protein LCCL domain containing 1 | 223475_at | 0.42 | 3.67 |
| ZNF83 | zinc finger protein 83 | 236429_at | 0.42 | 1.67 |
| TUBB2A | tubulin, beta 2A | 204141_at | 0.42 | 6.81 |
| PHTF1 | putative homeodomain transcription factor 1 | 235844_at | 0.42 | 4.15 |
| LOC650794 | hypothetical LOC650794 | 236837_x_at | 0.42 | 1.44 |
| CHML | choroideremia-like (Rab escort protein 2) | 226350_at | 0.42 | 1.00 |
| MGC12488 | hypothetical protein MGC12488 | 211386_at | 0.42 | 3.21 |
| EEF1D | eukaryotic translation elongation factor 1 delta (guanine nucleotide exchange protein) | 214395_x_at | 0.42 | 1.67 |
| IGF2R | insulin-like growth factor 2 receptor | 201392_s_at | 0.42 | 1.44 |
| RNFT1 | ring finger protein, transmembrane 1 | 227268_at | 0.42 | 2.06 |
| FLJ10038 | hypothetical protein FLJ10038 | 236164_at | 0.42 | 2.72 |
| TMEFF2 | transmembrane protein with EGF-like and two follistatin-like domains 2 | 224321_at | 0.42 | 2.45 |
| FAM164A | family with sequence similarity 164, member A | 241808_at | 0.42 | 2.45 |
| RAB27A | RAB27A, member RAS oncogene family | 235766_x_at | 0.42 | 0.86 |
| BAT2L2 | HLA-B associated transcript 2-like 2 | 211944_at | 0.42 | 1.67 |
| ZNF642 | zinc finger protein 642 | 1569107_s_at | 0.42 | 2.06 |
| LOC100509749 | Golgin subfamily A member 8B-like | 213650_at | 0.42 | 2.72 |
| PHACTR2 | phosphatase and actin regulator 2 | 227947_at | 0.42 | 2.45 |
| AP4S1 | Adaptor-related protein complex 4, sigma 1 subunit | 235647_at | 0.42 | 2.72 |
| RBM3 | RNA binding motif (RNP1, RRM) protein 3 | 208319_s_at | 0.42 | 3.67 |
| SRSF2IP | serine/arginine-rich splicing factor 2, interacting protein | 213850_s_at | 0.41 | 0.86 |
| ZNF268 | zinc finger protein 268 | 209989_at | 0.41 | 2.45 |
| SPAST | spastin | 209748_at | 0.41 | 1.67 |
| FBXO5 | F-box protein 5 | 218875_s_at | 0.41 | 5.82 |
| MAGI1 | membrane associated guanylate kinase, WW and PDZ domain containing 1 | 225465_at | 0.41 | 1.67 |
| ODF2L | outer dense fiber of sperm tails 2-like | 231909_x_at | 0.41 | 2.06 |
| C10orf18 | chromosome 10 open reading frame 18 | 244165_at | 0.41 | 4.15 |
| MAGT1 | magnesium transporter 1 | 210596_at | 0.41 | 2.45 |
| STK4 | serine/threonine kinase 4 | 223746_at | 0.41 | 9.00 |
| MPP7 | membrane protein, palmitoylated 7 (MAGUK p55 subfamily member 7) | 238778_at | 0.41 | 6.81 |
| SYNJ2BP | synaptojanin 2 binding protein | 235722_at | 0.41 | 2.45 |
| PLAC9 | placenta-specific 9 | 227419_x_at | 0.41 | 2.06 |
| FAHD1 | fumarylacetoacetate hydrolase domain containing 1 | 227960_s_at | 0.41 | 2.06 |
| PHACTR2 | phosphatase and actin regulator 2 | 204048_s_at | 0.41 | 2.72 |
| PPM1A | protein phosphatase, Mg2+/Mn2+ dependent, 1A | 229027_at | 0.41 | 1.67 |
| HNRNPD | heterogeneous nuclear ribonucleoprotein D (AU-rich element RNA binding protein 1, 37kDa) | 200073_s_at | 0.41 | 3.21 |
| ATP11B | ATPase, class VI, type 11B | 212536_at | 0.41 | 2.06 |
| C5orf24 | chromosome 5 open reading frame 24 | 1553107_s_at | 0.41 | 0.86 |
| RUFY3 | RUN and FYVE domain containing 3 | 213939_s_at | 0.41 | 2.45 |
| SRSF5 | serine/arginine-rich splicing factor 5 | 203380_x_at | 0.41 | 1.25 |
| SFXN3 | Sideroflexin 3 | 1559993_at | 0.41 | 4.15 |
| RUFY2 | RUN and FYVE domain containing 2 | 238550_at | 0.41 | 2.72 |
| SRRM2 | serine/arginine repetitive matrix 2 | 208610_s_at | 0.41 | 2.45 |
| C17orf42 | chromosome 17 open reading frame 42 | 219146_at | 0.41 | 1.67 |
| ADI1 | acireductone dioxygenase 1 | 217761_at | 0.41 | 3.67 |
| EBLN2 | endogenous Borna-like N element-2 | 219906_at | 0.41 | 4.93 |
| FAM13B | family with sequence similarity 13, member B | 218518_at | 0.41 | 2.06 |
| SBDS /// SBDSP1 | Shwachman-Bodian-Diamond syndrome /// Shwachman-Bodian-Diamond syndrome pseudogene 1 | 222669_s_at | 0.41 | 1.25 |
| MYO6 | myosin VI | 203215_s_at | 0.41 | 1.00 |
| HTR7P1 | 5-hydroxytryptamine (serotonin) receptor 7 pseudogene 1 | 236115_at | 0.41 | 2.45 |
| RBM4 | RNA binding motif protein 4 | 213718_at | 0.41 | 3.67 |
| TAF3 | TAF3 RNA polymerase II, TATA box binding protein (TBP)-associated factor, 140kDa | 235119_at | 0.41 | 1.44 |
| RBM41 | RNA binding motif protein 41 | 219754_at | 0.41 | 1.67 |
| CHD2 | chromodomain helicase DNA binding protein 2 | 1554014_at | 0.41 | 3.21 |
| HNRPLL | heterogeneous nuclear ribonucleoprotein L-like | 225385_s_at | 0.41 | 1.25 |
| LEPROT | leptin receptor overlapping transcript | 227095_at | 0.41 | 2.45 |
| WDR78 | WD repeat domain 78 | 1554140_at | 0.41 | 1.67 |
| LOC100505501 | hypothetical LOC100505501 | 235171_at | 0.41 | 3.21 |
| LOC100505759 | hypothetical LOC100505759 | 228971_at | 0.41 | 2.45 |
| RBM25 | RNA binding motif protein 25 | 212027_at | 0.41 | 1.44 |
| RBM25 | RNA binding motif protein 25 | 212033_at | 0.41 | 1.00 |
| TMEFF1 | transmembrane protein with EGF-like and two follistatin-like domains 1 | 205122_at | 0.41 | 0.86 |
| CUL2 | cullin 2 | 203078_at | 0.41 | 4.15 |
| MXRA5 | matrix-remodelling associated 5 | 209596_at | 0.41 | 7.84 |
| GUSBL1 | glucuronidase, beta-like 1 | 1555568_at | 0.41 | 3.21 |
| MARCH5 | membrane-associated ring finger (C3HC4) 5 | 226394_at | 0.41 | 1.25 |
| FAIM | Fas apoptotic inhibitory molecule | 220643_s_at | 0.41 | 2.45 |
| DAAM1 | dishevelled associated activator of morphogenesis 1 | 226666_at | 0.41 | 1.44 |
| AKAP12 | A kinase (PRKA) anchor protein 12 | 227530_at | 0.41 | 4.15 |
| LOC646214 | p21 protein (Cdc42/Rac)-activated kinase 2 pseudogene | 236283_x_at | 0.41 | 1.44 |
| PSPH | phosphoserine phosphatase | 205194_at | 0.41 | 3.67 |
| GOPC | Golgi-associated PDZ and coiled-coil motif containing | 236862_at | 0.41 | 3.67 |
| CSTF2T | cleavage stimulation factor, 3' pre-RNA, subunit 2, 64kDa, tau variant | 212901_s_at | 0.40 | 2.72 |
| CNTNAP3 | contactin associated protein-like 3 | 223796_at | 0.40 | 4.93 |
| ZNF468 | zinc finger protein 468 | 214751_at | 0.40 | 1.67 |
| SREK1 | splicing regulatory glutamine/lysine-rich protein 1 | 244287_at | 0.40 | 1.67 |
| HSPA4 | heat shock 70kDa protein 4 | 208814_at | 0.40 | 3.67 |
| KHDRBS1 | KH domain containing, RNA binding, signal transduction associated 1 | 201488_x_at | 0.40 | 1.00 |
| ZNF680 | zinc finger protein 680 | 229533_x_at | 0.40 | 2.06 |
| LYRM7 | Lyrm7 homolog (mouse) | 239960_x_at | 0.40 | 3.67 |
| MCL1 | Myeloid cell leukemia sequence 1 (BCL2-related) | 214056_at | 0.40 | 2.45 |
| POP1 | processing of precursor 1, ribonuclease P/MRP subunit (S. cerevisiae) | 213449_at | 0.40 | 4.15 |
| THOC2 | THO complex 2 | 222122_s_at | 0.40 | 1.25 |
| ND6 | NADH dehydrogenase, subunit 6 (complex I) | 1553575_at | 0.40 | 2.45 |
| CASP6 | caspase 6, apoptosis-related cysteine peptidase | 211464_x_at | 0.40 | 2.06 |
| TMX4 | thioredoxin-related transmembrane protein 4 | 201581_at | 0.40 | 1.25 |
| C6orf145 | Chromosome 6 open reading frame 145 | 231387_at | 0.40 | 3.21 |
| EMX2 | empty spiracles homeobox 2 | 221950_at | 0.40 | 3.67 |
| KCMF1 | potassium channel modulatory factor 1 | 242887_at | 0.40 | 1.44 |
| AXL | AXL receptor tyrosine kinase | 202686_s_at | 0.40 | 1.67 |
| ALG14 | asparagine-linked glycosylation 14 homolog (S. cerevisiae) | 1553954_at | 0.40 | 2.45 |
| SLC25A27 | solute carrier family 25, member 27 | 230624_at | 0.40 | 4.93 |
| C2orf67 | chromosome 2 open reading frame 67 | 230561_s_at | 0.40 | 3.67 |
| FNIP2 | folliculin interacting protein 2 | 225924_at | 0.40 | 5.82 |
| PTGIS | prostaglandin I2 (prostacyclin) synthase | 208131_s_at | 0.40 | 6.81 |
| CCT5 | chaperonin containing TCP1, subunit 5 (epsilon) | 229068_at | 0.40 | 3.67 |
| NCAM2 | neural cell adhesion molecule 2 | 205669_at | 0.40 | 3.67 |
| CHRDL1 | chordin-like 1 | 209763_at | 0.40 | 7.84 |
| NEK1 | NIMA (never in mitosis gene a)-related kinase 1 | 211086_x_at | 0.40 | 3.21 |
| TMEM17 | transmembrane protein 17 | 1557137_at | 0.40 | 2.45 |
| NOL7 | nucleolar protein 7, 27kDa | 213838_at | 0.40 | 2.06 |
| MPHOSPH9 | M-phase phosphoprotein 9 | 1558369_at | 0.40 | 1.67 |
| ANKRD12 | ankyrin repeat domain 12 | 216550_x_at | 0.40 | 2.45 |
| LOC148413 | hypothetical LOC148413 | 225934_at | 0.40 | 2.06 |
| VPS13C | vacuolar protein sorting 13 homolog C (S. cerevisiae) | 218396_at | 0.40 | 0.86 |
| ADK | adenosine kinase | 204120_s_at | 0.40 | 2.72 |
| ZNF765 | zinc finger protein 765 | 1558942_at | 0.40 | 1.44 |
| ANGPTL1 | angiopoietin-like 1 | 231773_at | 0.40 | 4.93 |
| NEXN | nexilin (F actin binding protein) | 1552309_a_at | 0.40 | 2.45 |
| MKLN1 | Muskelin 1, intracellular mediator containing kelch motifs | 1560145_at | 0.40 | 3.21 |
| RDH10 | retinol dehydrogenase 10 (all-trans) | 226021_at | 0.40 | 9.00 |
| RAB4A /// SPHAR | RAB4A, member RAS oncogene family /// S-phase response (cyclin related) | 206272_at | 0.40 | 1.25 |
| ZNF280B | zinc finger protein 280B | 230789_at | 0.40 | 2.72 |
| LRRC14 | leucine rich repeat containing 14 | 32062_at | 0.40 | 2.72 |
| CCDC18 | coiled-coil domain containing 18 | 236665_at | 0.40 | 4.93 |
| FAM161A | family with sequence similarity 161, member A | 1557385_at | 0.40 | 2.45 |
| C6orf62 | chromosome 6 open reading frame 62 | 208809_s_at | 0.40 | 0.63 |
| NUFIP2 | nuclear fragile X mental retardation protein interacting protein 2 | 224956_at | 0.39 | 1.67 |
| ALDH1L2 | aldehyde dehydrogenase 1 family, member L2 | 231202_at | 0.39 | 1.00 |
| DRAM1 | DNA-damage regulated autophagy modulator 1 | 241992_at | 0.39 | 1.67 |
| PGGT1B | protein geranylgeranyltransferase type I, beta subunit | 235615_at | 0.39 | 1.25 |
| EFCAB2 | EF-hand calcium binding domain 2 | 223608_at | 0.39 | 6.81 |
| ZNF711 | zinc finger protein 711 | 228988_at | 0.39 | 4.15 |
| MIDN | midnolin | 225954_s_at | 0.39 | 1.44 |
| NUCKS1 | Nuclear casein kinase and cyclin-dependent kinase substrate 1 | 222027_at | 0.39 | 2.06 |
| EEA1 | early endosome antigen 1 | 204840_s_at | 0.39 | 4.15 |
| C5orf44 | chromosome 5 open reading frame 44 | 236526_x_at | 0.39 | 3.67 |
| SLC24A1 | solute carrier family 24 (sodium/potassium/calcium exchanger), member 1 | 206081_at | 0.39 | 2.06 |
| ESF1 | ESF1, nucleolar pre-rRNA processing protein, homolog (S. cerevisiae) | 218859_s_at | 0.39 | 0.73 |
| SLC5A3 | solute carrier family 5 (sodium/myo-inositol cotransporter), member 3 | 212944_at | 0.39 | 1.67 |
| YWHAB | tyrosine 3-monooxygenase/tryptophan 5-monooxygenase activation protein, beta polypeptide | 217717_s_at | 0.39 | 1.44 |
| PXDN | peroxidasin homolog (Drosophila) | 212012_at | 0.39 | 1.25 |
| RIF1 | RAP1 interacting factor homolog (yeast) | 236620_at | 0.39 | 2.06 |
| IFT81 | intraflagellar transport 81 homolog (Chlamydomonas) | 219372_at | 0.39 | 1.25 |
| THUMPD3 | THUMP domain containing 3 | 225730_s_at | 0.39 | 0.86 |
| TCF25 | transcription factor 25 (basic helix-loop-helix) | 213311_s_at | 0.39 | 1.44 |
| PHAX | phosphorylated adaptor for RNA export | 235767_x_at | 0.39 | 0.86 |
| RNF213 | ring finger protein 213 | 241347_at | 0.39 | 3.21 |
| LOC100287616 | Hypothetical protein LOC100287616 | 232752_at | 0.39 | 2.06 |
| ATRX | alpha thalassemia/mental retardation syndrome X-linked | 208859_s_at | 0.39 | 2.06 |
| SPESP1 | sperm equatorial segment protein 1 | 229352_at | 0.39 | 3.67 |
| ANP32A | acidic (leucine-rich) nuclear phosphoprotein 32 family, member A | 201038_s_at | 0.39 | 1.44 |
| FAM172A | family with sequence similarity 172, member A | 212936_at | 0.39 | 0.86 |
| RRP15 | ribosomal RNA processing 15 homolog (S. cerevisiae) | 214764_at | 0.39 | 2.72 |
| SDAD1 | SDA1 domain containing 1 | 228408_s_at | 0.39 | 1.00 |
| GINS3 | GINS complex subunit 3 (Psf3 homolog) | 45633_at | 0.39 | 2.72 |
| SYS1 | SYS1 Golgi-localized integral membrane protein homolog (S. cerevisiae) | 238470_at | 0.39 | 2.45 |
| C3orf64 | chromosome 3 open reading frame 64 | 221935_s_at | 0.39 | 1.67 |
| CHSY3 | chondroitin sulfate synthase 3 | 242100_at | 0.39 | 4.15 |
| CPSF6 | cleavage and polyadenylation specific factor 6, 68kDa | 226934_at | 0.39 | 3.21 |
| UBE2G2 | ubiquitin-conjugating enzyme E2G 2 (UBC7 homolog, yeast) | 1557053_s_at | 0.39 | 1.25 |
| ESM1 | endothelial cell-specific molecule 1 | 208394_x_at | 0.39 | 2.45 |
| SEC14L1 | SEC14-like 1 (S. cerevisiae) | 202082_s_at | 0.39 | 1.25 |
| ERO1LB | ERO1-like beta (S. cerevisiae) | 231944_at | 0.39 | 2.72 |
| EIF3M | eukaryotic translation initiation factor 3, subunit M | 215190_at | 0.39 | 3.67 |
| WASL | Wiskott-Aldrich syndrome-like | 205809_s_at | 0.39 | 2.72 |
| CHD2 | chromodomain helicase DNA binding protein 2 | 228999_at | 0.39 | 2.45 |
| RASEF | RAS and EF-hand domain containing | 1553185_at | 0.39 | 3.67 |
| PRRG1 | proline rich Gla (G-carboxyglutamic acid) 1 | 205618_at | 0.39 | 0.86 |
| DENR | density-regulated protein | 238982_at | 0.38 | 3.21 |
| SPEN | spen homolog, transcriptional regulator (Drosophila) | 201996_s_at | 0.38 | 1.44 |
| NUP214 | nucleoporin 214kDa | 228902_at | 0.38 | 1.67 |
| FAM98B | family with sequence similarity 98, member B | 1564637_a_at | 0.38 | 1.25 |
| ACADSB | acyl-CoA dehydrogenase, short/branched chain | 205355_at | 0.38 | 2.45 |
| LOC100128822 | hypothetical LOC100128822 | 235174_s_at | 0.38 | 1.44 |
| COBLL1 | COBL-like 1 | 203642_s_at | 0.38 | 5.82 |
| PTMA | prothymosin, alpha | 211921_x_at | 0.38 | 1.00 |
| ZNF587 | zinc finger protein 587 | 1558253_x_at | 0.38 | 0.63 |
| USP25 | ubiquitin specific peptidase 25 | 223167_s_at | 0.38 | 1.67 |
| SLC35E3 | solute carrier family 35, member E3 | 218988_at | 0.38 | 1.44 |
| C10orf32 | chromosome 10 open reading frame 32 | 225334_at | 0.38 | 2.06 |
| AMIGO2 | adhesion molecule with Ig-like domain 2 | 222108_at | 0.38 | 2.06 |
| TCEB1 | transcription elongation factor B (SIII), polypeptide 1 (15kDa, elongin C) | 202823_at | 0.38 | 2.45 |
| LOC401093 | hypothetical LOC401093 | 232298_at | 0.38 | 2.72 |
| LOC145474 | hypothetical LOC145474 | 230505_at | 0.38 | 5.82 |
| ING3 | inhibitor of growth family, member 3 | 231863_at | 0.38 | 1.67 |
| COL4A3BP | collagen, type IV, alpha 3 (Goodpasture antigen) binding protein | 223465_at | 0.38 | 1.00 |
| RPS11 | Ribosomal protein S11 | 213350_at | 0.38 | 1.25 |
| LMO4 | LIM domain only 4 | 209204_at | 0.38 | 1.00 |
| SFRS18 | splicing factor, arginine/serine-rich 18 | 230375_at | 0.38 | 2.45 |
| FAM59A | family with sequence similarity 59, member A | 228115_at | 0.38 | 2.45 |
| FUS | fused in sarcoma | 200959_at | 0.38 | 3.67 |
| NHS | Nance-Horan syndrome (congenital cataracts and dental anomalies) | 242800_at | 0.38 | 2.72 |
| POLE2 | polymerase (DNA directed), epsilon 2 (p59 subunit) | 205909_at | 0.38 | 6.81 |
| DHTKD1 | dehydrogenase E1 and transketolase domain containing 1 | 209916_at | 0.38 | 2.06 |
| NUB1 | negative regulator of ubiquitin-like proteins 1 | 1569030_s_at | 0.38 | 3.21 |
| ATXN7 | ataxin 7 | 243259_at | 0.38 | 1.25 |
| B3GALT2 | UDP-Gal:betaGlcNAc beta 1,3-galactosyltransferase, polypeptide 2 | 217452_s_at | 0.38 | 3.67 |
| RBM26 | RNA binding motif protein 26 | 218422_s_at | 0.38 | 0.73 |
| HNRNPUL2 | heterogeneous nuclear ribonucleoprotein U-like 2 | 222264_at | 0.38 | 1.67 |
| UBTD2 | ubiquitin domain containing 2 | 224827_at | 0.38 | 0.73 |
| SERAC1 | serine active site containing 1 | 1569864_at | 0.38 | 2.06 |
| SORBS2 | sorbin and SH3 domain containing 2 | 1558815_at | 0.38 | 1.67 |
| CELF2 | CUGBP, Elav-like family member 2 | 202158_s_at | 0.38 | 2.45 |
| LOC100505497 | hypothetical LOC100505497 | 228204_at | 0.38 | 1.25 |
| RIOK3 | RIO kinase 3 (yeast) | 202131_s_at | 0.38 | 1.67 |
| DCAF16 | DDB1 and CUL4 associated factor 16 | 219717_at | 0.38 | 0.86 |
| USP45 | ubiquitin specific peptidase 45 | 238057_at | 0.38 | 1.00 |
| TET3 | tet oncogene family member 3 | 235542_at | 0.38 | 3.21 |
| SGOL2 | shugoshin-like 2 (S. pombe) | 235425_at | 0.38 | 2.06 |
| EIF3F | Eukaryotic translation initiation factor 3, subunit F | 226014_at | 0.38 | 2.72 |
| SEC22B | SEC22 vesicle trafficking protein homolog B (S. cerevisiae) (gene/pseudogene) | 209207_s_at | 0.37 | 1.00 |
| GLIPR1 | GLI pathogenesis-related 1 | 226136_at | 0.37 | 4.15 |
| AGBL5 | ATP/GTP binding protein-like 5 | 238889_at | 0.37 | 2.72 |
| NCRNA00182 | non-protein coding RNA 182 | 1558515_at | 0.37 | 1.25 |
| CA13 | carbonic anhydrase XIII | 231270_at | 0.37 | 3.67 |
| FMO3 | flavin containing monooxygenase 3 | 40665_at | 0.37 | 6.81 |
| GDAP1 | ganglioside-induced differentiation-associated protein 1 | 226269_at | 0.37 | 3.67 |
| TES | testis derived transcript (3 LIM domains) | 202720_at | 0.37 | 2.06 |
| LRRFIP1 | leucine rich repeat (in FLII) interacting protein 1 | 201861_s_at | 0.37 | 1.00 |
| ENAH | enabled homolog (Drosophila) | 217820_s_at | 0.37 | 0.86 |
| ATP1B1 | ATPase, Na+/K+ transporting, beta 1 polypeptide | 201243_s_at | 0.37 | 1.44 |
| SMAD9 | SMAD family member 9 | 227719_at | 0.37 | 3.67 |
| HSD17B6 | hydroxysteroid (17-beta) dehydrogenase 6 homolog (mouse) | 37512_at | 0.37 | 2.72 |
| STRAP | Serine/threonine kinase receptor associated protein | 1558002_at | 0.37 | 3.21 |
| INTS4 | integrator complex subunit 4 | 1569253_at | 0.37 | 2.72 |
| ZC3H7B | zinc finger CCCH-type containing 7B | 205877_s_at | 0.37 | 1.25 |
| SLC44A1 | solute carrier family 44, member 1 | 224595_at | 0.37 | 1.44 |
| CNTLN | centlein, centrosomal protein | 239989_at | 0.37 | 3.21 |
| SRSF3 | serine/arginine-rich splicing factor 3 | 208673_s_at | 0.37 | 2.72 |
| MSL3 | male-specific lethal 3 homolog (Drosophila) | 214009_at | 0.37 | 2.45 |
| MRAP2 | melanocortin 2 receptor accessory protein 2 | 227226_at | 0.37 | 4.93 |
| SNORA21 | small nucleolar RNA, H/ACA box 21 | 215224_at | 0.37 | 2.45 |
| NCAPG | non-SMC condensin I complex, subunit G | 218662_s_at | 0.37 | 3.67 |
| SMC3 | structural maintenance of chromosomes 3 | 209257_s_at | 0.37 | 2.72 |
| LOC100505971 | hypothetical LOC100505971 | 236704_at | 0.37 | 3.67 |
| THOC4 | THO complex 4 | 226319_s_at | 0.37 | 2.45 |
| LOC642361 | hypothetical LOC642361 | 228839_s_at | 0.37 | 1.67 |
| WSB2 | WD repeat and SOCS box-containing 2 | 213734_at | 0.37 | 0.86 |
| DNAJC7 | DnaJ (Hsp40) homolog, subfamily C, member 7 | 1556053_at | 0.37 | 3.67 |
| CDH11 | cadherin 11, type 2, OB-cadherin (osteoblast) | 236179_at | 0.37 | 2.45 |
| MIB1 | mindbomb homolog 1 (Drosophila) | 224726_at | 0.37 | 0.44 |
| HGF | hepatocyte growth factor (hepapoietin A; scatter factor) | 209960_at | 0.37 | 6.81 |
| TRAPPC10 | trafficking protein particle complex 10 | 1555446_s_at | 0.37 | 0.86 |
| APLP2 | Amyloid beta (A4) precursor-like protein 2 | 228520_s_at | 0.37 | 1.25 |
| ANKRD44 | ankyrin repeat domain 44 | 228471_at | 0.37 | 2.72 |
| PNMAL1 | PNMA-like 1 | 218824_at | 0.37 | 2.72 |
| RTN4 | reticulon 4 | 1556049_at | 0.37 | 5.82 |
| SDAD1 | SDA1 domain containing 1 | 224037_at | 0.36 | 3.21 |
| LOC389834 | ankyrin repeat domain 57 pseudogene | 226558_at | 0.36 | 2.45 |
| MPPED2 | metallophosphoesterase domain containing 2 | 205413_at | 0.36 | 6.81 |
| LIMA1 | LIM domain and actin binding 1 | 222457_s_at | 0.36 | 0.86 |
| STRN | striatin, calmodulin binding protein | 230734_x_at | 0.36 | 0.86 |
| XG | Xg blood group | 1554062_at | 0.36 | 5.82 |
| N4BP2L2 | NEDD4 binding protein 2-like 2 | 221899_at | 0.36 | 3.67 |
| SRSF1 | serine/arginine-rich splicing factor 1 | 211784_s_at | 0.36 | 1.25 |
| ZDHHC13 | zinc finger, DHHC-type containing 13 | 219296_at | 0.36 | 1.44 |
| RAB32 | RAB32, member RAS oncogene family | 228161_at | 0.36 | 1.00 |
| PCBD2 | pterin-4 alpha-carbinolamine dehydratase/dimerization cofactor of hepatocyte nuclear factor 1 alpha (TCF1) 2 | 223712_at | 0.36 | 2.45 |
| SKI | v-ski sarcoma viral oncogene homolog (avian) | 204270_at | 0.36 | 1.25 |
| ENY2 | enhancer of yellow 2 homolog (Drosophila) | 226776_at | 0.36 | 3.21 |
| TMEM19 | transmembrane protein 19 | 229126_at | 0.36 | 2.06 |
| PTMA | prothymosin, alpha | 200772_x_at | 0.36 | 0.86 |
| IER3IP1 | immediate early response 3 interacting protein 1 | 211406_at | 0.36 | 0.86 |
| ZHX1 | zinc fingers and homeoboxes 1 | 223213_s_at | 0.36 | 0.55 |
| TXN | Thioredoxin | 216609_at | 0.36 | 0.86 |
| NAP1L2 | nucleosome assembly protein 1-like 2 | 219368_at | 0.36 | 3.21 |
| CDC42 | cell division cycle 42 (GTP binding protein, 25kDa) | 208728_s_at | 0.36 | 0.33 |
| C1orf163 | chromosome 1 open reading frame 163 | 222883_at | 0.36 | 2.45 |
| FLG | filaggrin | 215704_at | 0.36 | 3.67 |
| PRPF39 | PRP39 pre-mRNA processing factor 39 homolog (S. cerevisiae) | 220553_s_at | 0.35 | 0.26 |
| LOC100506935 | hypothetical LOC100506935 | 222309_at | 0.35 | 2.45 |
| ZNF770 | zinc finger protein 770 | 220608_s_at | 0.35 | 2.45 |
| ELF1 | E74-like factor 1 (ets domain transcription factor) | 212418_at | 0.35 | 1.00 |
| JUN | jun proto-oncogene | 201465_s_at | 0.35 | 1.67 |
| NUTF2 | nuclear transport factor 2 | 202397_at | 0.35 | 0.86 |
| MEG3 | maternally expressed 3 (non-protein coding) | 235077_at | 0.35 | 1.25 |
| RAB12 | RAB12, member RAS oncogene family | 239891_x_at | 0.35 | 3.67 |
| ALDH5A1 | aldehyde dehydrogenase 5 family, member A1 | 203608_at | 0.35 | 2.06 |
| LDB3 | LIM domain binding 3 | 216887_s_at | 0.35 | 3.21 |
| ING3 | inhibitor of growth family, member 3 | 242293_at | 0.35 | 2.06 |
| ZNF704 | zinc finger protein 704 | 235079_at | 0.35 | 0.63 |
| DHFR | dihydrofolate reductase | 48808_at | 0.35 | 3.21 |
| FGD4 | FYVE, RhoGEF and PH domain containing 4 | 230559_x_at | 0.35 | 6.81 |
| PALLD | palladin, cytoskeletal associated protein | 200906_s_at | 0.35 | 1.25 |
| MCM9 | minichromosome maintenance complex component 9 | 1553759_at | 0.35 | 2.72 |
| FNIP1 /// RAPGEF6 | folliculin interacting protein 1 /// Rap guanine nucleotide exchange factor (GEF) 6 | 1555247_a_at | 0.35 | 1.25 |
| FARSB | phenylalanyl-tRNA synthetase, beta subunit | 232063_x_at | 0.35 | 3.21 |
| ZMAT3 | zinc finger, matrin-type 3 | 228315_at | 0.35 | 1.00 |
| TXNDC15 | thioredoxin domain containing 15 | 227873_at | 0.35 | 2.72 |
| MED7 | mediator complex subunit 7 | 204349_at | 0.35 | 1.44 |
| N4BP2 | NEDD4 binding protein 2 | 228242_at | 0.35 | 0.44 |
| CAV1 | caveolin 1, caveolae protein, 22kDa | 203065_s_at | 0.35 | 1.00 |
| EIF5B | eukaryotic translation initiation factor 5B | 201024_x_at | 0.35 | 0.63 |
| HNRNPL | heterogeneous nuclear ribonucleoprotein L | 35201_at | 0.35 | 0.86 |
| SOBP | sine oculis binding protein homolog (Drosophila) | 218974_at | 0.35 | 0.86 |
| SAMD9L | sterile alpha motif domain containing 9-like | 235643_at | 0.35 | 2.45 |
| CLIP4 | CAP-GLY domain containing linker protein family, member 4 | 226425_at | 0.35 | 0.73 |
| KCTD15 | potassium channel tetramerisation domain containing 15 | 222668_at | 0.35 | 1.00 |
| NCRNA00182 | non-protein coding RNA 182 | 242121_at | 0.35 | 2.72 |
| HECTD1 | HECT domain containing 1 | 241955_at | 0.35 | 0.55 |
| FRY | furry homolog (Drosophila) | 204072_s_at | 0.35 | 3.21 |
| NFIB | nuclear factor I/B | 213032_at | 0.35 | 2.06 |
| PCDH7 | protocadherin 7 | 228640_at | 0.35 | 3.67 |
| NMT2 | N-myristoyltransferase 2 | 215743_at | 0.35 | 3.67 |
| LOC283788 | FSHD region gene 1 pseudogene | 229187_at | 0.35 | 2.45 |
| FOXO3 | Forkhead box O3 | 231548_at | 0.35 | 3.21 |
| OPCML | opioid binding protein/cell adhesion molecule-like | 214111_at | 0.35 | 4.15 |
| CCNI | Cyclin I | 227299_at | 0.35 | 0.55 |
| MALAT1 | metastasis associated lung adenocarcinoma transcript 1 (non-protein coding) | 1558678_s_at | 0.34 | 2.45 |
| PRPF4B | PRP4 pre-mRNA processing factor 4 homolog B (yeast) | 202126_at | 0.34 | 1.00 |
| HAPLN1 | hyaluronan and proteoglycan link protein 1 | 230895_at | 0.34 | 6.81 |
| STRN3 | striatin, calmodulin binding protein 3 | 215505_s_at | 0.34 | 1.44 |
| CCDC88A | coiled-coil domain containing 88A | 219387_at | 0.34 | 1.25 |
| EP300 | E1A binding protein p300 | 213579_s_at | 0.34 | 0.35 |
| CDH6 | cadherin 6, type 2, K-cadherin (fetal kidney) | 214803_at | 0.34 | 2.06 |
| MYO5A | myosin VA (heavy chain 12, myoxin) | 227761_at | 0.34 | 1.67 |
| PRKAA2 | protein kinase, AMP-activated, alpha 2 catalytic subunit | 227892_at | 0.34 | 7.84 |
| NTN4 | netrin 4 | 223315_at | 0.34 | 3.67 |
| KLHL20 | kelch-like 20 (Drosophila) | 204176_at | 0.34 | 1.00 |
| DPY19L2P2 | dpy-19-like 2 pseudogene 2 (C. elegans) | 215143_at | 0.34 | 2.72 |
| CHAC2 | ChaC, cation transport regulator homolog 2 (E. coli) | 235117_at | 0.34 | 5.82 |
| ZNF573 | zinc finger protein 573 | 217627_at | 0.34 | 0.33 |
| ARPC4 | actin related protein 2/3 complex, subunit 4, 20kDa | 217818_s_at | 0.34 | 2.45 |
| VPS41 | vacuolar protein sorting 41 homolog (S. cerevisiae) | 235625_at | 0.34 | 0.63 |
| RPL14 | ribosomal protein L14 | 219138_at | 0.34 | 2.45 |
| AP1S1 | adaptor-related protein complex 1, sigma 1 subunit | 209635_at | 0.34 | 0.73 |
| RHEB | Ras homolog enriched in brain | 213409_s_at | 0.34 | 1.00 |
| CNTN3 | contactin 3 (plasmacytoma associated) | 229831_at | 0.34 | 2.72 |
| H2AFV | H2A histone family, member V | 212205_at | 0.34 | 1.44 |
| SLC7A11 | solute carrier family 7, (cationic amino acid transporter, y+ system) member 11 | 209921_at | 0.34 | 1.44 |
| PCDH19 | protocadherin 19 | 227282_at | 0.34 | 1.67 |
| B3GNT5 | UDP-GlcNAc:betaGal beta-1,3-N-acetylglucosaminyltransferase 5 | 225612_s_at | 0.34 | 2.72 |
| PCSK5 | proprotein convertase subtilisin/kexin type 5 | 213652_at | 0.34 | 1.44 |
| HELLS | helicase, lymphoid-specific | 227350_at | 0.34 | 6.81 |
| SETD5 | SET domain containing 5 | 222575_at | 0.34 | 0.86 |
| PCDHB2 | protocadherin beta 2 | 231725_at | 0.34 | 3.67 |
| MAP3K8 | Mitogen-activated protein kinase kinase kinase 8 | 235421_at | 0.34 | 2.06 |
| EML4 | echinoderm microtubule associated protein like 4 | 228674_s_at | 0.34 | 2.45 |
| BPTF | bromodomain PHD finger transcription factor | 207186_s_at | 0.33 | 0.86 |
| C3orf14 | chromosome 3 open reading frame 14 | 219288_at | 0.33 | 3.21 |
| SBNO1 | strawberry notch homolog 1 (Drosophila) | 218737_at | 0.33 | 0.86 |
| JAG1 | Jagged 1 (Alagille syndrome) | 231183_s_at | 0.33 | 2.06 |
| C10orf93 | chromosome 10 open reading frame 93 | 1555573_at | 0.33 | 2.72 |
| FAM105B | family with sequence similarity 105, member B | 240834_at | 0.33 | 2.06 |
| SSPN | sarcospan (Kras oncogene-associated gene) | 204963_at | 0.33 | 2.06 |
| CHD9 | chromodomain helicase DNA binding protein 9 | 235388_at | 0.33 | 1.25 |
| SRSF8 | serine/arginine-rich splicing factor 8 | 228760_at | 0.33 | 1.25 |
| THBS1 | thrombospondin 1 | 235086_at | 0.33 | 1.44 |
| PLAGL1 | pleiomorphic adenoma gene-like 1 | 207002_s_at | 0.33 | 0.86 |
| KCNQ5 | potassium voltage-gated channel, KQT-like subfamily, member 5 | 244623_at | 0.33 | 6.81 |
| ZNF654 | zinc finger protein 654 | 219239_s_at | 0.33 | 1.00 |
| HNMT | histamine N-methyltransferase | 228772_at | 0.33 | 2.06 |
| HCG11 | HLA complex group 11 | 1557169_x_at | 0.33 | 0.73 |
| MTMR9 | myotubularin related protein 9 | 204837_at | 0.33 | 0.23 |
| NUP50 | nucleoporin 50kDa | 218294_s_at | 0.33 | 0.86 |
| LHFP | Lipoma HMGIC fusion partner | 231411_at | 0.33 | 0.44 |
| BAX | BCL2-associated X protein | 211833_s_at | 0.33 | 0.73 |
| C6orf48 | chromosome 6 open reading frame 48 | 222968_at | 0.33 | 1.67 |
| ALS2 | amyotrophic lateral sclerosis 2 (juvenile) | 232184_at | 0.33 | 1.67 |
| ZNF326 | zinc finger protein 326 | 227680_at | 0.33 | 1.00 |
| LDLR | low density lipoprotein receptor | 202068_s_at | 0.33 | 2.72 |
| RAB27A | RAB27A, member RAS oncogene family | 222294_s_at | 0.33 | 0.19 |
| RAD50 | RAD50 homolog (S. cerevisiae) | 209349_at | 0.33 | 2.06 |
| PDZD2 | PDZ domain containing 2 | 209493_at | 0.33 | 3.67 |
| GULP1 | GULP, engulfment adaptor PTB domain containing 1 | 204237_at | 0.32 | 4.93 |
| SMARCA2 | SWI/SNF related, matrix associated, actin dependent regulator of chromatin, subfamily a, member 2 | 206543_at | 0.32 | 1.25 |
| FER | fer (fps/fes related) tyrosine kinase | 232064_at | 0.32 | 1.00 |
| ARHGAP32 | Rho GTPase activating protein 32 | 203431_s_at | 0.32 | 1.00 |
| FBXL17 | F-box and leucine-rich repeat protein 17 | 227203_at | 0.32 | 0.55 |
| DHFR | dihydrofolate reductase | 202534_x_at | 0.32 | 2.72 |
| LOC220729 /// SDHA /// SDHAP1 /// SDHAP2 | succinate dehydrogenase complex, subunit A, flavoprotein pseudogene /// succinate dehydrogenase complex, subunit A, flavoprotein (Fp) /// succinate dehydrogenase complex, subunit A, flavoprotein pseudogene 1 /// succinate dehydrogenase complex, subunit A, flavoprotein pseudogene 2 | 230077_at | 0.32 | 1.44 |
| LOC100192378 | hypothetical LOC100192378 | 1559965_at | 0.32 | 1.25 |
| CNTLN | centlein, centrosomal protein | 1559005_s_at | 0.32 | 2.45 |
| LOC647979 | hypothetical LOC647979 | 1558028_x_at | 0.32 | 0.63 |
| JUN | Jun oncogene | 213281_at | 0.32 | 1.67 |
| GOLIM4 | golgi integral membrane protein 4 | 204324_s_at | 0.32 | 1.00 |
| ARL15 | ADP-ribosylation factor-like 15 | 219842_at | 0.32 | 0.44 |
| FAM36A | family with sequence similarity 36, member A | 224820_at | 0.32 | 1.25 |
| WNK1 | WNK lysine deficient protein kinase 1 | 211993_at | 0.32 | 0.73 |
| PNN | pinin, desmosome associated protein | 1567213_at | 0.32 | 1.00 |
| JAK1 | Janus kinase 1 | 1552611_a_at | 0.32 | 0.44 |
| MYO9A | myosin IXA | 219027_s_at | 0.31 | 0.73 |
| TPM1 | Tropomyosin 1 (alpha) | 238688_at | 0.31 | 1.44 |
| CTTN | cortactin | 201059_at | 0.31 | 0.73 |
| ABCA5 | ATP-binding cassette, sub-family A (ABC1), member 5 | 213353_at | 0.31 | 1.25 |
| STK38L | serine/threonine kinase 38 like | 212572_at | 0.31 | 1.25 |
| N4BP2L2 | NEDD4 binding protein 2-like 2 | 214748_at | 0.31 | 1.67 |
| THOC4 | THO complex 4 | 226320_at | 0.31 | 1.67 |
| LOC100506076 /// LOC100506123 | hypothetical LOC100506076 /// hypothetical LOC100506123 | 221973_at | 0.31 | 2.06 |
| ARHGAP20 | Rho GTPase activating protein 20 | 228368_at | 0.31 | 1.00 |
| CHURC1 | churchill domain containing 1 | 226736_at | 0.31 | 4.93 |
| NOG | noggin | 231798_at | 0.31 | 2.72 |
| TLR4 | toll-like receptor 4 | 232068_s_at | 0.31 | 2.72 |
| LOC338620 | hypothetical protein LOC338620 | 230930_at | 0.31 | 4.15 |
| FAM115A | family with sequence similarity 115, member A | 224030_s_at | 0.31 | 1.00 |
| XPNPEP3 | X-prolyl aminopeptidase (aminopeptidase P) 3, putative | 237750_at | 0.31 | 1.44 |
| DNAJC21 | DnaJ (Hsp40) homolog, subfamily C, member 21 | 238335_at | 0.31 | 1.44 |
| NAPEPLD | N-acyl phosphatidylethanolamine phospholipase D | 238722_x_at | 0.31 | 0.55 |
| ANKRD11 | ankyrin repeat domain 11 | 231999_at | 0.31 | 0.26 |
| PKIB | protein kinase (cAMP-dependent, catalytic) inhibitor beta | 223551_at | 0.31 | 5.82 |
| LDB3 | LIM domain binding 3 | 213371_at | 0.31 | 3.21 |
| LOC100190939 | hypothetical LOC100190939 | 226407_at | 0.31 | 1.67 |
| NRP2 | Neuropilin 2 | 228699_at | 0.31 | 1.00 |
| LOC100507248 | hypothetical LOC100507248 | 235236_at | 0.31 | 4.15 |
| ANKRD11 | ankyrin repeat domain 11 | 219437_s_at | 0.31 | 0.73 |
| STK38L | serine/threonine kinase 38 like | 212565_at | 0.31 | 1.67 |
| LOC643792 | contactin associated protein-like 3 pseudogene | 244065_at | 0.31 | 3.21 |
| MTSS1L | metastasis suppressor 1-like | 1556175_at | 0.30 | 2.06 |
| MLEC | malectin | 200616_s_at | 0.30 | 0.19 |
| MAP9 | microtubule-associated protein 9 | 220145_at | 0.30 | 0.55 |
| ETV7 | ets variant 7 | 224225_s_at | 0.30 | 1.44 |
| UBXN4 | UBX domain protein 4 | 212007_at | 0.30 | 0.44 |
| B3GALT2 | UDP-Gal:betaGlcNAc beta 1,3-galactosyltransferase, polypeptide 2 | 210121_at | 0.30 | 3.21 |
| ZNF281 | zinc finger protein 281 | 218401_s_at | 0.30 | 1.25 |
| DST | dystonin | 232098_at | 0.30 | 1.67 |
| CTH | cystathionase (cystathionine gamma-lyase) | 217127_at | 0.30 | 4.93 |
| LNX1 | ligand of numb-protein X 1 | 223611_s_at | 0.30 | 1.44 |
| DENR | density-regulated protein | 234347_s_at | 0.30 | 1.25 |
| HECTD2 | HECT domain containing 2 | 227568_at | 0.30 | 0.55 |
| IGFBP7 | insulin-like growth factor binding protein 7 | 213910_at | 0.30 | 1.25 |
| PENK | proenkephalin | 213791_at | 0.30 | 6.81 |
| LOC283788 | FSHD region gene 1 pseudogene | 229007_at | 0.30 | 2.06 |
| SGK1 | serum/glucocorticoid regulated kinase 1 | 201739_at | 0.30 | 1.44 |
| ERICH1 | glutamate-rich 1 | 227016_at | 0.30 | 1.67 |
| LOC150759 | hypothetical LOC150759 | 213703_at | 0.30 | 1.44 |
| RUFY2 | RUN and FYVE domain containing 2 | 233191_at | 0.30 | 0.86 |
| ALG13 | asparagine-linked glycosylation 13 homolog (S. cerevisiae) | 222808_at | 0.30 | 2.72 |
| ITIH5 | inter-alpha (globulin) inhibitor H5 | 1553243_at | 0.30 | 2.72 |
| NUCKS1 | nuclear casein kinase and cyclin-dependent kinase substrate 1 | 222424_s_at | 0.30 | 0.63 |
| PHLDA2 | pleckstrin homology-like domain, family A, member 2 | 209803_s_at | 0.30 | 4.93 |
| GALNT12 | UDP-N-acetyl-alpha-D-galactosamine:polypeptide N-acetylgalactosaminyltransferase 12 (GalNAc-T12) | 218885_s_at | 0.30 | 7.84 |
| EGR1 | early growth response 1 | 201694_s_at | 0.29 | 2.06 |
| C18orf1 | chromosome 18 open reading frame 1 | 207996_s_at | 0.29 | 3.21 |
| FZD8 | frizzled homolog 8 (Drosophila) | 224325_at | 0.29 | 1.25 |
| SLC12A2 | solute carrier family 12 (sodium/potassium/chloride transporters), member 2 | 225835_at | 0.29 | 0.55 |
| LOC284801 | hypothetical protein LOC284801 | 225762_x_at | 0.29 | 1.67 |
| ST3GAL1 | ST3 beta-galactoside alpha-2,3-sialyltransferase 1 | 208322_s_at | 0.29 | 1.44 |
| MALAT1 | metastasis associated lung adenocarcinoma transcript 1 (non-protein coding) | 224559_at | 0.29 | 2.45 |
| RTKN2 | rhotekin 2 | 230469_at | 0.29 | 1.67 |
| SMC3 | structural maintenance of chromosomes 3 | 209258_s_at | 0.29 | 0.55 |
| FAM129A | family with sequence similarity 129, member A | 217967_s_at | 0.29 | 4.93 |
| CARD6 | caspase recruitment domain family, member 6 | 224414_s_at | 0.29 | 1.67 |
| RG9MTD2 | RNA (guanine-9-) methyltransferase domain containing 2 | 242442_x_at | 0.29 | 1.25 |
| MEF2A | myocyte enhancer factor 2A | 208328_s_at | 0.29 | 0.44 |
| CYP27C1 | cytochrome P450, family 27, subfamily C, polypeptide 1 | 1568868_at | 0.29 | 1.67 |
| NAA15 | N(alpha)-acetyltransferase 15, NatA auxiliary subunit | 222837_s_at | 0.29 | 0.73 |
| ZNF37A | zinc finger protein 37A | 228711_at | 0.29 | 0.44 |
| ZNF345 | zinc finger protein 345 | 207236_at | 0.29 | 1.44 |
| ARMC8 | armadillo repeat containing 8 | 236966_at | 0.29 | 0.86 |
| NAA40 | N(alpha)-acetyltransferase 40, NatD catalytic subunit, homolog (S. cerevisiae) | 222369_at | 0.29 | 0.26 |
| EPC1 | enhancer of polycomb homolog 1 (Drosophila) | 223875_s_at | 0.29 | 0.73 |
| ZNF827 | Zinc finger protein 827 | 243617_at | 0.28 | 1.67 |
| RBM25 | RNA binding motif protein 25 | 1557081_at | 0.28 | 0.35 |
| DCUN1D1 | DCN1, defective in cullin neddylation 1, domain containing 1 (S. cerevisiae) | 242428_at | 0.28 | 3.21 |
| BAT2L2 | HLA-B associated transcript 2-like 2 | 214052_x_at | 0.28 | 0.86 |
| HSPA1A /// HSPA1B | heat shock 70kDa protein 1A /// heat shock 70kDa protein 1B | 202581_at | 0.28 | 0.86 |
| VSIG10 | V-set and immunoglobulin domain containing 10 | 226485_at | 0.28 | 1.00 |
| ZDBF2 | zinc finger, DBF-type containing 2 | 228749_at | 0.28 | 0.73 |
| KLF13 | Kruppel-like factor 13 | 219878_s_at | 0.28 | 1.25 |
| RPL32P3 | ribosomal protein L32 pseudogene 3 | 226877_at | 0.28 | 1.44 |
| RPS27L | ribosomal protein S27-like | 222487_s_at | 0.28 | 0.23 |
| FNIP1 /// RAPGEF6 | folliculin interacting protein 1 /// Rap guanine nucleotide exchange factor (GEF) 6 | 219112_at | 0.28 | 0.35 |
| DDX52 | DEAD (Asp-Glu-Ala-Asp) box polypeptide 52 | 213637_at | 0.28 | 0.26 |
| RSPH10B2 | radial spoke head 10 homolog B2 (Chlamydomonas) | 1555272_at | 0.28 | 1.00 |
| RIT1 | Ras-like without CAAX 1 | 243463_s_at | 0.28 | 0.44 |
| NAV1 | neuron navigator 1 | 224771_at | 0.28 | 0.33 |
| ANK3 | ankyrin 3, node of Ranvier (ankyrin G) | 209442_x_at | 0.28 | 0.73 |
| COPG2 | coatomer protein complex, subunit gamma 2 | 223457_at | 0.28 | 0.55 |
| ATP6V0E1 | ATPase, H+ transporting, lysosomal 9kDa, V0 subunit e1 | 201171_at | 0.28 | 1.44 |
| TSIX | XIST antisense RNA (non-protein coding) | 231592_at | 0.28 | 3.67 |
| ID4 | Inhibitor of DNA binding 4, dominant negative helix-loop-helix protein | 226933_s_at | 0.28 | 0.86 |
| MTPN | myotrophin | 223925_s_at | 0.27 | 2.06 |
| PHF3 | PHD finger protein 3 | 217951_s_at | 0.27 | 0.33 |
| COL21A1 | collagen, type XXI, alpha 1 | 208096_s_at | 0.27 | 2.45 |
| EME1 | essential meiotic endonuclease 1 homolog 1 (S. pombe) | 234464_s_at | 0.27 | 0.33 |
| UACA | uveal autoantigen with coiled-coil domains and ankyrin repeats | 238868_at | 0.27 | 2.72 |
| TRIM4 | tripartite motif-containing 4 | 1554287_at | 0.27 | 1.25 |
| ZNF587 | zinc finger protein 587 | 1558251_a_at | 0.27 | 0.35 |
| PAIP1 | poly(A) binding protein interacting protein 1 | 208051_s_at | 0.27 | 2.06 |
| SLC39A4 /// SLC39A7 | solute carrier family 39 (zinc transporter), member 4 /// solute carrier family 39 (zinc transporter), member 7 | 202667_s_at | 0.27 | 0.35 |
| ERGIC1 | endoplasmic reticulum-golgi intermediate compartment (ERGIC) 1 | 224576_at | 0.27 | 0.09 |
| EIF5B | eukaryotic translation initiation factor 5B | 214314_s_at | 0.27 | 0.23 |
| GNAQ | guanine nucleotide binding protein (G protein), q polypeptide | 224863_at | 0.27 | 0.55 |
| IQCA1 | IQ motif containing with AAA domain 1 | 238584_at | 0.27 | 1.67 |
| EPHA5 | EPH receptor A5 | 237939_at | 0.27 | 2.06 |
| SFRP4 | secreted frizzled-related protein 4 | 204052_s_at | 0.26 | 3.67 |
| PDE10A | phosphodiesterase 10A | 205501_at | 0.26 | 1.00 |
| PAIP1 | poly(A) binding protein interacting protein 1 | 209064_x_at | 0.26 | 1.44 |
| SSFA2 | Sperm specific antigen 2 | 229744_at | 0.26 | 1.00 |
| MYH10 | myosin, heavy chain 10, non-muscle | 213067_at | 0.26 | 0.09 |
| GTF2A1L /// STON1-GTF2A1L | general transcription factor IIA, 1-like /// STON1-GTF2A1L readthrough | 220190_s_at | 0.26 | 3.21 |
| LUC7L3 | LUC7-like 3 (S. cerevisiae) | 208835_s_at | 0.26 | 0.09 |
| ZNF720 | zinc finger protein 720 | 238510_at | 0.26 | 0.09 |
| NLGN1 | neuroligin 1 | 205893_at | 0.26 | 4.15 |
| SLC38A1 | solute carrier family 38, member 1 | 218237_s_at | 0.26 | 3.21 |
| ZBED6 | zinc finger, BED-type containing 6 | 243648_at | 0.26 | 0.63 |
| SYNCRIP | synaptotagmin binding, cytoplasmic RNA interacting protein | 209025_s_at | 0.26 | 0.63 |
| FOXC1 | forkhead box C1 | 1553613_s_at | 0.26 | 0.26 |
| PXDN | peroxidasin homolog (Drosophila) | 212013_at | 0.26 | 0.23 |
| RGS4 | regulator of G-protein signaling 4 | 204337_at | 0.26 | 4.15 |
| MFAP5 | microfibrillar associated protein 5 | 213765_at | 0.25 | 2.45 |
| POGK | pogo transposable element with KRAB domain | 239392_s_at | 0.25 | 0.73 |
| PLCL1 | phospholipase C-like 1 | 205934_at | 0.25 | 5.82 |
| RBM3 | RNA binding motif (RNP1, RRM) protein 3 | 222026_at | 0.25 | 0.33 |
| RAB2A | RAB2A, member RAS oncogene family | 221960_s_at | 0.25 | 0.63 |
| CCDC58 | coiled-coil domain containing 58 | 235244_at | 0.25 | 0.86 |
| XPNPEP3 | X-prolyl aminopeptidase (aminopeptidase P) 3, putative | 227910_at | 0.25 | 0.73 |
| FNBP1L | formin binding protein 1-like | 215017_s_at | 0.25 | 0.73 |
| TUG1 | taurine upregulated 1 (non-protein coding) | 228397_at | 0.25 | 0.26 |
| RPL38 | Ribosomal protein L38 | 221943_x_at | 0.25 | 0.09 |
| SMOC1 | SPARC related modular calcium binding 1 | 222783_s_at | 0.25 | 3.21 |
| FNDC3A | fibronectin type III domain containing 3A | 241611_s_at | 0.25 | 0.63 |
| TP53I3 | tumor protein p53 inducible protein 3 | 210609_s_at | 0.25 | 1.00 |
| DCLK1 | doublecortin-like kinase 1 | 229800_at | 0.25 | 0.86 |
| PPP3CA | protein phosphatase 3, catalytic subunit, alpha isozyme | 202425_x_at | 0.24 | 0.09 |
| HS2ST1 | heparan sulfate 2-O-sulfotransferase 1 | 230465_at | 0.24 | 0.55 |
| ARGLU1 | arginine and glutamate rich 1 | 228477_at | 0.24 | 0.00 |
| RBM6 | RNA binding motif protein 6 | 1556672_a_at | 0.24 | 2.72 |
| ID4 | Inhibitor of DNA binding 4, dominant negative helix-loop-helix protein | 209292_at | 0.24 | 0.26 |
| ATP6V0E1 | ATPase, H+ transporting, lysosomal 9kDa, V0 subunit e1 | 214149_s_at | 0.24 | 0.00 |
| PPP1R3C | protein phosphatase 1, regulatory (inhibitor) subunit 3C | 240187_at | 0.24 | 2.72 |
| LMNB1 | lamin B1 | 203276_at | 0.24 | 1.00 |
| ANKRD36B | ankyrin repeat domain 36B | 220940_at | 0.24 | 0.73 |
| CEP290 | centrosomal protein 290kDa | 221683_s_at | 0.24 | 0.35 |
| MED13L | mediator complex subunit 13-like | 212207_at | 0.24 | 0.44 |
| ADAM12 | ADAM metallopeptidase domain 12 | 213790_at | 0.24 | 0.23 |
| SLC24A3 | solute carrier family 24 (sodium/potassium/calcium exchanger), member 3 | 219090_at | 0.24 | 3.21 |
| NUDCD2 | NudC domain containing 2 | 226643_s_at | 0.24 | 0.23 |
| SCUBE3 | signal peptide, CUB domain, EGF-like 3 | 228407_at | 0.24 | 1.25 |
| NDUFS1 | NADH dehydrogenase (ubiquinone) Fe-S protein 1, 75kDa (NADH-coenzyme Q reductase) | 236356_at | 0.24 | 0.15 |
| FLJ10038 | hypothetical protein FLJ10038 | 205511_at | 0.23 | 1.00 |
| CTNNB1 | catenin (cadherin-associated protein), beta 1, 88kDa | 1554411_at | 0.23 | 0.23 |
| GTPBP8 | GTP-binding protein 8 (putative) | 242685_at | 0.23 | 0.63 |
| PPP2R3C | protein phosphatase 2, regulatory subunit B'', gamma | 1569894_at | 0.23 | 0.86 |
| ROR1 | receptor tyrosine kinase-like orphan receptor 1 | 232060_at | 0.23 | 1.25 |
| ZBTB38 | zinc finger and BTB domain containing 38 | 236557_at | 0.23 | 0.44 |
| OSBPL1A | oxysterol binding protein-like 1A | 208158_s_at | 0.23 | 0.26 |
| COX5B | Cytochrome c oxidase subunit Vb | 213736_at | 0.23 | 0.55 |
| DHX29 | DEAH (Asp-Glu-Ala-His) box polypeptide 29 | 212649_at | 0.23 | 0.23 |
| ID4 | inhibitor of DNA binding 4, dominant negative helix-loop-helix protein | 209291_at | 0.23 | 0.44 |
| SMARCC1 | SWI/SNF related, matrix associated, actin dependent regulator of chromatin, subfamily c, member 1 | 201072_s_at | 0.23 | 0.55 |
| MARCKS | myristoylated alanine-rich protein kinase C substrate | 213002_at | 0.23 | 0.09 |
| LOC339929 | hypothetical LOC339929 | 1556827_at | 0.22 | 0.86 |
| HNRNPA0 | heterogeneous nuclear ribonucleoprotein A0 | 201055_s_at | 0.22 | 0.09 |
| SEL1L | Sel-1 suppressor of lin-12-like (C. elegans) | 230265_at | 0.22 | 0.19 |
| ARHGAP32 | Rho GTPase activating protein 32 | 229648_at | 0.22 | 0.35 |
| MFAP5 | microfibrillar associated protein 5 | 213764_s_at | 0.22 | 2.06 |
| FBN2 | fibrillin 2 | 203184_at | 0.22 | 0.73 |
| CELF2 | CUGBP, Elav-like family member 2 | 202156_s_at | 0.22 | 1.44 |
| PCDHB5 | protocadherin beta 5 | 223629_at | 0.22 | 1.25 |
| ASH1L | ash1 (absent, small, or homeotic)-like (Drosophila) | 222667_s_at | 0.22 | 0.00 |
| F2R | coagulation factor II (thrombin) receptor | 203989_x_at | 0.22 | 1.00 |
| BAX | BCL2-associated X protein | 208478_s_at | 0.22 | 0.19 |
| NTRK2 | neurotrophic tyrosine kinase, receptor, type 2 | 221796_at | 0.22 | 2.45 |
| THSD4 | thrombospondin, type I, domain containing 4 | 222835_at | 0.22 | 1.25 |
| SERTAD4 | SERTA domain containing 4 | 235337_at | 0.21 | 3.67 |
| TET2 | tet oncogene family member 2 | 1569385_s_at | 0.21 | 0.35 |
| TLCD2 | TLC domain containing 2 | 1557275_a_at | 0.21 | 0.35 |
| MEOX2 | mesenchyme homeobox 2 | 206201_s_at | 0.21 | 4.93 |
| TOP1 | topoisomerase (DNA) I | 208900_s_at | 0.21 | 0.23 |
| GPR155 | G protein-coupled receptor 155 | 239533_at | 0.21 | 4.15 |
| SLC24A3 | solute carrier family 24 (sodium/potassium/calcium exchanger), member 3 | 57588_at | 0.21 | 2.72 |
| NFIB | nuclear factor I/B | 213033_s_at | 0.21 | 0.63 |
| LOC100505633 | hypothetical LOC100505633 | 239370_at | 0.20 | 1.67 |
| KAL1 | Kallmann syndrome 1 sequence | 205206_at | 0.20 | 0.44 |
| METTL9 | Methyltransferase like 9 | 226220_at | 0.20 | 0.44 |
| NLGN1 | neuroligin 1 | 231361_at | 0.20 | 1.44 |
| ATF6B | activating transcription factor 6 beta | 203168_at | 0.20 | 0.19 |
| SEPT7 | Septin 7 | 1565823_at | 0.20 | 0.33 |
| FGFR2 | fibroblast growth factor receptor 2 | 208229_at | 0.20 | 0.73 |
| APP | amyloid beta (A4) precursor protein | 214953_s_at | 0.20 | 0.09 |
| AP3D1 | adaptor-related protein complex 3, delta 1 subunit | 208710_s_at | 0.20 | 0.44 |
| OSBPL1A | oxysterol binding protein-like 1A | 209485_s_at | 0.19 | 0.55 |
| MALAT1 | metastasis associated lung adenocarcinoma transcript 1 (non-protein coding) | 224568_x_at | 0.19 | 0.44 |
| HNRNPUL2 | heterogeneous nuclear ribonucleoprotein U-like 2 | 66053_at | 0.19 | 0.23 |
| SPG7 | spastic paraplegia 7 (pure and complicated autosomal recessive) | 230885_at | 0.19 | 0.33 |
| SRSF6 | serine/arginine-rich splicing factor 6 | 206108_s_at | 0.18 | 0.44 |
| LRRC17 | leucine rich repeat containing 17 | 205381_at | 0.18 | 1.67 |
| HIST1H4C | histone cluster 1, H4c | 205967_at | 0.18 | 2.45 |
| RPL38 | ribosomal protein L38 | 202028_s_at | 0.18 | 0.00 |
| LOC100506941 | hypothetical LOC100506941 | 231559_at | 0.17 | 0.09 |
| SPRY4 | sprouty homolog 4 (Drosophila) | 220983_s_at | 0.17 | 7.84 |
| CDC42BPA | CDC42 binding protein kinase alpha (DMPK-like) | 214464_at | 0.17 | 0.00 |
| PURB | purine-rich element binding protein B | 226762_at | 0.17 | 0.00 |
| EYA4 | eyes absent homolog 4 (Drosophila) | 207327_at | 0.17 | 0.55 |
| TAF15 | TAF15 RNA polymerase II, TATA box binding protein (TBP)-associated factor, 68kDa | 202840_at | 0.17 | 0.00 |
| MALAT1 | metastasis associated lung adenocarcinoma transcript 1 (non-protein coding) | 223940_x_at | 0.17 | 0.19 |
| YTHDC2 | YTH domain containing 2 | 1568680_s_at | 0.17 | 0.00 |
| MLL3 | myeloid/lymphoid or mixed-lineage leukemia 3 | 232940_s_at | 0.16 | 0.00 |
| NLGN4X | neuroligin 4, X-linked | 221933_at | 0.16 | 2.72 |
| EPHA3 | EPH receptor A3 | 206070_s_at | 0.16 | 0.23 |
| ARSB | arylsulfatase B | 232197_x_at | 0.16 | 0.19 |
| PCSK5 | proprotein convertase subtilisin/kexin type 5 | 205559_s_at | 0.16 | 0.23 |
| SUN1 | Sad1 and UNC84 domain containing 1 | 206487_at | 0.16 | 0.09 |
| POSTN | periostin, osteoblast specific factor | 214981_at | 0.16 | 0.86 |
| EBF1 | Early B-cell factor 1 | 233261_at | 0.15 | 0.44 |
| LUZP1 | leucine zipper protein 1 | 1558173_a_at | 0.15 | 0.19 |
| DMD | dystrophin | 203881_s_at | 0.15 | 1.00 |
| WASF2 | WAS protein family, member 2 | 224563_at | 0.15 | 0.23 |
| EIF5B | Eukaryotic translation initiation factor 5B | 214313_s_at | 0.15 | 0.35 |
| ALG5 | asparagine-linked glycosylation 5, dolichyl-phosphate beta-glucosyltransferase homolog (S. cerevisiae) | 222556_at | 0.14 | 0.35 |
| TMEM154 | transmembrane protein 154 | 238063_at | 0.14 | 0.19 |
| RAB12 | RAB12, member RAS oncogene family | 235059_at | 0.14 | 0.23 |
| EGR1 | early growth response 1 | 201693_s_at | 0.14 | 0.55 |
| TMEM200A | transmembrane protein 200A | 234994_at | 0.14 | 0.86 |
| TULP4 | tubby like protein 4 | 224170_s_at | 0.14 | 0.09 |
| EIF5B | eukaryotic translation initiation factor 5B | 201026_at | 0.14 | 0.00 |
| SLC38A1 | solute carrier family 38, member 1 | 224579_at | 0.14 | 0.35 |
| SERTAD4 | SERTA domain containing 4 | 229674_at | 0.14 | 0.86 |
| MALAT1 | metastasis associated lung adenocarcinoma transcript 1 (non-protein coding) | 224567_x_at | 0.13 | 0.00 |
| MALAT1 | metastasis associated lung adenocarcinoma transcript 1 (non-protein coding) | 226675_s_at | 0.13 | 0.00 |
| KRT18 | keratin 18 | 201596_x_at | 0.13 | 3.67 |
| PPP3R1 | protein phosphatase 3, regulatory subunit B, alpha | 204507_s_at | 0.12 | 0.00 |
| SLC22A3 | solute carrier family 22 (extraneuronal monoamine transporter), member 3 | 205421_at | 0.12 | 2.72 |
| SGCG | sarcoglycan, gamma (35kDa dystrophin-associated glycoprotein) | 207302_at | 0.12 | 0.55 |
| MED18 | mediator complex subunit 18 | 219730_at | 0.12 | 0.35 |
| LOC100507226 | hypothetical LOC100507226 | 243599_at | 0.12 | 0.00 |
| ITCH | itchy E3 ubiquitin protein ligase homolog (mouse) | 235057_at | 0.12 | 0.00 |
| FAM76B | family with sequence similarity 76, member B | 1553749_at | 0.11 | 0.23 |
| MYO10 | myosin X | 216222_s_at | 0.11 | 0.19 |
| MGP | matrix Gla protein | 202291_s_at | 0.11 | 3.67 |
| MAP4 | microtubule-associated protein 4 | 200835_s_at | 0.11 | 0.00 |
| PPP3R1 | protein phosphatase 3, regulatory subunit B, alpha | 204506_at | 0.09 | 0.00 |
| ANKRD1 | ankyrin repeat domain 1 (cardiac muscle) | 206029_at | 0.09 | 0.26 |
| COL14A1 | collagen, type XIV, alpha 1 | 212865_s_at | 0.09 | 0.55 |
| HSP90B1 | heat shock protein 90kDa beta (Grp94), member 1 | 216450_x_at | 0.09 | 0.00 |
| B4GALT1 | UDP-Gal:betaGlcNAc beta 1,4- galactosyltransferase, polypeptide 1 | 201883_s_at | 0.08 | 0.00 |
| PKIB | protein kinase (cAMP-dependent, catalytic) inhibitor beta | 231120_x_at | 0.08 | 0.23 |
| ANKRD11 | ankyrin repeat domain 11 | 234701_at | 0.08 | 0.00 |
| ZIC1 | Zic family member 1 (odd-paired homolog, Drosophila) | 206373_at | 0.07 | 0.00 |
| CTSZ | cathepsin Z | 210042_s_at | 0.07 | 0.00 |
| ANKRD12 | Ankyrin repeat domain 12 | 216563_at | 0.06 | 0.00 |
| THRAP3 | thyroid hormone receptor associated protein 3 | 222439_s_at | 0.04 | 0.00 |

FC = fold change (at least 2fold); FDR = false discovery rate (< 10%)
