# Supplementary material for: The Transcriptional Profile of Mesenchymal Stem Cell Populations in Primary Osteoporosis Is Distinct and Shows Overexpression of Osteogenic Inhibitors
Source: PLoS One. 2012 Sep 24;7(9):e45142. doi: 10.1371/journal.pone.0045142 (PMC3454401; doi:10.1371/journal.pone.0045142)
Supplement: Table S2 — Gene products differentially expressed in hMSC-OP, hMSC-old and hMSC-senescent when compared to hMSC-C. arrows pointing downward = significant, reduced expression in comparison to hMSC-C; arrows pointing upward = significant, enhanced expression in comparison to hMSC-C; FC = fold change (at least 2fold); FDR = false discovery rate (<10%); — = no expression in both hMSC groups compared. (DOC) [file pone.0045142.s002.doc]

**Table S2.** Gene products differentially expressed in hMSC-OP, hMSC-old and hMSC-senescent when compared to hMSC-C.

|  | | | **hMSC-OP versus hMSC-C** | | | **hMSC-old versus hMSC-C** | | | **hMSC-senescent versus hMSC-C** | | |
| --- | --- | --- | --- | --- | --- | --- | --- | --- | --- | --- | --- |
| **Symbol** | **Gene Name** | **Probeset ID** | **FC** | **FDR (%)** |  | **FC** | **FDR (%)** |  | **FC** | **FDR (%)** |  |
| MAB21L2 | mab-21-like 2 (C. elegans) | 210303_at | 14.43 | 0.00 | ↑ | — | — |  | — | — |  |
| C6orf182 | Chromosome 6 open reading frame 182 | 1560418_at | 12.85 | 0.00 | ↑ | — | — |  | — | — |  |
| SOST | sclerostin | 223869_at | 7.30 | 2.18 | ↑ | — | — |  | — | — |  |
| MAB21L2 | mab-21-like 2 (C. elegans) | 210302_s_at | 6.94 | 0.00 | ↑ | 2.71 | 9.01 | ↑ | 0.43 | 23.22 |  |
| IGF2 /// INS-IGF2 | insulin-like growth factor 2 (somatomedin A) /// INS-IGF2 readthrough transcript | 202410_x_at | 6.39 | 3.21 | ↑ | 0.49 | 17.74 |  | 0.86 | 23.22 |  |
| ZNF208 | zinc finger protein 208 | 1568646_x_at | 6.33 | 0.62 | ↑ | — | — |  | — | — |  |
| BMP8B | bone morphogenetic protein 8b | 235275_at | 6.29 | 3.54 | ↑ | 1.27 | 31.53 |  | 0.42 | 20.38 |  |
| EGR2 | early growth response 2 | 205249_at | 5.97 | 0.62 | ↑ | 7.06 | 10.02 |  | 0.67 | 29.62 |  |
| IGF2 /// INS-IGF2 | insulin-like growth factor 2 (somatomedin A) /// INS-IGF2 readthrough transcript | 210881_s_at | 5.80 | 2.70 | ↑ | 0.31 | 17.74 |  | 0.92 | 29.62 |  |
| IBSP | integrin-binding sialoprotein | 236028_at | 5.72 | 0.00 | ↑ | 1.17 | 60.11 |  | 1.21 | 58.81 |  |
| CTXN1 | cortexin 1 | 228126_x_at | 5.57 | 0.62 | ↑ | — | — |  | — | — |  |
| CD74 | CD74 molecule, major histocompatibility complex, class II invariant chain | 209619_at | 5.27 | 0.00 | ↑ | 0.53 | 15.95 |  | 0.39 | 7.00 | ↓ |
| COL10A1 | collagen, type X, alpha 1 | 205941_s_at | 5.25 | 0.00 | ↑ | 2.12 | 38.78 |  | 0.30 | 2.70 | ↓ |
| SEPT9 | septin 9 | 207425_s_at | 5.11 | 0.00 | ↑ | 2.40 | 6.80 | ↑ | 0.78 | 43.83 |  |
| CD74 | CD74 molecule, major histocompatibility complex, class II invariant chain | 1567628_at | 4.89 | 0.00 | ↑ | 0.61 | 10.02 |  | 0.81 | 32.58 |  |
| IBSP | integrin-binding sialoprotein | 207370_at | 4.82 | 0.90 | ↑ | 0.51 | 6.80 |  | 1.05 | 48.62 |  |
| CDK18 | cyclin-dependent kinase 18 | 214797_s_at | 4.80 | 1.40 | ↑ | 0.30 | 0.60 | ↓ | 0.41 | 1.99 | ↓ |
| PGF | placental growth factor | 209652_s_at | 4.71 | 0.62 | ↑ | 2.64 | 33.65 |  | 0.17 | 0.00 | ↓ |
| RNF208 | ring finger protein 208 | 221273_s_at | 4.70 | 0.00 | ↑ | — | — |  | — | — |  |
| CLEC2B | C-type lectin domain family 2, member B | 209732_at | 4.65 | 8.24 | ↑ | 2.38 | 33.65 |  | 1.27 | 35.63 |  |
| ZFP36L1 | zinc finger protein 36, C3H type-like 1 | 211965_at | 4.43 | 0.00 | ↑ | 0.42 | 9.01 | ↓ | 0.76 | 48.62 |  |
| MTSS1L | metastasis suppressor 1-like | 1555894_s_at | 4.39 | 0.00 | ↑ | 1.08 | 57.71 |  | 0.94 | 35.63 |  |
| FBXO17 /// SARS2 | F-box protein 17 /// seryl-tRNA synthetase 2, mitochondrial | 220233_at | 4.36 | 0.62 | ↑ | 3.26 | 1.74 | ↑ | — | — |  |
| AQP1 | aquaporin 1 (Colton blood group) | 209047_at | 4.34 | 2.70 | ↑ | 1.02 | 38.78 |  | 0.82 | 41.22 |  |
| TSPAN32 | tetraspanin 32 | 233467_s_at | 4.30 | 7.26 | ↑ | — | — |  | — | — |  |
| HLA-DRB1 /// HLA-DRB3 /// HLA-DRB4 | major histocompatibility complex, class II, DR beta 1 /// major histocompatibility complex, class II, DR beta 3 /// major histocompatibility complex, class II, DR beta 4 | 215193_x_at | 4.16 | 3.21 | ↑ | 1.35 | 45.34 |  | 0.87 | 20.38 |  |
| MUSK | muscle, skeletal, receptor tyrosine kinase | 207633_s_at | 4.10 | 4.38 | ↑ | — | — |  | 1.00 | 43.83 |  |
| NR1D1 /// THRA | nuclear receptor subfamily 1, group D, member 1 /// thyroid hormone receptor, alpha (erythroblastic leukemia viral (v-erb-a) oncogene homolog, avian) | 204760_s_at | 4.10 | 0.00 | ↑ | 0.91 | 38.78 |  | 1.07 | 48.62 |  |
| LOC389906 | hypothetical LOC389906 | 1569629_x_at | 4.09 | 0.62 | ↑ | 0.99 | 54.70 |  | 0.80 | 38.27 |  |
| CSF1 | colony stimulating factor 1 (macrophage) | 211839_s_at | 4.07 | 2.70 | ↑ | — | — |  | — | — |  |
| MAP2K7 | mitogen-activated protein kinase kinase 7 | 209951_s_at | 4.06 | 0.00 | ↑ | — | — |  | 2.19 | 1.61 | ↑ |
| CBX4 | chromobox homolog 4 | 206724_at | 4.04 | 0.00 | ↑ | — | — |  | 0.80 | 54.08 |  |
| COL10A1 | collagen, type X, alpha 1 | 217428_s_at | 4.00 | 0.62 | ↑ | 2.82 | 47.13 |  | 0.23 | 1.61 | ↓ |
| KHSRP | KH-type splicing regulatory protein | 212303_x_at | 3.99 | 0.00 | ↑ | 0.67 | 15.95 |  | 1.21 | 56.10 |  |
| ATXN2L | ataxin 2-like | 201806_s_at | 3.99 | 0.00 | ↑ | 0.67 | 17.74 |  | 1.30 | 35.63 |  |
| HMGA2 | high mobility group AT-hook 2 | 1558682_at | 3.94 | 1.60 | ↑ | 1.18 | 29.01 |  | — | — |  |
| HLA-DRA | major histocompatibility complex, class II, DR alpha | 208894_at | 3.93 | 2.46 | ↑ | 1.09 | 59.66 |  | 0.42 | 5.75 | ↓ |
| MINK1 | misshapen-like kinase 1 | 209241_x_at | 3.87 | 1.60 | ↑ | 0.87 | 47.13 |  | — | — |  |
| MTA1 | metastasis associated 1 | 202247_s_at | 3.87 | 0.00 | ↑ | 0.34 | 0.98 | ↓ | 0.94 | 58.81 |  |
| HSPB6 | heat shock protein, alpha-crystallin-related, B6 | 226304_at | 3.87 | 0.00 | ↑ | 0.43 | 5.22 | ↓ | 3.11 | 8.02 | ↑ |
| ZNF107 | Zinc finger protein 107 | 243312_at | 3.86 | 4.38 | ↑ | — | — |  | — | — |  |
| HLA-DRB1 /// HLA-DRB4 | major histocompatibility complex, class II, DR beta 1 /// major histocompatibility complex, class II, DR beta 4 | 204670_x_at | 3.81 | 3.54 | ↑ | 1.31 | 54.70 |  | 1.08 | 32.58 |  |
| SOX12 | SRY (sex determining region Y)-box 12 | 204432_at | 3.81 | 0.00 | ↑ | 2.11 | 3.81 | ↑ | 1.32 | 29.62 |  |
| PKD1 | polycystic kidney disease 1 (autosomal dominant) | 216949_s_at | 3.80 | 1.33 | ↑ | — | — |  | — | — |  |
| MARCH9 | membrane-associated ring finger (C3HC4) 9 | 226454_at | 3.75 | 0.62 | ↑ | 1.72 | 19.82 |  | 1.53 | 26.48 |  |
| C7orf68 | chromosome 7 open reading frame 68 | 1554452_a_at | 3.72 | 4.38 | ↑ | 2.49 | 45.34 |  | 0.39 | 4.16 | ↓ |
| HLA-DRB1 /// HLA-DRB4 /// HLA-DRB5 | major histocompatibility complex, class II, DR beta 1 /// major histocompatibility complex, class II, DR beta 4 /// major histocompatibility complex, class II, DR beta 5 | 209312_x_at | 3.72 | 5.70 | ↑ | 1.67 | 47.13 |  | 0.92 | 17.70 |  |
| APLP1 | amyloid beta (A4) precursor-like protein 1 | 209462_at | 3.72 | 2.46 | ↑ | — | — |  | — | — |  |
| PPDPF | pancreatic progenitor cell differentiation and proliferation factor homolog (zebrafish) | 227994_x_at | 3.71 | 0.00 | ↑ | 0.76 | 38.78 |  | 0.87 | 46.35 |  |
| ANGPTL4 | angiopoietin-like 4 | 223333_s_at | 3.69 | 7.26 | ↑ | 0.93 | 49.04 |  | 0.74 | 56.65 |  |
| PPDPF | pancreatic progenitor cell differentiation and proliferation factor homolog (zebrafish) | 218010_x_at | 3.68 | 0.00 | ↑ | 0.52 | 7.87 |  | 0.89 | 50.73 |  |
| PRR7 | proline rich 7 (synaptic) | 219742_at | 3.67 | 0.62 | ↑ | 1.73 | 14.26 |  | 0.43 | 4.84 | ↓ |
| NRXN2 | neurexin 2 | 209982_s_at | 3.66 | 0.00 | ↑ | 2.24 | 7.87 | ↑ | 0.69 | 17.70 |  |
| MINK1 | misshapen-like kinase 1 | 215909_x_at | 3.64 | 0.62 | ↑ | 0.70 | 29.01 |  | 1.14 | 56.65 |  |
| IGF2BP2 | insulin-like growth factor 2 mRNA binding protein 2 | 223963_s_at | 3.64 | 0.62 | ↑ | 0.81 | 45.34 |  | 1.75 | 9.54 |  |
| RHCE | Rh blood group, CcEe antigens | 216317_x_at | 3.55 | 1.33 | ↑ | 1.85 | 11.08 |  | — | — |  |
| TMEM148 | transmembrane protein 148 | 1552856_at | 3.54 | 0.62 | ↑ | — | — |  | — | — |  |
| SEZ6L2 | seizure related 6 homolog (mouse)-like 2 | 233337_s_at | 3.51 | 1.40 | ↑ | 1.02 | 59.66 |  | 0.33 | 0.72 | ↓ |
| GAS1 | growth arrest-specific 1 | 204456_s_at | 3.50 | 2.46 | ↑ | — | — |  | — | — |  |
| PTPRD | protein tyrosine phosphatase, receptor type, D | 213362_at | 3.50 | 3.90 | ↑ | 1.14 | 49.04 |  | 0.98 | 38.27 |  |
| ZFP36L2 | zinc finger protein 36, C3H type-like 2 | 201367_s_at | 3.48 | 1.90 | ↑ | 0.07 | 0.00 | ↓ | 1.29 | 54.85 |  |
| HLA-DPA1 | major histocompatibility complex, class II, DP alpha 1 | 211991_s_at | 3.47 | 2.18 | ↑ | 1.80 | 24.54 |  | 0.84 | 17.70 |  |
| GDNF | glial cell derived neurotrophic factor | 230090_at | 3.43 | 0.62 | ↑ | 0.86 | 43.50 |  | 0.95 | 58.81 |  |
| RPP25 | ribonuclease P/MRP 25kDa subunit | 219143_s_at | 3.43 | 0.90 | ↑ | — | — |  | — | — |  |
| AP2A1 | adaptor-related protein complex 2, alpha 1 subunit | 234068_s_at | 3.42 | 0.62 | ↑ | 0.15 | 0.00 | ↓ | 1.13 | 50.73 |  |
| HLA-DRA | major histocompatibility complex, class II, DR alpha | 210982_s_at | 3.42 | 2.46 | ↑ | 1.00 | 41.29 |  | — | — |  |
| SDC3 | syndecan 3 | 202898_at | 3.41 | 1.60 | ↑ | 2.72 | 5.22 | ↑ | — | — |  |
| XAB2 | XPA binding protein 2 | 218110_at | 3.41 | 0.62 | ↑ | 1.01 | 49.04 |  | — | — |  |
| TMED8 | transmembrane emp24 protein transport domain containing 8 | 232913_at | 3.40 | 2.70 | ↑ | — | — |  | 1.95 | 15.27 |  |
| FSTL3 | follistatin-like 3 (secreted glycoprotein) | 203592_s_at | 3.40 | 1.60 | ↑ | 1.46 | 22.17 |  | 1.05 | 57.14 |  |
| PLUNC | palate, lung and nasal epithelium associated | 220542_s_at | 3.36 | 3.21 | ↑ | — | — |  | — | — |  |
| CYFIP2 | cytoplasmic FMR1 interacting protein 2 | 215785_s_at | 3.36 | 1.60 | ↑ | 1.48 | 31.53 |  | 0.26 | 0.00 | ↓ |
| SLC35F5 | solute carrier family 35, member F5 | 240335_at | 3.35 | 1.40 | ↑ | 1.04 | 49.04 |  | 1.47 | 23.22 |  |
| C4orf47 | chromosome 4 open reading frame 47 | 236915_at | 3.35 | 9.04 | ↑ | 4.64 | 15.95 |  | 0.28 | 9.54 | ↓ |
| ANGPTL4 | angiopoietin-like 4 | 221009_s_at | 3.35 | 8.24 | ↑ | 1.56 | 50.64 |  | 0.64 | 48.62 |  |
| NBL1 | neuroblastoma, suppression of tumorigenicity 1 | 201621_at | 3.34 | 0.62 | ↑ | 0.68 | 26.77 |  | 1.04 | 54.08 |  |
| CMTM4 | CKLF-like MARVEL transmembrane domain containing 4 | 1554677_s_at | 3.33 | 2.70 | ↑ | — | — |  | 1.83 | 17.70 |  |
| LOC100128988 | hypothetical LOC100128988 | 232849_at | 3.27 | 3.54 | ↑ | 1.12 | 60.11 |  | 1.33 | 57.14 |  |
| DAB2IP | DAB2 interacting protein | 228942_s_at | 3.27 | 1.40 | ↑ | — | — |  | — | — |  |
| TRIB1 | tribbles homolog 1 (Drosophila) | 239818_x_at | 3.26 | 3.90 | ↑ | — | — |  | — | — |  |
| BCL3 | B-cell CLL/lymphoma 3 | 204907_s_at | 3.25 | 1.33 | ↑ | — | — |  | — | — |  |
| LSP1 | lymphocyte-specific protein 1 | 203523_at | 3.22 | 1.40 | ↑ | 0.65 | 29.01 |  | 0.09 | 0.00 | ↓ |
| ATN1 | atrophin 1 | 40489_at | 3.20 | 0.62 | ↑ | — | — |  | — | — |  |
| NHLH2 | nescient helix loop helix 2 | 215228_at | 3.20 | 8.24 | ↑ | 2.05 | 15.95 |  | 1.63 | 41.22 |  |
| LOC100508121 | meteorin-like | 232269_x_at | 3.19 | 0.00 | ↑ | 1.73 | 6.11 |  | 0.80 | 26.48 |  |
| EHD2 | EH-domain containing 2 | 205341_at | 3.18 | 1.40 | ↑ | 0.57 | 19.82 |  | 0.83 | 50.73 |  |
| BTG1 | B-cell translocation gene 1, anti-proliferative | 200921_s_at | 3.17 | 0.00 | ↑ | 2.39 | 6.11 | ↑ | 0.76 | 23.22 |  |
| RRAGD | Ras-related GTP binding D | 221523_s_at | 3.16 | 2.70 | ↑ | 1.76 | 36.20 |  | 1.82 | 20.38 |  |
| C7orf68 | chromosome 7 open reading frame 68 | 218507_at | 3.14 | 7.26 | ↑ | 2.23 | 47.13 |  | 0.43 | 3.36 | ↓ |
| C1QTNF1 | C1q and tumor necrosis factor related protein 1 | 224197_s_at | 3.13 | 0.62 | ↑ | 0.25 | 0.00 | ↓ | 1.28 | 50.73 |  |
| SLC6A8 | solute carrier family 6 (neurotransmitter transporter, creatine), member 8 | 213843_x_at | 3.13 | 0.90 | ↑ | 0.64 | 22.17 |  | 0.68 | 32.58 |  |
| KREMEN1 | kringle containing transmembrane protein 1 | 224534_at | 3.12 | 1.20 | ↑ | 0.68 | 14.26 |  | 1.09 | 58.81 |  |
| DDIT4 | DNA-damage-inducible transcript 4 | 202887_s_at | 3.11 | 2.46 | ↑ | 2.36 | 11.08 |  | 0.24 | 2.70 | ↓ |
| ARHGDIA | Rho GDP dissociation inhibitor (GDI) alpha | 201167_x_at | 3.11 | 1.40 | ↑ | 0.57 | 36.20 |  | 1.14 | 41.22 |  |
| CHST1 | carbohydrate (keratan sulfate Gal-6) sulfotransferase 1 | 205567_at | 3.10 | 7.26 | ↑ | 0.80 | 58.42 |  | 0.18 | 1.99 | ↓ |
| SLC6A10P /// SLC6A8 | solute carrier family 6 (neurotransmitter transporter, creatine), member 10 (pseudogene) /// solute carrier family 6 (neurotransmitter transporter, creatine), member 8 | 215812_s_at | 3.08 | 1.20 | ↑ | 1.29 | 33.65 |  | 0.90 | 57.64 |  |
| FRAS1 | Fraser syndrome 1 | 226145_s_at | 3.07 | 1.40 | ↑ | 2.68 | 2.80 | ↑ | — | — |  |
| HMGA1 | high mobility group AT-hook 1 | 210457_x_at | 3.07 | 2.18 | ↑ | 0.46 | 15.95 |  | 0.64 | 38.27 |  |
| DLGAP4 | discs, large (Drosophila) homolog-associated protein 4 | 202572_s_at | 3.06 | 0.90 | ↑ | 0.68 | 41.29 |  | 1.17 | 48.62 |  |
| PPDPF | pancreatic progenitor cell differentiation and proliferation factor homolog (zebrafish) | 233571_x_at | 3.06 | 0.62 | ↑ | 0.88 | 58.42 |  | 0.84 | 41.22 |  |
| DIRAS1 | DIRAS family, GTP-binding RAS-like 1 | 226573_at | 3.06 | 0.00 | ↑ | 0.42 | 1.45 | ↓ | 0.76 | 23.22 |  |
| C12orf69 | chromosome 12 open reading frame 69 | 237484_at | 3.06 | 3.21 | ↑ | 1.07 | 49.04 |  | 2.18 | 4.84 | ↑ |
| METRN | meteorin, glial cell differentiation regulator | 219051_x_at | 3.02 | 0.62 | ↑ | 1.22 | 36.20 |  | 0.80 | 29.62 |  |
| SF3A2 | splicing factor 3a, subunit 2, 66kDa | 37462_i_at | 3.00 | 0.62 | ↑ | 1.56 | 14.26 |  | 0.62 | 15.27 |  |
| PTH1R | parathyroid hormone 1 receptor | 205911_at | 3.00 | 2.70 | ↑ | — | — |  | 1.33 | 23.22 |  |
| C10orf10 | chromosome 10 open reading frame 10 | 209182_s_at | 3.00 | 2.46 | ↑ | 0.79 | 31.53 |  | 1.17 | 46.35 |  |
| FBXO16 /// ZNF395 | F-box protein 16 /// zinc finger protein 395 | 232693_s_at | 2.99 | 3.21 | ↑ | 1.56 | 36.20 |  | 0.40 | 15.27 |  |
| INF2 | inverted formin, FH2 and WH2 domain containing | 222534_s_at | 2.99 | 0.00 | ↑ | 1.04 | 58.42 |  | 0.80 | 26.48 |  |
| AQP1 | aquaporin 1 (Colton blood group) | 207542_s_at | 2.99 | 1.60 | ↑ | — | — |  | — | — |  |
| PTPN23 | protein tyrosine phosphatase, non-receptor type 23 | 223149_s_at | 2.98 | 0.00 | ↑ | — | — |  | — | — |  |
| OTUD5 | OTU domain containing 5 | 1555426_a_at | 2.98 | 0.62 | ↑ | 0.85 | 55.85 |  | 1.43 | 23.22 |  |
| HLA-DPA1 | major histocompatibility complex, class II, DP alpha 1 | 211990_at | 2.98 | 2.70 | ↑ | 2.40 | 11.08 |  | 0.67 | 11.20 |  |
| --- | --- | 223930_at | 2.98 | 2.18 | ↑ | 1.54 | 17.74 |  | 1.74 | 15.27 |  |
| C10orf58 | chromosome 10 open reading frame 58 | 224435_at | 2.97 | 5.70 | ↑ | 1.15 | 33.65 |  | — | — |  |
| LAPTM5 | lysosomal protein transmembrane 5 | 201720_s_at | 2.97 | 0.62 | ↑ | — | — |  | — | — |  |
| PHF6 | PHD finger protein 6 | 224442_at | 2.96 | 4.93 | ↑ | — | — |  | — | — |  |
| TMEM201 | transmembrane protein 201 | 228671_at | 2.96 | 0.90 | ↑ | — | — |  | — | — |  |
| P2RY4 | pyrimidinergic receptor P2Y, G-protein coupled, 4 | 221466_at | 2.95 | 8.24 | ↑ | 0.85 | 33.65 |  | 1.36 | 15.27 |  |
| ITGB2 | integrin, beta 2 (complement component 3 receptor 3 and 4 subunit) | 202803_s_at | 2.94 | 0.62 | ↑ | 0.91 | 55.85 |  | 1.31 | 55.52 |  |
| MEF2D | myocyte enhancer factor 2D | 203003_at | 2.93 | 2.46 | ↑ | — | — |  | — | — |  |
| DKFZp686O1327 | Hypothetical gene supported by BC043549; BX648102 | 216874_at | 2.92 | 6.30 | ↑ | 1.71 | 19.82 |  | 1.09 | 52.56 |  |
| PTGDS | prostaglandin D2 synthase 21kDa (brain) | 211663_x_at | 2.91 | 2.70 | ↑ | 0.99 | 59.12 |  | 0.53 | 9.54 |  |
| MPP2 | membrane protein, palmitoylated 2 (MAGUK p55 subfamily member 2) | 213270_at | 2.91 | 1.33 | ↑ | 1.15 | 38.78 |  | 0.96 | 58.81 |  |
| ARHGDIA | Rho GDP dissociation inhibitor (GDI) alpha | 213606_s_at | 2.91 | 2.18 | ↑ | 0.50 | 33.65 |  | 1.10 | 41.22 |  |
| NOTCH3 | notch 3 | 203238_s_at | 2.90 | 0.90 | ↑ | 0.71 | 24.54 |  | 0.40 | 2.70 | ↓ |
| EPN1 | epsin 1 | 226667_x_at | 2.89 | 0.62 | ↑ | 0.34 | 0.98 | ↓ | 1.04 | 58.81 |  |
| FOXC2 | forkhead box C2 (MFH-1, mesenchyme forkhead 1) | 214520_at | 2.89 | 0.62 | ↑ | 0.46 | 3.35 | ↓ | 0.70 | 20.38 |  |
| SNCAIP | synuclein, alpha interacting protein | 237833_s_at | 2.88 | 0.90 | ↑ | — | — |  | — | — |  |
| SLC16A6 | solute carrier family 16, member 6 (monocarboxylic acid transporter 7) | 207038_at | 2.88 | 7.26 | ↑ | 0.99 | 49.04 |  | 0.64 | 32.58 |  |
| ADAMTS7 | ADAM metallopeptidase with thrombospondin type 1 motif, 7 | 220705_s_at | 2.88 | 0.62 | ↑ | — | — |  | — | — |  |
| RIN3 | Ras and Rab interactor 3 | 219456_s_at | 2.88 | 0.62 | ↑ | 0.27 | 0.36 | ↓ | 1.29 | 55.52 |  |
| STAT4 | signal transducer and activator of transcription 4 | 206118_at | 2.87 | 0.00 | ↑ | 1.10 | 45.34 |  | 0.80 | 26.48 |  |
| EPAS1 | endothelial PAS domain protein 1 | 200879_s_at | 2.87 | 2.70 | ↑ | 0.37 | 0.76 | ↓ | 1.15 | 57.64 |  |
| ADAMTS2 | ADAM metallopeptidase with thrombospondin type 1 motif, 2 | 214535_s_at | 2.87 | 0.00 | ↑ | 0.58 | 3.35 |  | 0.93 | 43.83 |  |
| NFIX | nuclear factor I/X (CCAAT-binding transcription factor) | 227400_at | 2.86 | 1.33 | ↑ | 2.27 | 3.35 | ↑ | 1.31 | 54.85 |  |
| PTPRD | protein tyrosine phosphatase, receptor type, D | 205712_at | 2.85 | 4.38 | ↑ | — | — |  | 1.49 | 38.27 |  |
| ENO2 | enolase 2 (gamma, neuronal) | 201313_at | 2.83 | 4.38 | ↑ | 1.08 | 55.85 |  | 0.21 | 1.02 | ↓ |
| MYO15A | myosin XVA | 220288_at | 2.83 | 2.70 | ↑ | 0.97 | 57.71 |  | 1.06 | 55.52 |  |
| HES1 | hairy and enhancer of split 1, (Drosophila) | 203394_s_at | 2.83 | 2.18 | ↑ | 2.16 | 12.66 |  | 0.23 | 0.72 | ↓ |
| PACS1 | phosphofurin acidic cluster sorting protein 1 | 220557_s_at | 2.82 | 0.62 | ↑ | — | — |  | — | — |  |
| SORBS3 | sorbin and SH3 domain containing 3 | 207788_s_at | 2.81 | 1.33 | ↑ | 0.25 | 0.98 | ↓ | 0.95 | 56.65 |  |
| ZDHHC8 | zinc finger, DHHC-type containing 8 | 225744_at | 2.81 | 0.00 | ↑ | 0.64 | 9.01 |  | 0.75 | 17.70 |  |
| BGN | biglycan | 201262_s_at | 2.80 | 1.60 | ↑ | 0.23 | 2.30 | ↓ | 0.61 | 41.22 |  |
| PHLDB3 | pleckstrin homology-like domain, family B, member 3 | 1557948_at | 2.80 | 1.33 | ↑ | 0.87 | 45.34 |  | 0.84 | 38.27 |  |
| PRR14 | proline rich 14 | 1559397_s_at | 2.80 | 1.40 | ↑ | 0.15 | 0.25 | ↓ | 0.80 | 54.08 |  |
| FLJ22536 | hypothetical locus LOC401237 | 229280_s_at | 2.79 | 1.60 | ↑ | 2.25 | 14.26 |  | — | — |  |
| LMCD1 | LIM and cysteine-rich domains 1 | 242767_at | 2.78 | 3.90 | ↑ | 2.10 | 26.77 |  | 4.74 | 1.25 | ↑ |
| FCHSD1 | FCH and double SH3 domains 1 | 226699_at | 2.77 | 2.70 | ↑ | — | — |  | — | — |  |
| LAPTM5 | lysosomal protein transmembrane 5 | 201721_s_at | 2.77 | 0.62 | ↑ | 1.02 | 58.42 |  | 0.40 | 1.99 | ↓ |
| TRPM2 | transient receptor potential cation channel, subfamily M, member 2 | 205708_s_at | 2.77 | 4.93 | ↑ | 1.13 | 41.29 |  | 0.93 | 58.81 |  |
| CR1 | complement component (3b/4b) receptor 1 (Knops blood group) | 217484_at | 2.77 | 1.60 | ↑ | — | — |  | — | — |  |
| ZYX | zyxin | 200808_s_at | 2.76 | 0.90 | ↑ | 0.34 | 0.84 | ↓ | 1.11 | 50.73 |  |
| SPRED2 | sprouty-related, EVH1 domain containing 2 | 212466_at | 2.76 | 3.54 | ↑ | 0.29 | 9.01 | ↓ | 1.40 | 32.58 |  |
| ITGB5 | Integrin, beta 5 | 214021_x_at | 2.76 | 0.00 | ↑ | 3.26 | 0.21 | ↑ | 1.34 | 15.27 |  |
| SEMA4C | sema domain, immunoglobulin domain (Ig), transmembrane domain (TM) and short cytoplasmic domain, (semaphorin) 4C | 219039_at | 2.75 | 0.90 | ↑ | 1.91 | 10.02 |  | 0.70 | 11.20 |  |
| SLC27A1 | solute carrier family 27 (fatty acid transporter), member 1 | 226728_at | 2.75 | 0.90 | ↑ | 1.24 | 49.04 |  | 1.43 | 32.58 |  |
| FKBP8 | FK506 binding protein 8, 38kDa | 40850_at | 2.74 | 1.60 | ↑ | 0.13 | 0.36 | ↓ | 0.87 | 58.81 |  |
| MAZ | MYC-associated zinc finger protein (purine-binding transcription factor) | 207824_s_at | 2.74 | 0.90 | ↑ | 0.20 | 0.29 | ↓ | 0.77 | 43.83 |  |
| PPP1R9B | protein phosphatase 1, regulatory (inhibitor) subunit 9B | 225124_at | 2.73 | 0.00 | ↑ | 0.37 | 0.60 | ↓ | 0.70 | 17.70 |  |
| SCARF2 | scavenger receptor class F, member 2 | 227557_at | 2.72 | 3.21 | ↑ | 0.63 | 12.66 |  | 1.19 | 41.22 |  |
| ZNF579 | zinc finger protein 579 | 229140_at | 2.72 | 0.62 | ↑ | 1.31 | 54.70 |  | 0.99 | 50.73 |  |
| SLC5A5 | solute carrier family 5 (sodium iodide symporter), member 5 | 211123_at | 2.72 | 1.60 | ↑ | 1.82 | 19.82 |  | 2.33 | 2.70 | ↑ |
| PHF1 | PHD finger protein 1 | 202928_s_at | 2.72 | 0.90 | ↑ | 1.42 | 22.17 |  | — | — |  |
| BCL2 | B-cell CLL/lymphoma 2 | 203685_at | 2.71 | 8.24 | ↑ | 1.35 | 29.01 |  | 0.96 | 32.58 |  |
| CRYZL1 | crystallin, zeta (quinone reductase)-like 1 | 1560609_at | 2.71 | 7.26 | ↑ | 1.52 | 26.77 |  | 0.98 | 58.77 |  |
| COL6A1 | collagen, type VI, alpha 1 | 212937_s_at | 2.70 | 2.70 | ↑ | 0.14 | 0.60 | ↓ | 0.45 | 23.22 |  |
| LOC652346 /// PML | probable transcription factor PML-like /// promyelocytic leukemia | 211588_s_at | 2.70 | 1.40 | ↑ | 0.15 | 0.25 | ↓ | 1.73 | 13.22 |  |
| CABIN1 | calcineurin binding protein 1 | 202624_s_at | 2.69 | 1.33 | ↑ | 0.94 | 54.70 |  | 0.94 | 46.35 |  |
| RARA | retinoic acid receptor, alpha | 203749_s_at | 2.69 | 0.62 | ↑ | 1.61 | 9.01 |  | 0.79 | 17.70 |  |
| SIK1 | salt-inducible kinase 1 | 208078_s_at | 2.69 | 3.21 | ↑ | 2.03 | 10.02 |  | 1.28 | 32.58 |  |
| BET1L | blocked early in transport 1 homolog (S. cerevisiae)-like | 220470_at | 2.69 | 0.62 | ↑ | 1.20 | 33.65 |  | — | — |  |
| ARHGEF19 | Rho guanine nucleotide exchange factor (GEF) 19 | 226857_at | 2.68 | 0.90 | ↑ | — | — |  | — | — |  |
| BTG1 | B-cell translocation gene 1, anti-proliferative | 200920_s_at | 2.68 | 1.90 | ↑ | 1.23 | 53.46 |  | 0.61 | 11.20 |  |
| C9orf16 | chromosome 9 open reading frame 16 | 204480_s_at | 2.67 | 0.00 | ↑ | 0.57 | 0.98 |  | 0.93 | 41.22 |  |
| CRISPLD2 | cysteine-rich secretory protein LCCL domain containing 2 | 221541_at | 2.66 | 0.62 | ↑ | 2.23 | 36.20 |  | 0.20 | 0.00 | ↓ |
| ITGB2 | integrin, beta 2 (complement component 3 receptor 3 and 4 subunit) | 1555349_a_at | 2.66 | 1.33 | ↑ | 0.38 | 1.98 | ↓ | 1.34 | 54.08 |  |
| YWHAE | tyrosine 3-monooxygenase/tryptophan 5-monooxygenase activation protein, epsilon polypeptide | 210317_s_at | 2.66 | 2.18 | ↑ | 0.17 | 0.25 | ↓ | 1.09 | 48.62 |  |
| EPB41L1 | erythrocyte membrane protein band 4.1-like 1 | 212336_at | 2.66 | 1.40 | ↑ | 0.69 | 31.53 |  | 1.07 | 54.85 |  |
| SMYD3 | SET and MYND domain containing 3 | 218788_s_at | 2.65 | 1.20 | ↑ | 1.02 | 55.85 |  | 2.43 | 3.36 | ↑ |
| GADD45B | growth arrest and DNA-damage-inducible, beta | 209304_x_at | 2.65 | 0.62 | ↑ | 2.61 | 9.01 | ↑ | 1.39 | 20.38 |  |
| AP2A1 | adaptor-related protein complex 2, alpha 1 subunit | 223237_x_at | 2.65 | 2.18 | ↑ | 0.21 | 0.84 | ↓ | 0.80 | 54.08 |  |
| CHCHD10 | coiled-coil-helix-coiled-coil-helix domain containing 10 | 224932_at | 2.65 | 1.20 | ↑ | 0.78 | 33.65 |  | 0.84 | 41.22 |  |
| C9orf95 | chromosome 9 open reading frame 95 | 1562761_at | 2.65 | 3.90 | ↑ | 0.45 | 0.40 | ↓ | 0.41 | 4.84 | ↓ |
| --- | --- | 234114_at | 2.65 | 8.24 | ↑ | — | — |  | 1.38 | 29.62 |  |
| MASP2 | mannan-binding lectin serine peptidase 2 | 207041_at | 2.64 | 5.70 | ↑ | 0.86 | 41.29 |  | 1.45 | 17.70 |  |
| GRINA | glutamate receptor, ionotropic, N-methyl D-aspartate-associated protein 1 (glutamate binding) | 212090_at | 2.64 | 1.33 | ↑ | 0.43 | 3.81 | ↓ | 0.82 | 46.35 |  |
| GPM6B | glycoprotein M6B | 209168_at | 2.64 | 3.21 | ↑ | — | — |  | — | — |  |
| PRR24 | proline rich 24 | 227325_at | 2.63 | 1.40 | ↑ | 1.37 | 22.17 |  | 0.60 | 11.20 |  |
| BACH2 | BTB and CNC homology 1, basic leucine zipper transcription factor 2 | 221234_s_at | 2.63 | 4.93 | ↑ | 1.77 | 31.53 |  | 0.55 | 4.84 |  |
| CYGB | cytoglobin | 1553572_a_at | 2.63 | 4.93 | ↑ | 0.40 | 19.82 |  | 0.57 | 26.48 |  |
| HOXA11 | homeobox A11 | 208493_at | 2.62 | 2.46 | ↑ | 1.03 | 55.85 |  | — | — |  |
| CALR | calreticulin | 214315_x_at | 2.62 | 1.60 | ↑ | 1.83 | 10.02 |  | 0.83 | 58.81 |  |
| PNPLA2 | patatin-like phospholipase domain containing 2 | 212705_x_at | 2.62 | 2.18 | ↑ | 0.39 | 0.45 | ↓ | 1.35 | 20.38 |  |
| GJD3 | gap junction protein, delta 3, 31.9kDa | 1553511_at | 2.62 | 8.24 | ↑ | 0.57 | 9.01 |  | 1.13 | 57.64 |  |
| GRAMD1A | GRAM domain containing 1A | 224807_at | 2.62 | 0.00 | ↑ | 1.21 | 45.34 |  | 0.88 | 32.58 |  |
| BCL7A | B-cell CLL/lymphoma 7A | 203795_s_at | 2.62 | 3.21 | ↑ | 1.01 | 54.70 |  | — | — |  |
| GIMAP8 | GTPase, IMAP family member 8 | 235306_at | 2.61 | 3.21 | ↑ | — | — |  | 2.19 | 15.27 |  |
| USP53 | ubiquitin specific peptidase 53 | 216775_at | 2.61 | 8.24 | ↑ | 1.41 | 36.20 |  | 3.88 | 4.16 | ↑ |
| SCARB1 | scavenger receptor class B, member 1 | 201819_at | 2.61 | 2.70 | ↑ | 0.97 | 57.71 |  | 0.68 | 15.27 |  |
| RNF19B | ring finger protein 19B | 36564_at | 2.60 | 1.40 | ↑ | 1.09 | 38.78 |  | 0.79 | 48.62 |  |
| CIC | capicua homolog (Drosophila) | 212784_at | 2.60 | 3.54 | ↑ | 1.29 | 29.01 |  | 0.86 | 58.81 |  |
| MID1IP1 | MID1 interacting protein 1 (gastrulation specific G12 homolog (zebrafish)) | 218251_at | 2.59 | 1.40 | ↑ | 1.29 | 26.77 |  | 1.01 | 55.52 |  |
| ATN1 | atrophin 1 | 208871_at | 2.59 | 1.60 | ↑ | — | — |  | — | — |  |
| PMEPA1 | prostate transmembrane protein, androgen induced 1 | 217875_s_at | 2.59 | 0.00 | ↑ | 3.49 | 1.26 | ↑ | 0.42 | 0.43 | ↓ |
| HSF1 | heat shock transcription factor 1 | 213756_s_at | 2.59 | 1.60 | ↑ | 0.24 | 0.70 | ↓ | 1.37 | 23.22 |  |
| PRDX2 | peroxiredoxin 2 | 201006_at | 2.59 | 9.04 | ↑ | 1.45 | 31.53 |  | 1.77 | 11.20 |  |
| CYGB | cytoglobin | 226632_at | 2.59 | 2.46 | ↑ | 0.94 | 55.85 |  | 0.50 | 9.54 | ↓ |
| ABTB1 | ankyrin repeat and BTB (POZ) domain containing 1 | 226442_at | 2.59 | 4.38 | ↑ | 0.07 | 0.00 | ↓ | 1.22 | 58.81 |  |
| FOXS1 | forkhead box S1 | 229731_at | 2.59 | 1.90 | ↑ | — | — |  | — | — |  |
| SLC1A5 | solute carrier family 1 (neutral amino acid transporter), member 5 | 208916_at | 2.59 | 1.40 | ↑ | 0.63 | 24.54 |  | 0.84 | 46.35 |  |
| PITPNC1 | phosphatidylinositol transfer protein, cytoplasmic 1 | 1568949_at | 2.58 | 0.00 | ↑ | — | — |  | — | — |  |
| VEGFB | vascular endothelial growth factor B | 203683_s_at | 2.58 | 1.20 | ↑ | 0.37 | 1.74 | ↓ | 0.74 | 29.62 |  |
| COL6A1 | collagen, type VI, alpha 1 | 212940_at | 2.58 | 2.70 | ↑ | 1.91 | 14.26 |  | 0.54 | 38.27 |  |
| SAMD4B | Sterile alpha motif domain containing 4B | 227511_at | 2.58 | 0.62 | ↑ | 0.31 | 0.18 | ↓ | 1.22 | 35.63 |  |
| HNRNPA3 /// HNRNPA3P1 | heterogeneous nuclear ribonucleoprotein A3 /// heterogeneous nuclear ribonucleoprotein A3 pseudogene 1 | 1555653_at | 2.58 | 1.40 | ↑ | 0.95 | 52.07 |  | 1.51 | 26.48 |  |
| TRIM8 | tripartite motif-containing 8 | 223131_s_at | 2.58 | 1.60 | ↑ | 0.33 | 0.84 | ↓ | 1.15 | 48.62 |  |
| IGF2BP2 | insulin-like growth factor 2 mRNA binding protein 2 | 218847_at | 2.57 | 0.90 | ↑ | 3.08 | 0.70 | ↑ | 0.87 | 41.22 |  |
| SEZ6L2 | seizure related 6 homolog (mouse)-like 2 | 218720_x_at | 2.57 | 2.70 | ↑ | 1.01 | 38.78 |  | 0.19 | 0.43 | ↓ |
| ATOH8 | Atonal homolog 8 (Drosophila) | 1558705_at | 2.57 | 6.30 | ↑ | — | — |  | 1.72 | 9.54 |  |
| CACNA1D | calcium channel, voltage-dependent, L type, alpha 1D subunit | 1555993_at | 2.57 | 9.04 | ↑ | 0.79 | 31.53 |  | 1.43 | 54.08 |  |
| CALR | calreticulin | 200935_at | 2.56 | 2.70 | ↑ | 0.36 | 2.80 | ↓ | 1.28 | 32.58 |  |
| H1FX | H1 histone family, member X | 204805_s_at | 2.56 | 0.62 | ↑ | 0.97 | 54.70 |  | 0.72 | 15.27 |  |
| RAB11B | RAB11B, member RAS oncogene family | 217793_at | 2.56 | 3.54 | ↑ | 0.47 | 1.45 | ↓ | 0.70 | 17.70 |  |
| LRRC15 | leucine rich repeat containing 15 | 1552960_at | 2.56 | 7.26 | ↑ | 0.37 | 41.29 |  | 0.32 | 20.38 |  |
| ZNF358 | zinc finger protein 358 | 226260_x_at | 2.56 | 1.40 | ↑ | 0.35 | 1.07 | ↓ | 0.95 | 52.56 |  |
| LAMB2P1 | laminin, beta 2 pseudogene 1 | 230616_at | 2.55 | 6.30 | ↑ | 0.95 | 53.46 |  | 1.12 | 43.83 |  |
| ADSSL1 | adenylosuccinate synthase like 1 | 226325_at | 2.55 | 3.54 | ↑ | 0.99 | 47.13 |  | 0.82 | 58.81 |  |
| LYNX1 | Ly6/neurotoxin 1 | 226305_at | 2.54 | 1.33 | ↑ | 1.08 | 50.64 |  | 0.81 | 41.22 |  |
| SF3A1 | splicing factor 3a, subunit 1, 120kDa | 201357_s_at | 2.54 | 0.62 | ↑ | 0.81 | 38.78 |  | 0.94 | 52.56 |  |
| EPOR | erythropoietin receptor | 209962_at | 2.54 | 2.18 | ↑ | 0.30 | 0.98 | ↓ | 0.34 | 4.16 | ↓ |
| GHDC | GH3 domain containing | 227159_at | 2.54 | 0.90 | ↑ | 0.50 | 1.74 |  | 0.99 | 58.81 |  |
| ABHD14B | abhydrolase domain containing 14B | 224821_at | 2.54 | 0.90 | ↑ | 0.64 | 17.74 |  | 0.70 | 20.38 |  |
| BCL6 | B-cell CLL/lymphoma 6 | 215990_s_at | 2.54 | 0.62 | ↑ | 0.84 | 38.78 |  | 1.06 | 58.51 |  |
| PCGF2 | polycomb group ring finger 2 | 203792_x_at | 2.54 | 0.00 | ↑ | 0.50 | 2.30 |  | 0.65 | 7.00 |  |
| HSF1 | heat shock transcription factor 1 | 202344_at | 2.54 | 1.60 | ↑ | 1.31 | 26.77 |  | 0.97 | 58.81 |  |
| NPAS2 | neuronal PAS domain protein 2 | 205459_s_at | 2.54 | 1.33 | ↑ | — | — |  | 1.43 | 32.58 |  |
| ZFYVE28 | zinc finger, FYVE domain containing 28 | 232408_at | 2.54 | 2.70 | ↑ | 1.10 | 59.66 |  | 0.78 | 23.22 |  |
| KRTAP8-1 | keratin associated protein 8-1 | 1564974_at | 2.53 | 4.38 | ↑ | 0.44 | 0.84 | ↓ | 1.65 | 5.75 |  |
| IGSF8 | immunoglobulin superfamily, member 8 | 225025_at | 2.53 | 1.20 | ↑ | 0.61 | 11.08 |  | 0.67 | 15.27 |  |
| HBEGF | heparin-binding EGF-like growth factor | 38037_at | 2.53 | 4.93 | ↑ | 1.40 | 24.54 |  | 0.78 | 35.63 |  |
| KDM6B | lysine (K)-specific demethylase 6B | 213146_at | 2.53 | 3.90 | ↑ | 0.43 | 6.11 | ↓ | 0.82 | 50.73 |  |
| CALB2 | calbindin 2 | 205428_s_at | 2.53 | 5.70 | ↑ | 0.71 | 38.78 |  | 1.92 | 13.22 |  |
| PARVB | parvin, beta | 204629_at | 2.53 | 1.40 | ↑ | 0.51 | 15.95 |  | 0.51 | 13.22 |  |
| ERF | Ets2 repressor factor | 203643_at | 2.52 | 1.33 | ↑ | 0.72 | 29.01 |  | 0.77 | 32.58 |  |
| SEPT1 | septin 1 | 227552_at | 2.52 | 3.54 | ↑ | — | — |  | — | — |  |
| SLC2A3 | solute carrier family 2 (facilitated glucose transporter), member 3 | 202499_s_at | 2.52 | 3.21 | ↑ | 1.82 | 24.54 |  | 0.67 | 15.27 |  |
| SLC25A29 | solute carrier family 25, member 29 | 225305_at | 2.51 | 5.70 | ↑ | 1.35 | 26.77 |  | 1.43 | 29.62 |  |
| GDF15 | growth differentiation factor 15 | 221577_x_at | 2.51 | 1.60 | ↑ | 1.83 | 22.17 |  | 0.59 | 13.22 |  |
| JUND | jun D proto-oncogene | 203751_x_at | 2.51 | 3.90 | ↑ | — | — |  | — | — |  |
| RCC1 | regulator of chromosome condensation 1 | 206499_s_at | 2.50 | 1.40 | ↑ | 1.71 | 9.01 |  | 1.08 | 54.85 |  |
| MYO9B | myosin IXB | 208452_x_at | 2.50 | 1.33 | ↑ | — | — |  | — | — |  |
| MMP11 | matrix metallopeptidase 11 (stromelysin 3) | 203878_s_at | 2.50 | 5.70 | ↑ | 1.97 | 6.11 |  | — | — |  |
| TTTY5 | testis-specific transcript, Y-linked 5 (non-protein coding) | 224040_at | 2.50 | 3.54 | ↑ | — | — |  | — | — |  |
| SCARF2 | scavenger receptor class F, member 2 | 239454_at | 2.50 | 4.38 | ↑ | — | — |  | — | — |  |
| SCARB1 | scavenger receptor class B, member 1 | 1552256_a_at | 2.49 | 1.20 | ↑ | 0.77 | 31.53 |  | 0.79 | 26.48 |  |
| ARL4C | ADP-ribosylation factor-like 4C | 202208_s_at | 2.49 | 0.62 | ↑ | 1.55 | 22.17 |  | 1.20 | 56.65 |  |
| SIN3B | SIN3 homolog B, transcription regulator (yeast) | 242129_at | 2.49 | 4.93 | ↑ | 1.37 | 29.01 |  | 1.26 | 58.81 |  |
| WDTC1 | WD and tetratricopeptide repeats 1 | 216036_x_at | 2.49 | 2.18 | ↑ | 1.04 | 54.70 |  | — | — |  |
| SSBP3 | single stranded DNA binding protein 3 | 223635_s_at | 2.49 | 1.60 | ↑ | 0.46 | 10.02 |  | 0.86 | 54.08 |  |
| MMP14 | matrix metallopeptidase 14 (membrane-inserted) | 217279_x_at | 2.49 | 1.90 | ↑ | 0.75 | 53.46 |  | 2.08 | 11.20 |  |
| TPM4 | tropomyosin 4 | 209344_at | 2.49 | 1.60 | ↑ | 0.39 | 2.80 | ↓ | 0.96 | 58.81 |  |
| SYNRG | synergin, gamma | 1553165_at | 2.48 | 5.70 | ↑ | 0.81 | 45.34 |  | — | — |  |
| COL13A1 | collagen, type XIII, alpha 1 | 211809_x_at | 2.48 | 1.60 | ↑ | 0.86 | 49.04 |  | 1.52 | 17.70 |  |
| HEYL | hairy/enhancer-of-split related with YRPW motif-like | 226828_s_at | 2.48 | 6.30 | ↑ | 1.01 | 60.11 |  | 0.85 | 32.58 |  |
| SMTN | smoothelin | 209427_at | 2.48 | 0.90 | ↑ | 0.42 | 1.45 | ↓ | 0.77 | 29.62 |  |
| PNPLA3 | patatin-like phospholipase domain containing 3 | 220675_s_at | 2.48 | 4.38 | ↑ | 0.91 | 38.78 |  | 0.80 | 50.73 |  |
| HBEGF | heparin-binding EGF-like growth factor | 203821_at | 2.48 | 4.38 | ↑ | 1.59 | 15.95 |  | 0.56 | 8.02 |  |
| NXPH3 | neurexophilin 3 | 228210_at | 2.48 | 1.90 | ↑ | — | — |  | 1.60 | 13.22 |  |
| SYDE1 | synapse defective 1, Rho GTPase, homolog 1 (C. elegans) | 212962_at | 2.48 | 1.60 | ↑ | 0.29 | 0.98 | ↓ | 1.60 | 15.27 |  |
| PPP1R15A | protein phosphatase 1, regulatory (inhibitor) subunit 15A | 202014_at | 2.47 | 1.60 | ↑ | 0.42 | 3.81 | ↓ | 1.26 | 38.27 |  |
| VEGFA | vascular endothelial growth factor A | 211527_x_at | 2.47 | 2.46 | ↑ | 0.32 | 1.98 | ↓ | 0.35 | 2.43 | ↓ |
| PRR12 | proline rich 12 | 226716_at | 2.47 | 1.33 | ↑ | 1.23 | 33.65 |  | 1.05 | 58.81 |  |
| HOXC8 | homeobox C8 | 221350_at | 2.47 | 3.54 | ↑ | 1.31 | 22.17 |  | 1.53 | 35.63 |  |
| UAP1L1 | UDP-N-acteylglucosamine pyrophosphorylase 1-like 1 | 214755_at | 2.47 | 0.62 | ↑ | 0.96 | 59.12 |  | 0.71 | 13.22 |  |
| DOT1L | DOT1-like, histone H3 methyltransferase (S. cerevisiae) | 226201_at | 2.47 | 4.38 | ↑ | 1.10 | 43.50 |  | 1.61 | 9.54 |  |
| LSM14B | LSM14B, SCD6 homolog B (S. cerevisiae) | 219653_at | 2.47 | 1.20 | ↑ | 3.00 | 0.29 | ↑ | 1.21 | 43.83 |  |
| MYO9B | myosin IXB | 217297_s_at | 2.47 | 1.60 | ↑ | 0.84 | 53.46 |  | 0.74 | 38.27 |  |
| SLC6A8 | solute carrier family 6 (neurotransmitter transporter, creatine), member 8 | 210854_x_at | 2.46 | 1.60 | ↑ | 0.49 | 6.80 | ↓ | 0.85 | 48.62 |  |
| PIM3 | pim-3 oncogene | 224739_at | 2.46 | 0.62 | ↑ | 1.58 | 17.74 |  | 0.93 | 52.56 |  |
| PRSS22 | protease, serine, 22 | 205847_at | 2.46 | 2.70 | ↑ | — | — |  | 1.57 | 15.27 |  |
| ZYX | zyxin | 215706_x_at | 2.46 | 1.90 | ↑ | 0.24 | 0.70 | ↓ | 1.02 | 56.65 |  |
| ADAMTSL4 | ADAMTS-like 4 | 226071_at | 2.45 | 9.04 | ↑ | 0.15 | 0.18 | ↓ | 2.60 | 23.22 |  |
| PVR | poliovirus receptor | 216283_s_at | 2.45 | 1.40 | ↑ | — | — |  | — | — |  |
| CTSD | cathepsin D | 200766_at | 2.45 | 0.90 | ↑ | 0.82 | 29.01 |  | 0.95 | 46.35 |  |
| TRBC1 | T cell receptor beta constant 1 | 213193_x_at | 2.45 | 3.54 | ↑ | — | — |  | — | — |  |
| REPIN1 | replication initiator 1 | 222501_s_at | 2.45 | 7.26 | ↑ | 0.03 | 0.00 | ↓ | 1.02 | 50.73 |  |
| ZBTB10 | Zinc finger and BTB domain containing 10 | 235491_at | 2.45 | 2.70 | ↑ | 0.69 | 17.74 |  | 1.09 | 58.81 |  |
| C5orf46 | chromosome 5 open reading frame 46 | 1554195_a_at | 2.44 | 5.70 | ↑ | — | — |  | — | — |  |
| VASN | vasorin | 225867_at | 2.44 | 0.90 | ↑ | 0.97 | 54.70 |  | 0.75 | 11.20 |  |
| IGF2 /// INS-IGF2 | insulin-like growth factor 2 (somatomedin A) /// INS-IGF2 readthrough transcript | 202409_at | 2.44 | 4.93 | ↑ | 0.78 | 38.78 |  | 0.47 | 9.54 | ↓ |
| MIB2 | mindbomb homolog 2 (Drosophila) | 228261_at | 2.43 | 1.60 | ↑ | 0.78 | 31.53 |  | 0.66 | 11.20 |  |
| CLIP3 | CAP-GLY domain containing linker protein 3 | 235243_at | 2.43 | 8.24 | ↑ | 0.52 | 7.87 |  | 0.98 | 58.28 |  |
| INHA | inhibin, alpha | 210141_s_at | 2.43 | 3.90 | ↑ | — | — |  | — | — |  |
| DTX3 | deltex homolog 3 (Drosophila) | 49051_g_at | 2.43 | 4.38 | ↑ | — | — |  | — | — |  |
| HSPB6 | heat shock protein, alpha-crystallin-related, B6 | 214767_s_at | 2.43 | 1.60 | ↑ | 0.24 | 0.40 | ↓ | 3.08 | 5.75 | ↑ |
| PDE3B | phosphodiesterase 3B, cGMP-inhibited | 208591_s_at | 2.42 | 1.60 | ↑ | 1.29 | 26.77 |  | 1.76 | 20.38 |  |
| ZNF667 | zinc finger protein 667 | 236635_at | 2.42 | 4.38 | ↑ | 1.92 | 17.74 |  | 0.35 | 0.00 | ↓ |
| SAMD1 | sterile alpha motif domain containing 1 | 225650_at | 2.42 | 2.46 | ↑ | 0.80 | 56.87 |  | 0.81 | 50.73 |  |
| PRKACA | protein kinase, cAMP-dependent, catalytic, alpha | 202801_at | 2.41 | 0.62 | ↑ | 0.91 | 50.64 |  | 0.86 | 32.58 |  |
| MAP2K3 | mitogen-activated protein kinase kinase 3 | 207667_s_at | 2.41 | 0.90 | ↑ | 1.06 | 50.64 |  | 1.71 | 9.54 |  |
| PLXNB3 | plexin B3 | 205957_at | 2.41 | 4.38 | ↑ | 0.72 | 36.20 |  | 0.81 | 46.35 |  |
| IGFBP5 | insulin-like growth factor binding protein 5 | 203426_s_at | 2.40 | 2.70 | ↑ | 0.93 | 55.85 |  | 2.94 | 4.84 | ↑ |
| JOSD2 | Josephin domain containing 2 | 227096_at | 2.40 | 0.90 | ↑ | 0.37 | 0.84 | ↓ | 0.79 | 32.58 |  |
| DUSP6 | dual specificity phosphatase 6 | 208893_s_at | 2.40 | 3.90 | ↑ | 1.28 | 31.53 |  | 0.90 | 32.58 |  |
| TP53I11 | tumor protein p53 inducible protein 11 | 214667_s_at | 2.40 | 1.33 | ↑ | 0.40 | 1.07 | ↓ | 1.01 | 58.81 |  |
| PTPRD | protein tyrosine phosphatase, receptor type, D | 214043_at | 2.40 | 4.93 | ↑ | 1.76 | 17.74 |  | 0.95 | 58.81 |  |
| CD24 | CD24 molecule | 208650_s_at | 2.39 | 4.93 | ↑ | 0.97 | 43.50 |  | 0.15 | 1.43 | ↓ |
| PTGDS | prostaglandin D2 synthase 21kDa (brain) | 211748_x_at | 2.39 | 4.93 | ↑ | 1.79 | 14.26 |  | 0.39 | 5.75 | ↓ |
| PLTP | phospholipid transfer protein | 202075_s_at | 2.39 | 1.90 | ↑ | 0.33 | 0.25 | ↓ | 0.62 | 9.54 |  |
| PREX1 | phosphatidylinositol-3,4,5-trisphosphate-dependent Rac exchange factor 1 | 224909_s_at | 2.39 | 2.46 | ↑ | 0.29 | 0.98 | ↓ | 0.25 | 1.25 | ↓ |
| ANKRD5 | ankyrin repeat domain 5 | 220144_s_at | 2.39 | 4.38 | ↑ | 0.85 | 26.77 |  | 0.60 | 11.20 |  |
| CALHM1 | calcium homeostasis modulator 1 | 1554367_at | 2.39 | 1.90 | ↑ | 1.14 | 47.13 |  | 1.12 | 56.10 |  |
| SYT5 | synaptotagmin V | 206161_s_at | 2.39 | 3.54 | ↑ | 1.04 | 54.70 |  | 0.90 | 46.35 |  |
| ARHGEF1 | Rho guanine nucleotide exchange factor (GEF) 1 | 203055_s_at | 2.39 | 0.90 | ↑ | 0.54 | 3.81 |  | 1.15 | 46.35 |  |
| CNOT3 | CCR4-NOT transcription complex, subunit 3 | 203239_s_at | 2.38 | 1.40 | ↑ | 0.37 | 0.98 | ↓ | 0.79 | 35.63 |  |
| VAMP2 | vesicle-associated membrane protein 2 (synaptobrevin 2) | 201557_at | 2.38 | 0.62 | ↑ | 0.29 | 0.00 | ↓ | 1.24 | 32.58 |  |
| PTPRU | protein tyrosine phosphatase, receptor type, U | 211320_s_at | 2.38 | 0.62 | ↑ | — | — |  | — | — |  |
| MNT | MAX binding protein | 236749_at | 2.38 | 3.90 | ↑ | — | — |  | — | — |  |
| EYA2 | eyes absent homolog 2 (Drosophila) | 209692_at | 2.37 | 3.54 | ↑ | 2.10 | 10.02 |  | 0.75 | 32.58 |  |
| GALNTL4 | UDP-N-acetyl-alpha-D-galactosamine:polypeptide N-acetylgalactosaminyltransferase-like 4 | 1554079_at | 2.37 | 1.90 | ↑ | 1.39 | 47.13 |  | 0.74 | 26.48 |  |
| UBE2O | ubiquitin-conjugating enzyme E2O | 218141_at | 2.37 | 1.40 | ↑ | 1.93 | 5.22 |  | 0.85 | 32.58 |  |
| LOC221442 | adenylate cyclase 10 pseudogene | 236832_at | 2.37 | 2.46 | ↑ | 1.52 | 15.95 |  | — | — |  |
| CAPS | calcyphosine | 226424_at | 2.37 | 4.93 | ↑ | 1.87 | 5.22 |  | 0.83 | 29.62 |  |
| C11orf72 | chromosome 11 open reading frame 72 | 1553438_at | 2.36 | 8.24 | ↑ | — | — |  | 1.33 | 26.48 |  |
| IGFBP5 | insulin-like growth factor binding protein 5 | 203425_s_at | 2.36 | 3.90 | ↑ | 0.87 | 47.13 |  | 2.12 | 15.27 |  |
| EBF4 | early B-cell factor 4 | 233850_s_at | 2.36 | 3.90 | ↑ | 1.03 | 55.85 |  | 1.25 | 32.58 |  |
| SOX4 | SRY (sex determining region Y)-box 4 | 201418_s_at | 2.36 | 3.54 | ↑ | 1.06 | 55.85 |  | 0.32 | 0.00 | ↓ |
| LRRC68 | leucine rich repeat containing 68 | 213751_at | 2.36 | 2.70 | ↑ | — | — |  | — | — |  |
| SNTB1 | syntrophin, beta 1 (dystrophin-associated protein A1, 59kDa, basic component 1) | 208608_s_at | 2.36 | 3.21 | ↑ | 0.39 | 2.80 | ↓ | 0.48 | 7.00 | ↓ |
| ARID1A | AT rich interactive domain 1A (SWI-like) | 210649_s_at | 2.36 | 1.90 | ↑ | 0.15 | 0.00 | ↓ | 0.88 | 58.81 |  |
| PCDH7 | protocadherin 7 | 210273_at | 2.36 | 9.04 | ↑ | 0.45 | 1.07 | ↓ | 1.97 | 9.54 |  |
| BCAP29 | B-cell receptor-associated protein 29 | 217657_at | 2.36 | 4.38 | ↑ | 0.99 | 60.31 |  | 1.01 | 46.35 |  |
| PLK1S1 | polo-like kinase 1 substrate 1 | 233241_at | 2.35 | 7.26 | ↑ | 2.65 | 10.02 |  | 0.58 | 35.63 |  |
| UNC5B | unc-5 homolog B (C. elegans) | 213100_at | 2.34 | 2.18 | ↑ | 1.55 | 19.82 |  | 0.29 | 1.99 | ↓ |
| AHDC1 | AT hook, DNA binding motif, containing 1 | 205002_at | 2.34 | 2.18 | ↑ | 1.26 | 29.01 |  | 0.98 | 58.81 |  |
| ABCF2 | ATP-binding cassette, sub-family F (GCN20), member 2 | 207623_at | 2.34 | 3.54 | ↑ | 0.97 | 57.71 |  | — | — |  |
| MAPK8IP3 | mitogen-activated protein kinase 8 interacting protein 3 | 213178_s_at | 2.34 | 1.60 | ↑ | 1.53 | 24.54 |  | 0.76 | 17.70 |  |
| SOCS3 | suppressor of cytokine signaling 3 | 206359_at | 2.34 | 3.90 | ↑ | 0.80 | 43.50 |  | 0.45 | 5.75 | ↓ |
| PTGDS | prostaglandin D2 synthase 21kDa (brain) | 212187_x_at | 2.34 | 7.26 | ↑ | 1.10 | 49.04 |  | 0.32 | 4.84 | ↓ |
| PCDHGA3 | protocadherin gamma subfamily A, 3 | 216352_x_at | 2.33 | 2.70 | ↑ | 0.20 | 0.84 | ↓ | 1.44 | 23.22 |  |
| LOC100289058 | Hypothetical protein LOC100289058 | 1563077_at | 2.33 | 3.54 | ↑ | 1.01 | 54.70 |  | 1.71 | 5.75 |  |
| ZNF581 | zinc finger protein 581 | 223389_s_at | 2.32 | 1.40 | ↑ | 1.09 | 57.71 |  | 0.72 | 11.20 |  |
| NBL1 | neuroblastoma, suppression of tumorigenicity 1 | 37005_at | 2.32 | 1.90 | ↑ | 0.93 | 60.62 |  | 0.91 | 43.83 |  |
| RGL3 | ral guanine nucleotide dissociation stimulator-like 3 | 228877_at | 2.32 | 7.26 | ↑ | — | — |  | — | — |  |
| ZNF395 | zinc finger protein 395 | 223216_x_at | 2.32 | 3.90 | ↑ | 1.41 | 36.20 |  | 0.43 | 15.27 |  |
| NAB2 | NGFI-A binding protein 2 (EGR1 binding protein 2) | 212803_at | 2.32 | 2.46 | ↑ | 0.83 | 41.29 |  | 1.27 | 46.35 |  |
| CD97 | CD97 molecule | 202910_s_at | 2.31 | 2.70 | ↑ | 1.75 | 15.95 |  | 0.56 | 11.20 |  |
| SMARCD1 | SWI/SNF related, matrix associated, actin dependent regulator of chromatin, subfamily d, member 1 | 203183_s_at | 2.31 | 1.40 | ↑ | 0.46 | 1.74 | ↓ | 1.00 | 58.81 |  |
| OVOL2 | ovo-like 2 (Drosophila) | 211778_s_at | 2.31 | 8.24 | ↑ | 1.24 | 29.01 |  | 1.39 | 35.63 |  |
| RCE1 | RCE1 homolog, prenyl protein peptidase (S. cerevisiae) | 205333_s_at | 2.31 | 1.60 | ↑ | — | — |  | — | — |  |
| DPP7 | dipeptidyl-peptidase 7 | 224814_at | 2.31 | 1.40 | ↑ | 1.19 | 43.50 |  | 1.15 | 41.22 |  |
| SRGAP1 | SLIT-ROBO Rho GTPase activating protein 1 | 1555875_at | 2.31 | 1.60 | ↑ | 0.56 | 11.08 |  | 1.99 | 4.16 |  |
| E2F5 | E2F transcription factor 5, p130-binding | 221586_s_at | 2.31 | 3.21 | ↑ | 1.93 | 6.80 |  | 0.29 | 1.61 | ↓ |
| EPB41 | erythrocyte membrane protein band 4.1 (elliptocytosis 1, RH-linked) | 207793_s_at | 2.30 | 1.60 | ↑ | — | — |  | 1.39 | 15.27 |  |
| FOXO3 /// FOXO3B | forkhead box O3 /// forkhead box O3B pseudogene | 204132_s_at | 2.30 | 0.00 | ↑ | 0.94 | 49.04 |  | 0.99 | 58.81 |  |
| MALAT1 | metastasis associated lung adenocarcinoma transcript 1 (non-protein coding) | 227510_x_at | 2.30 | 7.26 | ↑ | 0.74 | 14.26 |  | 4.59 | 0.00 | ↑ |
| RXRA | retinoid X receptor, alpha | 202426_s_at | 2.29 | 1.60 | ↑ | 0.99 | 59.66 |  | 0.68 | 9.54 |  |
| HOXA11 | homeobox A11 | 213823_at | 2.29 | 2.70 | ↑ | 1.61 | 14.26 |  | — | — |  |
| ZNF395 | zinc finger protein 395 | 221123_x_at | 2.29 | 4.38 | ↑ | 1.22 | 45.34 |  | 0.41 | 11.20 |  |
| ARHGDIA | Rho GDP dissociation inhibitor (GDI) alpha | 201168_x_at | 2.29 | 1.90 | ↑ | 0.44 | 2.80 | ↓ | 0.95 | 58.81 |  |
| PLA2G16 | phospholipase A2, group XVI | 235110_at | 2.29 | 6.30 | ↑ | 1.55 | 15.95 |  | 1.59 | 17.70 |  |
| PHKA2 | phosphorylase kinase, alpha 2 (liver) | 209438_at | 2.29 | 4.38 | ↑ | 1.43 | 12.66 |  | 0.90 | 41.22 |  |
| CDC42EP1 | CDC42 effector protein (Rho GTPase binding) 1 | 204693_at | 2.29 | 2.18 | ↑ | 0.30 | 1.98 | ↓ | 0.87 | 58.81 |  |
| PRCD | progressive rod-cone degeneration | 230015_at | 2.28 | 6.30 | ↑ | — | — |  | — | — |  |
| KCTD13 | potassium channel tetramerisation domain containing 13 | 45653_at | 2.28 | 0.90 | ↑ | 0.79 | 33.65 |  | 0.79 | 29.62 |  |
| PTPRO | protein tyrosine phosphatase, receptor type, O | 211600_at | 2.28 | 3.21 | ↑ | 0.47 | 6.11 | ↓ | 1.58 | 23.22 |  |
| PTGS2 | prostaglandin-endoperoxide synthase 2 (prostaglandin G/H synthase and cyclooxygenase) | 204748_at | 2.28 | 7.26 | ↑ | 1.67 | 31.53 |  | 0.45 | 7.00 | ↓ |
| MEF2D | myocyte enhancer factor 2D | 203004_s_at | 2.28 | 8.24 | ↑ | 1.17 | 33.65 |  | — | — |  |
| SLC9A1 | solute carrier family 9 (sodium/hydrogen exchanger), member 1 | 209453_at | 2.28 | 3.21 | ↑ | 1.24 | 31.53 |  | 1.10 | 50.73 |  |
| GPR85 | G protein-coupled receptor 85 | 219898_at | 2.28 | 3.90 | ↑ | 2.18 | 6.11 | ↑ | 0.87 | 58.81 |  |
| PARVB | parvin, beta | 37965_at | 2.27 | 2.18 | ↑ | 0.35 | 2.30 | ↓ | 0.54 | 15.27 |  |
| SIX5 | SIX homeobox 5 | 229009_at | 2.27 | 0.90 | ↑ | 0.85 | 43.50 |  | 0.79 | 29.62 |  |
| PARVB | parvin, beta | 216253_s_at | 2.27 | 2.18 | ↑ | 0.24 | 0.60 | ↓ | 0.42 | 5.75 | ↓ |
| SNAP25 | Synaptosomal-associated protein, 25kDa | 1556629_a_at | 2.27 | 2.70 | ↑ | 0.84 | 58.42 |  | 0.74 | 52.56 |  |
| APBB1IP | amyloid beta (A4) precursor protein-binding, family B, member 1 interacting protein | 219994_at | 2.27 | 1.90 | ↑ | 0.29 | 0.98 | ↓ | 2.62 | 1.43 | ↑ |
| SLC9A5 | solute carrier family 9 (sodium/hydrogen exchanger), member 5 | 220650_s_at | 2.27 | 1.40 | ↑ | 1.48 | 22.17 |  | 2.12 | 3.36 | ↑ |
| PIR | pirin (iron-binding nuclear protein) | 207469_s_at | 2.27 | 3.21 | ↑ | 1.86 | 10.02 |  | 0.52 | 1.25 |  |
| DNAJC1 | DnaJ (Hsp40) homolog, subfamily C, member 1 | 218409_s_at | 2.26 | 1.40 | ↑ | 1.20 | 31.53 |  | 1.11 | 46.35 |  |
| ZNF740 | zinc finger protein 740 | 1558277_at | 2.26 | 6.30 | ↑ | 1.33 | 49.04 |  | 1.14 | 58.81 |  |
| FGFR1 | fibroblast growth factor receptor 1 | 207822_at | 2.26 | 3.90 | ↑ | 1.60 | 26.77 |  | 0.94 | 41.22 |  |
| FAM85A | family with sequence similarity 85, member A | 238716_at | 2.25 | 3.21 | ↑ | 1.55 | 31.53 |  | 0.51 | 8.02 |  |
| VAMP2 | vesicle-associated membrane protein 2 (synaptobrevin 2) | 201556_s_at | 2.25 | 1.40 | ↑ | 0.20 | 0.00 | ↓ | 0.99 | 54.08 |  |
| PER1 | period homolog 1 (Drosophila) | 202861_at | 2.25 | 2.46 | ↑ | 1.56 | 29.01 |  | — | — |  |
| RNF145 | ring finger protein 145 | 238949_at | 2.25 | 1.60 | ↑ | 2.78 | 0.53 | ↑ | 0.95 | 52.56 |  |
| PTOV1 | prostate tumor overexpressed 1 | 212032_s_at | 2.25 | 0.62 | ↑ | 0.88 | 41.29 |  | 0.59 | 2.70 |  |
| SLC17A7 | solute carrier family 17 (sodium-dependent inorganic phosphate cotransporter), member 7 | 204230_s_at | 2.25 | 3.21 | ↑ | — | — |  | — | — |  |
| MYL4 | myosin, light chain 4, alkali; atrial, embryonic | 216054_x_at | 2.25 | 2.70 | ↑ | 1.23 | 31.53 |  | 1.60 | 15.27 |  |
| SHROOM2 | shroom family member 2 | 204967_at | 2.25 | 3.21 | ↑ | 1.12 | 41.29 |  | 0.31 | 4.16 | ↓ |
| HOXC10 | homeobox C10 | 218959_at | 2.25 | 2.70 | ↑ | 0.79 | 43.50 |  | 1.37 | 35.63 |  |
| RAB11B | RAB11B, member RAS oncogene family | 34478_at | 2.25 | 1.90 | ↑ | 0.34 | 0.53 | ↓ | 1.17 | 41.22 |  |
| DLGAP4 | discs, large (Drosophila) homolog-associated protein 4 | 202571_s_at | 2.25 | 1.40 | ↑ | 0.95 | 59.12 |  | 1.30 | 32.58 |  |
| RHBDF2 | rhomboid 5 homolog 2 (Drosophila) | 219202_at | 2.24 | 3.54 | ↑ | — | — |  | — | — |  |
| SLC35A2 | solute carrier family 35 (UDP-galactose transporter), member A2 | 207440_at | 2.24 | 0.62 | ↑ | 0.63 | 6.11 |  | 0.78 | 17.70 |  |
| TBX2 | T-box 2 | 205993_s_at | 2.24 | 0.62 | ↑ | 0.70 | 12.66 |  | 0.83 | 20.38 |  |
| FST | follistatin | 204948_s_at | 2.24 | 1.60 | ↑ | 1.52 | 17.74 |  | 1.68 | 17.70 |  |
| GLCCI1 | glucocorticoid induced transcript 1 | 1560316_s_at | 2.24 | 3.90 | ↑ | — | — |  | — | — |  |
| ASTN2 | astrotactin 2 | 209693_at | 2.24 | 1.40 | ↑ | 1.25 | 29.01 |  | 0.79 | 23.22 |  |
| RAPGEF5 | Rap guanine nucleotide exchange factor (GEF) 5 | 204681_s_at | 2.24 | 3.21 | ↑ | 0.92 | 52.07 |  | 1.24 | 38.27 |  |
| BYSL | bystin-like | 203612_at | 2.24 | 0.90 | ↑ | 1.04 | 56.87 |  | 0.96 | 54.08 |  |
| ZNF667 | zinc finger protein 667 | 207120_at | 2.24 | 5.70 | ↑ | 1.83 | 24.54 |  | 0.39 | 1.99 | ↓ |
| ENPP2 | ectonucleotide pyrophosphatase/phosphodiesterase 2 | 210839_s_at | 2.24 | 0.00 | ↑ | 1.42 | 41.29 |  | 0.69 | 11.20 |  |
| ARL8A | ADP-ribosylation factor-like 8A | 225347_at | 2.23 | 1.40 | ↑ | 0.84 | 45.34 |  | 1.21 | 41.22 |  |
| IGFBP2 | insulin-like growth factor binding protein 2, 36kDa | 202718_at | 2.23 | 6.30 | ↑ | 1.20 | 53.46 |  | 0.38 | 13.22 |  |
| HNRNPUL1 | heterogeneous nuclear ribonucleoprotein U-like 1 | 209675_s_at | 2.23 | 1.40 | ↑ | 0.31 | 0.40 | ↓ | 0.83 | 43.83 |  |
| CD68 | CD68 molecule | 203507_at | 2.23 | 2.18 | ↑ | 1.05 | 54.70 |  | 1.16 | 52.56 |  |
| LOC100288778 /// WASH1 /// WASH2P /// WASH3P /// WASH7P | WAS protein family homolog 1 pseudogene /// WAS protein family homolog 1 /// WAS protein family homolog 2 pseudogene /// WAS protein family homolog 3 pseudogene /// WAS protein family homolog 7 pseudogene | 225035_x_at | 2.23 | 1.40 | ↑ | 1.11 | 53.46 |  | 0.86 | 32.58 |  |
| MTSS1 | metastasis suppressor 1 | 210360_s_at | 2.22 | 4.38 | ↑ | — | — |  | 2.16 | 5.75 | ↑ |
| AR | androgen receptor | 211621_at | 2.22 | 4.38 | ↑ | — | — |  | — | — |  |
| S1PR1 | sphingosine-1-phosphate receptor 1 | 204642_at | 2.22 | 8.24 | ↑ | 2.45 | 52.07 |  | 0.61 | 13.22 |  |
| IL27RA | interleukin 27 receptor, alpha | 222062_at | 2.22 | 3.90 | ↑ | 2.04 | 9.01 | ↑ | — | — |  |
| COL13A1 | collagen, type XIII, alpha 1 | 211343_s_at | 2.22 | 3.54 | ↑ | 1.17 | 47.13 |  | 1.99 | 15.27 |  |
| TCF3 | transcription factor 3 (E2A immunoglobulin enhancer binding factors E12/E47) | 215260_s_at | 2.22 | 0.90 | ↑ | 0.42 | 0.76 | ↓ | 0.83 | 26.48 |  |
| WNT5B | wingless-type MMTV integration site family, member 5B | 223537_s_at | 2.22 | 3.21 | ↑ | 0.18 | 0.25 | ↓ | 0.70 | 35.63 |  |
| WBP2 | WW domain binding protein 2 | 209117_at | 2.22 | 1.33 | ↑ | 0.48 | 2.80 | ↓ | 1.37 | 23.22 |  |
| OAF | OAF homolog (Drosophila) | 225510_at | 2.22 | 1.60 | ↑ | 0.47 | 3.81 | ↓ | 1.22 | 55.52 |  |
| NGLY1 | N-glycanase 1 | 207492_at | 2.22 | 3.21 | ↑ | — | — |  | — | — |  |
| GALK1 | galactokinase 1 | 204374_s_at | 2.22 | 3.90 | ↑ | 0.90 | 50.64 |  | 0.51 | 7.00 |  |
| ABCA2 | ATP-binding cassette, sub-family A (ABC1), member 2 | 212772_s_at | 2.22 | 2.18 | ↑ | 0.66 | 22.17 |  | 0.87 | 48.62 |  |
| FLJ39632 | hypothetical LOC642477 | 242546_at | 2.21 | 3.21 | ↑ | 2.14 | 10.02 |  | 0.95 | 43.83 |  |
| ZNF331 | zinc finger protein 331 | 227613_at | 2.21 | 0.00 | ↑ | 0.79 | 24.54 |  | 1.27 | 38.27 |  |
| RCC1 | regulator of chromosome condensation 1 | 215747_s_at | 2.21 | 2.18 | ↑ | 0.87 | 36.20 |  | 1.08 | 54.08 |  |
| TBC1D1 | TBC1 (tre-2/USP6, BUB2, cdc16) domain family, member 1 | 1568713_a_at | 2.21 | 4.38 | ↑ | — | — |  | — | — |  |
| RNF19B | ring finger protein 19B | 213038_at | 2.21 | 1.90 | ↑ | — | — |  | — | — |  |
| PTGS2 | prostaglandin-endoperoxide synthase 2 (prostaglandin G/H synthase and cyclooxygenase) | 1554997_a_at | 2.21 | 4.93 | ↑ | 1.95 | 38.78 |  | 0.33 | 2.43 | ↓ |
| ABCD1 | ATP-binding cassette, sub-family D (ALD), member 1 | 205142_x_at | 2.21 | 1.60 | ↑ | 0.81 | 36.20 |  | 1.28 | 38.27 |  |
| UNC5B | unc-5 homolog B (C. elegans) | 41856_at | 2.21 | 2.70 | ↑ | 1.52 | 19.82 |  | 0.41 | 5.75 | ↓ |
| LOC100130776 | hypothetical LOC100130776 | 1555907_at | 2.21 | 3.54 | ↑ | 0.60 | 12.66 |  | 0.33 | 0.72 | ↓ |
| LARP1 | La ribonucleoprotein domain family, member 1 | 210966_x_at | 2.20 | 1.40 | ↑ | 0.56 | 5.22 |  | 0.90 | 46.35 |  |
| CDKN1A | cyclin-dependent kinase inhibitor 1A (p21, Cip1) | 202284_s_at | 2.20 | 0.62 | ↑ | 0.98 | 50.64 |  | 1.72 | 1.43 |  |
| TMEFF2 | transmembrane protein with EGF-like and two follistatin-like domains 2 | 224321_at | 2.20 | 1.40 | ↑ | 5.28 | 0.00 | ↑ | 2.50 | 5.75 | ↑ |
| MFGE8 | milk fat globule-EGF factor 8 protein | 210605_s_at | 2.20 | 4.38 | ↑ | 0.71 | 17.74 |  | 1.69 | 43.83 |  |
| TNFRSF19 | tumor necrosis factor receptor superfamily, member 19 | 223827_at | 2.20 | 9.04 | ↑ | 0.36 | 1.26 | ↓ | 0.57 | 13.22 |  |
| HIC1 | hypermethylated in cancer 1 | 230218_at | 2.20 | 1.60 | ↑ | 1.06 | 50.64 |  | 0.90 | 43.83 |  |
| SMTN | smoothelin | 207390_s_at | 2.20 | 1.90 | ↑ | 0.44 | 3.35 | ↓ | 0.55 | 9.54 |  |
| BTBD2 | BTB (POZ) domain containing 2 | 207722_s_at | 2.20 | 3.54 | ↑ | 0.21 | 0.70 | ↓ | 0.75 | 50.73 |  |
| VWA3B | von Willebrand factor A domain containing 3B | 1555298_a_at | 2.19 | 3.54 | ↑ | — | — |  | — | — |  |
| TRIB2 | tribbles homolog 2 (Drosophila) | 202479_s_at | 2.19 | 0.90 | ↑ | 0.58 | 5.22 |  | 1.11 | 54.08 |  |
| RNF150 | ring finger protein 150 | 227657_at | 2.19 | 1.90 | ↑ | 1.94 | 14.26 |  | 1.17 | 38.27 |  |
| GPM6B | glycoprotein M6B | 209170_s_at | 2.19 | 5.70 | ↑ | — | — |  | — | — |  |
| CASKIN2 | CASK interacting protein 2 | 61297_at | 2.19 | 2.18 | ↑ | 1.20 | 43.50 |  | 0.70 | 17.70 |  |
| ERCC2 | excision repair cross-complementing rodent repair deficiency, complementation group 2 | 213468_at | 2.19 | 1.20 | ↑ | 0.74 | 24.54 |  | 0.91 | 48.62 |  |
| NPAS2 | neuronal PAS domain protein 2 | 39548_at | 2.19 | 2.70 | ↑ | 1.24 | 38.78 |  | 1.33 | 29.62 |  |
| MAP1S | microtubule-associated protein 1S | 218522_s_at | 2.19 | 1.40 | ↑ | 0.61 | 14.26 |  | 0.84 | 43.83 |  |
| EVL | Enah/Vasp-like | 217838_s_at | 2.19 | 1.33 | ↑ | 1.40 | 24.54 |  | 0.67 | 13.22 |  |
| POLR2A | polymerase (RNA) II (DNA directed) polypeptide A, 220kDa | 202725_at | 2.19 | 2.70 | ↑ | 0.78 | 49.04 |  | 1.28 | 35.63 |  |
| PLOD3 | procollagen-lysine, 2-oxoglutarate 5-dioxygenase 3 | 202185_at | 2.19 | 0.62 | ↑ | 1.00 | 58.42 |  | 0.62 | 2.70 |  |
| ELK1 | ELK1, member of ETS oncogene family | 210376_x_at | 2.19 | 1.33 | ↑ | 0.53 | 3.35 |  | 0.75 | 20.38 |  |
| GPR137 | G protein-coupled receptor 137 | 43934_at | 2.18 | 1.60 | ↑ | 0.62 | 9.01 |  | 1.07 | 55.52 |  |
| GPC4 | glypican 4 | 204983_s_at | 2.18 | 2.70 | ↑ | 2.38 | 3.81 | ↑ | 1.43 | 32.58 |  |
| HLA-DQB1 | major histocompatibility complex, class II, DQ beta 1 | 211654_x_at | 2.18 | 1.60 | ↑ | 2.49 | 14.26 |  | — | — |  |
| ARMC7 | armadillo repeat containing 7 | 229906_at | 2.18 | 1.90 | ↑ | 1.65 | 12.66 |  | 1.04 | 58.81 |  |
| SMARCD1 | SWI/SNF related, matrix associated, actin dependent regulator of chromatin, subfamily d, member 1 | 209518_at | 2.18 | 1.90 | ↑ | 1.24 | 33.65 |  | 1.66 | 15.27 |  |
| BCAS4 | breast carcinoma amplified sequence 4 | 220588_at | 2.17 | 1.40 | ↑ | 1.50 | 14.26 |  | 1.09 | 58.28 |  |
| PIK3R5 | phosphoinositide-3-kinase, regulatory subunit 5 | 220566_at | 2.17 | 2.70 | ↑ | 1.11 | 53.46 |  | 1.38 | 17.70 |  |
| PPP2R5B | protein phosphatase 2, regulatory subunit B', beta | 635_s_at | 2.17 | 3.54 | ↑ | 0.95 | 49.04 |  | 0.73 | 13.22 |  |
| LOC100510735 /// RPL29 | 60S ribosomal protein L29-like /// ribosomal protein L29 | 216570_x_at | 2.17 | 6.30 | ↑ | 1.79 | 4.45 |  | 0.89 | 32.58 |  |
| NFATC4 | nuclear factor of activated T-cells, cytoplasmic, calcineurin-dependent 4 | 213345_at | 2.17 | 2.70 | ↑ | 1.00 | 59.66 |  | 0.95 | 43.83 |  |
| TLE3 | transducin-like enhancer of split 3 (E(sp1) homolog, Drosophila) | 206472_s_at | 2.17 | 2.46 | ↑ | 0.77 | 26.77 |  | 0.48 | 2.43 | ↓ |
| MERTK | c-mer proto-oncogene tyrosine kinase | 211913_s_at | 2.16 | 5.70 | ↑ | 0.49 | 0.60 | ↓ | 1.38 | 9.54 |  |
| MMP14 | matrix metallopeptidase 14 (membrane-inserted) | 160020_at | 2.16 | 1.40 | ↑ | 1.99 | 7.87 |  | 1.23 | 54.08 |  |
| C1orf38 | chromosome 1 open reading frame 38 | 210785_s_at | 2.16 | 3.54 | ↑ | 0.92 | 52.07 |  | 0.36 | 2.70 | ↓ |
| MYPOP | Myb-related transcription factor, partner of profilin | 227937_at | 2.16 | 1.40 | ↑ | 0.95 | 58.42 |  | 0.75 | 23.22 |  |
| AKAP17A | A kinase (PRKA) anchor protein 17A | 228447_at | 2.16 | 9.04 | ↑ | — | — |  | 0.88 | 55.52 |  |
| CALR | calreticulin | 212953_x_at | 2.16 | 3.21 | ↑ | 2.26 | 4.45 | ↑ | — | — |  |
| SOLH | small optic lobes homolog (Drosophila) | 204275_at | 2.16 | 0.90 | ↑ | 0.39 | 0.70 | ↓ | 0.54 | 2.43 |  |
| VEGFA | vascular endothelial growth factor A | 210513_s_at | 2.16 | 3.90 | ↑ | 0.40 | 4.45 | ↓ | 0.48 | 9.54 | ↓ |
| TRIB3 | tribbles homolog 3 (Drosophila) | 218145_at | 2.16 | 7.26 | ↑ | 2.25 | 9.01 | ↑ | 0.57 | 43.83 |  |
| CPNE5 | copine V | 227189_at | 2.16 | 8.24 | ↑ | — | — |  | 1.24 | 43.83 |  |
| TNFRSF10B | tumor necrosis factor receptor superfamily, member 10b | 209294_x_at | 2.16 | 1.40 | ↑ | 0.34 | 0.60 | ↓ | 1.39 | 35.63 |  |
| SHB | Src homology 2 domain containing adaptor protein B | 204657_s_at | 2.16 | 3.21 | ↑ | 0.40 | 1.07 | ↓ | 0.85 | 41.22 |  |
| COMMD5 | COMM domain containing 5 | 232640_at | 2.15 | 3.54 | ↑ | 0.40 | 2.80 | ↓ | 1.81 | 9.54 |  |
| DLG4 | discs, large homolog 4 (Drosophila) | 204592_at | 2.15 | 2.70 | ↑ | 1.65 | 14.26 |  | 0.87 | 43.83 |  |
| PPP1R15A | protein phosphatase 1, regulatory (inhibitor) subunit 15A | 37028_at | 2.15 | 1.40 | ↑ | 0.48 | 1.98 | ↓ | 1.42 | 23.22 |  |
| MRC2 | mannose receptor, C type 2 | 209280_at | 2.15 | 1.40 | ↑ | 0.50 | 0.84 |  | 0.62 | 2.70 |  |
| PDXP /// SH3BP1 | pyridoxal (pyridoxine, vitamin B6) phosphatase /// SH3-domain binding protein 1 | 223290_at | 2.15 | 2.46 | ↑ | 0.82 | 47.13 |  | 0.67 | 17.70 |  |
| C16orf13 | chromosome 16 open reading frame 13 | 228114_x_at | 2.15 | 3.54 | ↑ | 0.75 | 24.54 |  | 0.74 | 15.27 |  |
| MEX3D | mex-3 homolog D (C. elegans) | 91816_f_at | 2.15 | 0.62 | ↑ | 2.83 | 0.70 | ↑ | 0.67 | 2.43 |  |
| DOCK6 | dedicator of cytokinesis 6 | 222003_s_at | 2.15 | 2.46 | ↑ | 0.49 | 5.22 | ↓ | 0.86 | 46.35 |  |
| CENPB | centromere protein B, 80kDa | 212437_at | 2.15 | 2.18 | ↑ | 0.46 | 4.45 | ↓ | 1.58 | 15.27 |  |
| CD24 | CD24 molecule | 266_s_at | 2.15 | 7.26 | ↑ | 2.31 | 55.85 |  | 0.19 | 2.43 | ↓ |
| TSKU | tsukushi small leucine rich proteoglycan homolog (Xenopus laevis) | 218245_at | 2.15 | 1.40 | ↑ | 0.63 | 6.11 |  | 0.82 | 23.22 |  |
| DMWD | dystrophia myotonica, WD repeat containing | 33768_at | 2.15 | 2.18 | ↑ | 0.69 | 15.95 |  | 1.16 | 46.35 |  |
| NFKB2 | nuclear factor of kappa light polypeptide gene enhancer in B-cells 2 (p49/p100) | 209636_at | 2.15 | 2.70 | ↑ | 0.53 | 7.87 |  | 0.95 | 58.81 |  |
| ZNF787 | zinc finger protein 787 | 213402_at | 2.15 | 1.60 | ↑ | 1.25 | 31.53 |  | 0.90 | 43.83 |  |
| LOC399491 | GPS, PLAT and transmembrane domain-containing protein | 214682_at | 2.15 | 9.04 | ↑ | 1.77 | 19.82 |  | 0.75 | 41.22 |  |
| NGF | nerve growth factor (beta polypeptide) | 206814_at | 2.14 | 1.40 | ↑ | 0.79 | 24.54 |  | 1.27 | 38.27 |  |
| PTK7 | PTK7 protein tyrosine kinase 7 | 207011_s_at | 2.14 | 1.90 | ↑ | 1.48 | 26.77 |  | 0.59 | 5.75 |  |
| MAP2K2 | mitogen-activated protein kinase kinase 2 | 202424_at | 2.14 | 1.40 | ↑ | 0.58 | 5.22 |  | 0.54 | 4.16 |  |
| LILRA5 | leukocyte immunoglobulin-like receptor, subfamily A (with TM domain), member 5 | 1555643_s_at | 2.14 | 9.04 | ↑ | 0.40 | 5.22 | ↓ | 0.47 | 13.22 |  |
| PTMS | parathymosin | 218045_x_at | 2.14 | 2.70 | ↑ | 0.69 | 36.20 |  | 0.95 | 58.81 |  |
| GOLGA6L4 /// PML | golgin A6 family-like 4 /// promyelocytic leukemia | 211014_s_at | 2.14 | 3.90 | ↑ | 0.48 | 1.74 | ↓ | 1.09 | 54.08 |  |
| LOC113230 | hypothetical LOC113230 | 1558412_at | 2.14 | 8.24 | ↑ | 0.46 | 0.76 | ↓ | 1.05 | 54.08 |  |
| LRSAM1 | leucine rich repeat and sterile alpha motif containing 1 | 227675_at | 2.14 | 0.90 | ↑ | 0.98 | 59.66 |  | 0.76 | 20.38 |  |
| TUBGCP3 | tubulin, gamma complex associated protein 3 | 1554086_at | 2.13 | 3.90 | ↑ | 0.94 | 53.46 |  | 0.69 | 15.27 |  |
| C16orf57 | chromosome 16 open reading frame 57 | 218060_s_at | 2.13 | 3.54 | ↑ | 0.92 | 59.66 |  | 1.28 | 35.63 |  |
| TANC1 | tetratricopeptide repeat, ankyrin repeat and coiled-coil containing 1 | 234870_at | 2.13 | 8.24 | ↑ | — | — |  | — | — |  |
| HCFC1R1 | host cell factor C1 regulator 1 (XPO1 dependent) | 45714_at | 2.13 | 7.26 | ↑ | 0.84 | 38.78 |  | 0.60 | 11.20 |  |
| ASS1 | argininosuccinate synthase 1 | 207076_s_at | 2.13 | 3.90 | ↑ | 1.19 | 45.34 |  | 1.06 | 56.10 |  |
| C16orf58 | chromosome 16 open reading frame 58 | 217891_at | 2.13 | 1.40 | ↑ | 0.30 | 0.18 | ↓ | 0.71 | 15.27 |  |
| DDHD1 | DDHD domain containing 1 | 244154_at | 2.13 | 9.04 | ↑ | 0.96 | 57.71 |  | 1.39 | 23.22 |  |
| TMEM63A | transmembrane protein 63A | 214833_at | 2.13 | 1.60 | ↑ | 0.93 | 50.64 |  | 0.91 | 46.35 |  |
| ENPP2 | ectonucleotide pyrophosphatase/phosphodiesterase 2 | 209392_at | 2.12 | 0.90 | ↑ | 1.72 | 15.95 |  | 0.69 | 11.20 |  |
| MAP1LC3A | microtubule-associated protein 1 light chain 3 alpha | 232011_s_at | 2.12 | 1.33 | ↑ | 0.75 | 22.17 |  | 1.28 | 23.22 |  |
| CLIP2 | CAP-GLY domain containing linker protein 2 | 211031_s_at | 2.12 | 1.60 | ↑ | 0.61 | 11.08 |  | 0.55 | 7.00 |  |
| FAM86B1 | family with sequence similarity 86, member B1 | 65585_at | 2.12 | 2.70 | ↑ | 0.70 | 33.65 |  | 0.75 | 35.63 |  |
| GALM | galactose mutarotase (aldose 1-epimerase) | 235256_s_at | 2.12 | 2.46 | ↑ | 1.39 | 19.82 |  | 0.82 | 29.62 |  |
| SYDE1 | synapse defective 1, Rho GTPase, homolog 1 (C. elegans) | 216271_x_at | 2.12 | 4.38 | ↑ | — | — |  | 1.21 | 46.35 |  |
| SEZ6L2 | seizure related 6 homolog (mouse)-like 2 | 223458_at | 2.12 | 3.21 | ↑ | 1.28 | 29.01 |  | 0.57 | 7.00 |  |
| ZNF785 | zinc finger protein 785 | 1554770_x_at | 2.12 | 4.93 | ↑ | 2.33 | 0.60 | ↑ | 0.72 | 13.22 |  |
| SLC2A8 | Solute carrier family 2 (facilitated glucose transporter), member 8 | 239426_at | 2.12 | 3.90 | ↑ | 1.61 | 14.26 |  | 2.01 | 15.27 |  |
| LOC100133612 | similar to hCG1815312 | 1569604_at | 2.12 | 3.21 | ↑ | 0.89 | 55.85 |  | 1.42 | 26.48 |  |
| PACS1 | phosphofurin acidic cluster sorting protein 1 | 224658_x_at | 2.11 | 1.20 | ↑ | 0.77 | 29.01 |  | 0.92 | 50.73 |  |
| PAPPA | pregnancy-associated plasma protein A, pappalysin 1 | 201982_s_at | 2.11 | 3.54 | ↑ | 0.66 | 17.74 |  | 2.39 | 35.63 |  |
| NRP2 | neuropilin 2 | 210841_s_at | 2.11 | 3.54 | ↑ | 0.41 | 3.35 | ↓ | 1.10 | 52.56 |  |
| RNF216 | ring finger protein 216 | 218425_at | 2.11 | 1.40 | ↑ | 1.06 | 50.64 |  | 0.88 | 43.83 |  |
| SLC2A4RG | SLC2A4 regulator | 222650_s_at | 2.11 | 3.54 | ↑ | 0.45 | 4.45 | ↓ | 0.70 | 29.62 |  |
| FAM84B | family with sequence similarity 84, member B | 225864_at | 2.11 | 3.90 | ↑ | 2.65 | 6.11 | ↑ | 1.05 | 38.27 |  |
| SFSWAP | splicing factor, suppressor of white-apricot homolog (Drosophila) | 202775_s_at | 2.10 | 0.62 | ↑ | 1.30 | 19.82 |  | 0.84 | 23.22 |  |
| SPEN | spen homolog, transcriptional regulator (Drosophila) | 1556058_s_at | 2.10 | 4.93 | ↑ | — | — |  | — | — |  |
| RAB33A | RAB33A, member RAS oncogene family | 206039_at | 2.10 | 9.04 | ↑ | 1.37 | 41.29 |  | 0.99 | 54.08 |  |
| IDUA | iduronidase, alpha-L- | 205059_s_at | 2.10 | 1.60 | ↑ | 0.74 | 19.82 |  | 0.84 | 32.58 |  |
| TLN1 | talin 1 | 203254_s_at | 2.10 | 4.38 | ↑ | 0.31 | 1.98 | ↓ | 0.80 | 50.73 |  |
| MAMSTR | MEF2 activating motif and SAP domain containing transcriptional regulator | 1557091_at | 2.10 | 9.04 | ↑ | 1.00 | 49.04 |  | 1.30 | 35.63 |  |
| KIDINS220 | kinase D-interacting substrate, 220kDa | 242032_at | 2.10 | 3.54 | ↑ | 1.57 | 14.26 |  | 1.04 | 50.73 |  |
| HOOK3 | hook homolog 3 (Drosophila) | 1558315_s_at | 2.10 | 2.70 | ↑ | 1.33 | 22.17 |  | 1.21 | 35.63 |  |
| FLJ39632 | hypothetical LOC642477 | 231882_at | 2.10 | 2.70 | ↑ | 1.71 | 15.95 |  | 0.88 | 32.58 |  |
| CALD1 | caldesmon 1 | 214880_x_at | 2.10 | 3.90 | ↑ | — | — |  | 1.62 | 4.16 |  |
| ITM2C | integral membrane protein 2C | 221004_s_at | 2.10 | 2.18 | ↑ | 1.42 | 29.01 |  | 0.43 | 0.89 | ↓ |
| LOC553103 | hypothetical LOC553103 | 241899_at | 2.09 | 5.70 | ↑ | — | — |  | — | — |  |
| ZBTB7A | zinc finger and BTB domain containing 7A | 226554_at | 2.09 | 0.62 | ↑ | 1.96 | 2.30 |  | 1.17 | 38.27 |  |
| SCAMP4 | secretory carrier membrane protein 4 | 213244_at | 2.09 | 2.18 | ↑ | 0.64 | 17.74 |  | 1.17 | 43.83 |  |
| CD151 | CD151 molecule (Raph blood group) | 204306_s_at | 2.09 | 0.90 | ↑ | 0.83 | 29.01 |  | 1.08 | 58.77 |  |
| --- | --- | 242326_at | 2.09 | 8.24 | ↑ | — | — |  | 1.32 | 35.63 |  |
| GATAD2B | GATA zinc finger domain containing 2B | 225393_at | 2.09 | 2.46 | ↑ | 0.66 | 26.77 |  | 1.23 | 32.58 |  |
| PDK2 | pyruvate dehydrogenase kinase, isozyme 2 | 202590_s_at | 2.09 | 0.90 | ↑ | 0.84 | 38.78 |  | 1.48 | 8.02 |  |
| DMWD | dystrophia myotonica, WD repeat containing | 213231_at | 2.09 | 1.60 | ↑ | 0.51 | 6.11 |  | 0.65 | 5.75 |  |
| LENG8 | leukocyte receptor cluster (LRC) member 8 | 224673_at | 2.08 | 1.60 | ↑ | 0.77 | 36.20 |  | 0.71 | 20.38 |  |
| DYRK1B | dual-specificity tyrosine-(Y)-phosphorylation regulated kinase 1B | 217270_s_at | 2.08 | 8.24 | ↑ | — | — |  | — | — |  |
| SHISA4 | shisa homolog 4 (Xenopus laevis) | 226674_at | 2.08 | 1.40 | ↑ | 1.38 | 17.74 |  | 1.16 | 48.62 |  |
| LARP1 | La ribonucleoprotein domain family, member 1 | 212193_s_at | 2.08 | 2.70 | ↑ | 0.31 | 0.45 | ↓ | 0.65 | 15.27 |  |
| RUNX3 | runt-related transcription factor 3 | 204198_s_at | 2.08 | 4.38 | ↑ | 0.94 | 55.85 |  | 0.33 | 4.84 | ↓ |
| FOXP4 | forkhead box P4 | 227120_at | 2.08 | 3.54 | ↑ | 0.66 | 29.01 |  | 0.43 | 4.16 | ↓ |
| SEPT9 | septin 9 | 208657_s_at | 2.08 | 0.90 | ↑ | 1.09 | 45.34 |  | 0.76 | 15.27 |  |
| TNFRSF10B | tumor necrosis factor receptor superfamily, member 10b | 210405_x_at | 2.08 | 1.40 | ↑ | 0.36 | 0.70 | ↓ | 1.38 | 32.58 |  |
| ACTN4 | actinin, alpha 4 | 200601_at | 2.08 | 7.26 | ↑ | 0.52 | 6.80 |  | 0.94 | 54.08 |  |
| SH3TC1 | SH3 domain and tetratricopeptide repeats 1 | 219256_s_at | 2.08 | 5.70 | ↑ | 0.98 | 49.04 |  | 0.47 | 11.20 |  |
| ARHGAP1 | Rho GTPase activating protein 1 | 216689_x_at | 2.08 | 5.70 | ↑ | 0.29 | 0.53 | ↓ | 1.58 | 11.20 |  |
| NPRL3 | nitrogen permease regulator-like 3 (S. cerevisiae) | 203289_s_at | 2.08 | 2.70 | ↑ | 0.36 | 2.80 | ↓ | 0.54 | 13.22 |  |
| ANGPTL2 | angiopoietin-like 2 | 219514_at | 2.08 | 2.46 | ↑ | 0.34 | 0.84 | ↓ | 0.89 | 41.22 |  |
| MAFB | v-maf musculoaponeurotic fibrosarcoma oncogene homolog B (avian) | 222670_s_at | 2.07 | 1.40 | ↑ | 0.82 | 33.65 |  | 0.71 | 17.70 |  |
| PEX14 | peroxisomal biogenesis factor 14 | 203503_s_at | 2.07 | 2.46 | ↑ | 0.69 | 14.26 |  | 1.30 | 41.22 |  |
| GDPD3 | glycerophosphodiester phosphodiesterase domain containing 3 | 219722_s_at | 2.07 | 3.90 | ↑ | — | — |  | — | — |  |
| VEGFA | vascular endothelial growth factor A | 212171_x_at | 2.07 | 6.30 | ↑ | 0.66 | 24.54 |  | 0.38 | 3.36 | ↓ |
| TNFRSF1B | tumor necrosis factor receptor superfamily, member 1B | 203508_at | 2.07 | 1.40 | ↑ | 1.05 | 59.66 |  | 0.77 | 20.38 |  |
| ARHGAP22 | Rho GTPase activating protein 22 | 206298_at | 2.07 | 3.90 | ↑ | 0.76 | 26.77 |  | 0.55 | 1.61 |  |
| BAP1 | BRCA1 associated protein-1 (ubiquitin carboxy-terminal hydrolase) | 201419_at | 2.07 | 1.40 | ↑ | 0.71 | 14.26 |  | 0.85 | 32.58 |  |
| TTC7A | tetratricopeptide repeat domain 7A | 224924_at | 2.07 | 2.46 | ↑ | 0.57 | 6.80 |  | 1.03 | 54.08 |  |
| MMP14 | matrix metallopeptidase 14 (membrane-inserted) | 202827_s_at | 2.07 | 4.93 | ↑ | 1.02 | 52.07 |  | 1.46 | 46.35 |  |
| FAM83H | family with sequence similarity 83, member H | 226129_at | 2.07 | 3.21 | ↑ | 1.54 | 19.82 |  | 0.65 | 13.22 |  |
| NINJ1 | ninjurin 1 | 203045_at | 2.07 | 1.90 | ↑ | 1.35 | 26.77 |  | 0.69 | 15.27 |  |
| ZFYVE27 | zinc finger, FYVE domain containing 27 | 225218_at | 2.06 | 1.40 | ↑ | 0.87 | 41.29 |  | 0.99 | 54.08 |  |
| SYNGR2 | synaptogyrin 2 | 201079_at | 2.06 | 3.21 | ↑ | 0.83 | 26.77 |  | 0.88 | 32.58 |  |
| STRN4 | striatin, calmodulin binding protein 4 | 217903_at | 2.06 | 1.60 | ↑ | 0.69 | 19.82 |  | 0.71 | 20.38 |  |
| GGT7 | gamma-glutamyltransferase 7 | 226469_s_at | 2.06 | 4.93 | ↑ | 2.41 | 9.01 | ↑ | 1.11 | 55.52 |  |
| TADA2A | transcriptional adaptor 2A | 209938_at | 2.06 | 9.04 | ↑ | 1.07 | 53.46 |  | 1.04 | 58.81 |  |
| FLJ42875 | hypothetical LOC440556 | 242269_at | 2.06 | 6.30 | ↑ | 1.14 | 38.78 |  | 1.31 | 43.83 |  |
| SMA4 | glucuronidase, beta pseudogene | 206565_x_at | 2.06 | 4.38 | ↑ | 1.02 | 60.31 |  | 1.16 | 52.56 |  |
| FAM133A | family with sequence similarity 133, member A | 239481_at | 2.06 | 3.90 | ↑ | 1.14 | 53.46 |  | 1.75 | 43.83 |  |
| CYSLTR1 | cysteinyl leukotriene receptor 1 | 216288_at | 2.06 | 9.04 | ↑ | 1.11 | 47.13 |  | 2.04 | 11.20 |  |
| NPC1 | Niemann-Pick disease, type C1 | 202679_at | 2.05 | 1.40 | ↑ | 1.45 | 26.77 |  | 0.88 | 29.62 |  |
| IRF2BP2 | interferon regulatory factor 2 binding protein 2 | 224572_s_at | 2.05 | 3.90 | ↑ | 0.96 | 50.64 |  | 0.57 | 5.75 |  |
| OGFR | opioid growth factor receptor | 211512_s_at | 2.05 | 3.21 | ↑ | 1.00 | 55.85 |  | 0.90 | 43.83 |  |
| ACSF3 | acyl-CoA synthetase family member 3 | 227464_at | 2.05 | 2.70 | ↑ | — | — |  | — | — |  |
| COL6A1 | collagen, type VI, alpha 1 | 212091_s_at | 2.05 | 3.21 | ↑ | 0.18 | 0.00 | ↓ | 0.56 | 11.20 |  |
| C22orf30 | chromosome 22 open reading frame 30 | 216555_at | 2.05 | 9.04 | ↑ | — | — |  | 1.22 | 43.83 |  |
| TRIM16L | tripartite motif-containing 16-like | 1559681_a_at | 2.05 | 3.54 | ↑ | 0.99 | 60.11 |  | 0.91 | 38.27 |  |
| CELSR3 | cadherin, EGF LAG seven-pass G-type receptor 3 (flamingo homolog, Drosophila) | 40020_at | 2.05 | 2.70 | ↑ | 0.98 | 60.31 |  | 0.87 | 35.63 |  |
| BCAM | basal cell adhesion molecule (Lutheran blood group) | 40093_at | 2.05 | 4.38 | ↑ | 1.10 | 59.12 |  | 0.80 | 23.22 |  |
| N4BP2L1 | NEDD4 binding protein 2-like 1 | 217197_x_at | 2.04 | 1.90 | ↑ | 0.96 | 49.04 |  | 1.30 | 35.63 |  |
| CTSL2 | cathepsin L2 | 210074_at | 2.04 | 7.26 | ↑ | 1.38 | 19.82 |  | 1.16 | 43.83 |  |
| ZSWIM1 | zinc finger, SWIM-type containing 1 | 223607_x_at | 2.04 | 4.38 | ↑ | 1.89 | 5.22 |  | 1.63 | 11.20 |  |
| CCDC71 | coiled-coil domain containing 71 | 219893_at | 2.04 | 8.24 | ↑ | 0.80 | 29.01 |  | 0.80 | 32.58 |  |
| MLL2 | myeloid/lymphoid or mixed-lineage leukemia 2 | 231974_at | 2.04 | 6.30 | ↑ | 0.55 | 31.53 |  | 1.09 | 52.56 |  |
| OR51B2 | olfactory receptor, family 51, subfamily B, member 2 | 234486_at | 2.04 | 8.24 | ↑ | — | — |  | 1.50 | 23.22 |  |
| TGFB1 | transforming growth factor, beta 1 | 203085_s_at | 2.04 | 3.90 | ↑ | 0.24 | 0.98 | ↓ | 0.45 | 13.22 |  |
| SSBP3 | single stranded DNA binding protein 3 | 217991_x_at | 2.04 | 3.90 | ↑ | 0.81 | 57.71 |  | 0.87 | 58.81 |  |
| PKD1 | polycystic kidney disease 1 (autosomal dominant) | 202328_s_at | 2.04 | 7.26 | ↑ | 0.82 | 45.34 |  | 0.79 | 38.27 |  |
| BCKDHA | branched chain keto acid dehydrogenase E1, alpha polypeptide | 202331_at | 2.04 | 4.93 | ↑ | 1.02 | 53.46 |  | 0.84 | 41.22 |  |
| ZNF555 | zinc finger protein 555 | 239839_at | 2.04 | 8.24 | ↑ | 1.62 | 15.95 |  | 1.17 | 52.56 |  |
| TRAF7 | TNF receptor-associated factor 7 | 223029_s_at | 2.03 | 2.46 | ↑ | 0.35 | 0.40 | ↓ | 1.11 | 50.73 |  |
| CNOT3 | CCR4-NOT transcription complex, subunit 3 | 211141_s_at | 2.03 | 7.26 | ↑ | — | — |  | — | — |  |
| VAMP2 | vesicle-associated membrane protein 2 (synaptobrevin 2) | 214792_x_at | 2.03 | 1.90 | ↑ | 0.31 | 0.00 | ↓ | 0.71 | 7.00 |  |
| SERBP1 | SERPINE1 mRNA binding protein 1 | 210076_x_at | 2.03 | 8.24 | ↑ | 1.32 | 29.01 |  | 2.38 | 1.43 | ↑ |
| UBTD1 | ubiquitin domain containing 1 | 219172_at | 2.03 | 3.54 | ↑ | 1.02 | 50.64 |  | 1.20 | 38.27 |  |
| ZBTB7A | zinc finger and BTB domain containing 7A | 219186_at | 2.03 | 1.40 | ↑ | 0.90 | 43.50 |  | 0.60 | 4.16 |  |
| TPM4 | tropomyosin 4 | 212481_s_at | 2.03 | 4.38 | ↑ | 0.68 | 33.65 |  | 0.90 | 58.81 |  |
| NFKB2 | nuclear factor of kappa light polypeptide gene enhancer in B-cells 2 (p49/p100) | 207535_s_at | 2.03 | 3.21 | ↑ | 0.49 | 6.11 | ↓ | 0.91 | 54.08 |  |
| LOC388152 /// LOC727751 /// LOC727849 /// LOC80154 | hypothetical LOC388152 /// hypothetical LOC727751 /// golgin A2 pseudogene /// hypothetical LOC80154 | 220602_s_at | 2.03 | 9.04 | ↑ | 1.42 | 24.54 |  | 0.53 | 38.27 |  |
| RAB27B | RAB27B, member RAS oncogene family | 207018_s_at | 2.03 | 6.30 | ↑ | 0.50 | 12.66 |  | 0.46 | 11.20 |  |
| AGPAT1 | 1-acylglycerol-3-phosphate O-acyltransferase 1 (lysophosphatidic acid acyltransferase, alpha) | 215535_s_at | 2.03 | 1.90 | ↑ | 0.74 | 31.53 |  | 0.93 | 52.56 |  |
| FOSL2 | FOS-like antigen 2 | 218880_at | 2.03 | 2.70 | ↑ | 0.67 | 17.74 |  | 0.67 | 15.27 |  |
| EFHD1 | EF-hand domain family, member D1 | 209343_at | 2.03 | 8.24 | ↑ | 2.50 | 9.01 | ↑ | 1.57 | 54.08 |  |
| MAP1LC3A | microtubule-associated protein 1 light chain 3 alpha | 227219_x_at | 2.03 | 0.62 | ↑ | 1.01 | 59.12 |  | 1.47 | 7.00 |  |
| ASAP1 | ArfGAP with SH3 domain, ankyrin repeat and PH domain 1 | 236533_at | 2.02 | 4.93 | ↑ | 2.13 | 3.81 | ↑ | 1.17 | 43.83 |  |
| INF2 | inverted formin, FH2 and WH2 domain containing | 218144_s_at | 2.02 | 1.33 | ↑ | 0.91 | 45.34 |  | 1.02 | 54.08 |  |
| WDR31 | WD repeat domain 31 | 241919_x_at | 2.02 | 7.26 | ↑ | 1.09 | 45.34 |  | 0.99 | 54.08 |  |
| CD24 | CD24 molecule | 208651_x_at | 2.02 | 7.26 | ↑ | 1.44 | 47.13 |  | 0.32 | 4.16 | ↓ |
| LHX4 | LIM homeobox 4 | 1553157_at | 2.02 | 8.24 | ↑ | 1.36 | 41.29 |  | 1.09 | 46.35 |  |
| NDRG1 | N-myc downstream regulated 1 | 200632_s_at | 2.02 | 8.24 | ↑ | 1.38 | 29.01 |  | 0.63 | 20.38 |  |
| MAF | v-maf musculoaponeurotic fibrosarcoma oncogene homolog (avian) | 209347_s_at | 2.02 | 8.24 | ↑ | — | — |  | — | — |  |
| LMNA | lamin A/C | 212089_at | 2.01 | 3.21 | ↑ | 0.75 | 19.82 |  | 1.17 | 43.83 |  |
| ANKRD13B | ankyrin repeat domain 13B | 227720_at | 2.01 | 1.33 | ↑ | 0.79 | 19.82 |  | 0.74 | 13.22 |  |
| CLASRP | CLK4-associating serine/arginine rich protein | 204978_at | 2.01 | 5.70 | ↑ | 1.18 | 36.20 |  | 1.35 | 26.48 |  |
| WASH1 /// WASH2P /// WASH3P /// WASH7P | WAS protein family homolog 1 /// WAS protein family homolog 2 pseudogene /// WAS protein family homolog 3 pseudogene /// WAS protein family homolog 7 pseudogene | 226340_x_at | 2.01 | 3.54 | ↑ | 1.62 | 11.08 |  | 1.05 | 58.81 |  |
| SERPINF1 | serpin peptidase inhibitor, clade F (alpha-2 antiplasmin, pigment epithelium derived factor), member 1 | 202283_at | 2.01 | 4.93 | ↑ | 0.80 | 38.78 |  | 0.50 | 8.02 | ↓ |
| LOC285735 | hypothetical LOC285735 | 1561691_at | 2.01 | 9.04 | ↑ | 1.15 | 43.50 |  | 0.77 | 26.48 |  |
| TMEM54 | transmembrane protein 54 | 225536_at | 2.01 | 3.90 | ↑ | 0.61 | 19.82 |  | 0.31 | 0.58 | ↓ |
| FST | follistatin | 226847_at | 2.01 | 3.90 | ↑ | 2.05 | 6.80 | ↑ | 1.86 | 9.54 |  |
| SLC16A3 | solute carrier family 16, member 3 (monocarboxylic acid transporter 4) | 202856_s_at | 2.01 | 4.93 | ↑ | 1.21 | 36.20 |  | 0.66 | 23.22 |  |
| SH2D3A | SH2 domain containing 3A | 222169_x_at | 2.01 | 3.54 | ↑ | 0.85 | 36.20 |  | 1.14 | 58.81 |  |
| RAVER1 | ribonucleoprotein, PTB-binding 1 | 223425_at | 2.01 | 3.54 | ↑ | 0.33 | 1.74 | ↓ | 0.54 | 9.54 |  |
| UBXN6 | UBX domain protein 6 | 220757_s_at | 2.01 | 0.62 | ↑ | 0.65 | 4.45 |  | 0.88 | 32.58 |  |
| GRAMD1B | GRAM domain containing 1B | 212906_at | 2.01 | 1.60 | ↑ | 1.08 | 54.70 |  | 1.14 | 43.83 |  |
| TBX18 | T-box 18 | 1559840_s_at | 2.01 | 5.70 | ↑ | 0.69 | 41.29 |  | 1.05 | 54.85 |  |
| MTA1 | metastasis associated 1 | 211783_s_at | 2.01 | 3.21 | ↑ | 1.17 | 38.78 |  | 0.76 | 23.22 |  |
| MAST2 | microtubule associated serine/threonine kinase 2 | 215660_s_at | 2.01 | 9.04 | ↑ | 0.22 | 0.53 | ↓ | 1.01 | 52.56 |  |
| ARID1A | AT rich interactive domain 1A (SWI-like) | 212152_x_at | 2.00 | 1.90 | ↑ | 0.35 | 0.45 | ↓ | 1.08 | 52.56 |  |
| DAB2IP | DAB2 interacting protein | 225020_at | 2.00 | 1.60 | ↑ | 0.68 | 12.66 |  | 0.82 | 29.62 |  |
| FAHD2A /// LOC285014 | fumarylacetoacetate hydrolase domain containing 2A /// hypothetical protein LOC285014 | 235621_at | 2.00 | 9.04 | ↑ | — | — |  | — | — |  |
| WASH3P | WAS protein family homolog 3 pseudogene | 233929_x_at | 2.00 | 1.33 | ↑ | 1.02 | 60.64 |  | 0.87 | 32.58 |  |
| TRIM61 | tripartite motif-containing 61 | 238990_x_at | 0.50 | 4.93 | ↓ | 0.77 | 26.77 |  | 0.81 | 26.48 |  |
| BIRC5 | baculoviral IAP repeat-containing 5 | 202095_s_at | 0.50 | 4.38 | ↓ | 0.63 | 9.01 |  | 0.05 | 0.00 | ↓ |
| NAPEPLD | N-acyl phosphatidylethanolamine phospholipase D | 226041_at | 0.50 | 9.04 | ↓ | 1.05 | 52.07 |  | 0.86 | 46.35 |  |
| RPL38 | ribosomal protein L38 | 202028_s_at | 0.50 | 3.21 | ↓ | 2.84 | 0.36 | ↑ | 1.36 | 26.48 |  |
| CSPP1 | centrosome and spindle pole associated protein 1 | 220072_at | 0.50 | 4.38 | ↓ | 1.10 | 49.04 |  | 0.78 | 20.38 |  |
| SPG7 | spastic paraplegia 7 (pure and complicated autosomal recessive) | 230885_at | 0.50 | 9.04 | ↓ | 2.68 | 0.70 | ↑ | 0.67 | 13.22 |  |
| SEC23IP | SEC23 interacting protein | 209176_at | 0.50 | 4.38 | ↓ | 0.95 | 53.46 |  | 0.94 | 48.62 |  |
| C9orf82 | chromosome 9 open reading frame 82 | 231995_at | 0.50 | 3.90 | ↓ | 1.08 | 50.64 |  | 0.91 | 43.83 |  |
| KIF15 | kinesin family member 15 | 219306_at | 0.50 | 4.38 | ↓ | 0.50 | 4.45 |  | 0.18 | 0.00 | ↓ |
| CAP2 | CAP, adenylate cyclase-associated protein, 2 (yeast) | 212554_at | 0.50 | 7.26 | ↓ | 1.10 | 43.50 |  | 1.69 | 9.54 |  |
| PIGY | phosphatidylinositol glycan anchor biosynthesis, class Y | 224661_at | 0.50 | 7.26 | ↓ | 0.97 | 56.87 |  | 0.61 | 9.54 |  |
| C1orf112 | chromosome 1 open reading frame 112 | 220840_s_at | 0.50 | 7.26 | ↓ | 0.73 | 24.54 |  | 0.49 | 1.99 | ↓ |
| CD59 | CD59 molecule, complement regulatory protein | 228748_at | 0.50 | 9.04 | ↓ | 0.62 | 15.95 |  | 3.76 | 0.19 | ↑ |
| ENY2 | enhancer of yellow 2 homolog (Drosophila) | 226775_at | 0.50 | 8.24 | ↓ | 0.81 | 33.65 |  | 1.18 | 43.83 |  |
| DCLK1 | doublecortin-like kinase 1 | 229800_at | 0.49 | 7.26 | ↓ | 2.02 | 9.01 | ↑ | 0.55 | 7.00 |  |
| LTV1 | LTV1 homolog (S. cerevisiae) | 239212_at | 0.49 | 4.38 | ↓ | 0.78 | 24.54 |  | 0.71 | 17.70 |  |
| MDM2 | Mdm2 p53 binding protein homolog (mouse) | 225160_x_at | 0.49 | 4.38 | ↓ | 0.95 | 58.42 |  | 1.03 | 58.28 |  |
| ZNF193 | zinc finger protein 193 | 239406_at | 0.49 | 9.04 | ↓ | 0.80 | 33.65 |  | 0.85 | 38.27 |  |
| CWC25 | CWC25 spliceosome-associated protein homolog (S. cerevisiae) | 222706_at | 0.49 | 4.93 | ↓ | 1.03 | 54.70 |  | 0.94 | 43.83 |  |
| MAP4K4 | mitogen-activated protein kinase kinase kinase kinase 4 | 222548_s_at | 0.49 | 1.40 | ↓ | 0.76 | 14.26 |  | 0.94 | 38.27 |  |
| FEN1 | flap structure-specific endonuclease 1 | 204767_s_at | 0.49 | 8.24 | ↓ | 1.04 | 59.12 |  | 0.36 | 0.50 | ↓ |
| SNRNP35 | small nuclear ribonucleoprotein 35kDa (U11/U12) | 205300_s_at | 0.49 | 5.70 | ↓ | 0.66 | 14.26 |  | 0.94 | 52.56 |  |
| LOC100192378 | hypothetical LOC100192378 | 1559965_at | 0.49 | 7.26 | ↓ | 1.54 | 17.74 |  | 0.79 | 35.63 |  |
| HNRNPM | heterogeneous nuclear ribonucleoprotein M | 200072_s_at | 0.49 | 4.38 | ↓ | 0.84 | 33.65 |  | 0.64 | 2.70 |  |
| GPATCH4 | G patch domain containing 4 | 224632_at | 0.49 | 4.38 | ↓ | 1.06 | 59.66 |  | 0.87 | 41.22 |  |
| DDX46 | DEAD (Asp-Glu-Ala-Asp) box polypeptide 46 | 202462_s_at | 0.49 | 3.54 | ↓ | 1.11 | 54.70 |  | 0.91 | 35.63 |  |
| ZNF814 | zinc finger protein 814 | 60794_f_at | 0.49 | 4.38 | ↓ | 0.54 | 2.80 |  | 0.82 | 26.48 |  |
| CSE1L | CSE1 chromosome segregation 1-like (yeast) | 201111_at | 0.49 | 4.38 | ↓ | 0.87 | 45.34 |  | 0.76 | 17.70 |  |
| ARSB | arylsulfatase B | 232197_x_at | 0.49 | 6.30 | ↓ | 3.08 | 1.74 | ↑ | 1.88 | 4.16 |  |
| CLSPN | claspin | 243840_at | 0.49 | 8.24 | ↓ | 0.70 | 15.95 |  | 0.41 | 1.25 | ↓ |
| TFG | TRK-fused gene | 221871_s_at | 0.49 | 5.70 | ↓ | 0.37 | 0.53 | ↓ | 1.60 | 5.75 |  |
| SCRN1 | secernin 1 | 201462_at | 0.49 | 4.93 | ↓ | 0.65 | 10.02 |  | 1.65 | 3.36 |  |
| MKLN1 | Muskelin 1, intracellular mediator containing kelch motifs | 1560145_at | 0.49 | 7.26 | ↓ | 1.23 | 38.78 |  | 0.87 | 32.58 |  |
| ACER3 | alkaline ceramidase 3 | 222688_at | 0.49 | 2.46 | ↓ | 1.00 | 60.11 |  | 0.92 | 41.22 |  |
| ALG13 | asparagine-linked glycosylation 13 homolog (S. cerevisiae) | 222808_at | 0.49 | 2.18 | ↓ | 1.65 | 33.65 |  | 1.06 | 58.81 |  |
| CCDC99 | coiled-coil domain containing 99 | 221685_s_at | 0.49 | 5.70 | ↓ | 0.83 | 36.20 |  | 0.76 | 23.22 |  |
| TMTC3 | transmembrane and tetratricopeptide repeat containing 3 | 226600_at | 0.49 | 4.93 | ↓ | 1.02 | 54.70 |  | 0.82 | 32.58 |  |
| USP25 | ubiquitin specific peptidase 25 | 223167_s_at | 0.49 | 4.38 | ↓ | 1.28 | 29.01 |  | 1.06 | 58.81 |  |
| CHCHD7 | coiled-coil-helix-coiled-coil-helix domain containing 7 | 218642_s_at | 0.49 | 5.70 | ↓ | 0.86 | 45.34 |  | 1.78 | 4.16 |  |
| CEPT1 | choline/ethanolamine phosphotransferase 1 | 1561884_at | 0.49 | 8.24 | ↓ | 0.51 | 3.35 |  | 0.93 | 43.83 |  |
| MED17 | mediator complex subunit 17 | 232483_at | 0.49 | 4.38 | ↓ | 1.10 | 55.85 |  | 0.69 | 11.20 |  |
| HNRNPA3 /// HNRNPA3P1 | heterogeneous nuclear ribonucleoprotein A3 /// heterogeneous nuclear ribonucleoprotein A3 pseudogene 1 | 211933_s_at | 0.49 | 2.70 | ↓ | 0.71 | 14.26 |  | 0.71 | 4.84 |  |
| GBP1 | guanylate binding protein 1, interferon-inducible, 67kDa | 231577_s_at | 0.49 | 4.38 | ↓ | 0.83 | 43.50 |  | 0.68 | 13.22 |  |
| ERAP1 | endoplasmic reticulum aminopeptidase 1 | 209788_s_at | 0.49 | 7.26 | ↓ | 1.01 | 58.42 |  | 0.68 | 11.20 |  |
| ASH1L | ash1 (absent, small, or homeotic)-like (Drosophila) | 222667_s_at | 0.49 | 1.33 | ↓ | 2.25 | 0.60 | ↑ | 1.16 | 41.22 |  |
| MCM2 | minichromosome maintenance complex component 2 | 202107_s_at | 0.49 | 9.04 | ↓ | 0.48 | 6.80 | ↓ | 0.32 | 0.43 | ↓ |
| FHL1 | four and a half LIM domains 1 | 214505_s_at | 0.49 | 8.24 | ↓ | 0.38 | 1.45 | ↓ | 1.27 | 54.08 |  |
| IGFBP7 | insulin-like growth factor binding protein 7 | 213910_at | 0.49 | 9.04 | ↓ | 1.62 | 11.08 |  | 0.76 | 26.48 |  |
| ID4 | inhibitor of DNA binding 4, dominant negative helix-loop-helix protein | 209291_at | 0.49 | 6.30 | ↓ | 2.15 | 7.87 | ↑ | 0.37 | 7.00 | ↓ |
| FAM122B | family with sequence similarity 122B | 222673_x_at | 0.49 | 3.90 | ↓ | 1.09 | 50.64 |  | 0.96 | 46.35 |  |
| BIVM | basic, immunoglobulin-like variable motif containing | 233255_s_at | 0.49 | 4.38 | ↓ | 0.87 | 45.34 |  | 0.96 | 54.08 |  |
| LOC642361 | hypothetical LOC642361 | 228839_s_at | 0.49 | 2.46 | ↓ | 1.32 | 29.01 |  | 0.90 | 32.58 |  |
| KLHL20 | kelch-like 20 (Drosophila) | 204176_at | 0.49 | 3.90 | ↓ | 1.41 | 22.17 |  | 1.19 | 43.83 |  |
| SRSF3 | serine/arginine-rich splicing factor 3 | 202899_s_at | 0.48 | 5.70 | ↓ | 0.77 | 29.01 |  | 0.78 | 26.48 |  |
| MCM8 | minichromosome maintenance complex component 8 | 224320_s_at | 0.48 | 6.30 | ↓ | 0.38 | 0.98 | ↓ | 0.45 | 1.61 | ↓ |
| MALAT1 | metastasis associated lung adenocarcinoma transcript 1 (non-protein coding) | 223940_x_at | 0.48 | 9.04 | ↓ | 2.89 | 3.81 | ↑ | 1.29 | 43.83 |  |
| --- | --- | 228259_s_at | 0.48 | 6.30 | ↓ | 0.80 | 31.53 |  | 1.33 | 43.83 |  |
| GLIPR1 | GLI pathogenesis-related 1 | 214085_x_at | 0.48 | 5.70 | ↓ | 0.83 | 26.77 |  | 0.72 | 11.20 |  |
| KIF23 | kinesin family member 23 | 204709_s_at | 0.48 | 8.24 | ↓ | 0.81 | 26.77 |  | 0.24 | 0.00 | ↓ |
| GLIS3 | GLIS family zinc finger 3 | 229435_at | 0.48 | 6.30 | ↓ | 0.74 | 31.53 |  | 1.04 | 57.64 |  |
| CUL2 | cullin 2 | 203078_at | 0.48 | 4.38 | ↓ | 1.19 | 52.07 |  | 1.01 | 54.08 |  |
| C13orf33 | chromosome 13 open reading frame 33 | 227058_at | 0.48 | 9.04 | ↓ | 1.05 | 52.07 |  | 1.60 | 17.70 |  |
| PLK4 | polo-like kinase 4 | 204887_s_at | 0.48 | 6.30 | ↓ | 0.51 | 4.45 |  | 0.10 | 0.00 | ↓ |
| ANKH | ankylosis, progressive homolog (mouse) | 223093_at | 0.48 | 8.24 | ↓ | 0.65 | 17.74 |  | 1.23 | 46.35 |  |
| LOC283788 | FSHD region gene 1 pseudogene | 229007_at | 0.48 | 9.04 | ↓ | 1.61 | 19.82 |  | 1.22 | 46.35 |  |
| CDCA8 | cell division cycle associated 8 | 221520_s_at | 0.48 | 9.04 | ↓ | 0.58 | 10.02 |  | 0.06 | 0.00 | ↓ |
| KIAA1324L | KIAA1324-like | 244317_at | 0.48 | 4.38 | ↓ | 0.63 | 10.02 |  | 1.04 | 54.08 |  |
| MBNL1 | muscleblind-like (Drosophila) | 1558111_at | 0.48 | 5.70 | ↓ | 0.57 | 6.80 |  | 0.67 | 15.27 |  |
| LUZP1 | leucine zipper protein 1 | 1558173_a_at | 0.48 | 7.26 | ↓ | 3.14 | 0.00 | ↑ | 0.80 | 15.27 |  |
| PPAPDC1B | phosphatidic acid phosphatase type 2 domain containing 1B | 226150_at | 0.48 | 3.90 | ↓ | 0.93 | 45.34 |  | 0.90 | 38.27 |  |
| ANKRD36B | ankyrin repeat domain 36B | 220940_at | 0.48 | 7.26 | ↓ | 2.00 | 12.66 |  | 0.56 | 8.02 |  |
| NAV1 | neuron navigator 1 | 224770_s_at | 0.48 | 5.70 | ↓ | 0.66 | 15.95 |  | 0.74 | 23.22 |  |
| CCNB1 | cyclin B1 | 228729_at | 0.48 | 4.93 | ↓ | 0.87 | 38.78 |  | 0.09 | 0.00 | ↓ |
| ZDHHC21 | zinc finger, DHHC-type containing 21 | 241946_at | 0.48 | 7.26 | ↓ | 0.43 | 0.84 | ↓ | 0.96 | 46.35 |  |
| CALD1 | Caldesmon 1 | 235834_at | 0.48 | 1.90 | ↓ | 1.02 | 60.11 |  | 1.89 | 4.84 |  |
| MED7 | mediator complex subunit 7 | 204349_at | 0.48 | 5.70 | ↓ | 1.36 | 29.01 |  | 0.97 | 58.81 |  |
| ISY1 | ISY1 splicing factor homolog (S. cerevisiae) | 223428_s_at | 0.48 | 4.38 | ↓ | 0.83 | 31.53 |  | 0.71 | 9.54 |  |
| TUBB2A | tubulin, beta 2A | 204141_at | 0.48 | 3.90 | ↓ | 1.14 | 60.11 |  | 0.67 | 11.20 |  |
| RPS9 | ribosomal protein S9 | 214317_x_at | 0.48 | 6.30 | ↓ | 0.94 | 55.85 |  | 0.43 | 1.25 | ↓ |
| ABCC10 | ATP-binding cassette, sub-family C (CFTR/MRP), member 10 | 213485_s_at | 0.48 | 4.38 | ↓ | 1.07 | 53.46 |  | 1.00 | 58.81 |  |
| FMN2 | formin 2 | 223618_at | 0.47 | 6.30 | ↓ | 0.92 | 45.34 |  | 1.97 | 1.99 |  |
| DNAJC9 | DnaJ (Hsp40) homolog, subfamily C, member 9 | 213092_x_at | 0.47 | 5.70 | ↓ | 0.96 | 60.11 |  | 0.69 | 15.27 |  |
| RACGAP1 | Rac GTPase activating protein 1 | 222077_s_at | 0.47 | 2.70 | ↓ | 0.74 | 15.95 |  | 0.30 | 0.00 | ↓ |
| SRSF1 | serine/arginine-rich splicing factor 1 | 201742_x_at | 0.47 | 8.24 | ↓ | 0.32 | 0.60 | ↓ | 0.67 | 20.38 |  |
| FHL1 | four and a half LIM domains 1 | 210298_x_at | 0.47 | 7.26 | ↓ | 0.45 | 3.81 | ↓ | 1.64 | 58.28 |  |
| LMBR1 | limb region 1 homolog (mouse) | 222506_at | 0.47 | 3.90 | ↓ | 0.84 | 36.20 |  | 0.87 | 32.58 |  |
| RBM25 | RNA binding motif protein 25 | 1557081_at | 0.47 | 2.18 | ↓ | 1.66 | 11.08 |  | 0.64 | 5.75 |  |
| USP1 | ubiquitin specific peptidase 1 | 202412_s_at | 0.47 | 3.90 | ↓ | 0.62 | 5.22 |  | 0.52 | 0.72 |  |
| ZC3H14 | zinc finger CCCH-type containing 14 | 213063_at | 0.47 | 4.38 | ↓ | 0.82 | 29.01 |  | 1.17 | 41.22 |  |
| SELT | selenoprotein T | 225561_at | 0.47 | 3.21 | ↓ | 0.88 | 45.34 |  | 1.36 | 23.22 |  |
| KIF14 | kinesin family member 14 | 206364_at | 0.47 | 4.93 | ↓ | 0.51 | 3.35 |  | 0.13 | 0.00 | ↓ |
| MED27 | mediator complex subunit 27 | 221598_s_at | 0.47 | 8.24 | ↓ | 1.00 | 60.11 |  | 0.63 | 4.84 |  |
| H2AFV | H2A histone family, member V | 212205_at | 0.47 | 2.70 | ↓ | 1.39 | 26.77 |  | 1.12 | 43.83 |  |
| LOC344595 | hypothetical LOC344595 | 235606_at | 0.47 | 8.24 | ↓ | 0.82 | 29.01 |  | 1.21 | 57.64 |  |
| SRSF3 | serine/arginine-rich splicing factor 3 | 208673_s_at | 0.47 | 6.30 | ↓ | 1.26 | 38.78 |  | 0.83 | 43.83 |  |
| PBK | PDZ binding kinase | 219148_at | 0.47 | 7.26 | ↓ | 0.81 | 29.01 |  | 0.06 | 0.00 | ↓ |
| FHL1 | four and a half LIM domains 1 | 201539_s_at | 0.47 | 5.70 | ↓ | 0.33 | 0.84 | ↓ | 1.56 | 58.81 |  |
| RIMKLB | ribosomal modification protein rimK-like family member B | 225978_at | 0.47 | 6.30 | ↓ | 1.08 | 50.64 |  | 0.92 | 52.56 |  |
| AURKA | aurora kinase A | 208079_s_at | 0.47 | 4.93 | ↓ | 0.54 | 6.11 |  | 0.20 | 0.00 | ↓ |
| DHFR | dihydrofolate reductase | 202534_x_at | 0.47 | 4.38 | ↓ | 1.45 | 36.20 |  | 0.47 | 0.00 | ↓ |
| VEPH1 | ventricular zone expressed PH domain homolog 1 (zebrafish) | 232122_s_at | 0.46 | 4.93 | ↓ | 0.60 | 11.08 |  | 1.04 | 55.52 |  |
| ANP32E | acidic (leucine-rich) nuclear phosphoprotein 32 family, member E | 208103_s_at | 0.46 | 3.90 | ↓ | 0.87 | 43.50 |  | 0.64 | 9.54 |  |
| KLF3 | Kruppel-like factor 3 (basic) | 225133_at | 0.46 | 5.70 | ↓ | 0.91 | 59.66 |  | 0.79 | 35.63 |  |
| TOP1 | topoisomerase (DNA) I | 208900_s_at | 0.46 | 4.93 | ↓ | 2.19 | 0.84 | ↑ | 0.62 | 4.16 |  |
| BMP2K | BMP2 inducible kinase | 219546_at | 0.46 | 6.30 | ↓ | 0.97 | 49.04 |  | 1.41 | 15.27 |  |
| ANKRD11 | ankyrin repeat domain 11 | 234701_at | 0.46 | 4.38 | ↓ | 5.99 | 0.00 | ↑ | 0.51 | 3.36 |  |
| FAM83D | family with sequence similarity 83, member D | 225687_at | 0.46 | 9.04 | ↓ | 0.63 | 12.66 |  | 0.12 | 0.00 | ↓ |
| STRA13 | stimulated by retinoic acid 13 homolog (mouse) | 209478_at | 0.46 | 2.18 | ↓ | 0.88 | 38.78 |  | 0.54 | 1.43 |  |
| SHCBP1 | SHC SH2-domain binding protein 1 | 219493_at | 0.46 | 4.93 | ↓ | 0.69 | 12.66 |  | 0.09 | 0.00 | ↓ |
| NOL7 | nucleolar protein 7, 27kDa | 213838_at | 0.46 | 1.60 | ↓ | 1.15 | 47.13 |  | 1.12 | 46.35 |  |
| FKBP1B | FK506 binding protein 1B, 12.6 kDa | 206857_s_at | 0.46 | 4.38 | ↓ | 0.80 | 38.78 |  | 1.09 | 58.51 |  |
| BIRC5 | baculoviral IAP repeat-containing 5 | 202094_at | 0.46 | 6.30 | ↓ | 0.59 | 6.80 |  | 0.07 | 0.00 | ↓ |
| FRMD5 | FERM domain containing 5 | 230831_at | 0.46 | 9.04 | ↓ | 0.38 | 6.11 | ↓ | 0.65 | 29.62 |  |
| ASPM | asp (abnormal spindle) homolog, microcephaly associated (Drosophila) | 219918_s_at | 0.46 | 4.38 | ↓ | 0.73 | 14.26 |  | 0.11 | 0.00 | ↓ |
| CDC6 | cell division cycle 6 homolog (S. cerevisiae) | 203967_at | 0.46 | 6.30 | ↓ | 0.85 | 38.78 |  | 0.15 | 0.00 | ↓ |
| C2orf3 | chromosome 2 open reading frame 3 | 210175_at | 0.46 | 2.46 | ↓ | 0.93 | 45.34 |  | 1.01 | 58.81 |  |
| TAF15 | TAF15 RNA polymerase II, TATA box binding protein (TBP)-associated factor, 68kDa | 202840_at | 0.46 | 4.38 | ↓ | 2.72 | 0.70 | ↑ | 0.85 | 38.27 |  |
| NIPBL | Nipped-B homolog (Drosophila) | 213918_s_at | 0.46 | 2.18 | ↓ | 1.03 | 60.11 |  | 0.99 | 54.08 |  |
| SLC5A3 | solute carrier family 5 (sodium/myo-inositol cotransporter), member 3 | 212944_at | 0.46 | 9.04 | ↓ | 1.16 | 36.20 |  | 0.50 | 7.00 | ↓ |
| PSMC2 | proteasome (prosome, macropain) 26S subunit, ATPase, 2 | 201067_at | 0.46 | 1.60 | ↓ | 0.51 | 1.45 |  | 1.29 | 23.22 |  |
| PLCD4 | phospholipase C, delta 4 | 224505_s_at | 0.46 | 9.04 | ↓ | 0.53 | 9.01 |  | 0.40 | 4.16 | ↓ |
| BMP2K | BMP2 inducible kinase | 59644_at | 0.46 | 1.90 | ↓ | 0.97 | 49.04 |  | 1.46 | 20.38 |  |
| NUB1 | negative regulator of ubiquitin-like proteins 1 | 1569030_s_at | 0.46 | 1.60 | ↓ | 1.20 | 52.07 |  | 0.87 | 35.63 |  |
| CASP6 | caspase 6, apoptosis-related cysteine peptidase | 209790_s_at | 0.46 | 1.90 | ↓ | 0.78 | 22.17 |  | 0.61 | 1.25 |  |
| HNRNPD | heterogeneous nuclear ribonucleoprotein D (AU-rich element RNA binding protein 1, 37kDa) | 221480_at | 0.45 | 1.40 | ↓ | 1.07 | 60.11 |  | 1.21 | 41.22 |  |
| NFIA | nuclear factor I/A | 224975_at | 0.45 | 7.26 | ↓ | 0.93 | 49.04 |  | 1.20 | 50.73 |  |
| GDAP2 | ganglioside induced differentiation associated protein 2 | 1554154_at | 0.45 | 6.30 | ↓ | 0.51 | 6.11 |  | 0.62 | 15.27 |  |
| EIF5B | eukaryotic translation initiation factor 5B | 201026_at | 0.45 | 3.21 | ↓ | 3.23 | 0.29 | ↑ | 1.01 | 58.81 |  |
| NCAPG | non-SMC condensin I complex, subunit G | 218663_at | 0.45 | 7.26 | ↓ | 0.74 | 17.74 |  | 0.06 | 0.00 | ↓ |
| TADA2B | transcriptional adaptor 2B | 236248_x_at | 0.45 | 1.40 | ↓ | 0.91 | 43.50 |  | 0.89 | 35.63 |  |
| PRDX2 | peroxiredoxin 2 | 211658_at | 0.45 | 5.70 | ↓ | 0.40 | 1.45 | ↓ | 0.77 | 32.58 |  |
| SMARCE1 | SWI/SNF related, matrix associated, actin dependent regulator of chromatin, subfamily e, member 1 | 229511_at | 0.45 | 5.70 | ↓ | 1.01 | 54.70 |  | 0.92 | 41.22 |  |
| HERPUD2 | HERPUD family member 2 | 1558699_a_at | 0.45 | 4.93 | ↓ | 0.83 | 47.13 |  | 0.65 | 13.22 |  |
| PHACTR2 | phosphatase and actin regulator 2 | 204048_s_at | 0.45 | 5.70 | ↓ | 1.09 | 49.04 |  | 2.75 | 0.58 | ↑ |
| HMMR | hyaluronan-mediated motility receptor (RHAMM) | 207165_at | 0.45 | 3.90 | ↓ | 0.79 | 22.17 |  | 0.03 | 0.00 | ↓ |
| LPXN | leupaxin | 216250_s_at | 0.45 | 4.38 | ↓ | 0.43 | 1.74 | ↓ | 1.50 | 48.62 |  |
| HCG11 | HLA complex group 11 | 1557169_x_at | 0.45 | 5.70 | ↓ | 1.36 | 22.17 |  | 0.87 | 43.83 |  |
| CCNB1 | cyclin B1 | 214710_s_at | 0.45 | 4.38 | ↓ | 0.68 | 14.26 |  | 0.11 | 0.00 | ↓ |
| LOC150759 | hypothetical LOC150759 | 213703_at | 0.45 | 3.90 | ↓ | 1.50 | 22.17 |  | 0.73 | 9.54 |  |
| ITFG2 | integrin alpha FG-GAP repeat containing 2 | 226295_at | 0.45 | 9.04 | ↓ | 0.59 | 11.08 |  | 0.83 | 35.63 |  |
| FAM54A | family with sequence similarity 54, member A | 228069_at | 0.45 | 5.70 | ↓ | 0.74 | 24.54 |  | 0.28 | 0.00 | ↓ |
| HAPLN1 | hyaluronan and proteoglycan link protein 1 | 205523_at | 0.45 | 9.04 | ↓ | 0.74 | 15.95 |  | 1.05 | 58.81 |  |
| NDP | Norrie disease (pseudoglioma) | 206022_at | 0.45 | 7.26 | ↓ | 0.77 | 26.77 |  | 0.84 | 32.58 |  |
| ZDHHC20 | zinc finger, DHHC-type containing 20 | 225365_at | 0.45 | 4.93 | ↓ | 0.49 | 2.80 | ↓ | 1.17 | 43.83 |  |
| SHFM1 | split hand/foot malformation (ectrodactyly) type 1 | 202276_at | 0.45 | 1.90 | ↓ | 0.56 | 2.80 |  | 0.71 | 11.20 |  |
| LOC100130219 | hypothetical LOC100130219 | 236042_at | 0.45 | 9.04 | ↓ | 0.99 | 60.64 |  | 0.79 | 20.38 |  |
| COPG2 | coatomer protein complex, subunit gamma 2 | 223457_at | 0.45 | 3.54 | ↓ | 1.62 | 11.08 |  | 0.91 | 41.22 |  |
| LXN | latexin | 218729_at | 0.45 | 4.38 | ↓ | 0.74 | 31.53 |  | 0.22 | 0.00 | ↓ |
| TRIM2 | tripartite motif-containing 2 | 215945_s_at | 0.45 | 6.30 | ↓ | 0.66 | 6.80 |  | 1.45 | 5.75 |  |
| PL-5283 | PL-5283 protein | 224758_at | 0.45 | 8.24 | ↓ | 0.88 | 56.87 |  | 1.08 | 54.08 |  |
| NDUFS1 | NADH dehydrogenase (ubiquinone) Fe-S protein 1, 75kDa (NADH-coenzyme Q reductase) | 236356_at | 0.44 | 1.33 | ↓ | 1.89 | 3.81 |  | 0.96 | 46.35 |  |
| POGK | pogo transposable element with KRAB domain | 239392_s_at | 0.44 | 7.26 | ↓ | 1.74 | 9.01 |  | 1.33 | 29.62 |  |
| RSPH10B2 | radial spoke head 10 homolog B2 (Chlamydomonas) | 1555272_at | 0.44 | 1.40 | ↓ | 1.58 | 22.17 |  | 1.43 | 29.62 |  |
| BCLAF1 | BCL2-associated transcription factor 1 | 239897_at | 0.44 | 2.18 | ↓ | 0.98 | 59.66 |  | 1.04 | 57.14 |  |
| ARNTL | aryl hydrocarbon receptor nuclear translocator-like | 209824_s_at | 0.44 | 6.30 | ↓ | 0.76 | 26.77 |  | 0.80 | 23.22 |  |
| GINS2 | GINS complex subunit 2 (Psf2 homolog) | 221521_s_at | 0.44 | 9.04 | ↓ | 0.65 | 22.17 |  | 0.14 | 0.50 | ↓ |
| ABHD3 | abhydrolase domain containing 3 | 213017_at | 0.44 | 7.26 | ↓ | 0.72 | 29.01 |  | 0.23 | 0.00 | ↓ |
| SMOC1 | SPARC related modular calcium binding 1 | 222783_s_at | 0.44 | 7.26 | ↓ | 1.76 | 33.65 |  | 0.63 | 15.27 |  |
| NEAT1 | nuclear paraspeckle assembly transcript 1 (non-protein coding) | 224566_at | 0.44 | 3.90 | ↓ | 0.58 | 6.11 |  | 1.34 | 26.48 |  |
| GREM2 | gremlin 2 | 240509_s_at | 0.44 | 6.30 | ↓ | 0.79 | 38.78 |  | 1.15 | 56.10 |  |
| DTL | denticleless homolog (Drosophila) | 218585_s_at | 0.44 | 4.38 | ↓ | 0.68 | 17.74 |  | 0.06 | 0.00 | ↓ |
| CDK1 | cyclin-dependent kinase 1 | 203213_at | 0.44 | 4.38 | ↓ | 0.61 | 6.80 |  | 0.05 | 0.00 | ↓ |
| RFC3 | replication factor C (activator 1) 3, 38kDa | 204127_at | 0.44 | 2.70 | ↓ | 0.76 | 24.54 |  | 0.40 | 0.00 | ↓ |
| C4orf46 | chromosome 4 open reading frame 46 | 238015_at | 0.44 | 5.70 | ↓ | 0.53 | 3.81 |  | 0.54 | 1.43 |  |
| NUF2 | NUF2, NDC80 kinetochore complex component, homolog (S. cerevisiae) | 223381_at | 0.44 | 3.90 | ↓ | 0.58 | 7.87 |  | 0.08 | 0.00 | ↓ |
| TPCN2 | two pore segment channel 2 | 235194_at | 0.44 | 5.70 | ↓ | 0.38 | 1.98 | ↓ | 1.00 | 54.08 |  |
| REV3L | REV3-like, catalytic subunit of DNA polymerase zeta (yeast) | 238736_at | 0.44 | 5.70 | ↓ | 0.92 | 49.04 |  | 0.96 | 52.56 |  |
| ETS2 | v-ets erythroblastosis virus E26 oncogene homolog 2 (avian) | 201329_s_at | 0.43 | 7.26 | ↓ | 0.34 | 0.98 | ↓ | 1.08 | 54.08 |  |
| HNRNPA3 /// HNRNPA3P1 | heterogeneous nuclear ribonucleoprotein A3 /// heterogeneous nuclear ribonucleoprotein A3 pseudogene 1 | 206809_s_at | 0.43 | 4.38 | ↓ | 0.57 | 9.01 |  | 0.60 | 4.84 |  |
| KCNQ5 | potassium voltage-gated channel, KQT-like subfamily, member 5 | 244623_at | 0.43 | 9.04 | ↓ | 1.31 | 57.71 |  | 0.93 | 43.83 |  |
| DLGAP5 | discs, large (Drosophila) homolog-associated protein 5 | 203764_at | 0.43 | 4.93 | ↓ | 0.81 | 26.77 |  | 0.07 | 0.00 | ↓ |
| ANLN | anillin, actin binding protein | 1552619_a_at | 0.43 | 7.26 | ↓ | 0.42 | 2.80 | ↓ | 0.06 | 0.00 | ↓ |
| MCM10 | minichromosome maintenance complex component 10 | 220651_s_at | 0.43 | 9.04 | ↓ | 0.73 | 31.53 |  | 0.06 | 0.00 | ↓ |
| UBE3C | ubiquitin protein ligase E3C | 1554793_at | 0.43 | 7.26 | ↓ | 0.88 | 33.65 |  | 1.06 | 54.85 |  |
| API5 | apoptosis inhibitor 5 | 233078_at | 0.43 | 8.24 | ↓ | 1.10 | 43.50 |  | 1.26 | 57.92 |  |
| CCDC50 | coiled-coil domain containing 50 | 226713_at | 0.43 | 4.38 | ↓ | 0.66 | 14.26 |  | 2.10 | 2.70 | ↑ |
| GINS3 | GINS complex subunit 3 (Psf3 homolog) | 45633_at | 0.43 | 4.93 | ↓ | 1.10 | 50.64 |  | 0.44 | 2.43 | ↓ |
| LRRC14 | leucine rich repeat containing 14 | 32062_at | 0.43 | 4.93 | ↓ | 1.08 | 52.07 |  | 0.82 | 32.58 |  |
| N4BP2L2 | NEDD4 binding protein 2-like 2 | 214748_at | 0.43 | 4.38 | ↓ | 1.36 | 33.65 |  | 2.24 | 1.61 | ↑ |
| PPM1A | protein phosphatase, Mg2+/Mn2+ dependent, 1A | 229027_at | 0.43 | 1.60 | ↓ | 1.03 | 55.85 |  | 0.96 | 50.73 |  |
| ENY2 | enhancer of yellow 2 homolog (Drosophila) | 226776_at | 0.43 | 7.26 | ↓ | 1.18 | 49.04 |  | 1.32 | 29.62 |  |
| GPR39 | G protein-coupled receptor 39 | 229105_at | 0.42 | 5.70 | ↓ | 0.57 | 6.80 |  | 2.03 | 4.16 | ↑ |
| RPP30 | ribonuclease P/MRP 30kDa subunit | 1556062_at | 0.42 | 4.93 | ↓ | 0.72 | 17.74 |  | 1.22 | 54.08 |  |
| RBM25 | RNA binding motif protein 25 | 212027_at | 0.42 | 1.40 | ↓ | 1.04 | 53.46 |  | 0.67 | 2.43 |  |
| ZWILCH | Zwilch, kinetochore associated, homolog (Drosophila) | 218349_s_at | 0.42 | 1.60 | ↓ | 0.22 | 0.00 | ↓ | 0.42 | 0.00 | ↓ |
| FAR2 | fatty acyl CoA reductase 2 | 220615_s_at | 0.42 | 9.04 | ↓ | 0.44 | 6.11 | ↓ | 0.67 | 26.48 |  |
| VPS13D | vacuolar protein sorting 13 homolog D (S. cerevisiae) | 212326_at | 0.42 | 7.26 | ↓ | 0.58 | 6.80 |  | 1.14 | 43.83 |  |
| HAPLN1 | hyaluronan and proteoglycan link protein 1 | 230204_at | 0.42 | 7.26 | ↓ | 0.73 | 15.95 |  | 0.93 | 43.83 |  |
| KIAA1609 | KIAA1609 | 233423_at | 0.42 | 8.24 | ↓ | 0.58 | 12.66 |  | 0.98 | 38.27 |  |
| HNRNPR | heterogeneous nuclear ribonucleoprotein R | 208765_s_at | 0.42 | 2.46 | ↓ | 0.60 | 4.45 |  | 0.52 | 0.58 |  |
| NMT2 | N-myristoyltransferase 2 | 215743_at | 0.42 | 6.30 | ↓ | 1.22 | 50.64 |  | 1.51 | 23.22 |  |
| EDNRA | endothelin receptor type A | 204463_s_at | 0.42 | 8.24 | ↓ | 0.40 | 3.35 | ↓ | 0.85 | 48.62 |  |
| ADK | adenosine kinase | 204120_s_at | 0.42 | 1.60 | ↓ | 1.06 | 59.12 |  | 0.87 | 29.62 |  |
| MPHOSPH9 | M-phase phosphoprotein 9 | 215731_s_at | 0.42 | 1.40 | ↓ | 1.12 | 49.04 |  | 0.86 | 26.48 |  |
| PTGR1 | Prostaglandin reductase 1 | 242775_at | 0.42 | 1.90 | ↓ | 0.74 | 22.17 |  | 0.61 | 7.00 |  |
| HMGN5 | high-mobility group nucleosome binding domain 5 | 221606_s_at | 0.42 | 1.09 | ↓ | 0.83 | 31.53 |  | 0.87 | 29.62 |  |
| MTMR9 | myotubularin related protein 9 | 204837_at | 0.42 | 1.33 | ↓ | 1.27 | 24.54 |  | 1.45 | 23.22 |  |
| SRSF1 | serine/arginine-rich splicing factor 1 | 211784_s_at | 0.42 | 1.40 | ↓ | 1.15 | 43.50 |  | 0.84 | 26.48 |  |
| MORF4L2 | Mortality factor 4 like 2 | 243683_at | 0.42 | 6.30 | ↓ | 0.53 | 11.08 |  | 0.90 | 52.56 |  |
| SOCS6 | suppressor of cytokine signaling 6 | 206020_at | 0.42 | 8.24 | ↓ | 0.89 | 43.50 |  | 1.30 | 20.38 |  |
| LOC284926 | hypothetical protein LOC284926 | 1556064_at | 0.41 | 6.30 | ↓ | 0.99 | 60.11 |  | 0.94 | 52.56 |  |
| SRSF1 | serine/arginine-rich splicing factor 1 | 208863_s_at | 0.41 | 3.21 | ↓ | 0.70 | 19.82 |  | 0.55 | 2.70 |  |
| HAPLN1 | hyaluronan and proteoglycan link protein 1 | 205524_s_at | 0.41 | 7.26 | ↓ | 0.52 | 6.11 |  | 1.03 | 52.56 |  |
| SYNCRIP | synaptotagmin binding, cytoplasmic RNA interacting protein | 209025_s_at | 0.41 | 1.90 | ↓ | 1.61 | 17.74 |  | 1.16 | 46.35 |  |
| RPP30 | ribonuclease P/MRP 30kDa subunit | 1556063_s_at | 0.41 | 4.38 | ↓ | 0.66 | 10.02 |  | 1.21 | 38.27 |  |
| ITGA4 | integrin, alpha 4 (antigen CD49D, alpha 4 subunit of VLA-4 receptor) | 205884_at | 0.41 | 9.04 | ↓ | 0.49 | 6.11 | ↓ | 2.71 | 2.70 | ↑ |
| ADI1 | acireductone dioxygenase 1 | 217761_at | 0.41 | 4.38 | ↓ | 1.00 | 58.42 |  | 1.62 | 13.22 |  |
| CEP55 | centrosomal protein 55kDa | 218542_at | 0.41 | 4.93 | ↓ | 0.59 | 10.02 |  | 0.08 | 0.00 | ↓ |
| CCNE2 | cyclin E2 | 205034_at | 0.41 | 5.70 | ↓ | 0.77 | 33.65 |  | 0.11 | 0.00 | ↓ |
| CNTNAP3 | contactin associated protein-like 3 | 223796_at | 0.41 | 9.04 | ↓ | 1.01 | 55.85 |  | 0.40 | 1.61 | ↓ |
| ROR1 | receptor tyrosine kinase-like orphan receptor 1 | 232060_at | 0.41 | 3.54 | ↓ | 1.78 | 24.54 |  | 2.36 | 3.36 | ↑ |
| CKS2 | CDC28 protein kinase regulatory subunit 2 | 204170_s_at | 0.41 | 1.20 | ↓ | 0.85 | 31.53 |  | 0.25 | 0.00 | ↓ |
| HNMT | histamine N-methyltransferase | 228772_at | 0.41 | 7.26 | ↓ | 1.23 | 33.65 |  | 0.45 | 4.16 | ↓ |
| NSD1 | nuclear receptor binding SET domain protein 1 | 243612_at | 0.41 | 3.90 | ↓ | 0.53 | 7.87 |  | 0.78 | 32.58 |  |
| FBXO5 | F-box protein 5 | 218875_s_at | 0.40 | 2.18 | ↓ | 0.98 | 49.04 |  | 0.28 | 0.00 | ↓ |
| GINS1 | GINS complex subunit 1 (Psf1 homolog) | 206102_at | 0.40 | 3.90 | ↓ | 0.79 | 29.01 |  | 0.18 | 0.00 | ↓ |
| CYP27C1 | cytochrome P450, family 27, subfamily C, polypeptide 1 | 1568868_at | 0.40 | 6.30 | ↓ | 1.40 | 17.74 |  | 0.59 | 8.02 |  |
| PKIA | protein kinase (cAMP-dependent, catalytic) inhibitor alpha | 226864_at | 0.40 | 4.38 | ↓ | 0.70 | 22.17 |  | 0.61 | 11.20 |  |
| BDH2 | 3-hydroxybutyrate dehydrogenase, type 2 | 235155_at | 0.40 | 8.24 | ↓ | 0.77 | 41.29 |  | 1.05 | 58.28 |  |
| OR2A20P /// OR2A9P | olfactory receptor, family 2, subfamily A, member 20 pseudogene /// olfactory receptor, family 2, subfamily A, member 9 pseudogene | 222290_at | 0.40 | 6.30 | ↓ | 0.56 | 7.87 |  | 0.74 | 26.48 |  |
| DENR | density-regulated protein | 238982_at | 0.40 | 7.26 | ↓ | 1.05 | 52.07 |  | 0.46 | 5.75 | ↓ |
| DIAPH3 | diaphanous homolog 3 (Drosophila) | 229097_at | 0.40 | 1.40 | ↓ | 0.72 | 17.74 |  | 0.73 | 13.22 |  |
| CDNF | cerebral dopamine neurotrophic factor | 1568696_at | 0.40 | 9.04 | ↓ | 0.65 | 31.53 |  | 1.21 | 46.35 |  |
| SEMA3F | sema domain, immunoglobulin domain (Ig), short basic domain, secreted, (semaphorin) 3F | 35666_at | 0.40 | 8.24 | ↓ | 0.64 | 33.65 |  | 0.76 | 41.22 |  |
| ZNF124 | zinc finger protein 124 | 206928_at | 0.40 | 5.70 | ↓ | 0.82 | 41.29 |  | 1.10 | 56.10 |  |
| C5orf56 | Chromosome 5 open reading frame 56 | 1560128_x_at | 0.40 | 9.04 | ↓ | 0.92 | 59.66 |  | 1.23 | 52.56 |  |
| STK38L | serine/threonine kinase 38 like | 212565_at | 0.40 | 7.26 | ↓ | 1.30 | 38.78 |  | 0.80 | 41.22 |  |
| MFAP5 | microfibrillar associated protein 5 | 209758_s_at | 0.40 | 9.04 | ↓ | 0.84 | 53.46 |  | 2.03 | 7.00 | ↑ |
| FOXM1 | forkhead box M1 | 202580_x_at | 0.40 | 6.30 | ↓ | 0.58 | 11.08 |  | 0.05 | 0.00 | ↓ |
| ADI1 | acireductone dioxygenase 1 | 222400_s_at | 0.40 | 3.90 | ↓ | 0.78 | 33.65 |  | 1.10 | 54.08 |  |
| PLK2 | polo-like kinase 2 | 201939_at | 0.40 | 3.54 | ↓ | 0.67 | 12.66 |  | 1.14 | 58.81 |  |
| MCL1 | Myeloid cell leukemia sequence 1 (BCL2-related) | 214056_at | 0.40 | 1.40 | ↓ | 0.98 | 56.87 |  | 1.54 | 11.20 |  |
| MTPN | myotrophin | 223925_s_at | 0.39 | 1.40 | ↓ | 1.43 | 41.29 |  | 1.89 | 2.70 |  |
| GOLIM4 | golgi integral membrane protein 4 | 204324_s_at | 0.39 | 6.30 | ↓ | 1.24 | 36.20 |  | 0.89 | 50.73 |  |
| MDN1 | MDN1, midasin homolog (yeast) | 1569484_s_at | 0.39 | 8.24 | ↓ | 0.88 | 52.07 |  | 0.85 | 41.22 |  |
| LHFPL2 | lipoma HMGIC fusion partner-like 2 | 212658_at | 0.39 | 4.93 | ↓ | 0.51 | 9.01 |  | 0.88 | 46.35 |  |
| DGKI | diacylglycerol kinase, iota | 206806_at | 0.39 | 9.04 | ↓ | 0.84 | 47.13 |  | 1.51 | 32.58 |  |
| PHACTR2 | phosphatase and actin regulator 2 | 204049_s_at | 0.39 | 2.70 | ↓ | 0.58 | 9.01 |  | 2.31 | 1.02 | ↑ |
| C14orf126 | chromosome 14 open reading frame 126 | 1553801_a_at | 0.39 | 5.70 | ↓ | 0.89 | 38.78 |  | 1.62 | 58.68 |  |
| TAF9B | TAF9B RNA polymerase II, TATA box binding protein (TBP)-associated factor, 31kDa | 226037_s_at | 0.39 | 7.26 | ↓ | 0.67 | 24.54 |  | 0.90 | 58.81 |  |
| UHRF1 | ubiquitin-like with PHD and ring finger domains 1 | 225655_at | 0.39 | 4.38 | ↓ | 0.80 | 33.65 |  | 0.10 | 0.00 | ↓ |
| PNN | pinin, desmosome associated protein | 1567213_at | 0.39 | 1.60 | ↓ | 1.23 | 38.78 |  | 1.26 | 43.83 |  |
| OBFC2A | oligonucleotide/oligosaccharide-binding fold containing 2A | 219334_s_at | 0.38 | 7.26 | ↓ | 0.77 | 24.54 |  | 1.26 | 38.27 |  |
| XG | Xg blood group | 1554062_at | 0.38 | 7.26 | ↓ | 1.06 | 56.87 |  | 3.18 | 13.22 |  |
| RAD51AP1 | RAD51 associated protein 1 | 204146_at | 0.38 | 1.40 | ↓ | 0.70 | 17.74 |  | 0.19 | 0.00 | ↓ |
| RRM2 | ribonucleotide reductase M2 | 201890_at | 0.38 | 4.93 | ↓ | 0.71 | 26.77 |  | 0.07 | 0.00 | ↓ |
| ZWINT | ZW10 interactor | 204026_s_at | 0.38 | 4.38 | ↓ | 0.79 | 31.53 |  | 0.34 | 0.21 | ↓ |
| SERPINE2 | Serpin peptidase inhibitor, clade E (nexin, plasminogen activator inhibitor type 1), member 2 | 227487_s_at | 0.38 | 4.38 | ↓ | 0.86 | 41.29 |  | 1.77 | 8.02 |  |
| ZFHX4 | zinc finger homeobox 4 | 241700_at | 0.38 | 1.40 | ↓ | 0.71 | 12.66 |  | 0.60 | 3.36 |  |
| ZFPM2 | zinc finger protein, multitype 2 | 219778_at | 0.38 | 2.18 | ↓ | 0.58 | 6.80 |  | 1.59 | 26.48 |  |
| C5orf44 | chromosome 5 open reading frame 44 | 236526_x_at | 0.38 | 3.90 | ↓ | 0.96 | 54.70 |  | 0.86 | 38.27 |  |
| C11orf82 | chromosome 11 open reading frame 82 | 228281_at | 0.38 | 2.46 | ↓ | 0.77 | 24.54 |  | 0.16 | 0.34 | ↓ |
| CNTLN | centlein, centrosomal protein | 239989_at | 0.38 | 4.38 | ↓ | 1.01 | 60.11 |  | 0.88 | 41.22 |  |
| SMARCA2 | SWI/SNF related, matrix associated, actin dependent regulator of chromatin, subfamily a, member 2 | 206543_at | 0.38 | 2.46 | ↓ | 1.16 | 43.50 |  | 1.42 | 15.27 |  |
| ATP6V0E1 | ATPase, H+ transporting, lysosomal 9kDa, V0 subunit e1 | 201171_at | 0.38 | 6.30 | ↓ | 1.36 | 24.54 |  | 2.03 | 4.16 | ↑ |
| HOXB3 | homeobox B3 | 228904_at | 0.38 | 6.30 | ↓ | 0.77 | 33.65 |  | 0.42 | 5.75 | ↓ |
| GREM2 | gremlin 2 | 220794_at | 0.37 | 6.30 | ↓ | 0.37 | 1.74 | ↓ | 1.36 | 35.63 |  |
| PCDHB5 | protocadherin beta 5 | 223629_at | 0.37 | 7.26 | ↓ | 1.72 | 12.66 |  | 0.35 | 1.99 | ↓ |
| DCTN4 | dynactin 4 (p62) | 233490_at | 0.37 | 6.30 | ↓ | 0.69 | 31.53 |  | 0.93 | 58.81 |  |
| TOP2A | topoisomerase (DNA) II alpha 170kDa | 201291_s_at | 0.37 | 2.70 | ↓ | 0.63 | 7.87 |  | 0.08 | 0.00 | ↓ |
| PBX1 | pre-B-cell leukemia homeobox 1 | 212148_at | 0.37 | 7.26 | ↓ | 0.39 | 6.11 | ↓ | 1.59 | 26.48 |  |
| IPO9 | importin 9 | 244703_x_at | 0.37 | 5.70 | ↓ | 0.58 | 7.87 |  | 0.93 | 50.73 |  |
| ZNF326 | zinc finger protein 326 | 227680_at | 0.37 | 1.40 | ↓ | 1.12 | 43.50 |  | 0.89 | 35.63 |  |
| DENR | density-regulated protein | 234347_s_at | 0.37 | 3.54 | ↓ | 1.21 | 36.20 |  | 1.60 | 35.63 |  |
| C9orf102 | chromosome 9 open reading frame 102 | 232013_at | 0.37 | 2.70 | ↓ | 0.79 | 24.54 |  | 0.93 | 38.27 |  |
| CENPK | centromere protein K | 222848_at | 0.36 | 1.20 | ↓ | 0.66 | 11.08 |  | 0.21 | 0.00 | ↓ |
| PGK1 | Phosphoglycerate kinase 1 | 217383_at | 0.36 | 2.46 | ↓ | 0.85 | 45.34 |  | 0.99 | 58.81 |  |
| EMX2 | empty spiracles homeobox 2 | 221950_at | 0.36 | 4.38 | ↓ | 0.90 | 53.46 |  | 0.55 | 11.20 |  |
| GLIPR1 | GLI pathogenesis-related 1 | 204221_x_at | 0.36 | 3.90 | ↓ | 0.83 | 26.77 |  | 0.60 | 8.02 |  |
| FANCI | Fanconi anemia, complementation group I | 213007_at | 0.36 | 4.38 | ↓ | 0.86 | 33.65 |  | 0.37 | 0.00 | ↓ |
| HAPLN1 | hyaluronan and proteoglycan link protein 1 | 230895_at | 0.36 | 5.70 | ↓ | 1.04 | 36.20 |  | 1.16 | 55.52 |  |
| NLGN1 | neuroligin 1 | 205893_at | 0.36 | 7.26 | ↓ | 1.38 | 49.04 |  | 1.13 | 46.35 |  |
| NCAPG | non-SMC condensin I complex, subunit G | 218662_s_at | 0.36 | 1.40 | ↓ | 0.97 | 49.04 |  | 0.18 | 0.00 | ↓ |
| HNRNPUL2 | heterogeneous nuclear ribonucleoprotein U-like 2 | 66053_at | 0.35 | 1.90 | ↓ | 1.86 | 3.35 |  | 0.74 | 11.20 |  |
| C3orf14 | chromosome 3 open reading frame 14 | 219288_at | 0.35 | 0.00 | ↓ | 1.05 | 53.46 |  | 0.67 | 5.75 |  |
| MIPOL1 | mirror-image polydactyly 1 | 244246_at | 0.35 | 3.90 | ↓ | 0.79 | 36.20 |  | 0.75 | 20.38 |  |
| CHAC2 | ChaC, cation transport regulator homolog 2 (E. coli) | 235117_at | 0.35 | 6.30 | ↓ | 1.02 | 54.70 |  | 0.65 | 23.22 |  |
| ZNF367 | zinc finger protein 367 | 229551_x_at | 0.35 | 2.18 | ↓ | 0.67 | 19.82 |  | 0.16 | 0.00 | ↓ |
| NOX4 | NADPH oxidase 4 | 219773_at | 0.35 | 4.38 | ↓ | 0.80 | 33.65 |  | 1.24 | 48.62 |  |
| PHLDA2 | pleckstrin homology-like domain, family A, member 2 | 209803_s_at | 0.35 | 2.46 | ↓ | 1.17 | 58.42 |  | 1.08 | 57.64 |  |
| POLE2 | polymerase (DNA directed), epsilon 2 (p59 subunit) | 205909_at | 0.35 | 2.18 | ↓ | 0.91 | 38.78 |  | 0.37 | 0.00 | ↓ |
| FARSB | phenylalanyl-tRNA synthetase, beta subunit | 232063_x_at | 0.34 | 8.24 | ↓ | 0.98 | 53.46 |  | 0.64 | 15.27 |  |
| PENK | proenkephalin | 213791_at | 0.34 | 5.70 | ↓ | 1.14 | 56.87 |  | 0.83 | 46.35 |  |
| P2RY6 | pyrimidinergic receptor P2Y, G-protein coupled, 6 | 208373_s_at | 0.34 | 2.18 | ↓ | 0.55 | 7.87 |  | 1.60 | 23.22 |  |
| ALDH1A3 | aldehyde dehydrogenase 1 family, member A3 | 203180_at | 0.34 | 1.40 | ↓ | 0.72 | 17.74 |  | 2.27 | 20.38 |  |
| PKIB | protein kinase (cAMP-dependent, catalytic) inhibitor beta | 223551_at | 0.34 | 9.04 | ↓ | 1.10 | 60.64 |  | 9.54 | 13.22 |  |
| RAD50 | RAD50 homolog (S. cerevisiae) | 209349_at | 0.34 | 4.38 | ↓ | 1.04 | 50.64 |  | 0.81 | 35.63 |  |
| STK38L | serine/threonine kinase 38 like | 212572_at | 0.34 | 4.38 | ↓ | 1.08 | 49.04 |  | 0.92 | 54.08 |  |
| TEK | TEK tyrosine kinase, endothelial | 206702_at | 0.34 | 9.04 | ↓ | 0.79 | 33.65 |  | 1.89 | 17.70 |  |
| PAIP1 | poly(A) binding protein interacting protein 1 | 209064_x_at | 0.34 | 1.20 | ↓ | 1.28 | 49.04 |  | 1.19 | 54.08 |  |
| CBL | Cas-Br-M (murine) ecotropic retroviral transforming sequence | 229010_at | 0.33 | 4.93 | ↓ | 0.46 | 6.80 | ↓ | 0.63 | 23.22 |  |
| KIAA1267 /// LOC100294337 | KIAA1267 /// hypothetical LOC100294337 | 243589_at | 0.33 | 8.24 | ↓ | 0.57 | 29.01 |  | 0.85 | 54.08 |  |
| MED13L | Mediator complex subunit 13-like | 216109_at | 0.33 | 4.38 | ↓ | 0.44 | 4.45 | ↓ | 0.50 | 8.02 | ↓ |
| NFIA | nuclear factor I/A | 226806_s_at | 0.33 | 6.30 | ↓ | 0.62 | 29.01 |  | 1.03 | 56.65 |  |
| ARMC9 | armadillo repeat containing 9 | 219637_at | 0.32 | 4.93 | ↓ | 0.49 | 12.66 |  | 3.12 | 1.99 | ↑ |
| HELLS | helicase, lymphoid-specific | 227350_at | 0.32 | 4.93 | ↓ | 0.96 | 36.20 |  | 0.28 | 0.00 | ↓ |
| NUDCD2 | NudC domain containing 2 | 226643_s_at | 0.32 | 1.09 | ↓ | 1.35 | 17.74 |  | 1.42 | 17.70 |  |
| OPCML | opioid binding protein/cell adhesion molecule-like | 214111_at | 0.32 | 7.26 | ↓ | 0.93 | 52.07 |  | 0.49 | 11.20 |  |
| TMEM200A | transmembrane protein 200A | 234994_at | 0.32 | 9.04 | ↓ | 2.26 | 17.74 |  | 3.13 | 1.99 | ↑ |
| MYO10 | myosin X | 216222_s_at | 0.32 | 6.30 | ↓ | 2.83 | 4.45 | ↑ | 1.01 | 57.92 |  |
| LRRC17 | leucine rich repeat containing 17 | 205381_at | 0.32 | 1.09 | ↓ | 1.73 | 47.13 |  | 0.75 | 17.70 |  |
| TUG1 | taurine upregulated 1 (non-protein coding) | 228397_at | 0.31 | 1.20 | ↓ | 1.26 | 31.53 |  | 0.61 | 9.54 |  |
| LOC100507248 | hypothetical LOC100507248 | 235236_at | 0.31 | 2.46 | ↓ | 1.02 | 52.07 |  | 1.13 | 54.08 |  |
| NCKAP1 | NCK-associated protein 1 | 217465_at | 0.31 | 1.20 | ↓ | 0.34 | 0.00 | ↓ | 2.08 | 1.99 | ↑ |
| KY | kyphoscoliosis peptidase | 244572_at | 0.31 | 1.09 | ↓ | 0.54 | 6.11 |  | 1.10 | 56.65 |  |
| PAIP1 | poly(A) binding protein interacting protein 1 | 208051_s_at | 0.31 | 2.18 | ↓ | 1.15 | 56.87 |  | 1.18 | 50.73 |  |
| SH3BP2 | SH3-domain binding protein 2 | 217257_at | 0.31 | 4.38 | ↓ | 0.77 | 17.74 |  | 1.05 | 46.35 |  |
| LDB2 | LIM domain binding 2 | 206481_s_at | 0.31 | 4.38 | ↓ | 0.36 | 3.35 | ↓ | 1.37 | 41.22 |  |
| SOBP | sine oculis binding protein homolog (Drosophila) | 218974_at | 0.30 | 4.93 | ↓ | 0.87 | 54.70 |  | 0.70 | 32.58 |  |
| ING3 | inhibitor of growth family, member 3 | 231863_at | 0.30 | 1.33 | ↓ | 0.79 | 36.20 |  | 1.32 | 41.22 |  |
| RRN3P3 | RNA polymerase I transcription factor homolog (S. cerevisiae) pseudogene 3 | 238412_at | 0.30 | 4.38 | ↓ | 1.05 | 45.34 |  | 1.06 | 58.68 |  |
| ADAM12 | ADAM metallopeptidase domain 12 | 213790_at | 0.30 | 3.90 | ↓ | 1.24 | 29.01 |  | 0.66 | 29.62 |  |
| CMPK2 | cytidine monophosphate (UMP-CMP) kinase 2, mitochondrial | 226702_at | 0.29 | 9.04 | ↓ | 0.32 | 6.11 | ↓ | 0.18 | 3.36 | ↓ |
| DIAPH3 | diaphanous homolog 3 (Drosophila) | 232596_at | 0.28 | 2.46 | ↓ | 0.30 | 0.36 | ↓ | 0.56 | 7.00 |  |
| CELF2 | CUGBP, Elav-like family member 2 | 202158_s_at | 0.28 | 4.38 | ↓ | 0.75 | 50.64 |  | 4.28 | 3.36 | ↑ |
| MFAP5 | microfibrillar associated protein 5 | 213764_s_at | 0.28 | 9.04 | ↓ | 1.30 | 41.29 |  | 3.02 | 2.70 | ↑ |
| KCNAB1 | potassium voltage-gated channel, shaker-related subfamily, beta member 1 | 210078_s_at | 0.28 | 4.93 | ↓ | 0.38 | 5.22 | ↓ | 1.30 | 52.56 |  |
| SUZ12 | Suppressor of zeste 12 homolog (Drosophila) | 1566191_at | 0.28 | 5.70 | ↓ | 0.96 | 53.46 |  | 0.42 | 13.22 |  |
| RGS4 | regulator of G-protein signaling 4 | 204337_at | 0.28 | 5.70 | ↓ | 1.09 | 49.04 |  | 2.76 | 2.70 | ↑ |
| NLGN1 | neuroligin 1 | 231361_at | 0.28 | 6.30 | ↓ | 1.38 | 38.78 |  | 1.11 | 48.62 |  |
| GULP1 | GULP, engulfment adaptor PTB domain containing 1 | 204237_at | 0.27 | 4.38 | ↓ | 0.84 | 47.13 |  | 1.57 | 17.70 |  |
| CARD6 | caspase recruitment domain family, member 6 | 224414_s_at | 0.27 | 1.20 | ↓ | 0.93 | 43.50 |  | 1.77 | 2.70 |  |
| FAM105B | family with sequence similarity 105, member B | 240834_at | 0.27 | 2.70 | ↓ | 0.81 | 41.29 |  | 0.60 | 9.54 |  |
| SAMD9L | sterile alpha motif domain containing 9-like | 235643_at | 0.27 | 4.93 | ↓ | 0.77 | 52.07 |  | 0.71 | 38.27 |  |
| DDX17 | DEAD (Asp-Glu-Ala-Asp) box polypeptide 17 | 213998_s_at | 0.27 | 4.93 | ↓ | 0.36 | 7.87 | ↓ | 0.34 | 5.75 | ↓ |
| SLC24A3 | solute carrier family 24 (sodium/potassium/calcium exchanger), member 3 | 57588_at | 0.26 | 9.04 | ↓ | 1.26 | 43.50 |  | 0.34 | 11.20 |  |
| RAB2A | RAB2A, member RAS oncogene family | 221960_s_at | 0.26 | 0.00 | ↓ | 1.03 | 60.11 |  | 1.41 | 23.22 |  |
| EMB | embigin | 226789_at | 0.26 | 4.38 | ↓ | 0.54 | 14.26 |  | 0.59 | 20.38 |  |
| ARHGAP29 | Rho GTPase activating protein 29 | 203910_at | 0.26 | 4.38 | ↓ | 0.49 | 14.26 |  | 2.39 | 3.36 | ↑ |
| MFAP5 | microfibrillar associated protein 5 | 213765_at | 0.26 | 4.93 | ↓ | 1.01 | 59.12 |  | 3.32 | 1.43 | ↑ |
| ARMC8 | armadillo repeat containing 8 | 236966_at | 0.25 | 4.38 | ↓ | 0.89 | 53.46 |  | 0.71 | 43.83 |  |
| RGS4 | regulator of G-protein signaling 4 | 204339_s_at | 0.25 | 5.70 | ↓ | 0.70 | 15.95 |  | 3.02 | 4.84 | ↑ |
| GTPBP8 | GTP-binding protein 8 (putative) | 242685_at | 0.25 | 1.40 | ↓ | 1.10 | 49.04 |  | 1.30 | 32.58 |  |
| LNX1 | ligand of numb-protein X 1 | 223611_s_at | 0.25 | 5.70 | ↓ | 0.84 | 58.42 |  | 1.28 | 50.73 |  |
| KCNAB1 | potassium voltage-gated channel, shaker-related subfamily, beta member 1 | 210471_s_at | 0.25 | 4.38 | ↓ | 0.33 | 3.81 | ↓ | 1.17 | 58.81 |  |
| SIGLEC15 | sialic acid binding Ig-like lectin 15 | 215856_at | 0.25 | 4.38 | ↓ | 0.51 | 17.74 |  | 1.13 | 56.10 |  |
| FLJ36848 | hypothetical LOC647115 | 231698_at | 0.25 | 3.21 | ↓ | 0.81 | 41.29 |  | 0.95 | 54.08 |  |
| SLC24A3 | solute carrier family 24 (sodium/potassium/calcium exchanger), member 3 | 219090_at | 0.25 | 9.04 | ↓ | 1.04 | 53.46 |  | 0.31 | 8.02 | ↓ |
| EPHA3 | EPH receptor A3 | 206070_s_at | 0.25 | 7.26 | ↓ | 1.53 | 14.26 |  | 0.57 | 9.54 |  |
| NEAT1 | nuclear paraspeckle assembly transcript 1 (non-protein coding) | 214657_s_at | 0.24 | 1.60 | ↓ | 0.41 | 6.11 | ↓ | 1.48 | 23.22 |  |
| ANKRD12 | Ankyrin repeat domain 12 | 216563_at | 0.24 | 1.90 | ↓ | 4.40 | 0.29 | ↑ | 1.53 | 13.22 |  |
| MEOX2 | mesenchyme homeobox 2 | 206201_s_at | 0.24 | 0.00 | ↓ | 1.13 | 50.64 |  | 0.17 | 0.00 | ↓ |
| LOC100190939 | hypothetical LOC100190939 | 226407_at | 0.24 | 1.40 | ↓ | 0.77 | 24.54 |  | 0.95 | 46.35 |  |
| LOC643792 | contactin associated protein-like 3 pseudogene | 244065_at | 0.24 | 2.18 | ↓ | 0.77 | 26.77 |  | 0.34 | 2.43 | ↓ |
| HELLS | helicase, lymphoid-specific | 220085_at | 0.23 | 1.40 | ↓ | 0.71 | 15.95 |  | 0.19 | 0.50 | ↓ |
| CHI3L1 | chitinase 3-like 1 (cartilage glycoprotein-39) | 209396_s_at | 0.23 | 4.93 | ↓ | 0.09 | 0.36 | ↓ | 1.44 | 57.92 |  |
| CHI3L1 | chitinase 3-like 1 (cartilage glycoprotein-39) | 209395_at | 0.22 | 4.38 | ↓ | 0.15 | 0.70 | ↓ | 1.19 | 54.08 |  |
| DHX29 | DEAH (Asp-Glu-Ala-His) box polypeptide 29 | 212649_at | 0.22 | 0.00 | ↓ | 0.98 | 54.70 |  | 1.58 | 2.43 |  |
| AKR1C1 | Aldo-keto reductase family 1, member C1 (dihydrodiol dehydrogenase 1; 20-alpha (3-alpha)-hydroxysteroid dehydrogenase) | 1562102_at | 0.21 | 4.38 | ↓ | 0.13 | 0.70 | ↓ | 0.86 | 54.08 |  |
| COL14A1 | collagen, type XIV, alpha 1 | 212865_s_at | 0.21 | 7.26 | ↓ | 2.41 | 17.74 |  | 0.25 | 9.54 | ↓ |
| KRT18 | keratin 18 | 201596_x_at | 0.21 | 4.38 | ↓ | 1.67 | 52.07 |  | 2.21 | 5.75 | ↑ |
| POM121L9P | POM121 membrane glycoprotein-like 9, pseudogene | 222253_s_at | 0.20 | 7.26 | ↓ | 0.30 | 12.66 |  | 0.55 | 46.35 |  |
| FGD4 | FYVE, RhoGEF and PH domain containing 4 | 227948_at | 0.20 | 5.70 | ↓ | 0.37 | 17.74 |  | 2.22 | 8.02 | ↑ |
| RGS4 | regulator of G-protein signaling 4 | 204338_s_at | 0.20 | 4.38 | ↓ | 0.23 | 1.45 | ↓ | 2.47 | 8.02 | ↑ |
| METTL9 | Methyltransferase like 9 | 226220_at | 0.19 | 1.09 | ↓ | 0.97 | 54.70 |  | 1.39 | 26.48 |  |
| CELF2 | CUGBP, Elav-like family member 2 | 202157_s_at | 0.19 | 3.21 | ↓ | 0.41 | 12.66 |  | 4.23 | 3.36 | ↑ |
| F2RL1 | coagulation factor II (thrombin) receptor-like 1 | 213506_at | 0.18 | 2.18 | ↓ | 1.39 | 41.29 |  | 0.10 | 0.34 | ↓ |
| TWF1 | Twinfilin, actin-binding protein, homolog 1 (Drosophila) | 214008_at | 0.17 | 0.00 | ↓ | 0.28 | 1.26 | ↓ | 1.50 | 23.22 |  |
| ALG5 | asparagine-linked glycosylation 5, dolichyl-phosphate beta-glucosyltransferase homolog (S. cerevisiae) | 222556_at | 0.16 | 1.20 | ↓ | 1.09 | 54.70 |  | 1.49 | 13.22 |  |
| LOC100506941 | hypothetical LOC100506941 | 231559_at | 0.15 | 0.00 | ↓ | 0.86 | 47.13 |  | 1.61 | 38.27 |  |
| FAM76B | family with sequence similarity 76, member B | 1553749_at | 0.14 | 1.09 | ↓ | 1.23 | 45.34 |  | 2.05 | 9.54 | ↑ |
| CNTN3 | contactin 3 (plasmacytoma associated) | 229831_at | 0.13 | 1.60 | ↓ | 0.39 | 11.08 |  | 6.13 | 1.43 | ↑ |
| ZIC1 | Zic family member 1 (odd-paired homolog, Drosophila) | 206373_at | 0.13 | 4.38 | ↓ | 1.77 | 14.26 |  | 1.85 | 55.52 |  |
| MALL | mal, T-cell differentiation protein-like | 209373_at | 0.13 | 4.38 | ↓ | 0.32 | 17.74 |  | 0.53 | 52.56 |  |
| HSP90B1 | heat shock protein 90kDa beta (Grp94), member 1 | 216450_x_at | 0.12 | 0.00 | ↓ | 1.42 | 19.82 |  | 1.18 | 46.35 |  |
| PKIB | protein kinase (cAMP-dependent, catalytic) inhibitor beta | 231120_x_at | 0.12 | 1.33 | ↓ | 1.51 | 41.29 |  | 11.59 | 38.27 |  |
| SCN3A | sodium channel, voltage-gated, type III, alpha subunit | 210432_s_at | 0.10 | 9.04 | ↓ | 0.31 | 22.17 |  | 5.35 | 4.84 | ↑ |
| KAL1 | Kallmann syndrome 1 sequence | 205206_at | 0.10 | 9.04 | ↓ | 0.50 | 50.64 |  | 0.05 | 1.61 | ↓ |
| COL5A3 | collagen, type V, alpha 3 | 218975_at | 3.14 | 14.24 |  | 1.31 | 31.53 |  | 0.22 | 0.43 | ↓ |
| TSPAN18 | tetraspanin 18 | 227307_at | 3.04 | 10.25 |  | 1.54 | 50.64 |  | 0.36 | 2.43 | ↓ |
| PRLH | prolactin releasing hormone | 221443_x_at | 3.04 | 17.32 |  | 0.47 | 3.35 | ↓ | 1.61 | 5.75 |  |
| CPE | carboxypeptidase E | 201116_s_at | 2.88 | 15.82 |  | 1.08 | 45.34 |  | 4.07 | 3.36 | ↑ |
| --- | --- | 208849_at | 2.85 | 40.42 |  | 1.37 | 26.77 |  | 2.21 | 1.61 | ↑ |
| COMP | cartilage oligomeric matrix protein | 205713_s_at | 2.65 | 11.75 |  | 5.35 | 6.11 | ↑ | 3.44 | 41.22 |  |
| TMEM206 | transmembrane protein 206 | 218814_s_at | 2.60 | 15.82 |  | 0.39 | 1.74 | ↓ | 1.27 | 32.58 |  |
| --- | --- | 217544_at | 2.58 | 24.35 |  | 2.43 | 1.45 | ↑ | 1.35 | 48.62 |  |
| SPAG4 | sperm associated antigen 4 | 219888_at | 2.39 | 17.32 |  | 1.45 | 50.64 |  | 0.33 | 7.00 | ↓ |
| NR4A1 | nuclear receptor subfamily 4, group A, member 1 | 202340_x_at | 2.38 | 11.75 |  | 1.59 | 36.20 |  | 0.33 | 1.25 | ↓ |
| ELN | elastin | 216269_s_at | 2.38 | 11.75 |  | 0.27 | 0.40 | ↓ | 2.46 | 1.61 | ↑ |
| ZNF853 | zinc finger protein 853 | 232884_s_at | 2.37 | 34.35 |  | 0.71 | 53.46 |  | 0.13 | 2.43 | ↓ |
| LOC284998 | hypothetical LOC284998 | 239879_at | 2.35 | 22.56 |  | 0.49 | 6.80 | ↓ | 0.77 | 23.22 |  |
| SPP1 | secreted phosphoprotein 1 | 209875_s_at | 2.33 | 15.82 |  | 0.92 | 43.50 |  | 0.47 | 7.00 | ↓ |
| TUBB2B | tubulin, beta 2B | 214023_x_at | 2.33 | 18.98 |  | 1.78 | 26.77 |  | 0.37 | 3.36 | ↓ |
| FAM70A | family with sequence similarity 70, member A | 219895_at | 2.32 | 17.32 |  | 1.14 | 54.70 |  | 0.07 | 0.21 | ↓ |
| ELN | elastin | 212670_at | 2.29 | 11.75 |  | 0.25 | 3.81 | ↓ | 1.93 | 13.22 |  |
| LCTL | lactase-like | 1563899_at | 2.28 | 11.75 |  | 1.34 | 43.50 |  | 0.48 | 7.00 | ↓ |
| BCL2L11 | BCL2-like 11 (apoptosis facilitator) | 1558143_a_at | 2.23 | 10.25 |  | 0.77 | 31.53 |  | 0.34 | 3.36 | ↓ |
| SLC5A12 | solute carrier family 5 (sodium/glucose cotransporter), member 12 | 231424_at | 2.22 | 10.25 |  | 0.69 | 12.66 |  | 0.27 | 2.70 | ↓ |
| USP9X | ubiquitin specific peptidase 9, X-linked | 230543_at | 2.19 | 12.80 |  | 0.18 | 0.18 | ↓ | 1.92 | 7.00 |  |
| MIAT | myocardial infarction associated transcript (non-protein coding) | 237322_at | 2.17 | 17.32 |  | 0.43 | 2.30 | ↓ | 0.65 | 17.70 |  |
| PHLDA1 | pleckstrin homology-like domain, family A, member 1 | 218000_s_at | 2.12 | 17.32 |  | 0.20 | 0.60 | ↓ | 1.03 | 58.81 |  |
| PNMA2 | paraneoplastic antigen MA2 | 209598_at | 2.09 | 10.25 |  | 1.80 | 17.74 |  | 2.56 | 8.02 | ↑ |
| ZNF467 | zinc finger protein 467 | 214746_s_at | 2.08 | 57.71 |  | 0.39 | 0.98 | ↓ | 1.05 | 57.64 |  |
| COL5A3 | collagen, type V, alpha 3 | 52255_s_at | 2.03 | 24.35 |  | 1.68 | 12.66 |  | 0.43 | 1.02 | ↓ |
| LOC100130522 | hypothetical LOC100130522 | 1557590_at | 2.03 | 22.56 |  | 0.50 | 9.01 | ↓ | 0.89 | 54.08 |  |
| PEG3 | paternally expressed 3 | 209243_s_at | 2.00 | 53.22 |  | 1.45 | 19.82 |  | 0.34 | 3.36 | ↓ |
| HSPG2 | heparan sulfate proteoglycan 2 | 201654_s_at | 2.00 | 4.93 |  | 0.26 | 5.22 | ↓ | 0.64 | 50.73 |  |
| WIZ | widely interspaced zinc finger motifs | 221785_at | 1.99 | 4.93 |  | 0.25 | 0.98 | ↓ | 0.98 | 56.65 |  |
| SEC16B | SEC16 homolog B (S. cerevisiae) | 1564423_a_at | 1.99 | 30.22 |  | 0.31 | 0.53 | ↓ | 0.92 | 41.22 |  |
| FRMPD4 | FERM and PDZ domain containing 4 | 239290_at | 1.98 | 5.70 |  | 1.63 | 33.65 |  | 4.44 | 0.00 | ↑ |
| PVR | poliovirus receptor | 32699_s_at | 1.98 | 2.46 |  | 0.41 | 1.26 | ↓ | 1.34 | 41.22 |  |
| FBXO44 | F-box protein 44 | 223517_at | 1.97 | 3.90 |  | 0.49 | 5.22 | ↓ | 1.45 | 26.48 |  |
| ADAMTS5 | ADAM metallopeptidase with thrombospondin type 1 motif, 5 | 1558636_s_at | 1.97 | 11.75 |  | 0.72 | 41.29 |  | 0.34 | 1.99 | ↓ |
| B4GALT1 | UDP-Gal:betaGlcNAc beta 1,4- galactosyltransferase, polypeptide 1 | 216627_s_at | 1.97 | 20.86 |  | 0.48 | 10.02 |  | 2.30 | 4.84 | ↑ |
| DMPK | dystrophia myotonica-protein kinase | 37996_s_at | 1.97 | 1.40 |  | 0.42 | 0.53 | ↓ | 1.87 | 8.02 |  |
| RAD23A | RAD23 homolog A (S. cerevisiae) | 201039_s_at | 1.97 | 1.33 |  | 0.45 | 0.53 | ↓ | 0.99 | 54.08 |  |
| B4GALT1 | UDP-Gal:betaGlcNAc beta 1,4- galactosyltransferase, polypeptide 1 | 211631_x_at | 1.97 | 11.75 |  | 0.57 | 12.66 |  | 2.47 | 3.36 | ↑ |
| MAFG | v-maf musculoaponeurotic fibrosarcoma oncogene homolog G (avian) | 204970_s_at | 1.97 | 2.46 |  | 3.57 | 0.40 | ↑ | 1.03 | 55.52 |  |
| PVRL2 | poliovirus receptor-related 2 (herpesvirus entry mediator B) | 232078_at | 1.96 | 10.25 |  | 0.22 | 0.70 | ↓ | 0.80 | 48.62 |  |
| C9orf25 | chromosome 9 open reading frame 25 | 225146_at | 1.96 | 11.75 |  | 0.91 | 60.31 |  | 0.47 | 9.54 | ↓ |
| PEG10 | paternally expressed 10 | 212092_at | 1.95 | 7.26 |  | 0.33 | 1.07 | ↓ | 1.16 | 58.28 |  |
| CD24 | CD24 molecule | 216379_x_at | 1.95 | 8.24 |  | 1.55 | 60.11 |  | 0.31 | 1.99 | ↓ |
| RCAN1 | regulator of calcineurin 1 | 215253_s_at | 1.95 | 6.30 |  | 0.25 | 0.18 | ↓ | 1.17 | 55.52 |  |
| ARFGAP1 | ADP-ribosylation factor GTPase activating protein 1 | 234001_s_at | 1.94 | 1.90 |  | 0.48 | 0.45 | ↓ | 0.90 | 38.27 |  |
| NDUFA4L2 | NADH dehydrogenase (ubiquinone) 1 alpha subcomplex, 4-like 2 | 218484_at | 1.94 | 20.86 |  | 1.07 | 54.70 |  | 0.28 | 8.02 | ↓ |
| CES1 | carboxylesterase 1 | 209616_s_at | 1.94 | 5.70 |  | 3.47 | 5.22 | ↑ | 0.26 | 4.16 | ↓ |
| SBNO2 | strawberry notch homolog 2 (Drosophila) | 204166_at | 1.93 | 2.46 |  | 0.27 | 0.18 | ↓ | 0.81 | 29.62 |  |
| MMP14 | matrix metallopeptidase 14 (membrane-inserted) | 202828_s_at | 1.93 | 8.24 |  | 2.20 | 2.80 | ↑ | 1.12 | 58.81 |  |
| ZSCAN18 | zinc finger and SCAN domain containing 18 | 217593_at | 1.92 | 11.75 |  | 0.71 | 45.34 |  | 0.28 | 4.16 | ↓ |
| PRKCSH | protein kinase C substrate 80K-H | 200707_at | 1.92 | 2.46 |  | 0.14 | 0.00 | ↓ | 0.70 | 8.02 |  |
| UQCRC2 | ubiquinol-cytochrome c reductase core protein II | 241755_at | 1.92 | 14.24 |  | 2.15 | 5.22 | ↑ | 1.24 | 50.73 |  |
| PIP5K1A | phosphatidylinositol-4-phosphate 5-kinase, type I, alpha | 211205_x_at | 1.92 | 11.75 |  | 0.34 | 5.22 | ↓ | 2.37 | 8.02 | ↑ |
| CHD7 | chromodomain helicase DNA binding protein 7 | 226123_at | 1.92 | 3.21 |  | 0.67 | 12.66 |  | 0.46 | 4.16 | ↓ |
| FOXK2 | forkhead box K2 | 242938_s_at | 1.92 | 7.26 |  | 0.36 | 0.84 | ↓ | 0.85 | 41.22 |  |
| CUL7 | cullin 7 | 203558_at | 1.92 | 3.21 |  | 0.47 | 1.98 | ↓ | 0.70 | 20.38 |  |
| PCDHGA1 /// PCDHGA10 /// PCDHGA11 /// PCDHGA12 /// PCDHGA2 /// PCDHGA3 /// PCDHGA4 /// PCDHGA5 /// PCDHGA6 /// PCDHGA7 /// PCDHGA8 /// PCDHGA9 /// PCDHGB1 /// PCDHGB2 /// PCDHGB3 /// PCDHGB4 /// PCDHGB5 /// PCDHGB6 /// PCDHGB7 /// PCDHGC3 /// PCDHGC4 /// PCDHGC5 | protocadherin gamma subfamily A, 1 /// protocadherin gamma subfamily A, 10 /// protocadherin gamma subfamily A, 11 /// protocadherin gamma subfamily A, 12 /// protocadherin gamma subfamily A, 2 /// protocadherin gamma subfamily A, 3 /// protocadherin gamma subfamily A, 4 /// protocadherin gamma subfamily A, 5 /// protocadherin gamma subfamily A, 6 /// protocadherin gamma subfamily A, 7 /// protocadherin gamma subfamily A, 8 /// protocadherin gamma subfamily A, 9 /// protocadherin gamma subfamily B, 1 /// protocadherin gamma subfamily B, 2 /// protocadherin gamma subfamily B, 3 /// protocadherin gamma subfamily B, 4 /// protocadherin gamma subfamily B, 5 /// protocadherin gamma subfamily B, 6 /// protocadherin gamma subfamily B, 7 /// protocadherin gamma subfamily C, 3 /// protocadherin gamma subfamily C, 4 /// protocadherin gamma subfamily C, 5 | 205717_x_at | 1.91 | 3.21 |  | 0.32 | 0.53 | ↓ | 1.08 | 54.08 |  |
| FOSL1 | FOS-like antigen 1 | 204420_at | 1.91 | 2.46 |  | 0.45 | 0.60 | ↓ | 1.34 | 11.20 |  |
| PATZ1 | POZ (BTB) and AT hook containing zinc finger 1 | 209431_s_at | 1.91 | 3.54 |  | 1.06 | 52.07 |  | 0.49 | 4.16 | ↓ |
| NUMA1 | nuclear mitotic apparatus protein 1 | 214251_s_at | 1.91 | 4.93 |  | 0.18 | 0.18 | ↓ | 0.86 | 52.56 |  |
| NUCB1 | nucleobindin 1 | 200646_s_at | 1.91 | 1.60 |  | 0.15 | 0.00 | ↓ | 1.06 | 56.65 |  |
| PLXND1 | plexin D1 | 1563657_at | 1.91 | 34.35 |  | 0.48 | 2.80 | ↓ | 1.32 | 26.48 |  |
| ADAM15 | ADAM metallopeptidase domain 15 | 217007_s_at | 1.90 | 2.18 |  | 0.43 | 0.98 | ↓ | 0.86 | 38.27 |  |
| PCDHGA10 /// PCDHGA11 /// PCDHGA12 /// PCDHGA3 /// PCDHGA5 /// PCDHGA6 | protocadherin gamma subfamily A, 10 /// protocadherin gamma subfamily A, 11 /// protocadherin gamma subfamily A, 12 /// protocadherin gamma subfamily A, 3 /// protocadherin gamma subfamily A, 5 /// protocadherin gamma subfamily A, 6 | 211876_x_at | 1.90 | 6.30 |  | 0.06 | 0.00 | ↓ | 1.16 | 41.22 |  |
| COL1A1 | collagen, type I, alpha 1 | 217430_x_at | 1.89 | 1.60 |  | 0.09 | 0.00 | ↓ | 1.30 | 32.58 |  |
| HHLA3 | HERV-H LTR-associating 3 | 234665_x_at | 1.89 | 2.46 |  | 0.46 | 1.26 | ↓ | 0.75 | 17.70 |  |
| SLC29A1 | solute carrier family 29 (nucleoside transporters), member 1 | 201801_s_at | 1.89 | 8.24 |  | 0.33 | 1.98 | ↓ | 0.43 | 5.75 | ↓ |
| TEX9 | testis expressed 9 | 243198_at | 1.89 | 17.32 |  | 1.71 | 17.74 |  | 6.16 | 0.72 | ↑ |
| FKBP1A | FK506 binding protein 1A, 12kDa | 210186_s_at | 1.89 | 4.93 |  | 0.41 | 1.45 | ↓ | 1.43 | 17.70 |  |
| ZNF785 | zinc finger protein 785 | 1554769_at | 1.88 | 14.24 |  | 2.79 | 0.36 | ↑ | 0.76 | 17.70 |  |
| ITGA7 | integrin, alpha 7 | 209663_s_at | 1.88 | 6.30 |  | 0.30 | 1.07 | ↓ | 1.24 | 50.73 |  |
| RUNX2 | runt-related transcription factor 2 | 216994_s_at | 1.88 | 15.82 |  | 0.16 | 0.53 | ↓ | 0.93 | 58.51 |  |
| BCL2L1 | BCL2-like 1 | 215037_s_at | 1.88 | 4.93 |  | 0.33 | 0.40 | ↓ | 1.72 | 8.02 |  |
| ZAK | sterile alpha motif and leucine zipper containing kinase AZK | 225662_at | 1.88 | 1.40 |  | 2.34 | 0.84 | ↑ | 1.53 | 1.61 |  |
| PDGFA | platelet-derived growth factor alpha polypeptide | 205463_s_at | 1.87 | 3.90 |  | 2.53 | 0.60 | ↑ | 0.53 | 3.36 |  |
| TLE2 | transducin-like enhancer of split 2 (E(sp1) homolog, Drosophila) | 40837_at | 1.87 | 9.04 |  | 0.83 | 49.04 |  | 0.40 | 4.84 | ↓ |
| PDLIM7 | PDZ and LIM domain 7 (enigma) | 203370_s_at | 1.87 | 5.70 |  | 0.20 | 0.45 | ↓ | 0.72 | 43.83 |  |
| LIF | leukemia inhibitory factor (cholinergic differentiation factor) | 205266_at | 1.87 | 18.98 |  | 0.82 | 45.34 |  | 0.48 | 7.00 | ↓ |
| ALPL | alkaline phosphatase, liver/bone/kidney | 215783_s_at | 1.87 | 34.35 |  | 0.69 | 50.64 |  | 0.36 | 8.02 | ↓ |
| LRRC23 | leucine rich repeat containing 23 | 206076_at | 1.87 | 28.36 |  | 1.15 | 41.29 |  | 0.43 | 0.43 | ↓ |
| GLT25D1 | glycosyltransferase 25 domain containing 1 | 222644_s_at | 1.86 | 4.38 |  | 0.48 | 0.76 | ↓ | 0.76 | 13.22 |  |
| LASS6 | LAG1 homolog, ceramide synthase 6 | 242019_at | 1.86 | 14.24 |  | 0.68 | 12.66 |  | 2.34 | 4.84 | ↑ |
| PCOLCE | procollagen C-endopeptidase enhancer | 202465_at | 1.86 | 3.90 |  | 1.07 | 55.85 |  | 0.30 | 0.00 | ↓ |
| CDK16 | cyclin-dependent kinase 16 | 208824_x_at | 1.86 | 3.21 |  | 0.37 | 0.53 | ↓ | 0.64 | 9.54 |  |
| DCAF15 | DDB1 and CUL4 associated factor 15 | 221849_s_at | 1.86 | 4.38 |  | 0.28 | 0.70 | ↓ | 0.68 | 17.70 |  |
| DCAF15 | DDB1 and CUL4 associated factor 15 | 91952_at | 1.86 | 3.90 |  | 0.27 | 0.29 | ↓ | 0.79 | 35.63 |  |
| PIK3R2 | phosphoinositide-3-kinase, regulatory subunit 2 (beta) | 207105_s_at | 1.86 | 4.93 |  | 0.45 | 1.45 | ↓ | 0.68 | 13.22 |  |
| AGRN | agrin | 217419_x_at | 1.85 | 3.54 |  | 0.38 | 0.98 | ↓ | 0.52 | 3.36 |  |
| ATF7 | activating transcription factor 7 | 244587_at | 1.85 | 3.90 |  | 0.47 | 3.81 | ↓ | 1.10 | 52.56 |  |
| GPC4 | glypican 4 | 204984_at | 1.85 | 7.26 |  | 2.76 | 0.76 | ↑ | 1.68 | 15.27 |  |
| CELSR1 | cadherin, EGF LAG seven-pass G-type receptor 1 (flamingo homolog, Drosophila) | 41660_at | 1.85 | 11.75 |  | 1.69 | 15.95 |  | 0.19 | 0.21 | ↓ |
| CDK16 | cyclin-dependent kinase 16 | 207239_s_at | 1.85 | 2.18 |  | 0.33 | 0.18 | ↓ | 0.65 | 8.02 |  |
| FGFR1 | fibroblast growth factor receptor 1 | 210973_s_at | 1.85 | 6.30 |  | 0.11 | 0.00 | ↓ | 1.03 | 56.10 |  |
| RXRB | retinoid X receptor, beta | 215099_s_at | 1.84 | 3.90 |  | 0.20 | 0.00 | ↓ | 1.08 | 54.85 |  |
| ITGB1 | integrin, beta 1 (fibronectin receptor, beta polypeptide, antigen CD29 includes MDF2, MSK12) | 216178_x_at | 1.84 | 12.80 |  | 0.62 | 14.26 |  | 2.23 | 2.70 | ↑ |
| PCDHGA1 /// PCDHGA10 /// PCDHGA11 /// PCDHGA12 /// PCDHGA2 /// PCDHGA3 /// PCDHGA4 /// PCDHGA5 /// PCDHGA6 /// PCDHGA7 /// PCDHGA8 /// PCDHGA9 /// PCDHGB1 /// PCDHGB2 /// PCDHGB3 /// PCDHGB4 /// PCDHGB5 /// PCDHGB6 /// PCDHGB7 /// PCDHGC3 /// PCDHGC4 /// PCDHGC5 | protocadherin gamma subfamily A, 1 /// protocadherin gamma subfamily A, 10 /// protocadherin gamma subfamily A, 11 /// protocadherin gamma subfamily A, 12 /// protocadherin gamma subfamily A, 2 /// protocadherin gamma subfamily A, 3 /// protocadherin gamma subfamily A, 4 /// protocadherin gamma subfamily A, 5 /// protocadherin gamma subfamily A, 6 /// protocadherin gamma subfamily A, 7 /// protocadherin gamma subfamily A, 8 /// protocadherin gamma subfamily A, 9 /// protocadherin gamma subfamily B, 1 /// protocadherin gamma subfamily B, 2 /// protocadherin gamma subfamily B, 3 /// protocadherin gamma subfamily B, 4 /// protocadherin gamma subfamily B, 5 /// protocadherin gamma subfamily B, 6 /// protocadherin gamma subfamily B, 7 /// protocadherin gamma subfamily C, 3 /// protocadherin gamma subfamily C, 4 /// protocadherin gamma subfamily C, 5 | 215836_s_at | 1.84 | 4.38 |  | 0.32 | 0.29 | ↓ | 1.09 | 52.56 |  |
| COL6A1 | collagen, type VI, alpha 1 | 212938_at | 1.83 | 8.24 |  | 0.17 | 0.53 | ↓ | 0.46 | 17.70 |  |
| CCL28 | chemokine (C-C motif) ligand 28 | 224240_s_at | 1.83 | 22.56 |  | 1.46 | 45.34 |  | 0.46 | 9.54 | ↓ |
| NRG1 | neuregulin 1 | 206237_s_at | 1.83 | 11.75 |  | 1.51 | 26.77 |  | 0.40 | 7.00 | ↓ |
| PHACTR2 | phosphatase and actin regulator 2 | 244774_at | 1.82 | 59.06 |  | 1.92 | 7.87 |  | 9.30 | 0.89 | ↑ |
| FLYWCH1 | FLYWCH-type zinc finger 1 | 234106_s_at | 1.82 | 3.54 |  | 0.40 | 0.40 | ↓ | 0.86 | 35.63 |  |
| SLC1A3 | solute carrier family 1 (glial high affinity glutamate transporter), member 3 | 202800_at | 1.82 | 9.04 |  | 0.77 | 29.01 |  | 0.33 | 0.89 | ↓ |
| COL6A2 | collagen, type VI, alpha 2 | 209156_s_at | 1.82 | 1.20 |  | 0.34 | 0.00 | ↓ | 0.47 | 0.34 | ↓ |
| COL11A1 | collagen, type XI, alpha 1 | 37892_at | 1.82 | 9.04 |  | 2.00 | 6.11 | ↑ | 1.32 | 41.22 |  |
| COL11A1 | collagen, type XI, alpha 1 | 229271_x_at | 1.82 | 14.24 |  | 2.08 | 9.01 | ↑ | 1.82 | 9.54 |  |
| PLD3 | phospholipase D family, member 3 | 201050_at | 1.81 | 3.21 |  | 0.24 | 0.00 | ↓ | 0.92 | 46.35 |  |
| SLC37A4 | solute carrier family 37 (glucose-6-phosphate transporter), member 4 | 217289_s_at | 1.80 | 8.24 |  | 0.41 | 1.07 | ↓ | 0.67 | 11.20 |  |
| UNC5B | unc-5 homolog B (C. elegans) | 226899_at | 1.80 | 6.30 |  | 2.25 | 7.87 | ↑ | 0.35 | 1.25 | ↓ |
| TEAD2 | TEA domain family member 2 | 238323_at | 1.80 | 6.30 |  | 0.20 | 0.25 | ↓ | 0.32 | 3.36 | ↓ |
| SLC9A3R2 | solute carrier family 9 (sodium/hydrogen exchanger), member 3 regulator 2 | 209830_s_at | 1.80 | 14.24 |  | 0.21 | 0.25 | ↓ | 0.61 | 11.20 |  |
| BCL7A | B-cell CLL/lymphoma 7A | 203796_s_at | 1.79 | 10.25 |  | 1.19 | 36.20 |  | 2.07 | 4.16 | ↑ |
| KDELR1 | KDEL (Lys-Asp-Glu-Leu) endoplasmic reticulum protein retention receptor 1 | 200922_at | 1.79 | 3.54 |  | 0.36 | 0.40 | ↓ | 0.91 | 50.73 |  |
| KDM1B | lysine (K)-specific demethylase 1B | 1553150_at | 1.78 | 14.24 |  | 0.13 | 0.25 | ↓ | 2.36 | 4.16 | ↑ |
| BHLHE40 | basic helix-loop-helix family, member e40 | 201169_s_at | 1.78 | 8.24 |  | 0.19 | 0.29 | ↓ | 0.40 | 4.84 | ↓ |
| THBS1 | thrombospondin 1 | 201107_s_at | 1.78 | 22.56 |  | 0.22 | 0.00 | ↓ | 2.13 | 3.36 | ↑ |
| RBBP9 | retinoblastoma binding protein 9 | 232751_at | 1.78 | 15.82 |  | 0.26 | 0.00 | ↓ | 2.22 | 8.02 | ↑ |
| C9orf16 | chromosome 9 open reading frame 16 | 222165_x_at | 1.78 | 1.60 |  | 0.42 | 0.00 | ↓ | 0.80 | 15.27 |  |
| LRRC15 | leucine rich repeat containing 15 | 213909_at | 1.78 | 12.80 |  | 0.74 | 33.65 |  | 0.41 | 4.16 | ↓ |
| SCAMP4 | secretory carrier membrane protein 4 | 235073_at | 1.78 | 11.75 |  | 0.35 | 2.80 | ↓ | 1.46 | 17.70 |  |
| FSCN1 | fascin homolog 1, actin-bundling protein (Strongylocentrotus purpuratus) | 210933_s_at | 1.77 | 3.21 |  | 0.16 | 0.00 | ↓ | 0.72 | 15.27 |  |
| PEA15 | phosphoprotein enriched in astrocytes 15 | 200787_s_at | 1.77 | 3.54 |  | 0.27 | 0.00 | ↓ | 1.26 | 26.48 |  |
| HPCAL1 | hippocalcin-like 1 | 205462_s_at | 1.77 | 3.54 |  | 0.33 | 0.29 | ↓ | 0.40 | 1.02 | ↓ |
| CHN1 | chimerin (chimaerin) 1 | 212624_s_at | 1.77 | 15.82 |  | 1.03 | 50.64 |  | 0.43 | 2.43 | ↓ |
| IRS2 | insulin receptor substrate 2 | 209184_s_at | 1.77 | 11.75 |  | 2.22 | 22.17 |  | 2.53 | 1.43 | ↑ |
| LOC728730 | Hypothetical LOC728730 | 242835_s_at | 1.77 | 9.04 |  | 2.40 | 2.30 | ↑ | 1.53 | 11.20 |  |
| CERCAM | cerebral endothelial cell adhesion molecule | 224794_s_at | 1.76 | 1.40 |  | 0.42 | 0.25 | ↓ | 0.91 | 38.27 |  |
| SLC26A6 | solute carrier family 26, member 6 | 221572_s_at | 1.76 | 7.26 |  | 0.68 | 7.87 |  | 0.49 | 2.70 | ↓ |
| GRPEL2 | GrpE-like 2, mitochondrial (E. coli) | 238427_at | 1.76 | 12.80 |  | 0.32 | 2.80 | ↓ | 1.29 | 41.22 |  |
| TRIM28 | tripartite motif-containing 28 | 200990_at | 1.76 | 3.90 |  | 0.32 | 0.25 | ↓ | 0.55 | 4.16 |  |
| KCND3 | potassium voltage-gated channel, Shal-related subfamily, member 3 | 213832_at | 1.76 | 32.33 |  | 4.12 | 0.40 | ↑ | 1.18 | 46.35 |  |
| FRMPD4 | FERM and PDZ domain containing 4 | 215052_at | 1.76 | 10.25 |  | — | — |  | 2.77 | 1.43 | ↑ |
| C19orf29 | chromosome 19 open reading frame 29 | 215954_s_at | 1.76 | 3.54 |  | 0.46 | 1.26 | ↓ | 0.89 | 41.22 |  |
| SNX19 | sorting nexin 19 | 1554986_a_at | 1.76 | 17.32 |  | 0.41 | 0.84 | ↓ | 1.17 | 46.35 |  |
| KCNK1 | potassium channel, subfamily K, member 1 | 204678_s_at | 1.76 | 9.04 |  | 1.26 | 38.78 |  | 0.30 | 1.99 | ↓ |
| NUCB1 | nucleobindin 1 | 200649_at | 1.76 | 3.21 |  | 0.23 | 0.00 | ↓ | 0.85 | 32.58 |  |
| PTPRS | protein tyrosine phosphatase, receptor type, S | 226571_s_at | 1.75 | 14.24 |  | 3.34 | 0.21 | ↑ | 0.98 | 54.08 |  |
| ELK3 | ELK3, ETS-domain protein (SRF accessory protein 2) | 206127_at | 1.75 | 7.26 |  | 0.37 | 0.36 | ↓ | 1.17 | 43.83 |  |
| TMEM223 | transmembrane protein 223 | 220934_s_at | 1.75 | 4.93 |  | 3.07 | 0.45 | ↑ | 0.69 | 9.54 |  |
| FZR1 | fizzy/cell division cycle 20 related 1 (Drosophila) | 209416_s_at | 1.75 | 6.30 |  | 0.31 | 0.60 | ↓ | 1.04 | 54.85 |  |
| EPM2AIP1 | EPM2A (laforin) interacting protein 1 | 236314_at | 1.75 | 24.35 |  | 1.79 | 19.82 |  | 0.21 | 0.72 | ↓ |
| NUDT10 | nudix (nucleoside diphosphate linked moiety X)-type motif 10 | 241596_at | 1.75 | 26.15 |  | 2.09 | 7.87 | ↑ | 0.96 | 52.56 |  |
| LOC100288985 | Hypothetical protein LOC100288985 | 230746_s_at | 1.74 | 22.56 |  | 0.23 | 1.74 | ↓ | 0.89 | 58.81 |  |
| COL4A2 | collagen, type IV, alpha 2 | 211966_at | 1.74 | 14.24 |  | 0.42 | 9.01 | ↓ | 0.55 | 17.70 |  |
| RBM14 | RNA binding motif protein 14 | 1555639_a_at | 1.74 | 8.24 |  | 0.32 | 1.26 | ↓ | 1.02 | 55.52 |  |
| BCOR | BCL6 corepressor | 223916_s_at | 1.74 | 20.86 |  | 0.49 | 3.35 | ↓ | 0.86 | 38.27 |  |
| PVRL2 | poliovirus receptor-related 2 (herpesvirus entry mediator B) | 232079_s_at | 1.73 | 6.30 |  | 0.30 | 0.60 | ↓ | 0.79 | 41.22 |  |
| ZMAT1 | zinc finger, matrin-type 1 | 226344_at | 1.73 | 12.80 |  | 2.24 | 6.11 | ↑ | 0.96 | 54.08 |  |
| CAPN5 | calpain 5 | 205166_at | 1.73 | 4.38 |  | 0.37 | 0.36 | ↓ | 0.70 | 13.22 |  |
| FLNA | filamin A, alpha | 213746_s_at | 1.73 | 4.93 |  | 0.46 | 1.98 | ↓ | 0.72 | 20.38 |  |
| C10orf118 | chromosome 10 open reading frame 118 | 229399_at | 1.73 | 18.98 |  | 2.16 | 1.98 | ↑ | 1.26 | 41.22 |  |
| ZNF204P | zinc finger protein 204, pseudogene | 214823_at | 1.73 | 8.24 |  | 1.65 | 24.54 |  | 0.35 | 4.16 | ↓ |
| PLCB4 | phospholipase C, beta 4 | 203896_s_at | 1.72 | 14.24 |  | 2.29 | 12.66 |  | 2.20 | 5.75 | ↑ |
| BSG | basigin (Ok blood group) | 208677_s_at | 1.72 | 2.18 |  | 0.39 | 0.40 | ↓ | 1.10 | 54.08 |  |
| CA12 | carbonic anhydrase XII | 210735_s_at | 1.72 | 6.30 |  | 0.40 | 1.45 | ↓ | 1.20 | 41.22 |  |
| ABCC1 | ATP-binding cassette, sub-family C (CFTR/MRP), member 1 | 202805_s_at | 1.72 | 6.30 |  | 0.42 | 0.18 | ↓ | 1.12 | 46.35 |  |
| PRKAR1B | protein kinase, cAMP-dependent, regulatory, type I, beta | 212559_at | 1.72 | 2.70 |  | 0.49 | 3.81 | ↓ | 1.00 | 54.08 |  |
| ADAT3 | adenosine deaminase, tRNA-specific 3, TAD3 homolog (S. cerevisiae) | 1553967_at | 1.72 | 54.53 |  | 0.32 | 4.45 | ↓ | 0.94 | 58.81 |  |
| FAM85A | family with sequence similarity 85, member A | 227917_at | 1.72 | 17.32 |  | 1.05 | 52.07 |  | 0.46 | 4.84 | ↓ |
| TRIM3 | tripartite motif-containing 3 | 204911_s_at | 1.71 | 8.24 |  | 0.37 | 1.26 | ↓ | 0.80 | 32.58 |  |
| ZNF395 | zinc finger protein 395 | 222536_s_at | 1.71 | 12.80 |  | 1.55 | 41.29 |  | 0.47 | 7.00 | ↓ |
| TNFRSF11A | tumor necrosis factor receptor superfamily, member 11a, NFKB activator | 238846_at | 1.71 | 32.33 |  | 0.62 | 11.08 |  | 3.77 | 4.16 | ↑ |
| FGFR2 | fibroblast growth factor receptor 2 | 208228_s_at | 1.71 | 15.82 |  | 1.49 | 22.17 |  | 0.36 | 1.61 | ↓ |
| SLC1A4 | solute carrier family 1 (glutamate/neutral amino acid transporter), member 4 | 209611_s_at | 1.71 | 10.25 |  | 0.59 | 26.77 |  | 0.36 | 4.84 | ↓ |
| RGNEF | 190 kDa guanine nucleotide exchange factor | 1560348_at | 1.71 | 6.30 |  | 0.52 | 3.81 |  | 2.00 | 2.70 | ↑ |
| EMD | emerin | 209477_at | 1.71 | 3.90 |  | 2.15 | 1.26 | ↑ | 1.05 | 56.65 |  |
| FAM129B | family with sequence similarity 129, member B | 233974_s_at | 1.71 | 5.70 |  | 0.32 | 0.25 | ↓ | 1.38 | 41.22 |  |
| HDAC9 | histone deacetylase 9 | 205659_at | 1.71 | 12.80 |  | 1.20 | 53.46 |  | 6.30 | 0.31 | ↑ |
| MRC2 | mannose receptor, C type 2 | 37408_at | 1.70 | 6.30 |  | 1.59 | 11.08 |  | 0.48 | 0.89 | ↓ |
| KLHL22 | kelch-like 22 (Drosophila) | 222141_at | 1.70 | 24.35 |  | 1.06 | 53.46 |  | 0.46 | 9.54 | ↓ |
| C20orf20 | chromosome 20 open reading frame 20 | 218586_at | 1.70 | 22.56 |  | 2.06 | 1.98 | ↑ | 0.72 | 9.54 |  |
| RBM39 | RNA binding motif protein 39 | 226404_at | 1.70 | 3.54 |  | 2.66 | 2.80 | ↑ | 0.45 | 0.00 | ↓ |
| THRA | thyroid hormone receptor, alpha (erythroblastic leukemia viral (v-erb-a) oncogene homolog, avian) | 1316_at | 1.70 | 56.74 |  | 2.51 | 0.60 | ↑ | 1.25 | 26.48 |  |
| VCAM1 | vascular cell adhesion molecule 1 | 203868_s_at | 1.70 | 11.75 |  | 1.33 | 31.53 |  | 0.43 | 5.75 | ↓ |
| RALGPS2 | Ral GEF with PH domain and SH3 binding motif 2 | 242458_at | 1.70 | 17.32 |  | 0.47 | 0.98 | ↓ | 1.47 | 32.58 |  |
| HES1 | hairy and enhancer of split 1, (Drosophila) | 203395_s_at | 1.70 | 10.25 |  | 1.72 | 19.82 |  | 0.42 | 5.75 | ↓ |
| RAC2 | ras-related C3 botulinum toxin substrate 2 (rho family, small GTP binding protein Rac2) | 207419_s_at | 1.69 | 11.75 |  | 0.19 | 0.45 | ↓ | 0.72 | 41.22 |  |
| DUXAP10 | Double homeobox A pseudogene 10 | 239010_at | 1.69 | 26.15 |  | 2.25 | 7.87 | ↑ | 1.69 | 55.52 |  |
| RARG | retinoic acid receptor, gamma | 204189_at | 1.69 | 17.32 |  | 0.44 | 0.45 | ↓ | 0.97 | 48.62 |  |
| CPE | carboxypeptidase E | 201117_s_at | 1.69 | 53.22 |  | 0.85 | 56.87 |  | 5.06 | 2.43 | ↑ |
| SH3GLB2 | SH3-domain GRB2-like endophilin B2 | 224907_s_at | 1.69 | 5.70 |  | 0.47 | 2.80 | ↓ | 1.08 | 54.08 |  |
| ELMO2 | engulfment and cell motility 2 | 220363_s_at | 1.69 | 8.24 |  | 0.38 | 1.07 | ↓ | 1.19 | 41.22 |  |
| TAPBP | TAP binding protein (tapasin) | 1555565_s_at | 1.69 | 12.80 |  | 0.34 | 0.70 | ↓ | 1.08 | 52.56 |  |
| RUNX3 | runt-related transcription factor 3 | 204197_s_at | 1.69 | 6.30 |  | 0.94 | 49.04 |  | 0.26 | 1.02 | ↓ |
| TPP1 | tripeptidyl peptidase I | 214196_s_at | 1.68 | 14.24 |  | 0.20 | 0.00 | ↓ | 0.86 | 38.27 |  |
| RNF126 | ring finger protein 126 | 205748_s_at | 1.68 | 10.25 |  | 0.35 | 0.36 | ↓ | 0.67 | 9.54 |  |
| AGPAT5 | 1-acylglycerol-3-phosphate O-acyltransferase 5 (lysophosphatidic acid acyltransferase, epsilon) | 232007_at | 1.68 | 43.91 |  | 1.70 | 24.54 |  | 0.37 | 0.34 | ↓ |
| PLXNA3 | plexin A3 | 1553139_s_at | 1.68 | 9.04 |  | 0.06 | 0.00 | ↓ | 1.04 | 58.81 |  |
| TMEM119 | transmembrane protein 119 | 227300_at | 1.68 | 11.75 |  | 0.65 | 22.17 |  | 0.33 | 2.70 | ↓ |
| SETD5 | SET domain containing 5 | 1569106_s_at | 1.68 | 26.15 |  | 0.11 | 0.00 | ↓ | 0.78 | 29.62 |  |
| FKBP10 | FK506 binding protein 10, 65 kDa | 219249_s_at | 1.67 | 10.25 |  | 2.07 | 5.22 | ↑ | 0.64 | 13.22 |  |
| TNFSF4 | tumor necrosis factor (ligand) superfamily, member 4 | 207426_s_at | 1.67 | 7.26 |  | 2.47 | 9.01 | ↑ | 0.96 | 46.35 |  |
| APOE | apolipoprotein E | 203381_s_at | 1.67 | 47.41 |  | 0.62 | 11.08 |  | 0.27 | 0.50 | ↓ |
| ISYNA1 | inositol-3-phosphate synthase 1 | 222240_s_at | 1.67 | 6.30 |  | 0.24 | 0.40 | ↓ | 0.72 | 20.38 |  |
| ZNF580 | zinc finger protein 580 | 220748_s_at | 1.67 | 11.75 |  | 0.45 | 9.01 | ↓ | 0.78 | 43.83 |  |
| TYRO3 | TYRO3 protein tyrosine kinase | 211431_s_at | 1.67 | 11.75 |  | 0.43 | 2.80 | ↓ | 1.03 | 57.92 |  |
| POLD1 | polymerase (DNA directed), delta 1, catalytic subunit 125kDa | 203422_at | 1.67 | 7.26 |  | 0.75 | 26.77 |  | 0.41 | 0.34 | ↓ |
| ECM1 | extracellular matrix protein 1 | 209365_s_at | 1.67 | 11.75 |  | 0.48 | 1.98 | ↓ | 1.92 | 29.62 |  |
| BTN3A1 | butyrophilin, subfamily 3, member A1 | 207485_x_at | 1.66 | 9.04 |  | 0.49 | 3.81 | ↓ | 0.80 | 29.62 |  |
| SYT12 | synaptotagmin XII | 228072_at | 1.66 | 18.98 |  | 0.87 | 59.66 |  | 0.25 | 1.25 | ↓ |
| C20orf117 | chromosome 20 open reading frame 117 | 225473_at | 1.66 | 1.90 |  | 0.46 | 0.18 | ↓ | 0.74 | 9.54 |  |
| CRTC1 | CREB regulated transcription coactivator 1 | 207159_x_at | 1.66 | 9.04 |  | 0.45 | 1.74 | ↓ | 1.05 | 58.81 |  |
| LOC645722 | hypothetical protein LOC645722 | 1555216_a_at | 1.66 | 28.36 |  | 0.83 | 50.64 |  | 0.39 | 2.43 | ↓ |
| GRIA3 | glutamate receptor, ionotrophic, AMPA 3 | 1569290_s_at | 1.66 | 6.30 |  | 1.09 | 60.11 |  | 2.92 | 0.43 | ↑ |
| PSG1 | pregnancy specific beta-1-glycoprotein 1 | 210195_s_at | 1.66 | 26.15 |  | 0.82 | 36.20 |  | 3.77 | 5.75 | ↑ |
| LOC643072 | hypothetical LOC643072 | 228412_at | 1.66 | 11.75 |  | 1.05 | 50.64 |  | 0.49 | 5.75 | ↓ |
| NKD2 | naked cuticle homolog 2 (Drosophila) | 232201_at | 1.66 | 8.24 |  | 1.06 | 38.78 |  | 0.44 | 2.70 | ↓ |
| CCRN4L | CCR4 carbon catabolite repression 4-like (S. cerevisiae) | 1554283_at | 1.66 | 11.75 |  | 2.28 | 0.98 | ↑ | 0.80 | 23.22 |  |
| ADM | adrenomedullin | 202912_at | 1.65 | 10.25 |  | 1.02 | 55.85 |  | 2.29 | 1.61 | ↑ |
| IMPA2 | inositol(myo)-1(or 4)-monophosphatase 2 | 203126_at | 1.65 | 14.24 |  | 0.93 | 59.12 |  | 0.34 | 3.36 | ↓ |
| MAZ | MYC-associated zinc finger protein (purine-binding transcription factor) | 212064_x_at | 1.65 | 4.93 |  | 0.26 | 0.00 | ↓ | 0.73 | 17.70 |  |
| PNPLA6 | patatin-like phospholipase domain containing 6 | 203718_at | 1.65 | 10.25 |  | 0.39 | 1.26 | ↓ | 0.80 | 35.63 |  |
| PDE4D | phosphodiesterase 4D, cAMP-specific | 204491_at | 1.65 | 8.24 |  | 1.27 | 36.20 |  | 0.24 | 0.00 | ↓ |
| DACT1 | dapper, antagonist of beta-catenin, homolog 1 (Xenopus laevis) | 219179_at | 1.65 | 20.86 |  | 3.16 | 0.53 | ↑ | 1.38 | 17.70 |  |
| IGFBP4 | insulin-like growth factor binding protein 4 | 201508_at | 1.65 | 2.46 |  | 0.39 | 0.84 | ↓ | 0.99 | 54.08 |  |
| EIF4EBP1 | eukaryotic translation initiation factor 4E binding protein 1 | 221539_at | 1.65 | 15.82 |  | 1.30 | 31.53 |  | 0.34 | 2.70 | ↓ |
| NAPA | N-ethylmaleimide-sensitive factor attachment protein, alpha | 208751_at | 1.65 | 3.90 |  | 0.23 | 0.00 | ↓ | 1.11 | 46.35 |  |
| HTR2A | 5-hydroxytryptamine (serotonin) receptor 2A | 211616_s_at | 1.65 | 28.36 |  | 0.48 | 7.87 | ↓ | 1.87 | 11.20 |  |
| RTN2 | reticulon 2 | 204217_s_at | 1.65 | 3.90 |  | 0.47 | 0.70 | ↓ | 1.13 | 48.62 |  |
| MOSPD3 | motile sperm domain containing 3 | 219070_s_at | 1.64 | 4.93 |  | 0.44 | 0.45 | ↓ | 0.93 | 46.35 |  |
| KCTD5 | potassium channel tetramerisation domain containing 5 | 222645_s_at | 1.64 | 5.70 |  | 0.47 | 1.07 | ↓ | 1.04 | 58.28 |  |
| INO80B | INO80 complex subunit B | 65133_i_at | 1.64 | 5.70 |  | 0.42 | 0.84 | ↓ | 0.87 | 41.22 |  |
| VDAC1 | voltage-dependent anion channel 1 | 217139_at | 1.64 | 28.36 |  | 0.48 | 6.80 | ↓ | 0.86 | 43.83 |  |
| RAB3D | RAB3D, member RAS oncogene family | 225001_at | 1.64 | 22.56 |  | 0.49 | 4.45 | ↓ | 0.69 | 15.27 |  |
| LSS | lanosterol synthase (2,3-oxidosqualene-lanosterol cyclase) | 211019_s_at | 1.64 | 15.82 |  | 0.33 | 1.07 | ↓ | 1.18 | 43.83 |  |
| AGRN | agrin | 212285_s_at | 1.64 | 12.80 |  | 0.42 | 1.26 | ↓ | 0.51 | 1.99 |  |
| FMO3 | flavin containing monooxygenase 3 | 40665_at | 1.64 | 45.69 |  | 4.39 | 4.45 | ↑ | 0.40 | 9.54 | ↓ |
| TRPV2 | transient receptor potential cation channel, subfamily V, member 2 | 222855_s_at | 1.64 | 15.82 |  | 0.17 | 0.18 | ↓ | 0.72 | 20.38 |  |
| HGS | hepatocyte growth factor-regulated tyrosine kinase substrate | 210428_s_at | 1.64 | 7.26 |  | 0.47 | 1.74 | ↓ | 0.74 | 20.38 |  |
| A2M | alpha-2-macroglobulin | 217757_at | 1.64 | 61.16 |  | 1.85 | 33.65 |  | 0.31 | 1.99 | ↓ |
| PFKFB4 | 6-phosphofructo-2-kinase/fructose-2,6-biphosphatase 4 | 228499_at | 1.64 | 34.35 |  | 1.03 | 56.87 |  | 0.19 | 2.70 | ↓ |
| C1orf38 | chromosome 1 open reading frame 38 | 207571_x_at | 1.64 | 7.26 |  | 0.72 | 22.17 |  | 0.44 | 1.99 | ↓ |
| FBXW11 | F-box and WD repeat domain containing 11 | 209456_s_at | 1.64 | 11.75 |  | 0.28 | 0.00 | ↓ | 1.33 | 41.22 |  |
| LPCAT4 | lysophosphatidylcholine acyltransferase 4 | 239609_s_at | 1.63 | 9.04 |  | 1.23 | 47.13 |  | 0.48 | 3.36 | ↓ |
| IGFBP5 | insulin-like growth factor binding protein 5 | 211958_at | 1.63 | 15.82 |  | 0.69 | 31.53 |  | 3.81 | 3.36 | ↑ |
| RRBP1 | ribosome binding protein 1 homolog 180kDa (dog) | 201203_s_at | 1.63 | 6.30 |  | 0.35 | 0.18 | ↓ | 0.62 | 3.36 |  |
| RGNEF | 190 kDa guanine nucleotide exchange factor | 219610_at | 1.63 | 15.82 |  | 1.12 | 60.11 |  | 2.29 | 8.02 | ↑ |
| CREB3L1 | cAMP responsive element binding protein 3-like 1 | 213498_at | 1.63 | 10.25 |  | 0.13 | 0.18 | ↓ | 0.58 | 29.62 |  |
| KPNA6 | karyopherin alpha 6 (importin alpha 7) | 212103_at | 1.63 | 2.46 |  | 2.44 | 0.14 | ↑ | 0.87 | 23.22 |  |
| HIPK1 | homeodomain interacting protein kinase 1 | 212291_at | 1.63 | 20.86 |  | 0.21 | 0.25 | ↓ | 1.26 | 35.63 |  |
| ZNF93 | zinc finger protein 93 | 1569241_a_at | 1.63 | 48.99 |  | 0.89 | 50.64 |  | 0.21 | 0.21 | ↓ |
| KCTD11 | potassium channel tetramerisation domain containing 11 | 235857_at | 1.62 | 32.33 |  | 2.64 | 9.01 | ↑ | 0.63 | 15.27 |  |
| ZER1 | zer-1 homolog (C. elegans) | 202448_s_at | 1.62 | 7.26 |  | 0.19 | 0.18 | ↓ | 1.04 | 54.08 |  |
| RELA | v-rel reticuloendotheliosis viral oncogene homolog A (avian) | 209878_s_at | 1.62 | 14.24 |  | 0.38 | 1.74 | ↓ | 1.05 | 54.08 |  |
| HHLA3 | HERV-H LTR-associating 3 | 220387_s_at | 1.62 | 9.04 |  | 0.44 | 0.45 | ↓ | 0.66 | 8.02 |  |
| MARCH6 | membrane-associated ring finger (C3HC4) 6 | 215512_at | 1.62 | 17.32 |  | 0.36 | 3.35 | ↓ | 1.13 | 54.85 |  |
| AKIRIN2 | akirin 2 | 223143_s_at | 1.62 | 11.75 |  | 0.19 | 0.00 | ↓ | 1.20 | 46.35 |  |
| GM2A | GM2 ganglioside activator | 209727_at | 1.62 | 38.54 |  | 0.30 | 0.84 | ↓ | 1.40 | 26.48 |  |
| MARCKS | myristoylated alanine-rich protein kinase C substrate | 201668_x_at | 1.62 | 10.25 |  | 0.30 | 0.70 | ↓ | 0.56 | 13.22 |  |
| EPHX1 | epoxide hydrolase 1, microsomal (xenobiotic) | 202017_at | 1.61 | 14.24 |  | 0.20 | 0.00 | ↓ | 1.02 | 52.56 |  |
| SMAD3 | SMAD family member 3 | 205397_x_at | 1.61 | 14.24 |  | 0.04 | 0.00 | ↓ | 0.73 | 15.27 |  |
| ZNF229 | zinc finger protein 229 | 1562789_at | 1.61 | 28.36 |  | 0.50 | 1.26 | ↓ | 1.02 | 58.51 |  |
| TRAPPC1 | trafficking protein particle complex 1 | 225294_s_at | 1.61 | 5.70 |  | 0.16 | 0.00 | ↓ | 0.92 | 48.62 |  |
| YOD1 | YOD1 OTU deubiquinating enzyme 1 homolog (S. cerevisiae) | 215150_at | 1.61 | 42.20 |  | 0.16 | 0.18 | ↓ | 1.55 | 9.54 |  |
| BAT2L1 | HLA-B associated transcript 2-like 1 | 212069_s_at | 1.61 | 14.24 |  | 0.39 | 1.07 | ↓ | 0.85 | 43.83 |  |
| EIF4E3 | eukaryotic translation initiation factor 4E family member 3 | 225939_at | 1.61 | 15.82 |  | 2.33 | 4.45 | ↑ | 0.96 | 38.27 |  |
| FMO3 | flavin containing monooxygenase 3 | 206496_at | 1.61 | 34.35 |  | 3.13 | 10.02 |  | 0.30 | 5.75 | ↓ |
| DDX11 /// DDX12 /// LOC642846 | DEAD/H (Asp-Glu-Ala-Asp/His) box polypeptide 11 /// DEAD/H (Asp-Glu-Ala-Asp/His) box polypeptide 12 /// DEAD/H (Asp-Glu-Ala-Asp/His) box polypeptide 11-like | 213378_s_at | 1.61 | 15.82 |  | 0.47 | 6.11 | ↓ | 0.64 | 20.38 |  |
| PFKP | phosphofructokinase, platelet | 201037_at | 1.61 | 9.04 |  | 1.01 | 60.11 |  | 0.38 | 0.72 | ↓ |
| RC3H2 | ring finger and CCCH-type domains 2 | 230134_s_at | 1.61 | 9.04 |  | 0.29 | 0.18 | ↓ | 1.28 | 38.27 |  |
| SOX4 | SRY (sex determining region Y)-box 4 | 201416_at | 1.61 | 30.22 |  | 3.91 | 11.08 |  | 0.17 | 0.00 | ↓ |
| DUSP6 | dual specificity phosphatase 6 | 208891_at | 1.61 | 24.35 |  | 0.90 | 45.34 |  | 0.42 | 0.34 | ↓ |
| C14orf34 | chromosome 14 open reading frame 34 | 1555786_s_at | 1.61 | 36.49 |  | 0.48 | 0.70 | ↓ | 1.37 | 17.70 |  |
| ZER1 | zer-1 homolog (C. elegans) | 202456_s_at | 1.61 | 10.25 |  | 0.42 | 2.30 | ↓ | 1.09 | 54.08 |  |
| SLC35A2 | solute carrier family 35 (UDP-galactose transporter), member A2 | 207439_s_at | 1.60 | 4.93 |  | 0.26 | 0.00 | ↓ | 0.95 | 50.73 |  |
| SNHG12 | small nucleolar RNA host gene 12 (non-protein coding) | 223774_at | 1.60 | 22.56 |  | 1.27 | 26.77 |  | 0.42 | 0.50 | ↓ |
| SLC35F2 | solute carrier family 35, member F2 | 218826_at | 1.60 | 17.32 |  | 1.09 | 57.71 |  | 0.36 | 0.58 | ↓ |
| PCSK5 | proprotein convertase subtilisin/kexin type 5 | 205560_at | 1.60 | 11.75 |  | 2.96 | 4.45 | ↑ | 0.87 | 43.83 |  |
| C9orf41 | chromosome 9 open reading frame 41 | 241781_at | 1.60 | 12.80 |  | 0.50 | 4.45 | ↓ | 1.02 | 58.81 |  |
| SYPL2 | synaptophysin-like 2 | 230611_at | 1.60 | 28.36 |  | 0.42 | 1.98 | ↓ | 1.31 | 20.38 |  |
| FOSL2 | FOS-like antigen 2 | 218881_s_at | 1.60 | 14.24 |  | 0.33 | 1.74 | ↓ | 0.92 | 54.08 |  |
| ZNF275 | zinc finger protein 275 | 225382_at | 1.60 | 3.90 |  | 0.33 | 0.00 | ↓ | 0.71 | 9.54 |  |
| ZFHX3 | zinc finger homeobox 3 | 208033_s_at | 1.59 | 9.04 |  | 0.41 | 0.98 | ↓ | 0.84 | 35.63 |  |
| BCL2L1 | BCL2-like 1 | 206665_s_at | 1.59 | 12.80 |  | 0.11 | 0.00 | ↓ | 1.55 | 5.75 |  |
| SLC12A9 | solute carrier family 12 (potassium/chloride transporters), member 9 | 223995_at | 1.59 | 43.91 |  | 2.06 | 1.98 | ↑ | 0.84 | 29.62 |  |
| SIRPA | signal-regulatory protein alpha | 202895_s_at | 1.59 | 11.75 |  | 0.29 | 0.70 | ↓ | 1.36 | 26.48 |  |
| TOM1 | target of myb1 (chicken) | 202807_s_at | 1.59 | 4.38 |  | 0.49 | 0.60 | ↓ | 1.18 | 32.58 |  |
| DOK1 | docking protein 1, 62kDa (downstream of tyrosine kinase 1) | 211121_s_at | 1.59 | 4.38 |  | 0.49 | 1.74 | ↓ | 0.86 | 32.58 |  |
| RTTN | rotatin | 1557388_at | 1.59 | 40.42 |  | 0.45 | 1.74 | ↓ | 1.05 | 57.64 |  |
| HLA-DMA | major histocompatibility complex, class II, DM alpha | 217478_s_at | 1.59 | 8.24 |  | 0.99 | 49.04 |  | 0.48 | 1.99 | ↓ |
| C19orf6 | chromosome 19 open reading frame 6 | 212574_x_at | 1.59 | 9.04 |  | 0.25 | 0.25 | ↓ | 0.95 | 58.81 |  |
| P4HB | prolyl 4-hydroxylase, beta polypeptide | 1564494_s_at | 1.59 | 11.75 |  | 0.36 | 0.60 | ↓ | 0.65 | 9.54 |  |
| CENPV | centromere protein V | 226611_s_at | 1.58 | 18.98 |  | 0.80 | 59.66 |  | 0.32 | 4.16 | ↓ |
| CECR2 | cat eye syndrome chromosome region, candidate 2 | 223729_at | 1.58 | 26.15 |  | 0.31 | 1.45 | ↓ | 1.24 | 46.35 |  |
| HDAC9 | histone deacetylase 9 | 234393_at | 1.58 | 47.41 |  | 0.51 | 4.45 |  | 2.05 | 2.70 | ↑ |
| ACBD4 | acyl-CoA binding domain containing 4 | 219413_at | 1.58 | 8.24 |  | 0.41 | 0.60 | ↓ | 0.92 | 46.35 |  |
| VPS13A | Vacuolar protein sorting 13 homolog A (S. cerevisiae) | 231585_at | 1.58 | 32.33 |  | 1.56 | 22.17 |  | 2.05 | 7.00 | ↑ |
| TNKS1BP1 | tankyrase 1 binding protein 1, 182kDa | 224792_at | 1.58 | 8.24 |  | 0.46 | 1.45 | ↓ | 0.82 | 29.62 |  |
| RRAD | Ras-related associated with diabetes | 204803_s_at | 1.58 | 38.54 |  | 0.46 | 2.30 | ↓ | 1.46 | 35.63 |  |
| LOC728855 | hypothetical LOC728855 | 222001_x_at | 1.58 | 7.26 |  | 1.32 | 31.53 |  | 0.46 | 0.50 | ↓ |
| FYN | FYN oncogene related to SRC, FGR, YES | 216033_s_at | 1.57 | 4.93 |  | 0.32 | 0.00 | ↓ | 1.15 | 46.35 |  |
| EGR3 | early growth response 3 | 206115_at | 1.57 | 30.22 |  | 2.75 | 0.98 | ↑ | 1.22 | 57.14 |  |
| CHD7 | chromodomain helicase DNA binding protein 7 | 218829_s_at | 1.57 | 15.82 |  | 1.73 | 17.74 |  | 0.34 | 1.43 | ↓ |
| PCDH10 | protocadherin 10 | 228635_at | 1.57 | 43.91 |  | 1.01 | 49.04 |  | 4.92 | 2.70 | ↑ |
| SLC25A37 | solute carrier family 25, member 37 | 221920_s_at | 1.57 | 14.24 |  | 1.05 | 50.64 |  | 0.48 | 1.99 | ↓ |
| C3orf21 | chromosome 3 open reading frame 21 | 226891_at | 1.57 | 15.82 |  | 2.43 | 0.70 | ↑ | — | — |  |
| RAPH1 | Ras association (RalGDS/AF-6) and pleckstrin homology domains 1 | 231075_x_at | 1.57 | 10.25 |  | 3.15 | 0.76 | ↑ | 1.30 | 26.48 |  |
| PKP4 | plakophilin 4 | 201927_s_at | 1.56 | 17.32 |  | 2.21 | 1.74 | ↑ | 1.24 | 32.58 |  |
| CTTN | cortactin | 214782_at | 1.56 | 28.36 |  | 3.06 | 9.01 | ↑ | 0.86 | 54.08 |  |
| LCAT | lecithin-cholesterol acyltransferase | 204428_s_at | 1.56 | 18.98 |  | 0.61 | 6.80 |  | 0.35 | 0.34 | ↓ |
| AKT2 | v-akt murine thymoma viral oncogene homolog 2 | 203809_s_at | 1.56 | 36.49 |  | 0.31 | 1.07 | ↓ | 1.16 | 46.35 |  |
| PPP2R1A | protein phosphatase 2, regulatory subunit A, alpha | 200695_at | 1.56 | 6.30 |  | 0.35 | 0.00 | ↓ | 1.51 | 4.84 |  |
| CIRBP | cold inducible RNA binding protein | 225191_at | 1.56 | 15.82 |  | 0.82 | 31.53 |  | 0.30 | 0.00 | ↓ |
| CLPTM1 | cleft lip and palate associated transmembrane protein 1 | 201640_x_at | 1.56 | 8.24 |  | 0.32 | 0.18 | ↓ | 1.01 | 58.81 |  |
| AOX1 | aldehyde oxidase 1 | 205082_s_at | 1.56 | 43.91 |  | 0.71 | 12.66 |  | 4.51 | 0.31 | ↑ |
| DLX5 | distal-less homeobox 5 | 213707_s_at | 1.56 | 15.82 |  | 1.09 | 50.64 |  | 0.49 | 5.75 | ↓ |
| CIRBP | cold inducible RNA binding protein | 230142_s_at | 1.56 | 12.80 |  | 0.90 | 45.34 |  | 0.47 | 0.34 | ↓ |
| AAAS | achalasia, adrenocortical insufficiency, alacrimia | 218075_at | 1.56 | 10.25 |  | 0.44 | 2.80 | ↓ | 0.77 | 32.58 |  |
| C19orf6 | chromosome 19 open reading frame 6 | 213986_s_at | 1.56 | 14.24 |  | 0.25 | 0.18 | ↓ | 1.37 | 23.22 |  |
| PFKL | phosphofructokinase, liver | 201102_s_at | 1.55 | 14.24 |  | 0.42 | 5.22 | ↓ | 0.68 | 32.58 |  |
| RSRC1 | arginine/serine-rich coiled-coil 1 | 235354_s_at | 1.55 | 15.82 |  | 3.00 | 1.98 | ↑ | 1.40 | 29.62 |  |
| PEX5 | peroxisomal biogenesis factor 5 | 215481_s_at | 1.55 | 10.25 |  | 0.50 | 2.80 | ↓ | 1.37 | 23.22 |  |
| RALGPS2 | Ral GEF with PH domain and SH3 binding motif 2 | 232112_at | 1.55 | 24.35 |  | 0.45 | 6.11 | ↓ | 1.35 | 46.35 |  |
| --- | --- | 230299_s_at | 1.55 | 14.24 |  | 0.41 | 2.30 | ↓ | 0.95 | 50.73 |  |
| COL7A1 | collagen, type VII, alpha 1 | 204136_at | 1.55 | 24.35 |  | 1.18 | 53.46 |  | 0.50 | 0.50 | ↓ |
| CBX3 | Chromobox homolog 3 (HP1 gamma homolog, Drosophila) | 1555920_at | 1.55 | 20.86 |  | 2.97 | 0.60 | ↑ | 0.66 | 7.00 |  |
| GABBR1 | gamma-aminobutyric acid (GABA) B receptor, 1 | 203146_s_at | 1.55 | 17.32 |  | 1.04 | 58.42 |  | 0.47 | 0.21 | ↓ |
| TNRC6A | trinucleotide repeat containing 6A | 234734_s_at | 1.55 | 10.25 |  | 2.08 | 4.45 | ↑ | 0.79 | 32.58 |  |
| CYB5R3 | cytochrome b5 reductase 3 | 1554574_a_at | 1.55 | 14.24 |  | 0.17 | 0.00 | ↓ | 1.12 | 50.73 |  |
| FASN | fatty acid synthase | 212218_s_at | 1.55 | 12.80 |  | 0.38 | 4.45 | ↓ | 0.64 | 29.62 |  |
| PPP1R3B | protein phosphatase 1, regulatory (inhibitor) subunit 3B | 1552670_a_at | 1.55 | 11.75 |  | 0.31 | 0.18 | ↓ | 1.25 | 46.35 |  |
| MAGI1 | membrane associated guanylate kinase, WW and PDZ domain containing 1 | 232859_s_at | 1.55 | 32.33 |  | 2.92 | 0.29 | ↑ | 2.82 | 0.00 | ↑ |
| GSN | gelsolin | 214040_s_at | 1.54 | 9.04 |  | 0.24 | 0.25 | ↓ | 1.33 | 29.62 |  |
| HSPB7 | heat shock 27kDa protein family, member 7 (cardiovascular) | 218934_s_at | 1.54 | 40.42 |  | 0.34 | 0.76 | ↓ | 2.36 | 7.00 | ↑ |
| ASPHD1 | aspartate beta-hydroxylase domain containing 1 | 1553997_a_at | 1.54 | 12.80 |  | 0.97 | 56.87 |  | 0.47 | 2.70 | ↓ |
| CLEC11A | C-type lectin domain family 11, member A | 205131_x_at | 1.54 | 10.25 |  | 0.49 | 1.45 | ↓ | 0.56 | 1.99 |  |
| TTYH3 | tweety homolog 3 (Drosophila) | 224674_at | 1.54 | 4.93 |  | 0.49 | 1.45 | ↓ | 0.69 | 4.84 |  |
| SOX4 | SRY (sex determining region Y)-box 4 | 201417_at | 1.54 | 14.24 |  | 2.20 | 12.66 |  | 0.36 | 0.00 | ↓ |
| MYO1C | myosin IC | 32811_at | 1.54 | 8.24 |  | 2.51 | 0.60 | ↑ | 1.37 | 15.27 |  |
| CD99L2 | CD99 molecule-like 2 | 1554758_a_at | 1.54 | 15.82 |  | 0.36 | 2.30 | ↓ | 0.95 | 58.81 |  |
| SLC6A6 | solute carrier family 6 (neurotransmitter transporter, taurine), member 6 | 205920_at | 1.54 | 26.15 |  | 0.19 | 0.36 | ↓ | 0.63 | 17.70 |  |
| PURA | purine-rich element binding protein A | 204021_s_at | 1.54 | 10.25 |  | 3.18 | 0.29 | ↑ | 1.24 | 38.27 |  |
| ZMAT3 | zinc finger, matrin-type 3 | 1555609_a_at | 1.54 | 11.75 |  | 0.40 | 0.18 | ↓ | 1.63 | 2.70 |  |
| CDH10 | cadherin 10, type 2 (T2-cadherin) | 220115_s_at | 1.54 | 32.33 |  | 0.43 | 6.11 | ↓ | 1.28 | 56.10 |  |
| KCNG1 | potassium voltage-gated channel, subfamily G, member 1 | 214595_at | 1.54 | 26.15 |  | 0.69 | 47.13 |  | 0.06 | 0.00 | ↓ |
| TEAD2 | TEA domain family member 2 | 226408_at | 1.53 | 8.24 |  | 0.60 | 4.45 |  | 0.46 | 0.21 | ↓ |
| SOX4 | SRY (sex determining region Y)-box 4 | 213668_s_at | 1.53 | 24.35 |  | 2.19 | 15.95 |  | 0.29 | 0.72 | ↓ |
| ARHGEF2 | Rho/Rac guanine nucleotide exchange factor (GEF) 2 | 1554783_s_at | 1.53 | 17.32 |  | 0.29 | 3.81 | ↓ | 0.68 | 46.35 |  |
| PRKX | protein kinase, X-linked | 204061_at | 1.53 | 28.36 |  | 0.82 | 45.34 |  | 0.48 | 4.16 | ↓ |
| ARHGEF7 | Rho guanine nucleotide exchange factor (GEF) 7 | 235412_at | 1.53 | 36.49 |  | 0.46 | 6.11 | ↓ | 0.36 | 2.70 | ↓ |
| JAG1 | jagged 1 | 209098_s_at | 1.53 | 15.82 |  | 1.18 | 47.13 |  | 0.43 | 4.16 | ↓ |
| SMOX | spermine oxidase | 210357_s_at | 1.53 | 14.24 |  | 0.78 | 36.20 |  | 0.46 | 1.99 | ↓ |
| GALNT3 | UDP-N-acetyl-alpha-D-galactosamine:polypeptide N-acetylgalactosaminyltransferase 3 (GalNAc-T3) | 203397_s_at | 1.53 | 18.98 |  | 1.34 | 38.78 |  | 0.24 | 2.70 | ↓ |
| COL4A3 | collagen, type IV, alpha 3 (Goodpasture antigen) | 222073_at | 1.53 | 59.48 |  | 0.77 | 14.26 |  | 25.29 | 0.00 | ↑ |
| DCAF15 | DDB1 and CUL4 associated factor 15 | 221851_at | 1.53 | 12.80 |  | 0.17 | 0.25 | ↓ | 0.36 | 2.43 | ↓ |
| GM2A | GM2 ganglioside activator | 215891_s_at | 1.53 | 26.15 |  | 0.23 | 0.40 | ↓ | 1.21 | 46.35 |  |
| MAPRE3 | microtubule-associated protein, RP/EB family, member 3 | 214270_s_at | 1.52 | 15.82 |  | 0.23 | 0.53 | ↓ | 0.89 | 58.81 |  |
| ITCH | itchy E3 ubiquitin protein ligase homolog (mouse) | 209744_x_at | 1.52 | 8.24 |  | 2.23 | 0.60 | ↑ | 1.20 | 38.27 |  |
| CCL28 | chemokine (C-C motif) ligand 28 | 224027_at | 1.52 | 28.36 |  | 1.19 | 49.04 |  | 0.25 | 1.61 | ↓ |
| COL7A1 | collagen, type VII, alpha 1 | 217312_s_at | 1.52 | 15.82 |  | 0.82 | 45.34 |  | 0.36 | 2.70 | ↓ |
| AXL | AXL receptor tyrosine kinase | 202686_s_at | 1.52 | 22.56 |  | 3.78 | 0.36 | ↑ | 0.92 | 54.08 |  |
| ASPHD1 | aspartate beta-hydroxylase domain containing 1 | 214993_at | 1.52 | 56.74 |  | 2.00 | 6.11 | ↑ | 0.29 | 0.58 | ↓ |
| CDC42SE1 | CDC42 small effector 1 | 229120_s_at | 1.52 | 12.80 |  | 2.77 | 0.53 | ↑ | 1.16 | 43.83 |  |
| AES | amino-terminal enhancer of split | 217729_s_at | 1.52 | 20.86 |  | 0.45 | 1.74 | ↓ | 0.86 | 43.83 |  |
| RASA3 | RAS p21 protein activator 3 | 206220_s_at | 1.52 | 11.75 |  | 0.39 | 1.07 | ↓ | 0.87 | 46.35 |  |
| PSAT1 | phosphoserine aminotransferase 1 | 223062_s_at | 1.52 | 15.82 |  | 2.30 | 9.01 | ↑ | 0.51 | 20.38 |  |
| PAK2 | p21 protein (Cdc42/Rac)-activated kinase 2 | 208875_s_at | 1.52 | 14.24 |  | 0.47 | 7.87 | ↓ | 1.21 | 46.35 |  |
| PADI2 | peptidyl arginine deiminase, type II | 209791_at | 1.52 | 54.53 |  | 0.34 | 6.80 | ↓ | 1.01 | 58.81 |  |
| LOC727924 | hypothetical LOC727924 | 1564856_s_at | 1.51 | 8.24 |  | 0.48 | 4.45 | ↓ | 1.48 | 13.22 |  |
| INO80E | INO80 complex subunit E | 227286_at | 1.51 | 14.24 |  | 0.45 | 4.45 | ↓ | 0.76 | 38.27 |  |
| ZC3H12A | zinc finger CCCH-type containing 12A | 218810_at | 1.51 | 15.82 |  | 0.29 | 0.70 | ↓ | 1.95 | 4.16 |  |
| GALNT4 /// POC1B | UDP-N-acetyl-alpha-D-galactosamine:polypeptide N-acetylgalactosaminyltransferase 4 (GalNAc-T4) /// POC1 centriolar protein homolog B (Chlamydomonas) | 220442_at | 1.51 | 26.15 |  | 0.39 | 2.30 | ↓ | 0.84 | 46.35 |  |
| NCRNA00092 | non-protein coding RNA 92 | 1562733_at | 1.51 | 28.36 |  | 0.33 | 0.53 | ↓ | 1.49 | 13.22 |  |
| UBQLN4 | ubiquilin 4 | 222252_x_at | 1.51 | 24.35 |  | 2.41 | 6.11 | ↑ | 1.38 | 26.48 |  |
| NAT14 | N-acetyltransferase 14 (GCN5-related, putative) | 223284_at | 1.51 | 11.75 |  | 0.66 | 7.87 |  | 0.32 | 1.43 | ↓ |
| STK4 | serine/threonine kinase 4 | 223746_at | 1.51 | 53.22 |  | 3.65 | 9.01 | ↑ | 1.30 | 38.27 |  |
| FOXP1 | forkhead box P1 | 223936_s_at | 1.51 | 20.86 |  | 0.42 | 5.22 | ↓ | 0.83 | 50.73 |  |
| OGDH | oxoglutarate (alpha-ketoglutarate) dehydrogenase (lipoamide) | 1554152_a_at | 1.51 | 40.42 |  | 0.22 | 0.60 | ↓ | 1.45 | 20.38 |  |
| SYNPO2 | synaptopodin 2 | 244108_at | 1.51 | 32.33 |  | 0.40 | 6.80 | ↓ | 1.70 | 32.58 |  |
| OTUD5 | OTU domain containing 5 | 233933_s_at | 1.51 | 6.30 |  | 0.49 | 0.60 | ↓ | 0.97 | 54.08 |  |
| SYNPO | synaptopodin | 202796_at | 1.51 | 17.32 |  | 0.69 | 24.54 |  | 0.25 | 0.89 | ↓ |
| MTHFD2 | methylenetetrahydrofolate dehydrogenase (NADP+ dependent) 2, methenyltetrahydrofolate cyclohydrolase | 201761_at | 1.51 | 18.98 |  | 2.26 | 4.45 | ↑ | 0.69 | 17.70 |  |
| LOC389831 | hypothetical LOC389831 | 225062_at | 1.51 | 20.86 |  | 1.00 | 59.66 |  | 0.49 | 9.54 | ↓ |
| CASP2 | caspase 2, apoptosis-related cysteine peptidase | 209811_at | 1.51 | 18.98 |  | 2.77 | 1.74 | ↑ | 0.98 | 58.81 |  |
| C6orf1 | chromosome 6 open reading frame 1 | 226306_at | 1.51 | 14.24 |  | 0.49 | 1.98 | ↓ | 0.82 | 29.62 |  |
| PDLIM7 | PDZ and LIM domain 7 (enigma) | 214266_s_at | 1.50 | 26.15 |  | 0.29 | 2.80 | ↓ | 0.64 | 38.27 |  |
| EHMT2 | euchromatic histone-lysine N-methyltransferase 2 | 202326_at | 1.50 | 10.25 |  | 0.33 | 0.25 | ↓ | 0.88 | 41.22 |  |
| COL27A1 | collagen, type XXVII, alpha 1 | 225288_at | 1.50 | 26.15 |  | 1.01 | 56.87 |  | 0.36 | 1.99 | ↓ |
| EGR1 | early growth response 1 | 201694_s_at | 1.50 | 11.75 |  | 5.10 | 0.60 | ↑ | 0.92 | 41.22 |  |
| TRIM14 | tripartite motif-containing 14 | 203147_s_at | 1.50 | 20.86 |  | 2.49 | 2.30 | ↑ | 0.66 | 13.22 |  |
| ASMTL | acetylserotonin O-methyltransferase-like | 209394_at | 1.50 | 11.75 |  | 0.48 | 0.98 | ↓ | 0.76 | 17.70 |  |
| ADCY9 | adenylate cyclase 9 | 204498_s_at | 1.50 | 17.32 |  | 0.37 | 0.53 | ↓ | 1.37 | 32.58 |  |
| KDM6B | lysine (K)-specific demethylase 6B | 41386_i_at | 1.50 | 15.82 |  | 0.38 | 1.07 | ↓ | 1.01 | 57.64 |  |
| ASB1 | ankyrin repeat and SOCS box-containing 1 | 212818_s_at | 1.50 | 20.86 |  | 1.02 | 57.71 |  | 2.09 | 1.25 | ↑ |
| CDH13 | cadherin 13, H-cadherin (heart) | 204726_at | 1.50 | 28.36 |  | 2.10 | 9.01 | ↑ | 1.22 | 43.83 |  |
| C15orf17 | chromosome 15 open reading frame 17 | 224805_s_at | 1.50 | 30.22 |  | 0.39 | 0.53 | ↓ | 1.40 | 26.48 |  |
| RHBDD2 | rhomboid domain containing 2 | 232053_x_at | 1.50 | 11.75 |  | 0.47 | 1.26 | ↓ | 1.35 | 13.22 |  |
| SLC1A3 | solute carrier family 1 (glial high affinity glutamate transporter), member 3 | 1569054_at | 1.50 | 36.49 |  | 0.14 | 0.25 | ↓ | 0.31 | 1.99 | ↓ |
| SHC1 | SHC (Src homology 2 domain containing) transforming protein 1 | 201469_s_at | 1.50 | 20.86 |  | 0.17 | 0.00 | ↓ | 1.01 | 57.14 |  |
| PRRT2 | proline-rich transmembrane protein 2 | 227192_at | 1.49 | 28.36 |  | 1.22 | 36.20 |  | 0.48 | 8.02 | ↓ |
| NID1 | nidogen 1 | 202008_s_at | 1.49 | 22.56 |  | 0.49 | 1.74 | ↓ | 0.65 | 8.02 |  |
| FKBP9 | FK506 binding protein 9, 63 kDa | 212169_at | 1.49 | 5.70 |  | 2.13 | 0.70 | ↑ | 1.12 | 46.35 |  |
| C1QTNF1 | C1q and tumor necrosis factor related protein 1 | 220975_s_at | 1.49 | 22.56 |  | 0.31 | 0.36 | ↓ | 0.96 | 54.08 |  |
| PM20D2 | peptidase M20 domain containing 2 | 225421_at | 1.49 | 36.49 |  | 2.45 | 9.01 | ↑ | 0.50 | 7.00 | ↓ |
| SLC39A14 | solute carrier family 39 (zinc transporter), member 14 | 1555434_a_at | 1.49 | 20.86 |  | 0.25 | 0.18 | ↓ | 0.95 | 50.73 |  |
| FLOT2 | flotillin 2 | 211299_s_at | 1.49 | 14.24 |  | 0.24 | 0.00 | ↓ | 1.28 | 29.62 |  |
| NCRNA00115 | non-protein coding RNA 115 | 220399_at | 1.49 | 28.36 |  | 1.49 | 22.17 |  | 0.43 | 5.75 | ↓ |
| TGFBR1 | transforming growth factor, beta receptor 1 | 206943_at | 1.49 | 42.20 |  | 0.32 | 0.53 | ↓ | 2.03 | 13.22 |  |
| FN1 | fibronectin 1 | 214701_s_at | 1.49 | 34.35 |  | 0.36 | 1.74 | ↓ | 1.56 | 17.70 |  |
| RDH11 | retinol dehydrogenase 11 (all-trans/9-cis/11-cis) | 217775_s_at | 1.49 | 5.70 |  | 2.25 | 0.84 | ↑ | 0.77 | 15.27 |  |
| LATS2 | LATS, large tumor suppressor, homolog 2 (Drosophila) | 223379_s_at | 1.49 | 28.36 |  | 0.41 | 4.45 | ↓ | 1.68 | 17.70 |  |
| PHF20 | PHD finger protein 20 | 206567_s_at | 1.49 | 18.98 |  | 2.66 | 0.53 | ↑ | 0.99 | 54.08 |  |
| CAMK2G | calcium/calmodulin-dependent protein kinase II gamma | 212669_at | 1.49 | 4.93 |  | 0.42 | 0.00 | ↓ | 1.34 | 17.70 |  |
| PKM2 | pyruvate kinase, muscle | 201251_at | 1.49 | 14.24 |  | 0.15 | 0.00 | ↓ | 0.99 | 58.81 |  |
| FOXO3 /// FOXO3B | forkhead box O3 /// forkhead box O3B pseudogene | 210655_s_at | 1.48 | 12.80 |  | 0.36 | 1.07 | ↓ | 1.05 | 55.52 |  |
| LOC100128252 | hypothetical LOC100128252 | 244741_s_at | 1.48 | 43.91 |  | 1.45 | 19.82 |  | 0.46 | 2.70 | ↓ |
| SEPN1 | selenoprotein N, 1 | 224659_at | 1.48 | 12.80 |  | 0.40 | 1.26 | ↓ | 0.47 | 2.43 | ↓ |
| MARCKS | myristoylated alanine-rich protein kinase C substrate | 201670_s_at | 1.48 | 4.93 |  | 1.59 | 9.01 |  | 0.44 | 0.00 | ↓ |
| MEX3B | mex-3 homolog B (C. elegans) | 223627_at | 1.48 | 18.98 |  | 1.70 | 14.26 |  | 0.30 | 2.43 | ↓ |
| HAS3 | hyaluronan synthase 3 | 223541_at | 1.48 | 42.20 |  | 0.98 | 59.66 |  | 3.70 | 5.75 | ↑ |
| HMBOX1 | homeobox containing 1 | 219269_at | 1.48 | 22.56 |  | 2.02 | 6.80 | ↑ | 0.83 | 29.62 |  |
| HIPK1 | homeodomain interacting protein kinase 1 | 1552516_a_at | 1.48 | 14.24 |  | 0.06 | 0.00 | ↓ | 1.52 | 13.22 |  |
| SSBP1 | single-stranded DNA binding protein 1 | 214060_at | 1.48 | 40.42 |  | 1.01 | 52.07 |  | 0.49 | 4.16 | ↓ |
| PDE1C | phosphodiesterase 1C, calmodulin-dependent 70kDa | 216869_at | 1.48 | 26.15 |  | 1.34 | 29.01 |  | 2.07 | 8.02 | ↑ |
| SOD2 | superoxide dismutase 2, mitochondrial | 221477_s_at | 1.48 | 9.04 |  | 0.31 | 0.36 | ↓ | 4.68 | 0.00 | ↑ |
| SMCR8 | Smith-Magenis syndrome chromosome region, candidate 8 | 1557986_s_at | 1.47 | 20.86 |  | 0.40 | 1.45 | ↓ | 1.00 | 58.81 |  |
| BDKRB1 | bradykinin receptor B1 | 207510_at | 1.47 | 14.24 |  | 0.96 | 56.87 |  | 0.35 | 1.61 | ↓ |
| LY6E | lymphocyte antigen 6 complex, locus E | 202145_at | 1.47 | 30.22 |  | 0.20 | 0.00 | ↓ | 0.41 | 1.43 | ↓ |
| MYL9 | myosin, light chain 9, regulatory | 201058_s_at | 1.47 | 17.32 |  | 0.41 | 1.45 | ↓ | 1.00 | 57.92 |  |
| PTER | phosphotriesterase related | 222798_at | 1.47 | 20.86 |  | 2.54 | 6.80 | ↑ | 0.32 | 0.50 | ↓ |
| TMEM132A | transmembrane protein 132A | 218834_s_at | 1.47 | 22.56 |  | 0.78 | 29.01 |  | 0.47 | 1.25 | ↓ |
| CLPTM1 | cleft lip and palate associated transmembrane protein 1 | 211136_s_at | 1.47 | 14.24 |  | 0.20 | 0.00 | ↓ | 1.09 | 50.73 |  |
| MATN2 | matrilin 2 | 202350_s_at | 1.47 | 22.56 |  | 0.78 | 53.46 |  | 0.23 | 1.99 | ↓ |
| ANAPC2 | anaphase promoting complex subunit 2 | 218555_at | 1.47 | 15.82 |  | 0.43 | 1.07 | ↓ | 0.85 | 41.22 |  |
| PPARA | peroxisome proliferator-activated receptor alpha | 1558631_at | 1.47 | 12.80 |  | 0.45 | 0.84 | ↓ | 0.85 | 29.62 |  |
| SLC17A9 | solute carrier family 17, member 9 | 233328_x_at | 1.47 | 17.32 |  | 0.36 | 2.80 | ↓ | 0.42 | 4.16 | ↓ |
| KYNU | kynureninase (L-kynurenine hydrolase) | 210663_s_at | 1.47 | 57.71 |  | 0.43 | 9.01 | ↓ | 0.25 | 4.16 | ↓ |
| GCOM1 | GRINL1A complex locus | 228568_at | 1.47 | 54.53 |  | 0.70 | 36.20 |  | 2.38 | 5.75 | ↑ |
| ASXL2 | additional sex combs like 2 (Drosophila) | 1555266_a_at | 1.47 | 15.82 |  | 0.16 | 0.00 | ↓ | 1.07 | 50.73 |  |
| EGFR | epidermal growth factor receptor | 211607_x_at | 1.46 | 30.22 |  | 0.21 | 7.87 | ↓ | 1.71 | 32.58 |  |
| GLS | glutaminase | 221510_s_at | 1.46 | 9.04 |  | 2.39 | 1.45 | ↑ | 2.12 | 1.25 | ↑ |
| PAK2 | p21 protein (Cdc42/Rac)-activated kinase 2 | 1559052_s_at | 1.46 | 17.32 |  | 0.33 | 3.81 | ↓ | 0.97 | 56.10 |  |
| CPEB3 | cytoplasmic polyadenylation element binding protein 3 | 205773_at | 1.46 | 30.22 |  | 2.12 | 6.80 | ↑ | 1.00 | 58.28 |  |
| ABLIM3 | actin binding LIM protein family, member 3 | 205730_s_at | 1.46 | 24.35 |  | 1.32 | 33.65 |  | 2.79 | 2.70 | ↑ |
| GLG1 | golgi glycoprotein 1 | 225918_at | 1.46 | 24.35 |  | 2.02 | 4.45 | ↑ | 1.10 | 56.10 |  |
| ASNS | asparagine synthetase (glutamine-hydrolyzing) | 205047_s_at | 1.46 | 20.86 |  | 1.96 | 11.08 |  | 0.39 | 9.54 | ↓ |
| MYO18A /// TIAF1 | myosin XVIIIA /// TGFB1-induced anti-apoptotic factor 1 | 202039_at | 1.46 | 9.04 |  | 0.32 | 0.00 | ↓ | 1.07 | 56.65 |  |
| CRYBB2P1 | crystallin, beta B2 pseudogene 1 | 222048_at | 1.46 | 17.32 |  | 2.46 | 4.45 | ↑ | 1.48 | 4.84 |  |
| FAM26F | family with sequence similarity 26, member F | 229391_s_at | 1.46 | 40.42 |  | 1.80 | 43.50 |  | 0.36 | 2.70 | ↓ |
| POLR3G | polymerase (RNA) III (DNA directed) polypeptide G (32kD) | 206654_s_at | 1.46 | 55.60 |  | 0.48 | 4.45 | ↓ | 1.24 | 35.63 |  |
| MAPKAPK2 | mitogen-activated protein kinase-activated protein kinase 2 | 215050_x_at | 1.46 | 12.80 |  | 0.27 | 0.18 | ↓ | 0.89 | 43.83 |  |
| PDE4D | phosphodiesterase 4D, cAMP-specific | 228962_at | 1.46 | 34.35 |  | 1.04 | 58.42 |  | 0.10 | 0.00 | ↓ |
| CDC42SE1 | CDC42 small effector 1 | 218157_x_at | 1.46 | 12.80 |  | 3.29 | 0.21 | ↑ | 1.11 | 48.62 |  |
| HDLBP | high density lipoprotein binding protein | 222916_s_at | 1.46 | 15.82 |  | 0.37 | 0.25 | ↓ | 0.97 | 50.73 |  |
| AKT1S1 | AKT1 substrate 1 (proline-rich) | 224982_at | 1.46 | 15.82 |  | 0.33 | 0.53 | ↓ | 1.16 | 52.56 |  |
| IRAK1 | interleukin-1 receptor-associated kinase 1 | 1555784_s_at | 1.45 | 20.86 |  | 0.30 | 0.36 | ↓ | 1.20 | 41.22 |  |
| SFRS11 | Splicing factor, arginine/serine-rich 11 | 237746_at | 1.45 | 24.35 |  | 0.22 | 0.25 | ↓ | 1.83 | 9.54 |  |
| PIP5K1A | phosphatidylinositol-4-phosphate 5-kinase, type I, alpha | 207391_s_at | 1.45 | 24.35 |  | 1.56 | 14.26 |  | 2.77 | 4.84 | ↑ |
| MAGI1 | membrane associated guanylate kinase, WW and PDZ domain containing 1 | 225465_at | 1.45 | 18.98 |  | 3.51 | 0.00 | ↑ | 1.45 | 13.22 |  |
| BNIP3 | BCL2/adenovirus E1B 19kDa interacting protein 3 | 201848_s_at | 1.45 | 26.15 |  | 1.44 | 36.20 |  | 0.42 | 8.02 | ↓ |
| ACAP3 | ArfGAP with coiled-coil, ankyrin repeat and PH domains 3 | 225529_at | 1.45 | 20.86 |  | 0.46 | 4.45 | ↓ | 0.73 | 29.62 |  |
| RASSF8 | Ras association (RalGDS/AF-6) domain family (N-terminal) member 8 | 235996_at | 1.45 | 26.15 |  | 0.26 | 1.74 | ↓ | 2.11 | 7.00 | ↑ |
| CXCL12 | chemokine (C-X-C motif) ligand 12 | 203666_at | 1.45 | 28.36 |  | 1.02 | 58.42 |  | 0.18 | 0.00 | ↓ |
| SSC5D | scavenger receptor cysteine-rich glycoprotein | 230228_at | 1.45 | 38.54 |  | 0.45 | 0.84 | ↓ | 0.69 | 7.00 |  |
| SERPINB6 | serpin peptidase inhibitor, clade B (ovalbumin), member 6 | 1556950_s_at | 1.45 | 40.42 |  | 3.34 | 3.35 | ↑ | 0.84 | 32.58 |  |
| PCGF1 | polycomb group ring finger 1 | 210023_s_at | 1.45 | 14.24 |  | 0.19 | 0.00 | ↓ | 0.88 | 43.83 |  |
| PRAF2 | PRA1 domain family, member 2 | 203456_at | 1.45 | 14.24 |  | 0.31 | 0.00 | ↓ | 0.85 | 26.48 |  |
| GNB4 | guanine nucleotide binding protein (G protein), beta polypeptide 4 | 223488_s_at | 1.45 | 30.22 |  | 0.29 | 0.00 | ↓ | 2.14 | 0.89 | ↑ |
| INSIG1 | insulin induced gene 1 | 201625_s_at | 1.45 | 28.36 |  | 0.73 | 31.53 |  | 0.50 | 5.75 | ↓ |
| SLC1A4 | solute carrier family 1 (glutamate/neutral amino acid transporter), member 4 | 212810_s_at | 1.45 | 20.86 |  | 1.63 | 14.26 |  | 0.38 | 0.72 | ↓ |
| ZNF462 | zinc finger protein 462 | 226575_at | 1.45 | 12.80 |  | 2.67 | 0.98 | ↑ | 0.54 | 1.25 |  |
| GNA13 | guanine nucleotide binding protein (G protein), alpha 13 | 206917_at | 1.44 | 26.15 |  | 0.23 | 0.29 | ↓ | 1.02 | 58.81 |  |
| LY75 | lymphocyte antigen 75 | 205668_at | 1.44 | 62.00 |  | 1.23 | 45.34 |  | 0.28 | 2.43 | ↓ |
| ASMTL | acetylserotonin O-methyltransferase-like | 36554_at | 1.44 | 8.24 |  | 0.39 | 0.18 | ↓ | 0.95 | 46.35 |  |
| YIF1B | Yip1 interacting factor homolog B (S. cerevisiae) | 1554553_s_at | 1.44 | 12.80 |  | 0.29 | 0.00 | ↓ | 0.69 | 8.02 |  |
| SMAD3 | SMAD family member 3 | 205396_at | 1.44 | 32.33 |  | 0.14 | 0.00 | ↓ | 1.04 | 58.28 |  |
| TLE4 | transducin-like enhancer of split 4 (E(sp1) homolog, Drosophila) | 216997_x_at | 1.44 | 26.15 |  | 0.43 | 3.81 | ↓ | 0.98 | 57.92 |  |
| HFE | hemochromatosis | 210864_x_at | 1.44 | 34.35 |  | 0.49 | 1.98 | ↓ | 1.30 | 26.48 |  |
| C3orf70 | chromosome 3 open reading frame 70 | 242447_at | 1.44 | 30.22 |  | 3.13 | 1.98 | ↑ | 0.93 | 58.81 |  |
| PPFIA4 | protein tyrosine phosphatase, receptor type, f polypeptide (PTPRF), interacting protein (liprin), alpha 4 | 214978_s_at | 1.44 | 61.35 |  | 1.95 | 38.78 |  | 0.22 | 2.43 | ↓ |
| INO80B | INO80 complex subunit B | 222093_s_at | 1.44 | 8.24 |  | 0.50 | 1.74 | ↓ | 0.87 | 26.48 |  |
| DAPK3 | death-associated protein kinase 3 | 203890_s_at | 1.44 | 18.98 |  | 0.36 | 2.30 | ↓ | 0.93 | 54.08 |  |
| RPRD2 | regulation of nuclear pre-mRNA domain containing 2 | 212553_at | 1.44 | 22.56 |  | 0.50 | 2.30 | ↓ | 0.69 | 15.27 |  |
| THBS1 | thrombospondin 1 | 201110_s_at | 1.44 | 9.04 |  | 2.42 | 0.84 | ↑ | 1.53 | 4.16 |  |
| MAPKAPK2 | mitogen-activated protein kinase-activated protein kinase 2 | 201461_s_at | 1.44 | 20.86 |  | 0.16 | 0.00 | ↓ | 0.88 | 46.35 |  |
| SMARCB1 | SWI/SNF related, matrix associated, actin dependent regulator of chromatin, subfamily b, member 1 | 212167_s_at | 1.44 | 15.82 |  | 0.34 | 0.84 | ↓ | 0.82 | 43.83 |  |
| SMO | smoothened homolog (Drosophila) | 218629_at | 1.44 | 12.80 |  | 0.58 | 2.80 |  | 0.49 | 1.61 | ↓ |
| SH3GLB2 | SH3-domain GRB2-like endophilin B2 | 218813_s_at | 1.44 | 15.82 |  | 0.23 | 0.25 | ↓ | 0.71 | 23.22 |  |
| ESCO1 | establishment of cohesion 1 homolog 1 (S. cerevisiae) | 235216_at | 1.44 | 47.41 |  | 0.15 | 0.00 | ↓ | 0.80 | 32.58 |  |
| EXOC6 | exocyst complex component 6 | 226259_at | 1.44 | 20.86 |  | 2.68 | 0.76 | ↑ | 0.93 | 41.22 |  |
| SLAIN2 | SLAIN motif family, member 2 | 224853_at | 1.44 | 42.20 |  | 2.43 | 0.45 | ↑ | 0.95 | 48.62 |  |
| CTNNB1 | catenin (cadherin-associated protein), beta 1, 88kDa | 201533_at | 1.44 | 12.80 |  | 3.23 | 0.29 | ↑ | 0.94 | 50.73 |  |
| OLFML2B | olfactomedin-like 2B | 213125_at | 1.44 | 32.33 |  | 0.82 | 36.20 |  | 0.24 | 0.21 | ↓ |
| FOSL2 | FOS-like antigen 2 | 205409_at | 1.44 | 17.32 |  | 0.27 | 0.60 | ↓ | 0.62 | 20.38 |  |
| ZNF438 | zinc finger protein 438 | 244752_at | 1.43 | 34.35 |  | 0.49 | 1.98 | ↓ | 1.31 | 26.48 |  |
| KIAA1984 | KIAA1984 | 236518_at | 1.43 | 28.36 |  | 1.16 | 41.29 |  | 0.29 | 1.25 | ↓ |
| BIN1 | bridging integrator 1 | 210202_s_at | 1.43 | 18.98 |  | 0.41 | 0.84 | ↓ | 0.47 | 3.36 | ↓ |
| APOBEC3C | apolipoprotein B mRNA editing enzyme, catalytic polypeptide-like 3C | 209584_x_at | 1.43 | 9.04 |  | 0.44 | 0.00 | ↓ | 0.59 | 1.02 |  |
| USP5 | ubiquitin specific peptidase 5 (isopeptidase T) | 206031_s_at | 1.43 | 26.15 |  | 0.35 | 0.84 | ↓ | 0.87 | 48.62 |  |
| NAV3 | neuron navigator 3 | 1562234_a_at | 1.43 | 51.95 |  | 0.11 | 0.00 | ↓ | 1.84 | 13.22 |  |
| SLC29A3 | solute carrier family 29 (nucleoside transporters), member 3 | 219344_at | 1.43 | 32.33 |  | 2.34 | 6.11 | ↑ | — | — |  |
| ADORA2B | adenosine A2b receptor | 205891_at | 1.43 | 18.98 |  | 1.19 | 59.12 |  | 0.31 | 0.34 | ↓ |
| ITGA11 | integrin, alpha 11 | 1554819_a_at | 1.43 | 32.33 |  | 0.13 | 0.00 | ↓ | 0.81 | 41.22 |  |
| LMLN | leishmanolysin-like (metallopeptidase M8 family) | 1553284_s_at | 1.43 | 18.98 |  | 0.47 | 6.11 | ↓ | 0.91 | 54.08 |  |
| LOC283914 | hypothetical LOC283914 | 1561518_at | 1.43 | 26.15 |  | 0.49 | 6.11 | ↓ | 1.24 | 35.63 |  |
| PRUNE | prune homolog (Drosophila) | 210988_s_at | 1.43 | 17.32 |  | 0.44 | 0.53 | ↓ | 1.15 | 35.63 |  |
| COL6A2 | collagen, type VI, alpha 2 | 213290_at | 1.43 | 15.82 |  | 0.57 | 3.81 |  | 0.49 | 1.43 | ↓ |
| HIP1 | huntingtin interacting protein 1 | 205426_s_at | 1.43 | 45.69 |  | 0.29 | 0.70 | ↓ | 0.76 | 35.63 |  |
| LIG3 | ligase III, DNA, ATP-dependent | 207348_s_at | 1.43 | 28.36 |  | 0.40 | 0.60 | ↓ | 0.82 | 26.48 |  |
| PEX26 | peroxisomal biogenesis factor 26 | 219180_s_at | 1.43 | 9.04 |  | 2.32 | 0.70 | ↑ | 1.62 | 5.75 |  |
| FKBP1A | FK506 binding protein 1A, 12kDa | 200709_at | 1.43 | 14.24 |  | 0.32 | 0.00 | ↓ | 1.42 | 13.22 |  |
| SNAI1 | snail homolog 1 (Drosophila) | 219480_at | 1.43 | 34.35 |  | 0.51 | 6.11 |  | 0.40 | 2.43 | ↓ |
| OSMR | oncostatin M receptor | 205729_at | 1.43 | 26.15 |  | 0.41 | 0.53 | ↓ | 1.25 | 41.22 |  |
| TBRG1 | transforming growth factor beta regulator 1 | 242701_at | 1.43 | 50.55 |  | 0.55 | 6.11 |  | 0.46 | 2.70 | ↓ |
| AGTR1 | angiotensin II receptor, type 1 | 208016_s_at | 1.42 | 53.22 |  | 0.48 | 3.81 | ↓ | 0.76 | 23.22 |  |
| HFE | hemochromatosis | 211863_x_at | 1.42 | 26.15 |  | 0.41 | 0.53 | ↓ | 1.38 | 15.27 |  |
| DENND5B | DENN/MADD domain containing 5B | 215058_at | 1.42 | 24.35 |  | 2.31 | 0.76 | ↑ | 1.14 | 50.73 |  |
| PKD1P1 | polycystic kidney disease 1 (autosomal dominant) pseudogene 1 | 221501_x_at | 1.42 | 15.82 |  | 1.27 | 31.53 |  | 0.50 | 1.99 | ↓ |
| TRIP10 | thyroid hormone receptor interactor 10 | 202734_at | 1.42 | 15.82 |  | 0.33 | 0.36 | ↓ | 1.07 | 56.65 |  |
| NADK | NAD kinase | 213607_x_at | 1.42 | 12.80 |  | 0.48 | 0.70 | ↓ | 0.77 | 17.70 |  |
| CALCOCO1 | calcium binding and coiled-coil domain 1 | 209002_s_at | 1.42 | 18.98 |  | 0.27 | 0.00 | ↓ | 1.33 | 23.22 |  |
| FNDC3A | fibronectin type III domain containing 3A | 215910_s_at | 1.42 | 43.91 |  | 0.32 | 0.18 | ↓ | 1.40 | 13.22 |  |
| BCL9L | B-cell CLL/lymphoma 9-like | 227616_at | 1.42 | 20.86 |  | 0.33 | 0.25 | ↓ | 1.02 | 58.28 |  |
| FZD1 | frizzled homolog 1 (Drosophila) | 204452_s_at | 1.42 | 22.56 |  | 0.50 | 7.87 | ↓ | 1.01 | 58.28 |  |
| OGDH | oxoglutarate (alpha-ketoglutarate) dehydrogenase (lipoamide) | 201282_at | 1.42 | 22.56 |  | 0.41 | 0.70 | ↓ | 1.26 | 23.22 |  |
| C10orf58 | chromosome 10 open reading frame 58 | 228155_at | 1.42 | 34.35 |  | 1.40 | 36.20 |  | 0.26 | 3.36 | ↓ |
| ST8SIA1 | ST8 alpha-N-acetyl-neuraminide alpha-2,8-sialyltransferase 1 | 210073_at | 1.42 | 34.35 |  | 0.29 | 4.45 | ↓ | 1.22 | 41.22 |  |
| SLAIN2 | SLAIN motif family, member 2 | 224854_s_at | 1.41 | 56.74 |  | 2.79 | 0.45 | ↑ | 0.99 | 54.08 |  |
| ACVR2A | activin A receptor, type IIA | 228416_at | 1.41 | 24.35 |  | 3.01 | 5.22 | ↑ | 0.86 | 41.22 |  |
| TAOK2 | TAO kinase 2 | 204986_s_at | 1.41 | 24.35 |  | 0.44 | 0.53 | ↓ | 0.85 | 29.62 |  |
| PCDHGA1 /// PCDHGA10 /// PCDHGA11 /// PCDHGA12 /// PCDHGA2 /// PCDHGA3 /// PCDHGA4 /// PCDHGA5 /// PCDHGA6 /// PCDHGA7 /// PCDHGA8 /// PCDHGA9 /// PCDHGB1 /// PCDHGB2 /// PCDHGB3 /// PCDHGB4 /// PCDHGB5 /// PCDHGB6 /// PCDHGB7 /// PCDHGC3 /// PCDHGC4 /// PCDHGC5 | protocadherin gamma subfamily A, 1 /// protocadherin gamma subfamily A, 10 /// protocadherin gamma subfamily A, 11 /// protocadherin gamma subfamily A, 12 /// protocadherin gamma subfamily A, 2 /// protocadherin gamma subfamily A, 3 /// protocadherin gamma subfamily A, 4 /// protocadherin gamma subfamily A, 5 /// protocadherin gamma subfamily A, 6 /// protocadherin gamma subfamily A, 7 /// protocadherin gamma subfamily A, 8 /// protocadherin gamma subfamily A, 9 /// protocadherin gamma subfamily B, 1 /// protocadherin gamma subfamily B, 2 /// protocadherin gamma subfamily B, 3 /// protocadherin gamma subfamily B, 4 /// protocadherin gamma subfamily B, 5 /// protocadherin gamma subfamily B, 6 /// protocadherin gamma subfamily B, 7 /// protocadherin gamma subfamily C, 3 /// protocadherin gamma subfamily C, 4 /// protocadherin gamma subfamily C, 5 | 211066_x_at | 1.41 | 15.82 |  | 0.46 | 1.45 | ↓ | 0.96 | 54.08 |  |
| SCML1 | sex comb on midleg-like 1 (Drosophila) | 222747_s_at | 1.41 | 26.15 |  | 2.35 | 0.45 | ↑ | 1.17 | 54.08 |  |
| KCNJ12 | potassium inwardly-rectifying channel, subfamily J, member 12 | 232289_at | 1.41 | 22.56 |  | 0.79 | 58.42 |  | 2.72 | 8.02 | ↑ |
| POLG | polymerase (DNA directed), gamma | 217635_s_at | 1.41 | 61.68 |  | 0.41 | 1.74 | ↓ | 0.77 | 20.38 |  |
| LOC642869 /// SET | SET translocation (myeloid leukemia-associated) pseudogene /// SET nuclear oncogene | 215780_s_at | 1.41 | 14.24 |  | 2.14 | 2.80 | ↑ | 0.99 | 52.56 |  |
| SBF2 | SET binding factor 2 | 242935_at | 1.41 | 32.33 |  | 0.21 | 0.18 | ↓ | 1.40 | 8.02 |  |
| TBC1D17 | TBC1 domain family, member 17 | 218466_at | 1.41 | 15.82 |  | 0.45 | 1.07 | ↓ | 1.17 | 41.22 |  |
| EGR1 | early growth response 1 | 201693_s_at | 1.40 | 24.35 |  | 9.81 | 0.40 | ↑ | 0.81 | 46.35 |  |
| RTKN | rhotekin | 225150_s_at | 1.40 | 22.56 |  | 0.38 | 1.07 | ↓ | 0.66 | 9.54 |  |
| PDE4B | phosphodiesterase 4B, cAMP-specific | 211302_s_at | 1.40 | 26.15 |  | 0.31 | 0.70 | ↓ | 1.01 | 35.63 |  |
| INSR | insulin receptor | 213792_s_at | 1.40 | 42.20 |  | 2.80 | 1.98 | ↑ | 1.02 | 58.81 |  |
| RPA1 | replication protein A1, 70kDa | 236675_at | 1.40 | 20.86 |  | 2.48 | 3.35 | ↑ | 1.28 | 52.56 |  |
| DARS | aspartyl-tRNA synthetase | 201623_s_at | 1.40 | 18.98 |  | 2.94 | 0.60 | ↑ | 0.53 | 1.61 |  |
| TNFRSF19 | tumor necrosis factor receptor superfamily, member 19 | 224090_s_at | 1.40 | 48.99 |  | 0.32 | 4.45 | ↓ | 0.36 | 7.00 | ↓ |
| SORBS2 | sorbin and SH3 domain containing 2 | 225728_at | 1.40 | 20.86 |  | 1.56 | 9.01 |  | 0.49 | 1.61 | ↓ |
| LOXL2 | lysyl oxidase-like 2 | 202997_s_at | 1.40 | 34.35 |  | 0.09 | 0.00 | ↓ | 0.76 | 38.27 |  |
| KCNE4 | potassium voltage-gated channel, Isk-related family, member 4 | 1552507_at | 1.40 | 22.56 |  | 0.42 | 1.26 | ↓ | 0.68 | 15.27 |  |
| PHF17 | PHD finger protein 17 | 225820_at | 1.40 | 28.36 |  | 3.32 | 1.45 | ↑ | 2.11 | 1.61 | ↑ |
| SCIN | scinderin | 1552365_at | 1.40 | 36.49 |  | 0.24 | 31.53 |  | 5.27 | 2.43 | ↑ |
| KDM6A | lysine (K)-specific demethylase 6A | 203990_s_at | 1.40 | 20.86 |  | 0.42 | 0.76 | ↓ | 1.05 | 57.92 |  |
| DDHD1 | DDHD domain containing 1 | 243705_at | 1.40 | 34.35 |  | 0.43 | 3.81 | ↓ | 1.10 | 58.81 |  |
| NALCN | sodium leak channel, non-selective | 228608_at | 1.40 | 17.32 |  | 2.01 | 6.80 | ↑ | 1.47 | 13.22 |  |
| GLT25D2 | glycosyltransferase 25 domain containing 2 | 209883_at | 1.40 | 32.33 |  | 0.69 | 11.08 |  | 2.34 | 8.02 | ↑ |
| EPOR | erythropoietin receptor | 37986_at | 1.39 | 18.98 |  | 0.85 | 41.29 |  | 0.27 | 0.58 | ↓ |
| VAC14 | Vac14 homolog (S. cerevisiae) | 216407_at | 1.39 | 26.15 |  | 0.13 | 0.00 | ↓ | 1.07 | 56.10 |  |
| PTPN11 | protein tyrosine phosphatase, non-receptor type 11 | 205868_s_at | 1.39 | 38.54 |  | 0.37 | 1.74 | ↓ | 1.36 | 41.22 |  |
| PATZ1 | POZ (BTB) and AT hook containing zinc finger 1 | 211391_s_at | 1.39 | 12.80 |  | 0.41 | 0.53 | ↓ | 1.01 | 58.81 |  |
| MKNK2 | MAP kinase interacting serine/threonine kinase 2 | 218205_s_at | 1.39 | 24.35 |  | 2.37 | 5.22 | ↑ | 0.45 | 4.84 | ↓ |
| PPP2R4 | protein phosphatase 2A activator, regulatory subunit 4 | 208874_x_at | 1.39 | 15.82 |  | 0.44 | 1.07 | ↓ | 0.96 | 54.08 |  |
| NPIPL3 | nuclear pore complex interacting protein-like 3 | 215921_at | 1.39 | 20.86 |  | 0.27 | 1.45 | ↓ | 0.30 | 1.25 | ↓ |
| SUMO3 | SMT3 suppressor of mif two 3 homolog 3 (S. cerevisiae) | 200739_s_at | 1.39 | 15.82 |  | 0.46 | 0.60 | ↓ | 1.14 | 38.27 |  |
| MARCH8 | membrane-associated ring finger (C3HC4) 8 | 231933_at | 1.39 | 48.99 |  | 0.42 | 1.45 | ↓ | 1.42 | 23.22 |  |
| --- | --- | 241763_s_at | 1.39 | 24.35 |  | 1.52 | 29.01 |  | 2.27 | 1.99 | ↑ |
| PITPNC1 | phosphatidylinositol transfer protein, cytoplasmic 1 | 219155_at | 1.39 | 22.56 |  | 0.73 | 33.65 |  | 0.40 | 4.16 | ↓ |
| LOC402778 | CD225 family protein FLJ76511 | 1564573_at | 1.39 | 20.86 |  | 0.49 | 2.80 | ↓ | 0.83 | 35.63 |  |
| BACH1 | BTB and CNC homology 1, basic leucine zipper transcription factor 1 | 210818_s_at | 1.39 | 36.49 |  | 0.50 | 2.80 | ↓ | 1.36 | 46.35 |  |
| SLC22A15 | solute carrier family 22, member 15 | 228497_at | 1.39 | 26.15 |  | 2.29 | 6.11 | ↑ | 1.43 | 23.22 |  |
| DBN1 | drebrin 1 | 217025_s_at | 1.38 | 20.86 |  | 0.39 | 1.45 | ↓ | 0.90 | 52.56 |  |
| JAG1 | jagged 1 | 216268_s_at | 1.38 | 22.56 |  | 2.00 | 11.08 |  | 0.40 | 1.99 | ↓ |
| PTPRM | protein tyrosine phosphatase, receptor type, M | 1555578_at | 1.38 | 58.62 |  | 2.92 | 1.74 | ↑ | 2.14 | 1.99 | ↑ |
| OSBP | oxysterol binding protein | 201799_s_at | 1.38 | 24.35 |  | 0.50 | 0.60 | ↓ | 1.03 | 57.64 |  |
| WWOX | WW domain containing oxidoreductase | 223868_s_at | 1.38 | 62.00 |  | 0.41 | 0.98 | ↓ | 0.75 | 11.20 |  |
| PDK4 | pyruvate dehydrogenase kinase, isozyme 4 | 205960_at | 1.38 | 26.15 |  | 0.18 | 0.70 | ↓ | 0.55 | 32.58 |  |
| TMTC1 | transmembrane and tetratricopeptide repeat containing 1 | 226931_at | 1.38 | 28.36 |  | 0.38 | 1.98 | ↓ | 0.65 | 9.54 |  |
| GSTM4 | glutathione S-transferase mu 4 | 204149_s_at | 1.38 | 17.32 |  | 0.27 | 0.18 | ↓ | 0.81 | 26.48 |  |
| TTBK2 | tau tubulin kinase 2 | 213922_at | 1.38 | 34.35 |  | 2.98 | 1.45 | ↑ | 0.96 | 58.81 |  |
| MAP3K2 | mitogen-activated protein kinase kinase kinase 2 | 221695_s_at | 1.38 | 22.56 |  | 0.27 | 0.00 | ↓ | 0.95 | 54.08 |  |
| SVEP1 | sushi, von Willebrand factor type A, EGF and pentraxin domain containing 1 | 213247_at | 1.38 | 24.35 |  | 0.95 | 54.70 |  | 2.15 | 5.75 | ↑ |
| SEL1L3 | sel-1 suppressor of lin-12-like 3 (C. elegans) | 212311_at | 1.38 | 30.22 |  | 0.39 | 4.45 | ↓ | 0.52 | 13.22 |  |
| CASP4 | caspase 4, apoptosis-related cysteine peptidase | 213596_at | 1.38 | 40.42 |  | 1.45 | 26.77 |  | 0.39 | 5.75 | ↓ |
| SLC9A3R1 | solute carrier family 9 (sodium/hydrogen exchanger), member 3 regulator 1 | 201349_at | 1.38 | 34.35 |  | 0.46 | 1.98 | ↓ | 1.11 | 58.81 |  |
| SLC1A4 | solute carrier family 1 (glutamate/neutral amino acid transporter), member 4 | 212811_x_at | 1.38 | 20.86 |  | 1.10 | 45.34 |  | 0.39 | 2.70 | ↓ |
| STEAP3 | STEAP family member 3 | 1554830_a_at | 1.38 | 28.36 |  | 0.48 | 3.81 | ↓ | 0.83 | 38.27 |  |
| C6orf136 | chromosome 6 open reading frame 136 | 227455_at | 1.38 | 32.33 |  | 0.41 | 0.98 | ↓ | 0.79 | 29.62 |  |
| FAM129B | family with sequence similarity 129, member B | 223019_at | 1.38 | 26.15 |  | 0.43 | 0.60 | ↓ | 1.10 | 54.08 |  |
| CHST11 | carbohydrate (chondroitin 4) sulfotransferase 11 | 226372_at | 1.38 | 15.82 |  | 0.88 | 47.13 |  | 0.50 | 3.36 | ↓ |
| GPR155 | G protein-coupled receptor 155 | 239533_at | 1.38 | 61.16 |  | 6.52 | 5.22 | ↑ | 1.07 | 58.81 |  |
| NT5DC2 | 5'-nucleotidase domain containing 2 | 218051_s_at | 1.38 | 26.15 |  | 0.42 | 4.45 | ↓ | 0.58 | 17.70 |  |
| LOC729021 /// LOC729218 | hypothetical protein LOC729021 /// hypothetical LOC729218 | 231821_x_at | 1.37 | 28.36 |  | 0.50 | 3.81 | ↓ | 1.28 | 38.27 |  |
| CD40 | CD40 molecule, TNF receptor superfamily member 5 | 215346_at | 1.37 | 26.15 |  | 0.82 | 41.29 |  | 0.32 | 0.89 | ↓ |
| TRANK1 | tetratricopeptide repeat and ankyrin repeat containing 1 | 213261_at | 1.37 | 15.82 |  | 0.48 | 0.60 | ↓ | 1.04 | 58.28 |  |
| LIMK2 | LIM domain kinase 2 | 217475_s_at | 1.37 | 24.35 |  | 0.31 | 0.53 | ↓ | 1.61 | 26.48 |  |
| C3orf23 | chromosome 3 open reading frame 23 | 241666_at | 1.37 | 32.33 |  | 0.50 | 1.74 | ↓ | 1.14 | 50.73 |  |
| LOC653160 | hypothetical LOC653160 | 236246_x_at | 1.37 | 26.15 |  | 0.18 | 0.18 | ↓ | 1.39 | 17.70 |  |
| MTFP1 | mitochondrial fission process 1 | 223172_s_at | 1.37 | 45.69 |  | 0.59 | 14.26 |  | 0.22 | 0.00 | ↓ |
| C7orf30 | Chromosome 7 open reading frame 30 | 230516_at | 1.37 | 28.36 |  | 1.25 | 41.29 |  | 0.44 | 7.00 | ↓ |
| TRERF1 | transcriptional regulating factor 1 | 229016_s_at | 1.37 | 54.53 |  | 0.99 | 56.87 |  | 0.48 | 2.70 | ↓ |
| SLC4A4 | solute carrier family 4, sodium bicarbonate cotransporter, member 4 | 210738_s_at | 1.37 | 45.69 |  | 0.13 | 0.00 | ↓ | 1.11 | 56.10 |  |
| ARHGEF2 | Rho/Rac guanine nucleotide exchange factor (GEF) 2 | 209435_s_at | 1.37 | 22.56 |  | 0.91 | 59.12 |  | 0.48 | 4.84 | ↓ |
| BMP1 | bone morphogenetic protein 1 | 207595_s_at | 1.37 | 15.82 |  | 0.31 | 0.18 | ↓ | 0.87 | 38.27 |  |
| PAK2 | p21 protein (Cdc42/Rac)-activated kinase 2 | 208876_s_at | 1.37 | 20.86 |  | 0.32 | 0.45 | ↓ | 1.24 | 38.27 |  |
| TMEM136 | transmembrane protein 136 | 238497_at | 1.37 | 22.56 |  | 1.31 | 36.20 |  | 0.48 | 1.02 | ↓ |
| GGA2 | golgi-associated, gamma adaptin ear containing, ARF binding protein 2 | 214190_x_at | 1.37 | 36.49 |  | 0.37 | 1.07 | ↓ | 1.17 | 54.08 |  |
| RCN3 | reticulocalbin 3, EF-hand calcium binding domain | 219102_at | 1.36 | 18.98 |  | 0.47 | 0.53 | ↓ | 0.65 | 1.99 |  |
| NRN1 | neuritin 1 | 218625_at | 1.36 | 48.99 |  | 0.92 | 49.04 |  | 0.19 | 1.61 | ↓ |
| SNRPA | small nuclear ribonucleoprotein polypeptide A | 201770_at | 1.36 | 14.24 |  | 0.62 | 5.22 |  | 0.45 | 0.00 | ↓ |
| H2AFX | H2A histone family, member X | 212525_s_at | 1.36 | 28.36 |  | 0.24 | 0.70 | ↓ | 0.64 | 23.22 |  |
| GM2A | GM2 ganglioside activator | 33646_g_at | 1.36 | 32.33 |  | 0.31 | 0.70 | ↓ | 1.16 | 43.83 |  |
| TMEM115 | transmembrane protein 115 | 216267_s_at | 1.36 | 22.56 |  | 0.44 | 0.76 | ↓ | 0.94 | 48.62 |  |
| KLF12 | Kruppel-like factor 12 | 206966_s_at | 1.36 | 18.98 |  | 0.44 | 1.45 | ↓ | 0.91 | 50.73 |  |
| SLC25A39 | solute carrier family 25, member 39 | 223649_s_at | 1.36 | 18.98 |  | 0.48 | 0.84 | ↓ | 0.91 | 43.83 |  |
| NPIPL2 /// NPIPL3 /// PDXDC2P | nuclear pore complex interacting protein-like 2 /// nuclear pore complex interacting protein-like 3 /// pyridoxal-dependent decarboxylase domain containing 2, pseudogene | 215920_s_at | 1.36 | 26.15 |  | 0.38 | 0.18 | ↓ | 0.74 | 9.54 |  |
| LOC728855 | hypothetical LOC728855 | 229429_x_at | 1.36 | 22.56 |  | 1.36 | 22.17 |  | 0.40 | 0.34 | ↓ |
| LRRFIP1 | leucine rich repeat (in FLII) interacting protein 1 | 227391_x_at | 1.36 | 34.35 |  | 2.02 | 7.87 | ↑ | 1.46 | 13.22 |  |
| LRRC41 | leucine rich repeat containing 41 | 1555831_s_at | 1.36 | 14.24 |  | 0.29 | 0.00 | ↓ | 0.99 | 58.81 |  |
| IRX3 | iroquois homeobox 3 | 229638_at | 1.36 | 18.98 |  | 1.25 | 33.65 |  | 0.49 | 7.00 | ↓ |
| TNRC6C | trinucleotide repeat containing 6C | 222820_at | 1.36 | 14.24 |  | 1.19 | 36.20 |  | 0.47 | 1.43 | ↓ |
| SDPR | serum deprivation response | 218711_s_at | 1.36 | 59.06 |  | 0.78 | 33.65 |  | 3.88 | 0.58 | ↑ |
| DDR2 | discoidin domain receptor tyrosine kinase 2 | 205168_at | 1.36 | 20.86 |  | 0.42 | 2.80 | ↓ | 1.01 | 57.92 |  |
| GAS7 | growth arrest-specific 7 | 202191_s_at | 1.36 | 48.99 |  | 0.99 | 59.66 |  | 0.40 | 5.75 | ↓ |
| INSIG1 | insulin induced gene 1 | 201627_s_at | 1.36 | 32.33 |  | 0.94 | 59.12 |  | 0.43 | 2.70 | ↓ |
| HIST1H4H | histone cluster 1, H4h | 208180_s_at | 1.36 | 34.35 |  | 1.48 | 19.82 |  | 2.31 | 1.61 | ↑ |
| PIP5K1C | phosphatidylinositol-4-phosphate 5-kinase, type I, gamma | 212518_at | 1.35 | 28.36 |  | 0.40 | 1.26 | ↓ | 0.96 | 52.56 |  |
| NDE1 | nudE nuclear distribution gene E homolog 1 (A. nidulans) | 222625_s_at | 1.35 | 26.15 |  | 0.44 | 0.70 | ↓ | 0.55 | 1.43 |  |
| PFKFB3 | 6-phosphofructo-2-kinase/fructose-2,6-biphosphatase 3 | 202464_s_at | 1.35 | 50.55 |  | 1.52 | 41.29 |  | 0.42 | 1.61 | ↓ |
| SET | SET nuclear oncogene | 200630_x_at | 1.35 | 17.32 |  | 3.19 | 0.21 | ↑ | 1.04 | 58.81 |  |
| ATL2 | atlastin GTPase 2 | 237968_at | 1.35 | 34.35 |  | 0.43 | 2.80 | ↓ | 0.97 | 58.81 |  |
| ILK | integrin-linked kinase | 201234_at | 1.35 | 18.98 |  | 0.36 | 0.45 | ↓ | 0.81 | 32.58 |  |
| NRP2 | neuropilin 2 | 223510_at | 1.35 | 38.54 |  | 0.42 | 2.30 | ↓ | 0.83 | 43.83 |  |
| PTPN11 | protein tyrosine phosphatase, non-receptor type 11 | 205867_at | 1.35 | 32.33 |  | 0.19 | 0.45 | ↓ | 1.30 | 32.58 |  |
| G6PC3 | glucose 6 phosphatase, catalytic, 3 | 221759_at | 1.35 | 20.86 |  | 0.30 | 1.45 | ↓ | 1.04 | 57.14 |  |
| LOC100510546 /// STX5 | syntaxin-5-like /// syntaxin 5 | 203330_s_at | 1.35 | 17.32 |  | 0.40 | 0.18 | ↓ | 1.18 | 32.58 |  |
| OCRL | oculocerebrorenal syndrome of Lowe | 208316_s_at | 1.35 | 18.98 |  | 0.50 | 0.98 | ↓ | 1.26 | 29.62 |  |
| DAGLB | diacylglycerol lipase, beta | 225828_at | 1.35 | 26.15 |  | 0.41 | 0.25 | ↓ | 1.69 | 1.61 |  |
| EWSR1 /// FLI1 | Ewing sarcoma breakpoint region 1 /// Friend leukemia virus integration 1 | 211825_s_at | 1.35 | 28.36 |  | 0.23 | 0.25 | ↓ | 1.07 | 55.52 |  |
| IRF3 | interferon regulatory factor 3 | 202621_at | 1.35 | 14.24 |  | 0.43 | 0.25 | ↓ | 0.92 | 41.22 |  |
| LRRN1 | leucine rich repeat neuronal 1 | 226884_at | 1.35 | 28.36 |  | 1.61 | 29.01 |  | 0.11 | 1.02 | ↓ |
| TMEM57 | transmembrane protein 57 | 241364_at | 1.35 | 36.49 |  | 2.19 | 1.45 | ↑ | 1.00 | 58.81 |  |
| SSX2IP | synovial sarcoma, X breakpoint 2 interacting protein | 203015_s_at | 1.35 | 55.60 |  | 0.50 | 2.80 | ↓ | 0.63 | 4.16 |  |
| MKNK1 | MAP kinase interacting serine/threonine kinase 1 | 243256_at | 1.35 | 34.35 |  | 2.04 | 7.87 | ↑ | 1.22 | 43.83 |  |
| ALG3 | asparagine-linked glycosylation 3, alpha-1,3- mannosyltransferase homolog (S. cerevisiae) | 207396_s_at | 1.35 | 10.25 |  | 0.43 | 0.25 | ↓ | 0.94 | 43.83 |  |
| UBTF | upstream binding transcription factor, RNA polymerase I | 214881_s_at | 1.35 | 30.22 |  | 0.49 | 1.26 | ↓ | 0.83 | 29.62 |  |
| SSFA2 | sperm specific antigen 2 | 236207_at | 1.35 | 43.91 |  | 0.17 | 0.40 | ↓ | 1.53 | 29.62 |  |
| G6PD | glucose-6-phosphate dehydrogenase | 202275_at | 1.34 | 32.33 |  | 0.43 | 2.30 | ↓ | 0.60 | 9.54 |  |
| RASEF | RAS and EF-hand domain containing | 1553186_x_at | 1.34 | 60.94 |  | 2.75 | 1.26 | ↑ | 0.99 | 54.08 |  |
| DSTYK | dual serine/threonine and tyrosine protein kinase | 211515_s_at | 1.34 | 38.54 |  | 0.44 | 0.18 | ↓ | 1.27 | 26.48 |  |
| PPFIBP1 | PTPRF interacting protein, binding protein 1 (liprin beta 1) | 203736_s_at | 1.34 | 26.15 |  | 0.42 | 0.76 | ↓ | 0.83 | 32.58 |  |
| TBL1X | transducin (beta)-like 1X-linked | 201868_s_at | 1.34 | 26.15 |  | 0.40 | 1.98 | ↓ | 0.44 | 2.70 | ↓ |
| KDSR | 3-ketodihydrosphingosine reductase | 202419_at | 1.34 | 12.80 |  | 2.05 | 1.45 | ↑ | 1.18 | 26.48 |  |
| CTSC | cathepsin C | 225646_at | 1.34 | 30.22 |  | 0.84 | 43.50 |  | 0.49 | 3.36 | ↓ |
| FLNA | filamin A, alpha | 200859_x_at | 1.34 | 26.15 |  | 0.49 | 3.35 | ↓ | 0.75 | 23.22 |  |
| SLC12A4 | solute carrier family 12 (potassium/chloride transporters), member 4 | 209401_s_at | 1.34 | 26.15 |  | 0.21 | 0.00 | ↓ | 1.14 | 43.83 |  |
| SYTL3 | Synaptotagmin-like 3 | 242109_at | 1.34 | 57.71 |  | 0.65 | 17.74 |  | 0.47 | 2.70 | ↓ |
| PCDHGA1 /// PCDHGA10 /// PCDHGA11 /// PCDHGA12 /// PCDHGA2 /// PCDHGA3 /// PCDHGA4 /// PCDHGA5 /// PCDHGA6 /// PCDHGA7 /// PCDHGA8 /// PCDHGA9 /// PCDHGB1 /// PCDHGB2 /// PCDHGB3 /// PCDHGB4 /// PCDHGB5 /// PCDHGB6 /// PCDHGB7 /// PCDHGC3 /// PCDHGC4 /// PCDHGC5 | protocadherin gamma subfamily A, 1 /// protocadherin gamma subfamily A, 10 /// protocadherin gamma subfamily A, 11 /// protocadherin gamma subfamily A, 12 /// protocadherin gamma subfamily A, 2 /// protocadherin gamma subfamily A, 3 /// protocadherin gamma subfamily A, 4 /// protocadherin gamma subfamily A, 5 /// protocadherin gamma subfamily A, 6 /// protocadherin gamma subfamily A, 7 /// protocadherin gamma subfamily A, 8 /// protocadherin gamma subfamily A, 9 /// protocadherin gamma subfamily B, 1 /// protocadherin gamma subfamily B, 2 /// protocadherin gamma subfamily B, 3 /// protocadherin gamma subfamily B, 4 /// protocadherin gamma subfamily B, 5 /// protocadherin gamma subfamily B, 6 /// protocadherin gamma subfamily B, 7 /// protocadherin gamma subfamily C, 3 /// protocadherin gamma subfamily C, 4 /// protocadherin gamma subfamily C, 5 | 209079_x_at | 1.34 | 20.86 |  | 0.32 | 0.36 | ↓ | 0.97 | 58.81 |  |
| UBTF | upstream binding transcription factor, RNA polymerase I | 1558215_s_at | 1.34 | 30.22 |  | 0.38 | 1.74 | ↓ | 0.87 | 46.35 |  |
| BAP1 | BRCA1 associated protein-1 (ubiquitin carboxy-terminal hydrolase) | 1555735_a_at | 1.33 | 22.56 |  | 0.40 | 0.53 | ↓ | 0.86 | 38.27 |  |
| RB1 | retinoblastoma 1 | 211540_s_at | 1.33 | 40.42 |  | 0.43 | 2.30 | ↓ | 1.07 | 58.81 |  |
| ELL2 | elongation factor, RNA polymerase II, 2 | 214446_at | 1.33 | 17.32 |  | 0.37 | 0.40 | ↓ | 1.90 | 3.36 |  |
| ODF3B | outer dense fiber of sperm tails 3B | 238327_at | 1.33 | 34.35 |  | 0.73 | 24.54 |  | 0.37 | 0.72 | ↓ |
| GLYR1 | glyoxylate reductase 1 homolog (Arabidopsis) | 221628_s_at | 1.33 | 51.95 |  | 0.21 | 0.25 | ↓ | 1.37 | 29.62 |  |
| CDKN2A | cyclin-dependent kinase inhibitor 2A (melanoma, p16, inhibits CDK4) | 209644_x_at | 1.33 | 18.98 |  | 0.68 | 5.22 |  | 2.88 | 1.25 | ↑ |
| SLC39A8 | solute carrier family 39 (zinc transporter), member 8 | 219869_s_at | 1.33 | 38.54 |  | 0.64 | 15.95 |  | 6.74 | 3.36 | ↑ |
| FAM89A | family with sequence similarity 89, member A | 226448_at | 1.33 | 20.86 |  | 2.15 | 2.30 | ↑ | 1.07 | 54.08 |  |
| COL8A1 | collagen, type VIII, alpha 1 | 214587_at | 1.33 | 47.41 |  | 0.42 | 0.98 | ↓ | 1.02 | 54.08 |  |
| DRAP1 | DR1-associated protein 1 (negative cofactor 2 alpha) | 203258_at | 1.33 | 17.32 |  | 0.32 | 0.00 | ↓ | 0.84 | 23.22 |  |
| ZNF480 | zinc finger protein 480 | 222283_at | 1.33 | 38.54 |  | 0.48 | 2.30 | ↓ | 0.97 | 48.62 |  |
| ASPH | aspartate beta-hydroxylase | 209135_at | 1.33 | 20.86 |  | 1.87 | 5.22 |  | 2.19 | 2.70 | ↑ |
| STAT2 | signal transducer and activator of transcription 2, 113kDa | 205170_at | 1.33 | 42.20 |  | 0.44 | 2.30 | ↓ | 0.77 | 32.58 |  |
| PLCXD1 | phosphatidylinositol-specific phospholipase C, X domain containing 1 | 218951_s_at | 1.33 | 38.54 |  | 0.54 | 5.22 |  | 0.37 | 1.02 | ↓ |
| MYST3 | MYST histone acetyltransferase (monocytic leukemia) 3 | 216361_s_at | 1.33 | 62.00 |  | 0.73 | 24.54 |  | 0.48 | 4.84 | ↓ |
| ITGA3 | integrin, alpha 3 (antigen CD49C, alpha 3 subunit of VLA-3 receptor) | 201474_s_at | 1.33 | 47.41 |  | 0.44 | 2.80 | ↓ | 1.61 | 26.48 |  |
| CCDC74A /// CCDC74B | coiled-coil domain containing 74A /// coiled-coil domain containing 74B | 227966_s_at | 1.33 | 30.22 |  | 0.62 | 12.66 |  | 0.31 | 1.43 | ↓ |
| NFYA | nuclear transcription factor Y, alpha | 204107_at | 1.33 | 20.86 |  | 0.46 | 0.84 | ↓ | 0.63 | 7.00 |  |
| MPRIP | myosin phosphatase Rho interacting protein | 238328_at | 1.33 | 45.69 |  | 0.09 | 0.25 | ↓ | 0.56 | 26.48 |  |
| CCND1 | cyclin D1 | 208711_s_at | 1.33 | 36.49 |  | 0.84 | 38.78 |  | 2.46 | 2.43 | ↑ |
| ATAT1 | alpha tubulin acetyltransferase 1 | 228510_at | 1.33 | 32.33 |  | 1.10 | 47.13 |  | 0.36 | 2.70 | ↓ |
| HIPK3 | homeodomain interacting protein kinase 3 | 207764_s_at | 1.33 | 59.48 |  | 0.36 | 1.98 | ↓ | 1.38 | 20.38 |  |
| SCIN | scinderin | 1552367_a_at | 1.33 | 18.98 |  | — | — |  | 4.39 | 4.84 | ↑ |
| EBAG9 | estrogen receptor binding site associated, antigen, 9 | 204278_s_at | 1.32 | 22.56 |  | 2.13 | 0.53 | ↑ | 0.99 | 54.08 |  |
| SNHG10 | small nucleolar RNA host gene 10 (non-protein coding) | 238691_at | 1.32 | 57.71 |  | 0.75 | 47.13 |  | 0.44 | 9.54 | ↓ |
| FAM120A | family with sequence similarity 120A | 1555945_s_at | 1.32 | 36.49 |  | 2.50 | 0.70 | ↑ | 1.07 | 57.14 |  |
| PHACTR4 | phosphatase and actin regulator 4 | 219235_s_at | 1.32 | 20.86 |  | 2.13 | 2.30 | ↑ | 0.75 | 20.38 |  |
| NCRNA00094 | non-protein coding RNA 94 | 213788_s_at | 1.32 | 26.15 |  | 0.46 | 0.60 | ↓ | 0.80 | 20.38 |  |
| EXTL3 | exostoses (multiple)-like 3 | 211051_s_at | 1.32 | 30.22 |  | 0.42 | 1.98 | ↓ | 0.95 | 58.81 |  |
| SLC1A4 | solute carrier family 1 (glutamate/neutral amino acid transporter), member 4 | 209610_s_at | 1.32 | 28.36 |  | 1.24 | 31.53 |  | 0.37 | 2.70 | ↓ |
| SULT1A3 /// SULT1A4 | sulfotransferase family, cytosolic, 1A, phenol-preferring, member 3 /// sulfotransferase family, cytosolic, 1A, phenol-preferring, member 4 | 209607_x_at | 1.32 | 38.54 |  | 1.01 | 60.11 |  | 0.50 | 1.61 | ↓ |
| HAS2 | hyaluronan synthase 2 | 230372_at | 1.32 | 28.36 |  | 1.08 | 59.66 |  | 0.38 | 0.72 | ↓ |
| KDM4A | lysine (K)-specific demethylase 4A | 203204_s_at | 1.32 | 28.36 |  | 2.34 | 0.76 | ↑ | 1.06 | 58.81 |  |
| DENND2C | DENN/MADD domain containing 2C | 233294_at | 1.32 | 38.54 |  | 0.33 | 1.45 | ↓ | 1.16 | 48.62 |  |
| LOX | lysyl oxidase | 213640_s_at | 1.32 | 30.22 |  | 0.18 | 0.00 | ↓ | 1.10 | 54.85 |  |
| P4HA3 | prolyl 4-hydroxylase, alpha polypeptide III | 228703_at | 1.32 | 26.15 |  | 0.96 | 53.46 |  | 0.37 | 0.00 | ↓ |
| PDLIM7 | PDZ and LIM domain 7 (enigma) | 214121_x_at | 1.32 | 22.56 |  | 0.33 | 1.26 | ↓ | 0.54 | 11.20 |  |
| NPIP /// PKD1P1 | nuclear pore complex interacting protein /// polycystic kidney disease 1 (autosomal dominant) pseudogene 1 | 214870_x_at | 1.32 | 20.86 |  | 1.08 | 54.70 |  | 0.49 | 1.43 | ↓ |
| DSEL | dermatan sulfate epimerase-like | 244852_at | 1.32 | 62.00 |  | 0.22 | 0.00 | ↓ | 1.80 | 15.27 |  |
| PDLIM7 | PDZ and LIM domain 7 (enigma) | 1569149_at | 1.31 | 34.35 |  | 1.03 | 54.70 |  | 0.31 | 1.25 | ↓ |
| BCL2L1 | BCL2-like 1 | 212312_at | 1.31 | 34.35 |  | 0.49 | 1.26 | ↓ | 0.96 | 52.56 |  |
| CTNND1 | catenin (cadherin-associated protein), delta 1 | 1557944_s_at | 1.31 | 28.36 |  | 0.17 | 0.00 | ↓ | 0.94 | 58.81 |  |
| CMTM4 | CKLF-like MARVEL transmembrane domain containing 4 | 225009_at | 1.31 | 24.35 |  | 0.37 | 0.84 | ↓ | 1.38 | 26.48 |  |
| PKN1 | protein kinase N1 | 202161_at | 1.31 | 24.35 |  | 0.34 | 0.18 | ↓ | 0.75 | 20.38 |  |
| HECTD3 | HECT domain containing 3 | 218632_at | 1.31 | 17.32 |  | 0.41 | 0.36 | ↓ | 0.48 | 1.02 | ↓ |
| ZNF485 | zinc finger protein 485 | 1552427_at | 1.31 | 53.22 |  | 0.96 | 60.64 |  | 0.39 | 1.25 | ↓ |
| DAAM2 | dishevelled associated activator of morphogenesis 2 | 212793_at | 1.31 | 38.54 |  | 0.89 | 43.50 |  | 0.50 | 1.61 | ↓ |
| ULBP2 | UL16 binding protein 2 | 221291_at | 1.31 | 34.35 |  | 0.38 | 0.84 | ↓ | 0.79 | 29.62 |  |
| ADPRH | ADP-ribosylarginine hydrolase | 238505_at | 1.31 | 38.54 |  | 0.47 | 0.98 | ↓ | 0.93 | 46.35 |  |
| PHLDA1 | pleckstrin homology-like domain, family A, member 1 | 225842_at | 1.31 | 28.36 |  | 0.83 | 36.20 |  | 0.44 | 2.43 | ↓ |
| ITPR3 | inositol 1,4,5-triphosphate receptor, type 3 | 201188_s_at | 1.31 | 57.71 |  | 0.45 | 0.98 | ↓ | 0.71 | 15.27 |  |
| TMED4 | transmembrane emp24 protein transport domain containing 4 | 224680_at | 1.31 | 34.35 |  | 2.50 | 0.84 | ↑ | 1.31 | 41.22 |  |
| MYO1C | myosin IC | 214656_x_at | 1.31 | 34.35 |  | 2.69 | 1.74 | ↑ | 0.99 | 58.81 |  |
| MFAP3L | microfibrillar-associated protein 3-like | 205442_at | 1.31 | 43.91 |  | 2.48 | 2.30 | ↑ | 2.44 | 4.16 | ↑ |
| ZNF395 | zinc finger protein 395 | 218149_s_at | 1.31 | 43.91 |  | 1.25 | 49.04 |  | 0.44 | 8.02 | ↓ |
| PTPN14 | protein tyrosine phosphatase, non-receptor type 14 | 205503_at | 1.31 | 32.33 |  | 0.24 | 0.40 | ↓ | 0.86 | 54.08 |  |
| NCRNA00183 | non-protein coding RNA 183 | 1554447_at | 1.31 | 20.86 |  | 3.04 | 0.53 | ↑ | 0.89 | 38.27 |  |
| CCDC123 | coiled-coil domain containing 123 | 1563919_a_at | 1.30 | 32.33 |  | 0.45 | 0.84 | ↓ | 0.79 | 23.22 |  |
| ROCK2 | Rho-associated, coiled-coil containing protein kinase 2 | 211504_x_at | 1.30 | 26.15 |  | 0.26 | 0.00 | ↓ | 1.24 | 41.22 |  |
| TRUB1 | TruB pseudouridine (psi) synthase homolog 1 (E. coli) | 235447_at | 1.30 | 58.62 |  | 0.29 | 0.98 | ↓ | 1.45 | 29.62 |  |
| FZD3 | frizzled homolog 3 (Drosophila) | 219683_at | 1.30 | 59.80 |  | 1.23 | 59.66 |  | 0.38 | 3.36 | ↓ |
| HS3ST3B1 | Heparan sulfate (glucosamine) 3-O-sulfotransferase 3B1 | 1561908_a_at | 1.30 | 30.22 |  | 0.17 | 0.18 | ↓ | 1.56 | 23.22 |  |
| POLH | polymerase (DNA directed), eta | 1557701_s_at | 1.30 | 48.99 |  | 0.46 | 0.84 | ↓ | 1.71 | 1.43 |  |
| IL17RC | interleukin 17 receptor C | 64440_at | 1.30 | 28.36 |  | 0.33 | 0.00 | ↓ | 1.00 | 58.81 |  |
| MEX3A | mex-3 homolog A (C. elegans) | 226346_at | 1.30 | 38.54 |  | 0.75 | 26.77 |  | 0.37 | 0.58 | ↓ |
| KLF12 | Kruppel-like factor 12 | 208467_at | 1.30 | 36.49 |  | 0.34 | 1.26 | ↓ | 0.85 | 38.27 |  |
| TAB3 | TGF-beta activated kinase 1/MAP3K7 binding protein 3 | 1552927_at | 1.30 | 42.20 |  | 0.35 | 0.98 | ↓ | 1.84 | 13.22 |  |
| TXNIP | thioredoxin interacting protein | 201008_s_at | 1.30 | 32.33 |  | 0.40 | 0.98 | ↓ | 0.99 | 54.08 |  |
| TMTC4 | transmembrane and tetratricopeptide repeat containing 4 | 1554101_a_at | 1.30 | 43.91 |  | 0.79 | 31.53 |  | 0.49 | 2.43 | ↓ |
| CRIP1 | cysteine-rich protein 1 (intestinal) | 205081_at | 1.30 | 38.54 |  | 1.37 | 26.77 |  | 0.11 | 1.61 | ↓ |
| BMPR2 | bone morphogenetic protein receptor, type II (serine/threonine kinase) | 209920_at | 1.30 | 22.56 |  | 2.29 | 0.53 | ↑ | 0.76 | 11.20 |  |
| POU2F2 | POU class 2 homeobox 2 | 228343_at | 1.30 | 57.71 |  | 0.44 | 6.80 | ↓ | 1.20 | 50.73 |  |
| NIPSNAP1 | nipsnap homolog 1 (C. elegans) | 201708_s_at | 1.30 | 18.98 |  | 0.47 | 0.25 | ↓ | 0.62 | 2.70 |  |
| B4GALT1 | UDP-Gal:betaGlcNAc beta 1,4- galactosyltransferase, polypeptide 1 | 201883_s_at | 1.30 | 40.42 |  | 15.74 | 0.00 | ↑ | 1.33 | 46.35 |  |
| KIAA2026 | KIAA2026 | 235021_at | 1.30 | 60.45 |  | 2.23 | 5.22 | ↑ | 1.39 | 29.62 |  |
| HK2 | hexokinase 2 | 202934_at | 1.30 | 32.33 |  | 2.04 | 2.80 | ↑ | 0.94 | 38.27 |  |
| SLC38A5 | solute carrier family 38, member 5 | 234973_at | 1.30 | 54.53 |  | 0.55 | 10.02 |  | 0.46 | 2.43 | ↓ |
| PTCH1 | patched 1 | 209815_at | 1.30 | 34.35 |  | 1.77 | 11.08 |  | 0.45 | 2.43 | ↓ |
| KIAA0748 | KIAA0748 | 219724_s_at | 1.30 | 58.62 |  | 0.56 | 10.02 |  | 0.49 | 7.00 | ↓ |
| P4HB | prolyl 4-hydroxylase, beta polypeptide | 200656_s_at | 1.30 | 24.35 |  | 0.50 | 0.40 | ↓ | 0.73 | 4.84 |  |
| C1orf110 | chromosome 1 open reading frame 110 | 1554960_at | 1.29 | 59.06 |  | 1.27 | 45.34 |  | 4.14 | 3.36 | ↑ |
| PTPN21 | protein tyrosine phosphatase, non-receptor type 21 | 1320_at | 1.29 | 34.35 |  | 0.46 | 1.98 | ↓ | 1.16 | 48.62 |  |
| MAMDC2 | MAM domain containing 2 | 228885_at | 1.29 | 45.69 |  | 0.48 | 9.01 | ↓ | 2.03 | 54.08 |  |
| HDLBP | high density lipoprotein binding protein | 200643_at | 1.29 | 20.86 |  | 0.49 | 0.84 | ↓ | 0.90 | 35.63 |  |
| GNG7 | guanine nucleotide binding protein (G protein), gamma 7 | 228831_s_at | 1.29 | 42.20 |  | 1.59 | 15.95 |  | 0.50 | 4.16 | ↓ |
| GMDS | GDP-mannose 4,6-dehydratase | 204875_s_at | 1.29 | 36.49 |  | 0.42 | 1.26 | ↓ | 0.46 | 1.25 | ↓ |
| PEX19 | peroxisomal biogenesis factor 19 | 201706_s_at | 1.29 | 17.32 |  | 0.36 | 0.00 | ↓ | 1.18 | 26.48 |  |
| SYNE1 | spectrin repeat containing, nuclear envelope 1 | 232027_at | 1.29 | 26.15 |  | 1.34 | 36.20 |  | 2.47 | 1.61 | ↑ |
| RANGAP1 | Ran GTPase activating protein 1 | 212125_at | 1.29 | 40.42 |  | 0.25 | 0.00 | ↓ | 1.28 | 38.27 |  |
| CCDC68 | coiled-coil domain containing 68 | 220180_at | 1.29 | 48.99 |  | 0.98 | 60.31 |  | 0.40 | 8.02 | ↓ |
| CBFB | core-binding factor, beta subunit | 206788_s_at | 1.29 | 38.54 |  | 0.35 | 0.45 | ↓ | 1.01 | 58.68 |  |
| CXCL16 | chemokine (C-X-C motif) ligand 16 | 223454_at | 1.29 | 26.15 |  | 0.81 | 29.01 |  | 0.50 | 1.61 | ↓ |
| PARP6 | poly (ADP-ribose) polymerase family, member 6 | 234710_s_at | 1.28 | 26.15 |  | 0.47 | 0.60 | ↓ | 0.84 | 23.22 |  |
| SBNO2 | strawberry notch homolog 2 (Drosophila) | 215760_s_at | 1.28 | 30.22 |  | 0.11 | 0.00 | ↓ | 0.98 | 54.08 |  |
| GAB1 | GRB2-associated binding protein 1 | 207112_s_at | 1.28 | 50.55 |  | 0.46 | 1.26 | ↓ | 0.85 | 35.63 |  |
| PXDN | peroxidasin homolog (Drosophila) | 212013_at | 1.28 | 26.15 |  | 5.01 | 0.00 | ↑ | 0.73 | 17.70 |  |
| HIST1H4C | histone cluster 1, H4c | 205967_at | 1.28 | 47.41 |  | 7.23 | 1.74 | ↑ | 0.70 | 9.54 |  |
| RPL31 /// TBC1D8 | ribosomal protein L31 /// TBC1 domain family, member 8 (with GRAM domain) | 241017_at | 1.28 | 58.62 |  | 1.01 | 56.87 |  | 0.42 | 0.50 | ↓ |
| GANAB | glucosidase, alpha; neutral AB | 211934_x_at | 1.28 | 28.36 |  | 0.47 | 0.70 | ↓ | 0.56 | 0.72 |  |
| ASAM | adipocyte-specific adhesion molecule | 228082_at | 1.28 | 28.36 |  | 0.40 | 0.76 | ↓ | 1.17 | 41.22 |  |
| CAPZB | capping protein (actin filament) muscle Z-line, beta | 201950_x_at | 1.28 | 20.86 |  | 0.49 | 0.53 | ↓ | 0.81 | 15.27 |  |
| SPOP | speckle-type POZ protein | 208927_at | 1.28 | 30.22 |  | 2.40 | 0.98 | ↑ | 0.81 | 29.62 |  |
| DHX35 | DEAH (Asp-Glu-Ala-His) box polypeptide 35 | 234728_s_at | 1.28 | 50.55 |  | 0.23 | 0.00 | ↓ | 1.15 | 43.83 |  |
| MYCL1 | v-myc myelocytomatosis viral oncogene homolog 1, lung carcinoma derived (avian) | 214058_at | 1.28 | 61.79 |  | 0.36 | 1.26 | ↓ | 1.32 | 11.20 |  |
| SNX27 | sorting nexin family member 27 | 221006_s_at | 1.28 | 30.22 |  | 0.34 | 0.76 | ↓ | 1.09 | 52.56 |  |
| EHD3 | EH-domain containing 3 | 218935_at | 1.28 | 34.35 |  | 0.91 | 45.34 |  | 0.50 | 0.34 | ↓ |
| CYTH3 | cytohesin 3 | 243752_s_at | 1.28 | 62.00 |  | 0.41 | 1.45 | ↓ | 1.07 | 54.85 |  |
| AKT1 | v-akt murine thymoma viral oncogene homolog 1 | 207163_s_at | 1.28 | 20.86 |  | 0.43 | 0.25 | ↓ | 0.60 | 2.70 |  |
| C2orf49 | chromosome 2 open reading frame 49 | 243093_at | 1.27 | 50.55 |  | 0.29 | 0.00 | ↓ | 0.97 | 54.08 |  |
| HGSNAT | heparan-alpha-glucosaminide N-acetyltransferase | 222491_at | 1.27 | 40.42 |  | 0.47 | 2.30 | ↓ | 1.41 | 13.22 |  |
| GFM2 | G elongation factor, mitochondrial 2 | 231917_at | 1.27 | 50.55 |  | 0.48 | 0.76 | ↓ | 0.90 | 41.22 |  |
| TRPC4 | transient receptor potential cation channel, subfamily C, member 4 | 224220_x_at | 1.27 | 30.22 |  | 0.78 | 41.29 |  | 3.05 | 0.89 | ↑ |
| HAS2 | hyaluronan synthase 2 | 206432_at | 1.27 | 34.35 |  | 1.10 | 60.31 |  | 0.42 | 0.34 | ↓ |
| CITED2 | Cbp/p300-interacting transactivator, with Glu/Asp-rich carboxy-terminal domain, 2 | 207980_s_at | 1.27 | 34.35 |  | 0.33 | 0.45 | ↓ | 1.26 | 32.58 |  |
| TNFRSF21 | tumor necrosis factor receptor superfamily, member 21 | 214581_x_at | 1.27 | 55.60 |  | 0.58 | 29.01 |  | 6.56 | 0.58 | ↑ |
| CCDC124 | coiled-coil domain containing 124 | 225454_at | 1.27 | 26.15 |  | 0.41 | 0.40 | ↓ | 0.90 | 41.22 |  |
| TP53 | tumor protein p53 | 201746_at | 1.27 | 32.33 |  | 0.44 | 9.01 | ↓ | 0.73 | 41.22 |  |
| FCHO2 | FCH domain only 2 | 1555153_s_at | 1.27 | 55.60 |  | 0.37 | 0.84 | ↓ | 1.14 | 54.08 |  |
| PHTF2 | putative homeodomain transcription factor 2 | 1554780_a_at | 1.27 | 32.33 |  | 0.43 | 0.45 | ↓ | 1.73 | 3.36 |  |
| BAT3 | HLA-B associated transcript 3 | 213318_s_at | 1.27 | 24.35 |  | 0.50 | 0.98 | ↓ | 0.76 | 17.70 |  |
| MDK | midkine (neurite growth-promoting factor 2) | 209035_at | 1.27 | 45.69 |  | 0.39 | 1.26 | ↓ | 0.31 | 0.00 | ↓ |
| PIK3IP1 | phosphoinositide-3-kinase interacting protein 1 | 221757_at | 1.27 | 59.80 |  | 0.55 | 5.22 |  | 3.00 | 0.00 | ↑ |
| YIF1B | Yip1 interacting factor homolog B (S. cerevisiae) | 226437_at | 1.27 | 14.24 |  | 0.46 | 0.00 | ↓ | 0.76 | 11.20 |  |
| ZNF37A /// ZNF37BP | zinc finger protein 37A /// zinc finger protein 37B, pseudogene | 214878_at | 1.27 | 58.62 |  | 2.54 | 1.74 | ↑ | 1.27 | 38.27 |  |
| KPNA4 | karyopherin alpha 4 (importin alpha 3) | 209653_at | 1.27 | 43.91 |  | 0.31 | 0.98 | ↓ | 1.08 | 50.73 |  |
| KLHDC3 | kelch domain containing 3 | 208784_s_at | 1.27 | 36.49 |  | 0.29 | 0.00 | ↓ | 1.28 | 23.22 |  |
| C5orf4 | chromosome 5 open reading frame 4 | 48030_i_at | 1.27 | 45.69 |  | 0.38 | 0.40 | ↓ | 1.97 | 1.25 |  |
| FAM115A | family with sequence similarity 115, member A | 210529_s_at | 1.27 | 32.33 |  | 0.49 | 0.84 | ↓ | 0.98 | 54.08 |  |
| PINK1 | PTEN induced putative kinase 1 | 209019_s_at | 1.27 | 36.49 |  | 0.83 | 31.53 |  | 2.45 | 1.43 | ↑ |
| PRRX1 | paired related homeobox 1 | 205991_s_at | 1.27 | 38.54 |  | 0.28 | 0.18 | ↓ | 1.10 | 56.10 |  |
| SLIT2 | slit homolog 2 (Drosophila) | 209897_s_at | 1.27 | 34.35 |  | 0.71 | 41.29 |  | 0.42 | 4.84 | ↓ |
| PCBD1 | pterin-4 alpha-carbinolamine dehydratase/dimerization cofactor of hepatocyte nuclear factor 1 alpha | 203557_s_at | 1.27 | 20.86 |  | 0.47 | 0.70 | ↓ | 0.98 | 48.62 |  |
| DDAH1 | Dimethylarginine dimethylaminohydrolase 1 | 229456_s_at | 1.27 | 45.69 |  | 0.45 | 1.74 | ↓ | 1.73 | 8.02 |  |
| PIK3C2A | phosphoinositide-3-kinase, class 2, alpha polypeptide | 1569022_a_at | 1.26 | 42.20 |  | 0.50 | 5.22 |  | 2.49 | 4.16 | ↑ |
| MBNL1 | muscleblind-like (Drosophila) | 1555594_a_at | 1.26 | 60.72 |  | 0.16 | 0.00 | ↓ | 1.15 | 43.83 |  |
| TAP2 | transporter 2, ATP-binding cassette, sub-family B (MDR/TAP) | 204770_at | 1.26 | 59.48 |  | 0.29 | 0.25 | ↓ | 1.53 | 43.83 |  |
| ENTPD4 | ectonucleoside triphosphate diphosphohydrolase 4 | 1555358_a_at | 1.26 | 45.69 |  | 0.42 | 2.80 | ↓ | 1.54 | 13.22 |  |
| CDK8 | cyclin-dependent kinase 8 | 1553112_s_at | 1.26 | 38.54 |  | 0.49 | 0.98 | ↓ | 0.89 | 38.27 |  |
| SLC17A9 | solute carrier family 17, member 9 | 232922_s_at | 1.26 | 28.36 |  | 0.30 | 0.25 | ↓ | 0.51 | 1.43 |  |
| NRSN2 | neurensin 2 | 218359_at | 1.26 | 34.35 |  | 0.33 | 0.60 | ↓ | 0.99 | 58.51 |  |
| CIZ1 | CDKN1A interacting zinc finger protein 1 | 211358_s_at | 1.26 | 38.54 |  | 0.33 | 0.18 | ↓ | 0.74 | 20.38 |  |
| SFXN3 | Sideroflexin 3 | 1559993_at | 1.26 | 51.95 |  | 3.05 | 3.35 | ↑ | 0.38 | 1.99 | ↓ |
| FOXP1 | forkhead box P1 | 1558996_at | 1.26 | 34.35 |  | 0.81 | 43.50 |  | 0.42 | 5.75 | ↓ |
| RASEF | RAS and EF-hand domain containing | 1553185_at | 1.26 | 62.00 |  | 3.26 | 2.30 | ↑ | 1.08 | 55.52 |  |
| LAPTM4B | lysosomal protein transmembrane 4 beta | 208767_s_at | 1.26 | 32.33 |  | 0.48 | 1.07 | ↓ | 0.85 | 29.62 |  |
| HFE | hemochromatosis | 211327_x_at | 1.26 | 60.18 |  | 0.45 | 0.60 | ↓ | 0.93 | 43.83 |  |
| SULT1A1 | sulfotransferase family, cytosolic, 1A, phenol-preferring, member 1 | 215299_x_at | 1.26 | 38.54 |  | 2.11 | 9.01 | ↑ | 0.32 | 1.43 | ↓ |
| LOC100132247 /// LOC348162 /// LOC613037 /// LOC728888 /// NPIPL3 | similar to Uncharacterized protein KIAA0220 /// hypothetical protein 348162 /// nuclear pore complex interacting protein pseudogene /// nuclear pore complex-interacting protein-like 3-like /// nuclear pore complex interacting protein-like 3 | 211996_s_at | 1.26 | 36.49 |  | 2.95 | 0.76 | ↑ | 0.73 | 13.22 |  |
| COL18A1 | collagen, type XVIII, alpha 1 | 209082_s_at | 1.26 | 57.71 |  | 0.89 | 50.64 |  | 0.49 | 3.36 | ↓ |
| PLEKHG2 | pleckstrin homology domain containing, family G (with RhoGef domain) member 2 | 225979_at | 1.25 | 48.99 |  | 0.79 | 31.53 |  | 0.49 | 4.84 | ↓ |
| PAPD5 | PAP associated domain containing 5 | 226843_s_at | 1.25 | 22.56 |  | 2.36 | 0.29 | ↑ | 0.98 | 50.73 |  |
| VDR | vitamin D (1,25- dihydroxyvitamin D3) receptor | 204255_s_at | 1.25 | 38.54 |  | 2.36 | 2.80 | ↑ | 1.10 | 50.73 |  |
| LMAN1 | lectin, mannose-binding, 1 | 203294_s_at | 1.25 | 53.22 |  | 0.09 | 0.00 | ↓ | 0.99 | 54.08 |  |
| FRYL | FRY-like | 1563687_a_at | 1.25 | 58.62 |  | 0.35 | 0.36 | ↓ | 1.09 | 57.14 |  |
| SYNJ2 | synaptojanin 2 | 1555009_a_at | 1.25 | 36.49 |  | 0.49 | 1.74 | ↓ | 1.16 | 43.83 |  |
| AKAP1 | A kinase (PRKA) anchor protein 1 | 210625_s_at | 1.25 | 32.33 |  | 0.45 | 3.81 | ↓ | 0.78 | 17.70 |  |
| CPNE2 | copine II | 225129_at | 1.25 | 32.33 |  | 1.11 | 45.34 |  | 0.36 | 0.58 | ↓ |
| PDE1C | phosphodiesterase 1C, calmodulin-dependent 70kDa | 1554378_a_at | 1.25 | 62.00 |  | 0.68 | 24.54 |  | 2.27 | 9.54 | ↑ |
| JAK2 | Janus kinase 2 | 1562031_at | 1.25 | 59.06 |  | 0.62 | 17.74 |  | 0.44 | 2.43 | ↓ |
| MLF2 | myeloid leukemia factor 2 | 200948_at | 1.25 | 30.22 |  | 0.24 | 0.00 | ↓ | 0.82 | 26.48 |  |
| SALL1 | sal-like 1 (Drosophila) | 206893_at | 1.25 | 43.91 |  | 0.89 | 49.04 |  | 0.29 | 3.36 | ↓ |
| AEBP1 | AE binding protein 1 | 201792_at | 1.25 | 38.54 |  | 0.84 | 47.13 |  | 0.46 | 5.75 | ↓ |
| FGF5 | fibroblast growth factor 5 | 210311_at | 1.25 | 40.42 |  | 0.41 | 1.45 | ↓ | 0.91 | 46.35 |  |
| ATP6V0E1 | ATPase, H+ transporting, lysosomal 9kDa, V0 subunit e1 | 214149_s_at | 1.25 | 32.33 |  | 5.18 | 0.00 | ↑ | 1.77 | 5.75 |  |
| BDNFOS | BDNF opposite strand (non-protein coding) | 1567359_at | 1.25 | 62.00 |  | 0.29 | 1.07 | ↓ | 1.07 | 58.81 |  |
| MGEA5 | Meningioma expressed antigen 5 (hyaluronidase) | 214972_at | 1.25 | 60.18 |  | 0.44 | 1.45 | ↓ | 0.81 | 29.62 |  |
| WISP1 | WNT1 inducible signaling pathway protein 1 | 211312_s_at | 1.24 | 54.53 |  | 0.17 | 3.35 | ↓ | 0.45 | 38.27 |  |
| F7 | coagulation factor VII (serum prothrombin conversion accelerator) | 207300_s_at | 1.24 | 36.49 |  | 0.45 | 1.98 | ↓ | 1.32 | 48.62 |  |
| GRK6 | G protein-coupled receptor kinase 6 | 211543_s_at | 1.24 | 62.00 |  | 0.37 | 0.29 | ↓ | 0.75 | 17.70 |  |
| ABCB6 | ATP-binding cassette, sub-family B (MDR/TAP), member 6 | 203192_at | 1.24 | 55.60 |  | 0.86 | 36.20 |  | 0.48 | 2.43 | ↓ |
| KLHL22 | kelch-like 22 (Drosophila) | 221837_at | 1.24 | 42.20 |  | 0.31 | 0.00 | ↓ | 0.85 | 26.48 |  |
| PLEKHA2 | pleckstrin homology domain containing, family A (phosphoinositide binding specific) member 2 | 238013_at | 1.24 | 42.20 |  | 0.32 | 0.36 | ↓ | 0.62 | 8.02 |  |
| EI24 | etoposide induced 2.4 mRNA | 216396_s_at | 1.24 | 32.33 |  | 2.16 | 1.26 | ↑ | 1.00 | 58.81 |  |
| MAF1 | MAF1 homolog (S. cerevisiae) | 222998_at | 1.24 | 30.22 |  | 0.30 | 0.00 | ↓ | 0.75 | 13.22 |  |
| MBD1 | methyl-CpG binding domain protein 1 | 1555611_s_at | 1.24 | 40.42 |  | 0.19 | 0.00 | ↓ | 1.04 | 56.10 |  |
| UBA1 | ubiquitin-like modifier activating enzyme 1 | 200964_at | 1.24 | 34.35 |  | 0.23 | 0.00 | ↓ | 0.73 | 15.27 |  |
| RNF144B | ring finger protein 144B | 228153_at | 1.24 | 34.35 |  | 1.47 | 47.13 |  | 3.79 | 0.72 | ↑ |
| SMARCA4 | SWI/SNF related, matrix associated, actin dependent regulator of chromatin, subfamily a, member 4 | 208793_x_at | 1.24 | 30.22 |  | 0.37 | 0.25 | ↓ | 0.71 | 5.75 |  |
| PALLD | palladin, cytoskeletal associated protein | 200906_s_at | 1.24 | 48.99 |  | 3.53 | 0.21 | ↑ | 1.27 | 38.27 |  |
| SRPK2 | SRSF protein kinase 2 | 214931_s_at | 1.24 | 36.49 |  | 0.49 | 1.45 | ↓ | 1.08 | 55.52 |  |
| LRSAM1 | leucine rich repeat and sterile alpha motif containing 1 | 235449_at | 1.24 | 34.35 |  | 0.14 | 0.00 | ↓ | 0.69 | 8.02 |  |
| ZNF587 | zinc finger protein 587 | 1558251_a_at | 1.24 | 43.91 |  | 4.57 | 0.00 | ↑ | 1.05 | 57.92 |  |
| FAM73A | family with sequence similarity 73, member A | 235125_x_at | 1.24 | 28.36 |  | 2.08 | 1.98 | ↑ | 1.41 | 17.70 |  |
| WAC | WW domain containing adaptor with coiled-coil | 219679_s_at | 1.24 | 45.69 |  | 0.38 | 0.25 | ↓ | 0.95 | 50.73 |  |
| AP2M1 | adaptor-related protein complex 2, mu 1 subunit | 200613_at | 1.24 | 24.35 |  | 0.39 | 0.00 | ↓ | 1.08 | 50.73 |  |
| RNF24 | ring finger protein 24 | 204669_s_at | 1.24 | 36.49 |  | 2.70 | 3.81 | ↑ | 0.85 | 52.56 |  |
| PILRB | paired immunoglobin-like type 2 receptor beta | 220954_s_at | 1.24 | 56.74 |  | 0.53 | 2.80 |  | 0.48 | 7.00 | ↓ |
| REEP3 | receptor accessory protein 3 | 235016_at | 1.23 | 55.60 |  | 0.31 | 0.45 | ↓ | 1.37 | 38.27 |  |
| ADD1 | adducin 1 (alpha) | 214726_x_at | 1.23 | 36.49 |  | 0.28 | 0.18 | ↓ | 1.12 | 46.35 |  |
| ANTXR1 | anthrax toxin receptor 1 | 220092_s_at | 1.23 | 40.42 |  | 0.66 | 12.66 |  | 2.35 | 8.02 | ↑ |
| IL6ST | interleukin 6 signal transducer (gp130, oncostatin M receptor) | 204864_s_at | 1.23 | 62.00 |  | 0.26 | 0.25 | ↓ | 2.14 | 8.02 | ↑ |
| CLDN23 | claudin 23 | 228707_at | 1.23 | 45.69 |  | 1.08 | 58.42 |  | 0.42 | 8.02 | ↓ |
| EP300 | E1A binding protein p300 | 213579_s_at | 1.23 | 26.15 |  | 3.58 | 0.00 | ↑ | 1.09 | 50.73 |  |
| ENG | endoglin | 201808_s_at | 1.23 | 58.62 |  | 0.26 | 0.25 | ↓ | 0.89 | 46.35 |  |
| STAT6 | signal transducer and activator of transcription 6, interleukin-4 induced | 201332_s_at | 1.23 | 40.42 |  | 0.31 | 0.40 | ↓ | 0.99 | 58.81 |  |
| JAK1 | Janus kinase 1 | 1552611_a_at | 1.23 | 38.54 |  | 3.90 | 0.00 | ↑ | 1.68 | 1.61 |  |
| AQR | Aquarius homolog (mouse) | 239895_at | 1.23 | 55.60 |  | 0.49 | 7.87 | ↓ | 0.95 | 43.83 |  |
| CYBASC3 | cytochrome b, ascorbate dependent 3 | 224735_at | 1.23 | 47.41 |  | 0.74 | 26.77 |  | 2.29 | 1.99 | ↑ |
| CEP152 | centrosomal protein 152kDa | 215170_s_at | 1.23 | 62.00 |  | 1.14 | 38.78 |  | 0.46 | 7.00 | ↓ |
| TMEM128 | transmembrane protein 128 | 225462_at | 1.23 | 34.35 |  | 2.12 | 1.45 | ↑ | 1.25 | 38.27 |  |
| GPR176 | G protein-coupled receptor 176 | 206673_at | 1.23 | 36.49 |  | 0.24 | 0.18 | ↓ | 2.12 | 1.99 | ↑ |
| RC3H2 | ring finger and CCCH-type domains 2 | 238421_at | 1.23 | 48.99 |  | 0.34 | 3.81 | ↓ | 1.18 | 52.56 |  |
| PADI2 | peptidyl arginine deiminase, type II | 1554384_at | 1.23 | 45.69 |  | 0.37 | 6.11 | ↓ | 0.39 | 9.54 | ↓ |
| ITPRIPL2 | inositol 1,4,5-triphosphate receptor interacting protein-like 2 | 227954_at | 1.22 | 47.41 |  | 2.13 | 2.30 | ↑ | 2.12 | 1.25 | ↑ |
| EIF4G3 | eukaryotic translation initiation factor 4 gamma, 3 | 1554310_a_at | 1.22 | 48.99 |  | 0.49 | 6.11 | ↓ | 1.46 | 17.70 |  |
| ATP6V1G2 /// BAT1 | ATPase, H+ transporting, lysosomal 13kDa, V1 subunit G2 /// HLA-B associated transcript 1 | 200041_s_at | 1.22 | 34.35 |  | 0.30 | 0.25 | ↓ | 0.58 | 5.75 |  |
| SLC39A9 | solute carrier family 39 (zinc transporter), member 9 | 217859_s_at | 1.22 | 59.06 |  | 0.40 | 1.07 | ↓ | 1.54 | 15.27 |  |
| SPIRE1 | spire homolog 1 (Drosophila) | 1559517_a_at | 1.22 | 48.99 |  | 0.47 | 1.26 | ↓ | 1.13 | 48.62 |  |
| ALKBH5 | alkB, alkylation repair homolog 5 (E. coli) | 228034_x_at | 1.22 | 43.91 |  | 0.48 | 4.45 | ↓ | 0.85 | 43.83 |  |
| C5orf53 | chromosome 5 open reading frame 53 | 241874_at | 1.22 | 38.54 |  | 0.36 | 0.53 | ↓ | 1.58 | 7.00 |  |
| NRG1 | neuregulin 1 | 206343_s_at | 1.22 | 56.74 |  | 1.08 | 56.87 |  | 0.41 | 5.75 | ↓ |
| ITFG1 | Integrin alpha FG-GAP repeat containing 1 | 1556151_at | 1.22 | 36.49 |  | 1.97 | 3.35 |  | 2.21 | 1.99 | ↑ |
| GPR116 | G protein-coupled receptor 116 | 212950_at | 1.22 | 60.72 |  | 6.67 | 43.50 |  | 4.86 | 1.25 | ↑ |
| TRNP1 | TMF1-regulated nuclear protein 1 | 227862_at | 1.22 | 38.54 |  | 1.99 | 5.22 |  | 3.15 | 0.58 | ↑ |
| ITGA5 | integrin, alpha 5 (fibronectin receptor, alpha polypeptide) | 201389_at | 1.22 | 40.42 |  | 0.39 | 0.53 | ↓ | 0.37 | 0.34 | ↓ |
| TSPAN2 | tetraspanin 2 | 227233_at | 1.22 | 55.60 |  | 2.28 | 6.11 | ↑ | 0.41 | 1.25 | ↓ |
| RSF1 | remodeling and spacing factor 1 | 222540_s_at | 1.22 | 32.33 |  | 2.78 | 0.14 | ↑ | 1.20 | 38.27 |  |
| MAPK14 | mitogen-activated protein kinase 14 | 210449_x_at | 1.22 | 40.42 |  | 0.49 | 0.98 | ↓ | 1.27 | 26.48 |  |
| PYCR1 | pyrroline-5-carboxylate reductase 1 | 202148_s_at | 1.22 | 48.99 |  | 0.76 | 26.77 |  | 0.49 | 1.43 | ↓ |
| RASA2 | RAS p21 protein activator 2 | 206636_at | 1.22 | 42.20 |  | 0.39 | 0.60 | ↓ | 1.81 | 2.43 |  |
| TOMM22 | translocase of outer mitochondrial membrane 22 homolog (yeast) | 222474_s_at | 1.22 | 36.49 |  | 2.06 | 1.45 | ↑ | 0.70 | 8.02 |  |
| SMARCA1 | SWI/SNF related, matrix associated, actin dependent regulator of chromatin, subfamily a, member 1 | 203873_at | 1.22 | 38.54 |  | 0.36 | 1.07 | ↓ | 1.42 | 46.35 |  |
| TPD52 | tumor protein D52 | 201690_s_at | 1.22 | 50.55 |  | 2.26 | 33.65 |  | 0.46 | 8.02 | ↓ |
| LOC401074 | hypothetical LOC401074 | 1559826_a_at | 1.22 | 57.71 |  | 0.83 | 43.50 |  | 2.41 | 9.54 | ↑ |
| HOXC6 | homeobox C6 | 206858_s_at | 1.22 | 40.42 |  | 2.02 | 2.30 | ↑ | 0.91 | 38.27 |  |
| MPP4 | membrane protein, palmitoylated 4 (MAGUK p55 subfamily member 4) | 1552489_s_at | 1.22 | 54.53 |  | 0.86 | 45.34 |  | 0.45 | 1.43 | ↓ |
| LMAN1 | lectin, mannose-binding, 1 | 203293_s_at | 1.22 | 60.45 |  | 0.15 | 0.00 | ↓ | 1.07 | 58.81 |  |
| LIMA1 | LIM domain and actin binding 1 | 222457_s_at | 1.22 | 36.49 |  | 3.35 | 0.00 | ↑ | 0.80 | 13.22 |  |
| HTRA4 | HtrA serine peptidase 4 | 1553706_at | 1.21 | 42.20 |  | 0.31 | 3.35 | ↓ | 0.57 | 20.38 |  |
| AP1M1 | adaptor-related protein complex 1, mu 1 subunit | 223025_s_at | 1.21 | 36.49 |  | 0.28 | 0.00 | ↓ | 0.86 | 35.63 |  |
| ATP2A2 | ATPase, Ca++ transporting, cardiac muscle, slow twitch 2 | 212362_at | 1.21 | 43.91 |  | 0.26 | 0.00 | ↓ | 1.99 | 2.43 |  |
| TRIO | triple functional domain (PTPRF interacting) | 209013_x_at | 1.21 | 36.49 |  | 0.48 | 4.45 | ↓ | 1.30 | 26.48 |  |
| PRKAB1 | protein kinase, AMP-activated, beta 1 non-catalytic subunit | 201835_s_at | 1.21 | 34.35 |  | 0.29 | 0.00 | ↓ | 1.24 | 26.48 |  |
| TOM1L1 | target of myb1 (chicken)-like 1 | 204485_s_at | 1.21 | 45.69 |  | 1.39 | 19.82 |  | 0.30 | 0.00 | ↓ |
| TNFRSF11B | tumor necrosis factor receptor superfamily, member 11b | 204933_s_at | 1.21 | 54.53 |  | 0.88 | 50.64 |  | 2.63 | 0.89 | ↑ |
| NSMAF | neutral sphingomyelinase (N-SMase) activation associated factor | 1558775_s_at | 1.21 | 59.80 |  | 0.25 | 0.18 | ↓ | 0.95 | 54.08 |  |
| UBE3B | ubiquitin protein ligase E3B | 212404_s_at | 1.21 | 43.91 |  | 0.19 | 0.00 | ↓ | 1.26 | 38.27 |  |
| TNFRSF10D | tumor necrosis factor receptor superfamily, member 10d, decoy with truncated death domain | 210654_at | 1.21 | 42.20 |  | 0.28 | 1.45 | ↓ | 1.45 | 26.48 |  |
| TNRC6B | trinucleotide repeat containing 6B | 230779_at | 1.21 | 43.91 |  | 2.62 | 1.07 | ↑ | 1.28 | 41.22 |  |
| PSMF1 | proteasome (prosome, macropain) inhibitor subunit 1 (PI31) | 201052_s_at | 1.21 | 32.33 |  | 0.34 | 0.00 | ↓ | 0.82 | 20.38 |  |
| HNMT | histamine N-methyltransferase | 211732_x_at | 1.21 | 50.55 |  | 1.18 | 36.20 |  | 0.34 | 0.34 | ↓ |
| LOC100128252 | hypothetical LOC100128252 | 244740_at | 1.21 | 47.41 |  | 1.09 | 60.11 |  | 0.31 | 0.00 | ↓ |
| ARHGAP32 | Rho GTPase activating protein 32 | 203431_s_at | 1.21 | 38.54 |  | 3.75 | 0.60 | ↑ | 0.63 | 11.20 |  |
| RAB33B | RAB33B, member RAS oncogene family | 221014_s_at | 1.21 | 36.49 |  | 2.01 | 2.80 | ↑ | 1.23 | 41.22 |  |
| TFDP1 | transcription factor Dp-1 | 204147_s_at | 1.21 | 43.91 |  | 0.38 | 0.18 | ↓ | 0.94 | 46.35 |  |
| PPM1A | protein phosphatase, Mg2+/Mn2+ dependent, 1A | 235344_at | 1.21 | 38.54 |  | 0.50 | 2.30 | ↓ | 1.11 | 54.08 |  |
| CDKN2C | cyclin-dependent kinase inhibitor 2C (p18, inhibits CDK4) | 211792_s_at | 1.21 | 42.20 |  | 0.80 | 41.29 |  | 0.31 | 0.34 | ↓ |
| COCH | coagulation factor C homolog, cochlin (Limulus polyphemus) | 1554241_at | 1.21 | 45.69 |  | 0.44 | 1.07 | ↓ | 1.27 | 48.62 |  |
| PRDM1 | PR domain containing 1, with ZNF domain | 235668_at | 1.20 | 60.94 |  | 0.34 | 1.26 | ↓ | 1.35 | 9.54 |  |
| AKT3 | v-akt murine thymoma viral oncogene homolog 3 (protein kinase B, gamma) | 222880_at | 1.20 | 38.54 |  | 0.38 | 0.76 | ↓ | 1.50 | 9.54 |  |
| PPP1R11 | protein phosphatase 1, regulatory (inhibitor) subunit 11 | 1566303_s_at | 1.20 | 38.54 |  | 0.45 | 6.11 | ↓ | 0.90 | 58.81 |  |
| CBFA2T2 | core-binding factor, runt domain, alpha subunit 2; translocated to, 2 | 238549_at | 1.20 | 47.41 |  | 1.21 | 59.12 |  | 0.46 | 0.89 | ↓ |
| SET | SET nuclear oncogene | 210231_x_at | 1.20 | 32.33 |  | 2.63 | 0.40 | ↑ | 0.97 | 52.56 |  |
| FAM86C | family with sequence similarity 86, member C | 220353_at | 1.20 | 47.41 |  | 0.34 | 1.07 | ↓ | 1.23 | 43.83 |  |
| WDR1 | WD repeat domain 1 | 210935_s_at | 1.20 | 47.41 |  | 0.29 | 0.70 | ↓ | 1.00 | 56.65 |  |
| --- | --- | 227062_at | 1.20 | 36.49 |  | 0.36 | 3.81 | ↓ | 1.75 | 20.38 |  |
| BBS5 | Bardet-Biedl syndrome 5 | 230697_at | 1.20 | 43.91 |  | 0.49 | 3.81 | ↓ | 1.42 | 26.48 |  |
| NUCKS1 | nuclear casein kinase and cyclin-dependent kinase substrate 1 | 217802_s_at | 1.20 | 32.33 |  | 2.33 | 0.98 | ↑ | 0.97 | 54.08 |  |
| CYP2U1 | cytochrome P450, family 2, subfamily U, polypeptide 1 | 226402_at | 1.20 | 42.20 |  | 1.39 | 26.77 |  | 2.01 | 2.70 | ↑ |
| --- | --- | 232991_at | 1.20 | 53.22 |  | 0.45 | 4.45 | ↓ | 1.00 | 58.81 |  |
| SGSM3 | small G protein signaling modulator 3 | 215519_x_at | 1.20 | 36.49 |  | 0.49 | 1.45 | ↓ | 0.86 | 32.58 |  |
| HEATR2 | HEAT repeat containing 2 | 1554761_a_at | 1.20 | 36.49 |  | 0.44 | 4.45 | ↓ | 0.55 | 13.22 |  |
| CARS | cysteinyl-tRNA synthetase | 240983_s_at | 1.20 | 36.49 |  | 0.36 | 0.98 | ↓ | 0.67 | 20.38 |  |
| DDX11 | DEAD/H (Asp-Glu-Ala-Asp/His) box polypeptide 11 | 208149_x_at | 1.20 | 45.69 |  | 0.52 | 1.26 |  | 0.39 | 1.61 | ↓ |
| KITLG | KIT ligand | 207029_at | 1.20 | 58.62 |  | 0.44 | 3.81 | ↓ | 0.77 | 32.58 |  |
| IRF7 | interferon regulatory factor 7 | 208436_s_at | 1.20 | 57.71 |  | 0.66 | 11.08 |  | 0.38 | 0.34 | ↓ |
| AK5 | adenylate kinase 5 | 222862_s_at | 1.20 | 42.20 |  | 0.36 | 1.74 | ↓ | 1.08 | 54.08 |  |
| MRPL30 | mitochondrial ribosomal protein L30 | 224173_s_at | 1.20 | 38.54 |  | 0.45 | 2.80 | ↓ | 1.52 | 15.27 |  |
| TMEM49 | transmembrane protein 49 | 1569003_at | 1.20 | 42.20 |  | 0.32 | 0.60 | ↓ | 0.47 | 3.36 | ↓ |
| TMEM214 | transmembrane protein 214 | 217899_at | 1.20 | 34.35 |  | 0.27 | 0.00 | ↓ | 0.88 | 41.22 |  |
| HOMER1 | homer homolog 1 (Drosophila) | 213793_s_at | 1.20 | 43.91 |  | 1.14 | 52.07 |  | 0.49 | 4.84 | ↓ |
| ASXL1 | additional sex combs like 1 (Drosophila) | 244519_at | 1.20 | 42.20 |  | 1.34 | 29.01 |  | 0.39 | 0.72 | ↓ |
| FGFR1OP2 | FGFR1 oncogene partner 2 | 233898_s_at | 1.20 | 36.49 |  | 2.18 | 1.45 | ↑ | 1.10 | 54.08 |  |
| PPP3CA | protein phosphatase 3, catalytic subunit, alpha isozyme | 202425_x_at | 1.20 | 32.33 |  | 4.89 | 0.00 | ↑ | 1.14 | 50.73 |  |
| CECR5 | cat eye syndrome chromosome region, candidate 5 | 218592_s_at | 1.20 | 47.41 |  | 1.20 | 36.20 |  | 0.49 | 4.84 | ↓ |
| NMT1 | N-myristoyltransferase 1 | 201159_s_at | 1.20 | 47.41 |  | 0.45 | 0.25 | ↓ | 1.23 | 48.62 |  |
| SP1 | Sp1 transcription factor | 1553685_s_at | 1.20 | 55.60 |  | 0.29 | 0.00 | ↓ | 0.96 | 54.08 |  |
| ZNF281 | zinc finger protein 281 | 218401_s_at | 1.20 | 42.20 |  | 3.94 | 0.53 | ↑ | 1.37 | 23.22 |  |
| VN1R1 | vomeronasal 1 receptor 1 | 221412_at | 1.20 | 42.20 |  | 0.50 | 9.01 | ↓ | 1.39 | 29.62 |  |
| SNHG3 | small nucleolar RNA host gene 3 (non-protein coding) | 215011_at | 1.19 | 51.95 |  | 1.27 | 29.01 |  | 0.35 | 0.89 | ↓ |
| FKBP1A | FK506 binding protein 1A, 12kDa | 214119_s_at | 1.19 | 54.53 |  | 0.36 | 0.45 | ↓ | 1.22 | 38.27 |  |
| UBAP2L | ubiquitin associated protein 2-like | 201378_s_at | 1.19 | 38.54 |  | 0.29 | 0.00 | ↓ | 1.17 | 43.83 |  |
| TAPBP | TAP binding protein (tapasin) | 208829_at | 1.19 | 45.69 |  | 0.41 | 0.36 | ↓ | 0.58 | 2.43 |  |
| ITGB8 | integrin, beta 8 | 226189_at | 1.19 | 36.49 |  | 0.97 | 52.07 |  | 2.81 | 1.99 | ↑ |
| WWP2 | WW domain containing E3 ubiquitin protein ligase 2 | 210200_at | 1.19 | 47.41 |  | 0.39 | 0.53 | ↓ | 1.13 | 50.73 |  |
| SP1 | Sp1 transcription factor | 214732_at | 1.19 | 36.49 |  | 0.34 | 3.35 | ↓ | 0.62 | 20.38 |  |
| AKAP2 /// PALM2-AKAP2 | A kinase (PRKA) anchor protein 2 /// PALM2-AKAP2 readthrough | 202760_s_at | 1.19 | 40.42 |  | 0.71 | 26.77 |  | 2.13 | 1.99 | ↑ |
| SLC8A1 | solute carrier family 8 (sodium/calcium exchanger), member 1 | 207053_at | 1.19 | 61.53 |  | 0.26 | 0.36 | ↓ | 0.98 | 48.62 |  |
| ACO2 | aconitase 2, mitochondrial | 200793_s_at | 1.19 | 34.35 |  | 0.29 | 0.00 | ↓ | 0.74 | 11.20 |  |
| DAB2 | disabled homolog 2, mitogen-responsive phosphoprotein (Drosophila) | 201278_at | 1.19 | 34.35 |  | 1.11 | 47.13 |  | 2.00 | 0.43 | ↑ |
| EGR1 | Early growth response 1 | 227404_s_at | 1.19 | 54.53 |  | 0.40 | 4.45 | ↓ | 0.77 | 35.63 |  |
| PEG10 | paternally expressed 10 | 212094_at | 1.19 | 53.22 |  | 0.29 | 5.22 | ↓ | 0.76 | 50.73 |  |
| NFYA | nuclear transcription factor Y, alpha | 204109_s_at | 1.19 | 38.54 |  | 0.45 | 0.76 | ↓ | 0.74 | 17.70 |  |
| TADA3 | transcriptional adaptor 3 | 215273_s_at | 1.19 | 36.49 |  | 0.39 | 0.98 | ↓ | 0.67 | 17.70 |  |
| AMFR | autocrine motility factor receptor | 202203_s_at | 1.19 | 51.95 |  | 2.28 | 9.01 | ↑ | 0.89 | 54.08 |  |
| SLC2A5 | solute carrier family 2 (facilitated glucose/fructose transporter), member 5 | 204430_s_at | 1.19 | 47.41 |  | 0.58 | 29.01 |  | 2.85 | 3.36 | ↑ |
| PEG3 | paternally expressed 3 | 209242_at | 1.19 | 62.00 |  | 1.52 | 12.66 |  | 0.32 | 2.70 | ↓ |
| LSM2 | LSM2 homolog, U6 small nuclear RNA associated (S. cerevisiae) | 209449_at | 1.19 | 45.69 |  | 0.96 | 57.71 |  | 0.45 | 0.21 | ↓ |
| EML3 | echinoderm microtubule associated protein like 3 | 212969_x_at | 1.19 | 38.54 |  | 0.39 | 0.70 | ↓ | 0.92 | 46.35 |  |
| MRPL38 | mitochondrial ribosomal protein L38 | 225103_at | 1.19 | 40.42 |  | 0.30 | 0.00 | ↓ | 0.77 | 11.20 |  |
| HSF2 | heat shock transcription factor 2 | 211220_s_at | 1.19 | 59.80 |  | 0.40 | 0.45 | ↓ | 0.83 | 29.62 |  |
| CHKB-CPT1B /// CPT1B | choline kinase-like, carnitine palmitoyltransferase 1B (muscle) transcription unit /// carnitine palmitoyltransferase 1B (muscle) | 210070_s_at | 1.19 | 45.69 |  | 0.43 | 3.35 | ↓ | 1.02 | 56.10 |  |
| TMEM150C | Transmembrane protein 150C | 229623_at | 1.19 | 50.55 |  | 2.14 | 12.66 |  | 0.40 | 4.84 | ↓ |
| GLE1 | GLE1 RNA export mediator homolog (yeast) | 206920_s_at | 1.19 | 43.91 |  | 0.40 | 1.07 | ↓ | 1.02 | 57.14 |  |
| SULT1A3 /// SULT1A4 | sulfotransferase family, cytosolic, 1A, phenol-preferring, member 3 /// sulfotransferase family, cytosolic, 1A, phenol-preferring, member 4 | 210580_x_at | 1.18 | 53.22 |  | 1.35 | 26.77 |  | 0.44 | 0.21 | ↓ |
| LOC401233 | HIV-1 Tat specific factor 1 pseudogene | 1558882_at | 1.18 | 43.91 |  | 1.81 | 17.74 |  | 0.36 | 8.02 | ↓ |
| ITGB3 | integrin, beta 3 (platelet glycoprotein IIIa, antigen CD61) | 204627_s_at | 1.18 | 38.54 |  | 0.50 | 24.54 |  | 0.26 | 3.36 | ↓ |
| BMP1 | bone morphogenetic protein 1 | 205574_x_at | 1.18 | 36.49 |  | 0.35 | 0.29 | ↓ | 0.62 | 7.00 |  |
| TP53 | tumor protein p53 | 211300_s_at | 1.18 | 38.54 |  | 0.18 | 0.36 | ↓ | 0.89 | 58.81 |  |
| GTF3C2 | general transcription factor IIIC, polypeptide 2, beta 110kDa | 210620_s_at | 1.18 | 62.00 |  | 0.36 | 1.98 | ↓ | 1.03 | 57.64 |  |
| CDK13 | cyclin-dependent kinase 13 | 207319_s_at | 1.18 | 48.99 |  | 0.31 | 0.60 | ↓ | 1.09 | 52.56 |  |
| CYB5R1 | cytochrome b5 reductase 1 | 202263_at | 1.18 | 43.91 |  | 1.02 | 57.71 |  | 2.47 | 0.43 | ↑ |
| ATP2B4 | ATPase, Ca++ transporting, plasma membrane 4 | 205410_s_at | 1.18 | 51.95 |  | 0.21 | 0.45 | ↓ | 2.17 | 7.00 | ↑ |
| SLC7A5 | solute carrier family 7 (cationic amino acid transporter, y+ system), member 5 | 201195_s_at | 1.18 | 56.74 |  | 1.16 | 41.29 |  | 0.45 | 5.75 | ↓ |
| ASB1 | ankyrin repeat and SOCS box-containing 1 | 212819_at | 1.18 | 45.69 |  | 0.91 | 49.04 |  | 2.03 | 1.25 | ↑ |
| SLFN5 | schlafen family member 5 | 1553055_a_at | 1.18 | 57.71 |  | 0.45 | 2.80 | ↓ | 1.07 | 58.28 |  |
| FBXO28 | F-box protein 28 | 1555972_s_at | 1.18 | 58.62 |  | 0.18 | 0.18 | ↓ | 1.44 | 29.62 |  |
| DNAJC5 | DnaJ (Hsp40) homolog, subfamily C, member 5 | 224613_s_at | 1.18 | 42.20 |  | 0.49 | 1.45 | ↓ | 1.24 | 32.58 |  |
| EHD4 | EH-domain containing 4 | 233660_at | 1.18 | 43.91 |  | 0.29 | 0.00 | ↓ | 1.13 | 56.10 |  |
| PTER | phosphotriesterase related | 218967_s_at | 1.18 | 48.99 |  | 1.39 | 26.77 |  | 0.49 | 4.84 | ↓ |
| RPS6KA2 | ribosomal protein S6 kinase, 90kDa, polypeptide 2 | 1557970_s_at | 1.18 | 53.22 |  | 0.04 | 0.00 | ↓ | 1.64 | 26.48 |  |
| TSC22D1 | TSC22 domain family, member 1 | 235315_at | 1.18 | 55.60 |  | 2.75 | 3.35 | ↑ | 1.22 | 48.62 |  |
| ATG13 | ATG13 autophagy related 13 homolog (S. cerevisiae) | 203363_s_at | 1.18 | 40.42 |  | 0.26 | 0.00 | ↓ | 1.22 | 15.27 |  |
| PLCG1 | phospholipase C, gamma 1 | 216551_x_at | 1.18 | 45.69 |  | 0.47 | 1.45 | ↓ | 0.86 | 35.63 |  |
| DLX1 | distal-less homeobox 1 | 242138_at | 1.18 | 42.20 |  | 1.32 | 31.53 |  | 0.41 | 8.02 | ↓ |
| NF2 | neurofibromin 2 (merlin) | 238618_at | 1.18 | 62.00 |  | 0.31 | 0.25 | ↓ | 0.93 | 50.73 |  |
| SPSB1 | splA/ryanodine receptor domain and SOCS box containing 1 | 226075_at | 1.18 | 43.91 |  | 0.34 | 0.18 | ↓ | 1.14 | 54.08 |  |
| NAPA | N-ethylmaleimide-sensitive factor attachment protein, alpha | 206491_s_at | 1.18 | 56.74 |  | 0.15 | 0.00 | ↓ | 1.13 | 46.35 |  |
| NFE2L3 | nuclear factor (erythroid-derived 2)-like 3 | 204702_s_at | 1.18 | 59.80 |  | 0.72 | 22.17 |  | 0.46 | 2.70 | ↓ |
| DNAJA4 | DnaJ (Hsp40) homolog, subfamily A, member 4 | 1554334_a_at | 1.17 | 50.55 |  | 0.48 | 2.80 | ↓ | 1.17 | 54.08 |  |
| MYL12A | myosin, light chain 12A, regulatory, non-sarcomeric | 201319_at | 1.17 | 38.54 |  | 2.22 | 0.36 | ↑ | 2.02 | 2.43 | ↑ |
| ABHD10 | abhydrolase domain containing 10 | 222697_s_at | 1.17 | 47.41 |  | 0.43 | 0.84 | ↓ | 0.77 | 26.48 |  |
| GPX3 | glutathione peroxidase 3 (plasma) | 214091_s_at | 1.17 | 47.41 |  | 0.33 | 0.36 | ↓ | 0.60 | 8.02 |  |
| CLCN7 | chloride channel 7 | 221961_at | 1.17 | 61.53 |  | 0.44 | 0.53 | ↓ | 1.06 | 57.64 |  |
| FOXN3 | forkhead box N3 | 241984_at | 1.17 | 43.91 |  | 0.46 | 4.45 | ↓ | 2.12 | 4.84 | ↑ |
| TRIO | triple functional domain (PTPRF interacting) | 208178_x_at | 1.17 | 40.42 |  | 0.44 | 3.35 | ↓ | 1.31 | 29.62 |  |
| PHPT1 | phosphohistidine phosphatase 1 | 225683_x_at | 1.17 | 61.68 |  | 1.14 | 49.04 |  | 0.44 | 3.36 | ↓ |
| FLCN | folliculin | 1552664_at | 1.17 | 47.41 |  | 2.20 | 0.76 | ↑ | 1.05 | 58.81 |  |
| SEMA4B | sema domain, immunoglobulin domain (Ig), transmembrane domain (TM) and short cytoplasmic domain, (semaphorin) 4B | 234725_s_at | 1.17 | 40.42 |  | 0.63 | 7.87 |  | 0.42 | 2.43 | ↓ |
| AGPAT3 | 1-acylglycerol-3-phosphate O-acyltransferase 3 | 219723_x_at | 1.17 | 58.62 |  | 0.28 | 0.36 | ↓ | 0.94 | 58.81 |  |
| ABCC4 | ATP-binding cassette, sub-family C (CFTR/MRP), member 4 | 1555039_a_at | 1.17 | 59.06 |  | 0.37 | 1.07 | ↓ | 1.45 | 26.48 |  |
| PCDHB10 | protocadherin beta 10 | 223854_at | 1.17 | 40.42 |  | 2.04 | 9.01 | ↑ | 1.38 | 43.83 |  |
| DUSP8 | dual specificity phosphatase 8 | 206374_at | 1.17 | 50.55 |  | 0.72 | 24.54 |  | 0.43 | 1.99 | ↓ |
| SPOCD1 | SPOC domain containing 1 | 1562415_a_at | 1.17 | 40.42 |  | 1.03 | 59.12 |  | 0.30 | 0.21 | ↓ |
| RBM33 | RNA binding motif protein 33 | 238801_at | 1.17 | 47.41 |  | 0.32 | 1.07 | ↓ | 0.67 | 15.27 |  |
| RDH5 | retinol dehydrogenase 5 (11-cis/9-cis) | 210106_at | 1.17 | 57.71 |  | 0.37 | 1.98 | ↓ | 0.67 | 17.70 |  |
| CALU | calumenin | 214845_s_at | 1.17 | 48.99 |  | 0.37 | 0.25 | ↓ | 1.23 | 32.58 |  |
| HECTD1 | HECT domain containing 1 | 241955_at | 1.16 | 42.20 |  | 3.36 | 0.00 | ↑ | 2.27 | 0.50 | ↑ |
| FGF2 | fibroblast growth factor 2 (basic) | 204422_s_at | 1.16 | 54.53 |  | 1.31 | 31.53 |  | 2.11 | 0.72 | ↑ |
| MST4 | serine/threonine protein kinase MST4 | 224407_s_at | 1.16 | 56.74 |  | 0.25 | 0.18 | ↓ | 0.19 | 0.21 | ↓ |
| ABHD12 | abhydrolase domain containing 12 | 228123_s_at | 1.16 | 42.20 |  | 0.42 | 0.18 | ↓ | 0.80 | 20.38 |  |
| ERAP1 | endoplasmic reticulum aminopeptidase 1 | 214012_at | 1.16 | 45.69 |  | 2.32 | 11.08 |  | 0.36 | 1.43 | ↓ |
| EFNB2 | ephrin-B2 | 202669_s_at | 1.16 | 58.62 |  | 0.57 | 6.11 |  | 0.45 | 5.75 | ↓ |
| --- | --- | 238320_at | 1.16 | 62.00 |  | 0.09 | 0.00 | ↓ | 2.07 | 5.75 | ↑ |
| BCL2L13 | BCL2-like 13 (apoptosis facilitator) | 224035_s_at | 1.16 | 50.55 |  | 0.17 | 0.00 | ↓ | 1.02 | 58.28 |  |
| RERE | arginine-glutamic acid dipeptide (RE) repeats | 221643_s_at | 1.16 | 48.99 |  | 0.22 | 0.00 | ↓ | 0.90 | 35.63 |  |
| CIZ1 | CDKN1A interacting zinc finger protein 1 | 213977_s_at | 1.16 | 47.41 |  | 0.18 | 0.00 | ↓ | 0.56 | 4.84 |  |
| GNS | glucosamine (N-acetyl)-6-sulfatase | 203676_at | 1.16 | 59.06 |  | 0.20 | 0.00 | ↓ | 1.64 | 13.22 |  |
| NAMPT | nicotinamide phosphoribosyltransferase | 1555167_s_at | 1.16 | 53.22 |  | 0.35 | 0.40 | ↓ | 1.15 | 48.62 |  |
| GPS1 | G protein pathway suppressor 1 | 217782_s_at | 1.16 | 43.91 |  | 0.43 | 0.40 | ↓ | 1.06 | 55.52 |  |
| MAGED4 /// MAGED4B | melanoma antigen family D, 4 /// melanoma antigen family D, 4B | 223313_s_at | 1.16 | 47.41 |  | 0.84 | 41.29 |  | 0.11 | 0.00 | ↓ |
| ANKRD12 | ankyrin repeat domain 12 | 216550_x_at | 1.16 | 57.71 |  | 2.92 | 0.60 | ↑ | 1.11 | 55.52 |  |
| LARP4 | La ribonucleoprotein domain family, member 4 | 238960_s_at | 1.16 | 61.91 |  | 0.43 | 0.45 | ↓ | 1.39 | 20.38 |  |
| RAB5A | RAB5A, member RAS oncogene family | 206113_s_at | 1.16 | 60.45 |  | 0.38 | 0.18 | ↓ | 1.23 | 35.63 |  |
| ASB5 | ankyrin repeat and SOCS box-containing 5 | 235503_at | 1.16 | 56.74 |  | 2.08 | 9.01 | ↑ | 0.73 | 26.48 |  |
| IRS2 | insulin receptor substrate 2 | 209185_s_at | 1.16 | 58.62 |  | 1.70 | 31.53 |  | 2.92 | 1.61 | ↑ |
| KCTD10 | potassium channel tetramerisation domain containing 10 | 223208_at | 1.16 | 42.20 |  | 0.50 | 1.74 | ↓ | 1.39 | 15.27 |  |
| FAM108B1 | family with sequence similarity 108, member B1 | 220285_at | 1.16 | 61.79 |  | 0.41 | 0.84 | ↓ | 1.17 | 46.35 |  |
| LOC100506057 /// STK32C | hypothetical LOC100506057 /// serine/threonine kinase 32C | 230934_at | 1.16 | 62.00 |  | 0.80 | 26.77 |  | 0.48 | 0.00 | ↓ |
| GLS | glutaminase | 223079_s_at | 1.16 | 48.99 |  | 0.27 | 0.29 | ↓ | 1.68 | 7.00 |  |
| ZNF546 | zinc finger protein 546 | 240429_at | 1.16 | 51.95 |  | 2.01 | 4.45 | ↑ | 1.07 | 54.08 |  |
| GRPEL2 | GrpE-like 2, mitochondrial (E. coli) | 226881_at | 1.16 | 42.20 |  | 2.05 | 0.45 | ↑ | 1.12 | 46.35 |  |
| TUBGCP3 | tubulin, gamma complex associated protein 3 | 215739_s_at | 1.16 | 47.41 |  | 0.45 | 0.29 | ↓ | 0.64 | 2.43 |  |
| TRUB1 | TruB pseudouridine (psi) synthase homolog 1 (E. coli) | 241606_s_at | 1.16 | 50.55 |  | 0.39 | 0.25 | ↓ | 1.01 | 58.81 |  |
| SLFN5 | schlafen family member 5 | 1557078_at | 1.16 | 61.79 |  | 0.42 | 1.26 | ↓ | 0.87 | 41.22 |  |
| MARS | methionyl-tRNA synthetase | 213672_at | 1.16 | 59.06 |  | 1.03 | 52.07 |  | 0.45 | 3.36 | ↓ |
| PHKB | phosphorylase kinase, beta | 242248_at | 1.16 | 50.55 |  | 0.51 | 7.87 |  | 0.45 | 5.75 | ↓ |
| SLC8A1 | solute carrier family 8 (sodium/calcium exchanger), member 1 | 211805_s_at | 1.16 | 58.62 |  | 0.45 | 4.45 | ↓ | 0.85 | 32.58 |  |
| C1orf144 | chromosome 1 open reading frame 144 | 212005_at | 1.16 | 48.99 |  | 0.44 | 1.74 | ↓ | 0.79 | 15.27 |  |
| HFE | hemochromatosis | 211329_x_at | 1.15 | 45.69 |  | 0.46 | 0.84 | ↓ | 0.84 | 32.58 |  |
| CANX | calnexin | 208853_s_at | 1.15 | 55.60 |  | 0.44 | 0.70 | ↓ | 0.96 | 50.73 |  |
| STX16 | syntaxin 16 | 221638_s_at | 1.15 | 55.60 |  | 0.13 | 0.00 | ↓ | 1.09 | 55.52 |  |
| ILF3 | interleukin enhancer binding factor 3, 90kDa | 217805_at | 1.15 | 48.99 |  | 0.79 | 26.77 |  | 0.48 | 0.21 | ↓ |
| PACSIN2 | protein kinase C and casein kinase substrate in neurons 2 | 1554691_a_at | 1.15 | 47.41 |  | 0.48 | 1.45 | ↓ | 0.94 | 50.73 |  |
| RPL37A | Ribosomal protein L37a | 214041_x_at | 1.15 | 47.41 |  | 2.10 | 2.30 | ↑ | 1.64 | 2.70 |  |
| RBAK | RB-associated KRAB zinc finger | 1553122_s_at | 1.15 | 45.69 |  | 0.32 | 0.70 | ↓ | 1.15 | 46.35 |  |
| PRIC285 | peroxisomal proliferator-activated receptor A interacting complex 285 | 228230_at | 1.15 | 61.35 |  | 0.40 | 0.70 | ↓ | 0.32 | 0.34 | ↓ |
| TRRAP | transformation/transcription domain-associated protein | 214908_s_at | 1.15 | 51.95 |  | 0.19 | 0.18 | ↓ | 1.05 | 55.52 |  |
| FLOT1 | flotillin 1 | 208748_s_at | 1.15 | 55.60 |  | 0.34 | 0.18 | ↓ | 0.91 | 41.22 |  |
| DAK | dihydroxyacetone kinase 2 homolog (S. cerevisiae) | 218688_at | 1.15 | 57.71 |  | 0.41 | 0.53 | ↓ | 0.89 | 32.58 |  |
| MT1H | metallothionein 1H | 206461_x_at | 1.15 | 45.69 |  | 1.49 | 31.53 |  | 0.46 | 2.43 | ↓ |
| ETV1 | ets variant 1 | 217053_x_at | 1.15 | 62.00 |  | 0.44 | 0.53 | ↓ | 0.85 | 29.62 |  |
| RAB6A | RAB6A, member RAS oncogene family | 201048_x_at | 1.15 | 54.53 |  | 0.15 | 0.00 | ↓ | 1.08 | 57.64 |  |
| CEPT1 | choline/ethanolamine phosphotransferase 1 | 219375_at | 1.15 | 45.69 |  | 2.50 | 0.98 | ↑ | 1.08 | 52.56 |  |
| JAG1 | jagged 1 | 209099_x_at | 1.15 | 50.55 |  | 1.64 | 15.95 |  | 0.41 | 1.61 | ↓ |
| SPATA24 | spermatogenesis associated 24 | 1558641_at | 1.15 | 47.41 |  | 0.55 | 3.81 |  | 2.33 | 1.99 | ↑ |
| SH3PXD2A | SH3 and PX domains 2A | 207661_s_at | 1.15 | 38.54 |  | 1.02 | 54.70 |  | 2.09 | 4.84 | ↑ |
| SPAG9 | sperm associated antigen 9 | 206748_s_at | 1.15 | 59.80 |  | 0.16 | 0.00 | ↓ | 1.44 | 15.27 |  |
| RORA | RAR-related orphan receptor A | 210426_x_at | 1.15 | 62.00 |  | 1.94 | 22.17 |  | 0.41 | 0.21 | ↓ |
| RIPK3 | receptor-interacting serine-threonine kinase 3 | 228139_at | 1.15 | 56.74 |  | 0.47 | 1.26 | ↓ | 1.29 | 48.62 |  |
| C18orf1 | chromosome 18 open reading frame 1 | 207996_s_at | 1.15 | 62.00 |  | 3.90 | 3.81 | ↑ | 0.37 | 2.70 | ↓ |
| SVIL | supervillin | 202566_s_at | 1.15 | 45.69 |  | 0.45 | 6.11 | ↓ | 1.54 | 15.27 |  |
| ZBTB47 | zinc finger and BTB domain containing 47 | 226500_at | 1.14 | 48.99 |  | 1.44 | 15.95 |  | 2.00 | 3.36 | ↑ |
| CSNK2A1 | casein kinase 2, alpha 1 polypeptide | 212075_s_at | 1.14 | 53.22 |  | 0.37 | 0.25 | ↓ | 0.92 | 43.83 |  |
| SPG7 | spastic paraplegia 7 (pure and complicated autosomal recessive) | 230884_s_at | 1.14 | 59.80 |  | 0.32 | 0.25 | ↓ | 1.00 | 58.81 |  |
| ACTR3B /// LOC100290215 | ARP3 actin-related protein 3 homolog B (yeast) /// actin-related protein 3B-like | 1555487_a_at | 1.14 | 58.62 |  | 0.49 | 1.98 | ↓ | 1.16 | 52.56 |  |
| GOLGA6L5 /// GOLGA6L9 | golgin A6 family-like 5 (pseudogene) /// golgin A6 family-like 9 | 213212_x_at | 1.14 | 60.72 |  | 2.17 | 7.87 | ↑ | 1.03 | 54.08 |  |
| TLE4 | transducin-like enhancer of split 4 (E(sp1) homolog, Drosophila) | 233575_s_at | 1.14 | 60.45 |  | 0.42 | 3.81 | ↓ | 0.83 | 46.35 |  |
| GKAP1 | G kinase anchoring protein 1 | 234192_s_at | 1.14 | 47.41 |  | 1.79 | 12.66 |  | 0.45 | 7.00 | ↓ |
| TAB3 | TGF-beta activated kinase 1/MAP3K7 binding protein 3 | 1552928_s_at | 1.14 | 60.72 |  | 0.22 | 1.26 | ↓ | 1.43 | 35.63 |  |
| HNRNPL | heterogeneous nuclear ribonucleoprotein L | 221860_at | 1.14 | 60.72 |  | 2.22 | 5.22 | ↑ | 0.77 | 29.62 |  |
| PPM1G | protein phosphatase, Mg2+/Mn2+ dependent, 1G | 200913_at | 1.14 | 56.74 |  | 1.11 | 47.13 |  | 0.49 | 3.36 | ↓ |
| ADC | arginine decarboxylase | 1554393_a_at | 1.14 | 58.62 |  | 0.45 | 0.98 | ↓ | 0.78 | 20.38 |  |
| USP25 | ubiquitin specific peptidase 25 | 1555559_s_at | 1.14 | 61.91 |  | 0.19 | 0.18 | ↓ | 0.96 | 54.08 |  |
| NCRNA00086 /// NCRNA00087 | non-protein coding RNA 86 /// non-protein coding RNA 87 | 227909_at | 1.14 | 53.22 |  | 1.24 | 52.07 |  | 0.47 | 4.84 | ↓ |
| C9orf5 | chromosome 9 open reading frame 5 | 223005_s_at | 1.14 | 59.80 |  | 0.43 | 0.18 | ↓ | 1.12 | 46.35 |  |
| CHMP4B | chromatin modifying protein 4B | 225119_at | 1.14 | 51.95 |  | 2.06 | 3.35 | ↑ | 1.86 | 9.54 |  |
| LOC729082 | Hypothetical protein LOC729082 | 225332_at | 1.14 | 53.22 |  | 2.27 | 3.35 | ↑ | 0.96 | 58.81 |  |
| DCTN1 | dynactin 1 | 201082_s_at | 1.14 | 59.06 |  | 0.20 | 0.00 | ↓ | 1.88 | 4.84 |  |
| BAZ2A | bromodomain adjacent to zinc finger domain, 2A | 201353_s_at | 1.14 | 50.55 |  | 0.34 | 0.18 | ↓ | 0.69 | 9.54 |  |
| PLBD1 | phospholipase B domain containing 1 | 218454_at | 1.14 | 58.62 |  | 0.76 | 33.65 |  | 0.42 | 1.61 | ↓ |
| DGKA | diacylglycerol kinase, alpha 80kDa | 211272_s_at | 1.14 | 51.95 |  | 0.20 | 0.00 | ↓ | 0.82 | 26.48 |  |
| GCLC | glutamate-cysteine ligase, catalytic subunit | 202922_at | 1.14 | 55.60 |  | 1.88 | 3.81 |  | 2.80 | 1.61 | ↑ |
| TBC1D5 | TBC1 domain family, member 5 | 201815_s_at | 1.14 | 54.53 |  | 0.35 | 0.25 | ↓ | 0.91 | 43.83 |  |
| NXT2 | nuclear transport factor 2-like export factor 2 | 209629_s_at | 1.14 | 62.00 |  | 0.24 | 0.18 | ↓ | 1.31 | 38.27 |  |
| RSBN1 | round spermatid basic protein 1 | 222788_s_at | 1.14 | 51.95 |  | 2.30 | 7.87 | ↑ | 0.66 | 29.62 |  |
| TRIO | triple functional domain (PTPRF interacting) | 209010_s_at | 1.14 | 53.22 |  | 0.45 | 4.45 | ↓ | 0.96 | 54.08 |  |
| TMEM170A | transmembrane protein 170A | 227586_at | 1.14 | 54.53 |  | 1.17 | 41.29 |  | 0.49 | 0.58 | ↓ |
| SBNO1 | strawberry notch homolog 1 (Drosophila) | 218737_at | 1.14 | 54.53 |  | 3.40 | 0.40 | ↑ | 1.10 | 55.52 |  |
| KCTD15 | potassium channel tetramerisation domain containing 15 | 222664_at | 1.14 | 50.55 |  | 1.58 | 14.26 |  | 0.19 | 0.00 | ↓ |
| LARP4 | La ribonucleoprotein domain family, member 4 | 1555384_a_at | 1.14 | 45.69 |  | 0.38 | 0.00 | ↓ | 1.54 | 4.16 |  |
| SLC25A37 | solute carrier family 25, member 37 | 242335_at | 1.13 | 48.99 |  | 1.39 | 24.54 |  | 0.47 | 0.89 | ↓ |
| VIPAR | VPS33B interacting protein, apical-basolateral polarity regulator | 233140_s_at | 1.13 | 45.69 |  | 0.34 | 0.53 | ↓ | 0.91 | 38.27 |  |
| MOBKL2C | MOB1, Mps One Binder kinase activator-like 2C (yeast) | 243052_at | 1.13 | 61.79 |  | 0.45 | 0.70 | ↓ | 0.98 | 50.73 |  |
| ADAMTS5 | ADAM metallopeptidase with thrombospondin type 1 motif, 5 | 229357_at | 1.13 | 42.20 |  | 1.20 | 38.78 |  | 0.30 | 2.70 | ↓ |
| TMCO3 | transmembrane and coiled-coil domains 3 | 220240_s_at | 1.13 | 55.60 |  | 0.44 | 0.25 | ↓ | 1.04 | 57.92 |  |
| RNF6 | ring finger protein (C3H2C3 type) 6 | 210932_s_at | 1.13 | 54.53 |  | 0.17 | 0.00 | ↓ | 1.31 | 41.22 |  |
| POM121L9P | POM121 membrane glycoprotein-like 9, pseudogene | 206819_at | 1.13 | 60.94 |  | 0.26 | 1.07 | ↓ | 0.83 | 52.56 |  |
| IL1A | interleukin 1, alpha | 208200_at | 1.13 | 58.62 |  | 0.48 | 2.80 | ↓ | 1.20 | 50.73 |  |
| PLEC | plectin | 216971_s_at | 1.13 | 53.22 |  | 0.07 | 0.00 | ↓ | 1.01 | 58.81 |  |
| HCG4 | HLA complex group 4 | 206685_at | 1.13 | 38.54 |  | 1.53 | 54.70 |  | 0.16 | 2.43 | ↓ |
| TEAD1 | TEA domain family member 1 (SV40 transcriptional enhancer factor) | 214600_at | 1.13 | 43.91 |  | 2.04 | 7.87 | ↑ | 1.42 | 26.48 |  |
| FAM126A | family with sequence similarity 126, member A | 231396_s_at | 1.13 | 55.60 |  | 0.47 | 0.84 | ↓ | 1.09 | 58.81 |  |
| KYNU | kynureninase (L-kynurenine hydrolase) | 217388_s_at | 1.13 | 58.62 |  | 0.41 | 7.87 | ↓ | 0.17 | 1.02 | ↓ |
| SLC25A28 | solute carrier family 25, member 28 | 221432_s_at | 1.13 | 45.69 |  | 0.45 | 1.98 | ↓ | 0.91 | 48.62 |  |
| KCTD15 | potassium channel tetramerisation domain containing 15 | 222668_at | 1.13 | 59.06 |  | 3.25 | 0.21 | ↑ | 0.16 | 0.00 | ↓ |
| PIP4K2B | phosphatidylinositol-5-phosphate 4-kinase, type II, beta | 201081_s_at | 1.13 | 53.22 |  | 0.46 | 0.25 | ↓ | 1.12 | 41.22 |  |
| APPL2 | adaptor protein, phosphotyrosine interaction, PH domain and leucine zipper containing 2 | 218218_at | 1.13 | 61.68 |  | 0.26 | 0.00 | ↓ | 1.37 | 20.38 |  |
| ZCCHC4 | zinc finger, CCHC domain containing 4 | 220473_s_at | 1.13 | 40.42 |  | 0.41 | 6.11 | ↓ | 0.71 | 41.22 |  |
| UHMK1 | U2AF homology motif (UHM) kinase 1 | 1552656_s_at | 1.13 | 53.22 |  | 0.28 | 0.25 | ↓ | 1.72 | 11.20 |  |
| RARS2 | arginyl-tRNA synthetase 2, mitochondrial | 1561048_at | 1.13 | 51.95 |  | 0.33 | 0.60 | ↓ | 2.26 | 1.02 | ↑ |
| APCDD1L | adenomatosis polyposis coli down-regulated 1-like | 235548_at | 1.13 | 55.60 |  | 0.89 | 41.29 |  | 0.45 | 1.02 | ↓ |
| TNFRSF21 | tumor necrosis factor receptor superfamily, member 21 | 218856_at | 1.13 | 61.53 |  | 0.65 | 31.53 |  | 5.74 | 0.58 | ↑ |
| MCTP2 | multiple C2 domains, transmembrane 2 | 229021_at | 1.13 | 47.41 |  | 0.19 | 0.40 | ↓ | 1.32 | 38.27 |  |
| SETD5 | SET domain containing 5 | 222575_at | 1.13 | 50.55 |  | 3.37 | 0.40 | ↑ | 0.78 | 20.38 |  |
| TRIM2 | tripartite motif-containing 2 | 202341_s_at | 1.13 | 58.62 |  | 2.52 | 1.07 | ↑ | 1.17 | 57.14 |  |
| KIAA1432 | KIAA1432 | 223826_s_at | 1.13 | 50.55 |  | 0.30 | 0.25 | ↓ | 1.22 | 43.83 |  |
| CFL1 | cofilin 1 (non-muscle) | 1555730_a_at | 1.13 | 50.55 |  | 0.21 | 0.00 | ↓ | 1.11 | 50.73 |  |
| LRCH3 | leucine-rich repeats and calponin homology (CH) domain containing 3 | 214739_at | 1.13 | 53.22 |  | 2.02 | 1.26 | ↑ | 1.24 | 29.62 |  |
| NTF3 | neurotrophin 3 | 206706_at | 1.13 | 62.00 |  | 0.69 | 17.74 |  | 2.29 | 0.58 | ↑ |
| PLA2G5 | phospholipase A2, group V | 206178_at | 1.13 | 55.60 |  | 0.81 | 41.29 |  | 0.41 | 1.99 | ↓ |
| LOC339803 | hypothetical LOC339803 | 227941_at | 1.13 | 61.35 |  | 2.49 | 2.80 | ↑ | 1.28 | 38.27 |  |
| CFLAR | CASP8 and FADD-like apoptosis regulator | 211317_s_at | 1.13 | 55.60 |  | 0.56 | 4.45 |  | 2.06 | 0.72 | ↑ |
| TRAK1 | trafficking protein, kinesin binding 1 | 201283_s_at | 1.13 | 61.91 |  | 0.49 | 2.30 | ↓ | 0.82 | 26.48 |  |
| LOC389199 | hypothetical LOC389199 | 1554809_at | 1.13 | 62.00 |  | 0.30 | 0.29 | ↓ | 0.94 | 35.63 |  |
| CACNB3 | calcium channel, voltage-dependent, beta 3 subunit | 209530_at | 1.13 | 60.18 |  | 0.67 | 7.87 |  | 0.41 | 0.00 | ↓ |
| FGF7 | fibroblast growth factor 7 | 1555103_s_at | 1.12 | 48.99 |  | 0.42 | 4.45 | ↓ | 1.44 | 58.81 |  |
| PPIG | peptidylprolyl isomerase G (cyclophilin G) | 208993_s_at | 1.12 | 56.74 |  | 2.18 | 0.53 | ↑ | 0.97 | 52.56 |  |
| ANKHD1 /// ANKHD1-EIF4EBP3 | ankyrin repeat and KH domain containing 1 /// ANKHD1-EIF4EBP3 readthrough | 233292_s_at | 1.12 | 56.74 |  | 0.27 | 0.00 | ↓ | 0.91 | 43.83 |  |
| ANKRD11 | ankyrin repeat domain 11 | 231999_at | 1.12 | 48.99 |  | 3.63 | 0.21 | ↑ | 1.02 | 58.81 |  |
| CYBRD1 | cytochrome b reductase 1 | 217889_s_at | 1.12 | 56.74 |  | 0.62 | 9.01 |  | 2.19 | 2.43 | ↑ |
| TACC1 | transforming, acidic coiled-coil containing protein 1 | 1554690_a_at | 1.12 | 53.22 |  | 2.59 | 1.98 | ↑ | 0.74 | 29.62 |  |
| ALAD | aminolevulinate dehydratase | 218489_s_at | 1.12 | 55.60 |  | 0.46 | 1.74 | ↓ | 0.73 | 11.20 |  |
| CALML4 | calmodulin-like 4 | 64408_s_at | 1.12 | 59.48 |  | 0.70 | 22.17 |  | 0.36 | 1.99 | ↓ |
| LOC100134259 | similar to hCG1987718 | 230799_at | 1.12 | 47.41 |  | 0.98 | 55.85 |  | 0.23 | 0.00 | ↓ |
| KCTD15 | potassium channel tetramerisation domain containing 15 | 218553_s_at | 1.12 | 50.55 |  | 1.13 | 43.50 |  | 0.24 | 0.50 | ↓ |
| SQSTM1 | sequestosome 1 | 213112_s_at | 1.12 | 60.94 |  | 0.50 | 15.95 |  | 2.46 | 8.02 | ↑ |
| LOC284801 | hypothetical protein LOC284801 | 225762_x_at | 1.12 | 58.62 |  | 3.83 | 0.45 | ↑ | 0.86 | 46.35 |  |
| LACTB | lactamase, beta | 1552486_s_at | 1.12 | 51.95 |  | 0.97 | 58.42 |  | 2.38 | 1.02 | ↑ |
| E2F1 | E2F transcription factor 1 | 2028_s_at | 1.12 | 50.55 |  | 0.38 | 0.76 | ↓ | 0.59 | 3.36 |  |
| AOX1 | aldehyde oxidase 1 | 205083_at | 1.12 | 62.00 |  | 1.01 | 60.62 |  | 4.44 | 0.31 | ↑ |
| COPE | coatomer protein complex, subunit epsilon | 201264_at | 1.12 | 51.95 |  | 0.42 | 0.70 | ↓ | 0.86 | 35.63 |  |
| PCDHB16 | protocadherin beta 16 | 232099_at | 1.12 | 54.53 |  | 1.60 | 14.26 |  | 2.58 | 4.16 | ↑ |
| CLCN5 | chloride channel 5 | 232127_at | 1.12 | 53.22 |  | 1.69 | 24.54 |  | 2.14 | 7.00 | ↑ |
| PSEN1 | presenilin 1 | 238816_at | 1.12 | 51.95 |  | 0.29 | 0.18 | ↓ | 1.03 | 57.64 |  |
| IQCE | IQ motif containing E | 217124_at | 1.12 | 48.99 |  | 0.70 | 36.20 |  | 0.48 | 9.54 | ↓ |
| ST3GAL2 | ST3 beta-galactoside alpha-2,3-sialyltransferase 2 | 217650_x_at | 1.12 | 59.80 |  | 2.17 | 1.98 | ↑ | 0.79 | 20.38 |  |
| USP21 | ubiquitin specific peptidase 21 | 218367_x_at | 1.12 | 53.22 |  | 0.49 | 0.98 | ↓ | 0.61 | 4.84 |  |
| CANT1 | calcium activated nucleotidase 1 | 1554327_a_at | 1.12 | 57.71 |  | 0.13 | 0.00 | ↓ | 0.88 | 46.35 |  |
| PKNOX1 | PBX/knotted 1 homeobox 1 | 216004_s_at | 1.12 | 54.53 |  | 0.28 | 0.76 | ↓ | 1.27 | 35.63 |  |
| KIAA1644 | KIAA1644 | 221901_at | 1.12 | 53.22 |  | 0.93 | 50.64 |  | 0.35 | 0.50 | ↓ |
| PTTG3P | pituitary tumor-transforming 3 (pseudogene) | 208511_at | 1.12 | 38.54 |  | 0.82 | 33.65 |  | 0.30 | 0.00 | ↓ |
| CHM | choroideremia (Rab escort protein 1) | 1569183_a_at | 1.12 | 43.91 |  | 2.05 | 7.87 | ↑ | 1.02 | 54.85 |  |
| EPSTI1 | epithelial stromal interaction 1 (breast) | 227609_at | 1.12 | 57.71 |  | 0.52 | 10.02 |  | 0.28 | 1.02 | ↓ |
| SPTAN1 | spectrin, alpha, non-erythrocytic 1 (alpha-fodrin) | 208611_s_at | 1.11 | 54.53 |  | 0.13 | 0.00 | ↓ | 1.12 | 54.85 |  |
| NUP133 | nucleoporin 133kDa | 233421_s_at | 1.11 | 62.00 |  | 0.32 | 0.98 | ↓ | 1.26 | 41.22 |  |
| TAF9B | TAF9B RNA polymerase II, TATA box binding protein (TBP)-associated factor, 31kDa | 221616_s_at | 1.11 | 58.62 |  | 0.48 | 3.81 | ↓ | 0.95 | 54.08 |  |
| STYX | serine/threonine/tyrosine interacting protein | 244030_at | 1.11 | 53.22 |  | 0.39 | 1.45 | ↓ | 1.31 | 38.27 |  |
| IRS1 | insulin receptor substrate 1 | 242979_at | 1.11 | 59.48 |  | 1.09 | 60.31 |  | 0.48 | 0.21 | ↓ |
| CYTH3 | cytohesin 3 | 206523_at | 1.11 | 54.53 |  | 0.41 | 0.53 | ↓ | 1.18 | 54.08 |  |
| MCM7 | minichromosome maintenance complex component 7 | 210983_s_at | 1.11 | 62.00 |  | 0.79 | 36.20 |  | 0.42 | 0.89 | ↓ |
| MAGI2 | membrane associated guanylate kinase, WW and PDZ domain containing 2 | 209737_at | 1.11 | 62.00 |  | 1.43 | 14.26 |  | 2.06 | 4.16 | ↑ |
| PROM2 | prominin 2 | 239528_at | 1.11 | 62.00 |  | 0.41 | 0.40 | ↓ | 1.01 | 54.08 |  |
| IRX1 | iroquois homeobox 1 | 230472_at | 1.11 | 61.35 |  | 1.17 | 49.04 |  | 0.39 | 2.70 | ↓ |
| CDA | cytidine deaminase | 205627_at | 1.11 | 55.60 |  | 0.21 | 0.25 | ↓ | 2.68 | 1.99 | ↑ |
| HPCAL1 | hippocalcin-like 1 | 212552_at | 1.11 | 50.55 |  | 0.71 | 22.17 |  | 0.41 | 0.72 | ↓ |
| KLHL7 | kelch-like 7 (Drosophila) | 220239_at | 1.11 | 59.80 |  | 1.08 | 50.64 |  | 0.46 | 0.89 | ↓ |
| UBR4 | ubiquitin protein ligase E3 component n-recognin 4 | 211950_at | 1.11 | 55.60 |  | 0.48 | 0.76 | ↓ | 0.95 | 52.56 |  |
| MIA3 | melanoma inhibitory activity family, member 3 | 1569057_s_at | 1.11 | 51.95 |  | 0.37 | 0.00 | ↓ | 1.10 | 46.35 |  |
| PARP11 | poly (ADP-ribose) polymerase family, member 11 | 229138_at | 1.11 | 58.62 |  | 2.21 | 1.07 | ↑ | 0.82 | 23.22 |  |
| ST3GAL2 | ST3 beta-galactoside alpha-2,3-sialyltransferase 2 | 205346_at | 1.11 | 62.00 |  | 0.41 | 0.98 | ↓ | 0.57 | 2.70 |  |
| CDKN2A | cyclin-dependent kinase inhibitor 2A (melanoma, p16, inhibits CDK4) | 207039_at | 1.11 | 60.18 |  | 1.09 | 49.04 |  | 4.79 | 0.00 | ↑ |
| SDC2 | syndecan 2 | 212154_at | 1.11 | 50.55 |  | 1.01 | 56.87 |  | 0.45 | 0.00 | ↓ |
| SENP3 | SUMO1/sentrin/SMT3 specific peptidase 3 | 203871_at | 1.11 | 51.95 |  | 0.46 | 1.45 | ↓ | 0.78 | 29.62 |  |
| JAM3 | junctional adhesion molecule 3 | 231721_at | 1.11 | 62.00 |  | 0.17 | 0.00 | ↓ | 0.84 | 41.22 |  |
| SLC4A7 | solute carrier family 4, sodium bicarbonate cotransporter, member 7 | 207604_s_at | 1.11 | 62.00 |  | 0.49 | 1.98 | ↓ | 2.05 | 1.43 | ↑ |
| PHLDA1 | pleckstrin homology-like domain, family A, member 1 | 217999_s_at | 1.11 | 59.80 |  | 0.45 | 0.76 | ↓ | 0.58 | 8.02 |  |
| B3GALNT2 | beta-1,3-N-acetylgalactosaminyltransferase 2 | 1553691_at | 1.11 | 55.60 |  | 0.39 | 3.81 | ↓ | 1.12 | 52.56 |  |
| GPNMB | glycoprotein (transmembrane) nmb | 201141_at | 1.11 | 54.53 |  | 1.04 | 60.11 |  | 2.14 | 7.00 | ↑ |
| KIAA1632 | KIAA1632 | 232031_s_at | 1.11 | 61.53 |  | 0.26 | 0.18 | ↓ | 1.53 | 8.02 |  |
| TGFB2 | transforming growth factor, beta 2 | 209908_s_at | 1.11 | 53.22 |  | 0.07 | 0.25 | ↓ | 0.28 | 4.84 | ↓ |
| PTPN12 | protein tyrosine phosphatase, non-receptor type 12 | 216915_s_at | 1.11 | 62.00 |  | 0.13 | 0.00 | ↓ | 1.34 | 35.63 |  |
| TGFBR2 | transforming growth factor, beta receptor II (70/80kDa) | 207334_s_at | 1.11 | 60.18 |  | 0.18 | 0.00 | ↓ | 3.17 | 2.43 | ↑ |
| MKRN2 | makorin ring finger protein 2 | 222510_s_at | 1.10 | 60.18 |  | 0.44 | 0.18 | ↓ | 1.04 | 57.64 |  |
| PITPNC1 | phosphatidylinositol transfer protein, cytoplasmic 1 | 238649_at | 1.10 | 62.00 |  | 0.48 | 17.74 |  | 0.27 | 5.75 | ↓ |
| ATP11B | ATPase, class VI, type 11B | 238811_at | 1.10 | 54.53 |  | 0.41 | 0.84 | ↓ | 1.15 | 46.35 |  |
| CFLAR | CASP8 and FADD-like apoptosis regulator | 211862_x_at | 1.10 | 57.71 |  | 0.49 | 2.80 | ↓ | 2.12 | 1.25 | ↑ |
| SLC4A7 | solute carrier family 4, sodium bicarbonate cotransporter, member 7 | 210286_s_at | 1.10 | 60.18 |  | 0.54 | 5.22 |  | 2.29 | 1.25 | ↑ |
| PDE7A | phosphodiesterase 7A | 224046_s_at | 1.10 | 55.60 |  | 0.32 | 0.25 | ↓ | 0.67 | 11.20 |  |
| CPNE1 | copine I | 206918_s_at | 1.10 | 53.22 |  | 0.42 | 0.18 | ↓ | 0.65 | 4.16 |  |
| PSRC1 | proline/serine-rich coiled-coil 1 | 201896_s_at | 1.10 | 54.53 |  | 0.81 | 36.20 |  | 0.32 | 0.00 | ↓ |
| NBR1 | neighbor of BRCA1 gene 1 | 201383_s_at | 1.10 | 59.80 |  | 0.40 | 0.00 | ↓ | 1.15 | 35.63 |  |
| LOC152742 | hypothetical LOC152742 | 240167_at | 1.10 | 40.42 |  | 1.64 | 31.53 |  | 2.79 | 4.84 | ↑ |
| AP1S1 | adaptor-related protein complex 1, sigma 1 subunit | 205195_at | 1.10 | 53.22 |  | 2.12 | 6.11 | ↑ | 1.52 | 41.22 |  |
| CHD3 | chromodomain helicase DNA binding protein 3 | 208806_at | 1.10 | 59.48 |  | 0.45 | 1.98 | ↓ | 0.80 | 32.58 |  |
| SENP7 | SUMO1/sentrin specific peptidase 7 | 223444_at | 1.10 | 59.48 |  | 2.09 | 1.45 | ↑ | 0.70 | 9.54 |  |
| SMC4 | structural maintenance of chromosomes 4 | 215623_x_at | 1.10 | 58.62 |  | 1.02 | 60.64 |  | 0.35 | 2.43 | ↓ |
| CROCC | ciliary rootlet coiled-coil, rootletin | 206274_s_at | 1.10 | 48.99 |  | 1.06 | 45.34 |  | 0.48 | 5.75 | ↓ |
| STX16 | syntaxin 16 | 221499_s_at | 1.10 | 54.53 |  | 0.49 | 1.07 | ↓ | 1.01 | 58.81 |  |
| VKORC1L1 | vitamin K epoxide reductase complex, subunit 1-like 1 | 1553679_s_at | 1.10 | 53.22 |  | 0.47 | 3.35 | ↓ | 1.14 | 46.35 |  |
| NDRG3 | NDRG family member 3 | 221082_s_at | 1.10 | 58.62 |  | 0.49 | 0.84 | ↓ | 1.11 | 55.52 |  |
| OLFML3 | olfactomedin-like 3 | 218162_at | 1.10 | 56.74 |  | 1.14 | 59.12 |  | 0.30 | 0.00 | ↓ |
| THADA | thyroid adenoma associated | 1554493_s_at | 1.10 | 59.48 |  | 0.43 | 0.45 | ↓ | 0.88 | 26.48 |  |
| GABARAPL1 | GABA(A) receptor-associated protein like 1 | 208869_s_at | 1.10 | 51.95 |  | 1.31 | 29.01 |  | 2.55 | 2.43 | ↑ |
| DDX3X | DEAD (Asp-Glu-Ala-Asp) box polypeptide 3, X-linked | 212514_x_at | 1.10 | 59.48 |  | 0.33 | 0.40 | ↓ | 1.31 | 26.48 |  |
| ACVR2A | activin A receptor, type IIA | 205327_s_at | 1.10 | 58.62 |  | 2.34 | 2.80 | ↑ | 1.12 | 54.08 |  |
| GNB4 | guanine nucleotide binding protein (G protein), beta polypeptide 4 | 223487_x_at | 1.10 | 59.80 |  | 0.40 | 4.45 | ↓ | 2.15 | 5.75 | ↑ |
| KPNA1 | karyopherin alpha 1 (importin alpha 5) | 202058_s_at | 1.10 | 61.53 |  | 0.41 | 0.53 | ↓ | 1.17 | 50.73 |  |
| HNRNPU | heterogeneous nuclear ribonucleoprotein U (scaffold attachment factor A) | 216855_s_at | 1.10 | 61.68 |  | 0.10 | 0.00 | ↓ | 0.61 | 5.75 |  |
| HMGB3P1 | high-mobility group box pseudogene 1 | 216548_x_at | 1.10 | 58.62 |  | 1.61 | 14.26 |  | 0.31 | 0.00 | ↓ |
| CLPB | ClpB caseinolytic peptidase B homolog (E. coli) | 221845_s_at | 1.10 | 54.53 |  | 0.83 | 29.01 |  | 0.30 | 0.00 | ↓ |
| XRN2 | 5'-3' exoribonuclease 2 | 233878_s_at | 1.10 | 60.45 |  | 0.44 | 0.76 | ↓ | 0.94 | 48.62 |  |
| DEDD | death effector domain containing | 211255_x_at | 1.10 | 51.95 |  | 0.41 | 0.60 | ↓ | 1.00 | 58.81 |  |
| ADAMTS5 | ADAM metallopeptidase with thrombospondin type 1 motif, 5 | 235368_at | 1.10 | 42.20 |  | 0.94 | 49.04 |  | 0.35 | 8.02 | ↓ |
| PLXNA4 | plexin A4 | 228104_at | 1.09 | 57.71 |  | 1.09 | 60.11 |  | 0.44 | 1.43 | ↓ |
| UQCC | ubiquinol-cytochrome c reductase complex chaperone | 222470_s_at | 1.09 | 59.48 |  | 0.31 | 0.60 | ↓ | 0.92 | 46.35 |  |
| HSD17B6 | hydroxysteroid (17-beta) dehydrogenase 6 homolog (mouse) | 205700_at | 1.09 | 62.00 |  | 2.22 | 11.08 |  | 0.12 | 0.72 | ↓ |
| KDSR | 3-ketodihydrosphingosine reductase | 229850_at | 1.09 | 51.95 |  | 2.06 | 1.98 | ↑ | 1.26 | 29.62 |  |
| ESYT2 | extended synaptotagmin-like protein 2 | 1555830_s_at | 1.09 | 60.72 |  | 0.21 | 0.00 | ↓ | 1.00 | 58.81 |  |
| CAPNS1 | calpain, small subunit 1 | 200001_at | 1.09 | 59.48 |  | 0.44 | 0.45 | ↓ | 0.91 | 35.63 |  |
| NXN | nucleoredoxin | 219489_s_at | 1.09 | 56.74 |  | 1.22 | 38.78 |  | 0.41 | 2.70 | ↓ |
| DCTN4 | dynactin 4 (p62) | 218013_x_at | 1.09 | 61.16 |  | 0.48 | 0.84 | ↓ | 1.11 | 50.73 |  |
| MBNL2 | muscleblind-like 2 (Drosophila) | 205017_s_at | 1.09 | 51.95 |  | 0.21 | 0.00 | ↓ | 1.43 | 26.48 |  |
| GALNTL2 | UDP-N-acetyl-alpha-D-galactosamine:polypeptide N-acetylgalactosaminyltransferase-like 2 | 236361_at | 1.09 | 58.62 |  | 0.93 | 38.78 |  | 0.50 | 8.02 | ↓ |
| MLPH | melanophilin | 218211_s_at | 1.09 | 54.53 |  | 0.81 | 41.29 |  | 0.35 | 0.89 | ↓ |
| CALU | calumenin | 200756_x_at | 1.09 | 59.48 |  | 0.31 | 0.25 | ↓ | 1.09 | 54.08 |  |
| LONRF1 | LON peptidase N-terminal domain and ring finger 1 | 226038_at | 1.09 | 57.71 |  | 1.46 | 15.95 |  | 0.45 | 0.34 | ↓ |
| SREK1 | splicing regulatory glutamine/lysine-rich protein 1 | 244287_at | 1.09 | 59.48 |  | 2.70 | 0.84 | ↑ | 1.02 | 57.64 |  |
| SCD | stearoyl-CoA desaturase (delta-9-desaturase) | 200832_s_at | 1.09 | 43.91 |  | 1.53 | 17.74 |  | 0.07 | 1.02 | ↓ |
| ZNF587 | zinc finger protein 587 | 1558253_x_at | 1.09 | 54.53 |  | 2.84 | 0.00 | ↑ | 1.23 | 43.83 |  |
| GEMIN4 | gem (nuclear organelle) associated protein 4 | 217099_s_at | 1.09 | 59.48 |  | 0.39 | 0.45 | ↓ | 0.99 | 50.73 |  |
| ANGPT1 | angiopoietin 1 | 205608_s_at | 1.09 | 50.55 |  | 0.38 | 3.35 | ↓ | 1.19 | 52.56 |  |
| C17orf101 | chromosome 17 open reading frame 101 | 219254_at | 1.09 | 59.48 |  | 1.11 | 49.04 |  | 0.43 | 0.72 | ↓ |
| HPS3 | Hermansky-Pudlak syndrome 3 | 238539_at | 1.09 | 62.00 |  | 0.31 | 0.00 | ↓ | 1.14 | 48.62 |  |
| LOH3CR2A | loss of heterozygosity, 3, chromosomal region 2, gene A | 220244_at | 1.09 | 56.74 |  | 1.14 | 49.04 |  | 2.43 | 1.25 | ↑ |
| SMPD1 | sphingomyelin phosphodiesterase 1, acid lysosomal | 216230_x_at | 1.09 | 57.71 |  | 0.25 | 0.00 | ↓ | 1.01 | 58.81 |  |
| C21orf91 | chromosome 21 open reading frame 91 | 226109_at | 1.09 | 57.71 |  | 2.04 | 6.80 | ↑ | 0.83 | 32.58 |  |
| CHD2 | Chromodomain helicase DNA binding protein 2 | 230156_x_at | 1.09 | 61.16 |  | 2.56 | 0.70 | ↑ | 1.63 | 15.27 |  |
| CHD9 | chromodomain helicase DNA binding protein 9 | 229586_at | 1.09 | 55.60 |  | 2.05 | 3.35 | ↑ | 0.97 | 54.08 |  |
| RWDD2B | RWD domain containing 2B | 222614_at | 1.09 | 56.74 |  | 0.48 | 1.26 | ↓ | 0.95 | 50.73 |  |
| CSGALNACT2 | chondroitin sulfate N-acetylgalactosaminyltransferase 2 | 239077_at | 1.09 | 61.53 |  | 0.09 | 0.00 | ↓ | 1.27 | 41.22 |  |
| HNRNPL | heterogeneous nuclear ribonucleoprotein L | 202072_at | 1.09 | 62.00 |  | 2.13 | 0.84 | ↑ | 0.84 | 20.38 |  |
| GTPBP2 | GTP binding protein 2 | 223789_s_at | 1.09 | 55.60 |  | 0.09 | 0.00 | ↓ | 0.50 | 8.02 |  |
| CSNK2A1 | casein kinase 2, alpha 1 polypeptide | 206075_s_at | 1.09 | 60.18 |  | 0.47 | 0.76 | ↓ | 0.75 | 15.27 |  |
| RANGAP1 | Ran GTPase activating protein 1 | 1553535_a_at | 1.08 | 62.00 |  | 0.31 | 0.25 | ↓ | 1.28 | 32.58 |  |
| GNB1L | guanine nucleotide binding protein (G protein), beta polypeptide 1-like | 220762_s_at | 1.08 | 59.48 |  | 0.49 | 0.98 | ↓ | 0.96 | 46.35 |  |
| KCNK2 | potassium channel, subfamily K, member 2 | 210261_at | 1.08 | 61.91 |  | 0.45 | 1.74 | ↓ | 1.15 | 52.56 |  |
| MAPK1IP1L | mitogen-activated protein kinase 1 interacting protein 1-like | 212497_at | 1.08 | 62.00 |  | 0.36 | 0.76 | ↓ | 1.01 | 58.81 |  |
| DAZAP1 | DAZ associated protein 1 | 218443_s_at | 1.08 | 54.53 |  | 2.05 | 1.07 | ↑ | 0.64 | 4.84 |  |
| DCP2 | DCP2 decapping enzyme homolog (S. cerevisiae) | 244777_at | 1.08 | 62.00 |  | 1.07 | 49.04 |  | 0.49 | 7.00 | ↓ |
| ORMDL3 | ORM1-like 3 (S. cerevisiae) | 223259_at | 1.08 | 59.06 |  | 0.75 | 10.02 |  | 2.24 | 0.43 | ↑ |
| PSEN1 | presenilin 1 | 207782_s_at | 1.08 | 59.48 |  | 0.34 | 0.00 | ↓ | 1.26 | 26.48 |  |
| CAB39 | calcium binding protein 39 | 224311_s_at | 1.08 | 58.62 |  | 0.29 | 0.00 | ↓ | 1.76 | 8.02 |  |
| LOC284513 | hypothetical protein LOC284513 | 1556597_a_at | 1.08 | 59.80 |  | 0.84 | 38.78 |  | 0.47 | 4.84 | ↓ |
| FAHD1 | fumarylacetoacetate hydrolase domain containing 1 | 226767_s_at | 1.08 | 55.60 |  | 2.35 | 0.76 | ↑ | 1.00 | 54.08 |  |
| FLNB | filamin B, beta | 208613_s_at | 1.08 | 58.62 |  | 0.88 | 57.71 |  | 0.27 | 0.50 | ↓ |
| SLC7A8 | solute carrier family 7 (amino acid transporter, L-type), member 8 | 216604_s_at | 1.08 | 57.71 |  | 0.29 | 6.11 | ↓ | 1.26 | 48.62 |  |
| SLC43A3 | solute carrier family 43, member 3 | 210692_s_at | 1.08 | 56.74 |  | 0.37 | 0.45 | ↓ | 1.01 | 54.08 |  |
| PHGDH | phosphoglycerate dehydrogenase | 201397_at | 1.08 | 43.91 |  | 0.90 | 53.46 |  | 0.24 | 1.99 | ↓ |
| C18orf25 | chromosome 18 open reading frame 25 | 217539_at | 1.08 | 59.06 |  | 0.24 | 0.40 | ↓ | 1.08 | 52.56 |  |
| SLC11A2 | solute carrier family 11 (proton-coupled divalent metal ion transporters), member 2 | 203125_x_at | 1.08 | 59.80 |  | 0.42 | 0.25 | ↓ | 1.77 | 7.00 |  |
| CASC5 | cancer susceptibility candidate 5 | 1552682_a_at | 1.08 | 54.53 |  | 0.50 | 6.80 | ↓ | 0.57 | 11.20 |  |
| FLJ42709 | hypothetical LOC441094 | 1556696_s_at | 1.08 | 59.06 |  | 2.15 | 3.81 | ↑ | 1.35 | 35.63 |  |
| SLC8A1 | solute carrier family 8 (sodium/calcium exchanger), member 1 | 241752_at | 1.08 | 61.35 |  | 0.43 | 0.53 | ↓ | 0.94 | 46.35 |  |
| MCAM | melanoma cell adhesion molecule | 211340_s_at | 1.08 | 50.55 |  | 0.95 | 60.11 |  | 0.41 | 5.75 | ↓ |
| PI4K2A | phosphatidylinositol 4-kinase type 2 alpha | 209346_s_at | 1.08 | 61.68 |  | 0.35 | 0.45 | ↓ | 1.37 | 35.63 |  |
| CD36 | CD36 molecule (thrombospondin receptor) | 228766_at | 1.08 | 48.99 |  | 1.19 | 41.29 |  | 7.14 | 7.00 | ↑ |
| NUP188 | nucleoporin 188kDa | 212691_at | 1.08 | 57.71 |  | 0.41 | 0.53 | ↓ | 0.90 | 38.27 |  |
| TXNRD1 | thioredoxin reductase 1 | 201266_at | 1.08 | 59.80 |  | 1.22 | 31.53 |  | 2.60 | 0.72 | ↑ |
| MBTPS2 | membrane-bound transcription factor peptidase, site 2 | 206473_at | 1.08 | 53.22 |  | 0.24 | 0.00 | ↓ | 1.33 | 29.62 |  |
| GNG12 | guanine nucleotide binding protein (G protein), gamma 12 | 1555240_s_at | 1.08 | 61.35 |  | 1.33 | 29.01 |  | 2.14 | 2.43 | ↑ |
| MEF2A | myocyte enhancer factor 2A | 208328_s_at | 1.08 | 62.00 |  | 3.73 | 0.00 | ↑ | 1.75 | 3.36 |  |
| LOC100506748 | hypothetical LOC100506748 | 226381_at | 1.08 | 62.00 |  | 1.39 | 19.82 |  | 2.54 | 0.00 | ↑ |
| WASF2 | WAS protein family, member 2 | 224563_at | 1.07 | 58.62 |  | 7.20 | 0.14 | ↑ | — | — |  |
| CLIP4 | CAP-GLY domain containing linker protein family, member 4 | 219944_at | 1.07 | 58.62 |  | 0.48 | 1.07 | ↓ | 1.21 | 38.27 |  |
| RAB27B | RAB27B, member RAS oncogene family | 228708_at | 1.07 | 50.55 |  | 0.58 | 17.74 |  | 0.15 | 0.43 | ↓ |
| PGD | phosphogluconate dehydrogenase | 201118_at | 1.07 | 60.45 |  | 0.49 | 0.76 | ↓ | 0.61 | 4.84 |  |
| ZFYVE1 | zinc finger, FYVE domain containing 1 | 223388_s_at | 1.07 | 59.06 |  | 0.45 | 0.45 | ↓ | 0.99 | 58.81 |  |
| GBA2 | glucosidase, beta (bile acid) 2 | 223921_s_at | 1.07 | 55.60 |  | 0.34 | 0.18 | ↓ | 0.83 | 20.38 |  |
| GGA2 | golgi-associated, gamma adaptin ear containing, ARF binding protein 2 | 208915_s_at | 1.07 | 59.06 |  | 0.46 | 1.26 | ↓ | 0.91 | 41.22 |  |
| AASDH | aminoadipate-semialdehyde dehydrogenase | 235435_at | 1.07 | 61.35 |  | 2.06 | 1.98 | ↑ | 0.83 | 26.48 |  |
| FAM126B | family with sequence similarity 126, member B | 1558959_at | 1.07 | 62.00 |  | 0.24 | 0.40 | ↓ | 1.00 | 54.08 |  |
| ATP8B1 | ATPase, aminophospholipid transporter, class I, type 8B, member 1 | 226302_at | 1.07 | 61.53 |  | 1.56 | 11.08 |  | 2.11 | 1.43 | ↑ |
| JAG1 | Jagged 1 (Alagille syndrome) | 231183_s_at | 1.07 | 62.00 |  | 3.21 | 1.26 | ↑ | 0.54 | 5.75 |  |
| DCUN1D5 | DCN1, defective in cullin neddylation 1, domain containing 5 (S. cerevisiae) | 223151_at | 1.07 | 56.74 |  | 2.05 | 2.80 | ↑ | 0.84 | 29.62 |  |
| FLJ39051 | hypothetical LOC399972 | 227925_at | 1.07 | 58.62 |  | 0.84 | 33.65 |  | 0.48 | 2.43 | ↓ |
| SLC44A1 | solute carrier family 44, member 1 | 222364_at | 1.07 | 55.60 |  | 0.76 | 41.29 |  | 0.46 | 7.00 | ↓ |
| GTSE1 | G-2 and S-phase expressed 1 | 204317_at | 1.07 | 58.62 |  | 0.75 | 22.17 |  | 0.17 | 0.00 | ↓ |
| OPA1 | optic atrophy 1 (autosomal dominant) | 241027_at | 1.07 | 62.00 |  | 0.23 | 0.25 | ↓ | 0.99 | 54.08 |  |
| OSBPL6 | oxysterol binding protein-like 6 | 223805_at | 1.07 | 59.80 |  | 1.31 | 24.54 |  | 0.35 | 0.58 | ↓ |
| UBL7 | ubiquitin-like 7 (bone marrow stromal cell-derived) | 225063_at | 1.07 | 58.62 |  | 0.43 | 0.18 | ↓ | 0.93 | 41.22 |  |
| SSR1 | signal sequence receptor, alpha | 226712_at | 1.07 | 60.45 |  | 2.06 | 1.74 | ↑ | 1.05 | 58.81 |  |
| FGFRL1 | fibroblast growth factor receptor-like 1 | 223321_s_at | 1.07 | 54.53 |  | 0.29 | 0.25 | ↓ | 1.23 | 54.08 |  |
| NUP98 | nucleoporin 98kDa | 203194_s_at | 1.07 | 60.45 |  | 0.34 | 0.00 | ↓ | 0.96 | 54.08 |  |
| FOLR1 | folate receptor 1 (adult) | 211074_at | 1.07 | 56.74 |  | 0.38 | 1.26 | ↓ | 2.26 | 4.16 | ↑ |
| IGFBP5 | insulin-like growth factor binding protein 5 | 211959_at | 1.07 | 42.20 |  | 1.70 | 26.77 |  | 3.92 | 4.84 | ↑ |
| H2BFXP | H2B histone family, member X, pseudogene | 230664_at | 1.07 | 38.54 |  | 1.41 | 33.65 |  | 0.18 | 0.43 | ↓ |
| C7orf64 | chromosome 7 open reading frame 64 | 232661_s_at | 1.07 | 62.00 |  | 0.49 | 0.70 | ↓ | 1.11 | 57.92 |  |
| MEGF6 | multiple EGF-like-domains 6 | 226869_at | 1.07 | 60.45 |  | 2.33 | 6.11 | ↑ | 0.88 | 38.27 |  |
| SNX13 | sorting nexin 13 | 215820_x_at | 1.07 | 61.68 |  | 0.46 | 4.45 | ↓ | 1.14 | 52.56 |  |
| SDAD1 | SDA1 domain containing 1 | 228408_s_at | 1.07 | 58.62 |  | 2.74 | 0.40 | ↑ | 1.01 | 58.81 |  |
| OSBPL8 | oxysterol binding protein-like 8 | 228986_at | 1.06 | 61.79 |  | 1.53 | 12.66 |  | 5.26 | 1.99 | ↑ |
| CDC25C | cell division cycle 25 homolog C (S. pombe) | 217010_s_at | 1.06 | 59.48 |  | 0.51 | 24.54 |  | 0.23 | 3.36 | ↓ |
| GALC | galactosylceramidase | 211810_s_at | 1.06 | 60.45 |  | 0.46 | 0.70 | ↓ | 0.95 | 48.62 |  |
| HFE | hemochromatosis | 206086_x_at | 1.06 | 61.16 |  | 0.49 | 0.76 | ↓ | 1.02 | 58.81 |  |
| GTF2I | general transcription factor IIi | 210892_s_at | 1.06 | 58.62 |  | 0.14 | 0.00 | ↓ | 1.06 | 56.10 |  |
| IL1R1 | interleukin 1 receptor, type I | 215561_s_at | 1.06 | 62.00 |  | 0.32 | 0.00 | ↓ | 1.36 | 17.70 |  |
| PLXNA4 | plexin A4 | 232317_at | 1.06 | 58.62 |  | 1.48 | 43.50 |  | 0.45 | 1.02 | ↓ |
| KIAA1109 | KIAA1109 | 214929_s_at | 1.06 | 60.18 |  | 0.43 | 6.11 | ↓ | 1.53 | 17.70 |  |
| KLHDC3 | kelch domain containing 3 | 214383_x_at | 1.06 | 59.48 |  | 0.40 | 0.25 | ↓ | 1.21 | 32.58 |  |
| TGM2 | transglutaminase 2 (C polypeptide, protein-glutamine-gamma-glutamyltransferase) | 211003_x_at | 1.06 | 53.22 |  | 0.18 | 0.00 | ↓ | 1.14 | 54.85 |  |
| UBE4B | ubiquitination factor E4B (UFD2 homolog, yeast) | 202316_x_at | 1.06 | 62.00 |  | 0.21 | 0.25 | ↓ | 1.38 | 15.27 |  |
| GLUL | glutamate-ammonia ligase | 217202_s_at | 1.06 | 60.45 |  | 0.37 | 0.00 | ↓ | 1.29 | 29.62 |  |
| CNOT1 | CCR4-NOT transcription complex, subunit 1 | 200861_at | 1.06 | 59.06 |  | 0.28 | 0.18 | ↓ | 1.19 | 38.27 |  |
| CCDC59 | coiled-coil domain containing 59 | 222792_s_at | 1.06 | 58.62 |  | 2.06 | 0.84 | ↑ | 0.80 | 9.54 |  |
| PIK3R1 | phosphoinositide-3-kinase, regulatory subunit 1 (alpha) | 212239_at | 1.06 | 59.06 |  | 2.23 | 3.81 | ↑ | 3.80 | 0.31 | ↑ |
| BMP6 | bone morphogenetic protein 6 | 206176_at | 1.06 | 55.60 |  | 0.50 | 11.08 |  | 2.26 | 7.00 | ↑ |
| CENPV | centromere protein V | 226610_at | 1.06 | 58.62 |  | 0.69 | 55.85 |  | 0.24 | 4.84 | ↓ |
| C1orf25 | chromosome 1 open reading frame 25 | 233750_s_at | 1.06 | 59.06 |  | 0.50 | 4.45 | ↓ | 0.83 | 41.22 |  |
| C1orf104 | Chromosome 1 open reading frame 104 | 230256_at | 1.06 | 53.22 |  | 2.19 | 2.80 | ↑ | 0.49 | 1.02 | ↓ |
| PCSK5 | proprotein convertase subtilisin/kexin type 5 | 205559_s_at | 1.06 | 53.22 |  | 6.63 | 0.40 | ↑ | 0.81 | 38.27 |  |
| PTPLAD2 | protein tyrosine phosphatase-like A domain containing 2 | 244050_at | 1.06 | 60.72 |  | 1.26 | 36.20 |  | 0.47 | 1.25 | ↓ |
| ERI3 | ERI1 exoribonuclease family member 3 | 208973_at | 1.06 | 60.72 |  | 0.22 | 0.00 | ↓ | 0.95 | 52.56 |  |
| ARHGAP18 | Rho GTPase activating protein 18 | 225166_at | 1.06 | 57.71 |  | 1.17 | 49.04 |  | 2.10 | 2.43 | ↑ |
| NETO2 | neuropilin (NRP) and tolloid (TLL)-like 2 | 222774_s_at | 1.06 | 62.00 |  | 0.63 | 22.17 |  | 0.28 | 2.70 | ↓ |
| UBE2S | ubiquitin-conjugating enzyme E2S | 202779_s_at | 1.06 | 60.45 |  | 0.69 | 17.74 |  | 0.45 | 0.72 | ↓ |
| TUBGCP2 | tubulin, gamma complex associated protein 2 | 202476_s_at | 1.06 | 60.45 |  | 0.29 | 0.40 | ↓ | 0.96 | 54.08 |  |
| UNC5C | unc-5 homolog C (C. elegans) | 206189_at | 1.05 | 54.53 |  | 1.15 | 47.13 |  | 0.44 | 8.02 | ↓ |
| DIP2A | DIP2 disco-interacting protein 2 homolog A (Drosophila) | 1561286_a_at | 1.05 | 54.53 |  | 1.07 | 59.12 |  | 0.45 | 7.00 | ↓ |
| ASB8 | ankyrin repeat and SOCS box-containing 8 | 218841_at | 1.05 | 56.74 |  | 2.03 | 3.81 | ↑ | 1.61 | 20.38 |  |
| DRG2 | developmentally regulated GTP binding protein 2 | 203267_s_at | 1.05 | 60.94 |  | 0.44 | 0.25 | ↓ | 0.80 | 15.27 |  |
| OBSL1 | obscurin-like 1 | 212776_s_at | 1.05 | 59.06 |  | 0.35 | 0.60 | ↓ | 0.91 | 50.73 |  |
| POMGNT1 | protein O-linked mannose beta1,2-N-acetylglucosaminyltransferase | 233638_s_at | 1.05 | 62.00 |  | 0.36 | 0.76 | ↓ | 1.00 | 57.92 |  |
| L3MBTL2 | l(3)mbt-like 2 (Drosophila) | 1555815_a_at | 1.05 | 59.80 |  | 0.37 | 0.60 | ↓ | 1.09 | 52.56 |  |
| CEP290 | centrosomal protein 290kDa | 205250_s_at | 1.05 | 60.45 |  | 2.09 | 1.74 | ↑ | 1.20 | 50.73 |  |
| EIF5A2 | eukaryotic translation initiation factor 5A2 | 220198_s_at | 1.05 | 62.00 |  | 0.93 | 49.04 |  | 2.49 | 1.61 | ↑ |
| WNT3 | wingless-type MMTV integration site family, member 3 | 229103_at | 1.05 | 57.71 |  | 1.94 | 15.95 |  | 2.09 | 8.02 | ↑ |
| EPOR | erythropoietin receptor | 215054_at | 1.05 | 59.06 |  | 1.30 | 41.29 |  | 0.15 | 0.00 | ↓ |
| PRPF31 | PRP31 pre-mRNA processing factor 31 homolog (S. cerevisiae) | 202407_s_at | 1.05 | 59.48 |  | 0.46 | 0.98 | ↓ | 1.07 | 56.65 |  |
| C5orf24 | chromosome 5 open reading frame 24 | 1553107_s_at | 1.05 | 57.71 |  | 2.55 | 0.70 | ↑ | 1.13 | 54.08 |  |
| WDR20 | WD repeat domain 20 | 227693_at | 1.05 | 61.35 |  | 1.13 | 43.50 |  | 0.47 | 0.43 | ↓ |
| LAMP1 | lysosomal-associated membrane protein 1 | 201551_s_at | 1.05 | 60.45 |  | 0.34 | 0.18 | ↓ | 0.96 | 52.56 |  |
| TNPO3 | transportin 3 | 212317_at | 1.05 | 60.94 |  | 2.12 | 0.84 | ↑ | 0.74 | 5.75 |  |
| ABCF2 | ATP-binding cassette, sub-family F (GCN20), member 2 | 207622_s_at | 1.05 | 61.79 |  | 0.45 | 0.40 | ↓ | 1.07 | 54.08 |  |
| CLINT1 | clathrin interactor 1 | 201768_s_at | 1.05 | 60.18 |  | 0.46 | 0.25 | ↓ | 1.17 | 32.58 |  |
| SLC43A2 | solute carrier family 43, member 2 | 226629_at | 1.05 | 62.00 |  | 1.32 | 29.01 |  | 2.02 | 4.16 | ↑ |
| KIAA1644 | KIAA1644 | 52837_at | 1.05 | 58.62 |  | 1.03 | 58.42 |  | 0.40 | 1.02 | ↓ |
| FYTTD1 | forty-two-three domain containing 1 | 224642_at | 1.05 | 62.00 |  | 0.39 | 0.25 | ↓ | 1.50 | 7.00 |  |
| DNALI1 | dynein, axonemal, light intermediate chain 1 | 205186_at | 1.05 | 60.94 |  | 0.66 | 5.22 |  | 0.35 | 0.43 | ↓ |
| CCDC117 | coiled-coil domain containing 117 | 235330_at | 1.05 | 58.62 |  | 0.48 | 1.07 | ↓ | 0.85 | 35.63 |  |
| ANK2 | ankyrin 2, neuronal | 202921_s_at | 1.05 | 48.99 |  | 0.26 | 0.40 | ↓ | 1.58 | 15.27 |  |
| NCAM1 | neural cell adhesion molecule 1 | 227394_at | 1.05 | 56.74 |  | 0.58 | 29.01 |  | 0.38 | 8.02 | ↓ |
| MBTPS1 | membrane-bound transcription factor peptidase, site 1 | 217543_s_at | 1.05 | 59.80 |  | 0.28 | 0.00 | ↓ | 1.04 | 56.10 |  |
| DNAJC13 | DnaJ (Hsp40) homolog, subfamily C, member 13 | 1560020_at | 1.05 | 57.71 |  | 0.31 | 0.76 | ↓ | 1.47 | 23.22 |  |
| BAG5 | BCL2-associated athanogene 5 | 202984_s_at | 1.05 | 60.45 |  | 2.22 | 1.45 | ↑ | 1.14 | 43.83 |  |
| FAM101B | family with sequence similarity 101, member B | 226876_at | 1.05 | 62.00 |  | 2.12 | 6.80 | ↑ | 1.37 | 35.63 |  |
| MAN1A1 | mannosidase, alpha, class 1A, member 1 | 208116_s_at | 1.04 | 51.95 |  | 0.32 | 0.25 | ↓ | 1.80 | 11.20 |  |
| MDC1 | mediator of DNA-damage checkpoint 1 | 203061_s_at | 1.04 | 61.79 |  | 0.19 | 0.25 | ↓ | 0.41 | 1.02 | ↓ |
| SH3GL1 | SH3-domain GRB2-like 1 | 201851_at | 1.04 | 59.48 |  | 0.46 | 0.76 | ↓ | 0.78 | 20.38 |  |
| GSK3B | glycogen synthase kinase 3 beta | 242336_at | 1.04 | 59.06 |  | 0.24 | 0.25 | ↓ | 1.38 | 26.48 |  |
| --- | --- | 242870_at | 1.04 | 62.00 |  | 0.24 | 0.00 | ↓ | 0.89 | 38.27 |  |
| JAM3 | junctional adhesion molecule 3 | 231720_s_at | 1.04 | 56.74 |  | 0.22 | 0.36 | ↓ | 1.21 | 43.83 |  |
| FOXC1 | forkhead box C1 | 1553613_s_at | 1.04 | 60.18 |  | 4.07 | 0.21 | ↑ | 0.37 | 0.89 | ↓ |
| CREB1 | cAMP responsive element binding protein 1 | 204312_x_at | 1.04 | 62.00 |  | 0.41 | 0.18 | ↓ | 0.94 | 46.35 |  |
| CCL26 | chemokine (C-C motif) ligand 26 | 223710_at | 1.04 | 59.48 |  | 0.76 | 47.13 |  | 0.45 | 9.54 | ↓ |
| CXCR7 | chemokine (C-X-C motif) receptor 7 | 212977_at | 1.04 | 40.42 |  | 2.12 | 9.01 | ↑ | 0.31 | 7.00 | ↓ |
| TYMP | thymidine phosphorylase | 204858_s_at | 1.04 | 47.41 |  | 0.67 | 19.82 |  | 0.38 | 1.25 | ↓ |
| C20orf27 | chromosome 20 open reading frame 27 | 50314_i_at | 1.04 | 59.06 |  | 0.46 | 2.30 | ↓ | 0.72 | 23.22 |  |
| EEA1 | early endosome antigen 1 | 204840_s_at | 1.04 | 55.60 |  | 2.65 | 4.45 | ↑ | 0.97 | 46.35 |  |
| CSRNP2 | cysteine-serine-rich nuclear protein 2 | 225042_s_at | 1.04 | 61.35 |  | 0.30 | 0.36 | ↓ | 0.97 | 58.81 |  |
| RPS23 | ribosomal protein S23 | 227722_at | 1.04 | 58.62 |  | 0.45 | 9.01 | ↓ | 1.96 | 11.20 |  |
| FRY | furry homolog (Drosophila) | 214318_s_at | 1.04 | 59.80 |  | 0.62 | 17.74 |  | 2.21 | 2.43 | ↑ |
| ELF1 | E74-like factor 1 (ets domain transcription factor) | 212420_at | 1.04 | 62.00 |  | 2.00 | 2.30 | ↑ | 1.22 | 43.83 |  |
| ATP6V1A | ATPase, H+ transporting, lysosomal 70kDa, V1 subunit A | 201971_s_at | 1.04 | 53.22 |  | 0.21 | 0.00 | ↓ | 1.79 | 1.61 |  |
| C16orf75 | chromosome 16 open reading frame 75 | 226456_at | 1.04 | 62.00 |  | 0.78 | 22.17 |  | 0.32 | 0.00 | ↓ |
| AKAP2 /// PALM2-AKAP2 | A kinase (PRKA) anchor protein 2 /// PALM2-AKAP2 readthrough | 226694_at | 1.04 | 61.16 |  | 1.15 | 36.20 |  | 2.01 | 0.58 | ↑ |
| DDR2 | Discoidin domain receptor tyrosine kinase 2 | 235631_at | 1.04 | 55.60 |  | 0.21 | 0.00 | ↓ | 1.28 | 29.62 |  |
| C1QTNF6 | C1q and tumor necrosis factor related protein 6 | 242444_at | 1.04 | 62.00 |  | 0.27 | 0.00 | ↓ | 0.72 | 15.27 |  |
| UBE3C | ubiquitin protein ligase E3C | 1554794_a_at | 1.04 | 58.62 |  | 0.36 | 0.40 | ↓ | 1.17 | 43.83 |  |
| ANK3 | ankyrin 3, node of Ranvier (ankyrin G) | 209442_x_at | 1.04 | 62.00 |  | 3.74 | 0.36 | ↑ | 1.91 | 29.62 |  |
| CSRP2 | cysteine and glycine-rich protein 2 | 207030_s_at | 1.04 | 53.22 |  | 0.89 | 38.78 |  | 2.03 | 2.70 | ↑ |
| RDH10 | retinol dehydrogenase 10 (all-trans) | 1552378_s_at | 1.04 | 60.45 |  | 0.97 | 57.71 |  | 3.24 | 3.36 | ↑ |
| ZFR | zinc finger RNA binding protein | 213286_at | 1.04 | 62.00 |  | 0.29 | 0.18 | ↓ | 1.19 | 38.27 |  |
| TNFSF11 | tumor necrosis factor (ligand) superfamily, member 11 | 210643_at | 1.04 | 53.22 |  | 0.71 | 58.42 |  | 0.16 | 5.75 | ↓ |
| SRPX2 | sushi-repeat-containing protein, X-linked 2 | 205499_at | 1.04 | 62.00 |  | 0.71 | 17.74 |  | 0.36 | 0.00 | ↓ |
| MAP4K5 | mitogen-activated protein kinase kinase kinase kinase 5 | 211081_s_at | 1.04 | 62.00 |  | 0.40 | 0.84 | ↓ | 0.96 | 58.81 |  |
| ATG13 | ATG13 autophagy related 13 homolog (S. cerevisiae) | 209021_x_at | 1.04 | 62.00 |  | 0.41 | 0.45 | ↓ | 0.81 | 23.22 |  |
| KIAA1147 | KIAA1147 | 223161_at | 1.03 | 61.68 |  | 0.46 | 1.98 | ↓ | 1.05 | 57.14 |  |
| GTDC1 | glycosyltransferase-like domain containing 1 | 238585_at | 1.03 | 62.00 |  | 0.45 | 0.40 | ↓ | 1.09 | 54.85 |  |
| UTRN | utrophin | 213023_at | 1.03 | 61.96 |  | 0.34 | 0.00 | ↓ | 1.55 | 8.02 |  |
| DGKA | diacylglycerol kinase, alpha 80kDa | 203385_at | 1.03 | 62.00 |  | 0.44 | 0.25 | ↓ | 0.72 | 11.20 |  |
| DLG1 | discs, large homolog 1 (Drosophila) | 202516_s_at | 1.03 | 61.96 |  | 0.40 | 0.45 | ↓ | 1.01 | 58.77 |  |
| SLC20A2 | solute carrier family 20 (phosphate transporter), member 2 | 202744_at | 1.03 | 51.95 |  | 0.55 | 2.80 |  | 2.01 | 1.02 | ↑ |
| TNFRSF11B | tumor necrosis factor receptor superfamily, member 11b | 204932_at | 1.03 | 61.79 |  | 1.12 | 50.64 |  | 2.08 | 1.25 | ↑ |
| SFRS15 | Splicing factor, arginine/serine-rich 15 | 243759_at | 1.03 | 61.53 |  | 0.41 | 2.30 | ↓ | 0.79 | 35.63 |  |
| NOD1 | nucleotide-binding oligomerization domain containing 1 | 224190_x_at | 1.03 | 62.00 |  | 0.47 | 1.45 | ↓ | 0.85 | 35.63 |  |
| DDX3X | DEAD (Asp-Glu-Ala-Asp) box polypeptide 3, X-linked | 201211_s_at | 1.03 | 59.06 |  | 0.34 | 1.26 | ↓ | 1.40 | 23.22 |  |
| SUDS3 | suppressor of defective silencing 3 homolog (S. cerevisiae) | 224974_at | 1.03 | 60.72 |  | 2.03 | 1.26 | ↑ | 0.94 | 46.35 |  |
| MAP2K3 | mitogen-activated protein kinase kinase 3 | 215498_s_at | 1.03 | 62.00 |  | 1.02 | 55.85 |  | 2.05 | 0.72 | ↑ |
| SEC14L1 | SEC14-like 1 (S. cerevisiae) | 202082_s_at | 1.03 | 60.45 |  | 2.66 | 1.26 | ↑ | 0.94 | 54.08 |  |
| SMEK1 | SMEK homolog 1, suppressor of mek1 (Dictyostelium) | 220368_s_at | 1.03 | 61.91 |  | 0.50 | 0.76 | ↓ | 0.70 | 8.02 |  |
| CCDC88A | coiled-coil domain containing 88A | 221078_s_at | 1.03 | 58.62 |  | 0.42 | 0.53 | ↓ | 1.03 | 58.81 |  |
| C5orf41 | chromosome 5 open reading frame 41 | 1554229_at | 1.03 | 61.35 |  | 0.48 | 0.76 | ↓ | 1.62 | 5.75 |  |
| FGD4 | FYVE, RhoGEF and PH domain containing 4 | 242445_at | 1.03 | 43.91 |  | 0.51 | 58.42 |  | 3.91 | 5.75 | ↑ |
| TTC37 | tetratricopeptide repeat domain 37 | 1554029_a_at | 1.03 | 53.22 |  | 0.25 | 0.00 | ↓ | 1.52 | 13.22 |  |
| CACNA2D1 | calcium channel, voltage-dependent, alpha 2/delta subunit 1 | 207050_at | 1.03 | 62.00 |  | 0.41 | 2.30 | ↓ | 1.71 | 38.27 |  |
| C15orf29 | chromosome 15 open reading frame 29 | 232094_at | 1.03 | 57.71 |  | 1.17 | 53.46 |  | 0.45 | 3.36 | ↓ |
| SLC39A13 | solute carrier family 39 (zinc transporter), member 13 | 1552295_a_at | 1.03 | 59.06 |  | 0.39 | 0.98 | ↓ | 0.73 | 26.48 |  |
| --- | --- | 230502_s_at | 1.03 | 60.45 |  | 0.31 | 0.53 | ↓ | 0.65 | 17.70 |  |
| STRBP | spermatid perinuclear RNA binding protein | 223245_at | 1.03 | 45.69 |  | 1.14 | 43.50 |  | 0.48 | 2.43 | ↓ |
| ANKH | ankylosis, progressive homolog (mouse) | 223094_s_at | 1.03 | 58.62 |  | 0.29 | 0.25 | ↓ | 0.85 | 38.27 |  |
| TET3 | tet oncogene family member 3 | 235542_at | 1.03 | 61.68 |  | 2.74 | 3.81 | ↑ | 1.04 | 57.92 |  |
| SYCP3 | synaptonemal complex protein 3 | 1553599_a_at | 1.03 | 34.35 |  | 0.47 | 1.98 | ↓ | 1.07 | 54.08 |  |
| ARHGAP24 | Rho GTPase activating protein 24 | 230803_s_at | 1.03 | 58.62 |  | 0.27 | 0.00 | ↓ | 1.31 | 32.58 |  |
| RAP1GDS1 | RAP1, GTP-GDP dissociation stimulator 1 | 229905_at | 1.03 | 62.00 |  | 0.21 | 0.00 | ↓ | 1.18 | 48.62 |  |
| TOB1 | transducer of ERBB2, 1 | 228834_at | 1.03 | 62.00 |  | 0.33 | 0.00 | ↓ | 1.38 | 11.20 |  |
| IKBKB | inhibitor of kappa light polypeptide gene enhancer in B-cells, kinase beta | 211027_s_at | 1.03 | 51.95 |  | 0.28 | 0.25 | ↓ | 1.17 | 43.83 |  |
| ARMCX4 | armadillo repeat containing, X-linked 4 | 1552327_at | 1.03 | 59.48 |  | 0.72 | 41.29 |  | 0.39 | 5.75 | ↓ |
| ATP6V1C1 | ATPase, H+ transporting, lysosomal 42kDa, V1 subunit C1 | 202873_at | 1.03 | 58.62 |  | 0.23 | 0.00 | ↓ | 1.87 | 13.22 |  |
| MTF1 | metal-regulatory transcription factor 1 | 205322_s_at | 1.03 | 58.62 |  | 0.41 | 0.29 | ↓ | 1.22 | 23.22 |  |
| DIO2 | deiodinase, iodothyronine, type II | 231240_at | 1.03 | 60.18 |  | 0.23 | 0.25 | ↓ | 0.51 | 4.16 |  |
| LEPR | leptin receptor | 209894_at | 1.03 | 59.48 |  | 2.10 | 7.87 | ↑ | 0.66 | 35.63 |  |
| HSP90AB1 | heat shock protein 90kDa alpha (cytosolic), class B member 1 | 1557910_at | 1.03 | 54.53 |  | 0.24 | 0.00 | ↓ | 0.88 | 35.63 |  |
| --- | --- | 223661_at | 1.03 | 53.22 |  | 0.40 | 0.84 | ↓ | 1.56 | 29.62 |  |
| KLHDC5 | kelch domain containing 5 | 225961_at | 1.03 | 62.00 |  | 2.42 | 0.70 | ↑ | 1.08 | 54.08 |  |
| ASAH1 | N-acylsphingosine amidohydrolase (acid ceramidase) 1 | 1555419_a_at | 1.03 | 61.35 |  | 0.49 | 1.98 | ↓ | 1.58 | 9.54 |  |
| SLC1A1 | solute carrier family 1 (neuronal/epithelial high affinity glutamate transporter, system Xag), member 1 | 206396_at | 1.03 | 54.53 |  | 0.16 | 0.00 | ↓ | 2.23 | 0.72 | ↑ |
| CCNI | Cyclin I | 227299_at | 1.03 | 60.94 |  | 2.97 | 0.45 | ↑ | 1.30 | 32.58 |  |
| MZF1 | myeloid zinc finger 1 | 210336_x_at | 1.02 | 62.00 |  | 1.03 | 56.87 |  | 0.46 | 2.43 | ↓ |
| PRNP | prion protein | 215707_s_at | 1.02 | 61.16 |  | 0.40 | 1.26 | ↓ | 1.08 | 52.56 |  |
| ST6GAL1 | ST6 beta-galactosamide alpha-2,6-sialyltranferase 1 | 201998_at | 1.02 | 58.62 |  | 1.65 | 14.26 |  | 0.43 | 3.36 | ↓ |
| VHL | von Hippel-Lindau tumor suppressor | 1559227_s_at | 1.02 | 62.00 |  | 2.20 | 0.98 | ↑ | 1.13 | 54.85 |  |
| PDPK1 | 3-phosphoinositide dependent protein kinase-1 | 204524_at | 1.02 | 62.00 |  | 2.00 | 1.74 | ↑ | 1.13 | 50.73 |  |
| SCRN3 | secernin 3 | 232108_at | 1.02 | 47.41 |  | 0.43 | 1.98 | ↓ | 0.88 | 32.58 |  |
| PRKACB | protein kinase, cAMP-dependent, catalytic, beta | 235780_at | 1.02 | 62.00 |  | 1.09 | 47.13 |  | 0.49 | 2.43 | ↓ |
| CYP26B1 | cytochrome P450, family 26, subfamily B, polypeptide 1 | 219825_at | 1.02 | 50.55 |  | 1.56 | 29.01 |  | 8.47 | 0.50 | ↑ |
| ZDBF2 | zinc finger, DBF-type containing 2 | 228749_at | 1.02 | 59.06 |  | 3.64 | 0.84 | ↑ | 0.78 | 41.22 |  |
| ASPH | aspartate beta-hydroxylase | 205808_at | 1.02 | 57.71 |  | 0.13 | 0.00 | ↓ | 0.83 | 32.58 |  |
| TMTC1 | transmembrane and tetratricopeptide repeat containing 1 | 226322_at | 1.02 | 61.79 |  | 0.50 | 11.08 |  | 0.44 | 3.36 | ↓ |
| LAMA4 | laminin, alpha 4 | 210990_s_at | 1.02 | 62.00 |  | 0.46 | 3.35 | ↓ | 0.79 | 26.48 |  |
| ZNF137P | zinc finger protein 137, pseudogene | 207394_at | 1.02 | 56.74 |  | 1.17 | 41.29 |  | 0.49 | 1.99 | ↓ |
| CCNT2 | cyclin T2 | 214638_s_at | 1.02 | 58.62 |  | 0.49 | 1.74 | ↓ | 1.24 | 52.56 |  |
| ASF1B | ASF1 anti-silencing function 1 homolog B (S. cerevisiae) | 218115_at | 1.02 | 62.00 |  | 0.51 | 6.11 |  | 0.21 | 0.50 | ↓ |
| CDK17 | cyclin-dependent kinase 17 | 206474_at | 1.02 | 54.53 |  | 0.32 | 0.00 | ↓ | 1.12 | 52.56 |  |
| OTUB1 | OTU domain, ubiquitin aldehyde binding 1 | 201246_s_at | 1.02 | 62.00 |  | 0.28 | 0.40 | ↓ | 1.01 | 57.64 |  |
| XG | Xg blood group | 241931_at | 1.02 | 47.41 |  | 0.68 | 14.26 |  | 3.28 | 4.84 | ↑ |
| YTHDF3 | YTH domain family, member 3 | 1564053_a_at | 1.02 | 53.22 |  | 0.25 | 0.18 | ↓ | 1.58 | 9.54 |  |
| SOCS3 | suppressor of cytokine signaling 3 | 227697_at | 1.02 | 60.72 |  | 1.31 | 29.01 |  | 0.45 | 5.75 | ↓ |
| MATR3 | matrin 3 | 1558093_s_at | 1.02 | 58.62 |  | 0.27 | 0.00 | ↓ | 1.05 | 57.92 |  |
| PRKG1 | protein kinase, cGMP-dependent, type I | 228396_at | 1.02 | 62.00 |  | 0.90 | 41.29 |  | 2.60 | 0.72 | ↑ |
| SVIL | supervillin | 202565_s_at | 1.02 | 56.74 |  | 0.88 | 60.64 |  | 2.05 | 9.54 | ↑ |
| KBTBD2 | kelch repeat and BTB (POZ) domain containing 2 | 223585_x_at | 1.02 | 62.00 |  | 0.34 | 0.25 | ↓ | 1.01 | 58.81 |  |
| SOD2 | superoxide dismutase 2, mitochondrial | 216841_s_at | 1.02 | 58.62 |  | 0.50 | 9.01 | ↓ | 3.93 | 2.70 | ↑ |
| ADAMTS1 | ADAM metallopeptidase with thrombospondin type 1 motif, 1 | 222486_s_at | 1.02 | 55.60 |  | 0.37 | 0.53 | ↓ | 1.00 | 58.81 |  |
| SSU72 | SSU72 RNA polymerase II CTD phosphatase homolog (S. cerevisiae) | 223052_x_at | 1.02 | 53.22 |  | 1.52 | 10.02 |  | 2.22 | 1.25 | ↑ |
| MKLN1 | muskelin 1, intracellular mediator containing kelch motifs | 244171_at | 1.02 | 54.53 |  | 0.38 | 0.53 | ↓ | 0.86 | 29.62 |  |
| GPAA1 | glycosylphosphatidylinositol anchor attachment protein 1 homolog (yeast) | 201618_x_at | 1.02 | 61.91 |  | 0.42 | 0.29 | ↓ | 0.91 | 41.22 |  |
| DAZAP2 | DAZ associated protein 2 | 212595_s_at | 1.02 | 62.00 |  | 0.27 | 0.00 | ↓ | 1.44 | 8.02 |  |
| LANCL1 | LanC lantibiotic synthetase component C-like 1 (bacterial) | 202019_s_at | 1.02 | 62.00 |  | 0.45 | 4.45 | ↓ | 1.22 | 52.56 |  |
| KIAA1632 | KIAA1632 | 1563471_at | 1.02 | 62.00 |  | 0.41 | 2.80 | ↓ | 1.46 | 50.73 |  |
| KIRREL3 | kin of IRRE like 3 (Drosophila) | 240402_at | 1.02 | 56.74 |  | 1.10 | 50.64 |  | 2.18 | 5.75 | ↑ |
| LRRFIP1 | leucine rich repeat (in FLII) interacting protein 1 | 201861_s_at | 1.02 | 60.94 |  | 2.73 | 0.98 | ↑ | 0.81 | 32.58 |  |
| EIF5A2 | eukaryotic translation initiation factor 5A2 | 235289_at | 1.02 | 58.62 |  | 1.27 | 29.01 |  | 3.24 | 0.00 | ↑ |
| LOC100507111 | hypothetical LOC100507111 | 236485_at | 1.02 | 62.00 |  | 0.40 | 1.26 | ↓ | 0.85 | 38.27 |  |
| LMBR1 | limb region 1 homolog (mouse) | 224410_s_at | 1.02 | 53.22 |  | 0.42 | 0.76 | ↓ | 1.01 | 58.81 |  |
| DYNC2H1 | dynein, cytoplasmic 2, heavy chain 1 | 1561939_at | 1.02 | 54.53 |  | 0.11 | 0.25 | ↓ | 1.20 | 43.83 |  |
| CHPF | chondroitin polymerizing factor | 202175_at | 1.01 | 62.00 |  | 0.25 | 0.00 | ↓ | 0.90 | 35.63 |  |
| SLC7A1 | solute carrier family 7 (cationic amino acid transporter, y+ system), member 1 | 212292_at | 1.01 | 59.48 |  | 0.45 | 4.45 | ↓ | 0.61 | 15.27 |  |
| ASXL2 | additional sex combs like 2 (Drosophila) | 218659_at | 1.01 | 62.00 |  | 0.42 | 0.40 | ↓ | 0.88 | 38.27 |  |
| C3orf31 | chromosome 3 open reading frame 31 | 234697_x_at | 1.01 | 58.62 |  | 0.70 | 36.20 |  | 2.57 | 2.43 | ↑ |
| ITCH | itchy E3 ubiquitin protein ligase homolog (mouse) | 235057_at | 1.01 | 58.62 |  | 8.79 | 0.00 | ↑ | 0.88 | 35.63 |  |
| PRSS35 | protease, serine, 35 | 235874_at | 1.01 | 61.96 |  | 1.20 | 50.64 |  | 2.71 | 1.99 | ↑ |
| SLC2A5 | solute carrier family 2 (facilitated glucose/fructose transporter), member 5 | 204429_s_at | 1.01 | 59.80 |  | 0.27 | 0.98 | ↓ | 4.10 | 0.72 | ↑ |
| FNTB | farnesyltransferase, CAAX box, beta | 1773_at | 1.01 | 62.00 |  | 0.45 | 0.98 | ↓ | 0.97 | 54.08 |  |
| C18orf54 | chromosome 18 open reading frame 54 | 1553652_a_at | 1.01 | 59.06 |  | 0.84 | 58.42 |  | 0.38 | 9.54 | ↓ |
| SERPINI1 | serpin peptidase inhibitor, clade I (neuroserpin), member 1 | 205352_at | 1.01 | 62.00 |  | 1.18 | 54.70 |  | 2.74 | 0.58 | ↑ |
| RRN3P1 | RNA polymerase I transcription factor homolog (S. cerevisiae) pseudogene 1 | 216908_x_at | 1.01 | 45.69 |  | 0.42 | 0.98 | ↓ | 1.22 | 48.62 |  |
| DBT | dihydrolipoamide branched chain transacylase E2 | 205369_x_at | 1.01 | 53.22 |  | 0.49 | 0.60 | ↓ | 1.12 | 58.81 |  |
| C5orf34 | chromosome 5 open reading frame 34 | 229886_at | 1.01 | 60.72 |  | 1.23 | 29.01 |  | 0.39 | 0.34 | ↓ |
| EXOC7 | exocyst complex component 7 | 212035_s_at | 1.01 | 61.35 |  | 0.43 | 0.84 | ↓ | 1.10 | 50.73 |  |
| ARG2 | arginase, type II | 203946_s_at | 1.01 | 58.62 |  | 1.54 | 36.20 |  | 0.27 | 3.36 | ↓ |
| MCAM | melanoma cell adhesion molecule | 210869_s_at | 1.01 | 51.95 |  | 0.79 | 54.70 |  | 0.32 | 5.75 | ↓ |
| DDX19A | DEAD (Asp-Glu-Ala-As) box polypeptide 19A | 202578_s_at | 1.01 | 58.62 |  | 0.45 | 0.40 | ↓ | 1.02 | 58.81 |  |
| DCTN1 | dynactin 1 | 211780_x_at | 1.01 | 62.00 |  | 0.34 | 0.29 | ↓ | 1.56 | 9.54 |  |
| IL1RAPL2 | interleukin 1 receptor accessory protein-like 2 | 221112_at | 1.01 | 34.35 |  | 0.42 | 0.45 | ↓ | 0.37 | 1.61 | ↓ |
| KDM4C | lysine (K)-specific demethylase 4C | 1556493_a_at | 1.01 | 40.42 |  | 0.44 | 3.81 | ↓ | 0.48 | 8.02 | ↓ |
| DLEU2 | deleted in lymphocytic leukemia 2 (non-protein coding) | 1556821_x_at | 1.01 | 58.62 |  | 0.82 | 55.85 |  | 0.35 | 4.84 | ↓ |
| UBE2W | ubiquitin-conjugating enzyme E2W (putative) | 218521_s_at | 1.01 | 62.00 |  | 2.31 | 0.84 | ↑ | 1.78 | 2.43 |  |
| FOXJ3 | forkhead box J3 | 217310_s_at | 1.01 | 59.80 |  | 0.35 | 0.98 | ↓ | 1.07 | 52.56 |  |
| MT1X | metallothionein 1X | 204326_x_at | 1.01 | 62.00 |  | 1.58 | 15.95 |  | 0.48 | 0.50 | ↓ |
| KIAA0494 | KIAA0494 | 201775_s_at | 1.01 | 58.62 |  | 0.37 | 0.25 | ↓ | 1.13 | 55.52 |  |
| TTC3 | tetratricopeptide repeat domain 3 | 208664_s_at | 1.01 | 57.71 |  | 0.32 | 0.00 | ↓ | 1.10 | 48.62 |  |
| CTDSPL2 | CTD (carboxy-terminal domain, RNA polymerase II, polypeptide A) small phosphatase like 2 | 1555106_a_at | 1.01 | 58.62 |  | 0.25 | 0.18 | ↓ | 0.77 | 29.62 |  |
| TMEM194A | transmembrane protein 194A | 212621_at | 1.01 | 58.62 |  | 1.08 | 52.07 |  | 0.43 | 0.00 | ↓ |
| MRC1 /// MRC1L1 | mannose receptor, C type 1 /// mannose receptor, C type 1-like 1 | 204438_at | 1.01 | 42.20 |  | 0.99 | 52.07 |  | 0.22 | 1.25 | ↓ |
| ODF2 | outer dense fiber of sperm tails 2 | 225617_at | 1.01 | 62.00 |  | 0.84 | 33.65 |  | 0.47 | 0.00 | ↓ |
| NEGR1 | neuronal growth regulator 1 | 1553194_at | 1.01 | 55.60 |  | 0.35 | 0.25 | ↓ | 1.03 | 58.81 |  |
| LOC100505584 /// MT1E | hypothetical protein LOC100505584 /// metallothionein 1E | 216336_x_at | 1.01 | 62.00 |  | 1.18 | 47.13 |  | 0.49 | 1.02 | ↓ |
| ZCCHC6 | Zinc finger, CCHC domain containing 6 | 236155_at | 1.01 | 62.00 |  | 2.14 | 0.60 | ↑ | 1.01 | 58.81 |  |
| POLR2E | polymerase (RNA) II (DNA directed) polypeptide E, 25kDa | 213887_s_at | 1.01 | 58.62 |  | 0.47 | 0.76 | ↓ | 1.09 | 52.56 |  |
| SFSWAP | splicing factor, suppressor of white-apricot homolog (Drosophila) | 202773_s_at | 1.01 | 58.62 |  | 0.38 | 0.25 | ↓ | 0.81 | 20.38 |  |
| CAPRIN1 | cell cycle associated protein 1 | 200722_s_at | 1.00 | 55.60 |  | 0.38 | 0.18 | ↓ | 1.12 | 43.83 |  |
| SOCS4 | suppressor of cytokine signaling 4 | 1552792_at | 1.00 | 55.60 |  | 0.49 | 6.11 | ↓ | 1.56 | 15.27 |  |
| ERC1 | ELKS/RAB6-interacting/CAST family member 1 | 1552663_a_at | 1.00 | 62.00 |  | 0.29 | 0.18 | ↓ | 1.07 | 56.10 |  |
| LOC389906 | hypothetical LOC389906 | 222196_at | 1.00 | 57.71 |  | 1.40 | 52.07 |  | 0.29 | 4.16 | ↓ |
| HNRNPH1 | heterogeneous nuclear ribonucleoprotein H1 (H) | 201031_s_at | 1.00 | 62.00 |  | 2.01 | 0.70 | ↑ | 0.78 | 8.02 |  |
| SPRED1 | sprouty-related, EVH1 domain containing 1 | 244439_at | 1.00 | 55.60 |  | 0.21 | 0.00 | ↓ | 1.06 | 56.65 |  |
| RBM26 | RNA binding motif protein 26 | 218422_s_at | 1.00 | 58.62 |  | 2.65 | 0.00 | ↑ | 0.74 | 4.84 |  |
| CCDC91 | Coiled-coil domain containing 91 | 1570571_at | 1.00 | 61.68 |  | 0.44 | 6.11 | ↓ | 1.03 | 54.08 |  |
| SCYL2 | SCY1-like 2 (S. cerevisiae) | 221220_s_at | 1.00 | 42.20 |  | 0.14 | 0.29 | ↓ | 1.31 | 32.58 |  |
| SLC35A4 | solute carrier family 35, member A4 | 224626_at | 1.00 | 58.62 |  | 0.37 | 0.25 | ↓ | 0.91 | 38.27 |  |
| AMOTL1 | angiomotin like 1 | 225459_at | 1.00 | 57.71 |  | 0.39 | 0.25 | ↓ | 0.98 | 50.73 |  |
| ZNF702P | zinc finger protein 702, pseudogene | 206557_at | 1.00 | 58.62 |  | 0.87 | 58.42 |  | 0.46 | 9.54 | ↓ |
| DAB2 | disabled homolog 2, mitogen-responsive phosphoprotein (Drosophila) | 201279_s_at | 1.00 | 62.00 |  | 0.46 | 0.70 | ↓ | 1.84 | 0.50 |  |
| ACVR1C | activin A receptor, type IC | 1552519_at | 1.00 | 58.62 |  | 0.33 | 0.84 | ↓ | 0.69 | 15.27 |  |
| ARL4A | ADP-ribosylation factor-like 4A | 205020_s_at | 1.00 | 53.22 |  | 1.78 | 14.26 |  | 2.08 | 5.75 | ↑ |
| LMO4 | LIM domain only 4 | 209204_at | 1.00 | 62.00 |  | 2.63 | 0.98 | ↑ | 0.60 | 11.20 |  |
| PARP3 | poly (ADP-ribose) polymerase family, member 3 | 209940_at | 1.00 | 62.00 |  | 0.44 | 0.70 | ↓ | 0.83 | 23.22 |  |
| PCDHB15 | protocadherin beta 15 | 231789_at | 1.00 | 50.55 |  | 1.06 | 47.13 |  | 0.43 | 3.36 | ↓ |
| SRPR | signal recognition particle receptor (docking protein) | 200918_s_at | 1.00 | 62.00 |  | 0.36 | 0.25 | ↓ | 0.91 | 35.63 |  |
| SLC4A2 | solute carrier family 4, anion exchanger, member 2 (erythrocyte membrane protein band 3-like 1) | 202111_at | 1.00 | 62.00 |  | 0.30 | 0.53 | ↓ | 1.12 | 54.08 |  |
| DDX42 | DEAD (Asp-Glu-Ala-Asp) box polypeptide 42 | 1559954_s_at | 1.00 | 56.74 |  | 0.15 | 0.00 | ↓ | 1.05 | 55.52 |  |
| TK2 | thymidine kinase 2, mitochondrial | 204276_at | 1.00 | 54.53 |  | 2.14 | 0.29 | ↑ | 0.91 | 35.63 |  |
| ZNF192 | zinc finger protein 192 | 206579_at | 1.00 | 45.69 |  | 0.29 | 0.84 | ↓ | 1.30 | 54.85 |  |
| CELF2 | CUGBP, Elav-like family member 2 | 227178_at | 1.00 | 47.41 |  | 0.36 | 1.26 | ↓ | 2.45 | 3.36 | ↑ |
| COASY | CoA synthase | 201913_s_at | 1.00 | 58.62 |  | 0.48 | 0.25 | ↓ | 0.82 | 13.22 |  |
| SOD2 | superoxide dismutase 2, mitochondrial | 215223_s_at | 1.00 | 59.80 |  | 0.43 | 7.87 | ↓ | 2.61 | 13.22 |  |
| FGFR2 | fibroblast growth factor receptor 2 | 203638_s_at | 1.00 | 53.22 |  | 1.67 | 14.26 |  | 0.09 | 0.00 | ↓ |
| RASD1 | RAS, dexamethasone-induced 1 | 223467_at | 1.00 | 48.99 |  | 0.42 | 22.17 |  | 0.26 | 5.75 | ↓ |
| GALNS | galactosamine (N-acetyl)-6-sulfate sulfatase | 206335_at | 1.00 | 62.00 |  | 1.66 | 15.95 |  | 0.45 | 1.25 | ↓ |
| PDE4DIP | phosphodiesterase 4D interacting protein | 212392_s_at | 1.00 | 54.53 |  | 0.29 | 0.00 | ↓ | 0.93 | 43.83 |  |
| C1GALT1C1 | C1GALT1-specific chaperone 1 | 238989_at | 1.00 | 58.62 |  | 0.42 | 1.98 | ↓ | 0.80 | 32.58 |  |
| DDAH1 | dimethylarginine dimethylaminohydrolase 1 | 1553565_s_at | 1.00 | 58.62 |  | 0.37 | 22.17 |  | 2.16 | 9.54 | ↑ |
| SSPN | sarcospan (Kras oncogene-associated gene) | 204963_at | 1.00 | 62.00 |  | 2.99 | 2.30 | ↑ | 0.50 | 2.43 | ↓ |
| SEL1L | Sel-1 suppressor of lin-12-like (C. elegans) | 230265_at | 1.00 | 58.62 |  | 4.54 | 0.00 | ↑ | 1.13 | 41.22 |  |
| LOC644538 | hypothetical protein LOC644538 | 227976_at | 1.00 | 53.22 |  | 2.00 | 2.30 | ↑ | 1.14 | 48.62 |  |
| MAP3K7 | mitogen-activated protein kinase kinase kinase 7 | 211536_x_at | 1.00 | 56.74 |  | 0.35 | 0.18 | ↓ | 1.19 | 32.58 |  |
| MFN2 | mitofusin 2 | 216205_s_at | 1.00 | 61.35 |  | 0.26 | 0.36 | ↓ | 1.59 | 17.70 |  |
| TRO | trophinin | 211700_s_at | 1.00 | 62.00 |  | 0.93 | 52.07 |  | 0.43 | 4.84 | ↓ |
| DAGLB | diacylglycerol lipase, beta | 225833_at | 0.99 | 50.55 |  | 0.38 | 0.18 | ↓ | 1.97 | 0.31 |  |
| PTBP1 | polypyrimidine tract binding protein 1 | 212016_s_at | 0.99 | 62.00 |  | 0.18 | 0.00 | ↓ | 0.72 | 17.70 |  |
| UBA7 | ubiquitin-like modifier activating enzyme 7 | 203281_s_at | 0.99 | 54.53 |  | 0.54 | 1.45 |  | 0.47 | 0.21 | ↓ |
| CD53 | CD53 molecule | 203416_at | 0.99 | 56.74 |  | 0.69 | 15.95 |  | 0.48 | 4.84 | ↓ |
| LOC100292909 | hypothetical protein LOC100292909 | 241535_at | 0.99 | 62.00 |  | 0.95 | 47.13 |  | 0.14 | 0.00 | ↓ |
| LOC401097 | hypothetical protein LOC401097 | 236738_at | 0.99 | 62.00 |  | 1.00 | 59.66 |  | 0.33 | 2.70 | ↓ |
| HOXA3 | homeobox A3 | 208604_s_at | 0.99 | 62.00 |  | 0.46 | 3.35 | ↓ | 1.20 | 50.73 |  |
| C10orf18 | chromosome 10 open reading frame 18 | 238795_at | 0.99 | 62.00 |  | 0.20 | 0.00 | ↓ | 1.31 | 32.58 |  |
| UNC5C | unc-5 homolog C (C. elegans) | 227838_at | 0.99 | 58.62 |  | 1.20 | 49.04 |  | 0.28 | 1.43 | ↓ |
| SPIRE1 | spire homolog 1 (Drosophila) | 1554807_a_at | 0.99 | 57.71 |  | 0.48 | 1.45 | ↓ | 1.14 | 46.35 |  |
| SYNPO2 | synaptopodin 2 | 227662_at | 0.99 | 55.60 |  | 2.02 | 9.01 | ↑ | 0.81 | 43.83 |  |
| MST4 | serine/threonine protein kinase MST4 | 218499_at | 0.99 | 58.62 |  | 1.14 | 41.29 |  | 0.20 | 0.00 | ↓ |
| CTSK | cathepsin K | 202450_s_at | 0.99 | 45.69 |  | 0.63 | 11.08 |  | 2.14 | 3.36 | ↑ |
| LOC730184 | hypothetical LOC730184 | 241053_at | 0.99 | 55.60 |  | 0.50 | 1.45 | ↓ | 0.87 | 35.63 |  |
| LRRC2 | leucine rich repeat containing 2 | 231781_s_at | 0.99 | 43.91 |  | 1.08 | 60.11 |  | 2.85 | 1.43 | ↑ |
| LUC7L | LUC7-like (S. cerevisiae) | 1557066_at | 0.99 | 60.45 |  | 0.38 | 3.35 | ↓ | 0.63 | 26.48 |  |
| PGGT1B | protein geranylgeranyltransferase type I, beta subunit | 235615_at | 0.99 | 62.00 |  | 2.52 | 1.74 | ↑ | 1.06 | 54.85 |  |
| TGM2 | transglutaminase 2 (C polypeptide, protein-glutamine-gamma-glutamyltransferase) | 211573_x_at | 0.99 | 51.95 |  | 0.27 | 0.00 | ↓ | 1.28 | 46.35 |  |
| THUMPD3 | THUMP domain containing 3 | 225730_s_at | 0.99 | 62.00 |  | 2.54 | 0.60 | ↑ | 1.19 | 35.63 |  |
| SRGAP1 | SLIT-ROBO Rho GTPase activating protein 1 | 1569269_s_at | 0.99 | 60.18 |  | 0.17 | 0.00 | ↓ | 1.40 | 29.62 |  |
| NONO | non-POU domain containing, octamer-binding | 210470_x_at | 0.99 | 62.00 |  | 0.37 | 0.53 | ↓ | 0.66 | 9.54 |  |
| SS18 | synovial sarcoma translocation, chromosome 18 | 216684_s_at | 0.99 | 62.00 |  | 0.42 | 1.26 | ↓ | 0.83 | 38.27 |  |
| SERPINB2 | serpin peptidase inhibitor, clade B (ovalbumin), member 2 | 204614_at | 0.99 | 60.45 |  | 0.37 | 17.74 |  | 8.99 | 0.89 | ↑ |
| UNC45A | unc-45 homolog A (C. elegans) | 207499_x_at | 0.99 | 58.62 |  | 0.31 | 0.25 | ↓ | 1.15 | 46.35 |  |
| --- | --- | 228638_at | 0.99 | 62.00 |  | 0.48 | 1.45 | ↓ | 1.49 | 20.38 |  |
| NOV | nephroblastoma overexpressed gene | 204501_at | 0.99 | 62.00 |  | 0.36 | 4.45 | ↓ | 3.26 | 17.70 |  |
| USO1 | USO1 vesicle docking protein homolog (yeast) | 201831_s_at | 0.99 | 54.53 |  | 0.45 | 0.25 | ↓ | 0.97 | 48.62 |  |
| SOX11 | SRY (sex determining region Y)-box 11 | 204915_s_at | 0.99 | 62.00 |  | 0.98 | 57.71 |  | 0.12 | 0.00 | ↓ |
| POR | P450 (cytochrome) oxidoreductase | 208928_at | 0.99 | 47.41 |  | 0.44 | 0.45 | ↓ | 0.75 | 11.20 |  |
| FASTK | Fas-activated serine/threonine kinase | 202676_x_at | 0.99 | 58.62 |  | 0.47 | 0.60 | ↓ | 1.10 | 46.35 |  |
| IFI44L | interferon-induced protein 44-like | 204439_at | 0.99 | 62.00 |  | 0.46 | 15.95 |  | 0.16 | 2.70 | ↓ |
| SLC44A1 | solute carrier family 44, member 1 | 224595_at | 0.99 | 55.60 |  | 2.66 | 0.70 | ↑ | 0.78 | 23.22 |  |
| SPTBN1 | spectrin, beta, non-erythrocytic 1 | 200671_s_at | 0.99 | 51.95 |  | 0.15 | 0.00 | ↓ | 0.87 | 48.62 |  |
| IL7 | interleukin 7 | 206693_at | 0.99 | 60.45 |  | 1.25 | 52.07 |  | 0.40 | 7.00 | ↓ |
| PILRB | paired immunoglobin-like type 2 receptor beta | 225321_s_at | 0.99 | 55.60 |  | 1.41 | 24.54 |  | 0.46 | 0.34 | ↓ |
| UTP23 | UTP23, small subunit (SSU) processome component, homolog (yeast) | 238561_s_at | 0.99 | 53.22 |  | 0.49 | 0.98 | ↓ | 1.46 | 8.02 |  |
| ITPR1 | inositol 1,4,5-triphosphate receptor, type 1 | 211323_s_at | 0.99 | 54.53 |  | 0.56 | 4.45 |  | 0.44 | 1.61 | ↓ |
| LOC442075 | hypothetical LOC442075 | 237005_at | 0.99 | 58.62 |  | 0.81 | 26.77 |  | 0.33 | 2.43 | ↓ |
| CTTN | cortactin | 201059_at | 0.99 | 62.00 |  | 3.14 | 0.60 | ↑ | 1.44 | 29.62 |  |
| TAB2 | TGF-beta activated kinase 1/MAP3K7 binding protein 2 | 210284_s_at | 0.99 | 55.60 |  | 0.47 | 0.36 | ↓ | 1.13 | 35.63 |  |
| FKBP11 | FK506 binding protein 11, 19 kDa | 219118_at | 0.99 | 58.62 |  | 0.95 | 54.70 |  | 0.49 | 0.43 | ↓ |
| FAM54A | family with sequence similarity 54, member A | 234944_s_at | 0.99 | 55.60 |  | 0.83 | 31.53 |  | 0.40 | 1.02 | ↓ |
| MCM3AP | minichromosome maintenance complex component 3 associated protein | 215581_s_at | 0.99 | 62.00 |  | 0.20 | 0.25 | ↓ | 0.77 | 43.83 |  |
| ZC3H11A | zinc finger CCCH-type containing 11A | 205787_x_at | 0.98 | 58.62 |  | 0.48 | 0.98 | ↓ | 1.15 | 52.56 |  |
| MIR21 | microRNA 21 | 224917_at | 0.98 | 62.00 |  | 1.42 | 29.01 |  | 0.41 | 0.34 | ↓ |
| CCR10 | chemokine (C-C motif) receptor 10 | 220565_at | 0.98 | 55.60 |  | 0.84 | 53.46 |  | 0.49 | 7.00 | ↓ |
| LOC100506653 | hypothetical LOC100506653 | 221919_at | 0.98 | 55.60 |  | 1.23 | 29.01 |  | 0.34 | 0.00 | ↓ |
| FNTB | farnesyltransferase, CAAX box, beta | 204764_at | 0.98 | 56.74 |  | 0.33 | 0.18 | ↓ | 1.06 | 55.52 |  |
| PICALM | phosphatidylinositol binding clathrin assembly protein | 215236_s_at | 0.98 | 58.62 |  | 0.29 | 0.40 | ↓ | 1.18 | 46.35 |  |
| MIA3 | melanoma inhibitory activity family, member 3 | 212305_s_at | 0.98 | 55.60 |  | 0.45 | 0.45 | ↓ | 1.04 | 56.65 |  |
| IGF2R | insulin-like growth factor 2 receptor | 201392_s_at | 0.98 | 56.74 |  | 2.36 | 0.45 | ↑ | 1.15 | 48.62 |  |
| PDPN | podoplanin | 226658_at | 0.98 | 57.71 |  | 0.26 | 1.98 | ↓ | 1.09 | 54.85 |  |
| C9orf85 | chromosome 9 open reading frame 85 | 238579_at | 0.98 | 50.55 |  | 0.41 | 0.98 | ↓ | 0.83 | 32.58 |  |
| KIAA1109 | KIAA1109 | 216294_s_at | 0.98 | 54.53 |  | 0.33 | 0.00 | ↓ | 1.58 | 1.99 |  |
| NFS1 | NFS1 nitrogen fixation 1 homolog (S. cerevisiae) | 1554321_a_at | 0.98 | 51.95 |  | 0.29 | 0.00 | ↓ | 0.89 | 29.62 |  |
| SLFN5 | schlafen family member 5 | 243999_at | 0.98 | 56.74 |  | 0.43 | 0.70 | ↓ | 1.08 | 52.56 |  |
| MCAM | melanoma cell adhesion molecule | 209087_x_at | 0.98 | 59.06 |  | 0.75 | 47.13 |  | 0.41 | 7.00 | ↓ |
| ALDH18A1 | aldehyde dehydrogenase 18 family, member A1 | 217791_s_at | 0.98 | 56.74 |  | 0.43 | 0.18 | ↓ | 0.90 | 32.58 |  |
| C6orf62 | chromosome 6 open reading frame 62 | 208809_s_at | 0.98 | 58.62 |  | 2.48 | 0.40 | ↑ | 0.94 | 46.35 |  |
| PI4KB | phosphatidylinositol 4-kinase, catalytic, beta | 210417_s_at | 0.98 | 56.74 |  | 0.31 | 0.00 | ↓ | 1.03 | 58.81 |  |
| BTN3A3 | butyrophilin, subfamily 3, member A3 | 204821_at | 0.98 | 58.62 |  | 0.50 | 3.81 | ↓ | 0.73 | 17.70 |  |
| KIAA1826 | KIAA1826 | 223799_at | 0.98 | 55.60 |  | 0.11 | 0.00 | ↓ | 1.54 | 9.54 |  |
| PSME4 | proteasome (prosome, macropain) activator subunit 4 | 212220_at | 0.98 | 62.00 |  | 0.49 | 4.45 | ↓ | 1.03 | 58.51 |  |
| NONO | non-POU domain containing, octamer-binding | 208698_s_at | 0.98 | 57.71 |  | 0.31 | 0.18 | ↓ | 0.79 | 20.38 |  |
| MN1 | meningioma (disrupted in balanced translocation) 1 | 205330_at | 0.98 | 58.62 |  | 1.81 | 10.02 |  | 0.50 | 7.00 | ↓ |
| LAPTM4B | lysosomal protein transmembrane 4 beta | 1554679_a_at | 0.98 | 56.74 |  | 0.26 | 0.18 | ↓ | 0.87 | 38.27 |  |
| MKX | mohawk homeobox | 241902_at | 0.98 | 62.00 |  | 0.40 | 22.17 |  | 2.66 | 9.54 | ↑ |
| KYNU | kynureninase (L-kynurenine hydrolase) | 204385_at | 0.98 | 36.49 |  | 0.28 | 0.40 | ↓ | 0.43 | 2.43 | ↓ |
| NADK | NAD kinase | 208917_x_at | 0.98 | 62.00 |  | 0.33 | 1.07 | ↓ | 0.79 | 32.58 |  |
| PATL1 | protein associated with topoisomerase II homolog 1 (yeast) | 235234_at | 0.98 | 60.94 |  | 0.33 | 0.70 | ↓ | 0.82 | 41.22 |  |
| SLMAP | sarcolemma associated protein | 224149_x_at | 0.98 | 58.62 |  | 0.26 | 0.25 | ↓ | 0.97 | 58.81 |  |
| SLAIN2 | SLAIN motif family, member 2 | 233230_s_at | 0.98 | 54.53 |  | 0.37 | 0.18 | ↓ | 1.34 | 11.20 |  |
| ZDHHC5 | zinc finger, DHHC-type containing 5 | 224868_at | 0.98 | 51.95 |  | 0.33 | 0.00 | ↓ | 0.82 | 17.70 |  |
| WDR27 | WD repeat domain 27 | 228326_at | 0.98 | 54.53 |  | 0.85 | 33.65 |  | 0.40 | 0.21 | ↓ |
| G3BP2 | GTPase activating protein (SH3 domain) binding protein 2 | 208840_s_at | 0.98 | 56.74 |  | 0.41 | 0.84 | ↓ | 1.10 | 56.10 |  |
| ARF3 | ADP-ribosylation factor 3 | 211622_s_at | 0.98 | 62.00 |  | 0.34 | 0.53 | ↓ | 0.64 | 9.54 |  |
| ALDH1B1 | aldehyde dehydrogenase 1 family, member B1 | 209645_s_at | 0.97 | 62.00 |  | 0.34 | 3.81 | ↓ | 0.80 | 54.08 |  |
| TANK | TRAF family member-associated NFKB activator | 210458_s_at | 0.97 | 47.41 |  | 0.46 | 0.70 | ↓ | 1.04 | 58.81 |  |
| NT5E | 5'-nucleotidase, ecto (CD73) | 1553995_a_at | 0.97 | 55.60 |  | 0.45 | 0.70 | ↓ | 0.93 | 46.35 |  |
| BCAT1 | branched chain amino-acid transaminase 1, cytosolic | 214452_at | 0.97 | 62.00 |  | 0.48 | 5.22 | ↓ | 1.46 | 29.62 |  |
| SNRPN /// SNURF | small nuclear ribonucleoprotein polypeptide N /// SNRPN upstream reading frame | 206042_x_at | 0.97 | 55.60 |  | 0.50 | 0.53 | ↓ | 0.99 | 54.08 |  |
| C17orf63 | chromosome 17 open reading frame 63 | 222641_s_at | 0.97 | 58.62 |  | 0.28 | 0.18 | ↓ | 0.85 | 41.22 |  |
| SDC1 | syndecan 1 | 201287_s_at | 0.97 | 60.72 |  | 0.34 | 2.80 | ↓ | 0.35 | 4.16 | ↓ |
| CABLES1 | Cdk5 and Abl enzyme substrate 1 | 225532_at | 0.97 | 62.00 |  | 0.75 | 31.53 |  | 2.00 | 4.16 | ↑ |
| RFWD3 | ring finger and WD repeat domain 3 | 218564_at | 0.97 | 53.22 |  | 0.96 | 52.07 |  | 0.48 | 0.00 | ↓ |
| NFIA | Nuclear factor I/A | 229994_at | 0.97 | 55.60 |  | 0.63 | 6.80 |  | 2.03 | 1.99 | ↑ |
| CDC42 | cell division cycle 42 (GTP binding protein, 25kDa) | 208727_s_at | 0.97 | 62.00 |  | 0.30 | 4.45 | ↓ | 0.94 | 58.81 |  |
| ADAT2 | adenosine deaminase, tRNA-specific 2, TAD2 homolog (S. cerevisiae) | 227213_at | 0.97 | 55.60 |  | 0.46 | 1.98 | ↓ | 0.91 | 38.27 |  |
| PRKAR1A | protein kinase, cAMP-dependent, regulatory, type I, alpha (tissue specific extinguisher 1) | 200604_s_at | 0.97 | 53.22 |  | 0.29 | 0.25 | ↓ | 0.94 | 54.08 |  |
| TOX4 | TOX high mobility group box family member 4 | 217448_s_at | 0.97 | 62.00 |  | 0.30 | 0.29 | ↓ | 1.01 | 57.64 |  |
| UCK1 | uridine-cytidine kinase 1 | 223142_s_at | 0.97 | 42.20 |  | 0.43 | 0.98 | ↓ | 0.83 | 29.62 |  |
| DONSON | downstream neighbor of SON | 221677_s_at | 0.97 | 54.53 |  | 0.90 | 41.29 |  | 0.45 | 0.00 | ↓ |
| KBTBD6 | kelch repeat and BTB (POZ) domain containing 6 | 1553111_a_at | 0.97 | 55.60 |  | 2.15 | 4.45 | ↑ | 0.65 | 11.20 |  |
| GALNT1 | UDP-N-acetyl-alpha-D-galactosamine:polypeptide N-acetylgalactosaminyltransferase 1 (GalNAc-T1) | 201723_s_at | 0.97 | 56.74 |  | 1.07 | 49.04 |  | 0.34 | 0.34 | ↓ |
| SYTL4 | Synaptotagmin-like 4 | 229991_s_at | 0.97 | 53.22 |  | 0.70 | 22.17 |  | 0.44 | 1.02 | ↓ |
| LOC400236 | Hypothetical LOC400236 | 227885_at | 0.97 | 50.55 |  | 0.38 | 0.40 | ↓ | 0.67 | 8.02 |  |
| ARID1B | AT rich interactive domain 1B (SWI1-like) | 225181_at | 0.97 | 58.62 |  | 2.08 | 1.45 | ↑ | 0.56 | 2.43 |  |
| DDX31 | DEAD (Asp-Glu-Ala-Asp) box polypeptide 31 | 235436_at | 0.97 | 55.60 |  | 2.14 | 2.80 | ↑ | 0.98 | 58.81 |  |
| TMED2 | transmembrane emp24 domain trafficking protein 2 | 204426_at | 0.97 | 58.62 |  | 0.39 | 0.60 | ↓ | 0.81 | 32.58 |  |
| EXOC4 | exocyst complex component 4 | 240528_s_at | 0.97 | 50.55 |  | 0.18 | 0.18 | ↓ | 1.26 | 46.35 |  |
| TRIM25 | tripartite motif-containing 25 | 206911_at | 0.97 | 48.99 |  | 0.39 | 0.53 | ↓ | 0.84 | 32.58 |  |
| ATG16L2 | ATG16 autophagy related 16-like 2 (S. cerevisiae) | 229389_at | 0.97 | 54.53 |  | 1.13 | 52.07 |  | 0.47 | 2.43 | ↓ |
| PTAR1 | protein prenyltransferase alpha subunit repeat containing 1 | 235484_at | 0.97 | 55.60 |  | 0.46 | 0.36 | ↓ | 1.64 | 15.27 |  |
| PHF16 | PHD finger protein 16 | 204866_at | 0.96 | 55.60 |  | 1.43 | 15.95 |  | 0.46 | 1.25 | ↓ |
| FAM55C | family with sequence similarity 55, member C | 243011_at | 0.96 | 51.95 |  | 0.38 | 0.25 | ↓ | 0.89 | 38.27 |  |
| KIAA1632 | KIAA1632 | 228453_at | 0.96 | 58.62 |  | 1.18 | 38.78 |  | 2.11 | 2.70 | ↑ |
| FIBIN | fin bud initiation factor homolog (zebrafish) | 226769_at | 0.96 | 58.62 |  | 1.49 | 17.74 |  | 0.50 | 7.00 | ↓ |
| NFE2L3 | nuclear factor (erythroid-derived 2)-like 3 | 236471_at | 0.96 | 58.62 |  | 0.75 | 29.01 |  | 0.49 | 4.84 | ↓ |
| TRPC4 | transient receptor potential cation channel, subfamily C, member 4 | 220817_at | 0.96 | 60.72 |  | 1.32 | 29.01 |  | 4.10 | 0.89 | ↑ |
| APOBEC3G | apolipoprotein B mRNA editing enzyme, catalytic polypeptide-like 3G | 204205_at | 0.96 | 58.62 |  | 1.33 | 41.29 |  | 0.39 | 1.43 | ↓ |
| SENP7 | SUMO1/sentrin specific peptidase 7 | 220735_s_at | 0.96 | 50.55 |  | 0.41 | 1.26 | ↓ | 0.66 | 4.16 |  |
| GMNN | geminin, DNA replication inhibitor | 218350_s_at | 0.96 | 54.53 |  | 0.97 | 59.12 |  | 0.33 | 0.00 | ↓ |
| ADAMTS5 | ADAM metallopeptidase with thrombospondin type 1 motif, 5 | 219935_at | 0.96 | 59.80 |  | 0.89 | 60.62 |  | 0.27 | 1.43 | ↓ |
| C1orf96 | chromosome 1 open reading frame 96 | 1553697_at | 0.96 | 55.60 |  | 0.36 | 2.80 | ↓ | 0.35 | 0.34 | ↓ |
| PEX13 | peroxisomal biogenesis factor 13 | 1556009_at | 0.96 | 56.74 |  | 2.15 | 1.45 | ↑ | 1.03 | 58.51 |  |
| ATP13A2 | ATPase type 13A2 | 218608_at | 0.96 | 61.96 |  | 0.59 | 14.26 |  | 0.47 | 4.16 | ↓ |
| MON1B | MON1 homolog B (yeast) | 203644_s_at | 0.96 | 48.99 |  | 1.05 | 50.64 |  | 0.43 | 4.16 | ↓ |
| API5 | apoptosis inhibitor 5 | 201686_x_at | 0.96 | 53.22 |  | 0.45 | 0.76 | ↓ | 1.12 | 46.35 |  |
| RBM22 | RNA binding motif protein 22 | 222527_s_at | 0.96 | 48.99 |  | 0.40 | 0.45 | ↓ | 1.03 | 58.81 |  |
| LRRC58 | leucine rich repeat containing 58 | 238506_at | 0.96 | 55.60 |  | 0.25 | 0.00 | ↓ | 1.19 | 50.73 |  |
| SCRN3 | secernin 3 | 219234_x_at | 0.96 | 54.53 |  | 0.50 | 1.07 | ↓ | 1.20 | 35.63 |  |
| DLST | dihydrolipoamide S-succinyltransferase (E2 component of 2-oxo-glutarate complex) | 215210_s_at | 0.96 | 58.62 |  | 2.11 | 2.30 | ↑ | 0.85 | 41.22 |  |
| GDE1 | glycerophosphodiester phosphodiesterase 1 | 202593_s_at | 0.96 | 51.95 |  | 0.49 | 0.84 | ↓ | 1.33 | 13.22 |  |
| RPS27L | ribosomal protein S27-like | 222487_s_at | 0.96 | 55.60 |  | 3.43 | 0.14 | ↑ | 1.49 | 9.54 |  |
| EML4 | echinoderm microtubule associated protein like 4 | 223069_s_at | 0.96 | 51.95 |  | 0.36 | 0.18 | ↓ | 0.61 | 1.02 |  |
| --- | --- | 213889_at | 0.96 | 54.53 |  | 1.05 | 52.07 |  | 0.48 | 0.00 | ↓ |
| SOS2 | son of sevenless homolog 2 (Drosophila) | 211665_s_at | 0.96 | 55.60 |  | 0.39 | 0.60 | ↓ | 1.34 | 23.22 |  |
| TNKS2 | tankyrase, TRF1-interacting ankyrin-related ADP-ribose polymerase 2 | 222563_s_at | 0.96 | 62.00 |  | 0.39 | 0.76 | ↓ | 1.10 | 58.28 |  |
| SNX5 | sorting nexin 5 | 229981_at | 0.96 | 54.53 |  | 0.80 | 38.78 |  | 0.46 | 2.43 | ↓ |
| TYMS | thymidylate synthetase | 1554696_s_at | 0.96 | 53.22 |  | 0.53 | 2.30 |  | 0.17 | 0.00 | ↓ |
| IGDCC4 | immunoglobulin superfamily, DCC subclass, member 4 | 227870_at | 0.96 | 54.53 |  | 1.34 | 29.01 |  | 0.39 | 0.43 | ↓ |
| TMEM30A | transmembrane protein 30A | 222391_at | 0.96 | 54.53 |  | 2.02 | 1.45 | ↑ | 1.58 | 2.43 |  |
| ARF1 | ADP-ribosylation factor 1 | 208750_s_at | 0.96 | 53.22 |  | 0.36 | 0.00 | ↓ | 1.07 | 56.10 |  |
| TFPI2 | tissue factor pathway inhibitor 2 | 209278_s_at | 0.96 | 56.74 |  | 0.73 | 45.34 |  | 0.10 | 0.00 | ↓ |
| MXRA7 | matrix-remodelling associated 7 | 235836_at | 0.96 | 50.55 |  | 1.15 | 47.13 |  | 2.11 | 1.25 | ↑ |
| CLDN23 | claudin 23 | 228706_s_at | 0.96 | 56.74 |  | 1.07 | 59.66 |  | 0.37 | 3.36 | ↓ |
| TMOD3 | tropomodulin 3 (ubiquitous) | 220800_s_at | 0.96 | 53.22 |  | 0.24 | 0.00 | ↓ | 1.13 | 56.10 |  |
| TNFAIP8L1 | tumor necrosis factor, alpha-induced protein 8-like 1 | 227420_at | 0.96 | 55.60 |  | 0.77 | 22.17 |  | 0.37 | 0.58 | ↓ |
| C6orf62 | Chromosome 6 open reading frame 62 | 213872_at | 0.95 | 50.55 |  | 0.45 | 1.26 | ↓ | 0.98 | 54.08 |  |
| SMARCA2 | SWI/SNF related, matrix associated, actin dependent regulator of chromatin, subfamily a, member 2 | 212257_s_at | 0.95 | 55.60 |  | 0.30 | 0.18 | ↓ | 0.98 | 58.81 |  |
| TSC2 | tuberous sclerosis 2 | 215735_s_at | 0.95 | 62.00 |  | 0.23 | 0.18 | ↓ | 0.67 | 17.70 |  |
| SPICE1 | spindle and centriole associated protein 1 | 234995_at | 0.95 | 51.95 |  | 1.22 | 31.53 |  | 0.46 | 0.34 | ↓ |
| FANCI | Fanconi anemia, complementation group I | 223785_at | 0.95 | 54.53 |  | 0.47 | 2.80 | ↓ | 0.84 | 38.27 |  |
| WWP2 | WW domain containing E3 ubiquitin protein ligase 2 | 1552737_s_at | 0.95 | 45.69 |  | 0.31 | 0.60 | ↓ | 1.11 | 52.56 |  |
| GALNT1 | UDP-N-acetyl-alpha-D-galactosamine:polypeptide N-acetylgalactosaminyltransferase 1 (GalNAc-T1) | 201722_s_at | 0.95 | 51.95 |  | 1.13 | 41.29 |  | 0.39 | 0.43 | ↓ |
| NCSTN | nicastrin | 208759_at | 0.95 | 51.95 |  | 0.42 | 0.18 | ↓ | 0.94 | 43.83 |  |
| LARP4 | La ribonucleoprotein domain family, member 4 | 238959_at | 0.95 | 50.55 |  | 0.20 | 0.25 | ↓ | 1.24 | 48.62 |  |
| CCPG1 | cell cycle progression 1 | 221156_x_at | 0.95 | 56.74 |  | 2.03 | 3.35 | ↑ | 1.55 | 20.38 |  |
| ITGB3 | integrin, beta 3 (platelet glycoprotein IIIa, antigen CD61) | 204628_s_at | 0.95 | 58.62 |  | 1.02 | 55.85 |  | 0.49 | 4.84 | ↓ |
| C18orf54 | chromosome 18 open reading frame 54 | 229442_at | 0.95 | 54.53 |  | 0.90 | 43.50 |  | 0.36 | 0.00 | ↓ |
| PLAU | plasminogen activator, urokinase | 211668_s_at | 0.95 | 45.69 |  | 0.17 | 0.00 | ↓ | 1.00 | 54.08 |  |
| UBE4B | ubiquitination factor E4B (UFD2 homolog, yeast) | 215533_s_at | 0.95 | 54.53 |  | 0.33 | 0.00 | ↓ | 1.16 | 41.22 |  |
| LOC728264 | hypothetical LOC728264 | 227183_at | 0.95 | 61.16 |  | 0.73 | 43.50 |  | 0.35 | 4.16 | ↓ |
| C5orf22 | chromosome 5 open reading frame 22 | 1552660_a_at | 0.95 | 58.62 |  | 0.25 | 0.25 | ↓ | 1.08 | 54.85 |  |
| GUCY1B3 | guanylate cyclase 1, soluble, beta 3 | 211555_s_at | 0.95 | 45.69 |  | 0.36 | 6.80 | ↓ | 0.50 | 17.70 |  |
| MFN1 | mitofusin 1 | 211801_x_at | 0.95 | 50.55 |  | 0.44 | 0.45 | ↓ | 0.92 | 41.22 |  |
| ZXDB | zinc finger, X-linked, duplicated B | 228005_at | 0.95 | 51.95 |  | 2.10 | 3.35 | ↑ | 0.74 | 11.20 |  |
| RECK | reversion-inducing-cysteine-rich protein with kazal motifs | 1558115_at | 0.95 | 53.22 |  | 1.12 | 49.04 |  | 2.45 | 1.02 | ↑ |
| KIF2C | kinesin family member 2C | 211519_s_at | 0.95 | 51.95 |  | 0.41 | 0.70 | ↓ | 0.29 | 0.00 | ↓ |
| ROR1 | receptor tyrosine kinase-like orphan receptor 1 | 205805_s_at | 0.95 | 45.69 |  | 0.87 | 43.50 |  | 2.15 | 3.36 | ↑ |
| UHRF1BP1L | UHRF1 binding protein 1-like | 1554292_a_at | 0.95 | 50.55 |  | 0.44 | 1.74 | ↓ | 0.99 | 58.81 |  |
| STX16 | syntaxin 16 | 1558249_s_at | 0.95 | 48.99 |  | 0.24 | 0.00 | ↓ | 0.89 | 29.62 |  |
| TRIM23 | tripartite motif-containing 23 | 210995_s_at | 0.95 | 50.55 |  | 0.43 | 0.29 | ↓ | 1.12 | 56.10 |  |
| WBSCR16 | Williams-Beuren syndrome chromosome region 16 | 1554410_a_at | 0.95 | 50.55 |  | 0.38 | 0.36 | ↓ | 0.79 | 23.22 |  |
| PRUNE | prune homolog (Drosophila) | 209599_s_at | 0.95 | 43.91 |  | 0.25 | 0.00 | ↓ | 1.23 | 32.58 |  |
| PLEKHM2 | pleckstrin homology domain containing, family M (with RUN domain) member 2 | 212146_at | 0.95 | 53.22 |  | 0.45 | 0.60 | ↓ | 1.14 | 43.83 |  |
| PDE7A | Phosphodiesterase 7A | 230500_at | 0.95 | 28.36 |  | 0.32 | 1.74 | ↓ | 0.41 | 1.02 | ↓ |
| TBRG4 | transforming growth factor beta regulator 4 | 220789_s_at | 0.95 | 50.55 |  | 0.45 | 0.98 | ↓ | 0.81 | 13.22 |  |
| TGFB2 | transforming growth factor, beta 2 | 209909_s_at | 0.95 | 58.62 |  | 1.09 | 43.50 |  | 0.35 | 1.61 | ↓ |
| LOC283663 | hypothetical LOC283663 | 230245_s_at | 0.94 | 34.35 |  | 0.63 | 7.87 |  | 0.48 | 5.75 | ↓ |
| TWIST1 | twist homolog 1 (Drosophila) | 213943_at | 0.94 | 58.62 |  | 1.40 | 22.17 |  | 0.41 | 0.43 | ↓ |
| PATL1 | protein associated with topoisomerase II homolog 1 (yeast) | 235235_s_at | 0.94 | 43.91 |  | 0.24 | 0.00 | ↓ | 0.62 | 2.70 |  |
| EZR | ezrin | 208623_s_at | 0.94 | 62.00 |  | 0.87 | 53.46 |  | 0.48 | 4.16 | ↓ |
| WHSC1 | Wolf-Hirschhorn syndrome candidate 1 | 209052_s_at | 0.94 | 56.74 |  | 0.23 | 0.00 | ↓ | 0.40 | 0.34 | ↓ |
| PLAT | plasminogen activator, tissue | 201860_s_at | 0.94 | 40.42 |  | 0.39 | 3.35 | ↓ | 1.69 | 9.54 |  |
| TRIM33 | tripartite motif-containing 33 | 210266_s_at | 0.94 | 56.74 |  | 2.10 | 1.74 | ↑ | 0.67 | 8.02 |  |
| RIPK1 | receptor (TNFRSF)-interacting serine-threonine kinase 1 | 209941_at | 0.94 | 48.99 |  | 0.38 | 0.00 | ↓ | 0.94 | 41.22 |  |
| FLJ10038 | hypothetical protein FLJ10038 | 236164_at | 0.94 | 54.53 |  | 2.26 | 3.35 | ↑ | 0.90 | 43.83 |  |
| MED12 | mediator complex subunit 12 | 203506_s_at | 0.94 | 62.00 |  | 0.41 | 3.35 | ↓ | 0.62 | 17.70 |  |
| GATA2 | GATA binding protein 2 | 209710_at | 0.94 | 42.20 |  | 1.59 | 10.02 |  | 0.47 | 2.43 | ↓ |
| CSF2RB | colony stimulating factor 2 receptor, beta, low-affinity (granulocyte-macrophage) | 205159_at | 0.94 | 61.16 |  | 0.83 | 59.66 |  | 8.01 | 1.25 | ↑ |
| LOC100509749 | Golgin subfamily A member 8B-like | 213650_at | 0.94 | 48.99 |  | 2.27 | 2.80 | ↑ | 1.34 | 23.22 |  |
| C18orf54 | chromosome 18 open reading frame 54 | 244324_at | 0.94 | 55.60 |  | 0.96 | 60.64 |  | 0.47 | 4.16 | ↓ |
| TRPS1 | trichorhinophalangeal syndrome I | 234351_x_at | 0.94 | 58.62 |  | 0.49 | 3.35 | ↓ | 0.97 | 54.08 |  |
| YEATS4 | YEATS domain containing 4 | 218911_at | 0.94 | 51.95 |  | 1.76 | 6.11 |  | 0.45 | 1.61 | ↓ |
| MSH6 | mutS homolog 6 (E. coli) | 211450_s_at | 0.94 | 50.55 |  | 0.36 | 0.18 | ↓ | 0.74 | 9.54 |  |
| API5 | apoptosis inhibitor 5 | 214959_s_at | 0.94 | 50.55 |  | 0.41 | 0.70 | ↓ | 1.00 | 54.08 |  |
| HGF | hepatocyte growth factor (hepapoietin A; scatter factor) | 210997_at | 0.94 | 62.00 |  | 1.65 | 31.53 |  | 0.07 | 0.00 | ↓ |
| MIPOL1 | mirror-image polydactyly 1 | 1552573_s_at | 0.94 | 58.62 |  | 0.91 | 60.31 |  | 0.43 | 4.16 | ↓ |
| TFCP2 | transcription factor CP2 | 207627_s_at | 0.94 | 50.55 |  | 0.44 | 0.25 | ↓ | 0.85 | 26.48 |  |
| SCARB2 | scavenger receptor class B, member 2 | 201646_at | 0.94 | 47.41 |  | 0.31 | 0.00 | ↓ | 0.95 | 46.35 |  |
| KLF3 | Kruppel-like factor 3 (basic) | 225140_at | 0.94 | 55.60 |  | 0.39 | 0.84 | ↓ | 0.92 | 50.73 |  |
| NUTF2 | nuclear transport factor 2 | 202397_at | 0.94 | 58.62 |  | 2.66 | 1.26 | ↑ | 1.07 | 54.08 |  |
| PTGER4 | prostaglandin E receptor 4 (subtype EP4) | 204897_at | 0.94 | 50.55 |  | 2.14 | 2.80 | ↑ | 2.23 | 58.81 |  |
| PPIA | peptidylprolyl isomerase A (cyclophilin A) | 217602_at | 0.94 | 36.49 |  | 0.42 | 3.35 | ↓ | 0.81 | 26.48 |  |
| CKAP5 | cytoskeleton associated protein 5 | 1555278_a_at | 0.94 | 48.99 |  | 0.34 | 0.00 | ↓ | 0.82 | 11.20 |  |
| LOC100128590 | hypothetical LOC100128590 | 1558920_at | 0.94 | 48.99 |  | 0.21 | 2.30 | ↓ | 0.90 | 48.62 |  |
| WASL | Wiskott-Aldrich syndrome-like | 205809_s_at | 0.94 | 42.20 |  | 2.44 | 0.40 | ↑ | 1.07 | 54.85 |  |
| SEC61A1 | Sec61 alpha 1 subunit (S. cerevisiae) | 222385_x_at | 0.94 | 53.22 |  | 0.14 | 0.00 | ↓ | 1.11 | 50.73 |  |
| FAM126A | family with sequence similarity 126, member A | 244115_at | 0.94 | 53.22 |  | 0.19 | 0.00 | ↓ | 1.40 | 26.48 |  |
| ZNF451 | zinc finger protein 451 | 215012_at | 0.94 | 58.62 |  | 0.80 | 38.78 |  | 0.43 | 1.61 | ↓ |
| LAMB1 | laminin, beta 1 | 211651_s_at | 0.94 | 50.55 |  | 1.06 | 53.46 |  | 0.37 | 0.00 | ↓ |
| MTMR1 | myotubularin related protein 1 | 214975_s_at | 0.94 | 58.62 |  | 0.21 | 0.98 | ↓ | 0.83 | 58.81 |  |
| PBLD | phenazine biosynthesis-like protein domain containing | 1555175_a_at | 0.94 | 53.22 |  | 0.47 | 1.74 | ↓ | 0.89 | 38.27 |  |
| LOC100506866 | hypothetical LOC100506866 | 242679_at | 0.94 | 54.53 |  | 0.29 | 1.26 | ↓ | 1.04 | 56.10 |  |
| SENP1 | SUMO1/sentrin specific peptidase 1 | 1552812_a_at | 0.94 | 51.95 |  | 0.34 | 0.18 | ↓ | 0.78 | 23.22 |  |
| UBE2QL1 | ubiquitin-conjugating enzyme E2Q family-like 1 | 226612_at | 0.94 | 62.00 |  | 1.46 | 36.20 |  | 0.36 | 5.75 | ↓ |
| SLC16A7 | solute carrier family 16, member 7 (monocarboxylic acid transporter 2) | 210807_s_at | 0.94 | 62.00 |  | 0.38 | 5.22 | ↓ | 1.72 | 11.20 |  |
| CELF1 | CUGBP, Elav-like family member 1 | 1555467_a_at | 0.94 | 51.95 |  | 0.23 | 0.36 | ↓ | 0.89 | 41.22 |  |
| DCAF8 | DDB1 and CUL4 associated factor 8 | 233637_at | 0.94 | 62.00 |  | 0.42 | 6.80 | ↓ | 0.97 | 57.64 |  |
| SYNJ1 | synaptojanin 1 | 207594_s_at | 0.94 | 48.99 |  | 0.26 | 0.00 | ↓ | 0.92 | 43.83 |  |
| CLOCK | clock homolog (mouse) | 204980_at | 0.94 | 45.69 |  | 0.37 | 0.18 | ↓ | 1.18 | 46.35 |  |
| HIPK3 | homeodomain interacting protein kinase 3 | 210148_at | 0.94 | 57.71 |  | 0.43 | 3.35 | ↓ | 1.76 | 13.22 |  |
| MARCKSL1 | MARCKS-like 1 | 200644_at | 0.94 | 58.62 |  | 1.57 | 31.53 |  | 0.13 | 0.00 | ↓ |
| FBXW7 | F-box and WD repeat domain containing 7 | 229419_at | 0.94 | 48.99 |  | 2.06 | 0.84 | ↑ | 1.28 | 23.22 |  |
| MAPKAP1 | mitogen-activated protein kinase associated protein 1 | 229846_s_at | 0.94 | 51.95 |  | 0.32 | 0.18 | ↓ | 1.03 | 57.64 |  |
| LPAR1 | lysophosphatidic acid receptor 1 | 204038_s_at | 0.93 | 54.53 |  | 0.46 | 2.30 | ↓ | 1.15 | 55.52 |  |
| STRAP | Serine/threonine kinase receptor associated protein | 1558002_at | 0.93 | 45.69 |  | 2.51 | 4.45 | ↑ | 0.74 | 15.27 |  |
| HNRNPD | Heterogeneous nuclear ribonucleoprotein D (AU-rich element RNA binding protein 1, 37kDa) | 213359_at | 0.93 | 53.22 |  | 1.17 | 41.29 |  | 0.47 | 1.99 | ↓ |
| SERTAD4 | SERTA domain containing 4 | 230660_at | 0.93 | 58.62 |  | 2.07 | 29.01 |  | 0.32 | 0.50 | ↓ |
| MAP3K7 | mitogen-activated protein kinase kinase kinase 7 | 211537_x_at | 0.93 | 47.41 |  | 0.45 | 0.18 | ↓ | 1.25 | 17.70 |  |
| WNK1 | WNK lysine deficient protein kinase 1 | 211993_at | 0.93 | 55.60 |  | 2.95 | 0.70 | ↑ | 1.37 | 29.62 |  |
| SSH1 | slingshot homolog 1 (Drosophila) | 1555624_a_at | 0.93 | 51.95 |  | 0.19 | 0.00 | ↓ | 0.92 | 48.62 |  |
| UBR3 | ubiquitin protein ligase E3 component n-recognin 3 (putative) | 234982_at | 0.93 | 47.41 |  | 1.17 | 38.78 |  | 2.12 | 1.25 | ↑ |
| GRAMD3 | GRAM domain containing 3 | 218706_s_at | 0.93 | 40.42 |  | 0.85 | 33.65 |  | 2.95 | 0.00 | ↑ |
| NUP160 | nucleoporin 160kDa | 214962_s_at | 0.93 | 54.53 |  | 0.49 | 3.35 | ↓ | 0.97 | 54.08 |  |
| TDRD9 | tudor domain containing 9 | 228285_at | 0.93 | 55.60 |  | 1.17 | 41.29 |  | 0.31 | 2.43 | ↓ |
| DENR | density-regulated protein | 234915_s_at | 0.93 | 54.53 |  | 0.46 | 1.45 | ↓ | 1.02 | 58.51 |  |
| GLUL | glutamate-ammonia ligase | 200648_s_at | 0.93 | 48.99 |  | 0.41 | 0.70 | ↓ | 1.22 | 52.56 |  |
| ANO10 | anoctamin 10 | 218910_at | 0.93 | 47.41 |  | 0.43 | 0.18 | ↓ | 1.09 | 52.56 |  |
| KLF5 | Kruppel-like factor 5 (intestinal) | 209212_s_at | 0.93 | 43.91 |  | 0.85 | 53.46 |  | 2.50 | 4.84 | ↑ |
| LAMP2 | lysosomal-associated membrane protein 2 | 226671_at | 0.93 | 50.55 |  | 1.29 | 29.01 |  | 2.27 | 0.72 | ↑ |
| GPR125 | G protein-coupled receptor 125 | 1555122_at | 0.93 | 43.91 |  | 0.37 | 0.70 | ↓ | 0.99 | 54.08 |  |
| XPNPEP1 | X-prolyl aminopeptidase (aminopeptidase P) 1, soluble | 208453_s_at | 0.93 | 47.41 |  | 0.45 | 0.25 | ↓ | 0.90 | 32.58 |  |
| FASTK | Fas-activated serine/threonine kinase | 210975_x_at | 0.93 | 53.22 |  | 0.43 | 0.45 | ↓ | 0.95 | 50.73 |  |
| CAT | catalase | 211922_s_at | 0.93 | 51.95 |  | 0.43 | 1.26 | ↓ | 0.82 | 35.63 |  |
| VIM | vimentin | 1555938_x_at | 0.93 | 50.55 |  | 1.26 | 41.29 |  | 0.47 | 1.61 | ↓ |
| RAD51L3 | RAD51-like 3 (S. cerevisiae) | 209965_s_at | 0.93 | 47.41 |  | 0.60 | 4.45 |  | 0.45 | 0.50 | ↓ |
| HECTD1 | HECT domain containing 1 | 1557100_s_at | 0.93 | 47.41 |  | 0.48 | 4.45 | ↓ | 1.22 | 50.73 |  |
| FNIP1 | folliculin interacting protein 1 | 228250_at | 0.93 | 50.55 |  | 0.40 | 0.70 | ↓ | 1.37 | 26.48 |  |
| LPHN2 | latrophilin 2 | 206953_s_at | 0.93 | 51.95 |  | 2.11 | 3.81 | ↑ | 0.93 | 41.22 |  |
| MPV17L2 | MPV17 mitochondrial membrane protein-like 2 | 223473_at | 0.93 | 51.95 |  | 0.48 | 1.98 | ↓ | 1.09 | 54.08 |  |
| CPSF6 | cleavage and polyadenylation specific factor 6, 68kDa | 226934_at | 0.93 | 48.99 |  | 2.40 | 5.22 | ↑ | 0.63 | 5.75 |  |
| CTAGE15P /// CTAGE4 /// CTAGE8 /// CTAGE9 | CTAGE family, member 15, pseudogene /// CTAGE family, member 4 /// CTAGE family, member 8 /// CTAGE family, member 9 | 214355_x_at | 0.93 | 54.53 |  | 0.46 | 1.07 | ↓ | 1.30 | 29.62 |  |
| DLG3 | discs, large homolog 3 (Drosophila) | 207732_s_at | 0.93 | 48.99 |  | 0.36 | 2.80 | ↓ | 0.58 | 4.16 |  |
| INPP5A | inositol polyphosphate-5-phosphatase, 40kDa | 1554757_a_at | 0.93 | 51.95 |  | 0.16 | 0.00 | ↓ | 1.34 | 26.48 |  |
| FMO2 | flavin containing monooxygenase 2 (non-functional) | 211726_s_at | 0.93 | 54.53 |  | 0.33 | 0.84 | ↓ | 0.87 | 41.22 |  |
| FGD6 | FYVE, RhoGEF and PH domain containing 6 | 1555137_a_at | 0.93 | 38.54 |  | 0.25 | 0.53 | ↓ | 1.35 | 17.70 |  |
| ZNF268 | zinc finger protein 268 | 209989_at | 0.93 | 50.55 |  | 2.23 | 1.98 | ↑ | 1.07 | 54.85 |  |
| ETV1 | ets variant 1 | 217061_s_at | 0.93 | 38.54 |  | 0.31 | 0.53 | ↓ | 1.20 | 46.35 |  |
| SEC22B | SEC22 vesicle trafficking protein homolog B (S. cerevisiae) (gene/pseudogene) | 209207_s_at | 0.93 | 62.00 |  | 2.47 | 2.80 | ↑ | 0.99 | 57.92 |  |
| WSB2 | WD repeat and SOCS box-containing 2 | 213734_at | 0.92 | 55.60 |  | 2.51 | 1.07 | ↑ | 1.16 | 48.62 |  |
| VAMP3 | vesicle-associated membrane protein 3 (cellubrevin) | 211749_s_at | 0.92 | 47.41 |  | 0.42 | 0.29 | ↓ | 1.16 | 38.27 |  |
| CYP1B1 | cytochrome P450, family 1, subfamily B, polypeptide 1 | 202434_s_at | 0.92 | 42.20 |  | 0.01 | 0.00 | ↓ | 1.26 | 52.56 |  |
| CDC42 | cell division cycle 42 (GTP binding protein, 25kDa) | 208728_s_at | 0.92 | 48.99 |  | 2.59 | 0.36 | ↑ | 0.93 | 46.35 |  |
| TRIM34 /// TRIM6-TRIM34 | tripartite motif-containing 34 /// TRIM6-TRIM34 readthrough | 224175_s_at | 0.92 | 54.53 |  | 0.49 | 2.30 | ↓ | 0.84 | 35.63 |  |
| CUL4B | cullin 4B | 210257_x_at | 0.92 | 53.22 |  | 0.44 | 1.26 | ↓ | 1.04 | 57.92 |  |
| ZNF594 | zinc finger protein 594 | 235690_at | 0.92 | 56.74 |  | 1.80 | 10.02 |  | 0.43 | 4.16 | ↓ |
| PLAC9 | placenta-specific 9 | 227419_x_at | 0.92 | 53.22 |  | 2.23 | 3.35 | ↑ | 1.45 | 54.08 |  |
| SAE1 | SUMO1 activating enzyme subunit 1 | 1555618_s_at | 0.92 | 45.69 |  | 0.32 | 0.18 | ↓ | 0.81 | 15.27 |  |
| DNER | delta/notch-like EGF repeat containing | 226281_at | 0.92 | 42.20 |  | 0.25 | 6.80 | ↓ | 2.61 | 26.48 |  |
| GORASP2 | golgi reassembly stacking protein 2, 55kDa | 208842_s_at | 0.92 | 45.69 |  | 0.45 | 0.36 | ↓ | 1.09 | 46.35 |  |
| TMPO | thymopoietin | 209753_s_at | 0.92 | 54.53 |  | 0.48 | 2.30 | ↓ | 0.38 | 0.21 | ↓ |
| SH3D19 | SH3 domain containing 19 | 1558647_at | 0.92 | 43.91 |  | 0.20 | 0.25 | ↓ | 1.39 | 43.83 |  |
| CDCP1 | CUB domain containing protein 1 | 218451_at | 0.92 | 58.62 |  | 0.39 | 3.81 | ↓ | 1.05 | 55.52 |  |
| CAPG | capping protein (actin filament), gelsolin-like | 201850_at | 0.92 | 47.41 |  | 0.50 | 3.81 | ↓ | 0.80 | 29.62 |  |
| PIK3IP1 | phosphoinositide-3-kinase interacting protein 1 | 221756_at | 0.92 | 47.41 |  | 0.79 | 26.77 |  | 2.56 | 1.25 | ↑ |
| VAMP3 | vesicle-associated membrane protein 3 (cellubrevin) | 201337_s_at | 0.92 | 45.69 |  | 0.31 | 0.00 | ↓ | 1.39 | 15.27 |  |
| FKBP7 | FK506 binding protein 7 | 223667_at | 0.92 | 47.41 |  | 0.31 | 0.18 | ↓ | 0.56 | 4.84 |  |
| C13orf34 | chromosome 13 open reading frame 34 | 219544_at | 0.92 | 54.53 |  | 0.73 | 22.17 |  | 0.48 | 1.43 | ↓ |
| NOTCH2 | notch 2 | 210756_s_at | 0.92 | 47.41 |  | 0.30 | 0.00 | ↓ | 0.59 | 3.36 |  |
| HBS1L | HBS1-like (S. cerevisiae) | 209314_s_at | 0.92 | 43.91 |  | 2.19 | 0.84 | ↑ | 1.32 | 41.22 |  |
| NEK9 | NIMA (never in mitosis gene a)- related kinase 9 | 214738_s_at | 0.92 | 40.42 |  | 0.10 | 0.00 | ↓ | 0.66 | 7.00 |  |
| PIGO | phosphatidylinositol glycan anchor biosynthesis, class O | 214991_s_at | 0.92 | 51.95 |  | 0.40 | 3.81 | ↓ | 1.09 | 57.14 |  |
| KAT5 | K(lysine) acetyltransferase 5 | 214258_x_at | 0.92 | 45.69 |  | 0.47 | 0.60 | ↓ | 0.90 | 35.63 |  |
| C1S | complement component 1, s subcomponent | 1555229_a_at | 0.92 | 57.71 |  | 0.19 | 0.18 | ↓ | 1.20 | 43.83 |  |
| UTRN | utrophin | 213022_s_at | 0.92 | 42.20 |  | 0.42 | 1.26 | ↓ | 1.82 | 7.00 |  |
| MEF2A | myocyte enhancer factor 2A | 214684_at | 0.92 | 47.41 |  | 1.33 | 19.82 |  | 2.02 | 2.70 | ↑ |
| DDA1 | DET1 and DDB1 associated 1 | 218260_at | 0.92 | 50.55 |  | 0.22 | 0.18 | ↓ | 1.19 | 41.22 |  |
| COPA | coatomer protein complex, subunit alpha | 214336_s_at | 0.92 | 45.69 |  | 0.22 | 0.18 | ↓ | 1.30 | 38.27 |  |
| BAALC | brain and acute leukemia, cytoplasmic | 222780_s_at | 0.92 | 58.62 |  | 0.47 | 6.80 | ↓ | 0.22 | 1.43 | ↓ |
| SULT1A1 | sulfotransferase family, cytosolic, 1A, phenol-preferring, member 1 | 203615_x_at | 0.92 | 55.60 |  | 1.53 | 19.82 |  | 0.43 | 2.70 | ↓ |
| MGEA5 | Meningioma expressed antigen 5 (hyaluronidase) | 235868_at | 0.92 | 36.49 |  | 0.33 | 0.84 | ↓ | 0.92 | 54.08 |  |
| SNAP23 | Synaptosomal-associated protein, 23kDa | 229773_at | 0.92 | 53.22 |  | 0.81 | 38.78 |  | 0.47 | 4.16 | ↓ |
| MICAL2 | microtubule associated monoxygenase, calponin and LIM domain containing 2 | 206275_s_at | 0.92 | 62.00 |  | 0.17 | 0.70 | ↓ | 1.22 | 41.22 |  |
| DOCK5 | Dedicator of cytokinesis 5 | 230207_s_at | 0.91 | 50.55 |  | 0.38 | 0.70 | ↓ | 0.95 | 54.08 |  |
| LIN7C | lin-7 homolog C (C. elegans) | 219399_at | 0.91 | 56.74 |  | 0.45 | 4.45 | ↓ | 1.21 | 48.62 |  |
| RBPMS2 | RNA binding protein with multiple splicing 2 | 228802_at | 0.91 | 50.55 |  | 0.95 | 60.46 |  | 0.28 | 0.43 | ↓ |
| EML4 | echinoderm microtubule associated protein like 4 | 220386_s_at | 0.91 | 50.55 |  | 0.36 | 0.76 | ↓ | 0.64 | 11.20 |  |
| MND1 | meiotic nuclear divisions 1 homolog (S. cerevisiae) | 223700_at | 0.91 | 40.42 |  | 1.00 | 58.42 |  | 0.22 | 0.00 | ↓ |
| MGAM | maltase-glucoamylase (alpha-glucosidase) | 206522_at | 0.91 | 58.62 |  | 1.47 | 31.53 |  | 2.23 | 8.02 | ↑ |
| BRCA2 | breast cancer 2, early onset | 214727_at | 0.91 | 51.95 |  | 0.73 | 29.01 |  | 0.32 | 0.21 | ↓ |
| MPHOSPH8 | M-phase phosphoprotein 8 | 225041_at | 0.91 | 47.41 |  | 2.00 | 1.26 | ↑ | 0.96 | 50.73 |  |
| WNT16 | wingless-type MMTV integration site family, member 16 | 224022_x_at | 0.91 | 43.91 |  | 0.11 | 1.74 | ↓ | 6.27 | 26.48 |  |
| TMOD3 | tropomodulin 3 (ubiquitous) | 223078_s_at | 0.91 | 50.55 |  | 0.48 | 1.74 | ↓ | 1.23 | 48.62 |  |
| SMAD3 | SMAD family member 3 | 205398_s_at | 0.91 | 45.69 |  | 0.44 | 1.26 | ↓ | 0.80 | 26.48 |  |
| PPIF | peptidylprolyl isomerase F | 201490_s_at | 0.91 | 62.00 |  | 0.37 | 2.30 | ↓ | 0.92 | 58.81 |  |
| --- | --- | 229787_s_at | 0.91 | 55.60 |  | 2.30 | 2.30 | ↑ | 0.89 | 46.35 |  |
| TMEM64 | transmembrane protein 64 | 242338_at | 0.91 | 57.71 |  | 0.39 | 2.30 | ↓ | 0.68 | 23.22 |  |
| HSPA4 | heat shock 70kDa protein 4 | 211016_x_at | 0.91 | 50.55 |  | 0.20 | 0.00 | ↓ | 1.06 | 57.14 |  |
| C13orf1 | chromosome 13 open reading frame 1 | 214668_at | 0.91 | 42.20 |  | 0.48 | 0.40 | ↓ | 1.02 | 58.81 |  |
| EPB41L2 | erythrocyte membrane protein band 4.1-like 2 | 201718_s_at | 0.91 | 47.41 |  | 0.49 | 1.98 | ↓ | 0.95 | 50.73 |  |
| PTPN22 | protein tyrosine phosphatase, non-receptor type 22 (lymphoid) | 206060_s_at | 0.91 | 40.42 |  | 0.28 | 1.74 | ↓ | 2.38 | 20.38 |  |
| LOC96610 | BMS1 homolog, ribosome assembly protein (yeast) pseudogene | 1569013_s_at | 0.91 | 48.99 |  | 0.45 | 1.74 | ↓ | 0.80 | 35.63 |  |
| CDC25C | cell division cycle 25 homolog C (S. pombe) | 205167_s_at | 0.91 | 51.95 |  | 0.81 | 29.01 |  | 0.10 | 0.00 | ↓ |
| MR1 | major histocompatibility complex, class I-related | 210223_s_at | 0.91 | 47.41 |  | 0.48 | 2.30 | ↓ | 1.01 | 58.51 |  |
| PCM1 | pericentriolar material 1 | 209997_x_at | 0.91 | 48.99 |  | 0.37 | 0.25 | ↓ | 0.91 | 43.83 |  |
| USP4 | ubiquitin specific peptidase 4 (proto-oncogene) | 202682_s_at | 0.91 | 40.42 |  | 2.15 | 0.40 | ↑ | 0.92 | 38.27 |  |
| SLC39A6 | solute carrier family 39 (zinc transporter), member 6 | 1556551_s_at | 0.91 | 43.91 |  | 0.16 | 0.00 | ↓ | 1.23 | 38.27 |  |
| ZNF654 | zinc finger protein 654 | 219239_s_at | 0.91 | 45.69 |  | 2.74 | 0.76 | ↑ | 0.87 | 38.27 |  |
| ENAH | enabled homolog (Drosophila) | 217820_s_at | 0.91 | 47.41 |  | 2.43 | 0.53 | ↑ | 1.14 | 54.85 |  |
| CHD1L | chromodomain helicase DNA binding protein 1-like | 207645_s_at | 0.91 | 57.71 |  | 0.32 | 1.26 | ↓ | 0.74 | 29.62 |  |
| NUFIP2 | nuclear fragile X mental retardation protein interacting protein 2 | 224956_at | 0.91 | 47.41 |  | 2.30 | 1.74 | ↑ | 1.27 | 26.48 |  |
| GALNT1 | UDP-N-acetyl-alpha-D-galactosamine:polypeptide N-acetylgalactosaminyltransferase 1 (GalNAc-T1) | 201724_s_at | 0.91 | 50.55 |  | 1.43 | 17.74 |  | 0.38 | 1.25 | ↓ |
| NFX1 | nuclear transcription factor, X-box binding 1 | 1553348_a_at | 0.91 | 45.69 |  | 0.28 | 0.00 | ↓ | 0.89 | 41.22 |  |
| WHSC1 | Wolf-Hirschhorn syndrome candidate 1 | 209054_s_at | 0.91 | 42.20 |  | 1.33 | 24.54 |  | 0.39 | 0.00 | ↓ |
| RIF1 | RAP1 interacting factor homolog (yeast) | 236620_at | 0.90 | 47.41 |  | 2.31 | 1.74 | ↑ | 0.80 | 29.62 |  |
| CCDC88A | coiled-coil domain containing 88A | 219387_at | 0.90 | 47.41 |  | 2.63 | 0.98 | ↑ | 0.84 | 32.58 |  |
| MAGED4 /// MAGED4B | melanoma antigen family D, 4 /// melanoma antigen family D, 4B | 221261_x_at | 0.90 | 56.74 |  | 0.81 | 41.29 |  | 0.17 | 0.00 | ↓ |
| ODZ3 | odz, odd Oz/ten-m homolog 3 (Drosophila) | 219523_s_at | 0.90 | 51.95 |  | 0.52 | 3.81 |  | 2.46 | 1.99 | ↑ |
| GFM2 | G elongation factor, mitochondrial 2 | 231918_s_at | 0.90 | 45.69 |  | 0.28 | 0.18 | ↓ | 1.19 | 50.73 |  |
| KTN1 | kinectin 1 (kinesin receptor) | 200914_x_at | 0.90 | 43.91 |  | 2.03 | 1.26 | ↑ | 1.09 | 50.73 |  |
| QSER1 | glutamine and serine rich 1 | 244563_at | 0.90 | 42.20 |  | 0.50 | 2.30 | ↓ | 0.80 | 29.62 |  |
| C16orf63 | chromosome 16 open reading frame 63 | 225088_at | 0.90 | 42.20 |  | 0.40 | 0.40 | ↓ | 1.09 | 54.85 |  |
| ATXN7 | ataxin 7 | 243259_at | 0.90 | 47.41 |  | 2.38 | 0.98 | ↑ | 0.90 | 43.83 |  |
| NRP2 | Neuropilin 2 | 228699_at | 0.90 | 43.91 |  | 2.93 | 0.70 | ↑ | 1.36 | 29.62 |  |
| XPNPEP3 | X-prolyl aminopeptidase (aminopeptidase P) 3, putative | 220020_at | 0.90 | 58.62 |  | 0.43 | 3.35 | ↓ | 0.92 | 50.73 |  |
| TFRC | transferrin receptor (p90, CD71) | 237215_s_at | 0.90 | 58.62 |  | 0.22 | 2.30 | ↓ | 0.90 | 50.73 |  |
| BANF1 | barrier to autointegration factor 1 | 210125_s_at | 0.90 | 43.91 |  | 0.18 | 0.00 | ↓ | 0.78 | 11.20 |  |
| FXR1 | fragile X mental retardation, autosomal homolog 1 | 201635_s_at | 0.90 | 42.20 |  | 0.44 | 0.25 | ↓ | 1.18 | 38.27 |  |
| HIST1H2BC | histone cluster 1, H2bc | 214455_at | 0.90 | 50.55 |  | 1.08 | 52.07 |  | 2.90 | 2.70 | ↑ |
| SMARCC1 | SWI/SNF related, matrix associated, actin dependent regulator of chromatin, subfamily c, member 1 | 201072_s_at | 0.90 | 45.69 |  | 4.00 | 0.21 | ↑ | 0.74 | 26.48 |  |
| CEP170 /// CEP170P1 | centrosomal protein 170kDa /// centrosomal protein 170kDa pseudogene 1 | 1552717_s_at | 0.90 | 54.53 |  | 0.18 | 0.18 | ↓ | 1.23 | 54.85 |  |
| CSPP1 | centrosome and spindle pole associated protein 1 | 242041_at | 0.90 | 30.22 |  | 0.52 | 9.01 |  | 0.43 | 2.43 | ↓ |
| LOC100505497 | hypothetical LOC100505497 | 228204_at | 0.90 | 51.95 |  | 2.39 | 1.74 | ↑ | 1.32 | 41.22 |  |
| WIPI2 | WD repeat domain, phosphoinositide interacting 2 | 214699_x_at | 0.90 | 45.69 |  | 0.48 | 0.84 | ↓ | 1.11 | 52.56 |  |
| PTK2 | PTK2 protein tyrosine kinase 2 | 207821_s_at | 0.90 | 42.20 |  | 0.41 | 0.18 | ↓ | 1.22 | 20.38 |  |
| CD46 | CD46 molecule, complement regulatory protein | 211574_s_at | 0.90 | 47.41 |  | 0.28 | 0.00 | ↓ | 1.25 | 32.58 |  |
| PAFAH1B1 | platelet-activating factor acetylhydrolase 1b, regulatory subunit 1 (45kDa) | 211547_s_at | 0.90 | 45.69 |  | 0.39 | 0.36 | ↓ | 1.31 | 29.62 |  |
| HERC4 | hect domain and RLD 4 | 208055_s_at | 0.90 | 45.69 |  | 0.41 | 1.74 | ↓ | 1.33 | 41.22 |  |
| MLEC | malectin | 200617_at | 0.90 | 45.69 |  | 0.40 | 0.25 | ↓ | 0.55 | 1.25 |  |
| TGFB2 | transforming growth factor, beta 2 | 228121_at | 0.90 | 51.95 |  | 1.29 | 24.54 |  | 0.36 | 1.02 | ↓ |
| ERLIN1 | ER lipid raft associated 1 | 202444_s_at | 0.90 | 45.69 |  | 0.21 | 0.00 | ↓ | 0.77 | 17.70 |  |
| NACA | nascent polypeptide-associated complex alpha subunit | 222018_at | 0.90 | 45.69 |  | 1.15 | 36.20 |  | 0.46 | 5.75 | ↓ |
| EXOC5 | exocyst complex component 5 | 218748_s_at | 0.90 | 47.41 |  | 0.20 | 0.00 | ↓ | 1.04 | 57.64 |  |
| RARRES3 | retinoic acid receptor responder (tazarotene induced) 3 | 204070_at | 0.90 | 56.74 |  | 0.62 | 19.82 |  | 0.46 | 4.84 | ↓ |
| LARS | leucyl-tRNA synthetase | 223888_s_at | 0.90 | 40.42 |  | 0.35 | 0.60 | ↓ | 1.27 | 43.83 |  |
| LHFP | Lipoma HMGIC fusion partner | 231411_at | 0.89 | 42.20 |  | 2.73 | 0.40 | ↑ | 1.26 | 32.58 |  |
| CLINT1 | clathrin interactor 1 | 230609_at | 0.89 | 47.41 |  | 0.12 | 0.00 | ↓ | 0.80 | 29.62 |  |
| CLIC4 | chloride intracellular channel 4 | 201559_s_at | 0.89 | 45.69 |  | 0.28 | 0.25 | ↓ | 1.91 | 2.70 |  |
| CREB1 | cAMP responsive element binding protein 1 | 214513_s_at | 0.89 | 42.20 |  | 0.39 | 0.18 | ↓ | 0.80 | 15.27 |  |
| FAT4 | FAT tumor suppressor homolog 4 (Drosophila) | 219427_at | 0.89 | 51.95 |  | 2.01 | 6.11 | ↑ | 1.40 | 20.38 |  |
| HP1BP3 | heterochromatin protein 1, binding protein 3 | 220633_s_at | 0.89 | 36.49 |  | 0.20 | 0.00 | ↓ | 0.81 | 23.22 |  |
| NHS | Nance-Horan syndrome (congenital cataracts and dental anomalies) | 242800_at | 0.89 | 53.22 |  | 2.35 | 6.11 | ↑ | 0.78 | 35.63 |  |
| C5orf54 | chromosome 5 open reading frame 54 | 220770_s_at | 0.89 | 40.42 |  | 1.31 | 24.54 |  | 0.33 | 1.61 | ↓ |
| C14orf145 | chromosome 14 open reading frame 145 | 244033_at | 0.89 | 40.42 |  | 1.21 | 43.50 |  | 0.32 | 0.34 | ↓ |
| C7orf58 | chromosome 7 open reading frame 58 | 228728_at | 0.89 | 54.53 |  | 0.59 | 17.74 |  | 3.22 | 1.25 | ↑ |
| AMPD3 | adenosine monophosphate deaminase 3 | 207992_s_at | 0.89 | 48.99 |  | 0.46 | 3.81 | ↓ | 3.93 | 0.58 | ↑ |
| COG7 | component of oligomeric golgi complex 7 | 213190_at | 0.89 | 40.42 |  | 0.47 | 0.29 | ↓ | 0.84 | 26.48 |  |
| HIST2H2BE | histone cluster 2, H2be | 202708_s_at | 0.89 | 48.99 |  | 0.79 | 41.29 |  | 2.70 | 1.99 | ↑ |
| C21orf7 | chromosome 21 open reading frame 7 | 221211_s_at | 0.89 | 51.95 |  | 1.64 | 60.56 |  | 3.16 | 1.99 | ↑ |
| DHX9 | DEAH (Asp-Glu-Ala-His) box polypeptide 9 | 212105_s_at | 0.89 | 51.95 |  | 0.07 | 0.00 | ↓ | 1.30 | 41.22 |  |
| PPFIA1 | protein tyrosine phosphatase, receptor type, f polypeptide (PTPRF), interacting protein (liprin), alpha 1 | 210235_s_at | 0.89 | 38.54 |  | 0.43 | 0.18 | ↓ | 1.21 | 29.62 |  |
| ASNA1 | arsA arsenite transporter, ATP-binding, homolog 1 (bacterial) | 202024_at | 0.89 | 45.69 |  | 0.40 | 0.36 | ↓ | 0.86 | 35.63 |  |
| SLC30A5 | solute carrier family 30 (zinc transporter), member 5 | 1555334_s_at | 0.89 | 43.91 |  | 0.45 | 0.53 | ↓ | 0.85 | 29.62 |  |
| YTHDC2 | YTH domain containing 2 | 1568680_s_at | 0.89 | 51.95 |  | 5.31 | 0.00 | ↑ | 1.53 | 17.70 |  |
| B3GNT2 | UDP-GlcNAc:betaGal beta-1,3-N-acetylglucosaminyltransferase 2 | 219326_s_at | 0.89 | 58.62 |  | 0.44 | 3.81 | ↓ | 0.79 | 41.22 |  |
| SORT1 | sortilin 1 | 212797_at | 0.89 | 62.00 |  | 0.10 | 0.00 | ↓ | 1.57 | 17.70 |  |
| MPP5 | membrane protein, palmitoylated 5 (MAGUK p55 subfamily member 5) | 219321_at | 0.89 | 40.42 |  | 0.50 | 0.98 | ↓ | 1.24 | 26.48 |  |
| ACYP1 | acylphosphatase 1, erythrocyte (common) type | 205260_s_at | 0.89 | 53.22 |  | 0.97 | 60.64 |  | 0.42 | 1.61 | ↓ |
| TNKS2 | tankyrase, TRF1-interacting ankyrin-related ADP-ribose polymerase 2 | 222562_s_at | 0.89 | 54.53 |  | 0.47 | 3.35 | ↓ | 1.30 | 41.22 |  |
| CASP1 | caspase 1, apoptosis-related cysteine peptidase (interleukin 1, beta, convertase) | 211367_s_at | 0.89 | 58.62 |  | 0.44 | 9.01 | ↓ | 0.69 | 29.62 |  |
| MPHOSPH9 | M-phase phosphoprotein 9 | 1558369_at | 0.89 | 42.20 |  | 2.22 | 0.98 | ↑ | 0.72 | 11.20 |  |
| WHSC1 | Wolf-Hirschhorn syndrome candidate 1 | 222778_s_at | 0.89 | 45.69 |  | 0.84 | 38.78 |  | 0.43 | 0.21 | ↓ |
| H2AFZ | H2A histone family, member Z | 213911_s_at | 0.89 | 40.42 |  | 1.06 | 56.87 |  | 0.44 | 0.00 | ↓ |
| AP3S2 | adaptor-related protein complex 3, sigma 2 subunit | 213215_at | 0.89 | 40.42 |  | 0.33 | 0.00 | ↓ | 1.65 | 15.27 |  |
| SPAST | spastin | 207724_s_at | 0.89 | 38.54 |  | 0.45 | 0.70 | ↓ | 1.24 | 35.63 |  |
| TJP1 | tight junction protein 1 (zona occludens 1) | 214168_s_at | 0.89 | 42.20 |  | 0.41 | 0.53 | ↓ | 1.28 | 32.58 |  |
| TCF3 | transcription factor 3 (E2A immunoglobulin enhancer binding factors E12/E47) | 209152_s_at | 0.88 | 42.20 |  | 1.28 | 26.77 |  | 0.44 | 0.34 | ↓ |
| C10orf136 | chromosome 10 open reading frame 136 | 1560851_at | 0.88 | 61.35 |  | 1.23 | 33.65 |  | 0.08 | 0.34 | ↓ |
| C22orf27 | chromosome 22 open reading frame 27 | 230566_at | 0.88 | 42.20 |  | 0.47 | 0.60 | ↓ | 0.97 | 50.73 |  |
| UBE2L6 | ubiquitin-conjugating enzyme E2L 6 | 201649_at | 0.88 | 54.53 |  | 0.46 | 3.35 | ↓ | 0.76 | 32.58 |  |
| LOC100507279 /// LOC728323 /// NCRNA00266 /// PCMTD2 | hypothetical LOC100507279 /// hypothetical LOC728323 /// non-protein coding RNA 266 /// protein-L-isoaspartate (D-aspartate) O-methyltransferase domain containing 2 | 232953_at | 0.88 | 38.54 |  | 0.26 | 0.29 | ↓ | 0.63 | 8.02 |  |
| ANKRD6 | ankyrin repeat domain 6 | 204672_s_at | 0.88 | 55.60 |  | 1.68 | 26.77 |  | 2.56 | 2.43 | ↑ |
| ZMYM6 | zinc finger, MYM-type 6 | 1552970_s_at | 0.88 | 51.95 |  | 0.49 | 3.81 | ↓ | 1.36 | 41.22 |  |
| SGMS2 | sphingomyelin synthase 2 | 243141_at | 0.88 | 43.91 |  | 0.40 | 1.74 | ↓ | 1.74 | 7.00 |  |
| ATAD2 | ATPase family, AAA domain containing 2 | 222740_at | 0.88 | 40.42 |  | 1.14 | 52.07 |  | 0.42 | 0.50 | ↓ |
| AASS | aminoadipate-semialdehyde synthase | 210852_s_at | 0.88 | 48.99 |  | 1.18 | 38.78 |  | 0.44 | 2.43 | ↓ |
| CDC25B | cell division cycle 25 homolog B (S. pombe) | 201853_s_at | 0.88 | 42.20 |  | 0.68 | 9.01 |  | 0.44 | 0.00 | ↓ |
| PTMA | prothymosin, alpha | 200772_x_at | 0.88 | 43.91 |  | 2.46 | 0.70 | ↑ | 0.45 | 0.21 | ↓ |
| KLF7 | Kruppel-like factor 7 (ubiquitous) | 204334_at | 0.88 | 40.42 |  | 2.01 | 4.45 | ↑ | 1.00 | 54.08 |  |
| MAPRE1 | microtubule-associated protein, RP/EB family, member 1 | 200712_s_at | 0.88 | 42.20 |  | 0.49 | 1.45 | ↓ | 0.88 | 38.27 |  |
| G3BP2 | GTPase activating protein (SH3 domain) binding protein 2 | 206383_s_at | 0.88 | 53.22 |  | 0.31 | 0.36 | ↓ | 1.06 | 56.65 |  |
| C20orf30 | chromosome 20 open reading frame 30 | 220477_s_at | 0.88 | 42.20 |  | 0.49 | 2.30 | ↓ | 0.85 | 35.63 |  |
| NCOA2 | nuclear receptor coactivator 2 | 205732_s_at | 0.88 | 43.91 |  | 0.21 | 0.00 | ↓ | 1.28 | 29.62 |  |
| FKBP5 | FK506 binding protein 5 | 224840_at | 0.88 | 47.41 |  | 1.32 | 33.65 |  | 0.30 | 0.00 | ↓ |
| ZNF780A /// ZNF780B | zinc finger protein 780A /// zinc finger protein 780B | 215570_s_at | 0.88 | 54.53 |  | 0.34 | 1.07 | ↓ | 0.93 | 54.08 |  |
| CCL5 | chemokine (C-C motif) ligand 5 | 1555759_a_at | 0.88 | 53.22 |  | 0.48 | 6.80 | ↓ | 1.25 | 50.73 |  |
| AMIGO2 | adhesion molecule with Ig-like domain 2 | 222108_at | 0.88 | 56.74 |  | 2.30 | 6.11 | ↑ | 1.21 | 50.73 |  |
| ATXN7 | ataxin 7 | 209964_s_at | 0.88 | 56.74 |  | 0.36 | 0.84 | ↓ | 1.11 | 55.52 |  |
| FABP5 | fatty acid binding protein 5 (psoriasis-associated) | 202345_s_at | 0.88 | 54.53 |  | 1.13 | 47.13 |  | 0.18 | 0.00 | ↓ |
| CD46 | CD46 molecule, complement regulatory protein | 207549_x_at | 0.88 | 42.20 |  | 0.25 | 0.00 | ↓ | 1.13 | 52.56 |  |
| MEX3C | mex-3 homolog C (C. elegans) | 222567_s_at | 0.88 | 42.20 |  | 0.32 | 0.53 | ↓ | 0.78 | 26.48 |  |
| SIPA1L2 | signal-induced proliferation-associated 1 like 2 | 225056_at | 0.88 | 38.54 |  | 1.03 | 38.78 |  | 0.18 | 0.00 | ↓ |
| TRIM5 | tripartite motif-containing 5 | 210705_s_at | 0.88 | 48.99 |  | 1.17 | 38.78 |  | 0.49 | 1.99 | ↓ |
| CCDC8 | coiled-coil domain containing 8 | 223496_s_at | 0.88 | 42.20 |  | 1.28 | 38.78 |  | 0.27 | 1.25 | ↓ |
| GTF3A | general transcription factor IIIA | 238880_at | 0.88 | 40.42 |  | 0.83 | 31.53 |  | 0.47 | 0.50 | ↓ |
| PSPH | phosphoserine phosphatase | 205194_at | 0.88 | 61.53 |  | 2.17 | 9.01 | ↑ | 0.42 | 8.02 | ↓ |
| ADPGK | ADP-dependent glucokinase | 224455_s_at | 0.88 | 36.49 |  | 0.45 | 0.76 | ↓ | 1.05 | 58.51 |  |
| INO80D | INO80 complex subunit D | 227924_at | 0.88 | 48.99 |  | 0.48 | 2.30 | ↓ | 1.15 | 50.73 |  |
| MAPK1 | mitogen-activated protein kinase 1 | 208351_s_at | 0.88 | 47.41 |  | 0.26 | 0.18 | ↓ | 0.97 | 58.81 |  |
| PHTF2 | putative homeodomain transcription factor 2 | 217097_s_at | 0.88 | 48.99 |  | 0.15 | 0.00 | ↓ | 1.94 | 11.20 |  |
| TNFAIP3 | tumor necrosis factor, alpha-induced protein 3 | 202643_s_at | 0.88 | 48.99 |  | 0.43 | 1.74 | ↓ | 0.89 | 35.63 |  |
| USP32 | ubiquitin specific peptidase 32 | 244871_s_at | 0.88 | 45.69 |  | 0.46 | 1.98 | ↓ | 0.90 | 43.83 |  |
| SFRS18 | splicing factor, arginine/serine-rich 18 | 212177_at | 0.88 | 45.69 |  | 1.69 | 17.74 |  | 0.43 | 1.61 | ↓ |
| EPR1 | effector cell peptidase receptor 1 (non-protein coding) | 1555826_at | 0.88 | 42.20 |  | 0.62 | 11.08 |  | 0.35 | 1.02 | ↓ |
| UBR1 | ubiquitin protein ligase E3 component n-recognin 1 | 238528_at | 0.88 | 40.42 |  | 0.47 | 0.70 | ↓ | 1.05 | 57.14 |  |
| C14orf101 | chromosome 14 open reading frame 101 | 219757_s_at | 0.88 | 45.69 |  | 0.38 | 0.36 | ↓ | 0.73 | 15.27 |  |
| TRAM1 | translocation associated membrane protein 1 | 201399_s_at | 0.88 | 38.54 |  | 0.43 | 0.18 | ↓ | 1.34 | 17.70 |  |
| NOTCH2NL | notch 2 N-terminal like | 227067_x_at | 0.88 | 62.00 |  | 0.63 | 33.65 |  | 0.47 | 8.02 | ↓ |
| EPB41L4B | erythrocyte membrane protein band 4.1 like 4B | 223427_s_at | 0.87 | 42.20 |  | 0.43 | 0.76 | ↓ | 1.08 | 58.81 |  |
| PTPN9 | Protein tyrosine phosphatase, non-receptor type 9 | 233226_at | 0.87 | 51.95 |  | 0.47 | 9.01 | ↓ | 1.00 | 52.56 |  |
| USP53 | ubiquitin specific peptidase 53 | 231817_at | 0.87 | 45.69 |  | 0.87 | 45.34 |  | 3.19 | 1.02 | ↑ |
| PDXDC1 | pyridoxal-dependent decarboxylase domain containing 1 | 1560013_at | 0.87 | 40.42 |  | 0.68 | 14.26 |  | 0.48 | 1.61 | ↓ |
| C13orf31 | chromosome 13 open reading frame 31 | 1553142_at | 0.87 | 42.20 |  | 0.56 | 6.80 |  | 2.66 | 2.43 | ↑ |
| NIN | ninein (GSK3B interacting protein) | 234299_s_at | 0.87 | 38.54 |  | 0.46 | 1.98 | ↓ | 0.76 | 23.22 |  |
| TMEM30A | transmembrane protein 30A | 232591_s_at | 0.87 | 47.41 |  | 0.41 | 1.07 | ↓ | 1.17 | 46.35 |  |
| GSPT1 | G1 to S phase transition 1 | 234975_at | 0.87 | 36.49 |  | 0.29 | 0.00 | ↓ | 1.25 | 23.22 |  |
| C13orf31 | chromosome 13 open reading frame 31 | 228937_at | 0.87 | 43.91 |  | 1.15 | 53.46 |  | 2.73 | 1.02 | ↑ |
| GABPA | GA binding protein transcription factor, alpha subunit 60kDa | 227428_at | 0.87 | 38.54 |  | 2.08 | 0.84 | ↑ | 0.88 | 32.58 |  |
| COPB2 | coatomer protein complex, subunit beta 2 (beta prime) | 226256_at | 0.87 | 40.42 |  | 0.34 | 2.30 | ↓ | 1.18 | 43.83 |  |
| SULF2 | sulfatase 2 | 233555_s_at | 0.87 | 62.00 |  | 0.59 | 26.77 |  | 0.35 | 4.84 | ↓ |
| C6orf26 /// MSH5 | chromosome 6 open reading frame 26 /// mutS homolog 5 (E. coli) | 210410_s_at | 0.87 | 38.54 |  | 0.51 | 0.76 |  | 0.14 | 0.00 | ↓ |
| SURF4 | surfeit 4 | 222979_s_at | 0.87 | 42.20 |  | 0.43 | 0.70 | ↓ | 0.94 | 48.62 |  |
| FAM72A /// FAM72B /// FAM72C /// FAM72D | family with sequence similarity 72, member A /// family with sequence similarity 72, member B /// family with sequence similarity 72, member C /// family with sequence similarity 72, member D | 225834_at | 0.87 | 42.20 |  | 0.79 | 31.53 |  | 0.21 | 0.00 | ↓ |
| --- | --- | 213789_at | 0.87 | 51.95 |  | 0.27 | 0.76 | ↓ | 0.74 | 26.48 |  |
| TCF3 | transcription factor 3 (E2A immunoglobulin enhancer binding factors E12/E47) | 213811_x_at | 0.87 | 43.91 |  | 1.57 | 15.95 |  | 0.49 | 1.25 | ↓ |
| HTATSF1 | HIV-1 Tat specific factor 1 | 202601_s_at | 0.87 | 36.49 |  | 0.25 | 0.00 | ↓ | 0.98 | 48.62 |  |
| CPD | carboxypeptidase D | 201942_s_at | 0.87 | 42.20 |  | 0.26 | 0.00 | ↓ | 1.18 | 38.27 |  |
| SIPA1L2 | signal-induced proliferation-associated 1 like 2 | 233587_s_at | 0.87 | 40.42 |  | 0.90 | 38.78 |  | 0.41 | 1.43 | ↓ |
| MC4R | melanocortin 4 receptor | 221467_at | 0.87 | 34.35 |  | 0.49 | 5.22 | ↓ | 1.08 | 48.62 |  |
| OSTM1 | osteopetrosis associated transmembrane protein 1 | 243287_s_at | 0.87 | 50.55 |  | 0.91 | 57.71 |  | 2.33 | 2.43 | ↑ |
| ESPL1 | extra spindle pole bodies homolog 1 (S. cerevisiae) | 38158_at | 0.87 | 40.42 |  | 0.81 | 31.53 |  | 0.44 | 0.00 | ↓ |
| SRSF4 | serine/arginine-rich splicing factor 4 | 239511_s_at | 0.87 | 47.41 |  | 0.46 | 7.87 | ↓ | 0.93 | 46.35 |  |
| EGFR | epidermal growth factor receptor | 201983_s_at | 0.87 | 62.00 |  | 0.34 | 2.80 | ↓ | 1.48 | 29.62 |  |
| FAM38B | family with sequence similarity 38, member B | 219602_s_at | 0.87 | 43.91 |  | 1.77 | 50.64 |  | 0.23 | 0.58 | ↓ |
| NUCKS1 | nuclear casein kinase and cyclin-dependent kinase substrate 1 | 222424_s_at | 0.87 | 62.00 |  | 2.93 | 2.80 | ↑ | 0.76 | 43.83 |  |
| FZD8 | frizzled homolog 8 (Drosophila) | 227405_s_at | 0.87 | 43.91 |  | 1.61 | 12.66 |  | 0.40 | 0.34 | ↓ |
| PTN | pleiotrophin | 209466_x_at | 0.87 | 45.69 |  | 1.63 | 19.82 |  | 0.34 | 0.21 | ↓ |
| NSUN6 | NOP2/Sun domain family, member 6 | 222128_at | 0.87 | 32.33 |  | 1.21 | 57.71 |  | 0.39 | 0.89 | ↓ |
| FLJ10038 | hypothetical protein FLJ10038 | 205511_at | 0.87 | 57.71 |  | 3.69 | 2.80 | ↑ | 0.95 | 58.81 |  |
| NAP1L4 | nucleosome assembly protein 1-like 4 | 1560339_s_at | 0.87 | 34.35 |  | 0.43 | 0.18 | ↓ | 0.89 | 32.58 |  |
| LOC650794 | hypothetical LOC650794 | 236837_x_at | 0.87 | 36.49 |  | 2.07 | 0.70 | ↑ | 0.71 | 8.02 |  |
| PHF17 | PHD finger protein 17 | 225816_at | 0.87 | 48.99 |  | 1.53 | 22.17 |  | 2.09 | 1.43 | ↑ |
| KCTD20 | potassium channel tetramerisation domain containing 20 | 214849_at | 0.87 | 47.41 |  | 0.31 | 0.45 | ↓ | 2.98 | 0.50 | ↑ |
| SNHG12 | small nucleolar RNA host gene 12 (non-protein coding) | 228990_at | 0.87 | 40.42 |  | 1.02 | 60.62 |  | 0.43 | 0.34 | ↓ |
| RALB | v-ral simian leukemia viral oncogene homolog B (ras related; GTP binding protein) | 202101_s_at | 0.87 | 36.49 |  | 0.42 | 0.36 | ↓ | 1.15 | 38.27 |  |
| FNIP1 /// RAPGEF6 | folliculin interacting protein 1 /// Rap guanine nucleotide exchange factor (GEF) 6 | 1555247_a_at | 0.87 | 45.69 |  | 2.47 | 1.74 | ↑ | 1.38 | 35.63 |  |
| CTNNB1 | catenin (cadherin-associated protein), beta 1, 88kDa | 1554411_at | 0.87 | 53.22 |  | 3.70 | 0.60 | ↑ | 0.83 | 41.22 |  |
| ATG16L1 | ATG16 autophagy related 16-like 1 (S. cerevisiae) | 220521_s_at | 0.87 | 45.69 |  | 0.49 | 4.45 | ↓ | 0.73 | 20.38 |  |
| PDE8A | phosphodiesterase 8A | 1552931_a_at | 0.87 | 36.49 |  | 0.36 | 0.25 | ↓ | 0.73 | 9.54 |  |
| SPEN | spen homolog, transcriptional regulator (Drosophila) | 201996_s_at | 0.86 | 53.22 |  | 2.25 | 4.45 | ↑ | 1.43 | 20.38 |  |
| EMP3 | epithelial membrane protein 3 | 203729_at | 0.86 | 42.20 |  | 0.29 | 0.25 | ↓ | 1.12 | 50.73 |  |
| MED20 | mediator complex subunit 20 | 206961_s_at | 0.86 | 42.20 |  | 0.30 | 0.36 | ↓ | 0.91 | 50.73 |  |
| SRGAP1 | SLIT-ROBO Rho GTPase activating protein 1 | 233888_s_at | 0.86 | 58.62 |  | 0.39 | 4.45 | ↓ | 1.33 | 32.58 |  |
| ABR | active BCR-related gene | 214671_s_at | 0.86 | 40.42 |  | 0.18 | 0.00 | ↓ | 0.84 | 29.62 |  |
| GALNT2 | UDP-N-acetyl-alpha-D-galactosamine:polypeptide N-acetylgalactosaminyltransferase 2 (GalNAc-T2) | 217787_s_at | 0.86 | 36.49 |  | 0.38 | 0.29 | ↓ | 1.07 | 57.14 |  |
| PPP5C | protein phosphatase 5, catalytic subunit | 201979_s_at | 0.86 | 36.49 |  | 0.34 | 0.00 | ↓ | 0.92 | 41.22 |  |
| TRAPPC10 | trafficking protein particle complex 10 | 1555446_s_at | 0.86 | 48.99 |  | 2.36 | 1.74 | ↑ | 1.12 | 58.81 |  |
| PDE5A | phosphodiesterase 5A, cGMP-specific | 1553175_s_at | 0.86 | 42.20 |  | 0.35 | 0.45 | ↓ | 1.78 | 13.22 |  |
| PRKACB | protein kinase, cAMP-dependent, catalytic, beta | 202742_s_at | 0.86 | 36.49 |  | 0.39 | 0.18 | ↓ | 1.24 | 17.70 |  |
| ATP2B4 | ATPase, Ca++ transporting, plasma membrane 4 | 212135_s_at | 0.86 | 34.35 |  | 0.44 | 0.70 | ↓ | 1.42 | 7.00 |  |
| --- | --- | 230028_at | 0.86 | 42.20 |  | 0.45 | 2.80 | ↓ | 1.28 | 38.27 |  |
| IPP | intracisternal A particle-promoted polypeptide | 1554740_a_at | 0.86 | 42.20 |  | 0.49 | 1.07 | ↓ | 1.13 | 48.62 |  |
| RPL32P3 | ribosomal protein L32 pseudogene 3 | 235314_at | 0.86 | 36.49 |  | 0.61 | 5.22 |  | 0.45 | 0.00 | ↓ |
| TFPI | tissue factor pathway inhibitor (lipoprotein-associated coagulation inhibitor) | 209676_at | 0.86 | 36.49 |  | 0.37 | 0.98 | ↓ | 1.08 | 46.35 |  |
| ARHGDIB | Rho GDP dissociation inhibitor (GDI) beta | 1555812_a_at | 0.86 | 45.69 |  | 0.39 | 4.45 | ↓ | 0.42 | 9.54 | ↓ |
| C18orf54 | Chromosome 18 open reading frame 54 | 241733_at | 0.86 | 36.49 |  | 0.55 | 4.45 |  | 0.34 | 0.00 | ↓ |
| PLA2G4A | phospholipase A2, group IVA (cytosolic, calcium-dependent) | 210145_at | 0.86 | 60.72 |  | 0.98 | 55.85 |  | 0.18 | 1.02 | ↓ |
| MAX | MYC associated factor X | 210734_x_at | 0.86 | 45.69 |  | 0.40 | 0.98 | ↓ | 1.35 | 26.48 |  |
| --- | --- | 234562_x_at | 0.86 | 38.54 |  | 2.70 | 1.74 | ↑ | 0.86 | 35.63 |  |
| WSB1 | WD repeat and SOCS box-containing 1 | 213406_at | 0.86 | 42.20 |  | 0.48 | 4.45 | ↓ | 0.94 | 50.73 |  |
| RASA2 | RAS p21 protein activator 2 | 230669_at | 0.86 | 43.91 |  | 0.37 | 0.29 | ↓ | 1.47 | 13.22 |  |
| KBTBD4 | kelch repeat and BTB (POZ) domain containing 4 | 223765_s_at | 0.86 | 32.33 |  | 0.25 | 0.45 | ↓ | 1.30 | 13.22 |  |
| PCIF1 | PDX1 C-terminal inhibiting factor 1 | 222045_s_at | 0.86 | 50.55 |  | 0.40 | 2.80 | ↓ | 0.63 | 17.70 |  |
| HNRNPL | heterogeneous nuclear ribonucleoprotein L | 35201_at | 0.86 | 40.42 |  | 2.46 | 1.07 | ↑ | 0.80 | 20.38 |  |
| TPR | translocated promoter region (to activated MET oncogene) | 1557227_s_at | 0.86 | 48.99 |  | 0.43 | 2.80 | ↓ | 1.07 | 52.56 |  |
| CHST11 | carbohydrate (chondroitin 4) sulfotransferase 11 | 219634_at | 0.86 | 50.55 |  | 1.01 | 53.46 |  | 0.20 | 1.02 | ↓ |
| ME2 | malic enzyme 2, NAD(+)-dependent, mitochondrial | 210154_at | 0.86 | 47.41 |  | 0.37 | 0.60 | ↓ | 0.67 | 13.22 |  |
| DHTKD1 | dehydrogenase E1 and transketolase domain containing 1 | 209916_at | 0.86 | 40.42 |  | 2.26 | 2.80 | ↑ | 0.64 | 11.20 |  |
| LCORL | ligand dependent nuclear receptor corepressor-like | 240592_at | 0.86 | 40.42 |  | 0.97 | 60.46 |  | 0.47 | 1.61 | ↓ |
| PVR | poliovirus receptor | 214444_s_at | 0.86 | 45.69 |  | 0.13 | 0.00 | ↓ | 0.83 | 46.35 |  |
| SCAMP1 | secretory carrier membrane protein 1 | 1552978_a_at | 0.86 | 40.42 |  | 0.47 | 0.84 | ↓ | 1.37 | 26.48 |  |
| AGFG1 | ArfGAP with FG repeats 1 | 213926_s_at | 0.86 | 38.54 |  | 0.27 | 0.00 | ↓ | 1.08 | 57.92 |  |
| NDFIP2 | Nedd4 family interacting protein 2 | 224801_at | 0.86 | 45.69 |  | 0.81 | 38.78 |  | 2.01 | 1.99 | ↑ |
| ESF1 | ESF1, nucleolar pre-rRNA processing protein, homolog (S. cerevisiae) | 218859_s_at | 0.86 | 42.20 |  | 2.18 | 1.45 | ↑ | 0.98 | 58.81 |  |
| MOSPD1 | motile sperm domain containing 1 | 1557455_s_at | 0.86 | 43.91 |  | 0.40 | 0.60 | ↓ | 1.61 | 13.22 |  |
| STS | steroid sulfatase (microsomal), isozyme S | 203768_s_at | 0.86 | 34.35 |  | 0.43 | 1.07 | ↓ | 1.12 | 50.73 |  |
| DUSP1 | dual specificity phosphatase 1 | 201044_x_at | 0.86 | 48.99 |  | 0.24 | 1.07 | ↓ | 1.48 | 35.63 |  |
| RPS11 | Ribosomal protein S11 | 213350_at | 0.85 | 40.42 |  | 2.24 | 1.74 | ↑ | 1.25 | 46.35 |  |
| SSPN | sarcospan (Kras oncogene-associated gene) | 204964_s_at | 0.85 | 43.91 |  | 1.04 | 60.11 |  | 0.43 | 1.61 | ↓ |
| GBA2 | glucosidase, beta (bile acid) 2 | 224627_at | 0.85 | 40.42 |  | 0.82 | 38.78 |  | 0.39 | 1.99 | ↓ |
| SNAP29 | synaptosomal-associated protein, 29kDa | 239084_at | 0.85 | 43.91 |  | 0.46 | 1.45 | ↓ | 0.90 | 46.35 |  |
| CIZ1 | CDKN1A interacting zinc finger protein 1 | 205516_x_at | 0.85 | 38.54 |  | 0.27 | 0.00 | ↓ | 0.68 | 11.20 |  |
| EIF4G2 | eukaryotic translation initiation factor 4 gamma, 2 | 217607_x_at | 0.85 | 48.99 |  | 0.41 | 2.80 | ↓ | 1.25 | 35.63 |  |
| C1orf128 | chromosome 1 open reading frame 128 | 223123_s_at | 0.85 | 42.20 |  | 0.49 | 5.22 | ↓ | 1.25 | 48.62 |  |
| ZNF37A | zinc finger protein 37A | 228711_at | 0.85 | 34.35 |  | 2.97 | 0.29 | ↑ | 0.77 | 15.27 |  |
| CCDC136 | coiled-coil domain containing 136 | 226972_s_at | 0.85 | 43.91 |  | 0.62 | 14.26 |  | 2.12 | 3.36 | ↑ |
| WHSC1 | Wolf-Hirschhorn syndrome candidate 1 | 209053_s_at | 0.85 | 42.20 |  | 0.64 | 10.02 |  | 0.35 | 0.72 | ↓ |
| TRAM2 | translocation associated membrane protein 2 | 1554383_a_at | 0.85 | 53.22 |  | 0.10 | 0.18 | ↓ | 1.08 | 52.56 |  |
| --- | --- | 229465_s_at | 0.85 | 58.62 |  | 0.22 | 0.36 | ↓ | 0.67 | 32.58 |  |
| CHTF18 | CTF18, chromosome transmission fidelity factor 18 homolog (S. cerevisiae) | 226569_s_at | 0.85 | 36.49 |  | 0.68 | 10.02 |  | 0.38 | 0.00 | ↓ |
| IDH3G | isocitrate dehydrogenase 3 (NAD+) gamma | 202471_s_at | 0.85 | 30.22 |  | 0.47 | 0.40 | ↓ | 0.78 | 9.54 |  |
| COX15 | COX15 homolog, cytochrome c oxidase assembly protein (yeast) | 223281_s_at | 0.85 | 38.54 |  | 0.45 | 0.60 | ↓ | 1.18 | 38.27 |  |
| DCAF16 | DDB1 and CUL4 associated factor 16 | 219717_at | 0.85 | 45.69 |  | 2.26 | 2.30 | ↑ | 0.75 | 26.48 |  |
| SMARCA4 | SWI/SNF related, matrix associated, actin dependent regulator of chromatin, subfamily a, member 4 | 217656_at | 0.85 | 38.54 |  | 0.49 | 6.11 | ↓ | 0.72 | 29.62 |  |
| TLR4 | toll-like receptor 4 | 221060_s_at | 0.85 | 34.35 |  | 0.34 | 0.29 | ↓ | 1.40 | 35.63 |  |
| PTGFR | prostaglandin F receptor (FP) | 1555097_a_at | 0.85 | 47.41 |  | 0.26 | 0.84 | ↓ | 0.42 | 7.00 | ↓ |
| MAT2A | methionine adenosyltransferase II, alpha | 200769_s_at | 0.85 | 48.99 |  | 0.34 | 0.98 | ↓ | 0.89 | 50.73 |  |
| RFX7 | regulatory factor X, 7 | 218430_s_at | 0.85 | 38.54 |  | 0.57 | 2.80 |  | 2.02 | 8.02 | ↑ |
| USP53 | ubiquitin specific peptidase 53 | 230083_at | 0.85 | 45.69 |  | 1.01 | 60.64 |  | 2.95 | 1.25 | ↑ |
| CCDC8 | coiled-coil domain containing 8 | 223495_at | 0.85 | 50.55 |  | 1.65 | 14.26 |  | 0.32 | 0.72 | ↓ |
| NUCKS1 | nuclear casein kinase and cyclin-dependent kinase substrate 1 | 224582_s_at | 0.85 | 34.35 |  | 0.46 | 0.70 | ↓ | 0.92 | 38.27 |  |
| COG3 | component of oligomeric golgi complex 3 | 1554339_a_at | 0.85 | 42.20 |  | 0.17 | 0.00 | ↓ | 1.10 | 57.64 |  |
| TNFRSF9 | tumor necrosis factor receptor superfamily, member 9 | 207536_s_at | 0.85 | 58.62 |  | 0.40 | 6.11 | ↓ | 1.14 | 52.56 |  |
| MCL1 | myeloid cell leukemia sequence 1 (BCL2-related) | 200796_s_at | 0.85 | 42.20 |  | 0.15 | 0.00 | ↓ | 1.25 | 35.63 |  |
| SOX11 | SRY (sex determining region Y)-box 11 | 204914_s_at | 0.85 | 34.35 |  | 0.69 | 14.26 |  | 0.09 | 0.00 | ↓ |
| GPX3 | glutathione peroxidase 3 (plasma) | 201348_at | 0.85 | 34.35 |  | 0.30 | 0.36 | ↓ | 0.47 | 2.43 | ↓ |
| PDE11A | phosphodiesterase 11A | 237248_at | 0.85 | 43.91 |  | 0.53 | 9.01 |  | 4.61 | 2.43 | ↑ |
| TOR1AIP1 | torsin A interacting protein 1 | 216100_s_at | 0.85 | 34.35 |  | 0.50 | 0.60 | ↓ | 1.35 | 17.70 |  |
| HJURP | Holliday junction recognition protein | 218726_at | 0.85 | 34.35 |  | 0.50 | 3.81 |  | 0.14 | 0.00 | ↓ |
| HIPK2 | homeodomain interacting protein kinase 2 | 225116_at | 0.85 | 43.91 |  | 1.00 | 54.70 |  | 2.30 | 0.89 | ↑ |
| GART | phosphoribosylglycinamide formyltransferase, phosphoribosylglycinamide synthetase, phosphoribosylaminoimidazole synthetase | 212379_at | 0.85 | 32.33 |  | 0.30 | 0.00 | ↓ | 0.86 | 32.58 |  |
| MIDN | midnolin | 225954_s_at | 0.85 | 47.41 |  | 2.15 | 3.81 | ↑ | 0.72 | 26.48 |  |
| DIP2A | DIP2 disco-interacting protein 2 homolog A (Drosophila) | 1555301_a_at | 0.85 | 32.33 |  | 1.11 | 43.50 |  | 0.49 | 7.00 | ↓ |
| EZR | ezrin | 217234_s_at | 0.85 | 50.55 |  | 1.08 | 41.29 |  | 0.41 | 7.00 | ↓ |
| MGC12916 | hypothetical protein MGC12916 | 224507_s_at | 0.85 | 40.42 |  | 0.20 | 0.60 | ↓ | 1.00 | 54.08 |  |
| PSME3 | proteasome (prosome, macropain) activator subunit 3 (PA28 gamma; Ki) | 209852_x_at | 0.85 | 42.20 |  | 0.42 | 1.07 | ↓ | 1.17 | 41.22 |  |
| SORBS2 | Sorbin and SH3 domain containing 2 | 233720_at | 0.85 | 59.80 |  | 0.72 | 55.85 |  | 0.29 | 7.00 | ↓ |
| ZNF823 | zinc finger protein 823 | 229732_at | 0.85 | 36.49 |  | 1.08 | 52.07 |  | 0.34 | 0.21 | ↓ |
| PHF3 | PHD finger protein 3 | 217951_s_at | 0.85 | 40.42 |  | 3.08 | 0.53 | ↑ | 1.13 | 46.35 |  |
| PGRMC2 | progesterone receptor membrane component 2 | 201701_s_at | 0.84 | 36.49 |  | 1.01 | 57.71 |  | 2.19 | 0.43 | ↑ |
| PIK3C2A | phosphoinositide-3-kinase, class 2, alpha polypeptide | 226094_at | 0.84 | 32.33 |  | 0.44 | 0.25 | ↓ | 1.51 | 5.75 |  |
| BARD1 | BRCA1 associated RING domain 1 | 205345_at | 0.84 | 40.42 |  | 0.51 | 5.22 |  | 0.19 | 1.25 | ↓ |
| TRPC4 | transient receptor potential cation channel, subfamily C, member 4 | 224219_s_at | 0.84 | 54.53 |  | 0.57 | 38.78 |  | 2.89 | 4.16 | ↑ |
| FBXO38 | F-box protein 38 | 219608_s_at | 0.84 | 34.35 |  | 0.05 | 0.00 | ↓ | 1.30 | 32.58 |  |
| MBNL2 | muscleblind-like 2 (Drosophila) | 205018_s_at | 0.84 | 36.49 |  | 0.10 | 0.00 | ↓ | 1.20 | 52.56 |  |
| C6orf106 | chromosome 6 open reading frame 106 | 217924_at | 0.84 | 36.49 |  | 0.40 | 0.45 | ↓ | 1.24 | 26.48 |  |
| SF3B1 | splicing factor 3b, subunit 1, 155kDa | 201070_x_at | 0.84 | 26.15 |  | 0.48 | 0.18 | ↓ | 1.02 | 58.81 |  |
| TOP3B | topoisomerase (DNA) III beta | 215781_s_at | 0.84 | 47.41 |  | 0.45 | 3.81 | ↓ | 0.54 | 11.20 |  |
| GAS5 | growth arrest-specific 5 (non-protein coding) | 227517_s_at | 0.84 | 45.69 |  | 0.97 | 60.11 |  | 0.39 | 1.43 | ↓ |
| SIKE1 | suppressor of IKBKE 1 | 235294_at | 0.84 | 34.35 |  | 0.32 | 0.36 | ↓ | 0.86 | 41.22 |  |
| ELFN2 | extracellular leucine-rich repeat and fibronectin type III domain containing 2 | 1559072_a_at | 0.84 | 47.41 |  | 0.89 | 43.50 |  | 2.52 | 7.00 | ↑ |
| ANKRD28 | ankyrin repeat domain 28 | 1561079_at | 0.84 | 50.55 |  | 0.63 | 24.54 |  | 0.45 | 8.02 | ↓ |
| CHD2 | chromodomain helicase DNA binding protein 2 | 228999_at | 0.84 | 40.42 |  | 2.18 | 5.22 | ↑ | 0.70 | 17.70 |  |
| ALG10B | asparagine-linked glycosylation 10, alpha-1,2-glucosyltransferase homolog B (yeast) | 228941_at | 0.84 | 48.99 |  | 1.80 | 9.01 |  | 0.45 | 3.36 | ↓ |
| MIB1 | mindbomb homolog 1 (Drosophila) | 224726_at | 0.84 | 47.41 |  | 2.29 | 2.80 | ↑ | 1.04 | 55.52 |  |
| FOXC1 | forkhead box C1 | 213260_at | 0.84 | 50.55 |  | 0.96 | 58.42 |  | 0.47 | 5.75 | ↓ |
| AGPAT5 | 1-acylglycerol-3-phosphate O-acyltransferase 5 (lysophosphatidic acid acyltransferase, epsilon) | 218096_at | 0.84 | 47.41 |  | 1.51 | 26.77 |  | 0.45 | 4.16 | ↓ |
| UBR3 | Ubiquitin protein ligase E3 component n-recognin 3 (putative) | 244121_at | 0.84 | 34.35 |  | 1.15 | 47.13 |  | 2.07 | 5.75 | ↑ |
| ZNF711 | zinc finger protein 711 | 228988_at | 0.84 | 40.42 |  | 2.13 | 10.02 |  | 0.23 | 0.00 | ↓ |
| FANCI | Fanconi anemia, complementation group I | 213008_at | 0.84 | 38.54 |  | 0.76 | 26.77 |  | 0.23 | 0.34 | ↓ |
| BAZ1A | bromodomain adjacent to zinc finger domain, 1A | 217985_s_at | 0.84 | 32.33 |  | 1.16 | 36.20 |  | 0.42 | 0.00 | ↓ |
| RECK | reversion-inducing-cysteine-rich protein with kazal motifs | 1558116_x_at | 0.84 | 32.33 |  | 1.10 | 52.07 |  | 2.76 | 0.89 | ↑ |
| TRAM1 | translocation associated membrane protein 1 | 210733_at | 0.84 | 42.20 |  | 0.85 | 47.13 |  | 2.12 | 4.16 | ↑ |
| UBE2G2 | ubiquitin-conjugating enzyme E2G 2 (UBC7 homolog, yeast) | 1557053_s_at | 0.84 | 32.33 |  | 2.16 | 1.26 | ↑ | 0.94 | 43.83 |  |
| PCF11 | PCF11, cleavage and polyadenylation factor subunit, homolog (S. cerevisiae) | 227622_at | 0.84 | 38.54 |  | 0.61 | 7.87 |  | 0.32 | 0.00 | ↓ |
| AGGF1 | angiogenic factor with G patch and FHA domains 1 | 208042_at | 0.84 | 34.35 |  | 0.44 | 0.84 | ↓ | 0.85 | 32.58 |  |
| IQGAP3 | IQ motif containing GTPase activating protein 3 | 229538_s_at | 0.84 | 32.33 |  | 0.76 | 19.82 |  | 0.21 | 0.00 | ↓ |
| RANBP2 | RAN binding protein 2 | 201711_x_at | 0.84 | 32.33 |  | 0.36 | 0.00 | ↓ | 1.19 | 32.58 |  |
| LBR | lamin B receptor | 201795_at | 0.84 | 32.33 |  | 1.08 | 57.71 |  | 0.20 | 0.00 | ↓ |
| H2AFY2 | H2A histone family, member Y2 | 218445_at | 0.84 | 30.22 |  | 0.29 | 0.18 | ↓ | 1.19 | 57.14 |  |
| ARHGAP32 | Rho GTPase activating protein 32 | 229648_at | 0.84 | 28.36 |  | 3.82 | 0.14 | ↑ | 0.73 | 8.02 |  |
| UBXN4 | UBX domain protein 4 | 212008_at | 0.84 | 34.35 |  | 0.33 | 0.00 | ↓ | 1.13 | 48.62 |  |
| RPL21 /// RPL21P19 /// RPL21P28 | ribosomal protein L21 /// ribosomal protein L21 pseudogene 19 /// ribosomal protein L21 pseudogene 28 | 216479_at | 0.83 | 30.22 |  | 0.24 | 0.98 | ↓ | 0.68 | 13.22 |  |
| PTPN11 | protein tyrosine phosphatase, non-receptor type 11 | 209896_s_at | 0.83 | 30.22 |  | 0.45 | 0.45 | ↓ | 1.07 | 55.52 |  |
| KCNAB1 | potassium voltage-gated channel, shaker-related subfamily, beta member 1 | 208213_s_at | 0.83 | 36.49 |  | 0.10 | 0.00 | ↓ | 1.10 | 58.81 |  |
| TK1 | thymidine kinase 1, soluble | 1554408_a_at | 0.83 | 32.33 |  | 0.45 | 1.07 | ↓ | 0.14 | 0.00 | ↓ |
| CDC5L | CDC5 cell division cycle 5-like (S. pombe) | 209055_s_at | 0.83 | 30.22 |  | 0.41 | 0.00 | ↓ | 0.89 | 32.58 |  |
| ANAPC5 | anaphase promoting complex subunit 5 | 208721_s_at | 0.83 | 38.54 |  | 0.19 | 0.00 | ↓ | 1.03 | 58.28 |  |
| C11orf80 | chromosome 11 open reading frame 80 | 238593_at | 0.83 | 54.53 |  | 1.28 | 49.04 |  | 0.44 | 8.02 | ↓ |
| PDE1A | phosphodiesterase 1A, calmodulin-dependent | 1558680_s_at | 0.83 | 60.72 |  | 0.39 | 19.82 |  | 3.31 | 8.02 | ↑ |
| ELL2 | elongation factor, RNA polymerase II, 2 | 226982_at | 0.83 | 54.53 |  | 0.86 | 59.12 |  | 2.40 | 1.43 | ↑ |
| SNAP23 | synaptosomal-associated protein, 23kDa | 214544_s_at | 0.83 | 38.54 |  | 0.38 | 0.45 | ↓ | 1.45 | 20.38 |  |
| ARFGEF1 | ADP-ribosylation factor guanine nucleotide-exchange factor 1(brefeldin A-inhibited) | 216266_s_at | 0.83 | 30.22 |  | 0.47 | 0.53 | ↓ | 1.05 | 55.52 |  |
| UBE2L3 | ubiquitin-conjugating enzyme E2L 3 | 200684_s_at | 0.83 | 30.22 |  | 0.46 | 0.53 | ↓ | 1.03 | 58.68 |  |
| C10orf28 | chromosome 10 open reading frame 28 | 210455_at | 0.83 | 34.35 |  | 0.36 | 0.40 | ↓ | 1.09 | 57.14 |  |
| PPP4R2 | protein phosphatase 4, regulatory subunit 2 | 220764_at | 0.83 | 47.41 |  | 0.47 | 6.80 | ↓ | 1.01 | 58.81 |  |
| --- | --- | 215447_at | 0.83 | 43.91 |  | 0.57 | 10.02 |  | 0.47 | 4.16 | ↓ |
| RAP2A /// RAP2B | RAP2A, member of RAS oncogene family /// RAP2B, member of RAS oncogene family | 214487_s_at | 0.83 | 40.42 |  | 0.31 | 0.29 | ↓ | 0.72 | 17.70 |  |
| TSN | translin | 201504_s_at | 0.83 | 36.49 |  | 0.48 | 1.74 | ↓ | 1.12 | 52.56 |  |
| ADORA1 | adenosine A1 receptor | 205481_at | 0.83 | 38.54 |  | 0.49 | 4.45 | ↓ | 1.40 | 54.08 |  |
| ZDHHC20 | zinc finger, DHHC-type containing 20 | 243786_at | 0.83 | 38.54 |  | 0.39 | 0.98 | ↓ | 0.97 | 50.73 |  |
| POLK | polymerase (DNA directed) kappa | 223260_s_at | 0.83 | 30.22 |  | 0.49 | 0.53 | ↓ | 1.28 | 17.70 |  |
| --- | --- | 243796_at | 0.83 | 38.54 |  | 0.56 | 12.66 |  | 0.32 | 2.43 | ↓ |
| IPO8 | importin 8 | 205701_at | 0.83 | 32.33 |  | 0.34 | 0.25 | ↓ | 1.04 | 58.81 |  |
| BCR | breakpoint cluster region | 226602_s_at | 0.83 | 30.22 |  | 0.44 | 0.40 | ↓ | 1.15 | 50.73 |  |
| GPR161 | G protein-coupled receptor 161 | 206971_at | 0.83 | 34.35 |  | 0.49 | 1.07 | ↓ | 0.66 | 15.27 |  |
| TUBB2C | tubulin, beta 2C | 208977_x_at | 0.83 | 38.54 |  | 0.76 | 26.77 |  | 0.44 | 0.72 | ↓ |
| ALKBH1 | alkB, alkylation repair homolog 1 (E. coli) | 205621_at | 0.83 | 30.22 |  | 0.48 | 0.98 | ↓ | 1.13 | 48.62 |  |
| PKN2 | protein kinase N2 | 210969_at | 0.83 | 40.42 |  | 0.43 | 1.74 | ↓ | 0.84 | 38.27 |  |
| FEN1 | flap structure-specific endonuclease 1 | 204768_s_at | 0.83 | 36.49 |  | 0.56 | 6.11 |  | 0.42 | 0.34 | ↓ |
| SHMT1 | serine hydroxymethyltransferase 1 (soluble) | 224954_at | 0.83 | 40.42 |  | 1.16 | 49.04 |  | 0.44 | 4.16 | ↓ |
| TUBB2C | tubulin, beta 2C | 213726_x_at | 0.83 | 34.35 |  | 0.76 | 24.54 |  | 0.48 | 1.02 | ↓ |
| CDK2 | cyclin-dependent kinase 2 | 211804_s_at | 0.83 | 38.54 |  | 0.37 | 0.60 | ↓ | 0.52 | 2.70 |  |
| FZD8 | frizzled homolog 8 (Drosophila) | 224325_at | 0.83 | 32.33 |  | 2.81 | 1.74 | ↑ | 0.45 | 0.34 | ↓ |
| NEXN | nexilin (F actin binding protein) | 1552309_a_at | 0.83 | 34.35 |  | 2.08 | 2.30 | ↑ | 1.80 | 5.75 |  |
| ZNF638 | zinc finger protein 638 | 1554249_a_at | 0.83 | 42.20 |  | 0.45 | 3.35 | ↓ | 1.10 | 54.85 |  |
| CCDC6 | coiled-coil domain containing 6 | 204716_at | 0.82 | 34.35 |  | 0.35 | 0.18 | ↓ | 1.07 | 58.81 |  |
| HS2ST1 | heparan sulfate 2-O-sulfotransferase 1 | 203285_s_at | 0.82 | 40.42 |  | 0.85 | 47.13 |  | 2.12 | 8.02 | ↑ |
| RMI1 | RMI1, RecQ mediated genome instability 1, homolog (S. cerevisiae) | 218979_at | 0.82 | 28.36 |  | 1.14 | 36.20 |  | 0.49 | 0.21 | ↓ |
| INO80C | INO80 complex subunit C | 229582_at | 0.82 | 45.69 |  | 1.26 | 29.01 |  | 0.40 | 1.61 | ↓ |
| ABHD2 | abhydrolase domain containing 2 | 87100_at | 0.82 | 30.22 |  | 0.16 | 0.00 | ↓ | 1.41 | 13.22 |  |
| APLP2 | amyloid beta (A4) precursor-like protein 2 | 214875_x_at | 0.82 | 30.22 |  | 0.49 | 0.84 | ↓ | 0.78 | 15.27 |  |
| RUFY3 | RUN and FYVE domain containing 3 | 229334_at | 0.82 | 45.69 |  | 0.29 | 0.36 | ↓ | 0.81 | 38.27 |  |
| --- | --- | 239336_at | 0.82 | 42.20 |  | 1.05 | 50.64 |  | 2.17 | 4.16 | ↑ |
| SPTBN1 | spectrin, beta, non-erythrocytic 1 | 215918_s_at | 0.82 | 53.22 |  | 0.23 | 0.70 | ↓ | 0.84 | 52.56 |  |
| EXO1 | exonuclease 1 | 204603_at | 0.82 | 38.54 |  | 0.66 | 14.26 |  | 0.05 | 0.00 | ↓ |
| TGFB2 | transforming growth factor, beta 2 | 220407_s_at | 0.82 | 50.55 |  | 0.50 | 7.87 | ↓ | 0.31 | 1.02 | ↓ |
| AP1S1 | adaptor-related protein complex 1, sigma 1 subunit | 209635_at | 0.82 | 38.54 |  | 2.42 | 1.74 | ↑ | 1.47 | 17.70 |  |
| ARFIP1 | ADP-ribosylation factor interacting protein 1 | 214483_s_at | 0.82 | 36.49 |  | 0.34 | 0.25 | ↓ | 1.31 | 29.62 |  |
| TMPO | thymopoietin | 209754_s_at | 0.82 | 38.54 |  | 0.21 | 0.00 | ↓ | 0.25 | 0.00 | ↓ |
| PDLIM5 | PDZ and LIM domain 5 | 211681_s_at | 0.82 | 48.99 |  | 0.19 | 0.25 | ↓ | 0.97 | 56.10 |  |
| STK11IP | serine/threonine kinase 11 interacting protein | 225713_at | 0.82 | 40.42 |  | 0.61 | 11.08 |  | 0.48 | 5.75 | ↓ |
| ITGB5 | Integrin, beta 5 | 214020_x_at | 0.82 | 45.69 |  | 0.83 | 52.07 |  | 3.18 | 1.99 | ↑ |
| SLC7A11 | solute carrier family 7, (cationic amino acid transporter, y+ system) member 11 | 209921_at | 0.82 | 45.69 |  | 2.43 | 4.45 | ↑ | 0.70 | 32.58 |  |
| EIF3M | eukaryotic translation initiation factor 3, subunit M | 215190_at | 0.82 | 32.33 |  | 2.12 | 6.11 | ↑ | 1.20 | 41.22 |  |
| ANKFY1 | ankyrin repeat and FYVE domain containing 1 | 219868_s_at | 0.82 | 28.36 |  | 0.20 | 0.00 | ↓ | 1.00 | 58.81 |  |
| USP34 | ubiquitin specific peptidase 34 | 212065_s_at | 0.82 | 32.33 |  | 0.31 | 0.18 | ↓ | 1.42 | 17.70 |  |
| STK38 | serine/threonine kinase 38 | 1553117_a_at | 0.82 | 32.33 |  | 0.44 | 1.07 | ↓ | 1.27 | 23.22 |  |
| GIMAP2 | GTPase, IMAP family member 2 | 232024_at | 0.82 | 53.22 |  | 0.75 | 43.50 |  | 0.19 | 1.43 | ↓ |
| PARP2 | poly (ADP-ribose) polymerase 2 | 214086_s_at | 0.82 | 24.35 |  | 0.80 | 19.82 |  | 0.47 | 0.34 | ↓ |
| STIP1 | stress-induced-phosphoprotein 1 | 213330_s_at | 0.82 | 26.15 |  | 0.35 | 0.18 | ↓ | 0.66 | 3.36 |  |
| PIK3C2A | phosphoinositide-3-kinase, class 2, alpha polypeptide | 1553694_a_at | 0.82 | 30.22 |  | 0.20 | 0.00 | ↓ | 1.47 | 9.54 |  |
| GALNTL2 | UDP-N-acetyl-alpha-D-galactosamine:polypeptide N-acetylgalactosaminyltransferase-like 2 | 228501_at | 0.82 | 54.53 |  | 1.00 | 43.50 |  | 0.44 | 8.02 | ↓ |
| EXOC3 | exocyst complex component 3 | 212630_at | 0.82 | 32.33 |  | 0.48 | 1.74 | ↓ | 1.31 | 17.70 |  |
| ANKRD6 | ankyrin repeat domain 6 | 204671_s_at | 0.82 | 45.69 |  | 1.58 | 19.82 |  | 2.70 | 2.43 | ↑ |
| TWF1 | twinfilin, actin-binding protein, homolog 1 (Drosophila) | 214007_s_at | 0.82 | 32.33 |  | 0.14 | 0.00 | ↓ | 1.11 | 54.08 |  |
| CENPH | centromere protein H | 231772_x_at | 0.82 | 24.35 |  | 0.65 | 6.80 |  | 0.29 | 0.00 | ↓ |
| KCTD20 | potassium channel tetramerisation domain containing 20 | 228299_at | 0.82 | 34.35 |  | 1.28 | 29.01 |  | 2.09 | 1.25 | ↑ |
| CUL4B | cullin 4B | 215997_s_at | 0.81 | 40.42 |  | 0.43 | 1.98 | ↓ | 0.94 | 54.08 |  |
| TRAK2 | trafficking protein, kinesin binding 2 | 202124_s_at | 0.81 | 34.35 |  | 0.43 | 1.07 | ↓ | 1.83 | 4.16 |  |
| CXCL12 | chemokine (C-X-C motif) ligand 12 | 209687_at | 0.81 | 26.15 |  | 0.95 | 54.70 |  | 0.42 | 0.34 | ↓ |
| SGSH | N-sulfoglucosamine sulfohydrolase | 204293_at | 0.81 | 26.15 |  | 0.43 | 1.74 | ↓ | 1.11 | 55.52 |  |
| USP34 | ubiquitin specific peptidase 34 | 242647_at | 0.81 | 36.49 |  | 0.18 | 0.00 | ↓ | 1.29 | 35.63 |  |
| CEP120 | centrosomal protein 120kDa | 1554606_at | 0.81 | 28.36 |  | 0.48 | 1.07 | ↓ | 0.87 | 32.58 |  |
| PSG4 | pregnancy specific beta-1-glycoprotein 4 | 208191_x_at | 0.81 | 45.69 |  | 0.41 | 19.82 |  | 7.72 | 9.54 | ↑ |
| LOC440983 | Hypothetical gene supported by BC066916 | 227338_at | 0.81 | 34.35 |  | 0.35 | 0.29 | ↓ | 0.81 | 29.62 |  |
| NSUN4 | NOP2/Sun domain family, member 4 | 1559848_at | 0.81 | 47.41 |  | 0.75 | 43.50 |  | 0.33 | 4.16 | ↓ |
| GOPC | Golgi-associated PDZ and coiled-coil motif containing | 236862_at | 0.81 | 30.22 |  | 2.00 | 9.01 | ↑ | 0.95 | 46.35 |  |
| AP1G1 | adaptor-related protein complex 1, gamma 1 subunit | 225754_at | 0.81 | 34.35 |  | 0.44 | 0.98 | ↓ | 1.09 | 55.52 |  |
| EHD1 | EH-domain containing 1 | 209038_s_at | 0.81 | 34.35 |  | 0.36 | 0.45 | ↓ | 0.71 | 17.70 |  |
| TNFSF13B | tumor necrosis factor (ligand) superfamily, member 13b | 223502_s_at | 0.81 | 47.41 |  | 0.56 | 14.26 |  | 0.45 | 5.75 | ↓ |
| SH3BGRL2 | SH3 domain binding glutamic acid-rich protein like 2 | 225354_s_at | 0.81 | 36.49 |  | 0.43 | 0.98 | ↓ | 1.31 | 35.63 |  |
| SNAP29 | synaptosomal-associated protein, 29kDa | 222597_at | 0.81 | 36.49 |  | 0.48 | 1.98 | ↓ | 0.95 | 52.56 |  |
| ODZ4 | odz, odd Oz/ten-m homolog 4 (Drosophila) | 213273_at | 0.81 | 28.36 |  | 1.28 | 47.13 |  | 0.49 | 7.00 | ↓ |
| PRR15 | proline rich 15 | 226961_at | 0.81 | 51.95 |  | 0.60 | 38.78 |  | 2.44 | 5.75 | ↑ |
| SLC24A1 | solute carrier family 24 (sodium/potassium/calcium exchanger), member 1 | 206081_at | 0.81 | 30.22 |  | 2.06 | 1.45 | ↑ | 1.04 | 58.81 |  |
| B9D1 | B9 protein domain 1 | 210534_s_at | 0.81 | 28.36 |  | 0.93 | 47.13 |  | 0.50 | 0.34 | ↓ |
| THBS1 | thrombospondin 1 | 235086_at | 0.81 | 43.91 |  | 2.44 | 4.45 | ↑ | 1.87 | 5.75 |  |
| FAM168A | family with sequence similarity 168, member A | 212357_at | 0.81 | 26.15 |  | 0.46 | 0.45 | ↓ | 0.99 | 54.08 |  |
| C13orf31 | chromosome 13 open reading frame 31 | 1553141_at | 0.81 | 32.33 |  | 0.29 | 0.25 | ↓ | 3.25 | 1.61 | ↑ |
| PERP | PERP, TP53 apoptosis effector | 222392_x_at | 0.81 | 28.36 |  | 1.31 | 29.01 |  | 2.21 | 1.61 | ↑ |
| SUPT16H | suppressor of Ty 16 homolog (S. cerevisiae) | 233827_s_at | 0.81 | 32.33 |  | 0.15 | 0.00 | ↓ | 0.92 | 48.62 |  |
| MEG3 | maternally expressed 3 (non-protein coding) | 235077_at | 0.81 | 43.91 |  | 2.28 | 3.35 | ↑ | 1.07 | 58.81 |  |
| BOP1 | block of proliferation 1 | 216397_s_at | 0.81 | 36.49 |  | 0.45 | 1.07 | ↓ | 0.83 | 35.63 |  |
| ALDH1L2 | aldehyde dehydrogenase 1 family, member L2 | 231202_at | 0.81 | 38.54 |  | 2.05 | 3.81 | ↑ | 0.76 | 26.48 |  |
| LOC375196 | hypothetical LOC375196 | 1556244_s_at | 0.81 | 28.36 |  | 0.93 | 45.34 |  | 0.46 | 5.75 | ↓ |
| CDKN2AIPNL | CDKN2A interacting protein N-terminal like | 235006_at | 0.81 | 32.33 |  | 0.50 | 1.98 | ↓ | 0.88 | 38.27 |  |
| CLEC11A | C-type lectin domain family 11, member A | 211709_s_at | 0.81 | 28.36 |  | 1.15 | 43.50 |  | 0.44 | 0.43 | ↓ |
| CDV3 | CDV3 homolog (mouse) | 213548_s_at | 0.81 | 36.49 |  | 0.12 | 0.00 | ↓ | 1.25 | 35.63 |  |
| PRIM2 | primase, DNA, polypeptide 2 (58kDa) | 215708_s_at | 0.80 | 30.22 |  | 0.30 | 0.25 | ↓ | 1.13 | 54.85 |  |
| HN1 | hematological and neurological expressed 1 | 217755_at | 0.80 | 30.22 |  | 0.78 | 26.77 |  | 0.43 | 0.21 | ↓ |
| CANX | calnexin | 238034_at | 0.80 | 26.15 |  | 1.25 | 38.78 |  | 2.16 | 0.31 | ↑ |
| PAQR4 | progestin and adipoQ receptor family member IV | 212858_at | 0.80 | 28.36 |  | 0.81 | 29.01 |  | 0.35 | 1.43 | ↓ |
| PIK3R1 | phosphoinositide-3-kinase, regulatory subunit 1 (alpha) | 212249_at | 0.80 | 58.62 |  | 0.39 | 10.02 |  | 6.34 | 0.72 | ↑ |
| CFI | complement factor I | 203854_at | 0.80 | 40.42 |  | 1.24 | 33.65 |  | 0.26 | 3.36 | ↓ |
| GHRL | ghrelin/obestatin prepropeptide | 223862_at | 0.80 | 22.56 |  | 0.32 | 0.76 | ↓ | 1.07 | 58.81 |  |
| TLR4 | toll-like receptor 4 | 1552798_a_at | 0.80 | 28.36 |  | 0.40 | 0.25 | ↓ | 1.04 | 54.08 |  |
| ETV1 | ets variant 1 | 221910_at | 0.80 | 34.35 |  | 0.38 | 2.30 | ↓ | 0.85 | 38.27 |  |
| SALL1 | sal-like 1 (Drosophila) | 229273_at | 0.80 | 34.35 |  | 1.05 | 45.34 |  | 0.34 | 3.36 | ↓ |
| ERGIC1 | endoplasmic reticulum-golgi intermediate compartment (ERGIC) 1 | 224576_at | 0.80 | 28.36 |  | 2.99 | 0.21 | ↑ | 1.20 | 46.35 |  |
| ITGB8 | integrin, beta 8 | 205816_at | 0.80 | 43.91 |  | 0.74 | 43.50 |  | 3.31 | 3.36 | ↑ |
| NR1D2 | nuclear receptor subfamily 1, group D, member 2 | 209750_at | 0.80 | 40.42 |  | 0.27 | 0.40 | ↓ | 1.13 | 54.85 |  |
| TCF25 | transcription factor 25 (basic helix-loop-helix) | 213311_s_at | 0.80 | 28.36 |  | 2.05 | 1.45 | ↑ | 1.06 | 54.85 |  |
| MPHOSPH9 | M-phase phosphoprotein 9 | 237158_s_at | 0.80 | 36.49 |  | 0.24 | 0.25 | ↓ | 0.76 | 23.22 |  |
| DNAJC3 | DnaJ (Hsp40) homolog, subfamily C, member 3 | 208499_s_at | 0.80 | 28.36 |  | 0.77 | 24.54 |  | 2.13 | 4.16 | ↑ |
| EXOG | endo/exonuclease (5'-3'), endonuclease G-like | 206902_s_at | 0.80 | 30.22 |  | 0.41 | 0.76 | ↓ | 1.18 | 52.56 |  |
| STMN3 | stathmin-like 3 | 222557_at | 0.80 | 40.42 |  | 1.16 | 49.04 |  | 0.38 | 2.43 | ↓ |
| TIMELESS | timeless homolog (Drosophila) | 203046_s_at | 0.80 | 43.91 |  | 0.91 | 55.85 |  | 0.36 | 1.02 | ↓ |
| MLF1IP | MLF1 interacting protein | 229305_at | 0.80 | 34.35 |  | 0.70 | 22.17 |  | 0.46 | 2.43 | ↓ |
| SEC23A | Sec23 homolog A (S. cerevisiae) | 204344_s_at | 0.80 | 32.33 |  | 0.41 | 0.60 | ↓ | 0.90 | 43.83 |  |
| PRPF39 | PRP39 pre-mRNA processing factor 39 homolog (S. cerevisiae) | 220553_s_at | 0.80 | 36.49 |  | 2.25 | 1.74 | ↑ | 0.91 | 48.62 |  |
| ACLY | ATP citrate lyase | 210337_s_at | 0.80 | 34.35 |  | 0.46 | 1.26 | ↓ | 0.75 | 20.38 |  |
| HTR7 | 5-hydroxytryptamine (serotonin) receptor 7 (adenylate cyclase-coupled) | 236281_x_at | 0.80 | 28.36 |  | 1.35 | 29.01 |  | 0.48 | 5.75 | ↓ |
| AURKB | aurora kinase B | 209464_at | 0.80 | 26.15 |  | 0.48 | 1.74 | ↓ | 0.11 | 0.00 | ↓ |
| ATP11C | ATPase, class VI, type 11C | 242690_at | 0.80 | 45.69 |  | 0.40 | 1.98 | ↓ | 1.41 | 23.22 |  |
| MAPK1 | mitogen-activated protein kinase 1 | 1552264_a_at | 0.80 | 38.54 |  | 0.35 | 0.53 | ↓ | 0.92 | 52.56 |  |
| DKFZP434I0714 | hypothetical protein DKFZP434I0714 | 231954_at | 0.80 | 34.35 |  | 1.24 | 41.29 |  | 0.42 | 3.36 | ↓ |
| TCF3 | transcription factor 3 (E2A immunoglobulin enhancer binding factors E12/E47) | 209153_s_at | 0.80 | 24.35 |  | 0.70 | 9.01 |  | 0.41 | 0.00 | ↓ |
| TACC3 | transforming, acidic coiled-coil containing protein 3 | 218308_at | 0.80 | 28.36 |  | 0.53 | 3.35 |  | 0.09 | 0.00 | ↓ |
| NCAM1 | neural cell adhesion molecule 1 | 212843_at | 0.80 | 55.60 |  | 0.51 | 50.64 |  | 0.17 | 7.00 | ↓ |
| ADPRH | ADP-ribosylarginine hydrolase | 228042_at | 0.80 | 30.22 |  | 1.20 | 41.29 |  | 0.49 | 0.58 | ↓ |
| CDC42BPA | CDC42 binding protein kinase alpha (DMPK-like) | 214464_at | 0.80 | 40.42 |  | 4.65 | 0.14 | ↑ | 1.01 | 57.64 |  |
| GUCY1B3 | guanylate cyclase 1, soluble, beta 3 | 203817_at | 0.80 | 47.41 |  | 0.65 | 41.29 |  | 0.36 | 5.75 | ↓ |
| CASP8 | caspase 8, apoptosis-related cysteine peptidase | 207686_s_at | 0.80 | 28.36 |  | 0.42 | 0.70 | ↓ | 1.05 | 58.81 |  |
| KIF18A | kinesin family member 18A | 221258_s_at | 0.80 | 26.15 |  | 0.85 | 31.53 |  | 0.39 | 1.61 | ↓ |
| GOLGA6A | golgin A6 family, member A | 207575_at | 0.80 | 28.36 |  | 0.38 | 0.84 | ↓ | 0.71 | 15.27 |  |
| MLLT10 | myeloid/lymphoid or mixed-lineage leukemia (trithorax homolog, Drosophila); translocated to, 10 | 205408_at | 0.80 | 30.22 |  | 0.39 | 0.53 | ↓ | 0.77 | 20.38 |  |
| SCAMP1 | secretory carrier membrane protein 1 | 206667_s_at | 0.80 | 34.35 |  | 0.40 | 1.07 | ↓ | 1.74 | 20.38 |  |
| ST6GALNAC5 | ST6 (alpha-N-acetyl-neuraminyl-2,3-beta-galactosyl-1,3)-N-acetylgalactosaminide alpha-2,6-sialyltransferase 5 | 220979_s_at | 0.79 | 58.62 |  | 0.62 | 43.50 |  | 3.13 | 9.54 | ↑ |
| PTPN11 | protein tyrosine phosphatase, non-receptor type 11 | 209895_at | 0.79 | 32.33 |  | 0.24 | 0.18 | ↓ | 1.13 | 50.73 |  |
| SIX2 | SIX homeobox 2 | 206511_s_at | 0.79 | 34.35 |  | 0.64 | 12.66 |  | 0.43 | 1.61 | ↓ |
| ESR1 | estrogen receptor 1 | 205225_at | 0.79 | 32.33 |  | 0.71 | 17.74 |  | 0.42 | 0.50 | ↓ |
| TBCEL | tubulin folding cofactor E-like | 231997_at | 0.79 | 34.35 |  | 0.49 | 3.35 | ↓ | 1.44 | 35.63 |  |
| PTN | pleiotrophin | 211737_x_at | 0.79 | 32.33 |  | 1.62 | 17.74 |  | 0.11 | 0.00 | ↓ |
| YWHAB | tyrosine 3-monooxygenase/tryptophan 5-monooxygenase activation protein, beta polypeptide | 217717_s_at | 0.79 | 26.15 |  | 2.02 | 1.74 | ↑ | 0.92 | 41.22 |  |
| --- | --- | 243711_at | 0.79 | 28.36 |  | 1.28 | 33.65 |  | 2.23 | 2.70 | ↑ |
| RHOJ | ras homolog gene family, member J | 235489_at | 0.79 | 28.36 |  | 1.42 | 55.85 |  | 2.41 | 4.84 | ↑ |
| TDP1 | tyrosyl-DNA phosphodiesterase 1 | 219715_s_at | 0.79 | 22.56 |  | 0.40 | 0.18 | ↓ | 0.64 | 4.16 |  |
| RNF213 | ring finger protein 213 | 225931_s_at | 0.79 | 30.22 |  | 0.41 | 0.76 | ↓ | 0.74 | 15.27 |  |
| MIR155HG | MIR155 host gene (non-protein coding) | 229437_at | 0.79 | 47.41 |  | 1.19 | 31.53 |  | 0.46 | 9.54 | ↓ |
| SCRG1 | stimulator of chondrogenesis 1 | 205475_at | 0.79 | 61.53 |  | 1.93 | 43.50 |  | 0.05 | 0.21 | ↓ |
| WDR26 | WD repeat domain 26 | 224898_at | 0.79 | 24.35 |  | 0.39 | 0.18 | ↓ | 1.63 | 5.75 |  |
| PTGR1 | prostaglandin reductase 1 | 228824_s_at | 0.79 | 24.35 |  | 0.31 | 0.00 | ↓ | 0.70 | 8.02 |  |
| RBP1 | retinol binding protein 1, cellular | 203423_at | 0.79 | 43.91 |  | 0.98 | 43.50 |  | 0.38 | 7.00 | ↓ |
| --- | --- | 231697_s_at | 0.79 | 32.33 |  | 1.50 | 19.82 |  | 0.32 | 0.00 | ↓ |
| BMS1P5 | BMS1 pseudogene 5 | 231106_at | 0.79 | 34.35 |  | 0.48 | 2.30 | ↓ | 1.05 | 58.28 |  |
| UHMK1 | U2AF homology motif (UHM) kinase 1 | 227740_at | 0.79 | 43.91 |  | 0.37 | 1.45 | ↓ | 1.58 | 17.70 |  |
| ZFR | zinc finger RNA binding protein | 33148_at | 0.79 | 26.15 |  | 0.33 | 0.00 | ↓ | 1.20 | 20.38 |  |
| RG9MTD2 | RNA (guanine-9-) methyltransferase domain containing 2 | 242442_x_at | 0.79 | 28.36 |  | 2.73 | 1.98 | ↑ | 1.31 | 35.63 |  |
| OSBPL8 | oxysterol binding protein-like 8 | 212582_at | 0.79 | 26.15 |  | 0.65 | 7.87 |  | 2.29 | 1.02 | ↑ |
| CDCP1 | CUB domain containing protein 1 | 234932_s_at | 0.79 | 58.62 |  | 0.12 | 0.29 | ↓ | 1.03 | 52.56 |  |
| FNIP1 /// RAPGEF6 | folliculin interacting protein 1 /// Rap guanine nucleotide exchange factor (GEF) 6 | 219112_at | 0.79 | 30.22 |  | 2.82 | 0.40 | ↑ | 1.11 | 54.08 |  |
| DPP4 | dipeptidyl-peptidase 4 | 211478_s_at | 0.79 | 45.69 |  | 0.44 | 7.87 | ↓ | 7.55 | 0.19 | ↑ |
| CRABP2 | cellular retinoic acid binding protein 2 | 202575_at | 0.79 | 47.41 |  | 0.40 | 3.81 | ↓ | 0.55 | 13.22 |  |
| GFM1 | G elongation factor, mitochondrial 1 | 232296_s_at | 0.79 | 36.49 |  | 0.44 | 5.22 | ↓ | 0.83 | 38.27 |  |
| JUN | Jun oncogene | 213281_at | 0.79 | 34.35 |  | 2.47 | 5.22 | ↑ | 1.54 | 11.20 |  |
| COPG2IT1 | COPG2 imprinted transcript 1 (non-protein coding) | 213486_at | 0.79 | 28.36 |  | 0.82 | 38.78 |  | 2.11 | 7.00 | ↑ |
| RNF213 | ring finger protein 213 | 231956_at | 0.79 | 36.49 |  | 0.65 | 15.95 |  | 0.49 | 1.99 | ↓ |
| NUDT13 | nudix (nucleoside diphosphate linked moiety X)-type motif 13 | 214136_at | 0.78 | 34.35 |  | 0.94 | 60.11 |  | 0.43 | 2.70 | ↓ |
| EIF5A2 | eukaryotic translation initiation factor 5A2 | 235296_at | 0.78 | 22.56 |  | 1.02 | 59.66 |  | 2.42 | 0.43 | ↑ |
| FBLN1 | fibulin 1 | 202995_s_at | 0.78 | 55.60 |  | 0.44 | 9.01 | ↓ | 0.61 | 17.70 |  |
| EPSTI1 | epithelial stromal interaction 1 (breast) | 235276_at | 0.78 | 48.99 |  | 0.49 | 12.66 |  | 0.36 | 4.84 | ↓ |
| LONP2 | Lon peptidase 2, peroxisomal | 221834_at | 0.78 | 26.15 |  | 0.48 | 0.98 | ↓ | 0.89 | 38.27 |  |
| ENSA | endosulfine alpha | 221487_s_at | 0.78 | 36.49 |  | 0.35 | 1.07 | ↓ | 1.13 | 50.73 |  |
| HSD17B6 | hydroxysteroid (17-beta) dehydrogenase 6 homolog (mouse) | 37512_at | 0.78 | 42.20 |  | 2.10 | 10.02 |  | 0.27 | 1.99 | ↓ |
| PPTC7 | PTC7 protein phosphatase homolog (S. cerevisiae) | 235744_at | 0.78 | 28.36 |  | 0.43 | 0.76 | ↓ | 1.22 | 48.62 |  |
| BRE | brain and reproductive organ-expressed (TNFRSF1A modulator) | 211566_x_at | 0.78 | 18.98 |  | 0.50 | 0.29 | ↓ | 1.74 | 4.84 |  |
| ALDH6A1 | aldehyde dehydrogenase 6 family, member A1 | 204290_s_at | 0.78 | 24.35 |  | 0.30 | 0.00 | ↓ | 0.74 | 13.22 |  |
| GALNT1 | UDP-N-acetyl-alpha-D-galactosamine:polypeptide N-acetylgalactosaminyltransferase 1 (GalNAc-T1) | 1568618_a_at | 0.78 | 30.22 |  | 1.22 | 31.53 |  | 0.34 | 0.21 | ↓ |
| CCNA2 | cyclin A2 | 203418_at | 0.78 | 40.42 |  | 0.48 | 6.11 | ↓ | 0.15 | 0.00 | ↓ |
| PRPS1 | phosphoribosyl pyrophosphate synthetase 1 | 208447_s_at | 0.78 | 28.36 |  | 0.66 | 14.26 |  | 2.34 | 1.99 | ↑ |
| DST | dystonin | 212253_x_at | 0.78 | 28.36 |  | 0.46 | 1.45 | ↓ | 1.07 | 57.14 |  |
| MED13L | mediator complex subunit 13-like | 212207_at | 0.78 | 30.22 |  | 3.26 | 0.40 | ↑ | 0.78 | 26.48 |  |
| OSBPL8 | oxysterol binding protein-like 8 | 212585_at | 0.78 | 24.35 |  | 1.00 | 59.12 |  | 2.51 | 0.89 | ↑ |
| PSG6 | pregnancy specific beta-1-glycoprotein 6 | 208106_x_at | 0.78 | 38.54 |  | 0.53 | 11.08 |  | 7.12 | 0.50 | ↑ |
| VSIG10 | V-set and immunoglobulin domain containing 10 | 226485_at | 0.78 | 28.36 |  | 2.78 | 0.45 | ↑ | 0.69 | 11.20 |  |
| ZBTB38 | zinc finger and BTB domain containing 38 | 1558733_at | 0.78 | 26.15 |  | 0.35 | 0.25 | ↓ | 1.74 | 4.16 |  |
| MUC1 | mucin 1, cell surface associated | 213693_s_at | 0.78 | 30.22 |  | 1.81 | 15.95 |  | 2.56 | 0.58 | ↑ |
| SORBS2 | sorbin and SH3 domain containing 2 | 220858_at | 0.78 | 61.96 |  | 0.55 | 38.78 |  | 0.32 | 9.54 | ↓ |
| TARS2 | threonyl-tRNA synthetase 2, mitochondrial (putative) | 221189_s_at | 0.78 | 22.56 |  | 0.39 | 1.26 | ↓ | 0.84 | 13.22 |  |
| NARG2 | NMDA receptor regulated 2 | 235189_at | 0.78 | 28.36 |  | 0.35 | 0.29 | ↓ | 0.87 | 38.27 |  |
| DBNL | drebrin-like | 222429_at | 0.78 | 28.36 |  | 0.50 | 1.45 | ↓ | 0.75 | 17.70 |  |
| ZNF644 | zinc finger protein 644 | 1553725_s_at | 0.78 | 38.54 |  | 0.30 | 0.60 | ↓ | 0.72 | 26.48 |  |
| C5orf13 | chromosome 5 open reading frame 13 | 201309_x_at | 0.78 | 28.36 |  | 0.47 | 0.98 | ↓ | 1.36 | 41.22 |  |
| NSD1 | nuclear receptor binding SET domain protein 1 | 219084_at | 0.78 | 20.86 |  | 0.42 | 0.84 | ↓ | 0.95 | 43.83 |  |
| ARID2 | AT rich interactive domain 2 (ARID, RFX-like) | 231090_s_at | 0.78 | 34.35 |  | 0.23 | 0.00 | ↓ | 0.60 | 11.20 |  |
| BTN3A2 | butyrophilin, subfamily 3, member A2 | 209846_s_at | 0.78 | 26.15 |  | 0.49 | 1.98 | ↓ | 0.50 | 0.89 | ↓ |
| USP10 | ubiquitin specific peptidase 10 | 209136_s_at | 0.78 | 28.36 |  | 0.35 | 0.18 | ↓ | 1.18 | 46.35 |  |
| ACBD3 | acyl-CoA binding domain containing 3 | 202323_s_at | 0.78 | 20.86 |  | 0.44 | 0.60 | ↓ | 0.90 | 35.63 |  |
| TRIM59 | tripartite motif-containing 59 | 235476_at | 0.78 | 36.49 |  | 0.28 | 0.45 | ↓ | 0.95 | 58.81 |  |
| RBMX | RNA binding motif protein, X-linked | 213762_x_at | 0.78 | 22.56 |  | 0.83 | 29.01 |  | 0.46 | 0.00 | ↓ |
| EZR | ezrin | 208621_s_at | 0.78 | 47.41 |  | 0.85 | 58.42 |  | 0.36 | 3.36 | ↓ |
| BZW1 | basic leucine zipper and W2 domains 1 | 200776_s_at | 0.78 | 28.36 |  | 0.49 | 1.45 | ↓ | 1.05 | 57.14 |  |
| MYCBP2 | MYC binding protein 2 | 1557370_s_at | 0.78 | 30.22 |  | 0.42 | 0.84 | ↓ | 1.41 | 20.38 |  |
| TNRC6B | Trinucleotide repeat containing 6B | 240044_x_at | 0.78 | 30.22 |  | 0.28 | 0.25 | ↓ | 0.99 | 54.08 |  |
| TRO | trophinin | 205028_at | 0.78 | 22.56 |  | 0.48 | 3.81 | ↓ | 0.31 | 0.72 | ↓ |
| IAH1 | isoamyl acetate-hydrolyzing esterase 1 homolog (S. cerevisiae) | 230621_at | 0.78 | 26.15 |  | 0.44 | 0.60 | ↓ | 0.85 | 32.58 |  |
| RPL37A | ribosomal protein L37a | 213459_at | 0.78 | 36.49 |  | 1.21 | 31.53 |  | 0.41 | 7.00 | ↓ |
| TMEM100 | transmembrane protein 100 | 219230_at | 0.77 | 42.20 |  | 1.15 | 54.70 |  | 0.46 | 4.16 | ↓ |
| AFF4 | AF4/FMR2 family, member 4 | 243487_at | 0.77 | 40.42 |  | 0.42 | 3.35 | ↓ | 1.52 | 35.63 |  |
| AK1 | adenylate kinase 1 | 202587_s_at | 0.77 | 38.54 |  | 0.44 | 3.81 | ↓ | 0.89 | 50.73 |  |
| SRSF6 | serine/arginine-rich splicing factor 6 | 206108_s_at | 0.77 | 40.42 |  | 4.21 | 1.26 | ↑ | 1.11 | 58.81 |  |
| DCUN1D1 | DCN1, defective in cullin neddylation 1, domain containing 1 (S. cerevisiae) | 242428_at | 0.77 | 34.35 |  | 2.72 | 7.87 | ↑ | 0.83 | 52.56 |  |
| UBE2Z | ubiquitin-conjugating enzyme E2Z | 222395_s_at | 0.77 | 22.56 |  | 0.44 | 0.53 | ↓ | 0.90 | 38.27 |  |
| MAGI2-IT | MAGI2 intronic transcript (non-protein coding) | 1560526_at | 0.77 | 42.20 |  | 0.37 | 2.30 | ↓ | 1.28 | 46.35 |  |
| THOC4 | THO complex 4 | 226319_s_at | 0.77 | 24.35 |  | 2.10 | 6.11 | ↑ | 0.64 | 3.36 |  |
| SCARA3 | scavenger receptor class A, member 3 | 223842_s_at | 0.77 | 26.15 |  | 0.32 | 0.18 | ↓ | 0.39 | 1.02 | ↓ |
| GIPC2 | GIPC PDZ domain containing family, member 2 | 219970_at | 0.77 | 28.36 |  | 0.91 | 43.50 |  | 4.77 | 0.50 | ↑ |
| RDH10 | retinol dehydrogenase 10 (all-trans) | 227467_at | 0.77 | 47.41 |  | 1.38 | 60.46 |  | 3.03 | 8.02 | ↑ |
| PSG3 | pregnancy specific beta-1-glycoprotein 3 | 215821_x_at | 0.77 | 32.33 |  | 0.47 | 2.80 | ↓ | 4.78 | 8.02 | ↑ |
| INPP4B | inositol polyphosphate-4-phosphatase, type II, 105kDa | 205376_at | 0.77 | 34.35 |  | 0.90 | 47.13 |  | 2.15 | 2.43 | ↑ |
| CD164 | CD164 molecule, sialomucin | 208653_s_at | 0.77 | 28.36 |  | 0.43 | 0.70 | ↓ | 0.65 | 8.02 |  |
| RNGTT | RNA guanylyltransferase and 5'-phosphatase | 204207_s_at | 0.77 | 26.15 |  | 0.44 | 0.70 | ↓ | 1.08 | 55.52 |  |
| DLEU2 | deleted in lymphocytic leukemia 2 (non-protein coding) | 1556820_a_at | 0.77 | 42.20 |  | 0.57 | 19.82 |  | 0.32 | 2.43 | ↓ |
| C9orf150 | chromosome 9 open reading frame 150 | 227443_at | 0.77 | 32.33 |  | 1.14 | 43.50 |  | 0.46 | 2.43 | ↓ |
| RBFA | ribosome binding factor A (putative) | 243001_at | 0.77 | 18.98 |  | 0.66 | 12.66 |  | 0.46 | 4.16 | ↓ |
| TFPI2 | tissue factor pathway inhibitor 2 | 209277_at | 0.77 | 38.54 |  | 0.60 | 31.53 |  | 0.13 | 0.00 | ↓ |
| C1orf144 | chromosome 1 open reading frame 144 | 212003_at | 0.77 | 34.35 |  | 0.47 | 1.98 | ↓ | 0.88 | 46.35 |  |
| SERTAD4 | SERTA domain containing 4 | 229674_at | 0.77 | 26.15 |  | 5.69 | 2.30 | ↑ | 0.28 | 0.58 | ↓ |
| ARL15 | ADP-ribosylation factor-like 15 | 219842_at | 0.77 | 24.35 |  | 2.42 | 0.60 | ↑ | 1.54 | 13.22 |  |
| SFRS3 | Splicing factor, arginine/serine-rich 3 | 232392_at | 0.77 | 28.36 |  | 0.42 | 1.26 | ↓ | 0.40 | 0.89 | ↓ |
| KLRAQ1 | KLRAQ motif containing 1 | 1554145_a_at | 0.77 | 20.86 |  | 0.39 | 0.25 | ↓ | 0.89 | 35.63 |  |
| ASAH2B | N-acylsphingosine amidohydrolase (non-lysosomal ceramidase) 2B | 231791_at | 0.77 | 48.99 |  | 0.26 | 1.74 | ↓ | 0.68 | 38.27 |  |
| IL6ST | interleukin 6 signal transducer (gp130, oncostatin M receptor) | 212196_at | 0.77 | 24.35 |  | 0.35 | 0.25 | ↓ | 1.32 | 26.48 |  |
| TM9SF3 | Transmembrane 9 superfamily member 3 | 228610_at | 0.77 | 18.98 |  | 0.22 | 0.53 | ↓ | 1.62 | 11.20 |  |
| CEP70 | centrosomal protein 70kDa | 224150_s_at | 0.77 | 18.98 |  | 0.80 | 22.17 |  | 0.34 | 0.00 | ↓ |
| TMED4 | transmembrane emp24 protein transport domain containing 4 | 224676_at | 0.77 | 30.22 |  | 0.31 | 0.25 | ↓ | 0.84 | 38.27 |  |
| KIAA1632 | KIAA1632 | 236108_at | 0.77 | 26.15 |  | 0.20 | 0.53 | ↓ | 1.70 | 13.22 |  |
| DSP | desmoplakin | 200606_at | 0.77 | 26.15 |  | 1.68 | 14.26 |  | 2.19 | 4.84 | ↑ |
| PTPN20A /// PTPN20B | protein tyrosine phosphatase, non-receptor type 20A /// protein tyrosine phosphatase, non-receptor type 20B | 215172_at | 0.76 | 36.49 |  | 1.18 | 49.04 |  | 0.48 | 4.84 | ↓ |
| NBN | nibrin | 202906_s_at | 0.76 | 28.36 |  | 1.67 | 14.26 |  | 0.45 | 1.61 | ↓ |
| SNAP23 | synaptosomal-associated protein, 23kDa | 209131_s_at | 0.76 | 24.35 |  | 0.28 | 0.00 | ↓ | 1.43 | 17.70 |  |
| ITGA2 | integrin, alpha 2 (CD49B, alpha 2 subunit of VLA-2 receptor) | 205032_at | 0.76 | 32.33 |  | 0.29 | 0.40 | ↓ | 2.22 | 8.02 | ↑ |
| NT5C2 | 5'-nucleotidase, cytosolic II | 209155_s_at | 0.76 | 34.35 |  | 1.22 | 36.20 |  | 2.23 | 4.84 | ↑ |
| ANKRD29 | ankyrin repeat domain 29 | 238332_at | 0.76 | 54.53 |  | 0.56 | 31.53 |  | 2.17 | 8.02 | ↑ |
| MCCC2 | methylcrotonoyl-CoA carboxylase 2 (beta) | 209624_s_at | 0.76 | 26.15 |  | 0.40 | 0.60 | ↓ | 1.23 | 43.83 |  |
| PRMT2 | protein arginine methyltransferase 2 | 228725_x_at | 0.76 | 18.98 |  | 0.48 | 0.76 | ↓ | 1.04 | 57.92 |  |
| COL8A1 | collagen, type VIII, alpha 1 | 221152_at | 0.76 | 62.00 |  | 0.18 | 1.45 | ↓ | 0.84 | 58.81 |  |
| EMP1 | epithelial membrane protein 1 | 213895_at | 0.76 | 45.69 |  | 0.40 | 4.45 | ↓ | 1.73 | 17.70 |  |
| EXD2 | exonuclease 3'-5' domain containing 2 | 1555808_a_at | 0.76 | 26.15 |  | 0.25 | 0.18 | ↓ | 0.93 | 46.35 |  |
| PRSS12 | protease, serine, 12 (neurotrypsin, motopsin) | 205515_at | 0.76 | 58.62 |  | 0.38 | 29.01 |  | 4.70 | 4.16 | ↑ |
| KIAA0802 | KIAA0802 | 240448_at | 0.76 | 22.56 |  | 0.23 | 0.70 | ↓ | 0.85 | 46.35 |  |
| CABYR | calcium binding tyrosine-(Y)-phosphorylation regulated | 219928_s_at | 0.76 | 24.35 |  | 0.43 | 0.98 | ↓ | 1.06 | 55.52 |  |
| CDK6 | cyclin-dependent kinase 6 | 235287_at | 0.76 | 24.35 |  | 0.36 | 0.76 | ↓ | 2.65 | 1.61 | ↑ |
| CHD9 | chromodomain helicase DNA binding protein 9 | 235388_at | 0.76 | 30.22 |  | 2.28 | 2.80 | ↑ | 0.61 | 7.00 |  |
| APLP2 | Amyloid beta (A4) precursor-like protein 2 | 228520_s_at | 0.76 | 32.33 |  | 2.07 | 4.45 | ↑ | 1.08 | 58.81 |  |
| NDFIP2 | Nedd4 family interacting protein 2 | 224802_at | 0.76 | 30.22 |  | 1.20 | 41.29 |  | 2.07 | 1.61 | ↑ |
| SCARB2 | scavenger receptor class B, member 2 | 201647_s_at | 0.76 | 26.15 |  | 0.29 | 0.18 | ↓ | 1.00 | 54.08 |  |
| SMC3 | structural maintenance of chromosomes 3 | 209258_s_at | 0.76 | 24.35 |  | 2.61 | 0.53 | ↑ | 0.73 | 11.20 |  |
| CIAO1 | cytosolic iron-sulfur protein assembly 1 | 203536_s_at | 0.76 | 18.98 |  | 0.48 | 1.74 | ↓ | 0.95 | 50.73 |  |
[truncated: 109,181 more chars]
